# Combinatorial control of *Pseudomonas aeruginosa* biofilm development by quorum-sensing and nutrient-sensing regulators

**DOI:** 10.1128/msystems.00372-24

**Published:** 2024-08-14

**Authors:** Gong Chen, Georgia Fanouraki, Aathmaja Anandhi Rangarajan, Bradford T. Winkelman, Jared T. Winkelman, Christopher M. Waters, Sampriti Mukherjee

**Affiliations:** 1 Department of Molecular Genetics & Cell Biology, The University of Chicago, Chicago, Illinois, USA; 2 Department of Microbiology and Molecular Genetics, Michigan State University, East Lansing, Michigan, USA; 3 Trestle, LLC, Milwaukee, Wisconsin, USA; National Cancer Institute, Bethesda, Maryland, USA

**Keywords:** quorum sensing, catabolite repression, biofilms, Crc, RhlR, CbrA, nutrient sensing

## Abstract

The human pathogen *Pseudomonas aeruginosa*, a leading cause of hospital-acquired infections, inhabits and forms sessile antibiotic-resistant communities called biofilms in a wide range of biotic and abiotic environments. In this study, we examined how two global sensory signaling pathways—the RhlR quorum-sensing system and the CbrA/CbrB nutritional adaptation system—intersect to control biofilm development. Previous work has shown that individually these two systems repress biofilm formation. Here, we used biofilm analyses, RNA-seq, and reporter assays to explore the combined effect of information flow through RhlR and CbrA on biofilm development. We find that the Δ*rhlR*Δ*cbrA* double mutant exhibits a biofilm morphology and an associated transcriptional response distinct from wildtype and the parent Δ*rhlR* and Δ*cbrA* mutants indicating codominance of each signaling pathway. The Δ*rhlR*Δ*cbrA* mutant gains suppressor mutations that allow biofilm expansion; these mutations map to the *crc* gene resulting in loss of function of the carbon catabolite repression protein Crc. Furthermore, the combined absence of RhlR and CbrA leads to a drastic reduction in the abundance of the Crc antagonist small RNA CrcZ. Thus, CrcZ acts as the molecular convergence point for quorum- and nutrient-sensing cues. We find that in the absence of antagonism by CrcZ, Crc promotes the expression of biofilm matrix components—Pel exopolysaccharide, and CupB and CupC fimbriae. Therefore, this study uncovers a regulatory link between nutritional adaption and quorum sensing with potential implications for anti-biofilm targeting strategies.

**IMPORTANCE:**

Bacteria often form multicellular communities encased in an extracytoplasmic matrix called biofilms. Biofilm development is controlled by various environmental stimuli that are decoded and converted into appropriate cellular responses. To understand how information from two distinct stimuli is integrated, we used biofilm formation in the human pathogen *Pseudomonas aeruginosa* as a model and studied the intersection of two global sensory signaling pathways—quorum sensing and nutritional adaptation. Global transcriptomics on biofilm cells and reporter assays suggest parallel regulation of biofilms by each pathway that converges on the abundance of a small RNA antagonist of the carbon catabolite repression protein, Crc. We find a new role of Crc as it modulates the expression of biofilm matrix components in response to the environment. These results expand our understanding of the genetic regulatory strategies that allow *P. aeruginosa* to successfully develop biofilm communities.

## INTRODUCTION

Bacteria predominantly exist in structured communities called biofilms. Biofilms are defined as aggregates of cells that are embedded in a matrix made of extracellular polymeric substances (EPS) including exopolysaccharides, proteins, lipids, and nucleic acids (1–3). The EPS is crucial for the emergent properties of biofilms such as superior resilience to environmental stresses like antimicrobials and host immune responses. Biofilm formation is a dynamic process that is governed by various intracellular and exogenous stimuli. Often, two-component signaling (TCS) systems integrate and relay the information contained in sensory cues into the control of biofilm formation (4, 5). TCSs are typically composed of sensor histidine kinases (HK) and partner response regulators (RR) that, via phosphorylation cascades, couple stimulus sensing to appropriate changes in behavior (4, 6).

**Fig 1:**
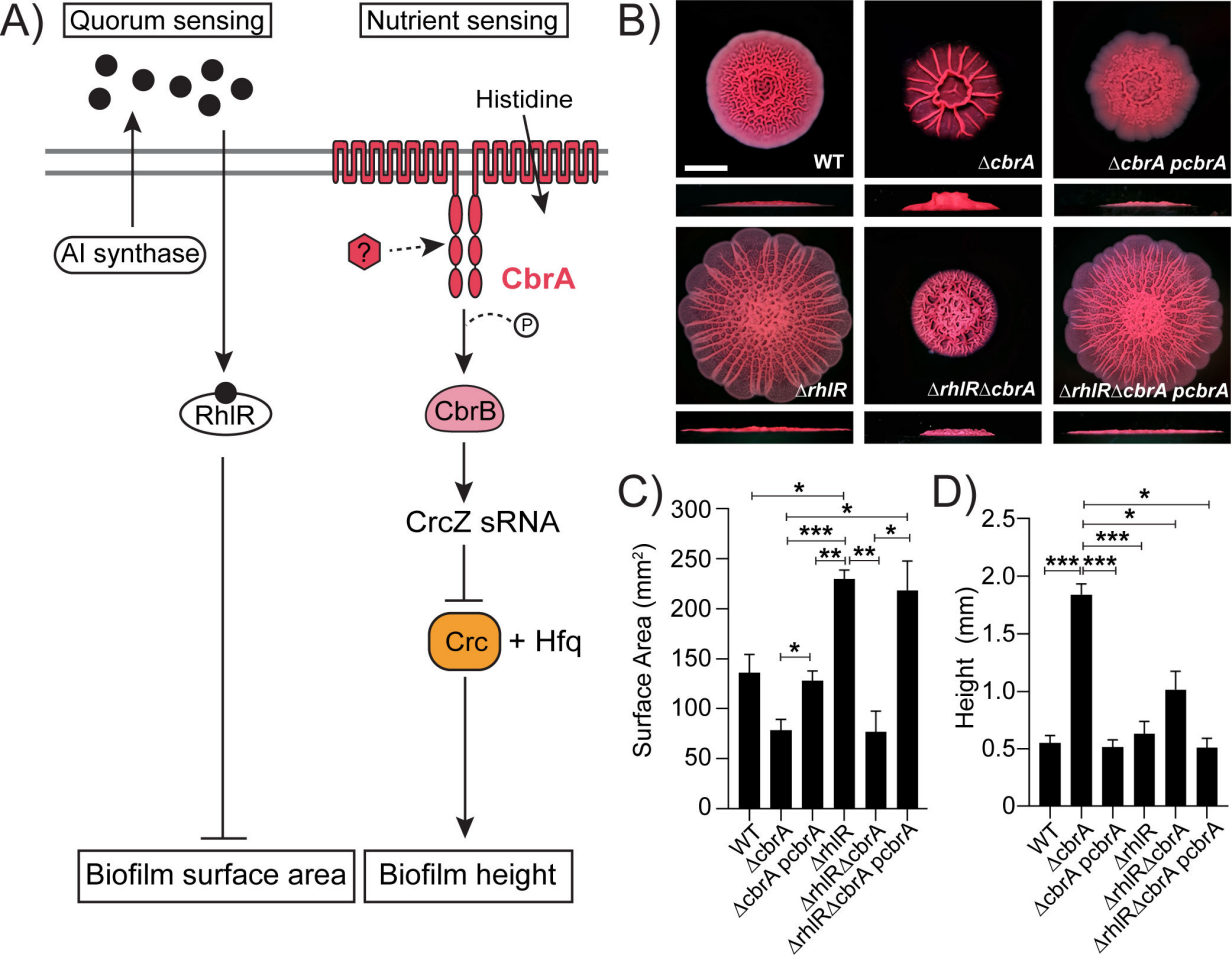
*P. aeruginosa* Δ*cbrA* and Δ*rhlR* mutants have distinct hyper-rugose biofilm phenotypes. (**A**) Schematic of the RhlR quorum sensing and CbrA nutrient sensing pathways. The two gray horizontal lines represent the cytoplasmic membrane, black circles represent autoinducer sensed by RhlR, red hexagon represents the unknown signal that activates CbrA sensor kinase. CbrA also functions as a histidine transporter, but its kinase function appears to be independent of histidine transport (17). (**B**) Colony biofilm phenotypes of WT PA14 and the designated mutants on Congo red agar medium after 120 h of growth. Scale bar, 5 mm. (**C**) Colony biofilm surface area quantitation for the indicated strains after 120 h of growth. Error bars represent standard deviation of three independent experiments. (**D**) Colony biofilm height quantitation for the indicated strains after 120 h of growth. Error bars represent standard deviation of three independent experiments. (**C and D**) Only pairwise comparisons that had *P* value < 0.05 are denoted. Statistical significance was determined using Welch’s ANOVA with Dunnett’s T3 multiple comparisons test in GraphPad Prism software. ***P < 0.001, **P < 0.01, *P < 0.05.

The opportunistic pathogen *Pseudomonas aeruginosa* forms biofilms in diverse environments, such as soil and water and in host-associated environments, such as burn wounds, lungs of cystic fibrosis (CF) patients, and plant tissues (7–10). Accordingly, *P. aeruginosa* encodes a large suite of >60 TCS systems that allow it to respond to diverse external cues (11–13). One such TCS is the CbrA/CbrB system that is involved in nutritional adaptation and hierarchical utilization of various carbon sources (14–17). CbrA represents a non-canonical sensor HK that also functions as a histidine transporter (17). CbrA has two putative sensor domains—SLC5 and PAS, yet the sensory stimulus activating its kinase is unknown. CbrA kinase phosphorylates its cognate RR CbrB that activates the expression of the small RNA CrcZ (17, 18; Fig. 1A). CrcZ sequesters complexes of the catabolite repression protein Crc with Hfq to antagonize Crc function in the carbon catabolite repression (CCR) process termed “reverse diauxie” or reverse CCR (rCCR; 19, 20). In the presence of preferred carbon sources, CrcZ-mediated antagonism is relieved and Crc/Hfq post-transcriptionally inhibits the expression of enzymes involved in the catabolism of non-preferred carbon sources. In addition to the metabolic regulation of carbon and nitrogen utilization in *P. aeruginosa*, Cbr TCS plays an important role in various virulence-associated processes including biofilm formation and antibiotic resistance (15).

Another sensory cue commonly detected by bacteria is their population density via the chemical communication process called quorum sensing (21). Quorum sensing relies on the production of extracellular signaling molecules called autoinducers and their subsequent detection by cognate receptors (21, 22) and thereby allows groups of bacteria to coordinate their gene expression patterns in response to changes in population density to exhibit collective behaviors. Quorum sensing controls biofilm development and virulence in *P. aeruginosa* (23–25). The *P. aeruginosa* quorum-sensing circuit consists of two canonical LuxI/R pairs: LasI/R and RhlI/R (26–28). LasI produces and LasR responds to the autoinducer 3OC12-homoserine lactone (3OC12-HSL), while RhlR binds to the autoinducer C4-HSL, the product of RhlI. While LasR promotes biofilm formation (23, 29), RhlR represses biofilms (25; Fig. 1A).

Bacteria encounter multiple sensory cues simultaneously and must integrate information from each cue into a response. In this study, we examine how the CbrA/CbrB signaling pathway intersects with the RhlR-dependent quorum sensing system to control biofilm development. We find that the combined absence of quorum sensing via RhlR and nutrient sensing via CbrA results in (i) a biofilm morphology and an associated transcriptional response that indicates codominance of the RhlR and CbrA/CbrB pathways and (ii) a significant reduction in the levels of the small RNA CrcZ. We further find that the Δ*rhlR*Δ*cbrA* mutant gives rise to spontaneous suppressor mutations in the *crc* gene that allows biofilm spreading. Transcriptomic profiling of biofilms of wildtype (WT) and mutant *P. aeruginosa* strains and reporter assays demonstrate that Crc promotes the expression of Pel exopolysaccharide and Cup fimbriae components of the biofilm matrix. Thus, our work uncovers a new role for Crc as a master regulator of biofilm formation that allows the integration of quorum and nutritional cues.

## RESULTS

### CbrA-mediated nutrient-sensing and RhlR-mediated quorum-sensing codominantly control biofilm development

To study the role of the CbrA/CbrB TCS in *P. aeruginosa* biofilm formation, we first generated in-frame marker-less deletion of the *cbrA* gene in the WT *P. aeruginosa* UCBPP-PA14 (hereafter called PA14) background. PA14 exhibits a characteristic rugose-center/smooth-periphery colony biofilm phenotype on Congo red biofilm medium, while the Δ*cbrA* mutant exhibits a distinct colony biofilm phenotype with decreased surface area coverage and increased height, defined as the maximum vertical rise from the base of the colony, when compared to WT (Fig. 1B through D; Fig. S1). To uncouple the two functions of CbrA—HK and histidine transport (30–32)—and determine whether biofilm regulation is a consequence of information flow from CbrA HK to CbrB RR, we generated a Δ*cbrB* mutant. The Δ*cbrB* mutant produces hyper-rugose biofilms identical to the Δ*cbrA* mutant (Fig. S2). In addition, we built a *cbrA^H766A^* mutant where the histidine residue that undergoes autophosphorylation is changed to alanine to abolish kinase function of CbrA. Similar to the Δ*cbrB* strain, the *cbrA^H766A^* mutant has a biofilm phenotype that resembles the Δ*cbrA* mutant (Fig. S2). Introduction of a plasmid expressing *cbrA* under its native promoter to the Δ*cbrA* and *cbrA^H766A^* mutants restored biofilm formation to WT levels (Fig. 1B through D; Fig. S2). These analyses indicate that CbrA kinase activity is required for the repression of biofilm development in *P. aeruginosa*.

**Fig 2:**
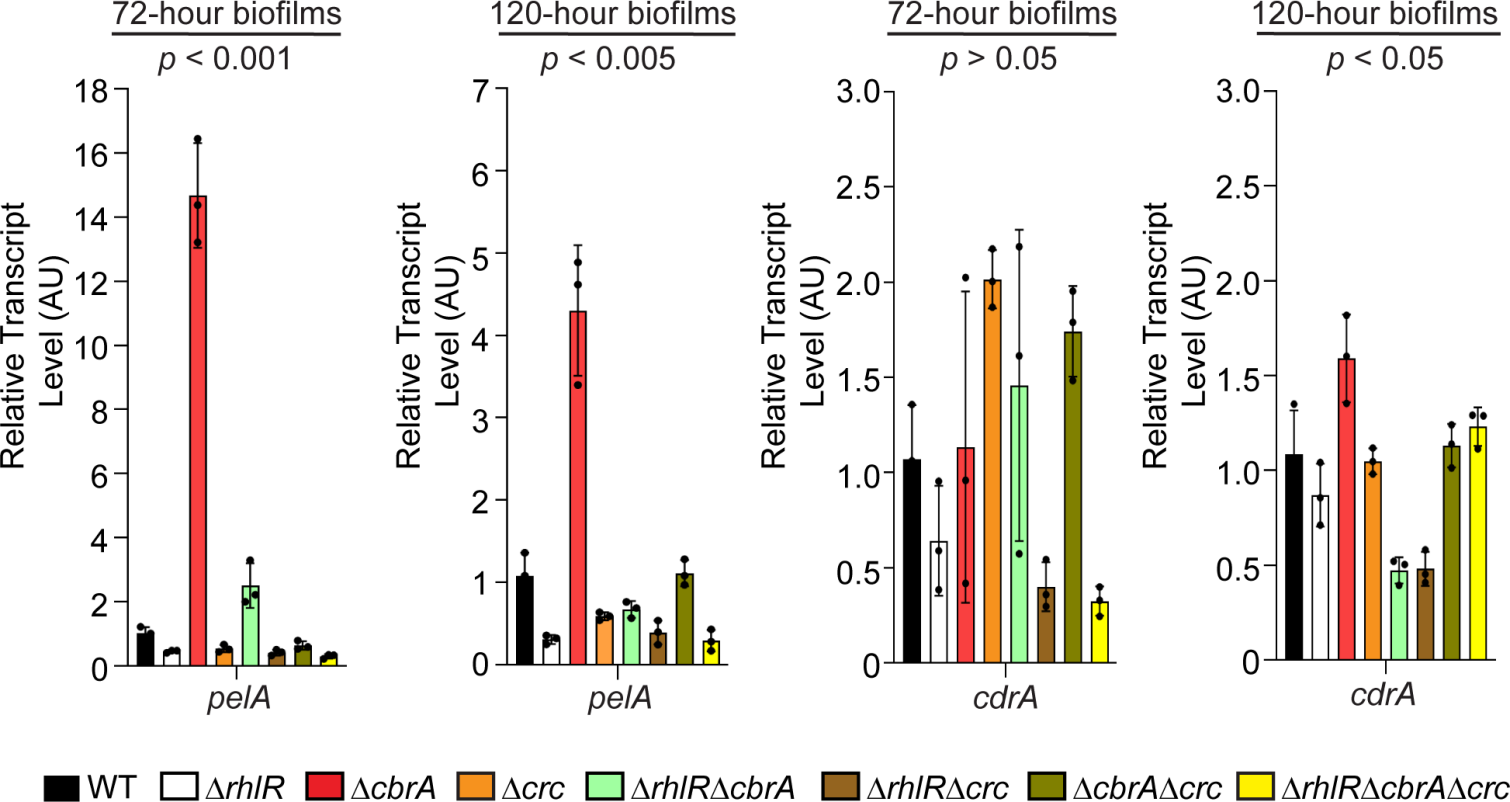
*P. aeruginosa* Δ*cbrA* mutant exhibits increased *pel* expression in biofilms. Relative expression of *pelA* and *cdrA* genes normalized to 16S RNA, *ostA,* and *rpsO* transcript levels in WT PA14 and indicated mutants after 72 and 120 h of colony biofilm growth. AU denotes arbitrary units. Error bars represent standard deviation of three biological replicates. Statistical significance was determined using Welch’s ANOVA in GraphPad Prism software. For *pelA* expression at 72 h, the following comparisons were statistically significant (*P* < 0.001): WT vs Δ*rhlR*, and Δ*cbrA* vs all other strains. For *pelA* expression at 120 h, the following comparisons were statistically significant (*P* < 0.005): WT vs Δ*rhlR*, and Δ*cbrA* vs all other strains. For *cdrA* expression at 120 h, the following comparisons were statistically significant (*P* < 0.05): Δ*cbrA* vs Δ*rhlR*Δ*cbrA* and Δ*rhlR*Δ*crc* mutants.

The production of EPS is a defining feature of biofilms, and the matrix composition can vary depending on the bacterial species. *P. aeruginosa* biofilm matrix has been reported to be composed of three different exopolysaccharides—Pel, Psl, and alginate—depending on the strain (reviewed in reference 33). In PA14, Pel is the major exopolysaccharide under commonly used laboratory growth conditions (34). Pel biosynthetic enzymes are encoded by the *pelABCDEFG* operon (*pel*). In addition, the matrix protein CdrA interacts with Pel exopolysaccharide and promotes structural stability in biofilm aggregates (35). Here, we assessed the expression of *pelA* and *cdrA* in the biofilms of WT and Δ*cbrA* mutant using quantitative RT-PCR. We find that *pelA* transcript levels are increased by 15- and 5-fold in the Δ*cbrA* mutant compared to WT at 72 h and 120 h timepoints, respectively (Fig. 2). In contrast, *cdrA* transcript levels do not exhibit any significant differences between WT and the Δ*cbrA* mutant (Fig. 2). Next, we assessed the contribution of Pel to the Δ*cbrA* mutant biofilms by comparing the Δ*pelA* and Δ*cbrA*Δ*pelA* double mutants in biofilm assays. The absence of PelA abolished the formation of colony biofilms and pellicles (Fig. S3 and S4). Both the Δ*pelA* and Δ*cbrA*Δ*pelA* mutants had completely smooth colony morphologies and lower biofilm height compared to WT (Fig. S4A through C). Furthermore, to test the generality of these observations, we employed a solid surface-associated (SSA) biofilm assay using polystyrene multi-well plates and found that the Δ*pelA* and Δ*cbrA*Δ*pelA* mutants showed significantly less crystal violet staining compared to the Δ*cbrA* mutant (Fig. S4D). Thus, CbrA represses *pel* expression, and Pel exopolysaccharide is required for the Δ*cbrA* mutant to exhibit its characteristic hyper-rugose biofilm phenotype.

We have previously reported that the *P. aeruginosa* quorum-sensing receptor RhlR represses colony biofilm development and the hyper-rugosity conferred by the absence of RhlR requires Pel exopolysaccharide (Fig. 1; 25). Our quantitative RT-PCR analyses on biofilm samples demonstrate that RhlR promotes the expression of *pelA* as the Δ*rhlR* mutant showed fourfold reduction in *pelA* transcript levels compared to WT in mature biofilms (Fig. 2). Intriguingly, while both Δ*rhlR* and Δ*cbrA* mutants appear to be hyper-rugose compared to WT, the Δ*cbrA* mutant exhibits increased biofilm height and higher levels of *pel* expression as opposed to the increased surface area coverage and decreased *pel* transcript levels of the Δ*rhlR* mutant (Fig. 1B through D and 2; Fig. S1). To explore the combined effect of Rhl and Cbr pathways on biofilm formation, we deleted *rhlR* and *cbrA* genes together. The Δ*rhlR*Δ*cbrA* double mutant showed a growth profile indistinguishable from WT and its parent Δ*rhlR* and Δ*cbrA* mutants but formed colony biofilms that were markedly distinct from those of the WT and single Δ*rhlR* and Δ*cbrA* mutants, i.e., had significantly lower surface coverage than Δ*rhlR* mutant and lower height than Δ*cbrA* mutant (Fig. 1B through D; Fig. S1). Furthermore, the Δ*rhlR*Δ*cbrA* double mutant showed decreased *pel* expression compared to the Δ*cbrA* mutant (Fig. 2). Introduction of a plasmid expressing *cbrA* under its native promoter to the Δ*rhlR*Δ*cbrA* double mutant resulted in a hyper-rugose biofilm that resembles the Δ*rhlR* mutant in terms of biofilm morphology, surface area, and height (Fig. 1B through D). We conclude that RhlR and CbrA control different properties, surface area coverage and vertical rise, respectively, of a growing biofilm.

### Loss-of-function mutations in *crc* affects biofilm development

**Fig 3:**
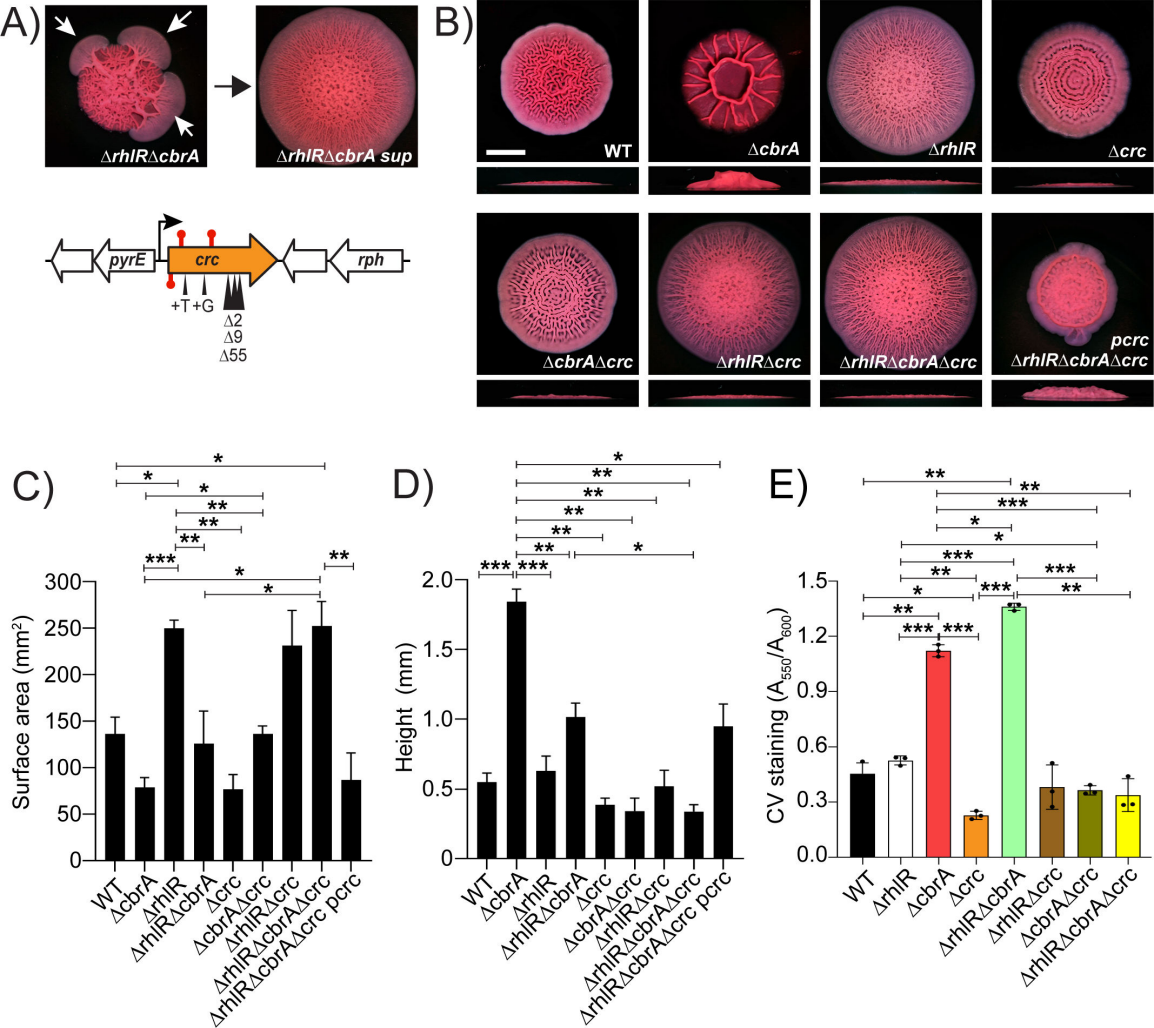
*P. aeruginosa* Δ*rhlR*Δ*cbrA* mutant biofilms generate spontaneous mutations that allow biofilm expansion. (**A**) (Top) Shown is a representative isolation of a suppressor mutation of the Δ*rhlR*Δ*cbrA* biofilm phenotype. The white arrows in the left panel indicate flares radiating from the biofilm diagnostic of the emergence of suppressor mutations. The right panel shows the biofilm phenotype of a mutant following isolation. (Bottom) Chromosomal arrangement of the *crc* (orange) gene. Large white arrows represent open reading frames (lengths not to scale), black bent arrow indicates promoter, red stem-loops indicate STOP mutations, and black triangles indicate the locations of insertion and deletion suppressor mutations. (**B**) Colony biofilm phenotypes of WT PA14 and the designated mutants on Congo red agar medium after 120 h of growth. Scale bar, 5 mm. (**C**) Colony biofilm surface area quantitation for the indicated strains after 120 h of growth. Error bars represent the standard deviation of three independent experiments. (**D**) Colony biofilm height quantitation for the indicated strains after 120 h of growth. Error bars represent the standard deviation of three independent experiments. (**E**) Biofilm development assays followed by crystal violet staining for WT and indicated mutant strains. Error bars represent the standard deviation of three biological replicates. (**C–E**) Only pairwise comparisons that had *P* value < 0.05 are denoted. Statistical significance was determined using Welch’s ANOVA with Dunnett’s T3 multiple comparisons test in GraphPad Prism software. ***P < 0.001, **P < 0.01, *P < 0.05.

The Δ*rhlR*Δ*cbrA* mutant gave rise to suppressor flares after 72 h of biofilm growth that allowed biofilm expansion similar to the Δ*rhlR* mutant (Fig. 3A; Fig. S5). We isolated 12 spontaneously arising suppressor mutants from Δ*rhlR*Δ*cbrA* colony biofilms; 10 suppressors contained deletions, insertions, or missense mutations in the *crc* gene, while the remaining 2 suppressors harbored mutations in the *rplP* and *rplC* genes that encode for 50S ribosomal protein L16 and 50S ribosomal protein L3, respectively (Fig. 3A; Table S1). We generated Δ*crc* single, Δ*rhlR*Δ*crc* and Δ*cbrA*Δ*crc* double, and Δ*rhlR*Δ*cbrA*Δ*crc* mutants and analyzed their biofilm-forming behaviors. The absence of Crc showed little effect in WT and Δ*rhlR* backgrounds but substantially altered the biofilms of the Δ*cbrA* and Δ*rhlR*Δ*cbrA* mutants (Fig. 3B through D). Specifically, the deletion of *crc* abolished the vertical rise of the Δ*cbrA* mutant colony biofilm (Fig. 3B through D) and led to increased surface area coverage of Δ*rhlR*Δ*cbrA* double mutant colony biofilms (Fig. 3B through D; Fig. S1). Introduction of a plasmid expressing *crc* under its native promoter to the Δ*rhlR*Δ*cbrA*Δ*crc* triple mutant resulted in colony biofilm phenotype identical to the Δ*rhlR*Δ*cbrA* double mutant validating our deletion analyses (Fig. 3B through D). We conclude that Crc promotes colony biofilm height when CbrA is inactive and restricts colony biofilm expansion when signal transduction via both RhlR and CbrA are absent.

To explore the extent of biofilm regulation by Crc, we analyzed the ability of WT and the Δ*crc* single and combination mutants to form SSA biofilms on polystyrene. Crystal violet staining of these SSA biofilms showed a significant increase in mature biofilm formation in the Δ*cbrA* and Δ*rhlR*Δ*cbrA* mutants compared to WT (Fig. 3E). We note that there was no significant difference between WT and Δ*rhlR* mutants in this assay. Nonetheless, the absence of Crc significantly decreased SSA biofilms in WT, as well as Δ*cbrA* and Δ*rhlR*Δ*cbrA* mutant backgrounds (Fig. 3E). Taken together, our data suggest a new role for Crc beyond reverse diauxie, i.e., Crc promotes biofilm development.

The absence of phenazines contributes to the colony biofilm spreading phenotype of the Δ*rhlR* mutant (25) and one known regulatory target of Crc is *phzM* that encodes for a key enzyme involved in the biosynthesis of the phenazine pyocyanin (36). Therefore, we assessed the contribution of phenazines to the Δ*rhlR*Δ*cbrA* double-mutant phenotype. We deleted the two phenazine biosynthesis operons *phzA1-G1* and *phzA2-G2* (Δ*phz*) in the WT, the Δ*cbrA* single-, and the Δ*rhlR*Δ*cbrA* double-mutant backgrounds and assayed pyocyanin production and biofilm development. As reported previously, the Δ*phz* mutant did not produce pyocyanin and its biofilm spread radially outward and covered more surface area than WT (Fig. S6A through C; 37). Mutation of the phenazine biosynthesis operons, however, failed to increase biofilm spreading in cells lacking CbrA (Fig. S6B and C). Likewise, there was no detectable change in biofilm morphology of the Δ*rhlR*Δ*cbrA*Δ*phz* mutant when compared with its parent Δ*rhlR*Δ*cbrA* strain (Fig. S6B and C). We infer that the absence of CbrA is epistatic to the absence of phenazines during colony biofilm development.

### CrcZ small RNA serves as the point of convergence for quorum and nutrient-sensing cues

**Fig 4:**
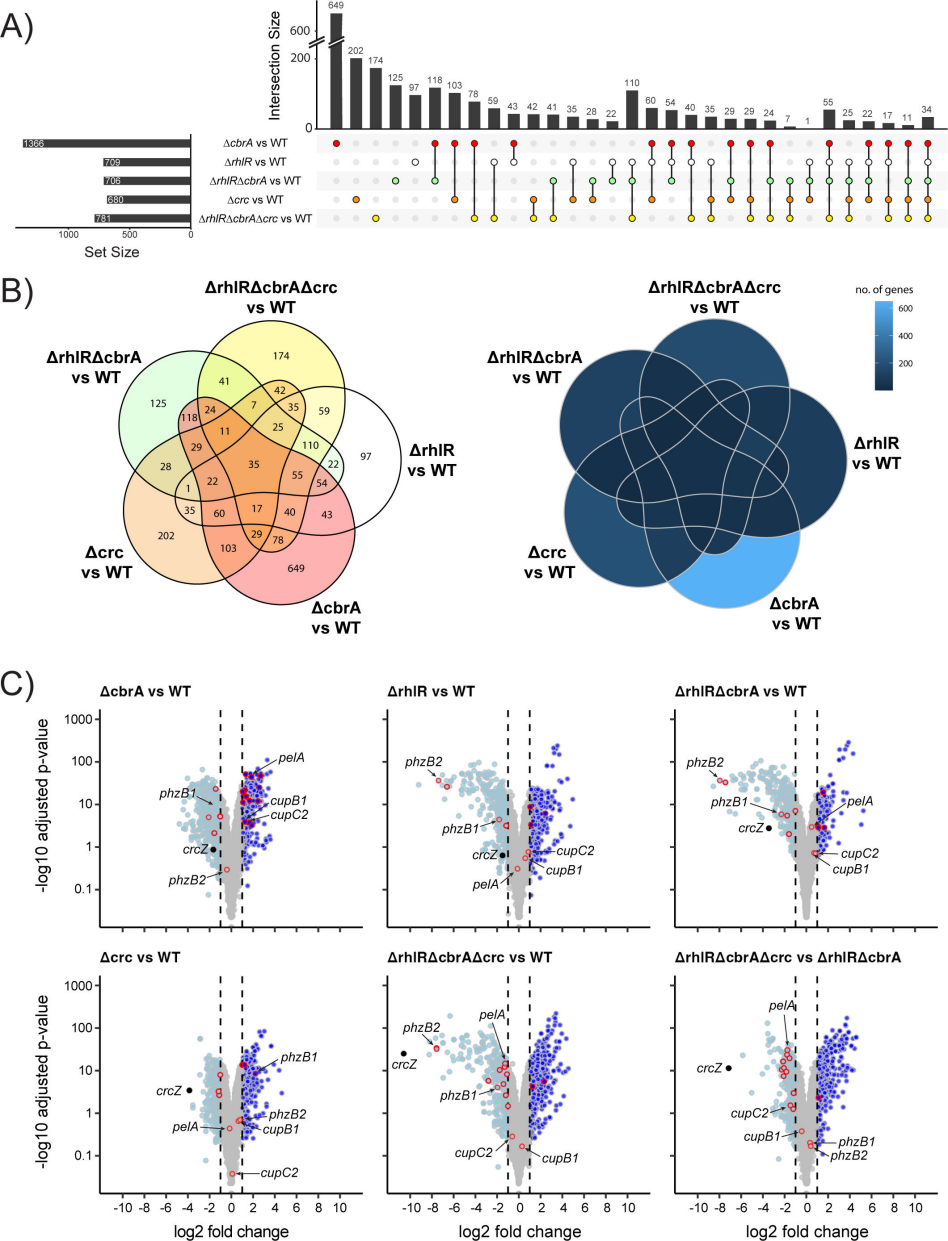
RNA-seq analysis of biofilms of PA14 and mutants. (**A**) Upset plot showing overlaps in genes that are differentially regulated in Δ*rhlR,* Δ*cbrA,* Δ*crc*, Δ*rhlR*Δ*cbrA* double, and Δ*rhlR*Δ*cbrA*Δ*crc* triple mutants compared to WT. Numbers on top of each vertical bar indicate number of genes differentially regulated in each intersection. Set size indicates the total number of genes that are significantly differentially regulated in a particular mutant when compared to WT. (**B**) Venn diagrams showing overlaps in genes that are differentially regulated in Δ*rhlR,* Δ*cbrA,* Δ*crc*, Δ*rhlR*Δ*cbrA* double, and Δ*rhlR*Δ*cbrA*Δ*crc* triple mutants compared to WT. Numbers (left) or shade of blue (right) indicate number of genes differentially regulated in each intersection. (**C**) Volcano plots of RNA-seq data for Δ*rhlR,* Δ*cbrA,* Δ*crc*, Δ*rhlR*Δ*cbrA,* and Δ*rhlR*Δ*cbrA*Δ*crc* compared to WT, and Δ*rhlR*Δ*cbrA*Δ*crc* compared to Δ*rhlR*Δ*cbrA*. Light blue solid circles with gray outlines represent genes with expression fold-changes ≤−2 and the *P* values (*P*adj), adjusted using the Benjamini-Hochberg procedure, <0.05; dark blue solid circles with gray outlines represent genes with expression fold-changes ≥ 2 and the *P* values (*P*adj), adjusted using the Benjamini-Hochberg procedure, <0.05. Gray solid circles represent genes with gene expression fold changes ≥−2 or ≤2 or *P*adj ≥ 0.05. Genes reported to be associated with biofilms are outlined in red.

To define the molecular basis underpinning the different Δ*rhlR* and Δ*cbrA* biofilm phenotypes, we used RNA-seq to compare the global transcriptional profiles of the biofilms of WT PA14 and the Δ*rhlR,* Δ*cbrA,* and Δ*rhlR*Δ*cbrA* double mutants grown on Congo red agar medium for 72 h. Principal component analysis (PCA) of normalized read counts for 5,978 genes showed that biofilm samples of each mutant clustered separately from WT (Fig. S7). Comparative transcriptomic analysis revealed a total of 1,366 differentially expressed genes (DEGs; genes with expression fold changes ≤−2 and ≥2, and the *P* values (Padj), adjusted using the Benjamini-Hochberg procedure, <0.05; see Table S3 in the supplemental material) in the Δ*cbrA* mutant (Fig. 4A and B). In the Δ*rhlR* mutant, we find a total of 709 DEGs, of which 326 transcripts overlapped with the CbrA regulon. Comparing the Δ*rhlR*Δ*cbrA* double to the Δ*cbrA* single mutant showed a 51.6% reduction in the number of DEGs in the double mutant (Fig. 4A and B). Furthermore, 125 genes were uniquely regulated in the Δ*rhlR*Δ*cbrA* mutant (Fig. 4A and B). We conclude that the combined absence of each regulator—CbrA and RhlR—gives rise to a transcriptomic response distinct from mere addition of their individual regulons.

Next, we determined the transcriptomic response to the absence of Crc. The Crc regulon consists of 680 genes, of which ~48% are also regulated by CbrA and ~36% coregulated by RhlR (Fig. 4A and B; Table S3). We note that, similar to the Δ*rhlR*Δ*cbrA* biofilm samples, a subset of genes was uniquely regulated in the Δ*rhlR*Δ*cbrA*Δ*crc* triple mutant, i.e., these 174 genes were not differentially expressed in the Δ*cbrA*, Δ*rhlR,* and Δ*crc* single mutant strains compared to WT (Fig. 4A and B). Furthermore, binning the DEGs according to their function defined by their PseudoCAP categories (38) suggests that RhlR functions downstream of CbrA mediated regulation of genes in categories such as “Biosynthesis of cofactors, prosthetic groups and carrier” and “Antibiotic resistance and susceptibility” (Fig. S8). Notably, in biofilms, Crc appears to both positively and negatively regulate several categories unrelated to carbon catabolite repression such as “Cell wall/LPS/Capsule,” “Protein secretion/export apparatus,” and “Adaptation, Protection” (Fig. S8). Taken together, the combined absence of RhlR, CbrA, and Crc results in a distinct regulon that does not overlap with the single mutants. We infer that CbrA, RhlR, and Crc regulons exhibit complex non-linear interactions.

**Fig 5:**
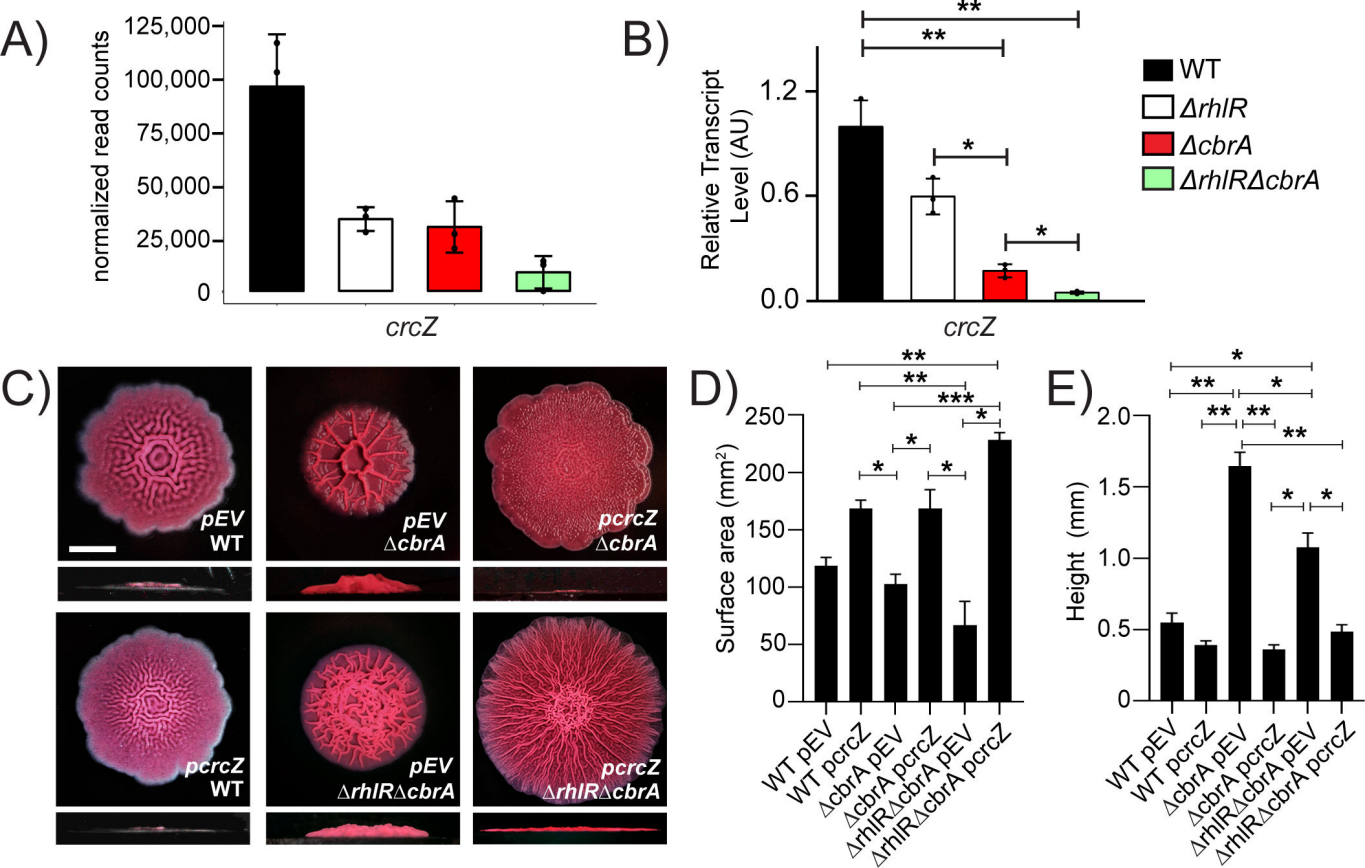
CrcZ small RNA allows integration of Rhl- and Cbr-signaling pathways and modulates biofilm development. (**A**) Normalized read counts obtained via Median Ratio Normalization (MRN) analysis for *crcZ* gene from RNA-seq run on biofilm samples of WT and indicated mutants. (**B**) Relative expression of *crcZ* gene normalized to 16S RNA, *ostA,* and *rpsO* transcript levels measured by qRT-PCR in WT PA14 and indicated mutants after 120 h of colony biofilm growth. AU denotes arbitrary units. Error bars represent the standard deviation of three biological replicates. (**C**) Colony biofilm phenotypes of WT PA14 and the designated mutants on Congo red agar medium after 120 h of growth. Scale bar, 5 mm. (**D**) Colony biofilm surface area quantitation for the indicated strains after 120 h of growth. Error bars represent the standard deviation of three independent experiments. (**E**) Colony biofilm height quantitation for the indicated strains after 120 h of growth. Error bars represent the standard deviation of three independent experiments. (**B, D, E**) Only pairwise comparisons that had *P* value < 0.05 are denoted. Statistical significance was determined using Welch’s ANOVA with Dunnett’s T3 multiple comparisons test in GraphPad Prism software. ***P < 0.001, **P < 0.01, *P < 0.05.

The expression of the small RNA CrcZ that antagonizes Crc to allow coordinated utilization of carbon sources is activated by CbrA/CbrB (Fig. 1A; 14). Thus, CrcZ levels are downregulated in the Δ*cbrA* mutant compared to WT (Fig. 4C). However, our RNA-seq data revealed a more severe reduction in CrcZ abundance in the Δ*rhlR*Δ*cbrA* double mutant compared to WT than in the Δ*rhlR* and Δ*cbrA* single mutants (Fig. 4C and 5A). Indeed, quantitative RT-PCR shows that *crcZ* transcript levels are ~6-fold lower in the Δ*cbrA* mutant than WT but 23-fold lower in the Δ*rhlR*Δ*cbrA* mutant compared to WT (Fig. 5B). Furthermore, consistent with previous report that CrcZ small RNA is stabilized by Crc in *Pseudomonas putida* (39), we find a significant reduction in CrcZ abundance in the Δ*crc* and Δ*rhlR*Δ*cbrA*Δ*crc* mutants compared to WT PA14 (Fig. 4C; Table S3). Thus, our data suggest that low levels of CrcZ promote biofilm morphology with reduced surface coverage and increased height.

To assess the contribution of CrcZ small RNA to biofilm development, we introduced a plasmid expressing *crcZ* from *Plac* promoter to WT PA14, the Δ*cbrA* single, and Δ*rhlR*Δ*cbrA* double mutants. Overexpression of CrcZ small RNA led to increased surface area coverage and a severe reduction in biofilm height in the Δ*cbrA* mutant compared to the empty vector control (Fig. 5C through E). Furthermore, overexpression of CrcZ small RNA in the Δ*rhlR*Δ*cbrA* double mutant resulted in a hyper-rugose biofilm that resembled the Δ*rhlR* mutant in terms of biofilm morphology, surface area, and height (Fig. 5C through E). We conclude that the abundance of CrcZ small RNA is a key determinant of biofilm development in response to signals transduced via CbrA and RhlR. We further conclude that drastic reduction in *crcZ* expression in the Δ*rhlR*Δ*cbrA* double mutant leads to uncontrolled Crc activity which, in turn, restricts surface area coverage and promotes height, likely explaining why *crc* suppressors arose in the Δ*rhlR*Δ*cbrA* mutant to allow biofilm expansion.

### Crc activates the expression of biofilm matrix components

Closer inspection of the transcriptomics data revealed downregulation of Pel biosynthesis genes such as *pelA* in the Δ*rhlR*Δ*cbrA*Δ*crc* triple mutant compared to WT and Δ*rhlR*Δ*cbrA* double mutant (Fig. 4C; Table S3), suggesting that Crc can be a positive regulator of Pel biosynthesis. Accordingly, we performed qRT-PCR for *pelA* from colony biofilm samples in the WT and a series of *crc* mutants—Δ*crc,* Δ*rhlR*Δ*crc,* Δ*cbrA*Δ*crc,* and Δ*rhlR*Δ*cbrA*Δ*crc*. We find that there is a twofold reduction in the relative abundance of *pelA* transcript in the Δ*crc* mutant compared to WT at 72 h and 120 h timepoints, respectively, and 27- and 8-fold reduction compared to the Δ*cbrA* strain at 72 h and 120 h timepoints, respectively (Fig. 2). Furthermore, the absence of Crc is epistatic to Δ*cbrA* as *pelA* transcript levels are drastically reduced in the Δ*cbrA*Δ*crc* and Δ*rhlR*Δ*cbrA*Δ*crc* mutants (Fig. 2). Thus, we conclude that Crc activates *pel* expression and the high expression of *pel* in the Δ*cbrA* and Δ*rhlR*Δ*cbrA* mutants is due to the unchecked activity of Crc in the absence of CrcZ small RNA.

**Fig 6:**
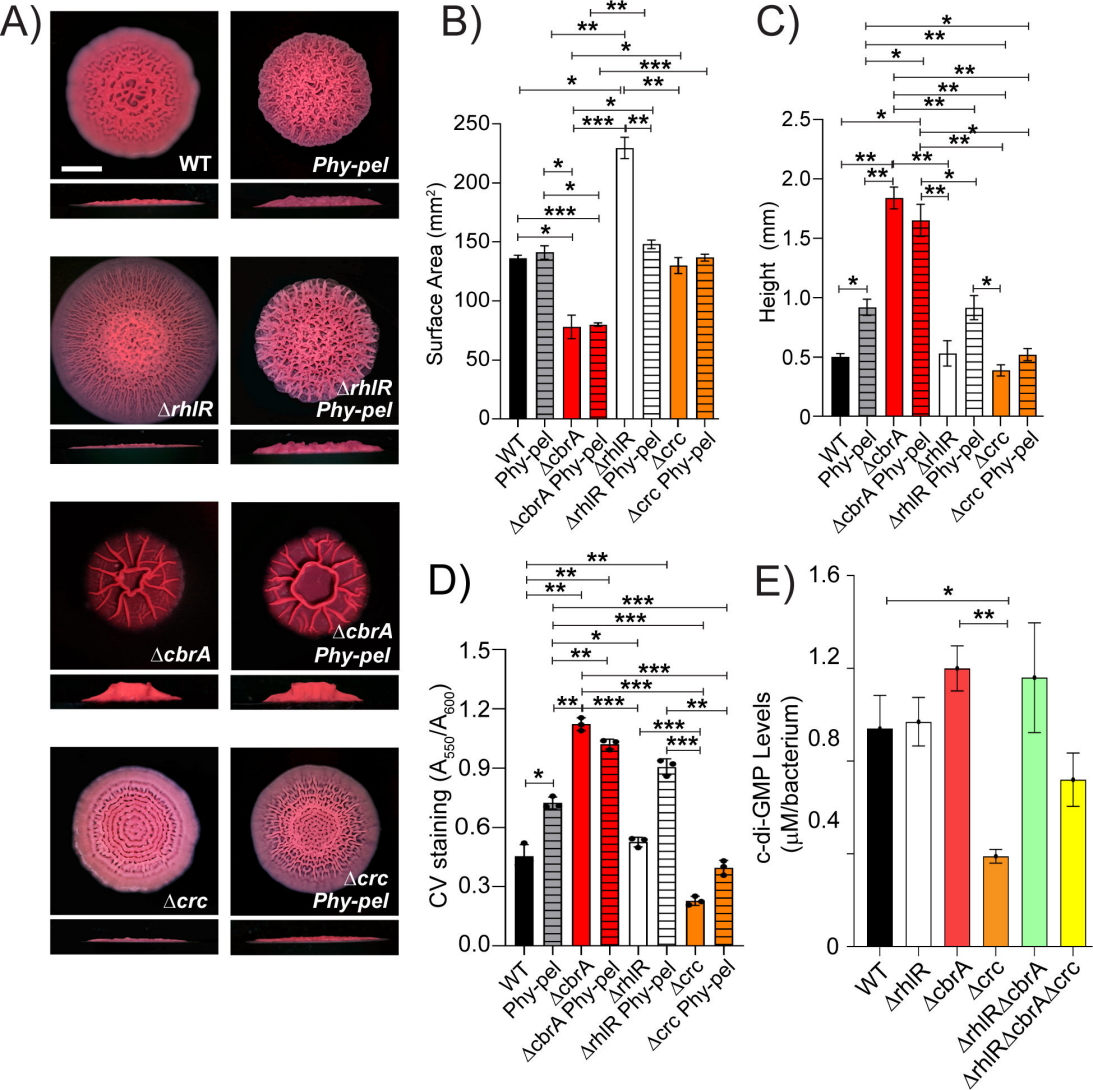
Crc-dependent increase in *pel* expression is post-transcriptional. (**A**) Colony biofilm phenotypes of WT PA14 and the designated mutants on Congo red agar medium after 120 h of growth. Scale bar, 5 mm. (**B**) Colony biofilm surface area quantitation for the indicated strains after 120 h of growth. Error bars represent the standard deviation of three independent experiments. (**C**) Colony biofilm height quantitation for the indicated strains after 120 h of growth. Error bars represent the standard deviation of three independent experiments. (**D**) Biofilm crystal violet staining assays for WT and indicated mutant strains. Error bars represent the standard deviation of three biological replicates. (**E**) c-di-GMP levels of the indicated strains. Error bars represent the standard deviation of three biological replicates. (**B–E**) Only pairwise comparisons that had *P* value < 0.05 are denoted. Statistical significance was determined using Welch’s ANOVA with Dunnett’s T3 multiple comparisons test in GraphPad Prism software. ***P < 0.001, **P < 0.01, *P < 0.05.

To determine whether different expression level of *pel* is involved in the distinct surface area and height of the Δ*rhlR* and Δ*cbrA* mutants, we generated a Pel overexpression strain where we replaced the native promoter of the *pelABCDEFG* operon (*PpelA*) with a constitutively expressed artificial promoter region comprised of transcription terminators T1 and T2 from the *Escherichia coli rrnB* gene followed by the *Bacillus subtilis Physpank* promoter (we call this strain *Phy-pel*). Compared to WT, relative transcript abundance of *pelA* is increased about 18-fold in the *Phy-pel* strain (Fig. S9). Accordingly, the *Phy-pel* strain exhibits a hyper-wrinkled biofilm phenotype that is distinct from WT in the colony biofilm assay (Fig. 6A through C) and increased attachment in the SSA biofilm assay (Fig. 6D). Consistent with the result that RhlR activates the expression of *pel* (Fig. 2), overexpression of *pel* is epistatic to Δ*rhlR* as the Δ*rhlR Phy-pel* strain shows reduced biofilm spreading and increased attachment to polystyrene surface compared to the parent Δ*rhlR* mutant (Fig. 6). Although the abundance of *pelA* transcript is similar between Δ*cbrA* and *Phy-pel* mutants, the *Phy-pel* strain produced a hyper-rugose biofilm morphology that is markedly distinct from the Δ*cbrA* mutant biofilm and showed lower crystal violet staining (Fig. 6; Fig. S9). Furthermore, the absence of *cbrA* is epistatic to overexpression of *pel* as the Δ*cbrA Phy-pel* double mutant formed biofilms identical to the Δ*cbrA* mutant (Fig. 6). Notably, the Δ*crc Phy-pel* double mutant mirrors the Δ*crc* mutant biofilm phenotypes in the colony biofilm and solid-surface attachment assays (Fig. 6). Thus, we conclude that the absence of *crc* is epistatic to transcriptional activation of the *pelABCDEFG* operon.

Because Crc functions as a post-transcriptional regulator in reverse diauxie, we hypothesized that Crc mediates an increase in *pel* expression via a post-transcriptional mechanism. One well-known post-transcriptional mode of control of Pel biosynthesis is the second messenger cyclic di-guanosine monophosphate (c-di-GMP) such that high c-di-GMP levels correlate with biofilm formation (reviewed in reference 40). Furthermore, Hfq, and thereby carbon catabolite repression, has been linked to c-di-GMP concentration in cells (reviewed in reference 41). This prompted us to determine intracellular c-di-GMP concentration of the mutants in this study. Figure 6E shows that while c-di-GMP concentration is lowered in the Δ*crc* mutant compared to WT, none of the Δ*rhlR,* Δ*cbrA,* Δ*rhlR*Δ*cbrA,* and Δ*rhlR*Δ*cbrA*Δ*crc* mutants displayed any discernable changes from WT. We conclude that c-di-GMP levels do not contribute to the difference in biofilm development of the Δ*rhlR,* Δ*cbrA,* and Δ*rhlR*Δ*cbrA* mutants.

**Fig 7:**
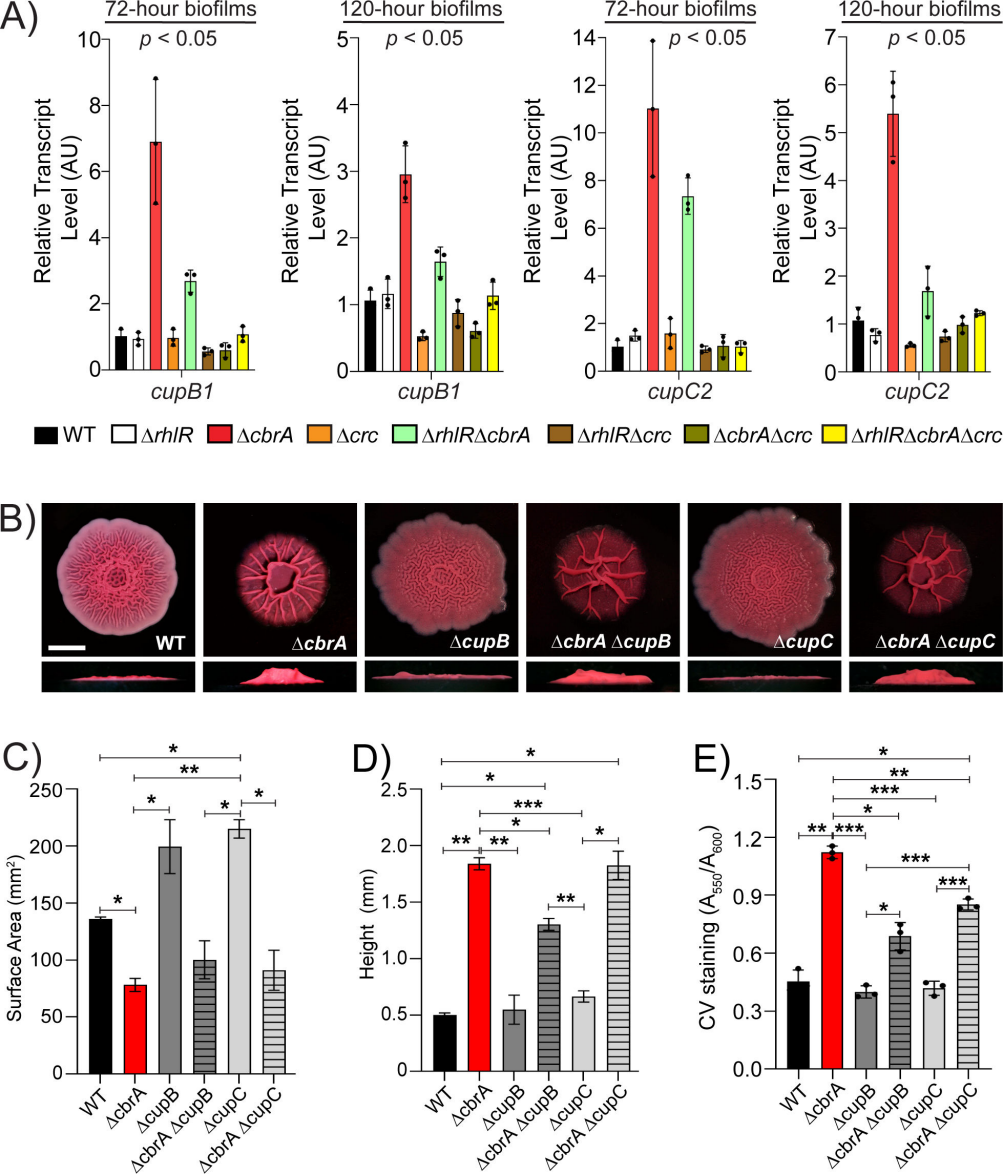
The *cupB* and *cupC* fimbrial operons are overexpressed in the absence of CbrA. (**A**) Relative expression of *cupB1* and *cupC2* genes normalized to 16S RNA, *ostA,* and *rpsO* transcript levels in WT PA14 and indicated mutants after 72 and 120 h of colony biofilm growth. AU denotes arbitrary units. Error bars represent the standard deviation of three biological replicates. Statistical significance was determined using Welch’s ANOVA in GraphPad Prism software. For *cupB1* expression at 72 and 120 h, the following comparisons were statistically significant (*P* < 0.05): Δ*cbrA* vs all other strains. For *cupC2* expression at 72 h, the following comparisons were statistically significant (*P* < 0.05): Δ*cbrA* vs all other strains except Δ*rhlR*Δ*cbrA*, and Δ*rhlR*Δ*cbrA* vs all other strains except Δ*cbrA*. For *cupC2* expression at 120 h, the following comparisons were statistically significant (*P* < 0.05): Δ*cbrA* vs all other strains except Δ*rhlR*Δ*cbrA* mutant. (**B**) Colony biofilm phenotypes of WT PA14 and the designated mutants on Congo red agar medium after 120 h of growth. Scale bar, 5 mm. (**C**) Colony biofilm surface area quantitation for the indicated strains after 120 h of growth. Error bars represent the standard deviation of three independent experiments. (**D**) Colony biofilm height quantitation for the indicated strains after 120 h of growth. Error bars represent the standard deviation of three independent experiments. (**E**) Biofilm crystal violet staining assays for WT and indicated mutant strains. Error bars represent the standard deviation of three biological replicates. (**C–E**) Only pairwise comparisons that had *P* value < 0.05 are denoted. Statistical significance was determined using Welch’s ANOVA with Dunnett’s T3 multiple comparisons test in GraphPad Prism software. ***P < 0.001, **P < 0.01, *P < 0.05.

In addition to Pel and CdrA, the biofilm matrix involves a large arsenal of cell surface-associated structures, including type IV pili, Fap fibrils, and the cup fimbriae (42–47). *P. aeruginosa* can produce five types of cup fimbriae—CupA, CupB, CupC, CupD, and CupE, products of *cupA*, *cupB*, *cupC*, *cupD*, and *cupE* gene clusters each of which encodes an usher, a chaperone, and at least one fimbrial subunit. Our RNA-seq data revealed that *cupB* and *cupC* biosynthetic operons are upregulated in the Δ*cbrA* and Δ*rhlR*Δ*cbrA* mutants (Fig. 4C; Table S3). To assess the coordinated regulation of the Cup components of the biofilm matrix by RhlR, CbrA, and Crc over time, we performed quantitative RT-PCR for *cupA1*, *cupB1*, *cupC2*, *cupD4*, and *cupE1* from colony biofilm samples after 72 and 120 h of growth, respectively, in the WT and the Δ*rhlR,* Δ*cbrA,* Δ*crc,* Δ*rhlR*Δ*cbrA,* Δ*rhlR*Δ*crc,* Δ*cbrA*Δ*crc,* and Δ*rhlR*Δ*cbrA*Δ*crc* mutants (Fig. 7A; Fig. S9). Our data show that the relative mRNA levels of the *cupB1* and *cupC2* genes increased greater than fivefold at 72 h and more than threefold at 120 h in the Δ*cbrA* single and Δ*rhlR*Δ*cbrA* double mutants, but these increases in transcript levels were abolished in the Δ*cbrA*Δ*crc* double and Δ*rhlR*Δ*cbrA*Δ*crc* triple mutants (Fig. 7A; Fig. S9). This suggests that, in addition to Pel, Crc promotes the expression of CupB and CupC matrix components.

To determine the role of CupB and CupC fimbriae in biofilms of the Δ*cbrA* mutant, we generated deletion mutants where we removed entire *cupB* (*cupB1-B5*) and *cupC* (*cupC1-C3*) operons in WT and Δ*cbrA* backgrounds. The Δ*cbrA*Δ*cupB* mutant exhibited significant, albeit minor, decrease in biofilm height compared to the Δ*cbrA* mutant while for the Δ*cbrA*Δ*cupC* mutant, surface area coverage and height parameters show values similar to the Δ*cbrA* mutant (Fig. 7B through D). Notably, the absence of CupB and CupC reduced SSA biofilms in the Δ*cbrA* mutant (Fig. 7E). We conclude that Crc promotes the expression of CupB and CupC fimbriae that, in turn, contribute to the formation of hyper-rugose biofilms.

**Fig 8:**
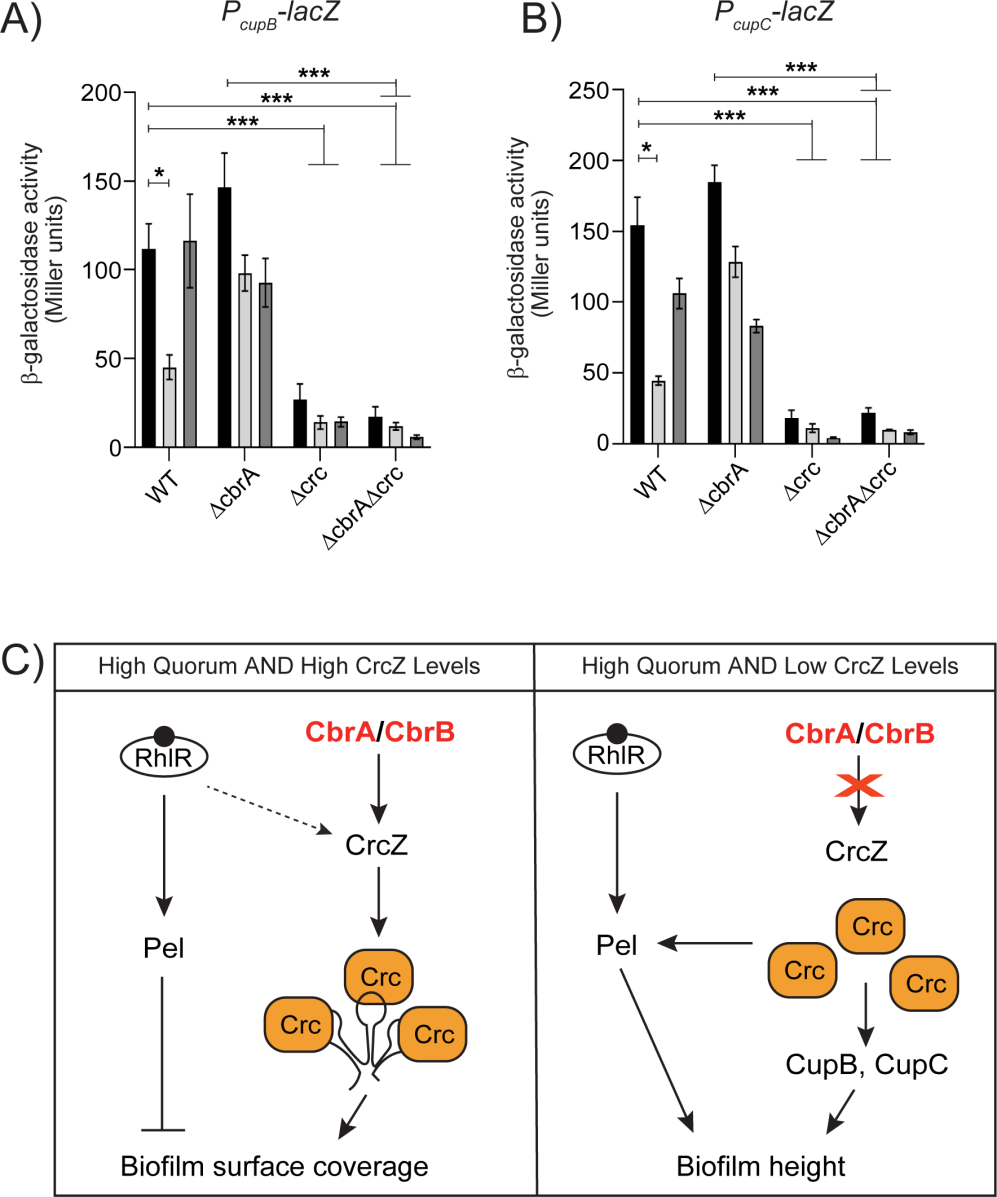
Crc promotes the transcription of *cupB* and *cupC* fimbrial operons. (**A and B**) β-Galactosidase assays of *PcupB-lacZ* transcriptional (**A**) or *PcupC-lacZ* transcriptional (**B**) fusions for background genotypes indicated on the *x*-axis and grown in tryptone broth with no addition (black), 50 mM pyruvate (light gray), or 50 mM glucose (dark gray). Error bars represent SEM of three biological replicates. Only pairwise comparisons that had *P* value < 0.05 are denoted. Statistical significance was determined using Welch’s ANOVA with Dunnett’s T3 multiple comparisons test in GraphPad Prism software. ***P < 0.001, *P < 0.05. (**C**) Model for Crc-dependent activation of biofilm matrix components. At high population density, RhlR-mediated quorum sensing activates the expression of Pel exopolysaccharide while Crc, depending on the abundance of CrcZ small RNA, promotes the expression of Pel, CupB, and CupC fimbriae components of the biofilm matrix. The arrow indicates activation and T-bar indicates inhibition.

The levels of CrcZ small RNA have been reported to vary according to the carbon source in the growth medium which, in turn, affects catabolite repression (14). Therefore, we probed the expression of biofilm matrix genes in response to carbon sources by generating transcriptional reporter fusions to the *cupB* and *cupC* promoters (P*cupB-lacZ* and P*cupC-lacZ*). We incorporated the reporter fusion into an intergenic region on the chromosomes of WT *P. aeruginosa* and the Δ*cbrA,* Δ*crc* and Δ*cbrA*Δ*crc* mutants. The P*cupB-lacZ* and P*cupC-lacZ* reporters exhibited approximately four- and eightfold lower expression in the Δ*crc* and Δ*cbrA*Δ*crc* mutants than the WT in tryptone broth (Fig. 8A and B). These results show that Crc is absolutely required for the expression of *cupB* and *cupC* operons in both WT and Δ*cbrA* backgrounds. In *P. aeruginosa*, “reverse” carbon catabolite repression is known to be triggered by organic acids such as pyruvate (48). Furthermore, pyruvate activates the expression of *crcZ* in both *P. aeruginosa* and *P. putida* (49). Therefore, we tested pyruvate as preferred carbon source and glucose as non-preferred sugar. The expression of the reporters in the WT was reproducibly and significantly lowered in the presence of pyruvate but not glucose (Fig. 8A and B). Taken together, our data lead to a regulatory model in which environmental cues that reduce the levels of CrcZ small RNA allow for Crc-dependent activation of biofilm matrix gene expression in *P. aeruginosa* (Fig. 8C).

**Fig 9:**
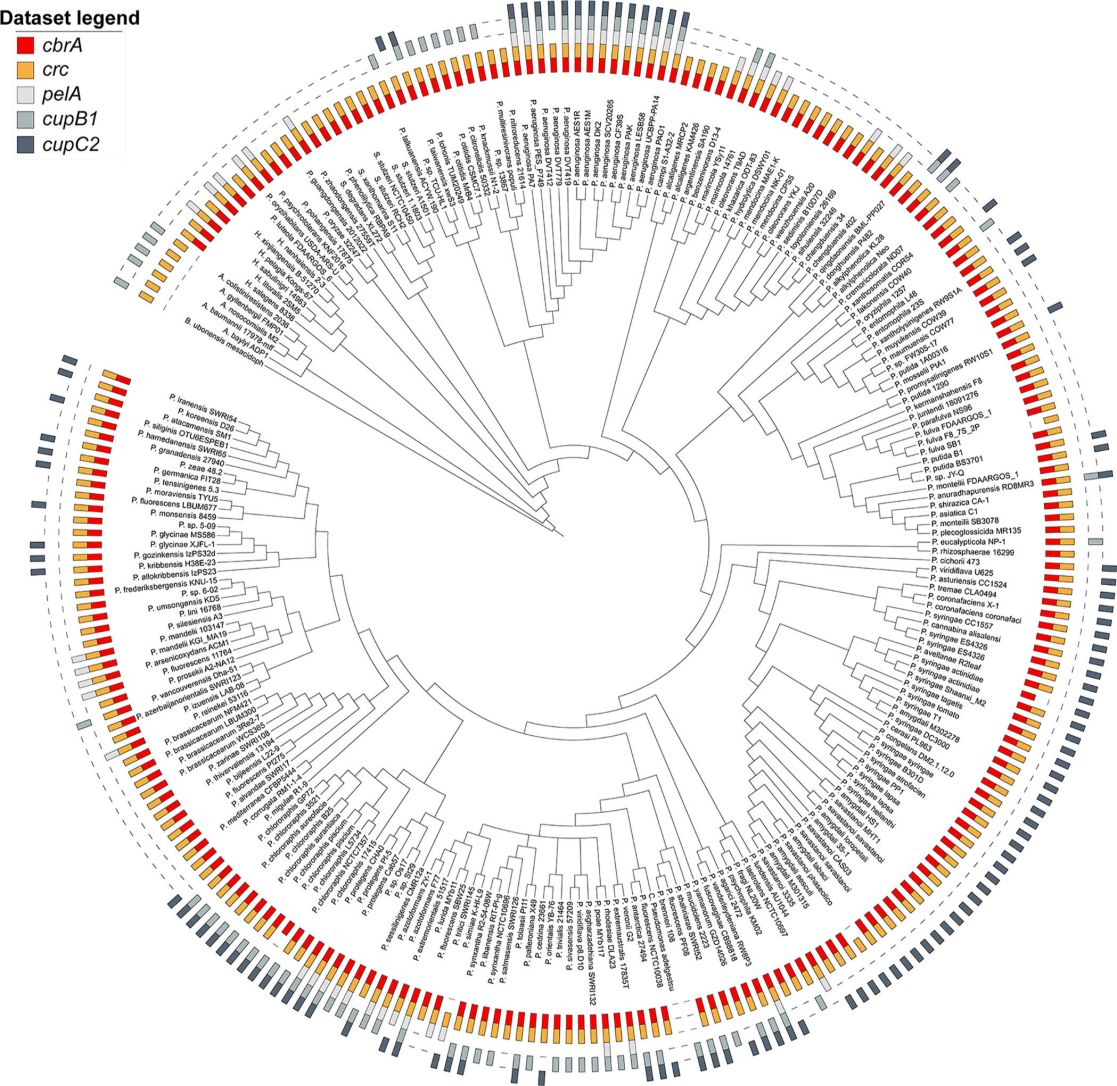
The CbrA and Crc proteins and their regulatory targets Pel, CupB, and CupC are conserved in diverse pseudomonads. Co-occurrences of CbrA and Crc are depicted in red and orange, respectively, while that of their regulatory target genes obtained from this study—*pelA*, *cupB1*, and *cupC2—*are depicted in shades of gray as indicated in the data set legend.

Bacteria of the genus Pseudomonas are metabolically versatile, can be isolated from a wide range of niches, and preferentially catabolize organic acids as they lack a functional glycolytic pathway (reviewed in reference 50). This “reverse” carbon catabolite repression is thought to provide adaptive advantage in different niche colonization. As such, the CbrA/CbrB/CrcZ/Crc signaling cascade is conserved in most pseudomonads, and components of this pathway have been found to be exchangeable between *P. aeruginosa* and *P. putida* (49). We explored whether Crc in other species of *Pseudomonas* might also be involved in controlling biofilm formation via phylogenetic analysis of the co-occurrence of *cbrA, crc, pelA, cupB1,* and *cupC2* in the genomes of diverse pseudomonads (Fig. 9). Consistent with previous reports, majority of the pseudomonads encode for CbrA and Crc orthologs. PelA and CupB1 are found to co-occur in a subset of the genomes that include several CF isolates of *P. aeruginosa,* while CupC2 is present in ~45% of the genomes analyzed here. For example, pseudomonads that colonize the rhizosphere of plants such as *P. stutzeri*, *P. chlororaphis*, *P. syringae,* and *P. protegens* encode for genes required to assemble CupC fimbriae. These bacteria are exposed to glucose as well as organic acids like succinate and pyruvate that are commonly found in root exudates and likely derive ecological advantage by integrating nutritional cues to control adhesion to root surfaces. Thus, it will be interesting to know whether Crc has a conserved function in the regulation of CupC fimbriae which might also contribute to biofilm development in the environmental pseudomonads.

## DISCUSSION

Combinatorial gene regulation by multiple two-component systems is highly utilized in bacteria in responding to environmental cues to allow distinct gene expression patterns. *P. aeruginosa* is a versatile human pathogen that causes both acute and chronic infections such as in the lungs of cystic fibrosis (CF) patients and in patients suffering from burn and diabetic wounds that are often associated with worse disease outcomes (51–54). In this study, we sought to define the interaction of Rhl quorum sensing with another global regulatory system, i.e., CbrA/CbrB pathway. As the sensory signal that activates CbrA kinase is unknown (17), we studied biofilm development and biofilm-associated transcriptomic changes in the absence of RhlR, CbrA, and both. Our RNA-seq analysis sheds light on the transcriptomic landscape of WT *P. aeruginosa* biofilms and provides a map of the regulatory network orchestrated by RhlR, CbrA, and Crc. Furthermore, our data highlight the complexity of sensory regulation mediated by these two signaling pathways in *P. aeruginosa* biofilms and reveals a previously unexplored facet of Crc as it promotes the expression of specific biofilm matrix components—Pel exopolysaccharide and CupB/CupC fimbriae.

*P. aeruginosa*, one of the major causes of morbidity and mortality of CF patients, colonize the lung mucus which is a complex substrate that provides carbon, nitrogen, and other energy sources during infection (55). Recent studies have highlighted the importance of carbon catabolite repression in virulence-associated processes such as quorum sensing, phenazine production, and antibiotic resistance (56, 57). Although glucose is a less preferred carbon source for *P. aeruginosa*, it is present at high concentration in the mucus of both CF and non-CF patients, with CF mucus typically having higher glucose content than those of other pulmonary diseases (55, 56, 58). Our work suggests that glucose (or another less preferred carbon source) could function as an inducer of biofilm formation via the CbrA/CbrB/CrcZ/Crc pathway. Additional work is required to define the molecular mechanism by which Crc activates the expression of *pel, cupB,* or *cupC* genes. We speculate that one possible mechanism for increased *pel* expression might be at the post-transcriptional level as the absence of Crc decreases biofilm formation even after exchanging the *PpelA* promoter with an artificial constitutive promoter. Nonetheless, given that both carbon catabolite repression and Pel biosynthetic genes are widespread in bacteria (59), we expect future studies defining the molecular details underlying the link between reverse diauxie and *pel* expression would be informative.

The material properties of the matrix contribute to biofilm growth and architecture. For instance, in *Vibrio cholerae* and *Bacillus subtilis*, matrix production allows biofilm-dwelling cells to establish an osmotic pressure difference between the biofilm interior and the external environment that promotes biofilm expansion (60, 61). Specifically, *V. cholerae* colony biofilms were found to expand more in the absence of the matrix protein RbmA (60). Furthermore, biofilm matrix components contribute to processes such as signaling, migration, and genetic exchange over the course of biofilm development (reviewed in reference 62). *B. subtilis* biofilm matrix protein BslA and exopolysaccharide EPS are essential for sliding motility that allows colony expansion via surface spreading (63). Our work suggests that in PA14, lower Pel polysaccharide levels allow greater surface area coverage as observed in the Δ*rhlR* mutant. Conversely, increased Pel polysaccharide levels limits biofilm expansion as is the case with Δ*cbrA* and Δ*rhlR* Δ*cbrA* mutants. Increased matrix combined with reduced levels of CrcZ in the Δ*rhlR* Δ*cbrA* mutant leads to spontaneous mutations in Crc that, in turn, decreases Pel levels to allow biofilm growth and expansion.

The *cup* genes in *P. aeruginosa* are poorly expressed in standard laboratory conditions making it difficult to study their regulation, and therefore, few regulators such as the H-NS-like protein MvaT that represses *cupA* gene expression and the two-component systems named Roc1 and Roc2 that induce the expression of *cupB* and *cupC* genes have been described (46, 64). Nonetheless, the Cup fimbrial structures have been reported to facilitate bacterial attachment to host tissue and promote biofilm formation and pathogenesis (43, 65). We uncover the significant contribution of CupB and CupC fimbriae to the biofilms made by the Δ*cbrA* mutant (Fig. 7A) suggesting the CbrA/CbrB system downregulates Cup fimbriae expression under laboratory settings. Furthermore, our finding that organic acids such as pyruvate can repress the expression of *cupB* and *cupC* fimbrial genes hints at a change in adhesion depending on nutritional cues that might contribute to biofilms within hosts.

Transcriptome analysis may reveal both direct and indirect targets of Crc. Crc/Hfq binding to transcripts is thought to involve at least one CA motif (AAnAAnAA) in the neighborhood of the translational start site in target mRNAs (16, 18). Accordingly, we scanned the 5′ untranslated region of Crc-regulated genes in two RNA-seq data sets—Δ*crc* vs WT and Δ*rhlR*Δ*cbrA* vs Δ*rhlR*Δ*cbrA*Δ*crc* mutants. We found 32 and 73 Crc-regulated transcripts having a putative CA motif, respectively, but none of these transcripts belong to biofilm-matrix biosynthesis genes (Table S4). One caveat is that the Crc/Hfq recognition motif for biofilm-matrix biosynthesis genes might be distinct from the aforementioned CA motif. Another possibility is that Crc indirectly activates the expression of biofilm matrix components. This study provides the basis for future biochemical characterization of Crc to define how it functions as a positive regulator of biofilm development. Taken together, by characterizing the convergence of global signaling pathways on biofilm development, we have expanded our understanding of the molecular interplay between key regulators—CbrA and RhlR and identified Crc as a master regulator of biofilm matrix gene expression in response to environmental stimuli.

## MATERIALS AND METHODS

### Strains and growth conditions

*P. aeruginosa* UCBPP-PA14 strain was grown in lysogeny broth (LB) (10 g tryptone, 5 g yeast extract, 5 g NaCl per L), in 1% tryptone broth (TB) (10 g tryptone per L), and on LB plates fortified with 1.5% Bacto agar at 37˚C. When appropriate, antimicrobials were included at the following concentrations: 400 µg/mL carbenicillin, 50 µg/mL gentamycin, 100 µg/mL irgasan.

### Strain construction

Strains and plasmids were constructed as described previously (25). To construct marker-less in-frame chromosomal deletions in *P. aeruginosa*, DNA fragments flanking the gene of interest were amplified, assembled by the Gibson method, and cloned into pEXG2 (66). The resulting plasmids were used to transform *Escherichia coli* SM10λ*pir* and, subsequently, mobilized into *P. aeruginosa* PA14 via biparental mating. Exconjugants were selected on LB containing gentamicin and irgasan, followed by recovery of deletion mutants on LB medium containing 5% sucrose. Candidate mutants were confirmed by PCR. The *cbrA* complementation plasmid was constructed by inserting DNA containing the promoter of *dksA* and the entire *cbrA* open-reading frame using HindIII and XbaI, followed by cloning into similarly digested pUCP18. The *crc* complementation plasmid was constructed by inserting DNA containing the promoter and entire *crc* open-reading frame using HindIII and XbaI, followed by cloning into similarly digested pUCP18. The *crcZ* overexpression plasmid was constructed by inserting DNA containing the entire *crcZ* gene using HindIII and XbaI, followed by cloning into similarly digested pUCP18.

To construct the P*cupB-lacZ* and P*cupC-lacZ* transcriptional reporter fusions, 500 bp of DNA upstream of the *cupB1 and cupC1* genes and the DNA encoding the *lacZ* open-reading frame were amplified using *P. aeruginosa* PA14 genomic DNA and the plasmid pIT2 as templates, respectively. Next, two DNA fragments of ~730 bp, one corresponding to the intergenic region ~700 bp downstream of the *P. aeruginosa PA14_20500* gene and the other corresponding to ~1,000 bp upstream of the *P. aeruginosa PA14_20510* gene, were amplified from *P. aeruginosa* PA14 genomic DNA. The four DNA fragments were assembled by the Gibson method and cloned into pEXG2. The resulting plasmid was used to transform *E. coli* SM10λ*pir* and subsequently mobilized into *P. aeruginosa* PA14 WT and the Δ*rhlR* and Δ*rhlI* mutants via biparental mating as described above.

### Colony biofilm assay

One microliter of overnight *P. aeruginosa* cultures grown at 37°C in 1% tryptone broth was spotted onto 60 × 15 mm petri plates containing 10 mL 1% tryptone medium fortified with 40 mg per L Congo red and 20 mg per L Coomassie brilliant blue dyes and solidified with 1% agar. Colonies were grown at 25°C under dark conditions, and images were acquired using a Zeiss AxioZoom v16 microscope.

### Crystal violet staining of SSA biofilms

*P. aeruginosa* was cultured overnight in 200 µL LB in a BioTek Synergy Neo2 microplate reader at 37°C under shaking conditions. The OD_600_ of the culture was taken by the plate reader to calculate the amount of culture needed to inoculate 200 µL 1% tryptone broth so that the final OD_600_ is 0.005. Biofilms were developed for 72 h in stationary 96-well polystyrene plate at 25°C in dark conditions. After 72 h, OD_600_ measurement was taken, and the supernatant was discarded. The cultures were washed vigorously with tap water and were left to dry for an hour. The biofilms were then stained 250 µL 0.1% crystal violet solution for a half an hour. The excess solution was poured out, and the samples were washed thrice with tap water and let to dry overnight. For elution, 250 µL of 33% glacial acetic acid solution was used in each well, and samples were left to rest for an hour. The crystal violet stain was quantified at OD_550_ using the microplate reader.

### RNA-seq

*P. aeruginosa* colony biofilms were harvested with a sterile plastic inoculation loop. Half of each biofilm was picked up and transferred into 600 µL of Tri-reagent (Zymo Research) and disrupted by pipetting with a 1 mL tip. Total RNA was extracted with the Zymo Direct-zol RNA kit following the manufacturer’s instructions. Samples were subjected to DNase treatment using Ambion DNase I (RNase-free) kit, followed by rRNA depletion and library preparation with Illumina Stranded Total RNA Prep with Ribo-Zero kit. Libraries were sequenced on Illumina’s NextSeq 2000, with 51 bp paired-end reads and 10 bp long index reads (51-10-10-51).

### RNA-seq analysis

RNA-seq reads were processed using the nf-core RNAseq pipeline version 3.4. Briefly, low-quality bases and contaminant adapters were trimmed using Cutadapt version 3.4 and Trimgalore version 0.6.7. RNA-seq reads passing quality filters were mapped against the reference genome of *P. aeruginosa* UCBPP_PA14 strain (GenBank assembly accession number CP000438.1) using HISAT2 version 2.2.0 (with parameters --rna-strandness RF, --no-mixed, --no-discordant, --no-spliced-alignment [67]). Next, read mappings for each annotated gene were counted using the featureCounts program within the Subread package version 2.0.1 (with parameters -B -C and -s 2 [68]). Analysis of differentially expressed genes (DEGs) was performed using the DESeq2 package 1.28.0 (69). Genes were considered significantly differentially expressed when the *P* value (*P*adj), adjusted using the Benjamini-Hochberg procedure, was <0.05. RNA-seq analysis pipeline is available on GitHub at https://github.com/trestle-biosciences/rnaseq-cbra-rhlr-crc-mukherjee. Assessment of functional classification enrichment was performed by assigning DEGs to at least one of 27 manually defined and curated PseuodCAP functional classifications (38). The percentage of genes in each category that exhibited downregulation or upregulation was then calculated.

### qRT-PCR

*P. aeruginosa* colony biofilms were harvested with a sterile plastic inoculation loop. Half of each biofilm was picked up and transferred into 600 µL of Tri-reagent (Zymo Research) and disrupted by pipetting with a 1 mL tip. Total RNA was extracted with the Zymo Direct-zol RNA kit following the manufacturer’s instructions and quantified with a BioTek Synergy Neo2 microplate reader. 0.5 µg of total RNA was used for reverse-transcription using the TaKaRa PrimeScript RT Reagent Kit with gDNA Eraser. The resulting cDNA was used for real-time PCR using the Applied biosystems PowerTrack SYBR Green Master Mix on a Bio-Rad C1000 Touch Thermal Cycler. The results were exported into RDML v1.1 format and analyzed with web-based LinRegPCR (https://www.gear-genomics.com/rdml-tools/). Relative fold change was calculated with the qBase method (70) using *16 s, clpX*, *ostA,* and *rpsO* as reference genes.

### β-Galactosidase assay

Briefly, bacterial cultures were grown to OD_600_ ~1.0. Pellets of 1 mL cultures were collected and redissolved in 1 mL Z-buffer with the addition of 200 µg lysozyme for permeabilization. Samples were then incubated in 30°C for 15 min and diluted as needed in a total volume of 500 µL. To start the reaction, 100 µL of 4 mg/mL of ONPG solution was added to the samples and time was recorded. The samples were incubating in 30°C until sufficient color change was observed. The reaction was quenched by 250 µL 1M Na_2_CO_3_. Standard activity was calculated in Miller units.

### Intracellular c-di-GMP measurements

For intracellular c-di-GMP determination, cells were grown in tryptone broth and an aliquot was taken for OD measurement. One milliliter of cells was harvested, resuspended in 100 µL nucleotide extraction solution (40% acetonitrile, 40% methanol, 0.1% formic acid, and 19.9% water), and incubated at −20°C for 20 min. The samples were centrifuged at 15,000 rpm for 5 min, and the supernatant was transferred to a fresh tube. The samples were dried under vacuum, resuspended in 100 µL HPLC grade H_2_O, and analyzed by HPLC-MS/MS as described previously (71). Intracellular c-di-GMP levels were determined by fitting the peak intensity to a standard curve with the known concentrations of c-di-GMP and by normalizing the levels to the total number of cells.

### Phylogenetic tree construction

Genomes and associated proteomes GFF annotation files of strains from the *Pseudomonas* (238), *Acinetobacter* (5), and *Burkholderia* (1) genera were downloaded from the NCBI data sets database. To determine orthologous relationships between protein-coding genes, we used OrthoFinder version 2.5.4. The analysis was performed on an AWS EC2 instance type (c6a.48xlarge) with default settings. OrthoFinder computed hierarchical orthologous groups (HOGs) for each internal node in the species tree. To improve HOG prediction accuracy, an outgroup proteome (*Burkholderia*) was used to root the resulting species tree. HOGs are sets of proteins descended from a single gene in the ancestral species corresponding to the respective internal node. In this study, we focused on analyzing HOGs associated with the species tree node representing the last common ancestor of all A. *Pseudomonas*, *Acinetobacter*, and *Burkholderia*. Specifically, we examined HOGs containing cbrA, crc, and members of the *pel*, *cupB*, and *cupC* operons. For visualizing and annotating phylogenetic trees, custom Python scripts were employed to generate the data sets for annotation in the Interactive Tree of Life (iTol) tool (https://itol.embl.de/). Jupyter notebooks for downloading genomes from NCBI, processing OrthoFinder results, and creating figures are available on GitHub at https://github.com/JonWinkelman/dash_app_pseudomonas.

## Data Availability

All relevant data are provided within the article and the supplemental information. Whole genome sequencing and RNA-seq data sets generated in this paper have been deposited to SRA with accession number PRJNA1109357.

## SUPPLEMENTAL MATERIAL

Supplemental material msystems.00372-24-s0001.docx Figures S1 to S9; Tables S1 and S2; captions for Tables S3 and S4.

Table S3 msystems.00372-24-s0002.pdf RNA-seq data sets.

Table S4 msystems.00372-24-s0003.pdf Crc binding motif analysis.

ASM does not own the copyrights to Supplemental Material that may be linked to, or accessed through, an article. The authors have granted ASM a non-exclusive, world-wide license to publish the Supplemental Material files. Please contact the corresponding author directly for reuse.
