# Supplementary material for: Combinatorial control of Pseudomonas aeruginosa biofilm development by quorum-sensing and nutrient-sensing regulators
Source: mSystems. 2024 Aug 14;9(9):e00372-24. doi: 10.1128/msystems.00372-24 (PMC11406991; doi:10.1128/msystems.00372-24)
Supplement: Supplemental material — Figures S1 to S9; Tables S1 and S2; captions for Tables S3 and S4. [file msystems.00372-24-s0001.docx]

**Combinatorial control of *Pseudomonas aeruginosa* biofilm development by quorum-sensing and nutrient-sensing regulators**

Gong Chen^1^, Georgia Fanouraki^1^, Aathmaja Anandhi Rangarajan^2^, Bradford T. Winkelman^3^, Jared T. Winkelman^1^, Christopher M. Waters^2^, Sampriti Mukherjee^1^*

^1^The University of Chicago, Department of Molecular Genetics & Cell Biology, Chicago, Illinois, USA.

^2^Department of Microbiology and Molecular Genetics, Michigan State University, East Lansing, Michigan, USA.

^3^Trestle, LLC, Milwaukee, Wisconsin, USA.

*Correspondence to: sampriti@uchicago.edu

**Supplemental Figures S1-S9**

**Supplemental Tables S1-S4**


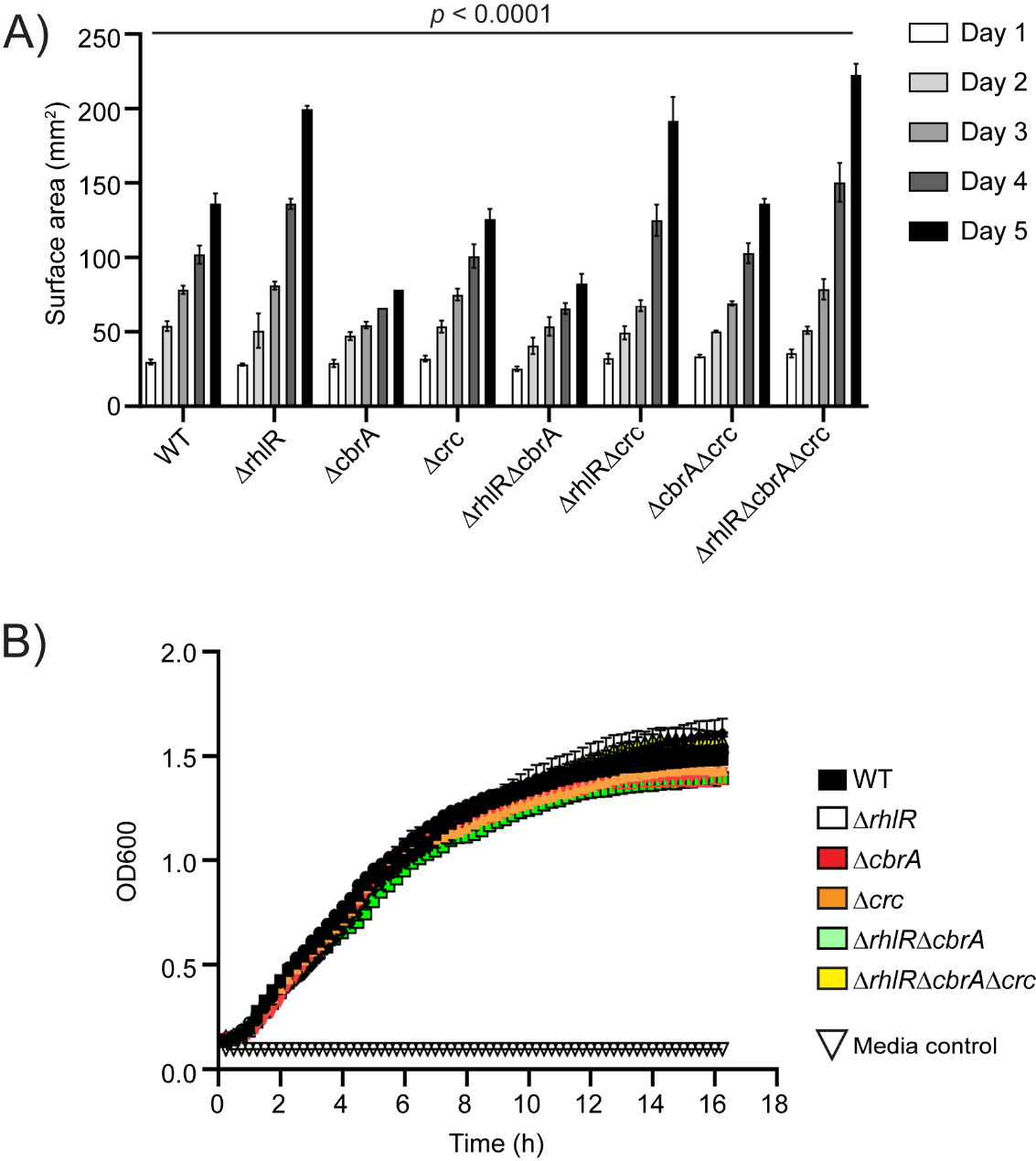


**Supplemental Fig. S1: Increase in biofilm surface area coverage of Δ*cbrA* and Δ*rhlR*Δ*cbrA* mutants is slower compared to WT and other studied mutants.** (A) Five-day time courses showing development of the colony biofilm surface area of the WT and indicated mutant strains. Error bars represent standard deviation of three biological replicates. Statistical significance was determined using Nested ANOVA in GraphPad Prism software. The following comparisons were statistically significant (p<0.0001): day 1 vs day 2, day 2 vs day 3, day 3 vs day 4, day 4 vs day 5 for all strains except Δ*cbrA* and Δ*rhlR*Δ*cbrA*. (B) Growth profiles of WT and indicated mutant strains in planktonic shaken cultures.


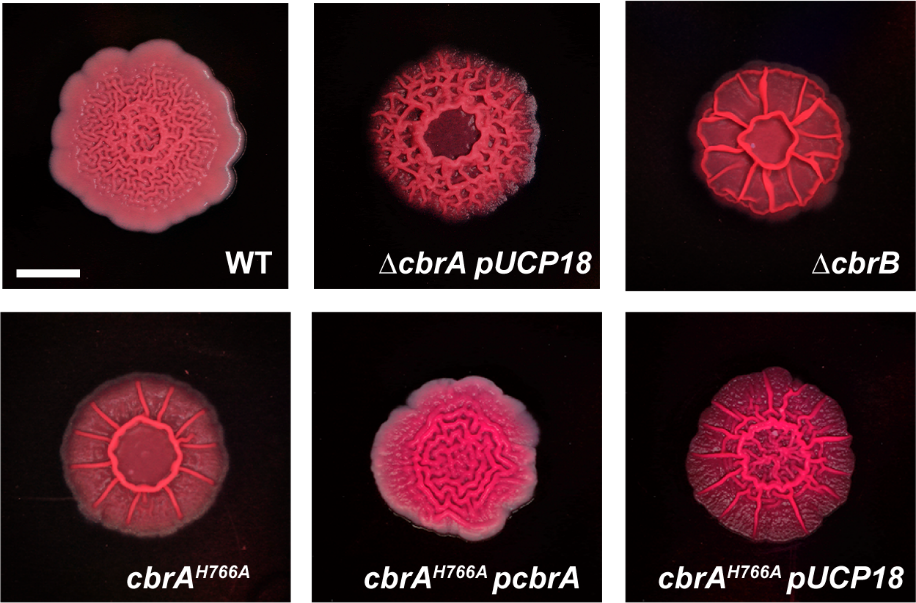


**Supplemental Fig. S2: CbrA mediated repression of biofilms requires its cognate response regulator CbrB and its kinase activity.** Colony biofilm phenotypes of the designated mutants on Congo red agar medium after 120 h of growth. Scale bar, 5 mm.


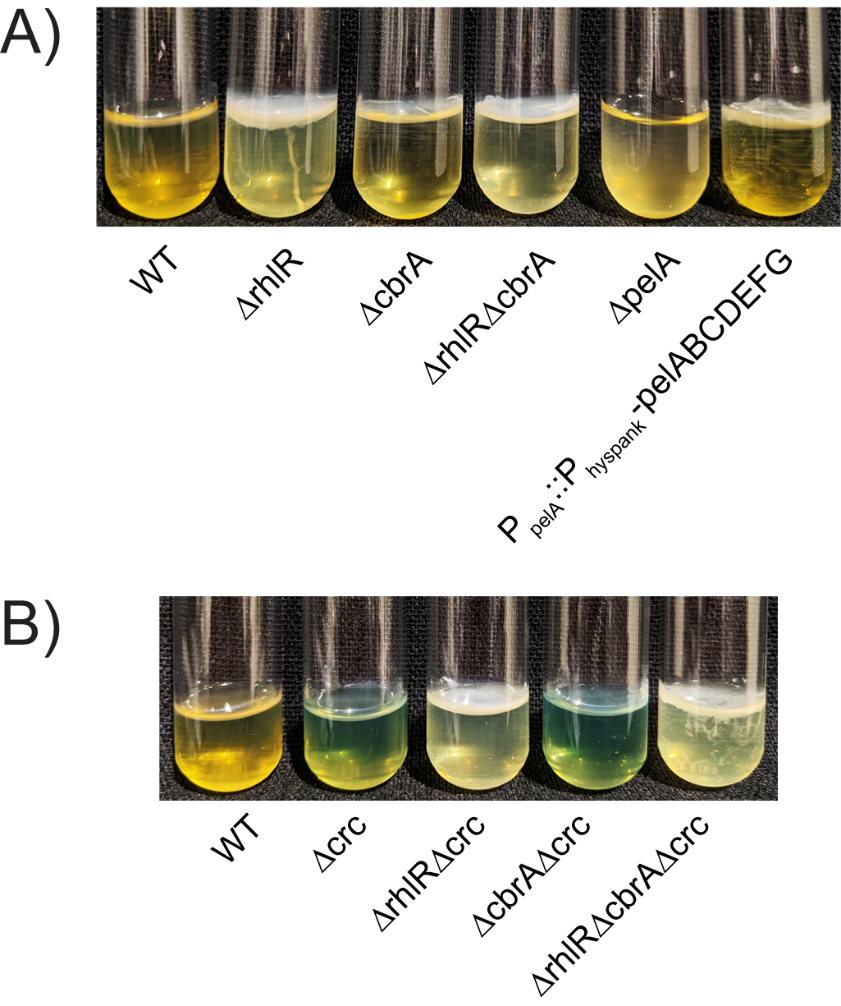


**Supplemental Fig. S3: CbrA and RhlR pathways repress pellicle development in addition to colony biofilms.** Pellicle biofilms of the indicated *P. aeruginosa* mutants imaged after 72 h of growth in standing cultures.


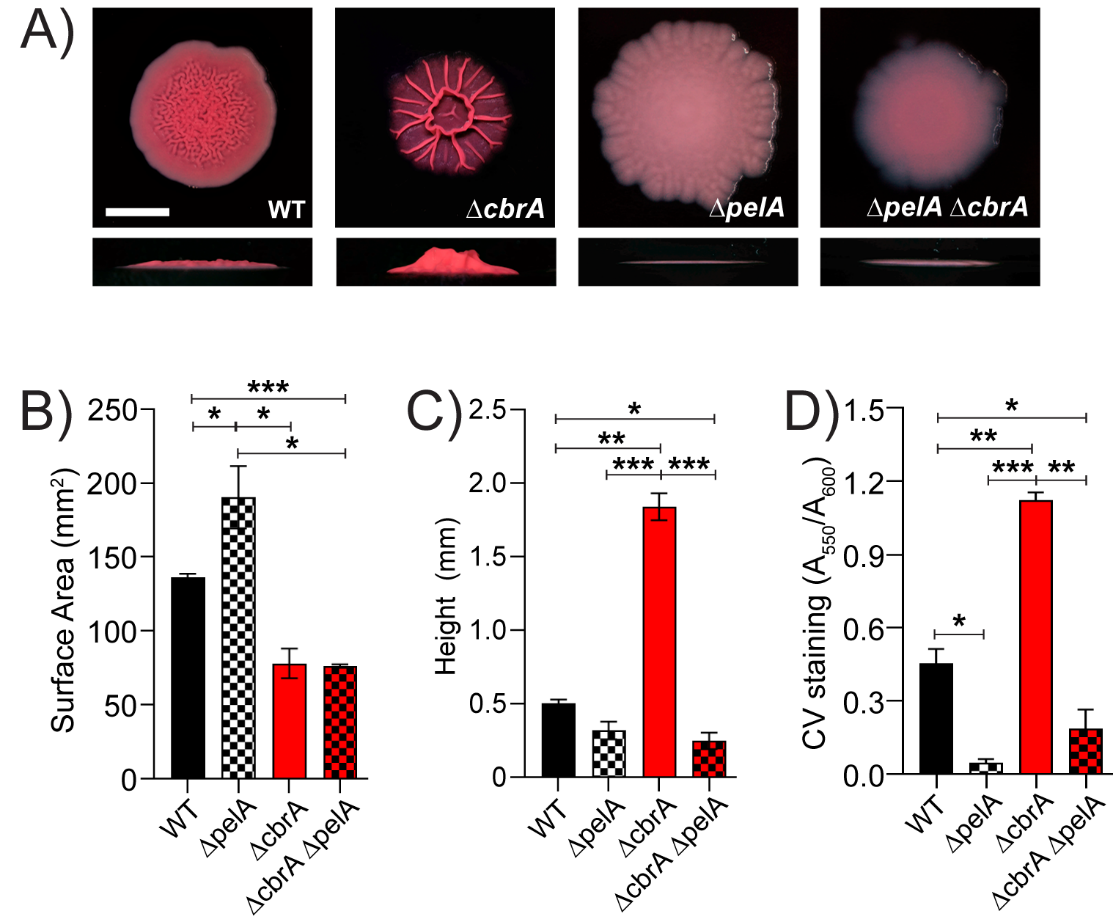


**Supplemental Fig. S4: Pel polysaccharide contributes to biofilm formation of the Δ*cbrA* mutant.** A) Colony biofilm phenotypes of WT PA14 and the designated mutants on Congo red agar medium after 120 h of growth. Scale bar, 5 mm. B) Colony biofilm surface area quantitation for the indicated strains after 120 h of growth. Error bars represent standard deviation of three independent experiments. C) Colony biofilm height quantitation for the indicated strains after 120 h of growth. Error bars represent standard deviation of three independent experiments. D) Biofilm crystal violet staining assays for WT and indicated mutant strains. Error bars represent standard deviation of three biological replicates. B-D) Only pairwise comparisons that had *p* value <0.05 are denoted. Statistical significance was determined using Welch’s ANOVA with Dunnett's T3 multiple comparisons test in GraphPad Prism software. *** P <0.001, ** P <0.01. * P <0.05.


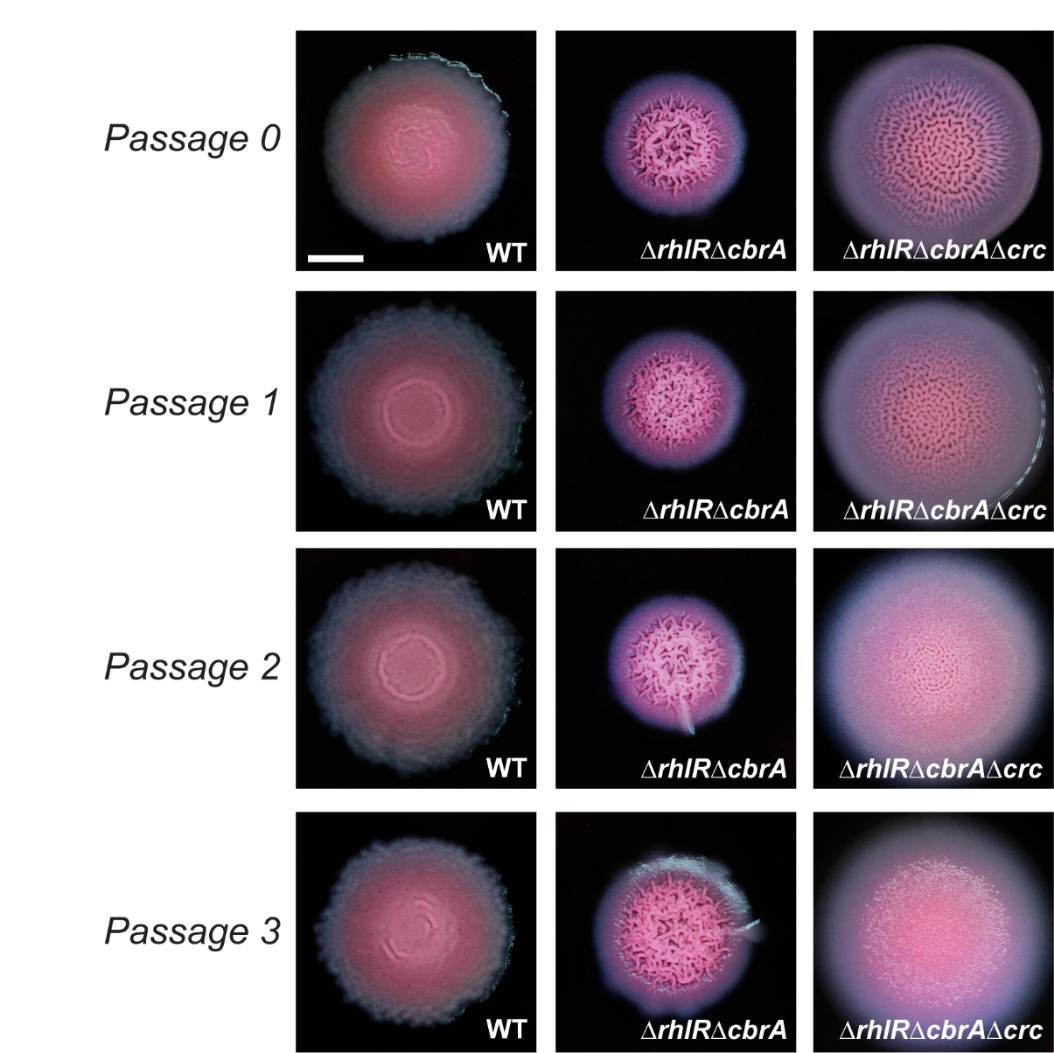


**Supplemental Fig. S5: Emergence of suppressor flares in the Δ*rhlR*Δ*cbrA*** **mutant is a biofilm-specific event.** The WT, Δ*rhlR*Δ*cbrA* and Δ*rhlR*Δ*cbrA*Δ*crc* strains were passaged overnight at 37C in Tryptone broth for three consecutive days and culture samples were inoculated on biofilm media after each overnight. Colony biofilm phenotypes of the designated mutants on Congo red agar medium after 72 h of growth. Scale bar, 2 mm.


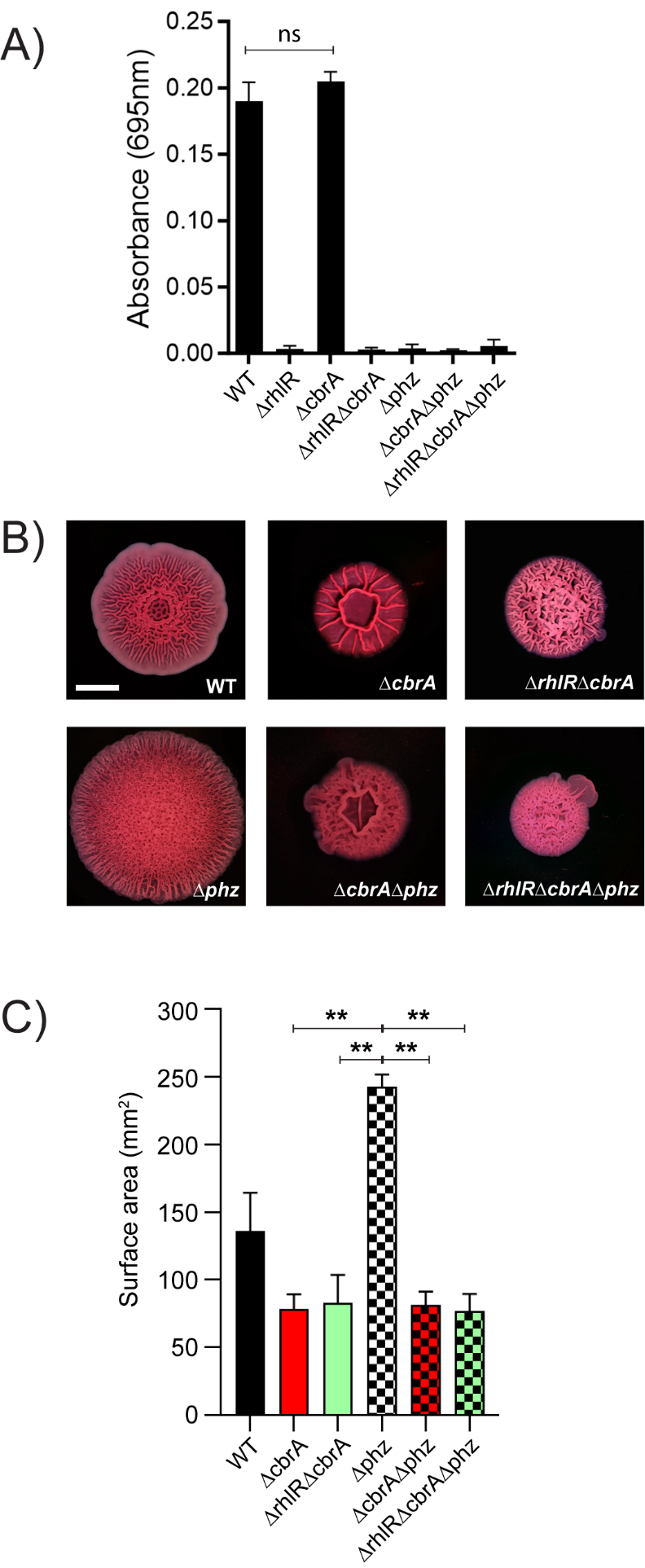


**Supplemental Fig. S6: Absence of phenazines is not epistatic to the combined absence of RhlR and CbrA.** A) Pyocyanin production phenotypes of the WT PA14 and indicated mutants imaged after overnight growth in LB. Statistical significance was determined using Welch’s ANOVA with Dunnett's T3 multiple comparison tests in GraphPad Prism software. All pairwise comparisons except WT with Δ*cbrA* had *p* value <0.0001. Not significant, ns. B) Colony biofilm phenotypes of the designated mutants on Congo red agar medium after 120 h of growth. Scale bar, 5 mm. C) Colony biofilm surface area quantitation for the indicated strains after 120 h of growth. Error bars represent standard deviation of three independent experiments. Only pairwise comparisons that had *p* value <0.05 are denoted. Statistical significance was determined using Welch’s ANOVA with Dunnett's T3 multiple comparisons test in GraphPad Prism software. ** P <0.01.


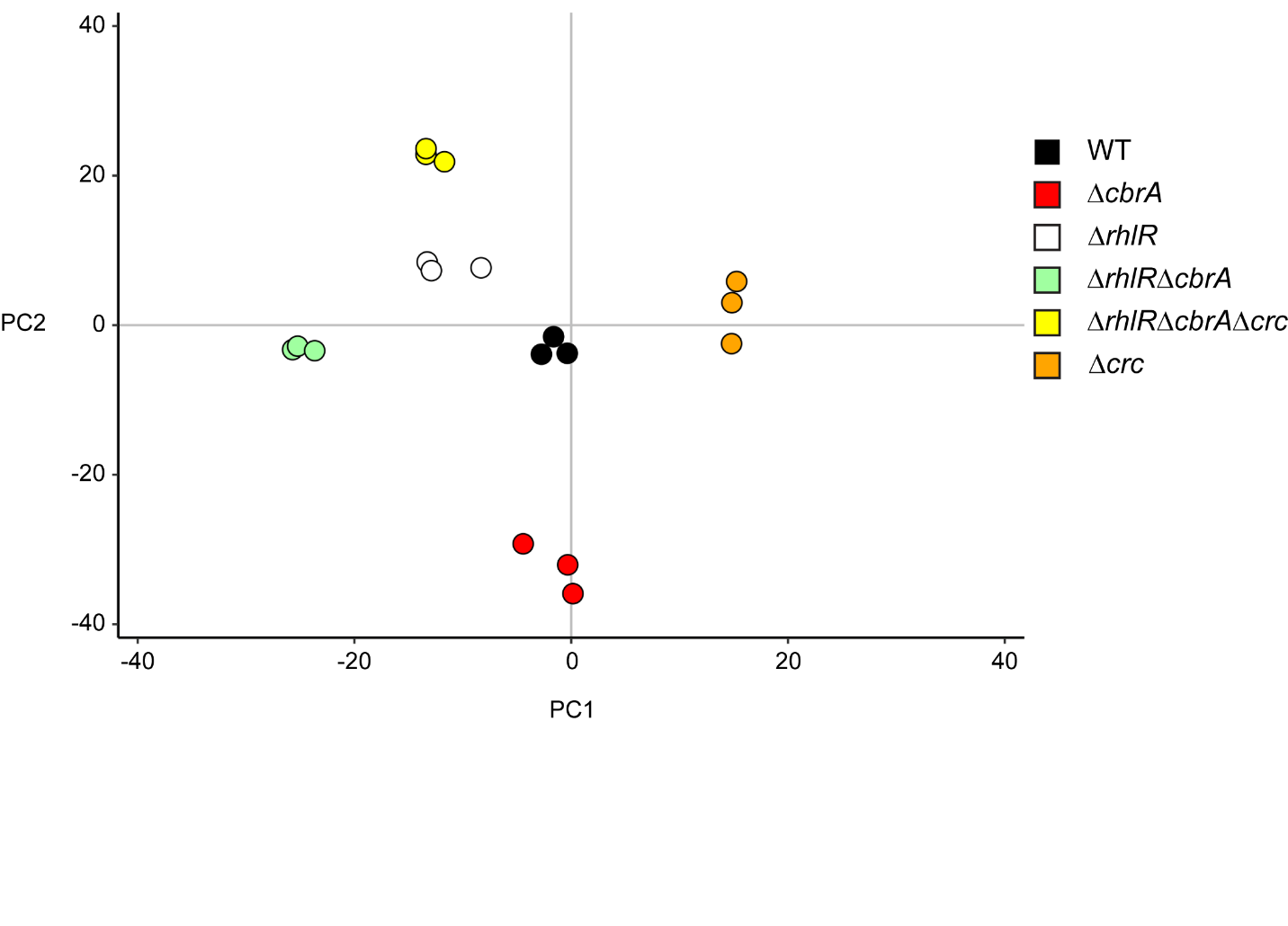


**Supplemental Fig. S7: Principal component analysis (PCA) for RNA-seq on biofilm samples from the indicated strains.**


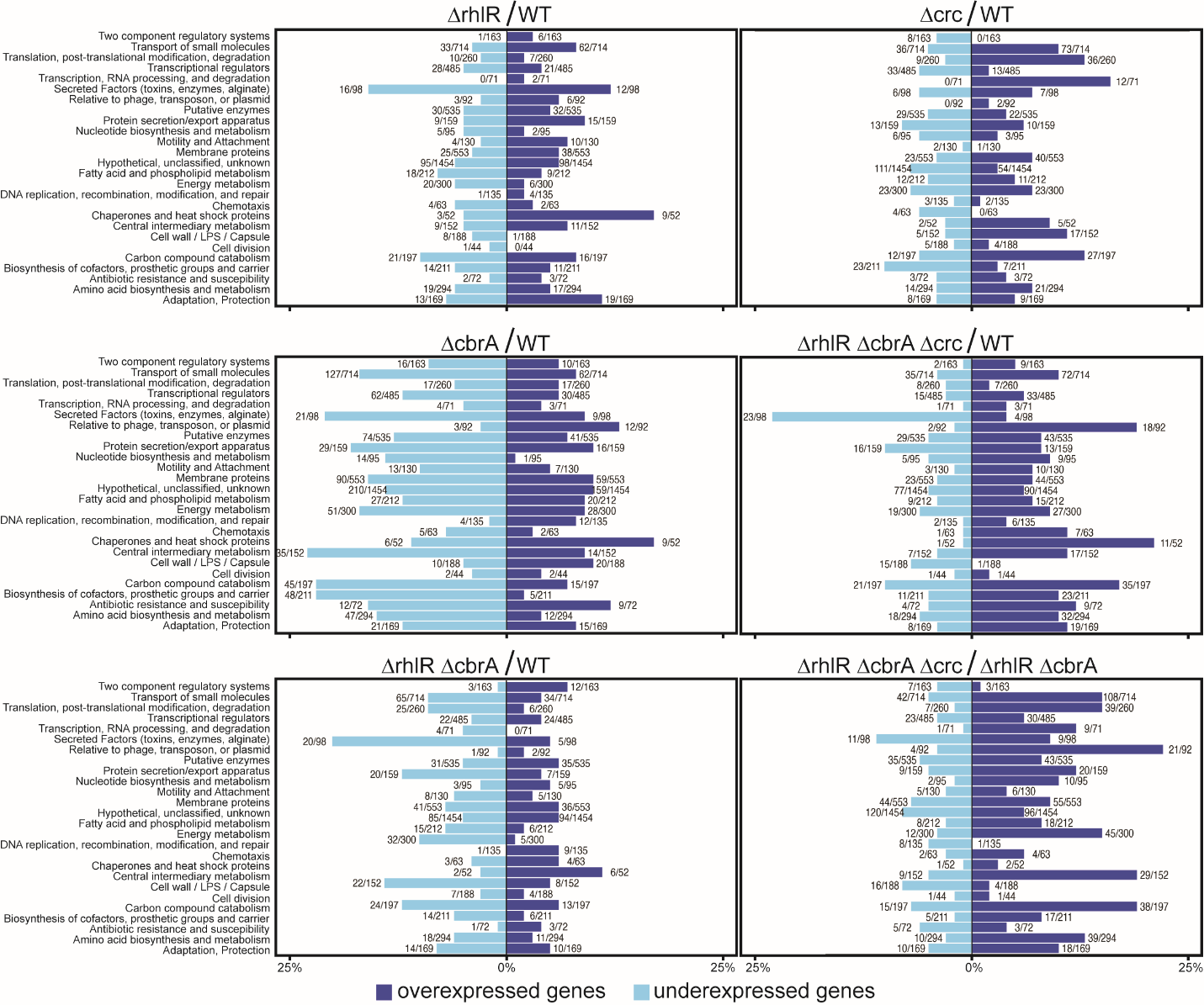


**Supplemental Fig. S8: Gene Ontology analysis of RNA-seq data.** Percentage of genes in each PseudoCAP category that are downregulated (light blue) or upregulated (dark blue) in Δ*rhlR,* Δ*cbrA,* Δ*crc*, Δ*rhlR*Δ*cbrA* or Δ*rhlR*Δ*cbrA*Δ*crc* compared to WT and Δ*rhlR*Δ*cbrA*Δ*crc* compared to Δ*rhlR*Δ*cbrA*. Numbers next to bars indicated the number of genes up- or downregulated and total number of genes in each PseudoCAP category.

**
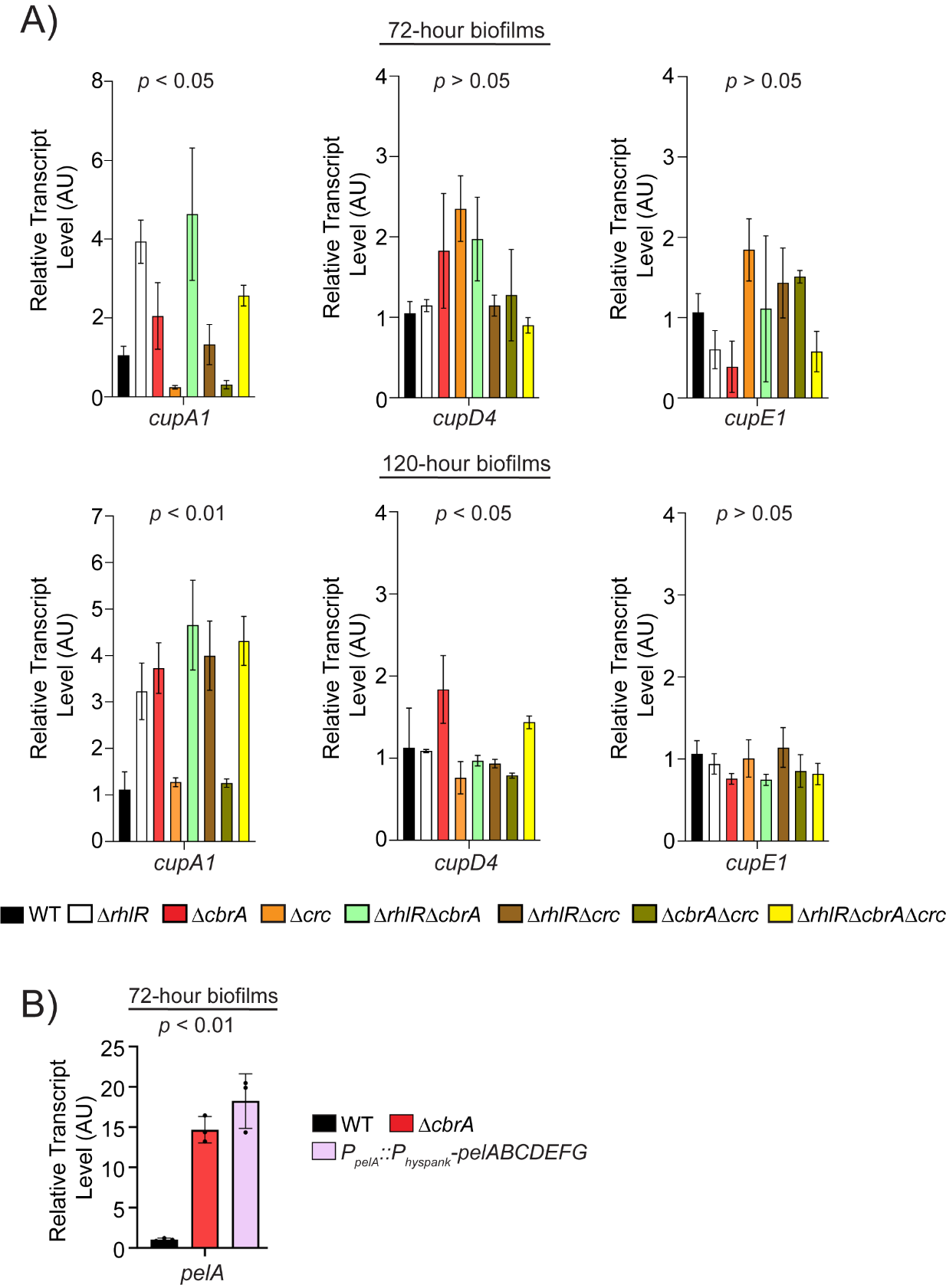
Supplemental Fig. S9: Expression of genes encoding biofilm matrix components in WT and mutant strains in this study.** A) Relative expression of *cupA1*, *cupD4*, *cupE1* genes normalized to 16S RNA, *ostA* and *rpsO* transcript levels measured by qRT-PCR in WT PA14 and indicated mutants after 72 h and 120 h of colony biofilm growth. AU denotes arbitrary units. Error bars represent standard deviation of three biological replicates. Statistical significance was determined using Welch’s ANOVA in GraphPad Prism software. For *cupA1* expression at 72 h, the following comparisons were statistically significant (p<0.05): WT vs Δ*rhlR*, and WT vs Δ*rhlR*Δ*cbrA.* For *cupA1* expression at 120 h, the following comparisons were statistically significant (p<0.05): Δ*crc* vs Δ*rhlR*Δ*cbrA*, Δ*crc* vs Δ*rhlR*Δ*crc* and Δ*crc* vs Δ*rhlR*Δ*cbrA*Δ*crc.* For *cupD4* expression at 120 h, the following comparisons were statistically significant (p<0.05): Δ*cbrA* vs Δ*rhlR*Δ*cbrA*, Δ*cbrA* vs Δ*rhlR*Δ*crc*, Δ*cbrA* vs Δ*cbrA*Δ*crc.* B) Relative expression of *pelA* gene normalized to 16S RNA, *ostA* and *rpsO* transcript levels measured by qRT-PCR in WT PA14 and indicated mutants after 72 h of colony biofilm growth. AU denotes arbitrary units. Error bars represent standard deviation of three biological replicates. Statistical significance was determined using Welch’s ANOVA in GraphPad Prism software; the following comparisons were statistically significant (p<0.01): WT vs all other strains.

**SUPPLEMENTAL TABLES**

**TABLE S1: Unique suppressor mutations of the Δ*rhlR*Δ*cbrA* biofilm phenotype**

| **Strain** | **PA14 ID^a^** | **Gene name** | **Nucleotide position** | **Mutation** |
| --- | --- | --- | --- | --- |
| SPa1068 | PA14_70390 | *crc* | 6275079 | +T (frameshift) |
| SPa1070 | PA14_70390 | *crc* | 6276133 | +T (frameshift) |
| SPa1073 | PA14_70390 | *crc* | 6275042 | M1I (ATG→ATA) |
| SPa1074 | PA14_70390 | *crc* | 6275704 | +G (frameshift) |
| SPa1078 | PA14_70390 | *crc* | 6275207 | +G (frameshift) |
| SPa1079 | PA14_70390 | *crc* | 6275254 | Δ2 bp (frameshift) |
| SPa1088 | PA14_08920 | *rplP* | 763531 | R82P (CGT→CCT) |
| SPa1090 | PA14_08850 | *rplC* | 759596 | T13N (ACC→AAC) |
| SPa1092 | PA14_70390 | *crc* | 6275734 | Δ55 bp (frameshift) |
| SPa1093 | PA14_70390 | *crc* | 6275223 | E61STOP (GAG→TAG) |
| SPa1095 | PA14_70390 | *crc* | 6275107 | W22STOP (TGG→TAG) |
| SPa1096 | PA14_70390 | *crc* | 6275757 | Δ9 bp |

a: annotation from www.pseudomonas.com (69)

**TABLE S2: Bacterial strains and plasmids**

| **Strain** | **Description** | **Reference** |
| --- | --- | --- |
| UCBPP-PA14 | Wild type *Pseudomonas aeruginosa* | Laboratory stock |
| SM32 | Δ*rhlR* | (25) |
| SM404 | Δ*pelA* | (25) |
| SPa1058 | Δ*crc* | This study |
| SPa1111 | Δ*cbrA* Δ*crc* | This study |
| SPa1117 | Δ*rhlR* Δ*cbrA* Δ*crc* pUCP18-*P_crc_-crc* | This study |
| SPa1128 | Δ*rhlR* Δ*crc* | This study |
| SPa1170 | Δ*cbrA* | This study |
| SPa1174 | Δ*rhlR* Δ*cbrA* | This study |
| SPa1183 | Δ*rhlR* Δ*cbrA* Δ*crc* | This study |
| SPa1187 | *cbrA^H766A^* pUCP18-*P_cbrA_-cbrA* | This study |
| SPa1189 | *cbrA^H766A^* pUCP18 | This study |
| SPa1191 | Δ*cbrA* pUCP18-*P_cbrA_-cbrA* | This study |
| SPa1193 | Δ*cbrA* pUCP18 | This study |
| SPa1200 | pUCP18-*crcZ* | This study |
| SPa1609 | Δ*cbrA* pUCP18-*crcZ* | This study |
| SPa1202 | Δ*rhlR* Δ*cbrA* pUCP18-*P_cbrA_-cbrA* | This study |
| SPa1206 | Δ*rhlR* Δ*cbrA* Δ*crc* pUCP18 | This study |
| SPa1220 | Δ*rhlR* Δ*cbrA* pUCP18-*crcZ* | This study |
| SPa1379 | Δ*cbrA* Δ*pelA* | This study |
| SPa594 | *P_pelA_*::*P_hyspank_-pelABCDEFG* | This study |
| SPa1232 | Δ*rhlR* *P_pelA_*::*P_hyspank_-pelABCDEFG* | This study |
| SPa1234 | Δ*crc* *P_pelA_*::*P_hyspank_-pelABCDEFG* | This study |
| SPa1236 | Δ*cbrA* *P_pelA_*::*P_hyspank_-pelABCDEFG* | This study |
| SPa1489 | Δ*cupB1-B5* | This study |
| SPa1487 | Δ*cbrA* Δ*cupB1-B5* | This study |
| SPa1565 | Δ*cupC1-C3* | This study |
| SPa1554 | Δ*cbrA* Δ*cupC1-C3* | This study |
| SPa1567 | *P_cupB_-lacZ* | This study |
| SPa1543 | Δ*cbrA* *P_cupB_-lacZ* | This study |
| SPa1484 | Δ*cbrA* Δ*crc P_cupB_-lacZ* | This study |
| SPA1468 | Δ*crc P_cupB_-lacZ* | This study |
| SPa1464 | *P_cupC_-lacZ* | This study |
| SPa1545 | Δ*cbrA* *P_cupC_-lacZ* | This study |
| SPa1542 | Δ*cbrA* Δ*crc P_cupC_-lacZ* | This study |
| SPa1470 | Δ*crc P_cupC_-lacZ* | This study |
| SM645 | Δ*phz (*Δ*phzA1-G1,* Δ*phzA2-G2)* | This study |
| SPa1646 | Δ*cbrA* Δ*phz* | This study |
| SPa1238 | Δ*rhlR* Δ*cbrA* Δ*phz* | This study |
| *E.coli* DH5α | F^–^ *endA1* *glnV44* *thi-1* *recA1* *relA1* *gyrA96* *deoR* *nupG* *purB20* φ80d*lacZ*ΔM15 Δ(*lacZYA-argF*) U169, hsdR17(*r_K_*^–^*m_K_*^+^), λ^–^ | Laboratory stock |
| *E.coli* SM10λ*pir* | *thi thr leu tonA lacY supE recA*::RP4-2-Tc::Mu | Laboratory stock |
| pEXG2 | Allelic exchange vector with pBR origin, gentamicin resistance, *sacB* gene | (65) |
| pUCP18 | E. coli-Pseudomonas Amp^r^ shuttle vector | Laboratory stock |

**TABLE S3: RNAseq datasets**

**TABLE S4: Crc binding site prediction in selected target transcripts.**
