# Supplementary material for: Combinatorial control of Pseudomonas aeruginosa biofilm development by quorum-sensing and nutrient-sensing regulators
Source: mSystems. 2024 Aug 14;9(9):e00372-24. doi: 10.1128/msystems.00372-24 (PMC11406991; doi:10.1128/msystems.00372-24)
Supplement: Table S3 — RNA-seq data sets. [file msystems.00372-24-s0002.pdf]

Table S3 RNAseq datasets

| locus_tag | pseudocap_category | gene_id | gene_nam | condition_comparison | log2FoldCl | padj |
|---|---|---|---|---|---|---|
| PA14_00230 | DNA_replication_recombinat | PGD1650875 | NA | DrhlR DcbrA vs WT | 1.150421 | 0.001878 |
| PA14_00230 | membrane_proteins | PGD1650875 | NA | DrhlR DcbrA vs WT | 1.150421 | 0.001878 |
| PA14_00310 | translation_posttranslational | PGD1650887 | NA | DrhlR DcbrA vs WT | 1.281358 | 4.73E-15 |
| PA14_00320 | hypothetical_unclassified | PGD1650889 | NA | DrhlR DcbrA vs WT | 1.227627 | 5.55E-08 |
| PA14_00430 | transcriptional_regulators | PGD1650903 | NA | DrhlR DcbrA vs WT | 1.142499 | 3.10E-04 |
| PA14_00430 | two_component_regulators | PGD1650903 | NA | DrhlR DcbrA vs WT | 1.142499 | 3.10E-04 |
| PA14_00450 | amino_acid_biosynthesis_me | PGD1650907 | trpB | DrhlR DcbrA vs WT | 1.062715 | 2.46E-05 |
| PA14_00490 | protein_secretion_export | PGD1650915 | NA | DrhlR DcbrA vs WT | 1.623477 | 4.28E-06 |
| PA14_00630 | hypothetical_unclassified | PGD1650937 | NA | DrhlR DcbrA vs WT | -1.89854 | 1.52E-21 |
| PA14_00640 | putative_enzymes | PGD1650939 | phzH | DrhlR DcbrA vs WT | -5.11489 | 3.24E-39 |
| PA14_00650 | hypothetical_unclassified | PGD1650941 | NA | DrhlR DcbrA vs WT | -1.92521 | 3.29E-36 |
| PA14_00860 | protein_secretion_export | PGD1650981 | tagT1 | DrhlR DcbrA vs WT | 1.374155 | 0.013445 |
| PA14_00860 | transport_of_small_molecule | PGD1650981 | tagT1 | DrhlR DcbrA vs WT | 1.374155 | 0.013445 |
| PA14_01290 | energy_metabolism_CAP | PGD1651049 | coxB | DrhlR DcbrA vs WT | 1.669764 | 1.94E-15 |
| PA14_01310 | central_intermediary_metab | PGD1651053 | NA | DrhlR DcbrA vs WT | 1.208381 | 2.69E-06 |
| PA14_01310 | energy_metabolism_CAP | PGD1651053 | NA | DrhlR DcbrA vs WT | 1.208381 | 2.69E-06 |
| PA14_01340 | hypothetical_unclassified | PGD1651059 | NA | DrhlR DcbrA vs WT | 1.459543 | 2.15E-13 |
| PA14_01350 | hypothetical_unclassified | PGD1651061 | NA | DrhlR DcbrA vs WT | 1.645772 | 1.74E-08 |
| PA14_01360 | energy_metabolism_CAP | PGD1651063 | NA | DrhlR DcbrA vs WT | 1.491087 | 3.09E-07 |
| PA14_01360 | membrane_proteins | PGD1651063 | NA | DrhlR DcbrA vs WT | 1.491087 | 3.09E-07 |
| PA14_01390 | energy_metabolism_CAP | PGD1651067 | NA | DrhlR DcbrA vs WT | -1.48885 | 1.46E-20 |
| PA14_01490 | adaptation_and_protection | PGD1651083 | NA | DrhlR DcbrA vs WT | -4.88648 | 2.27E-52 |
| PA14_01490 | putative_enzymes | PGD1651083 | NA | DrhlR DcbrA vs WT | -4.88648 | 2.27E-52 |
| PA14_01660 | nucleotide_biosynthesis_and | PGD1651107 | NA | DrhlR DcbrA vs WT | 1.103898 | 1.03E-08 |
| PA14_02220 | chemotaxis | PGD1651191 | NA | DrhlR DcbrA vs WT | -1.00756 | 7.99E-09 |
| PA14_02370 | membrane_proteins | PGD1651213 | NA | DrhlR DcbrA vs WT | 1.375808 | 0.041401 |
| PA14_02510 | transport_of_small_molecule | PGD1651235 | exbD1 | DrhlR DcbrA vs WT | -1.51285 | 0.017085 |
| PA14_02610 | carbon_compound_catabolis | PGD1651251 | NA | DrhlR DcbrA vs WT | 1.124579 | 0.005211 |
| PA14_02620 | carbon_compound_catabolis | PGD1651253 | NA | DrhlR DcbrA vs WT | 1.640153 | 1.13E-06 |
| PA14_02760 | carbon_compound_catabolis | PGD1651277 | NA | DrhlR DcbrA vs WT | -2.16902 | 3.93E-14 |
| PA14_02770 | carbon_compound_catabolis | PGD1651279 | NA | DrhlR DcbrA vs WT | -2.21629 | 7.54E-09 |
| PA14_02790 | carbon_compound_catabolis | PGD1651281 | pcaF | DrhlR DcbrA vs WT | -1.44618 | 1.03E-07 |
| PA14_02790 | fatty_acid_and_phospholipid | PGD1651281 | pcaF | DrhlR DcbrA vs WT | -1.44618 | 1.03E-07 |
| PA14_02830 | carbon_compound_catabolis | PGD1651285 | pcaB | DrhlR DcbrA vs WT | 1.000883 | 0.002913 |
| PA14_02900 | carbon_compound_catabolis | PGD1651295 | pcaK | DrhlR DcbrA vs WT | -3.51645 | 1.95E-24 |
| PA14_02900 | membrane_proteins | PGD1651295 | pcaK | DrhlR DcbrA vs WT | -3.51645 | 1.95E-24 |
| PA14_02900 | transport_of_small_molecule | PGD1651295 | pcaK | DrhlR DcbrA vs WT | -3.51645 | 1.95E-24 |
| PA14_02910 | transcriptional_regulators | PGD1651297 | NA | DrhlR DcbrA vs WT | -1.75952 | 6.60E-14 |
| PA14_02970 | membrane_proteins | PGD1651303 | NA | DrhlR DcbrA vs WT | 1.582189 | 3.72E-06 |
| PA14_02990 | transport_of_small_molecule | PGD1651307 | NA | DrhlR DcbrA vs WT | -1.79095 | 0.002162 |
| PA14_03240 | secreted_factors | PGD1651355 | hcpC | DrhlR DcbrA vs WT | -1.74649 | 4.00E-09 |
| PA14_03490 | carbon_compound_catabolis | PGD1651401 | NA | DrhlR DcbrA vs WT | -4.18896 | 7.73E-107 |
| PA14_03510 | hypothetical_unclassified | PGD1651403 | NA | DrhlR DcbrA vs WT | -4.24185 | 4.47E-43 |
| PA14_03520 | hypothetical_unclassified | PGD1651405 | NA | DrhlR DcbrA vs WT | -3.63704 | 2.33E-36 |
| PA14_03550 | transport_of_small_molecule | PGD1651409 | NA | DrhlR DcbrA vs WT | 1.619562 | 0.010562 |
| PA14_03650 | transport_of_small_molecule | PGD1651423 | cysA | DrhlR DcbrA vs WT | -1.54794 | 1.22E-08 |
| PA14_03670 | membrane_proteins | PGD1651425 | cysW | DrhlR DcbrA vs WT | -1.71753 | 5.99E-07 |
| PA14_03670 | transport_of_small_molecule | PGD1651425 | cysW | DrhlR DcbrA vs WT | -1.71753 | 5.99E-07 |
| PA14_03680 | membrane_proteins | PGD1651427 | cysT | DrhlR DcbrA vs WT | -1.77297 | 2.81E-06 |

| | | | | | | |
|---|---|---|---|---|---|---|
| PA14_03680 | transport_of_small_molecule | PGD1651427 | cysT | DrhlR DcbrA vs WT | -1.77297 | 2.81E-06 |
| PA14_03700 | transport_of_small_molecule | PGD1651429 | sbp | DrhlR DcbrA vs WT | -1.82769 | 1.15E-08 |
| PA14_03710 | hypothetical_unclassified | PGD1651431 | NA | DrhlR DcbrA vs WT | -1.08044 | 0.024671 |
| PA14_04230 | membrane_proteins | PGD1651513 | NA | DrhlR DcbrA vs WT | -2.36993 | 9.86E-04 |
| PA14_04230 | transport_of_small_molecule | PGD1651513 | NA | DrhlR DcbrA vs WT | -2.36993 | 9.86E-04 |
| PA14_04250 | transport_of_small_molecule | PGD1651517 | NA | DrhlR DcbrA vs WT | -1.40083 | 0.018373 |
| PA14_04520 | membrane_proteins | PGD1651557 | NA | DrhlR DcbrA vs WT | 1.381884 | 6.72E-12 |
| PA14_04580 | biosynthesis_of_cofactors | PGD1651567 | folA | DrhlR DcbrA vs WT | -1.08765 | 5.88E-04 |
| PA14_04580 | central_intermediary_metab | PGD1651567 | folA | DrhlR DcbrA vs WT | -1.08765 | 5.88E-04 |
| PA14_04640 | transport_of_small_molecule | PGD1651575 | NA | DrhlR DcbrA vs WT | 1.543408 | 6.34E-16 |
| PA14_04650 | translation_posttranslational | PGD1651577 | pfpI | DrhlR DcbrA vs WT | 1.422794 | 7.11E-13 |
| PA14_05110 | hypothetical_unclassified | PGD1651653 | NA | DrhlR DcbrA vs WT | 1.580023 | 1.24E-16 |
| PA14_05380 | chemotaxis | PGD1651697 | pilK | DrhlR DcbrA vs WT | 1.096752 | 3.62E-10 |
| PA14_05410 | chemotaxis | PGD1651703 | NA | DrhlR DcbrA vs WT | 1.431462 | 2.26E-10 |
| PA14_05420 | transcriptional_regulators | PGD1651705 | NA | DrhlR DcbrA vs WT | 1.405788 | 4.15E-08 |
| PA14_05630 | hypothetical_unclassified | PGD1651739 | NA | DrhlR DcbrA vs WT | 1.321354 | 8.99E-05 |
| PA14_05660 | transcriptional_regulators | PGD1651745 | NA | DrhlR DcbrA vs WT | -2.03091 | 1.19E-39 |
| PA14_05740 | nucleotide_biosynthesis_and | PGD1651751 | NA | DrhlR DcbrA vs WT | 2.035298 | 0.006604 |
| PA14_05740 | putative_enzymes | PGD1651751 | NA | DrhlR DcbrA vs WT | 2.035298 | 0.006604 |
| PA14_05750 | amino_acid_biosynthesis_me | PGD1651753 | NA | DrhlR DcbrA vs WT | 1.268433 | 0.030387 |
| PA14_05750 | putative_enzymes | PGD1651753 | NA | DrhlR DcbrA vs WT | 1.268433 | 0.030387 |
| PA14_05770 | nucleotide_biosynthesis_and | PGD1651755 | dhT | DrhlR DcbrA vs WT | 1.44018 | 3.09E-08 |
| PA14_05890 | membrane_proteins | PGD1651775 | NA | DrhlR DcbrA vs WT | -1.24754 | 9.33E-08 |
| PA14_06120 | amino_acid_biosynthesis_me | PGD1651805 | NA | DrhlR DcbrA vs WT | -1.24338 | 3.54E-05 |
| PA14_06180 | transcriptional_regulators | PGD1651815 | NA | DrhlR DcbrA vs WT | 1.11125 | 0.002577 |
| PA14_06260 | transcriptional_regulators | PGD1651829 | NA | DrhlR DcbrA vs WT | -1.02458 | 1.29E-05 |
| PA14_06360 | nucleotide_biosynthesis_and | PGD1651849 | NA | DrhlR DcbrA vs WT | 1.191324 | 7.62E-04 |
| PA14_06360 | putative_enzymes | PGD1651849 | NA | DrhlR DcbrA vs WT | 1.191324 | 7.62E-04 |
| PA14_06390 | hypothetical_unclassified | PGD1651851 | NA | DrhlR DcbrA vs WT | 1.153999 | 9.43E-07 |
| PA14_06430 | putative_enzymes | PGD1651857 | NA | DrhlR DcbrA vs WT | 1.430227 | 0.002957 |
| PA14_06790 | energy_metabolism_CAP | PGD1651907 | NA | DrhlR DcbrA vs WT | -1.25318 | 1.62E-04 |
| PA14_06800 | hypothetical_unclassified | PGD1651909 | NA | DrhlR DcbrA vs WT | -1.62295 | 1.58E-04 |
| PA14_06830 | energy_metabolism_CAP | PGD1651913 | norB | DrhlR DcbrA vs WT | -1.1936 | 2.23E-05 |
| PA14_06840 | energy_metabolism_CAP | PGD1651915 | NA | DrhlR DcbrA vs WT | -1.09083 | 1.02E-04 |
| PA14_06860 | hypothetical_unclassified | PGD1651917 | NA | DrhlR DcbrA vs WT | 1.000873 | 6.28E-04 |
| PA14_06960 | carbon_compound_catabolis | PGD1651937 | NA | DrhlR DcbrA vs WT | -1.67049 | 5.39E-14 |
| PA14_06960 | energy_metabolism_CAP | PGD1651937 | NA | DrhlR DcbrA vs WT | -1.67049 | 5.39E-14 |
| PA14_06960 | putative_enzymes | PGD1651937 | NA | DrhlR DcbrA vs WT | -1.67049 | 5.39E-14 |
| PA14_06970 | transcriptional_regulators | PGD1651939 | NA | DrhlR DcbrA vs WT | -1.41059 | 6.46E-19 |
| PA14_07030 | energy_metabolism_CAP | PGD1651951 | NA | DrhlR DcbrA vs WT | -1.28233 | 6.71E-21 |
| PA14_07200 | hypothetical_unclassified | PGD1651975 | NA | DrhlR DcbrA vs WT | 1.264775 | 1.52E-16 |
| PA14_07370 | membrane_proteins | PGD1652003 | NA | DrhlR DcbrA vs WT | 1.117291 | 9.69E-07 |
| PA14_07550 | hypothetical_unclassified | PGD1652031 | NA | DrhlR DcbrA vs WT | -1.42084 | 4.75E-07 |
| PA14_07560 | translation_posttranslational | PGD1652033 | rpsU | DrhlR DcbrA vs WT | -1.20719 | 1.09E-07 |
| PA14_07850 | transport_of_small_molecule | PGD1652079 | NA | DrhlR DcbrA vs WT | 1.475484 | 5.23E-11 |
| PA14_08270 | hypothetical_unclassified | PGD1652155 | NA | DrhlR DcbrA vs WT | 1.342534 | 0.030727 |
| NA | NA | PGD1652224 | NA | DrhlR DcbrA vs WT | -1.00781 | 0.00305 |
| PA14_08840 | transcription_RNA_processin | PGD1652257 | rpsJ | DrhlR DcbrA vs WT | -1.03074 | 8.91E-05 |
| PA14_08840 | translation_posttranslational | PGD1652257 | rpsJ | DrhlR DcbrA vs WT | -1.03074 | 8.91E-05 |
| PA14_08860 | transcription_RNA_processin | PGD1652261 | rplD | DrhlR DcbrA vs WT | -1.01468 | 2.94E-07 |

| | | | | | | |
|---|---|---|---|---|---|---|
| PA14_08860 | translation_posttranslational | PGD1652261 | rplD | DrhlR DcbrA vs WT | -1.01468 | 2.94E-07 |
| PA14_08920 | translation_posttranslational | PGD1652273 | rplP | DrhlR DcbrA vs WT | -1.15557 | 3.38E-10 |
| PA14_08940 | translation_posttranslational | PGD1652277 | rpsQ | DrhlR DcbrA vs WT | -1.00279 | 7.29E-06 |
| PA14_09150 | adaptation_and_protection | PGD1652313 | katA | DrhlR DcbrA vs WT | -1.22088 | 3.26E-10 |
| PA14_09400 | putative_enzymes | PGD1652351 | phzS | DrhlR DcbrA vs WT | -2.24919 | 2.64E-14 |
| PA14_09410 | secreted_factors | PGD1652353 | phzG1 | DrhlR DcbrA vs WT | -2.0246 | 0.001695 |
| PA14_09440 | secreted_factors | PGD1652357 | phzE1 | DrhlR DcbrA vs WT | -3.52637 | 5.95E-20 |
| PA14_09450 | secreted_factors | PGD1652359 | phzD1 | DrhlR DcbrA vs WT | -7.06561 | 1.57E-07 |
| PA14_09460 | secreted_factors | PGD1652361 | phzC1 | DrhlR DcbrA vs WT | -2.59648 | 1.43E-05 |
| PA14_09470 | secreted_factors | PGD1652363 | phzB1 | DrhlR DcbrA vs WT | -2.30018 | 1.28E-06 |
| PA14_09480 | secreted_factors | PGD1652365 | phzA1 | DrhlR DcbrA vs WT | -6.54533 | 9.22E-07 |
| PA14_09490 | putative_enzymes | PGD1652367 | phzM | DrhlR DcbrA vs WT | -1.48269 | 9.77E-12 |
| PA14_09500 | membrane_proteins | PGD1652369 | opmD | DrhlR DcbrA vs WT | -4.55601 | 1.15E-23 |
| PA14_09500 | transport_of_small_molecule | PGD1652369 | opmD | DrhlR DcbrA vs WT | -4.55601 | 1.15E-23 |
| PA14_09520 | transport_of_small_molecule | PGD1652371 | mexI | DrhlR DcbrA vs WT | -3.74856 | 3.17E-57 |
| PA14_09530 | transport_of_small_molecule | PGD1652373 | mexH | DrhlR DcbrA vs WT | -6.02841 | 1.67E-31 |
| PA14_09540 | membrane_proteins | PGD1652375 | mexG | DrhlR DcbrA vs WT | -5.58685 | 4.52E-29 |
| PA14_10330 | protein_secretion_export | PGD1652499 | NA | DrhlR DcbrA vs WT | 1.58305 | 8.98E-21 |
| PA14_10340 | protein_secretion_export | PGD1652501 | NA | DrhlR DcbrA vs WT | 1.752912 | 5.13E-13 |
| PA14_10360 | hypothetical_unclassified | PGD1652505 | NA | DrhlR DcbrA vs WT | -3.01214 | 8.21E-46 |
| PA14_10370 | fatty_acid_and_phospholipid | PGD1652507 | NA | DrhlR DcbrA vs WT | 2.468529 | 5.71E-21 |
| PA14_10380 | hypothetical_unclassified | PGD1652509 | NA | DrhlR DcbrA vs WT | 2.199461 | 1.04E-49 |
| PA14_10500 | energy_metabolism_CAP | PGD1652525 | NA | DrhlR DcbrA vs WT | -1.14336 | 3.14E-17 |
| PA14_10530 | transcriptional_regulators | PGD1652527 | NA | DrhlR DcbrA vs WT | -1.44688 | 3.06E-30 |
| PA14_10540 | energy_metabolism_CAP | PGD1652529 | NA | DrhlR DcbrA vs WT | -1.75407 | 9.95E-39 |
| PA14_10550 | central_intermediary_metab | PGD1652531 | NA | DrhlR DcbrA vs WT | -1.09002 | 2.55E-52 |
| PA14_10560 | hypothetical_unclassified | PGD1652533 | NA | DrhlR DcbrA vs WT | -1.24631 | 3.49E-08 |
| PA14_10650 | central_intermediary_metab | PGD1652549 | NA | DrhlR DcbrA vs WT | -1.59068 | 0.03061 |
| PA14_10850 | putative_enzymes | PGD1652581 | NA | DrhlR DcbrA vs WT | -1.01064 | 0.00161 |
| PA14_10950 | hypothetical_unclassified | PGD1652595 | NA | DrhlR DcbrA vs WT | -1.30854 | 5.41E-04 |
| PA14_10990 | carbon_compound_catabolis | PGD1652603 | hpaC | DrhlR DcbrA vs WT | -2.18197 | 5.75E-09 |
| PA14_11000 | carbon_compound_catabolis | PGD1652605 | hpaA | DrhlR DcbrA vs WT | -1.03604 | 2.35E-06 |
| PA14_11110 | motility_and_attachment | PGD1652625 | cupB6 | DrhlR DcbrA vs WT | 1.144611 | 0.006788 |
| PA14_11130 | putative_enzymes | PGD1652629 | NA | DrhlR DcbrA vs WT | -1.60577 | 7.35E-24 |
| PA14_11140 | biosynthesis_of_cofactors | PGD1652631 | NA | DrhlR DcbrA vs WT | -4.64104 | 7.57E-91 |
| PA14_11150 | transcriptional_regulators | PGD1652633 | NA | DrhlR DcbrA vs WT | -1.33845 | 0.021058 |
| PA14_11190 | putative_enzymes | PGD1652641 | NA | DrhlR DcbrA vs WT | -1.26234 | 2.60E-08 |
| PA14_11240 | transcriptional_regulators | PGD1652647 | NA | DrhlR DcbrA vs WT | -1.31176 | 6.44E-17 |
| PA14_11730 | translation_posttranslational | PGD1652729 | NA | DrhlR DcbrA vs WT | 1.217752 | 1.23E-04 |
| PA14_12640 | hypothetical_unclassified | PGD1652863 | NA | DrhlR DcbrA vs WT | -1.39149 | 0.021208 |
| PA14_12740 | hypothetical_unclassified | PGD1652879 | NA | DrhlR DcbrA vs WT | 1.008471 | 3.35E-04 |
| PA14_12920 | transport_of_small_molecule | PGD1652907 | NA | DrhlR DcbrA vs WT | -2.13111 | 1.71E-07 |
| PA14_12940 | transport_of_small_molecule | PGD1652909 | NA | DrhlR DcbrA vs WT | -2.31007 | 3.49E-06 |
| PA14_13010 | hypothetical_unclassified | PGD1652921 | NA | DrhlR DcbrA vs WT | -1.13608 | 6.15E-04 |
| PA14_13130 | hypothetical_unclassified | PGD1652937 | NA | DrhlR DcbrA vs WT | -1.67862 | 2.46E-17 |
| PA14_13140 | hypothetical_unclassified | PGD1652939 | NA | DrhlR DcbrA vs WT | -2.21428 | 2.76E-25 |
| PA14_13170 | transport_of_small_molecule | PGD1652943 | NA | DrhlR DcbrA vs WT | -3.74885 | 1.50E-52 |
| PA14_13190 | hypothetical_unclassified | PGD1652945 | NA | DrhlR DcbrA vs WT | -1.01079 | 3.55E-08 |
| PA14_13210 | hypothetical_unclassified | PGD1652949 | NA | DrhlR DcbrA vs WT | -3.30817 | 2.54E-49 |
| PA14_13230 | biosynthesis_of_cofactors | PGD1652953 | moaC | DrhlR DcbrA vs WT | -1.20781 | 3.67E-14 |

| | | | | | | |
|---|---|---|---|---|---|---|
| PA14_13240 | biosynthesis_of_cofactors | PGD1652955 | moaD | DrhlR DcbrA vs WT | -1.75311 | 2.54E-09 |
| PA14_13250 | biosynthesis_of_cofactors | PGD1652957 | moaE | DrhlR DcbrA vs WT | -1.42395 | 3.26E-10 |
| PA14_13260 | biosynthesis_of_cofactors | PGD1652959 | moaB1 | DrhlR DcbrA vs WT | -1.90767 | 1.04E-12 |
| PA14_13280 | biosynthesis_of_cofactors | PGD1652961 | moeA1 | DrhlR DcbrA vs WT | -1.84348 | 7.87E-09 |
| PA14_13300 | translation_posttranslational | PGD1652965 | NA | DrhlR DcbrA vs WT | -1.18444 | 4.53E-05 |
| PA14_13340 | transcription_RNA_processin | PGD1652971 | NA | DrhlR DcbrA vs WT | -1.3041 | 3.60E-06 |
| PA14_13450 | membrane_proteins | PGD1652989 | NA | DrhlR DcbrA vs WT | 1.171514 | 0.016479 |
| PA14_13460 | transcriptional_regulators | PGD1652991 | NA | DrhlR DcbrA vs WT | 1.376027 | 0.011262 |
| PA14_13580 | transport_of_small_molecule | PGD1653007 | NA | DrhlR DcbrA vs WT | 1.237758 | 2.67E-05 |
| PA14_13590 | membrane_proteins | PGD1653009 | NA | DrhlR DcbrA vs WT | 1.313277 | 8.15E-06 |
| PA14_13590 | transport_of_small_molecule | PGD1653009 | NA | DrhlR DcbrA vs WT | 1.313277 | 8.15E-06 |
| PA14_13610 | membrane_proteins | PGD1653013 | NA | DrhlR DcbrA vs WT | 1.056745 | 7.87E-07 |
| PA14_13610 | transport_of_small_molecule | PGD1653013 | NA | DrhlR DcbrA vs WT | 1.056745 | 7.87E-07 |
| PA14_13630 | hypothetical_unclassified | PGD1653017 | NA | DrhlR DcbrA vs WT | 1.484016 | 9.26E-13 |
| PA14_13770 | transport_of_small_molecule | PGD1653039 | narK2 | DrhlR DcbrA vs WT | -1.00924 | 6.02E-05 |
| PA14_13780 | energy_metabolism_CAP | PGD1653041 | narG | DrhlR DcbrA vs WT | -1.24832 | 4.89E-08 |
| PA14_13800 | energy_metabolism_CAP | PGD1653043 | narH | DrhlR DcbrA vs WT | -1.26696 | 1.71E-07 |
| PA14_13810 | energy_metabolism_CAP | PGD1653045 | narJ | DrhlR DcbrA vs WT | -1.1957 | 7.54E-04 |
| PA14_13830 | energy_metabolism_CAP | PGD1653047 | narI | DrhlR DcbrA vs WT | -1.54679 | 3.58E-08 |
| PA14_13840 | chaperones_heat_shock | PGD1653049 | NA | DrhlR DcbrA vs WT | -1.25088 | 2.98E-04 |
| PA14_13840 | translation_posttranslational | PGD1653049 | NA | DrhlR DcbrA vs WT | -1.25088 | 2.98E-04 |
| PA14_13850 | biosynthesis_of_cofactors | PGD1653051 | moaA | DrhlR DcbrA vs WT | -1.09545 | 0.00116 |
| PA14_14320 | hypothetical_unclassified | PGD1653121 | NA | DrhlR DcbrA vs WT | -1.07914 | 0.029346 |
| PA14_14370 | transport_of_small_molecule | PGD1653129 | NA | DrhlR DcbrA vs WT | -1.00961 | 8.53E-07 |
| PA14_14560 | hypothetical_unclassified | PGD1653165 | NA | DrhlR DcbrA vs WT | -1.18227 | 4.48E-05 |
| PA14_14610 | protein_secretion_export | PGD1653173 | yajC | DrhlR DcbrA vs WT | -1.00965 | 1.71E-07 |
| PA14_15050 | membrane_proteins | PGD1653235 | NA | DrhlR DcbrA vs WT | -1.12628 | 4.60E-18 |
| PA14_15070 | transport_of_small_molecule | PGD1653237 | oprC | DrhlR DcbrA vs WT | -1.16872 | 2.91E-11 |
| PA14_16100 | hypothetical_unclassified | PGD1653413 | NA | DrhlR DcbrA vs WT | -2.65146 | 1.79E-35 |
| PA14_16250 | amino_acid_biosynthesis_me | PGD1653435 | lasB | DrhlR DcbrA vs WT | -4.00675 | 6.50E-41 |
| PA14_16250 | secreted_factors | PGD1653435 | lasB | DrhlR DcbrA vs WT | -4.00675 | 6.50E-41 |
| PA14_16250 | translation_posttranslational | PGD1653435 | lasB | DrhlR DcbrA vs WT | -4.00675 | 6.50E-41 |
| PA14_16310 | membrane_proteins | PGD1653447 | NA | DrhlR DcbrA vs WT | -1.88603 | 9.40E-10 |
| PA14_16310 | transport_of_small_molecule | PGD1653447 | NA | DrhlR DcbrA vs WT | -1.88603 | 9.40E-10 |
| PA14_16410 | transport_of_small_molecule | PGD1653465 | NA | DrhlR DcbrA vs WT | -1.21717 | 1.45E-05 |
| PA14_16680 | hypothetical_unclassified | PGD1653507 | NA | DrhlR DcbrA vs WT | -1.2862 | 3.43E-14 |
| PA14_16990 | hypothetical_unclassified | PGD1653559 | NA | DrhlR DcbrA vs WT | 3.412398 | 5.99E-20 |
| PA14_17480 | transcriptional_regulators | PGD1653639 | rpoS | DrhlR DcbrA vs WT | -1.00791 | 8.72E-08 |
| PA14_17700 | translation_posttranslational | PGD1653681 | rpmE2 | DrhlR DcbrA vs WT | -1.00414 | 4.86E-04 |
| PA14_17810 | putative_enzymes | PGD1653697 | NA | DrhlR DcbrA vs WT | 1.262102 | 0.013154 |
| PA14_17910 | putative_enzymes | PGD1653711 | NA | DrhlR DcbrA vs WT | 1.337064 | 2.85E-06 |
| PA14_18020 | secreted_factors | PGD1653727 | NA | DrhlR DcbrA vs WT | 1.425701 | 2.46E-17 |
| PA14_18100 | hypothetical_unclassified | PGD1653741 | NA | DrhlR DcbrA vs WT | 1.372819 | 8.17E-05 |
| PA14_18565 | adaptation_and_protection | PGD1653803 | alg8 | DrhlR DcbrA vs WT | 1.090843 | 0.015822 |
| PA14_18565 | cell_wall_LPS | PGD1653803 | alg8 | DrhlR DcbrA vs WT | 1.090843 | 0.015822 |
| PA14_18565 | secreted_factors | PGD1653803 | alg8 | DrhlR DcbrA vs WT | 1.090843 | 0.015822 |
| PA14_18630 | putative_enzymes | PGD1653815 | NA | DrhlR DcbrA vs WT | -1.23592 | 2.46E-15 |
| PA14_18800 | transport_of_small_molecule | PGD1653845 | NA | DrhlR DcbrA vs WT | -5.27642 | 6.51E-55 |
| PA14_18810 | hypothetical_unclassified | PGD1653847 | NA | DrhlR DcbrA vs WT | -2.36025 | 1.41E-13 |
| PA14_18850 | carbon_compound_catabolis | PGD1653853 | NA | DrhlR DcbrA vs WT | 1.375148 | 0.001487 |

| | | | | | | |
|---|---|---|---|---|---|---|
| PA14_18850 | putative_enzymes | PGD1653853 | NA | DrhlR DcbrA vs WT | 1.375148 | 0.001487 |
| PA14_18960 | hypothetical_unclassified | PGD1653873 | tli5a | DrhlR DcbrA vs WT | -1.93899 | 1.41E-16 |
| PA14_18970 | fatty_acid_and_phospholipid | PGD1653875 | pldA | DrhlR DcbrA vs WT | -1.41558 | 3.56E-17 |
| PA14_18985 | hypothetical_unclassified | PGD1653877 | vgrG4b | DrhlR DcbrA vs WT | -1.66164 | 1.03E-13 |
| PA14_19100 | secreted_factors | PGD1653891 | rhlA | DrhlR DcbrA vs WT | -6.75203 | 2.27E-37 |
| PA14_19120 | transcriptional_regulators | PGD1653895 | rhlR | DrhlR DcbrA vs WT | -6.05744 | 1.48E-51 |
| PA14_19140 | amino_acid_biosynthesis_me | PGD1653899 | pheC | DrhlR DcbrA vs WT | -1.15345 | 1.20E-14 |
| PA14_19350 | carbon_compound_catabolis | PGD1653927 | NA | DrhlR DcbrA vs WT | 1.073064 | 3.63E-05 |
| PA14_19360 | putative_enzymes | PGD1653929 | NA | DrhlR DcbrA vs WT | 1.098621 | 1.93E-05 |
| PA14_19500 | transport_of_small_molecule | PGD1653951 | NA | DrhlR DcbrA vs WT | -1.2256 | 0.021217 |
| PA14_19590 | central_intermediary_metab | PGD1653967 | NA | DrhlR DcbrA vs WT | -2.35573 | 2.62E-04 |
| PA14_19750 | hypothetical_unclassified | PGD1653999 | NA | DrhlR DcbrA vs WT | -1.47396 | 0.001271 |
| PA14_19920 | energy_metabolism_CAP | PGD1654019 | NA | DrhlR DcbrA vs WT | -1.06429 | 2.35E-07 |
| PA14_20060 | hypothetical_unclassified | PGD1654045 | NA | DrhlR DcbrA vs WT | 1.026875 | 3.49E-06 |
| PA14_20140 | biosynthesis_of_cofactors | PGD1654059 | fpr | DrhlR DcbrA vs WT | -1.54977 | 1.41E-11 |
| PA14_20140 | energy_metabolism_CAP | PGD1654059 | fpr | DrhlR DcbrA vs WT | -1.54977 | 1.41E-11 |
| PA14_20250 | antibiotic_resistance_and_su | PGD1654075 | NA | DrhlR DcbrA vs WT | 1.420403 | 4.03E-05 |
| PA14_20470 | membrane_proteins | PGD1654113 | NA | DrhlR DcbrA vs WT | 1.010504 | 1.53E-05 |
| PA14_20530 | hypothetical_unclassified | PGD1654125 | NA | DrhlR DcbrA vs WT | 1.386739 | 0.001523 |
| PA14_20560 | carbon_compound_catabolis | PGD1654129 | amiE | DrhlR DcbrA vs WT | 1.260684 | 2.64E-09 |
| PA14_20610 | motility_and_attachment | PGD1654139 | lecB | DrhlR DcbrA vs WT | -7.45339 | 1.21E-33 |
| PA14_20620 | secreted_factors | PGD1654141 | NA | DrhlR DcbrA vs WT | 1.03247 | 0.005038 |
| PA14_20900 | membrane_proteins | PGD1654189 | NA | DrhlR DcbrA vs WT | -2.41391 | 5.43E-18 |
| PA14_20920 | hypothetical_unclassified | PGD1654191 | NA | DrhlR DcbrA vs WT | -1.4483 | 7.59E-16 |
| PA14_20940 | fatty_acid_and_phospholipid | PGD1654193 | NA | DrhlR DcbrA vs WT | -3.27217 | 3.43E-20 |
| PA14_20950 | fatty_acid_and_phospholipid | PGD1654195 | fabH2 | DrhlR DcbrA vs WT | -3.38651 | 2.00E-26 |
| PA14_20960 | putative_enzymes | PGD1654197 | NA | DrhlR DcbrA vs WT | -2.69404 | 1.41E-12 |
| PA14_20970 | adaptation_and_protection | PGD1654199 | cyp23 | DrhlR DcbrA vs WT | -3.51849 | 3.25E-26 |
| PA14_20980 | putative_enzymes | PGD1654201 | NA | DrhlR DcbrA vs WT | -2.50622 | 1.89E-19 |
| PA14_21000 | hypothetical_unclassified | PGD1654203 | NA | DrhlR DcbrA vs WT | -3.47503 | 8.06E-29 |
| PA14_21010 | energy_metabolism_CAP | PGD1654205 | NA | DrhlR DcbrA vs WT | -3.17069 | 5.33E-21 |
| PA14_21020 | biosynthesis_of_cofactors | PGD1654207 | NA | DrhlR DcbrA vs WT | -2.25487 | 2.76E-25 |
| PA14_21030 | cell_wall_LPS | PGD1654209 | NA | DrhlR DcbrA vs WT | -2.1533 | 7.64E-37 |
| PA14_21030 | translation_posttranslational | PGD1654209 | NA | DrhlR DcbrA vs WT | -2.1533 | 7.64E-37 |
| PA14_21450 | hypothetical_unclassified | PGD1654275 | NA | DrhlR DcbrA vs WT | -1.09133 | 7.86E-08 |
| PA14_21460 | hypothetical_unclassified | PGD1654277 | NA | DrhlR DcbrA vs WT | -1.11314 | 1.50E-04 |
| PA14_21470 | hypothetical_unclassified | PGD1654279 | NA | DrhlR DcbrA vs WT | -1.41957 | 5.10E-09 |
| PA14_21480 | hypothetical_unclassified | PGD1654281 | tli1 | DrhlR DcbrA vs WT | -1.52789 | 2.35E-06 |
| PA14_21570 | hypothetical_unclassified | PGD1654297 | NA | DrhlR DcbrA vs WT | 2.482333 | 6.47E-05 |
| PA14_21580 | hypothetical_unclassified | PGD1654299 | NA | DrhlR DcbrA vs WT | 1.895049 | 0.007423 |
| PA14_21590 | hypothetical_unclassified | PGD1654301 | NA | DrhlR DcbrA vs WT | 1.738243 | 0.023368 |
| PA14_21600 | membrane_proteins | PGD1654303 | NA | DrhlR DcbrA vs WT | 2.417912 | 8.49E-04 |
| PA14_21610 | transport_of_small_molecule | PGD1654305 | oprO | DrhlR DcbrA vs WT | 1.488919 | 1.43E-05 |
| PA14_21630 | membrane_proteins | PGD1654309 | NA | DrhlR DcbrA vs WT | 1.693627 | 3.86E-11 |
| PA14_21670 | hypothetical_unclassified | PGD1654317 | NA | DrhlR DcbrA vs WT | 1.541304 | 3.11E-14 |
| PA14_21680 | hypothetical_unclassified | PGD1654319 | NA | DrhlR DcbrA vs WT | 1.476325 | 1.36E-09 |
| PA14_21830 | hypothetical_unclassified | PGD1654347 | NA | DrhlR DcbrA vs WT | 1.066996 | 1.09E-14 |
| PA14_22110 | hypothetical_unclassified | PGD1654399 | NA | DrhlR DcbrA vs WT | 1.165741 | 0.004187 |
| PA14_22220 | hypothetical_unclassified | PGD1654415 | NA | DrhlR DcbrA vs WT | -1.45696 | 0.013685 |
| PA14_22320 | membrane_proteins | PGD1654433 | NA | DrhlR DcbrA vs WT | -2.36223 | 1.19E-06 |

| | | | | | | |
|---|---|---|---|---|---|---|
| PA14_22340 | membrane_proteins | PGD1654437 | NA | DrhlR DcbrA vs WT | 1.188819 | 5.84E-06 |
| PA14_22740 | transport_of_small_molecule | PGD1654509 | NA | DrhlR DcbrA vs WT | 1.061648 | 0.046184 |
| PA14_22880 | hypothetical_unclassified | PGD1654529 | NA | DrhlR DcbrA vs WT | 1.798516 | 1.18E-05 |
| PA14_22990 | transport_of_small_molecule | PGD1654543 | NA | DrhlR DcbrA vs WT | -1.05726 | 2.63E-06 |
| PA14_23680 | chaperones_heat_shock | PGD1654661 | ibpA | DrhlR DcbrA vs WT | 2.106727 | 1.68E-07 |
| PA14_23970 | protein_secretion_export | PGD1654703 | xcpQ | DrhlR DcbrA vs WT | -1.22232 | 2.88E-23 |
| NA | NA | PGD1654728 | NA | DrhlR DcbrA vs WT | -1.40561 | 8.65E-07 |
| NA | NA | PGD1654730 | NA | DrhlR DcbrA vs WT | -1.1486 | 7.32E-05 |
| PA14_24180 | hypothetical_unclassified | PGD1654737 | NA | DrhlR DcbrA vs WT | 1.436122 | 1.93E-25 |
| PA14_24190 | amino_acid_biosynthesis_me | PGD1654739 | NA | DrhlR DcbrA vs WT | 1.028618 | 9.23E-05 |
| PA14_24500 | cell_wall_LPS | PGD1654791 | pelC | DrhlR DcbrA vs WT | 1.060042 | 8.15E-04 |
| PA14_24500 | fatty_acid_and_phospholipid | PGD1654791 | pelC | DrhlR DcbrA vs WT | 1.060042 | 8.15E-04 |
| PA14_24530 | cell_wall_LPS | PGD1654795 | pelE | DrhlR DcbrA vs WT | 1.097761 | 0.00136 |
| PA14_24860 | energy_metabolism_CAP | PGD1654853 | snr1 | DrhlR DcbrA vs WT | -1.00275 | 6.85E-07 |
| PA14_26020 | secreted_factors | PGD1655043 | NA | DrhlR DcbrA vs WT | -1.17808 | 7.58E-06 |
| PA14_26190 | hypothetical_unclassified | PGD1655067 | NA | DrhlR DcbrA vs WT | -1.5293 | 8.59E-11 |
| PA14_26330 | transcriptional_regulators | PGD1655089 | NA | DrhlR DcbrA vs WT | 1.53626 | 7.25E-12 |
| PA14_26340 | membrane_proteins | PGD1655091 | NA | DrhlR DcbrA vs WT | 3.221339 | 5.91E-07 |
| PA14_26600 | transcriptional_regulators | PGD1655131 | sbrI | DrhlR DcbrA vs WT | 1.139639 | 1.32E-15 |
| PA14_26610 | transcriptional_regulators | PGD1655133 | sbrR | DrhlR DcbrA vs WT | 1.075765 | 4.31E-07 |
| PA14_26650 | putative_enzymes | PGD1655139 | NA | DrhlR DcbrA vs WT | -1.01156 | 0.015393 |
| PA14_26670 | fatty_acid_and_phospholipid | PGD1655141 | NA | DrhlR DcbrA vs WT | -1.32967 | 2.14E-08 |
| PA14_26700 | fatty_acid_and_phospholipid | PGD1655145 | NA | DrhlR DcbrA vs WT | -1.91148 | 5.21E-12 |
| PA14_26700 | putative_enzymes | PGD1655145 | NA | DrhlR DcbrA vs WT | -1.91148 | 5.21E-12 |
| PA14_26720 | fatty_acid_and_phospholipid | PGD1655147 | NA | DrhlR DcbrA vs WT | -1.79846 | 5.44E-13 |
| PA14_26720 | putative_enzymes | PGD1655147 | NA | DrhlR DcbrA vs WT | -1.79846 | 5.44E-13 |
| PA14_26730 | putative_enzymes | PGD1655149 | NA | DrhlR DcbrA vs WT | -2.12817 | 4.51E-08 |
| PA14_26750 | hypothetical_unclassified | PGD1655151 | NA | DrhlR DcbrA vs WT | -1.6876 | 7.55E-14 |
| PA14_26780 | hypothetical_unclassified | PGD1655157 | NA | DrhlR DcbrA vs WT | 1.591236 | 6.52E-12 |
| PA14_26980 | hypothetical_unclassified | PGD1655185 | NA | DrhlR DcbrA vs WT | 1.555934 | 6.75E-15 |
| PA14_27210 | translation_posttranslational | PGD1655221 | efp | DrhlR DcbrA vs WT | -1.09697 | 3.19E-08 |
| PA14_27220 | adaptation_and_protection | PGD1655223 | ohr | DrhlR DcbrA vs WT | -1.70562 | 2.55E-15 |
| PA14_27230 | transcriptional_regulators | PGD1655225 | NA | DrhlR DcbrA vs WT | -1.09613 | 4.58E-06 |
| PA14_27270 | membrane_proteins | PGD1655229 | NA | DrhlR DcbrA vs WT | -1.51378 | 1.14E-08 |
| NA | NA | PGD1655290 | NA | DrhlR DcbrA vs WT | -1.02326 | 0.002428 |
| PA14_27680 | hypothetical_unclassified | PGD1655301 | NA | DrhlR DcbrA vs WT | 1.155046 | 7.91E-08 |
| PA14_27870 | hypothetical_unclassified | PGD1655331 | NA | DrhlR DcbrA vs WT | 1.229476 | 2.59E-11 |
| PA14_28050 | adaptation_and_protection | PGD1655365 | NA | DrhlR DcbrA vs WT | -1.53462 | 8.83E-14 |
| PA14_28050 | chemotaxis | PGD1655365 | NA | DrhlR DcbrA vs WT | -1.53462 | 8.83E-14 |
| PA14_28100 | hypothetical_unclassified | PGD1655375 | NA | DrhlR DcbrA vs WT | 1.11788 | 0.008927 |
| PA14_28110 | hypothetical_unclassified | PGD1655377 | NA | DrhlR DcbrA vs WT | 1.679291 | 0.001143 |
| PA14_28170 | transport_of_small_molecule | PGD1655387 | NA | DrhlR DcbrA vs WT | 1.382976 | 2.56E-18 |
| PA14_28230 | hypothetical_unclassified | PGD1655399 | NA | DrhlR DcbrA vs WT | -1.18272 | 1.21E-05 |
| PA14_28250 | secreted_factors | PGD1655403 | NA | DrhlR DcbrA vs WT | -4.26316 | 3.46E-46 |
| PA14_28260 | hypothetical_unclassified | PGD1655405 | NA | DrhlR DcbrA vs WT | -1.58978 | 3.11E-10 |
| PA14_28280 | central_intermediary_metab | PGD1655407 | NA | DrhlR DcbrA vs WT | -1.26794 | 4.13E-11 |
| PA14_28350 | hypothetical_unclassified | PGD1655421 | NA | DrhlR DcbrA vs WT | 1.116414 | 1.20E-10 |
| PA14_28360 | hypothetical_unclassified | PGD1655423 | NA | DrhlR DcbrA vs WT | -4.97724 | 2.75E-37 |
| PA14_28370 | membrane_proteins | PGD1655425 | NA | DrhlR DcbrA vs WT | -2.47182 | 1.40E-10 |
| PA14_28380 | hypothetical_unclassified | PGD1655427 | NA | DrhlR DcbrA vs WT | -1.10756 | 4.97E-05 |

| | | | | | | |
|---|---|---|---|---|---|---|
| PA14_28410 | hypothetical_unclassified | PGD1655433 | NA | DrhlR DcbrA vs WT | -2.91652 | 5.01E-08 |
| PA14_28490 | membrane_proteins | PGD1655447 | NA | DrhlR DcbrA vs WT | 1.030026 | 1.03E-05 |
| PA14_28520 | hypothetical_unclassified | PGD1655453 | NA | DrhlR DcbrA vs WT | 1.334383 | 6.76E-08 |
| PA14_28530 | membrane_proteins | PGD1655455 | NA | DrhlR DcbrA vs WT | 1.436507 | 1.44E-24 |
| PA14_28630 | putative_enzymes | PGD1655473 | NA | DrhlR DcbrA vs WT | 1.074896 | 1.00E-09 |
| PA14_29090 | transport_of_small_molecule | PGD1655555 | NA | DrhlR DcbrA vs WT | -1.02443 | 0.001473 |
| PA14_29120 | hypothetical_unclassified | PGD1655561 | NA | DrhlR DcbrA vs WT | 1.28538 | 2.59E-14 |
| PA14_29300 | transcriptional_regulators | PGD1655593 | NA | DrhlR DcbrA vs WT | 1.362642 | 6.41E-08 |
| PA14_29420 | putative_enzymes | PGD1655612 | NA | DrhlR DcbrA vs WT | -1.08242 | 4.60E-07 |
| PA14_29470 | hypothetical_unclassified | PGD1655618 | NA | DrhlR DcbrA vs WT | 1.80099 | 8.40E-24 |
| PA14_29620 | transcriptional_regulators | PGD1655646 | NA | DrhlR DcbrA vs WT | 1.297213 | 3.07E-06 |
| PA14_29650 | hypothetical_unclassified | PGD1655650 | NA | DrhlR DcbrA vs WT | 1.200713 | 0.027656 |
| PA14_30240 | translation_posttranslational | PGD1655738 | infA | DrhlR DcbrA vs WT | -1.30717 | 8.23E-06 |
| PA14_30490 | energy_metabolism_CAP | PGD1655782 | NA | DrhlR DcbrA vs WT | -1.05052 | 0.018674 |
| PA14_30560 | translation_posttranslational | PGD1655792 | NA | DrhlR DcbrA vs WT | -2.13498 | 1.72E-18 |
| PA14_30570 | transport_of_small_molecule | PGD1655794 | NA | DrhlR DcbrA vs WT | -1.84609 | 1.64E-24 |
| PA14_30620 | transcriptional_regulators | PGD1655802 | NA | DrhlR DcbrA vs WT | -2.90648 | 9.56E-74 |
| PA14_30630 | biosynthesis_of_cofactors | PGD1655804 | pqsH | DrhlR DcbrA vs WT | -1.05223 | 1.68E-07 |
| PA14_30850 | relative_phage_transposon | PGD1655844 | NA | DrhlR DcbrA vs WT | 1.095025 | 0.008004 |
| PA14_30970 | transcriptional_regulators | PGD1655866 | NA | DrhlR DcbrA vs WT | 1.387671 | 0.001509 |
| PA14_31280 | relative_phage_transposon | PGD1655920 | NA | DrhlR DcbrA vs WT | -1.3386 | 3.16E-09 |
| PA14_31290 | adaptation_and_protection | PGD1655922 | pa1L | DrhlR DcbrA vs WT | -6.53 | 1.26E-39 |
| PA14_31290 | cell_wall_LPS | PGD1655922 | pa1L | DrhlR DcbrA vs WT | -6.53 | 1.26E-39 |
| PA14_31290 | motility_and_attachment | PGD1655922 | pa1L | DrhlR DcbrA vs WT | -6.53 | 1.26E-39 |
| PA14_31350 | energy_metabolism_CAP | PGD1655932 | NA | DrhlR DcbrA vs WT | -3.21343 | 9.52E-74 |
| PA14_31360 | hypothetical_unclassified | PGD1655934 | NA | DrhlR DcbrA vs WT | -3.27777 | 8.24E-58 |
| PA14_31370 | putative_enzymes | PGD1655936 | NA | DrhlR DcbrA vs WT | -2.5464 | 7.19E-55 |
| PA14_31530 | fatty_acid_and_phospholipid | PGD1655962 | NA | DrhlR DcbrA vs WT | -1.17051 | 7.65E-06 |
| PA14_31530 | putative_enzymes | PGD1655962 | NA | DrhlR DcbrA vs WT | -1.17051 | 7.65E-06 |
| PA14_31540 | energy_metabolism_CAP | PGD1655964 | NA | DrhlR DcbrA vs WT | -1.10994 | 6.79E-07 |
| PA14_31950 | two_component_regulators | PGD1656022 | NA | DrhlR DcbrA vs WT | 1.01976 | 0.010612 |
| PA14_31960 | two_component_regulators | PGD1656024 | NA | DrhlR DcbrA vs WT | 1.296521 | 4.71E-06 |
| PA14_32060 | transcriptional_regulators | PGD1656032 | xylS | DrhlR DcbrA vs WT | -1.32777 | 3.79E-05 |
| PA14_32140 | energy_metabolism_CAP | PGD1656042 | antC | DrhlR DcbrA vs WT | -4.59601 | 1.47E-13 |
| PA14_32150 | biosynthesis_of_cofactors | PGD1656044 | antB | DrhlR DcbrA vs WT | -4.37007 | 1.19E-06 |
| PA14_32160 | carbon_compound_catabolis | PGD1656046 | antA | DrhlR DcbrA vs WT | -3.03642 | 7.73E-18 |
| PA14_32190 | transcriptional_regulators | PGD1656048 | NA | DrhlR DcbrA vs WT | -1.93971 | 1.94E-14 |
| PA14_32220 | carbon_compound_catabolis | PGD1656052 | catB | DrhlR DcbrA vs WT | -2.85803 | 2.27E-09 |
| PA14_32230 | carbon_compound_catabolis | PGD1656054 | catC | DrhlR DcbrA vs WT | -3.12038 | 8.07E-14 |
| PA14_32240 | carbon_compound_catabolis | PGD1656056 | catA | DrhlR DcbrA vs WT | -2.67271 | 3.06E-14 |
| PA14_32250 | hypothetical_unclassified | PGD1656058 | NA | DrhlR DcbrA vs WT | -1.53594 | 0.006905 |
| PA14_32380 | antibiotic_resistance_and_su | PGD1656080 | oprN | DrhlR DcbrA vs WT | 1.126671 | 0.016851 |
| PA14_32380 | membrane_proteins | PGD1656080 | oprN | DrhlR DcbrA vs WT | 1.126671 | 0.016851 |
| PA14_32380 | transport_of_small_molecule | PGD1656080 | oprN | DrhlR DcbrA vs WT | 1.126671 | 0.016851 |
| PA14_32440 | hypothetical_unclassified | PGD1656090 | NA | DrhlR DcbrA vs WT | 1.059671 | 1.34E-04 |
| PA14_32490 | hypothetical_unclassified | PGD1656100 | NA | DrhlR DcbrA vs WT | 1.368558 | 3.81E-08 |
| PA14_32830 | hypothetical_unclassified | PGD1656152 | NA | DrhlR DcbrA vs WT | 1.262936 | 1.48E-06 |
| PA14_32860 | hypothetical_unclassified | PGD1656158 | NA | DrhlR DcbrA vs WT | 1.133388 | 5.54E-05 |
| PA14_33050 | hypothetical_unclassified | PGD1656184 | NA | DrhlR DcbrA vs WT | 2.032153 | 1.10E-25 |
| PA14_33060 | hypothetical_unclassified | PGD1656186 | NA | DrhlR DcbrA vs WT | 2.257722 | 1.59E-15 |

| | | | | | | |
|---|---|---|---|---|---|---|
| PA14_33120 | hypothetical_unclassified | PGD1656194 | NA | DrhlR DcbrA vs WT | 1.046197 | 2.99E-07 |
| PA14_33160 | hypothetical_unclassified | PGD1656200 | NA | DrhlR DcbrA vs WT | 1.435297 | 6.24E-11 |
| PA14_33250 | hypothetical_unclassified | PGD1656214 | NA | DrhlR DcbrA vs WT | 1.202373 | 2.33E-04 |
| PA14_33420 | putative_enzymes | PGD1656244 | NA | DrhlR DcbrA vs WT | 1.821454 | 2.35E-05 |
| PA14_33430 | DNA_replication_recombinat | PGD1656246 | NA | DrhlR DcbrA vs WT | 1.603266 | 4.72E-07 |
| PA14_33450 | carbon_compound_catabolis | PGD1656250 | treA | DrhlR DcbrA vs WT | 1.211228 | 6.30E-08 |
| PA14_33460 | membrane_proteins | PGD1656252 | NA | DrhlR DcbrA vs WT | 1.007055 | 1.12E-04 |
| PA14_33630 | adaptation_and_protection | PGD1656280 | pvdJ | DrhlR DcbrA vs WT | 1.013115 | 5.23E-05 |
| PA14_33870 | hypothetical_unclassified | PGD1656318 | NA | DrhlR DcbrA vs WT | -1.50317 | 1.43E-18 |
| PA14_33910 | transport_of_small_molecule | PGD1656326 | NA | DrhlR DcbrA vs WT | 2.210531 | 3.83E-10 |
| PA14_34390 | transport_of_small_molecule | PGD1656402 | NA | DrhlR DcbrA vs WT | -1.32756 | 0.016219 |
| PA14_34420 | transport_of_small_molecule | PGD1656406 | NA | DrhlR DcbrA vs WT | -1.24998 | 2.90E-05 |
| PA14_34460 | adaptation_and_protection | PGD1656412 | NA | DrhlR DcbrA vs WT | -1.57885 | 1.04E-16 |
| PA14_34500 | transport_of_small_molecule | PGD1656416 | NA | DrhlR DcbrA vs WT | -1.37688 | 3.15E-08 |
| PA14_34510 | transport_of_small_molecule | PGD1656418 | NA | DrhlR DcbrA vs WT | -1.24079 | 7.49E-10 |
| PA14_34670 | putative_enzymes | PGD1656438 | NA | DrhlR DcbrA vs WT | -1.382 | 6.67E-09 |
| PA14_34730 | transcriptional_regulators | PGD1656450 | NA | DrhlR DcbrA vs WT | -1.37347 | 0.015008 |
| PA14_34740 | hypothetical_unclassified | PGD1656452 | NA | DrhlR DcbrA vs WT | -1.31246 | 0.027837 |
| PA14_34870 | carbon_compound_catabolis | PGD1656474 | chiC | DrhlR DcbrA vs WT | -6.81691 | 4.71E-47 |
| PA14_34880 | transcriptional_regulators | PGD1656476 | NA | DrhlR DcbrA vs WT | -1.85043 | 2.59E-07 |
| PA14_34900 | energy_metabolism_CAP | PGD1656478 | NA | DrhlR DcbrA vs WT | -2.12548 | 9.90E-15 |
| PA14_34900 | putative_enzymes | PGD1656478 | NA | DrhlR DcbrA vs WT | -2.12548 | 9.90E-15 |
| PA14_34920 | energy_metabolism_CAP | PGD1656480 | NA | DrhlR DcbrA vs WT | -4.09097 | 1.35E-05 |
| PA14_35160 | hypothetical_unclassified | PGD1656520 | NA | DrhlR DcbrA vs WT | -2.33044 | 6.87E-15 |
| PA14_35340 | carbon_compound_catabolis | PGD1656546 | NA | DrhlR DcbrA vs WT | 1.746648 | 5.36E-07 |
| PA14_35370 | transcriptional_regulators | PGD1656550 | ptxS | DrhlR DcbrA vs WT | 1.076777 | 3.55E-09 |
| PA14_35380 | transcriptional_regulators | PGD1656552 | ptxR | DrhlR DcbrA vs WT | 1.015012 | 4.00E-05 |
| PA14_35550 | hypothetical_unclassified | PGD1656578 | pslO | DrhlR DcbrA vs WT | 1.772305 | 2.81E-11 |
| PA14_35570 | DNA_replication_recombinat | PGD1656580 | pslN | DrhlR DcbrA vs WT | 1.564209 | 3.64E-10 |
| PA14_36200 | transport_of_small_molecule | PGD1656688 | NA | DrhlR DcbrA vs WT | -1.56159 | 1.89E-05 |
| PA14_36220 | membrane_proteins | PGD1656690 | NA | DrhlR DcbrA vs WT | -1.90339 | 0.006609 |
| PA14_36220 | transport_of_small_molecule | PGD1656690 | NA | DrhlR DcbrA vs WT | -1.90339 | 0.006609 |
| PA14_36230 | membrane_proteins | PGD1656692 | NA | DrhlR DcbrA vs WT | -1.71 | 0.001434 |
| PA14_36230 | transport_of_small_molecule | PGD1656692 | NA | DrhlR DcbrA vs WT | -1.71 | 0.001434 |
| PA14_36260 | two_component_regulators | PGD1656696 | NA | DrhlR DcbrA vs WT | 1.077709 | 1.22E-04 |
| PA14_36310 | central_intermediary_metab | PGD1656706 | hcnC | DrhlR DcbrA vs WT | -2.21918 | 2.61E-28 |
| PA14_36320 | central_intermediary_metab | PGD1656708 | hcnB | DrhlR DcbrA vs WT | -3.30964 | 8.07E-35 |
| PA14_36330 | central_intermediary_metab | PGD1656710 | hcnA | DrhlR DcbrA vs WT | -3.02931 | 2.96E-38 |
| PA14_36370 | amino_acid_biosynthesis_me | PGD1656718 | NA | DrhlR DcbrA vs WT | 1.62672 | 1.17E-07 |
| PA14_36375 | hypothetical_unclassified | PGD1656720 | NA | DrhlR DcbrA vs WT | 1.529992 | 3.45E-06 |
| PA14_36390 | translation_posttranslational | PGD1656722 | NA | DrhlR DcbrA vs WT | 1.295105 | 2.45E-05 |
| PA14_36400 | hypothetical_unclassified | PGD1656724 | NA | DrhlR DcbrA vs WT | 1.337589 | 0.001187 |
| PA14_36410 | hypothetical_unclassified | PGD1656726 | NA | DrhlR DcbrA vs WT | 1.192463 | 4.66E-07 |
| PA14_36420 | two_component_regulators | PGD1656728 | NA | DrhlR DcbrA vs WT | 1.101609 | 9.86E-12 |
| PA14_36450 | hypothetical_unclassified | PGD1656730 | NA | DrhlR DcbrA vs WT | 1.449643 | 5.37E-07 |
| PA14_36460 | hypothetical_unclassified | PGD1656732 | NA | DrhlR DcbrA vs WT | 1.35804 | 1.05E-13 |
| PA14_36480 | hypothetical_unclassified | PGD1656736 | NA | DrhlR DcbrA vs WT | 1.125973 | 0.003532 |
| PA14_36490 | hypothetical_unclassified | PGD1656738 | NA | DrhlR DcbrA vs WT | 1.362663 | 0.002907 |
| PA14_36500 | carbon_compound_catabolis | PGD1656740 | NA | DrhlR DcbrA vs WT | 1.317482 | 2.67E-07 |
| PA14_36520 | hypothetical_unclassified | PGD1656742 | NA | DrhlR DcbrA vs WT | 1.049766 | 1.88E-07 |

| | | | | | | |
|---|---|---|---|---|---|---|
| PA14_36540 | putative_enzymes | PGD1656746 | NA | DrhlR DcbrA vs WT | 1.591322 | 3.03E-06 |
| PA14_36550 | hypothetical_unclassified | PGD1656748 | NA | DrhlR DcbrA vs WT | 2.01555 | 2.37E-27 |
| PA14_36570 | carbon_compound_catabolis | PGD1656752 | glgA | DrhlR DcbrA vs WT | 1.104799 | 1.48E-08 |
| PA14_36570 | energy_metabolism_CAP | PGD1656752 | glgA | DrhlR DcbrA vs WT | 1.104799 | 1.48E-08 |
| PA14_36580 | putative_enzymes | PGD1656754 | NA | DrhlR DcbrA vs WT | 1.361424 | 3.13E-07 |
| PA14_36590 | carbon_compound_catabolis | PGD1656756 | NA | DrhlR DcbrA vs WT | 1.676103 | 7.74E-06 |
| PA14_36605 | putative_enzymes | PGD1656758 | NA | DrhlR DcbrA vs WT | 1.422901 | 1.85E-04 |
| PA14_36650 | hypothetical_unclassified | PGD1656764 | NA | DrhlR DcbrA vs WT | 1.538804 | 4.60E-07 |
| PA14_36660 | amino_acid_biosynthesis_me | PGD1656766 | NA | DrhlR DcbrA vs WT | 1.206851 | 8.63E-07 |
| PA14_36670 | hypothetical_unclassified | PGD1656768 | NA | DrhlR DcbrA vs WT | 2.394668 | 3.42E-12 |
| PA14_36680 | putative_enzymes | PGD1656770 | NA | DrhlR DcbrA vs WT | 1.491408 | 1.37E-04 |
| PA14_36690 | fatty_acid_and_phospholipid | PGD1656772 | NA | DrhlR DcbrA vs WT | 2.164408 | 3.73E-11 |
| PA14_36690 | putative_enzymes | PGD1656772 | NA | DrhlR DcbrA vs WT | 2.164408 | 3.73E-11 |
| PA14_36700 | membrane_proteins | PGD1656774 | NA | DrhlR DcbrA vs WT | 1.489745 | 5.19E-05 |
| PA14_36710 | energy_metabolism_CAP | PGD1656776 | glgB | DrhlR DcbrA vs WT | 1.527754 | 9.35E-04 |
| PA14_36730 | carbon_compound_catabolis | PGD1656778 | NA | DrhlR DcbrA vs WT | 1.263973 | 3.65E-06 |
| PA14_36730 | putative_enzymes | PGD1656778 | NA | DrhlR DcbrA vs WT | 1.263973 | 3.65E-06 |
| PA14_36740 | putative_enzymes | PGD1656780 | NA | DrhlR DcbrA vs WT | 1.00382 | 3.04E-07 |
| PA14_36770 | hypothetical_unclassified | PGD1656784 | NA | DrhlR DcbrA vs WT | 1.039791 | 0.009967 |
| PA14_36780 | transport_of_small_molecule | PGD1656786 | NA | DrhlR DcbrA vs WT | 1.424639 | 2.72E-05 |
| PA14_36790 | hypothetical_unclassified | PGD1656788 | NA | DrhlR DcbrA vs WT | 1.517918 | 2.96E-09 |
| PA14_36820 | hypothetical_unclassified | PGD1656792 | NA | DrhlR DcbrA vs WT | 1.208088 | 0.004864 |
| PA14_36870 | central_intermediary_metab | PGD1656802 | NA | DrhlR DcbrA vs WT | 1.038254 | 2.39E-05 |
| PA14_36880 | transcriptional_regulators | PGD1656804 | NA | DrhlR DcbrA vs WT | 1.931953 | 1.17E-06 |
| PA14_36900 | hypothetical_unclassified | PGD1656808 | NA | DrhlR DcbrA vs WT | 1.145458 | 0.001383 |
| PA14_36910 | DNA_replication_recombinat | PGD1656810 | ligD | DrhlR DcbrA vs WT | 1.417775 | 5.44E-13 |
| PA14_36920 | two_component_regulators | PGD1656812 | NA | DrhlR DcbrA vs WT | 1.35109 | 8.48E-10 |
| PA14_36930 | hypothetical_unclassified | PGD1656814 | NA | DrhlR DcbrA vs WT | 1.490492 | 1.94E-07 |
| PA14_36960 | membrane_proteins | PGD1656818 | NA | DrhlR DcbrA vs WT | 1.191732 | 4.09E-06 |
| PA14_36960 | transport_of_small_molecule | PGD1656818 | NA | DrhlR DcbrA vs WT | 1.191732 | 4.09E-06 |
| PA14_36980 | membrane_proteins | PGD1656820 | NA | DrhlR DcbrA vs WT | 1.009345 | 4.54E-05 |
| PA14_37060 | motility_and_attachment | PGD1656832 | cupA1 | DrhlR DcbrA vs WT | 1.653941 | 0.001428 |
| PA14_37070 | central_intermediary_metab | PGD1656834 | NA | DrhlR DcbrA vs WT | 1.555423 | 4.26E-10 |
| PA14_37070 | transcriptional_regulators | PGD1656834 | NA | DrhlR DcbrA vs WT | 1.555423 | 4.26E-10 |
| PA14_37190 | DNA_replication_recombinat | PGD1656852 | ada | DrhlR DcbrA vs WT | 1.36719 | 2.62E-10 |
| PA14_37190 | transcriptional_regulators | PGD1656852 | ada | DrhlR DcbrA vs WT | 1.36719 | 2.62E-10 |
| PA14_37210 | hypothetical_unclassified | PGD1656856 | NA | DrhlR DcbrA vs WT | 1.917194 | 2.58E-15 |
| PA14_37350 | hypothetical_unclassified | PGD1656874 | NA | DrhlR DcbrA vs WT | 1.69499 | 2.62E-07 |
| PA14_37360 | central_intermediary_metab | PGD1656876 | NA | DrhlR DcbrA vs WT | -2.93752 | 1.04E-20 |
| PA14_37370 | fatty_acid_and_phospholipid | PGD1656878 | NA | DrhlR DcbrA vs WT | -2.87462 | 2.77E-06 |
| PA14_37380 | transport_of_small_molecule | PGD1656880 | NA | DrhlR DcbrA vs WT | -1.90066 | 3.14E-17 |
| PA14_37610 | amino_acid_biosynthesis_me | PGD1656914 | NA | DrhlR DcbrA vs WT | -1.13068 | 2.32E-04 |
| PA14_37730 | transport_of_small_molecule | PGD1656932 | NA | DrhlR DcbrA vs WT | -1.01379 | 1.75E-07 |
| PA14_37745 | putative_enzymes | PGD1656934 | NA | DrhlR DcbrA vs WT | -5.7491 | 1.02E-22 |
| PA14_37760 | membrane_proteins | PGD1656936 | NA | DrhlR DcbrA vs WT | -4.13073 | 5.71E-61 |
| PA14_37760 | transport_of_small_molecule | PGD1656936 | NA | DrhlR DcbrA vs WT | -4.13073 | 5.71E-61 |
| PA14_37770 | carbon_compound_catabolis | PGD1656938 | NA | DrhlR DcbrA vs WT | -2.79215 | 8.92E-21 |
| PA14_37780 | hypothetical_unclassified | PGD1656940 | NA | DrhlR DcbrA vs WT | -3.64916 | 2.21E-34 |
| PA14_37790 | adaptation_and_protection | PGD1656942 | pcoA | DrhlR DcbrA vs WT | -1.052 | 3.84E-08 |
| PA14_38050 | hypothetical_unclassified | PGD1656982 | NA | DrhlR DcbrA vs WT | 1.841806 | 2.03E-10 |

| | | | | | | |
|---|---|---|---|---|---|---|
| PA14_38110 | transport_of_small_molecule | PGD1656990 | NA | DrhlR DcbrA vs WT | -1.05388 | 3.44E-07 |
| PA14_38210 | central_intermediary_metab | PGD1657006 | NA | DrhlR DcbrA vs WT | 1.503489 | 9.47E-04 |
| PA14_38220 | transport_of_small_molecule | PGD1657008 | NA | DrhlR DcbrA vs WT | 1.151357 | 0.002253 |
| PA14_38260 | hypothetical_unclassified | PGD1657012 | NA | DrhlR DcbrA vs WT | -1.68174 | 1.08E-08 |
| PA14_38270 | hypothetical_unclassified | PGD1657014 | NA | DrhlR DcbrA vs WT | -1.26118 | 9.87E-05 |
| PA14_38360 | cell_wall_LPS | PGD1657030 | NA | DrhlR DcbrA vs WT | 1.300004 | 1.77E-10 |
| PA14_38360 | putative_enzymes | PGD1657030 | NA | DrhlR DcbrA vs WT | 1.300004 | 1.77E-10 |
| PA14_38370 | hypothetical_unclassified | PGD1657032 | NA | DrhlR DcbrA vs WT | 1.83097 | 2.57E-12 |
| PA14_38590 | carbon_compound_catabolis | PGD1657068 | bdhA | DrhlR DcbrA vs WT | -1.22375 | 2.02E-09 |
| PA14_38660 | fatty_acid_and_phospholipid | PGD1657076 | NA | DrhlR DcbrA vs WT | 1.209293 | 9.72E-17 |
| PA14_38770 | amino_acid_biosynthesis_me | PGD1657094 | NA | DrhlR DcbrA vs WT | 1.167354 | 1.10E-06 |
| PA14_38950 | membrane_proteins | PGD1657122 | NA | DrhlR DcbrA vs WT | -1.22017 | 0.023655 |
| PA14_39010 | biosynthesis_of_cofactors | PGD1657130 | pqqF | DrhlR DcbrA vs WT | 1.107141 | 4.30E-04 |
| PA14_39220 | hypothetical_unclassified | PGD1657164 | NA | DrhlR DcbrA vs WT | 5.239621 | 8.01E-08 |
| PA14_39240 | hypothetical_unclassified | PGD1657168 | fapC | DrhlR DcbrA vs WT | 3.719278 | 1.75E-19 |
| PA14_39420 | hypothetical_unclassified | PGD1657192 | NA | DrhlR DcbrA vs WT | -1.51488 | 0.00162 |
| PA14_39440 | hypothetical_unclassified | PGD1657194 | NA | DrhlR DcbrA vs WT | -1.38423 | 1.10E-11 |
| PA14_39460 | hypothetical_unclassified | PGD1657196 | NA | DrhlR DcbrA vs WT | -3.34838 | 2.22E-27 |
| PA14_39470 | hypothetical_unclassified | PGD1657198 | NA | DrhlR DcbrA vs WT | -1.42598 | 2.31E-05 |
| PA14_39500 | hypothetical_unclassified | PGD1657202 | NA | DrhlR DcbrA vs WT | 2.053397 | 4.84E-11 |
| PA14_39590 | amino_acid_biosynthesis_me | PGD1657216 | metE | DrhlR DcbrA vs WT | -1.78336 | 3.84E-05 |
| PA14_39700 | hypothetical_unclassified | PGD1657234 | NA | DrhlR DcbrA vs WT | -1.15315 | 0.027656 |
| PA14_39780 | transport_of_small_molecule | PGD1657246 | NA | DrhlR DcbrA vs WT | -4.92274 | 3.71E-77 |
| PA14_39790 | hypothetical_unclassified | PGD1657248 | NA | DrhlR DcbrA vs WT | -1.86583 | 9.77E-04 |
| PA14_39880 | secreted_factors | PGD1657264 | phzG2 | DrhlR DcbrA vs WT | -5.64037 | 2.24E-23 |
| PA14_39945 | secreted_factors | PGD1657272 | phzC2 | DrhlR DcbrA vs WT | -5.99253 | 5.09E-23 |
| PA14_39960 | secreted_factors | PGD1657274 | phzB2 | DrhlR DcbrA vs WT | -7.96826 | 2.70E-37 |
| PA14_39970 | secreted_factors | PGD1657276 | phzA2 | DrhlR DcbrA vs WT | -8.56046 | 2.54E-27 |
| PA14_39980 | transcriptional_regulators | PGD1657278 | qscR | DrhlR DcbrA vs WT | -1.91468 | 1.40E-14 |
| PA14_39990 | fatty_acid_and_phospholipid | PGD1657280 | NA | DrhlR DcbrA vs WT | -2.85909 | 3.03E-28 |
| PA14_40010 | hypothetical_unclassified | PGD1657282 | NA | DrhlR DcbrA vs WT | -1.91916 | 8.60E-21 |
| PA14_40020 | hypothetical_unclassified | PGD1657284 | NA | DrhlR DcbrA vs WT | -2.67641 | 1.30E-36 |
| PA14_40030 | putative_enzymes | PGD1657286 | NA | DrhlR DcbrA vs WT | -2.33462 | 4.82E-25 |
| PA14_40040 | central_intermediary_metab | PGD1657288 | NA | DrhlR DcbrA vs WT | -2.16553 | 7.40E-28 |
| PA14_40040 | putative_enzymes | PGD1657288 | NA | DrhlR DcbrA vs WT | -2.16553 | 7.40E-28 |
| PA14_40050 | hypothetical_unclassified | PGD1657290 | NA | DrhlR DcbrA vs WT | -1.92041 | 6.49E-21 |
| PA14_40060 | fatty_acid_and_phospholipid | PGD1657292 | NA | DrhlR DcbrA vs WT | -1.89099 | 1.37E-08 |
| PA14_40060 | membrane_proteins | PGD1657292 | NA | DrhlR DcbrA vs WT | -1.89099 | 1.37E-08 |
| PA14_40170 | membrane_proteins | PGD1657310 | NA | DrhlR DcbrA vs WT | -1.03581 | 2.30E-05 |
| PA14_40170 | protein_secretion_export | PGD1657310 | NA | DrhlR DcbrA vs WT | -1.03581 | 2.30E-05 |
| PA14_40170 | transport_of_small_molecule | PGD1657310 | NA | DrhlR DcbrA vs WT | -1.03581 | 2.30E-05 |
| PA14_40230 | protein_secretion_export | PGD1657320 | NA | DrhlR DcbrA vs WT | -2.28469 | 1.22E-26 |
| PA14_40240 | protein_secretion_export | PGD1657322 | NA | DrhlR DcbrA vs WT | -2.2801 | 1.14E-20 |
| PA14_40250 | membrane_proteins | PGD1657324 | NA | DrhlR DcbrA vs WT | -2.17536 | 3.57E-29 |
| PA14_40260 | hypothetical_unclassified | PGD1657326 | NA | DrhlR DcbrA vs WT | -2.32571 | 4.04E-24 |
| PA14_40270 | membrane_proteins | PGD1657328 | NA | DrhlR DcbrA vs WT | -2.72494 | 4.55E-11 |
| PA14_40270 | transport_of_small_molecule | PGD1657328 | NA | DrhlR DcbrA vs WT | -2.72494 | 4.55E-11 |
| PA14_40290 | secreted_factors | PGD1657332 | lasA | DrhlR DcbrA vs WT | -2.24261 | 4.42E-31 |
| PA14_40290 | translation_posttranslational | PGD1657332 | lasA | DrhlR DcbrA vs WT | -2.24261 | 4.42E-31 |
| PA14_40310 | fatty_acid_and_phospholipid | PGD1657336 | NA | DrhlR DcbrA vs WT | -5.79418 | 7.72E-47 |

| | | | | | | |
|---|---|---|---|---|---|---|
| PA14_40770 | central_intermediary_metab | PGD1657406 | cysI | DrhlR DcbrA vs WT | -1.14675 | 8.81E-10 |
| PA14_40780 | hypothetical_unclassified | PGD1657408 | NA | DrhlR DcbrA vs WT | -1.02965 | 3.89E-07 |
| PA14_41500 | putative_enzymes | PGD1657526 | NA | DrhlR DcbrA vs WT | -1.30941 | 1.57E-23 |
| PA14_41563 | biosynthesis_of_cofactors | PGD1657538 | cobA | DrhlR DcbrA vs WT | 1.397982 | 9.97E-06 |
| PA14_41900 | central_intermediary_metab | PGD1657590 | panE | DrhlR DcbrA vs WT | 1.137229 | 7.34E-17 |
| PA14_42390 | transcriptional_regulators | PGD1657668 | exsA | DrhlR DcbrA vs WT | 1.393463 | 1.11E-06 |
| PA14_42450 | protein_secretion_export | PGD1657678 | popB | DrhlR DcbrA vs WT | 1.294567 | 0.004279 |
| PA14_42470 | protein_secretion_export | PGD1657682 | pcrV | DrhlR DcbrA vs WT | 1.010396 | 0.027465 |
| PA14_42880 | protein_secretion_export | PGD1657754 | stk1 | DrhlR DcbrA vs WT | -1.25999 | 3.62E-10 |
| PA14_42880 | translation_posttranslational | PGD1657754 | stk1 | DrhlR DcbrA vs WT | -1.25999 | 3.62E-10 |
| PA14_42890 | protein_secretion_export | PGD1657756 | stp1 | DrhlR DcbrA vs WT | -1.01863 | 1.07E-04 |
| PA14_42890 | translation_posttranslational | PGD1657756 | stp1 | DrhlR DcbrA vs WT | -1.01863 | 1.07E-04 |
| PA14_42910 | membrane_proteins | PGD1657760 | dotU2 | DrhlR DcbrA vs WT | -1.04667 | 1.17E-07 |
| PA14_42940 | fatty_acid_and_phospholipid | PGD1657764 | lip2.1 | DrhlR DcbrA vs WT | -1.13579 | 0.001302 |
| PA14_42950 | hypothetical_unclassified | PGD1657766 | fha2 | DrhlR DcbrA vs WT | -1.29669 | 6.56E-09 |
| PA14_42960 | protein_secretion_export | PGD1657768 | lip2.2 | DrhlR DcbrA vs WT | -1.43998 | 1.36E-06 |
| PA14_42970 | transcriptional_regulators | PGD1657770 | sfa2 | DrhlR DcbrA vs WT | -1.3328 | 2.22E-15 |
| PA14_42980 | chaperones_heat_shock | PGD1657772 | clpV2 | DrhlR DcbrA vs WT | -1.3206 | 3.58E-16 |
| PA14_42990 | hypothetical_unclassified | PGD1657774 | hsiH2 | DrhlR DcbrA vs WT | -1.5498 | 3.00E-15 |
| PA14_43000 | protein_secretion_export | PGD1657776 | hsiG2 | DrhlR DcbrA vs WT | -1.59316 | 1.21E-17 |
| PA14_43020 | protein_secretion_export | PGD1657778 | hsiF2 | DrhlR DcbrA vs WT | -1.42422 | 1.96E-10 |
| PA14_43030 | protein_secretion_export | PGD1657780 | hsiC2 | DrhlR DcbrA vs WT | -1.75685 | 1.80E-20 |
| PA14_43040 | protein_secretion_export | PGD1657782 | hsiB2 | DrhlR DcbrA vs WT | -1.41541 | 5.10E-14 |
| PA14_43050 | hypothetical_unclassified | PGD1657784 | hsiA2 | DrhlR DcbrA vs WT | -1.49357 | 1.93E-15 |
| PA14_43070 | secreted_factors | PGD1657786 | hcp2 | DrhlR DcbrA vs WT | -1.66165 | 2.93E-08 |
| PA14_43090 | hypothetical_unclassified | PGD1657790 | tap | DrhlR DcbrA vs WT | -1.88326 | 3.89E-07 |
| PA14_43760 | hypothetical_unclassified | PGD1657898 | NA | DrhlR DcbrA vs WT | 1.286563 | 1.34E-07 |
| PA14_44430 | hypothetical_unclassified | PGD1658010 | NA | DrhlR DcbrA vs WT | -1.07679 | 1.52E-16 |
| PA14_44440 | membrane_proteins | PGD1658012 | NA | DrhlR DcbrA vs WT | -1.14596 | 1.69E-16 |
| PA14_44440 | transport_of_small_molecule | PGD1658012 | NA | DrhlR DcbrA vs WT | -1.14596 | 1.69E-16 |
| PA14_44520 | membrane_proteins | PGD1658028 | NA | DrhlR DcbrA vs WT | 1.509191 | 2.39E-05 |
| PA14_44520 | transport_of_small_molecule | PGD1658028 | NA | DrhlR DcbrA vs WT | 1.509191 | 2.39E-05 |
| PA14_44530 | membrane_proteins | PGD1658030 | NA | DrhlR DcbrA vs WT | 1.212136 | 7.03E-06 |
| PA14_44890 | secreted_factors | PGD1658086 | hcpA | DrhlR DcbrA vs WT | -1.94923 | 1.34E-05 |
| PA14_44950 | membrane_proteins | PGD1658096 | NA | DrhlR DcbrA vs WT | 1.639199 | 1.31E-17 |
| PA14_44950 | transport_of_small_molecule | PGD1658096 | NA | DrhlR DcbrA vs WT | 1.639199 | 1.31E-17 |
| PA14_44960 | putative_enzymes | PGD1658098 | NA | DrhlR DcbrA vs WT | 1.692913 | 1.31E-08 |
| PA14_45020 | putative_enzymes | PGD1658110 | NA | DrhlR DcbrA vs WT | 1.338754 | 1.48E-04 |
| PA14_45100 | transcriptional_regulators | PGD1658122 | NA | DrhlR DcbrA vs WT | 1.152409 | 3.28E-04 |
| PA14_45100 | transport_of_small_molecule | PGD1658122 | NA | DrhlR DcbrA vs WT | 1.152409 | 3.28E-04 |
| PA14_45310 | energy_metabolism_CAP | PGD1658152 | ccmF | DrhlR DcbrA vs WT | -1.09359 | 3.36E-11 |
| PA14_45330 | energy_metabolism_CAP | PGD1658154 | ccmE | DrhlR DcbrA vs WT | -1.00487 | 1.33E-11 |
| PA14_45340 | transport_of_small_molecule | PGD1658156 | NA | DrhlR DcbrA vs WT | -1.05549 | 1.21E-04 |
| PA14_45350 | transport_of_small_molecule | PGD1658158 | ccmC | DrhlR DcbrA vs WT | -1.20093 | 1.92E-19 |
| PA14_45370 | membrane_proteins | PGD1658160 | ccmB | DrhlR DcbrA vs WT | -1.2744 | 2.82E-15 |
| PA14_45370 | transport_of_small_molecule | PGD1658160 | ccmB | DrhlR DcbrA vs WT | -1.2744 | 2.82E-15 |
| PA14_45680 | adaptation_and_protection | PGD1658204 | flhA | DrhlR DcbrA vs WT | 1.010516 | 1.83E-14 |
| PA14_45680 | chemotaxis | PGD1658204 | flhA | DrhlR DcbrA vs WT | 1.010516 | 1.83E-14 |
| PA14_45680 | motility_and_attachment | PGD1658204 | flhA | DrhlR DcbrA vs WT | 1.010516 | 1.83E-14 |
| PA14_46230 | carbon_compound_catabolis | PGD1658288 | aphA | DrhlR DcbrA vs WT | 1.020685 | 8.45E-04 |

| | | | | | | |
|---|---|---|---|---|---|---|
| PA14_46240 | membrane_proteins | PGD1658290 | NA | DrhlR DcbrA vs WT | 1.17555 | 2.15E-11 |
| PA14_46250 | putative_enzymes | PGD1658292 | NA | DrhlR DcbrA vs WT | 1.057607 | 8.01E-08 |
| PA14_46850 | transcriptional_regulators | PGD1658400 | NA | DrhlR DcbrA vs WT | 2.140958 | 0.021676 |
| PA14_46860 | amino_acid_biosynthesis_me | PGD1658402 | NA | DrhlR DcbrA vs WT | 1.635438 | 0.039162 |
| PA14_47040 | membrane_proteins | PGD1658432 | NA | DrhlR DcbrA vs WT | -1.04491 | 1.37E-08 |
| PA14_47130 | hypothetical_unclassified | PGD1658448 | NA | DrhlR DcbrA vs WT | 1.17344 | 1.30E-07 |
| PA14_47390 | two_component_regulators | PGD1658492 | NA | DrhlR DcbrA vs WT | 1.045339 | 0.005825 |
| PA14_47400 | transcriptional_regulators | PGD1658494 | NA | DrhlR DcbrA vs WT | 1.010237 | 0.006579 |
| PA14_47920 | transport_of_small_molecule | PGD1658574 | NA | DrhlR DcbrA vs WT | 1.704983 | 0.003958 |
| PA14_48115 | protein_secretion_export | PGD1658602 | aprD | DrhlR DcbrA vs WT | 1.212623 | 6.09E-06 |
| PA14_48160 | two_component_regulators | PGD1658608 | NA | DrhlR DcbrA vs WT | 1.185184 | 1.11E-11 |
| PA14_48170 | motility_and_attachment | PGD1658610 | srpP | DrhlR DcbrA vs WT | 1.536078 | 5.76E-20 |
| PA14_48170 | translation_posttranslational | PGD1658610 | srpP | DrhlR DcbrA vs WT | 1.536078 | 5.76E-20 |
| PA14_48530 | putative_enzymes | PGD1658662 | NA | DrhlR DcbrA vs WT | -1.92399 | 1.41E-05 |
| PA14_48590 | hypothetical_unclassified | PGD1658672 | NA | DrhlR DcbrA vs WT | -2.06283 | 7.59E-04 |
| PA14_49050 | membrane_proteins | PGD1658756 | NA | DrhlR DcbrA vs WT | 1.099912 | 6.40E-07 |
| PA14_49130 | transport_of_small_molecule | PGD1658770 | dctA | DrhlR DcbrA vs WT | 2.020752 | 3.80E-08 |
| PA14_49300 | putative_enzymes | PGD1658798 | NA | DrhlR DcbrA vs WT | 1.11996 | 9.99E-07 |
| PA14_49410 | adaptation_and_protection | PGD1658818 | NA | DrhlR DcbrA vs WT | -1.21375 | 2.52E-06 |
| PA14_49410 | transcriptional_regulators | PGD1658818 | NA | DrhlR DcbrA vs WT | -1.21375 | 2.52E-06 |
| PA14_49560 | secreted_factors | PGD1658840 | toxA | DrhlR DcbrA vs WT | 2.12997 | 3.26E-10 |
| PA14_49690 | putative_enzymes | PGD1658862 | NA | DrhlR DcbrA vs WT | 3.414093 | 3.14E-13 |
| PA14_49720 | hypothetical_unclassified | PGD1658868 | NA | DrhlR DcbrA vs WT | 1.001002 | 0.022243 |
| PA14_49750 | antibiotic_resistance_and_su | PGD1658874 | NA | DrhlR DcbrA vs WT | -5.05654 | 3.29E-49 |
| PA14_49750 | membrane_proteins | PGD1658874 | NA | DrhlR DcbrA vs WT | -5.05654 | 3.29E-49 |
| PA14_49750 | transport_of_small_molecule | PGD1658874 | NA | DrhlR DcbrA vs WT | -5.05654 | 3.29E-49 |
| PA14_49760 | adaptation_and_protection | PGD1658876 | rhlC | DrhlR DcbrA vs WT | -4.48061 | 1.03E-13 |
| PA14_49800 | putative_enzymes | PGD1658882 | NA | DrhlR DcbrA vs WT | -1.01645 | 9.55E-06 |
| PA14_49850 | hypothetical_unclassified | PGD1658890 | NA | DrhlR DcbrA vs WT | -1.02255 | 2.00E-09 |
| PA14_49940 | membrane_proteins | PGD1658908 | NA | DrhlR DcbrA vs WT | 1.446316 | 1.98E-23 |
| PA14_49960 | hypothetical_unclassified | PGD1658910 | NA | DrhlR DcbrA vs WT | 1.364959 | 1.52E-16 |
| PA14_50020 | hypothetical_unclassified | PGD1658920 | NA | DrhlR DcbrA vs WT | 1.524878 | 4.96E-09 |
| PA14_50540 | membrane_proteins | PGD1658998 | livM | DrhlR DcbrA vs WT | -1.08495 | 1.22E-05 |
| PA14_50540 | transport_of_small_molecule | PGD1658998 | livM | DrhlR DcbrA vs WT | -1.08495 | 1.22E-05 |
| PA14_51080 | putative_enzymes | PGD1659096 | NA | DrhlR DcbrA vs WT | 1.144866 | 0.026278 |
| PA14_51350 | adaptation_and_protection | PGD1659144 | phnB | DrhlR DcbrA vs WT | 3.021765 | 1.89E-111 |
| PA14_51350 | amino_acid_biosynthesis_me | PGD1659144 | phnB | DrhlR DcbrA vs WT | 3.021765 | 1.89E-111 |
| PA14_51360 | adaptation_and_protection | PGD1659146 | phnA | DrhlR DcbrA vs WT | 3.739357 | 1.07E-243 |
| PA14_51380 | biosynthesis_of_cofactors | PGD1659148 | pqsE | DrhlR DcbrA vs WT | 4.299212 | 7.42E-157 |
| PA14_51390 | hypothetical_unclassified | PGD1659150 | pqsD | DrhlR DcbrA vs WT | 3.877689 | 1.03E-287 |
| PA14_51410 | biosynthesis_of_cofactors | PGD1659152 | pqsC | DrhlR DcbrA vs WT | 3.393778 | 1.99E-124 |
| PA14_51420 | biosynthesis_of_cofactors | PGD1659154 | pqsB | DrhlR DcbrA vs WT | 3.580773 | 5.78E-197 |
| PA14_51430 | biosynthesis_of_cofactors | PGD1659156 | pqsA | DrhlR DcbrA vs WT | 3.058249 | 6.93E-195 |
| PA14_51490 | hypothetical_unclassified | PGD1659168 | NA | DrhlR DcbrA vs WT | 1.731684 | 1.40E-14 |
| PA14_51630 | relative_phage_transposon | PGD1659196 | NA | DrhlR DcbrA vs WT | 1.159103 | 0.001456 |
| PA14_51730 | membrane_proteins | PGD1659214 | tolA | DrhlR DcbrA vs WT | 1.029581 | 8.45E-16 |
| PA14_51730 | transport_of_small_molecule | PGD1659214 | tolA | DrhlR DcbrA vs WT | 1.029581 | 8.45E-16 |
| PA14_51940 | hypothetical_unclassified | PGD1659252 | NA | DrhlR DcbrA vs WT | 2.486029 | 1.52E-16 |
| PA14_52130 | hypothetical_unclassified | PGD1659284 | NA | DrhlR DcbrA vs WT | -2.02206 | 1.31E-31 |
| PA14_52250 | transport_of_small_molecule | PGD1659302 | NA | DrhlR DcbrA vs WT | 1.49575 | 8.27E-08 |

| | | | | | | |
|---|---|---|---|---|---|---|
| PA14_52250 | two_component_regulators | PGD1659302 | NA | DrhlR DcbrA vs WT | 1.49575 | 8.27E-08 |
| PA14_52800 | central_intermediary_metab | PGD1659394 | acsA | DrhlR DcbrA vs WT | 1.613608 | 7.11E-18 |
| PA14_52840 | transport_of_small_molecule | PGD1659400 | NA | DrhlR DcbrA vs WT | 1.364455 | 9.90E-06 |
| PA14_52870 | putative_enzymes | PGD1659404 | NA | DrhlR DcbrA vs WT | 1.502502 | 0.001724 |
| PA14_52880 | putative_enzymes | PGD1659406 | NA | DrhlR DcbrA vs WT | 5.054211 | 4.27E-04 |
| PA14_52900 | putative_enzymes | PGD1659410 | NA | DrhlR DcbrA vs WT | 1.191296 | 6.38E-04 |
| PA14_52910 | hypothetical_unclassified | PGD1659412 | NA | DrhlR DcbrA vs WT | 1.150702 | 0.020572 |
| PA14_53050 | transport_of_small_molecule | PGD1659436 | aroP2 | DrhlR DcbrA vs WT | -1.73263 | 2.77E-11 |
| PA14_53250 | secreted_factors | PGD1659466 | cpbD | DrhlR DcbrA vs WT | -3.11583 | 1.29E-74 |
| PA14_53370 | secreted_factors | PGD1659484 | plcR | DrhlR DcbrA vs WT | 1.193368 | 8.28E-06 |
| PA14_53380 | putative_enzymes | PGD1659486 | NA | DrhlR DcbrA vs WT | 1.605004 | 0.001819 |
| PA14_53540 | putative_enzymes | PGD1659514 | NA | DrhlR DcbrA vs WT | 1.252428 | 0.001307 |
| PA14_53550 | transcriptional_regulators | PGD1659516 | NA | DrhlR DcbrA vs WT | 1.260509 | 3.41E-07 |
| PA14_53740 | hypothetical_unclassified | PGD1659552 | NA | DrhlR DcbrA vs WT | 1.017645 | 0.009653 |
| PA14_53750 | hypothetical_unclassified | PGD1659554 | NA | DrhlR DcbrA vs WT | 1.41975 | 0.001029 |
| PA14_54150 | membrane_proteins | PGD1659618 | putP | DrhlR DcbrA vs WT | -1.25103 | 1.72E-12 |
| PA14_54150 | transport_of_small_molecule | PGD1659618 | putP | DrhlR DcbrA vs WT | -1.25103 | 1.72E-12 |
| PA14_54240 | hypothetical_unclassified | PGD1659632 | NA | DrhlR DcbrA vs WT | 1.150491 | 1.92E-08 |
| PA14_54520 | membrane_proteins | PGD1659676 | NA | DrhlR DcbrA vs WT | 1.683337 | 1.02E-07 |
| PA14_54720 | hypothetical_unclassified | PGD1659710 | NA | DrhlR DcbrA vs WT | 1.902245 | 1.29E-07 |
| PA14_54740 | hypothetical_unclassified | PGD1659714 | NA | DrhlR DcbrA vs WT | 1.487694 | 6.40E-08 |
| PA14_54750 | hypothetical_unclassified | PGD1659716 | NA | DrhlR DcbrA vs WT | 1.322899 | 8.25E-08 |
| PA14_55040 | transport_of_small_molecule | PGD1659768 | NA | DrhlR DcbrA vs WT | 1.050838 | 0.041235 |
| PA14_55080 | hypothetical_unclassified | PGD1659776 | NA | DrhlR DcbrA vs WT | -1.59611 | 2.50E-26 |
| PA14_55540 | transcriptional_regulators | PGD1659854 | NA | DrhlR DcbrA vs WT | 1.168039 | 0.001669 |
| PA14_55730 | transcriptional_regulators | PGD1659894 | NA | DrhlR DcbrA vs WT | 1.300206 | 0.003716 |
| PA14_55750 | adaptation_and_protection | PGD1659898 | NA | DrhlR DcbrA vs WT | 2.44118 | 1.91E-31 |
| PA14_55750 | chemotaxis | PGD1659898 | NA | DrhlR DcbrA vs WT | 2.44118 | 1.91E-31 |
| PA14_55780 | two_component_regulators | PGD1659904 | NA | DrhlR DcbrA vs WT | -1.36993 | 1.82E-17 |
| PA14_55790 | protein_secretion_export | PGD1659906 | NA | DrhlR DcbrA vs WT | -1.4634 | 6.99E-21 |
| PA14_55800 | protein_secretion_export | PGD1659908 | NA | DrhlR DcbrA vs WT | -1.28551 | 2.36E-04 |
| PA14_55850 | protein_secretion_export | PGD1659916 | NA | DrhlR DcbrA vs WT | -1.23396 | 1.21E-06 |
| PA14_55860 | membrane_proteins | PGD1659918 | NA | DrhlR DcbrA vs WT | -1.18203 | 5.06E-08 |
| PA14_55860 | motility_and_attachment | PGD1659918 | NA | DrhlR DcbrA vs WT | -1.18203 | 5.06E-08 |
| PA14_55860 | protein_secretion_export | PGD1659918 | NA | DrhlR DcbrA vs WT | -1.18203 | 5.06E-08 |
| PA14_55890 | protein_secretion_export | PGD1659922 | NA | DrhlR DcbrA vs WT | -1.07937 | 3.92E-07 |
| PA14_55900 | motility_and_attachment | PGD1659924 | NA | DrhlR DcbrA vs WT | -1.05819 | 1.92E-06 |
| PA14_55900 | protein_secretion_export | PGD1659924 | NA | DrhlR DcbrA vs WT | -1.05819 | 1.92E-06 |
| PA14_55920 | motility_and_attachment | PGD1659926 | NA | DrhlR DcbrA vs WT | -1.24143 | 1.54E-11 |
| PA14_55920 | protein_secretion_export | PGD1659926 | NA | DrhlR DcbrA vs WT | -1.24143 | 1.54E-11 |
| PA14_55940 | protein_secretion_export | PGD1659930 | NA | DrhlR DcbrA vs WT | -1.3727 | 4.63E-05 |
| PA14_55960 | chemotaxis | PGD1659932 | pctC | DrhlR DcbrA vs WT | -1.10033 | 1.58E-09 |
| PA14_56480 | putative_enzymes | PGD1660006 | NA | DrhlR DcbrA vs WT | 1.474303 | 1.33E-09 |
| PA14_56480 | translation_posttranslational | PGD1660006 | NA | DrhlR DcbrA vs WT | 1.474303 | 1.33E-09 |
| PA14_56510 | hypothetical_unclassified | PGD1660008 | NA | DrhlR DcbrA vs WT | 1.144412 | 9.30E-13 |
| PA14_56520 | hypothetical_unclassified | PGD1660010 | NA | DrhlR DcbrA vs WT | 1.592788 | 3.80E-09 |
| PA14_56570 | fatty_acid_and_phospholipid | PGD1660020 | NA | DrhlR DcbrA vs WT | -2.18386 | 1.96E-05 |
| PA14_56590 | adaptation_and_protection | PGD1660022 | NA | DrhlR DcbrA vs WT | -1.70027 | 1.11E-09 |
| PA14_56620 | transcriptional_regulators | PGD1660026 | NA | DrhlR DcbrA vs WT | -1.76525 | 3.36E-06 |
| PA14_56750 | hypothetical_unclassified | PGD1660046 | NA | DrhlR DcbrA vs WT | 1.283099 | 0.00472 |

| | | | | | | |
|---|---|---|---|---|---|---|
| PA14_56770 | membrane_proteins | PGD1660048 | NA | DrhlR DcbrA vs WT | 1.608278 | 0.011434 |
| PA14_56770 | transport_of_small_molecule | PGD1660048 | NA | DrhlR DcbrA vs WT | 1.608278 | 0.011434 |
| PA14_56830 | membrane_proteins | PGD1660058 | icmP | DrhlR DcbrA vs WT | -1.08678 | 4.09E-06 |
| PA14_56990 | hypothetical_unclassified | PGD1660088 | NA | DrhlR DcbrA vs WT | -3.10235 | 2.02E-43 |
| PA14_57030 | membrane_proteins | PGD1660094 | fxsA | DrhlR DcbrA vs WT | 1.776108 | 2.20E-12 |
| PA14_57140 | transcriptional_regulators | PGD1660112 | NA | DrhlR DcbrA vs WT | 1.151311 | 2.75E-08 |
| PA14_57140 | two_component_regulators | PGD1660112 | NA | DrhlR DcbrA vs WT | 1.151311 | 2.75E-08 |
| PA14_57590 | translation_posttranslational | PGD1660186 | rplM | DrhlR DcbrA vs WT | -1.08032 | 2.80E-09 |
| PA14_57710 | amino_acid_biosynthesis_me | PGD1660203 | cysN | DrhlR DcbrA vs WT | -1.72563 | 6.64E-21 |
| PA14_57710 | central_intermediary_metab | PGD1660203 | cysN | DrhlR DcbrA vs WT | -1.72563 | 6.64E-21 |
| PA14_57720 | amino_acid_biosynthesis_me | PGD1660205 | cysD | DrhlR DcbrA vs WT | -1.82524 | 2.71E-12 |
| PA14_57720 | central_intermediary_metab | PGD1660205 | cysD | DrhlR DcbrA vs WT | -1.82524 | 2.71E-12 |
| PA14_57980 | transport_of_small_molecule | PGD1660251 | NA | DrhlR DcbrA vs WT | -1.04222 | 8.63E-08 |
| PA14_58040 | hypothetical_unclassified | PGD1660261 | NA | DrhlR DcbrA vs WT | 1.260259 | 0.017766 |
| PA14_58360 | transport_of_small_molecule | PGD1660313 | dppA2 | DrhlR DcbrA vs WT | -1.13516 | 7.32E-07 |
| PA14_58410 | membrane_proteins | PGD1660321 | opdP | DrhlR DcbrA vs WT | -2.95717 | 5.76E-46 |
| PA14_58410 | transport_of_small_molecule | PGD1660321 | opdP | DrhlR DcbrA vs WT | -2.95717 | 5.76E-46 |
| PA14_58420 | transport_of_small_molecule | PGD1660323 | dppA4 | DrhlR DcbrA vs WT | -1.40604 | 7.92E-11 |
| PA14_58440 | membrane_proteins | PGD1660325 | dppB | DrhlR DcbrA vs WT | -1.61814 | 4.16E-15 |
| PA14_58440 | transport_of_small_molecule | PGD1660325 | dppB | DrhlR DcbrA vs WT | -1.61814 | 4.16E-15 |
| PA14_58450 | membrane_proteins | PGD1660327 | dppC | DrhlR DcbrA vs WT | -1.51462 | 4.21E-06 |
| PA14_58450 | transport_of_small_molecule | PGD1660327 | dppC | DrhlR DcbrA vs WT | -1.51462 | 4.21E-06 |
| PA14_58470 | transport_of_small_molecule | PGD1660329 | dppD | DrhlR DcbrA vs WT | -1.39369 | 1.07E-06 |
| PA14_58490 | transport_of_small_molecule | PGD1660331 | dppF | DrhlR DcbrA vs WT | -1.63078 | 1.67E-13 |
| PA14_58500 | membrane_proteins | PGD1660333 | NA | DrhlR DcbrA vs WT | 1.609747 | 5.24E-06 |
| PA14_58510 | transcriptional_regulators | PGD1660335 | NA | DrhlR DcbrA vs WT | -1.00373 | 0.008774 |
| PA14_58580 | membrane_proteins | PGD1660349 | NA | DrhlR DcbrA vs WT | 1.291848 | 2.50E-04 |
| PA14_58630 | amino_acid_biosynthesis_me | PGD1660357 | NA | DrhlR DcbrA vs WT | -1.94601 | 6.75E-43 |
| PA14_59360 | motility_and_attachment | PGD1660489 | pilM2 | DrhlR DcbrA vs WT | -1.83816 | 6.14E-08 |
| PA14_59890 | hypothetical_unclassified | PGD1660586 | NA | DrhlR DcbrA vs WT | 1.560862 | 0.037917 |
| PA14_60140 | DNA_replication_recombinat | PGD1660636 | NA | DrhlR DcbrA vs WT | 1.128056 | 0.027095 |
| NA | NA | PGD1660639 | NA | DrhlR DcbrA vs WT | -1.46967 | 8.50E-08 |
| PA14_60190 | chaperones_heat_shock | PGD1660644 | clpB | DrhlR DcbrA vs WT | 1.000758 | 9.19E-05 |
| PA14_60280 | motility_and_attachment | PGD1660660 | fimU | DrhlR DcbrA vs WT | 1.07299 | 3.61E-09 |
| PA14_60400 | central_intermediary_metab | PGD1660682 | rpsT | DrhlR DcbrA vs WT | -1.29455 | 2.51E-09 |
| PA14_60400 | translation_posttranslational | PGD1660682 | rpsT | DrhlR DcbrA vs WT | -1.29455 | 2.51E-09 |
| PA14_60460 | translation_posttranslational | PGD1660692 | rplU | DrhlR DcbrA vs WT | -1.11101 | 2.17E-11 |
| NA | NA | PGD1660721 | NA | DrhlR DcbrA vs WT | -1.13721 | 2.18E-09 |
| PA14_60630 | membrane_proteins | PGD1660722 | NA | DrhlR DcbrA vs WT | 1.265821 | 2.02E-05 |
| PA14_60750 | carbon_compound_catabolis | PGD1660738 | pra | DrhlR DcbrA vs WT | -2.74316 | 1.04E-16 |
| PA14_61000 | hypothetical_unclassified | PGD1660778 | NA | DrhlR DcbrA vs WT | 1.105009 | 0.003161 |
| PA14_61080 | transport_of_small_molecule | PGD1660790 | NA | DrhlR DcbrA vs WT | 1.855636 | 2.19E-10 |
| PA14_61370 | hypothetical_unclassified | PGD1660838 | NA | DrhlR DcbrA vs WT | 1.479719 | 1.41E-04 |
| PA14_61500 | hypothetical_unclassified | PGD1660860 | NA | DrhlR DcbrA vs WT | -1.27134 | 1.36E-09 |
| PA14_61510 | hypothetical_unclassified | PGD1660862 | NA | DrhlR DcbrA vs WT | -1.09613 | 1.66E-05 |
| PA14_61520 | hypothetical_unclassified | PGD1660864 | NA | DrhlR DcbrA vs WT | -1.22069 | 2.81E-06 |
| PA14_61530 | motility_and_attachment | PGD1660866 | NA | DrhlR DcbrA vs WT | -1.04516 | 2.89E-05 |
| PA14_61540 | motility_and_attachment | PGD1660868 | NA | DrhlR DcbrA vs WT | -1.0358 | 9.37E-06 |
| NA | NA | PGD1660905 | NA | DrhlR DcbrA vs WT | -1.16674 | 5.08E-07 |
| PA14_61780 | adaptation_and_protection | PGD1660908 | NA | DrhlR DcbrA vs WT | -1.26062 | 2.57E-11 |

| | | | | | | |
|---|---|---|---|---|---|---|
| PA14_61780 | translation_posttranslational | PGD1660908 | NA | DrhlR DcbrA vs WT | -1.26062 | 2.57E-11 |
| NA | NA | PGD1660915 | NA | DrhlR DcbrA vs WT | -1.3304 | 0.007592 |
| PA14_62350 | transport_of_small_molecule | PGD1661000 | NA | DrhlR DcbrA vs WT | 1.279976 | 5.10E-12 |
| PA14_62520 | hypothetical_unclassified | PGD1661030 | NA | DrhlR DcbrA vs WT | -1.06813 | 1.07E-05 |
| PA14_62530 | carbon_compound_catabolis | PGD1661032 | cbrA | DrhlR DcbrA vs WT | -1.59688 | 0.009648 |
| PA14_62530 | two_component_regulators | PGD1661032 | cbrA | DrhlR DcbrA vs WT | -1.59688 | 0.009648 |
| PA14_62960 | adaptation_and_protection | PGD1661106 | dnaJ | DrhlR DcbrA vs WT | 1.139278 | 4.26E-06 |
| PA14_62960 | chaperones_heat_shock | PGD1661106 | dnaJ | DrhlR DcbrA vs WT | 1.139278 | 4.26E-06 |
| PA14_62960 | DNA_replication_recombinat | PGD1661106 | dnaJ | DrhlR DcbrA vs WT | 1.139278 | 4.26E-06 |
| PA14_62970 | adaptation_and_protection | PGD1661108 | dnaK | DrhlR DcbrA vs WT | 1.136237 | 1.23E-09 |
| PA14_62970 | chaperones_heat_shock | PGD1661108 | dnaK | DrhlR DcbrA vs WT | 1.136237 | 1.23E-09 |
| PA14_62970 | DNA_replication_recombinat | PGD1661108 | dnaK | DrhlR DcbrA vs WT | 1.136237 | 1.23E-09 |
| PA14_62990 | chaperones_heat_shock | PGD1661110 | grpE | DrhlR DcbrA vs WT | 1.111762 | 1.45E-09 |
| PA14_62990 | DNA_replication_recombinat | PGD1661110 | grpE | DrhlR DcbrA vs WT | 1.111762 | 1.45E-09 |
| PA14_63270 | putative_enzymes | PGD1661158 | fabG | DrhlR DcbrA vs WT | 1.208146 | 1.68E-15 |
| PA14_63800 | transport_of_small_molecule | PGD1661242 | mgtA | DrhlR DcbrA vs WT | 1.126759 | 7.10E-04 |
| PA14_64050 | transcriptional_regulators | PGD1661278 | NA | DrhlR DcbrA vs WT | 1.435089 | 5.13E-13 |
| PA14_64050 | two_component_regulators | PGD1661278 | NA | DrhlR DcbrA vs WT | 1.435089 | 5.13E-13 |
| PA14_64270 | transport_of_small_molecule | PGD1661310 | NA | DrhlR DcbrA vs WT | 1.432121 | 0.007566 |
| PA14_64370 | central_intermediary_metab | PGD1661328 | ureB | DrhlR DcbrA vs WT | -1.22051 | 1.25E-04 |
| PA14_64430 | hypothetical_unclassified | PGD1661338 | NA | DrhlR DcbrA vs WT | -1.08186 | 2.03E-05 |
| PA14_64490 | hypothetical_unclassified | PGD1661350 | NA | DrhlR DcbrA vs WT | 1.06682 | 3.96E-06 |
| PA14_64520 | central_intermediary_metab | PGD1661356 | NA | DrhlR DcbrA vs WT | 1.241594 | 2.98E-14 |
| PA14_64560 | hypothetical_unclassified | PGD1661364 | NA | DrhlR DcbrA vs WT | -1.44878 | 0.018846 |
| PA14_64860 | transport_of_small_molecule | PGD1661414 | NA | DrhlR DcbrA vs WT | -1.00231 | 0.002067 |
| PA14_64930 | nucleotide_biosynthesis_and | PGD1661428 | NA | DrhlR DcbrA vs WT | -3.33301 | 1.25E-59 |
| PA14_64940 | biosynthesis_of_cofactors | PGD1661430 | NA | DrhlR DcbrA vs WT | -3.40132 | 3.25E-31 |
| PA14_64950 | central_intermediary_metab | PGD1661432 | pncA | DrhlR DcbrA vs WT | 1.00675 | 1.96E-05 |
| PA14_65170 | translation_posttranslational | PGD1661464 | rpsR | DrhlR DcbrA vs WT | -1.15152 | 5.04E-08 |
| PA14_65690 | nucleotide_biosynthesis_and | PGD1661538 | NA | DrhlR DcbrA vs WT | 1.666945 | 1.15E-16 |
| PA14_65690 | putative_enzymes | PGD1661538 | NA | DrhlR DcbrA vs WT | 1.666945 | 1.15E-16 |
| PA14_65820 | fatty_acid_and_phospholipid | PGD1661558 | NA | DrhlR DcbrA vs WT | -1.55569 | 0.00383 |
| PA14_65820 | putative_enzymes | PGD1661558 | NA | DrhlR DcbrA vs WT | -1.55569 | 0.00383 |
| PA14_65940 | energy_metabolism_CAP | PGD1661574 | NA | DrhlR DcbrA vs WT | -1.24927 | 2.01E-06 |
| PA14_65940 | putative_enzymes | PGD1661574 | NA | DrhlR DcbrA vs WT | -1.24927 | 2.01E-06 |
| PA14_66420 | membrane_proteins | PGD1661650 | NA | DrhlR DcbrA vs WT | -2.21281 | 5.38E-18 |
| PA14_66460 | adaptation_and_protection | PGD1661656 | NA | DrhlR DcbrA vs WT | -1.34298 | 2.08E-07 |
| PA14_66510 | membrane_proteins | PGD1661662 | NA | DrhlR DcbrA vs WT | -1.85254 | 1.11E-06 |
| PA14_66510 | transport_of_small_molecule | PGD1661662 | NA | DrhlR DcbrA vs WT | -1.85254 | 1.11E-06 |
| PA14_66540 | hypothetical_unclassified | PGD1661668 | NA | DrhlR DcbrA vs WT | 1.029137 | 1.06E-04 |
| PA14_66710 | translation_posttranslational | PGD1661700 | rpmE | DrhlR DcbrA vs WT | -1.28839 | 1.58E-10 |
| PA14_66770 | chaperones_heat_shock | PGD1661708 | hslV | DrhlR DcbrA vs WT | 1.384307 | 2.77E-14 |
| PA14_66840 | central_intermediary_metab | PGD1661718 | phaC2 | DrhlR DcbrA vs WT | -1.68518 | 2.21E-18 |
| PA14_66850 | transcriptional_regulators | PGD1661720 | NA | DrhlR DcbrA vs WT | -1.96726 | 2.80E-24 |
| PA14_67150 | amino_acid_biosynthesis_me | PGD1661770 | NA | DrhlR DcbrA vs WT | 1.069478 | 4.95E-05 |
| PA14_67180 | hypothetical_unclassified | PGD1661774 | tli5b4 | DrhlR DcbrA vs WT | -1.0071 | 7.03E-06 |
| PA14_67190 | hypothetical_unclassified | PGD1661776 | tli5b3 | DrhlR DcbrA vs WT | -1.22438 | 3.32E-07 |
| PA14_67210 | hypothetical_unclassified | PGD1661780 | tli5b1 | DrhlR DcbrA vs WT | -1.20544 | 1.71E-07 |
| PA14_67220 | hypothetical_unclassified | PGD1661782 | tle5b | DrhlR DcbrA vs WT | -1.13926 | 4.44E-10 |
| PA14_67230 | hypothetical_unclassified | PGD1661784 | vgrG5 | DrhlR DcbrA vs WT | -1.03528 | 1.96E-13 |

| | | | | | | |
|---|---|---|---|---|---|---|
| PA14_67240 | amino_acid_biosynthesis_me | PGD1661786 | hutG | DrhlR DcbrA vs WT | -1.01686 | 1.11E-07 |
| PA14_67310 | transport_of_small_molecule | PGD1661798 | NA | DrhlR DcbrA vs WT | -1.74839 | 2.78E-13 |
| PA14_67320 | amino_acid_biosynthesis_me | PGD1661800 | hutH | DrhlR DcbrA vs WT | -1.7067 | 1.38E-09 |
| PA14_67320 | central_intermediary_metab | PGD1661800 | hutH | DrhlR DcbrA vs WT | -1.7067 | 1.38E-09 |
| PA14_67340 | transport_of_small_molecule | PGD1661802 | NA | DrhlR DcbrA vs WT | -1.65289 | 1.32E-17 |
| PA14_67350 | amino_acid_biosynthesis_me | PGD1661804 | hutU | DrhlR DcbrA vs WT | -1.57723 | 1.87E-12 |
| PA14_67840 | transport_of_small_molecule | PGD1661878 | NA | DrhlR DcbrA vs WT | 1.083632 | 2.19E-06 |
| PA14_67860 | transport_of_small_molecule | PGD1661882 | NA | DrhlR DcbrA vs WT | 1.178359 | 8.29E-05 |
| PA14_68080 | membrane_proteins | PGD1661918 | NA | DrhlR DcbrA vs WT | -1.56965 | 2.81E-08 |
| PA14_68080 | transport_of_small_molecule | PGD1661918 | NA | DrhlR DcbrA vs WT | -1.56965 | 2.81E-08 |
| PA14_68090 | membrane_proteins | PGD1661920 | NA | DrhlR DcbrA vs WT | -1.62423 | 6.07E-12 |
| PA14_68090 | transport_of_small_molecule | PGD1661920 | NA | DrhlR DcbrA vs WT | -1.62423 | 6.07E-12 |
| PA14_68100 | hypothetical_unclassified | PGD1661922 | NA | DrhlR DcbrA vs WT | -1.02266 | 0.004807 |
| PA14_68170 | cell_wall_LPS | PGD1661934 | rmlB | DrhlR DcbrA vs WT | -2.10292 | 8.59E-78 |
| PA14_68190 | cell_wall_LPS | PGD1661936 | rmlD | DrhlR DcbrA vs WT | -1.61356 | 8.33E-27 |
| PA14_68200 | cell_wall_LPS | PGD1661938 | rmlA | DrhlR DcbrA vs WT | -1.52969 | 9.26E-24 |
| PA14_68210 | cell_wall_LPS | PGD1661940 | rmlC | DrhlR DcbrA vs WT | -1.43228 | 7.27E-27 |
| PA14_68300 | amino_acid_biosynthesis_me | PGD1661952 | arcD | DrhlR DcbrA vs WT | 1.155557 | 1.25E-04 |
| PA14_68300 | membrane_proteins | PGD1661952 | arcD | DrhlR DcbrA vs WT | 1.155557 | 1.25E-04 |
| PA14_68300 | transport_of_small_molecule | PGD1661952 | arcD | DrhlR DcbrA vs WT | 1.155557 | 1.25E-04 |
| PA14_68430 | putative_enzymes | PGD1661972 | NA | DrhlR DcbrA vs WT | -1.94575 | 1.10E-27 |
| PA14_68440 | energy_metabolism_CAP | PGD1661974 | NA | DrhlR DcbrA vs WT | -1.98755 | 5.19E-21 |
| PA14_68440 | putative_enzymes | PGD1661974 | NA | DrhlR DcbrA vs WT | -1.98755 | 5.19E-21 |
| PA14_68450 | membrane_proteins | PGD1661976 | NA | DrhlR DcbrA vs WT | 1.063672 | 4.67E-04 |
| PA14_68810 | putative_enzymes | PGD1662032 | NA | DrhlR DcbrA vs WT | 1.332979 | 3.57E-13 |
| PA14_68930 | membrane_proteins | PGD1662052 | NA | DrhlR DcbrA vs WT | -5.76871 | 1.32E-23 |
| PA14_68930 | transport_of_small_molecule | PGD1662052 | NA | DrhlR DcbrA vs WT | -5.76871 | 1.32E-23 |
| PA14_68940 | hypothetical_unclassified | PGD1662054 | NA | DrhlR DcbrA vs WT | -5.76822 | 1.55E-41 |
| PA14_69550 | hypothetical_unclassified | PGD1662150 | NA | DrhlR DcbrA vs WT | -2.25445 | 0.008789 |
| PA14_69560 | secreted_factors | PGD1662152 | hcpB | DrhlR DcbrA vs WT | -1.79744 | 6.72E-17 |
| PA14_69780 | hypothetical_unclassified | PGD1662190 | NA | DrhlR DcbrA vs WT | -1.29262 | 2.99E-13 |
| PA14_69795 | membrane_proteins | PGD1662192 | amtB | DrhlR DcbrA vs WT | -1.27068 | 1.30E-14 |
| PA14_69795 | transport_of_small_molecule | PGD1662192 | amtB | DrhlR DcbrA vs WT | -1.27068 | 1.30E-14 |
| PA14_69850 | transport_of_small_molecule | PGD1662200 | NA | DrhlR DcbrA vs WT | 1.080009 | 4.33E-18 |
| PA14_70180 | translation_posttranslational | PGD1662248 | rpmG | DrhlR DcbrA vs WT | -1.36478 | 4.98E-10 |
| PA14_70190 | translation_posttranslational | PGD1662250 | rpmB | DrhlR DcbrA vs WT | -1.33163 | 1.25E-11 |
| PA14_70600 | DNA_replication_recombinat | PGD1662314 | NA | DrhlR DcbrA vs WT | -1.13022 | 1.03E-04 |
| PA14_70640 | carbon_compound_catabolis | PGD1662320 | rubA1 | DrhlR DcbrA vs WT | -2.27887 | 1.76E-15 |
| PA14_70650 | putative_enzymes | PGD1662322 | NA | DrhlR DcbrA vs WT | -2.16003 | 4.58E-08 |
| PA14_70670 | carbon_compound_catabolis | PGD1662324 | glcF | DrhlR DcbrA vs WT | -1.93742 | 8.20E-09 |
| PA14_70670 | central_intermediary_metab | PGD1662324 | glcF | DrhlR DcbrA vs WT | -1.93742 | 8.20E-09 |
| PA14_70670 | energy_metabolism_CAP | PGD1662324 | glcF | DrhlR DcbrA vs WT | -1.93742 | 8.20E-09 |
| PA14_70680 | carbon_compound_catabolis | PGD1662326 | glcE | DrhlR DcbrA vs WT | -2.38985 | 1.17E-16 |
| PA14_70680 | central_intermediary_metab | PGD1662326 | glcE | DrhlR DcbrA vs WT | -2.38985 | 1.17E-16 |
| PA14_70690 | carbon_compound_catabolis | PGD1662328 | glcD | DrhlR DcbrA vs WT | -2.12299 | 6.92E-19 |
| PA14_70690 | central_intermediary_metab | PGD1662328 | glcD | DrhlR DcbrA vs WT | -2.12299 | 6.92E-19 |
| PA14_70720 | biosynthesis_of_cofactors | PGD1662332 | ubiC | DrhlR DcbrA vs WT | -1.0951 | 1.86E-08 |
| PA14_70720 | central_intermediary_metab | PGD1662332 | ubiC | DrhlR DcbrA vs WT | -1.0951 | 1.86E-08 |
| PA14_70950 | adaptation_and_protection | PGD1662374 | betB | DrhlR DcbrA vs WT | 1.213299 | 2.34E-05 |
| PA14_70950 | amino_acid_biosynthesis_me | PGD1662374 | betB | DrhlR DcbrA vs WT | 1.213299 | 2.34E-05 |

| | | | | | | |
|---|---|---|---|---|---|---|
| PA14_71100 | membrane_proteins | PGD1662394 | NA | DrhlR DcbrA vs WT | -2.97507 | 8.17E-08 |
| PA14_71240 | amino_acid_biosynthesis_me | PGD1662420 | NA | DrhlR DcbrA vs WT | -1.39021 | 0.0059 |
| PA14_71250 | hypothetical_unclassified | PGD1662422 | NA | DrhlR DcbrA vs WT | -1.6537 | 0.014695 |
| PA14_71280 | amino_acid_biosynthesis_me | PGD1662426 | NA | DrhlR DcbrA vs WT | -1.14677 | 0.002753 |
| PA14_71280 | energy_metabolism_CAP | PGD1662426 | NA | DrhlR DcbrA vs WT | -1.14677 | 0.002753 |
| PA14_71410 | transport_of_small_molecule | PGD1662450 | NA | DrhlR DcbrA vs WT | -2.26831 | 9.81E-04 |
| PA14_71420 | energy_metabolism_CAP | PGD1662452 | NA | DrhlR DcbrA vs WT | -1.32329 | 2.24E-04 |
| PA14_71460 | amino_acid_biosynthesis_me | PGD1662460 | glyA1 | DrhlR DcbrA vs WT | -2.6234 | 2.52E-06 |
| PA14_71470 | amino_acid_biosynthesis_me | PGD1662462 | soxB | DrhlR DcbrA vs WT | -1.4905 | 9.01E-05 |
| PA14_71470 | carbon_compound_catabolis | PGD1662462 | soxB | DrhlR DcbrA vs WT | -1.4905 | 9.01E-05 |
| PA14_71500 | carbon_compound_catabolis | PGD1662466 | soxA | DrhlR DcbrA vs WT | -1.77133 | 1.73E-06 |
| PA14_71510 | amino_acid_biosynthesis_me | PGD1662468 | soxG | DrhlR DcbrA vs WT | -1.70645 | 0.001856 |
| PA14_71510 | carbon_compound_catabolis | PGD1662468 | soxG | DrhlR DcbrA vs WT | -1.70645 | 0.001856 |
| PA14_71530 | nucleotide_biosynthesis_and | PGD1662470 | purU2 | DrhlR DcbrA vs WT | -1.71846 | 0.001008 |
| PA14_71560 | amino_acid_biosynthesis_me | PGD1662472 | fdhA | DrhlR DcbrA vs WT | -2.17385 | 1.14E-07 |
| PA14_71650 | amino_acid_biosynthesis_me | PGD1662488 | aspA | DrhlR DcbrA vs WT | -1.05741 | 2.92E-07 |
| PA14_71720 | central_intermediary_metab | PGD1662500 | NA | DrhlR DcbrA vs WT | -1.16976 | 2.66E-07 |
| PA14_71890 | fatty_acid_and_phospholipid | PGD1662524 | NA | DrhlR DcbrA vs WT | 1.639395 | 4.50E-21 |
| PA14_71890 | putative_enzymes | PGD1662524 | NA | DrhlR DcbrA vs WT | 1.639395 | 4.50E-21 |
| PA14_71960 | cell_wall_LPS | PGD1662536 | wzm | DrhlR DcbrA vs WT | -1.45484 | 1.54E-14 |
| PA14_72090 | hypothetical_unclassified | PGD1662560 | NA | DrhlR DcbrA vs WT | 1.27411 | 3.27E-07 |
| PA14_72280 | transport_of_small_molecule | PGD1662586 | citA | DrhlR DcbrA vs WT | 1.397003 | 6.52E-10 |
| PA14_72390 | two_component_regulators | PGD1662602 | NA | DrhlR DcbrA vs WT | 1.152575 | 5.96E-14 |
| PA14_72520 | nucleotide_biosynthesis_and | PGD1662626 | NA | DrhlR DcbrA vs WT | -1.0908 | 4.67E-12 |
| PA14_72760 | adaptation_and_protection | PGD1662662 | NA | DrhlR DcbrA vs WT | 1.197121 | 3.57E-13 |
| PA14_72760 | antibiotic_resistance_and_su | PGD1662662 | NA | DrhlR DcbrA vs WT | 1.197121 | 3.57E-13 |
| PA14_72760 | putative_enzymes | PGD1662662 | NA | DrhlR DcbrA vs WT | 1.197121 | 3.57E-13 |
| PA14_72900 | fatty_acid_and_phospholipid | PGD1662688 | NA | DrhlR DcbrA vs WT | 1.221506 | 1.14E-11 |
| PA14_73020 | adaptation_and_protection | PGD1662708 | NA | DrhlR DcbrA vs WT | -1.5249 | 0.005676 |
| PA14_73020 | transcriptional_regulators | PGD1662708 | NA | DrhlR DcbrA vs WT | -1.5249 | 0.005676 |
| PA14_73020 | two_component_regulators | PGD1662708 | NA | DrhlR DcbrA vs WT | -1.5249 | 0.005676 |
| PA14_73050 | hypothetical_unclassified | PGD1662714 | NA | DrhlR DcbrA vs WT | -1.36435 | 0.021257 |
| PA14_73100 | hypothetical_unclassified | PGD1662722 | NA | DrhlR DcbrA vs WT | -1.28226 | 0.038354 |
| PA14_73110 | membrane_proteins | PGD1662724 | NA | DrhlR DcbrA vs WT | -1.60956 | 3.65E-16 |
| PA14_73110 | transport_of_small_molecule | PGD1662724 | NA | DrhlR DcbrA vs WT | -1.60956 | 3.65E-16 |
| PA14_73280 | energy_metabolism_CAP | PGD1662750 | atpH | DrhlR DcbrA vs WT | -1.44698 | 1.97E-13 |
| PA14_73290 | energy_metabolism_CAP | PGD1662752 | atpF | DrhlR DcbrA vs WT | -1.08956 | 4.24E-11 |
| PA14_73300 | energy_metabolism_CAP | PGD1662754 | atpE | DrhlR DcbrA vs WT | -1.0307 | 2.37E-12 |
| PA14_73420 | transcription_RNA_processin | PGD1662774 | rnpA | DrhlR DcbrA vs WT | -1.19997 | 1.72E-20 |
| PA14_73420 | translation_posttranslational | PGD1662774 | rnpA | DrhlR DcbrA vs WT | -1.19997 | 1.72E-20 |
| PA14_99999 | NA | gene_crcZ | crcZ | DrhlR DcbrA vs WT | -3.44895 | 0.001724 |
| PA14_00180 | transcription_RNA_processin | PGD1650867 | NA | DcbrA vs WT | -1.39303 | 4.45E-07 |
| PA14_00190 | amino_acid_biosynthesis_me | PGD1650869 | fmt | DcbrA vs WT | -1.02574 | 0.003716 |
| PA14_00190 | translation_posttranslational | PGD1650869 | fmt | DcbrA vs WT | -1.02574 | 0.003716 |
| PA14_00240 | translation_posttranslational | PGD1650877 | NA | DcbrA vs WT | -1.1321 | 2.86E-09 |
| PA14_00340 | transport_of_small_molecule | PGD1650891 | NA | DcbrA vs WT | -1.12801 | 0.029468 |
| PA14_00430 | transcriptional_regulators | PGD1650903 | NA | DcbrA vs WT | 1.651303 | 2.80E-08 |
| PA14_00430 | two_component_regulators | PGD1650903 | NA | DcbrA vs WT | 1.651303 | 2.80E-08 |
| PA14_00470 | hypothetical_unclassified | PGD1650911 | NA | DcbrA vs WT | 2.084875 | 4.62E-33 |
| PA14_00480 | hypothetical_unclassified | PGD1650913 | NA | DcbrA vs WT | 1.064177 | 5.26E-06 |

| | | | | | | |
|---|---|---|---|---|---|---|
| PA14_00490 | protein_secretion_export | PGD1650915 | NA | DcbrA vs WT | 1.506341 | 1.70E-05 |
| PA14_00560 | secreted_factors | PGD1650925 | exoT | DcbrA vs WT | 1.175508 | 5.99E-07 |
| PA14_00590 | fatty_acid_and_phospholipid | PGD1650931 | NA | DcbrA vs WT | -2.00063 | 1.16E-04 |
| PA14_00620 | hypothetical_unclassified | PGD1650935 | NA | DcbrA vs WT | -1.12155 | 5.08E-08 |
| PA14_00630 | hypothetical_unclassified | PGD1650937 | NA | DcbrA vs WT | -2.24625 | 7.49E-30 |
| PA14_00650 | hypothetical_unclassified | PGD1650941 | NA | DcbrA vs WT | -2.66901 | 1.04E-66 |
| PA14_00680 | transcriptional_regulators | PGD1650947 | NA | DcbrA vs WT | -1.11084 | 0.041527 |
| PA14_00710 | adaptation_and_protection | PGD1650953 | osmC | DcbrA vs WT | 1.197808 | 2.41E-05 |
| PA14_00720 | hypothetical_unclassified | PGD1650955 | NA | DcbrA vs WT | 1.536143 | 2.45E-30 |
| PA14_00820 | membrane_proteins | PGD1650975 | tagQ1 | DcbrA vs WT | -1.30318 | 2.79E-15 |
| PA14_00820 | protein_secretion_export | PGD1650975 | tagQ1 | DcbrA vs WT | -1.30318 | 2.79E-15 |
| PA14_00850 | membrane_proteins | PGD1650979 | tagS1 | DcbrA vs WT | -1.48229 | 3.64E-06 |
| PA14_00850 | protein_secretion_export | PGD1650979 | tagS1 | DcbrA vs WT | -1.48229 | 3.64E-06 |
| PA14_00850 | transport_of_small_molecule | PGD1650979 | tagS1 | DcbrA vs WT | -1.48229 | 3.64E-06 |
| PA14_00860 | protein_secretion_export | PGD1650981 | tagT1 | DcbrA vs WT | -2.4938 | 0.007692 |
| PA14_00860 | transport_of_small_molecule | PGD1650981 | tagT1 | DcbrA vs WT | -2.4938 | 0.007692 |
| PA14_00890 | protein_secretion_export | PGD1650985 | pppA | DcbrA vs WT | -1.59157 | 1.11E-06 |
| PA14_00890 | putative_enzymes | PGD1650985 | pppA | DcbrA vs WT | -1.59157 | 1.11E-06 |
| PA14_00900 | protein_secretion_export | PGD1650987 | tagF1 | DcbrA vs WT | -2.3199 | 9.92E-09 |
| PA14_00910 | hypothetical_unclassified | PGD1650989 | icmF1 | DcbrA vs WT | -1.35481 | 5.35E-11 |
| PA14_00925 | hypothetical_unclassified | PGD1650991 | tssL1 | DcbrA vs WT | -1.43759 | 6.91E-09 |
| PA14_00940 | hypothetical_unclassified | PGD1650993 | tssK1 | DcbrA vs WT | -1.32466 | 1.86E-05 |
| PA14_00960 | fatty_acid_and_phospholipid | PGD1650995 | tssJ1 | DcbrA vs WT | -1.27233 | 0.021102 |
| PA14_00980 | hypothetical_unclassified | PGD1650999 | fha1 | DcbrA vs WT | 1.020754 | 1.56E-07 |
| PA14_00990 | hypothetical_unclassified | PGD1651001 | tssA1 | DcbrA vs WT | 1.148172 | 1.14E-05 |
| PA14_01060 | protein_secretion_export | PGD1651011 | tssE1 | DcbrA vs WT | -1.10865 | 0.004472 |
| PA14_01080 | hypothetical_unclassified | PGD1651015 | tssG1 | DcbrA vs WT | -1.5492 | 3.41E-09 |
| PA14_01170 | hypothetical_unclassified | PGD1651031 | NA | DcbrA vs WT | -2.23624 | 2.03E-08 |
| PA14_01180 | hypothetical_unclassified | PGD1651033 | NA | DcbrA vs WT | -1.23471 | 3.70E-05 |
| PA14_01190 | fatty_acid_and_phospholipid | PGD1651035 | NA | DcbrA vs WT | -1.67337 | 4.18E-07 |
| PA14_01230 | hypothetical_unclassified | PGD1651041 | NA | DcbrA vs WT | -1.14215 | 1.12E-06 |
| PA14_01250 | transport_of_small_molecule | PGD1651045 | NA | DcbrA vs WT | 1.547027 | 1.05E-10 |
| PA14_01290 | energy_metabolism_CAP | PGD1651049 | coxB | DcbrA vs WT | 2.74203 | 1.02E-40 |
| PA14_01300 | energy_metabolism_CAP | PGD1651051 | coxA | DcbrA vs WT | 2.385833 | 8.22E-56 |
| PA14_01310 | central_intermediary_metab | PGD1651053 | NA | DcbrA vs WT | 2.213071 | 3.54E-20 |
| PA14_01310 | energy_metabolism_CAP | PGD1651053 | NA | DcbrA vs WT | 2.213071 | 3.54E-20 |
| PA14_01320 | energy_metabolism_CAP | PGD1651055 | coIII | DcbrA vs WT | 2.067188 | 1.23E-36 |
| PA14_01340 | hypothetical_unclassified | PGD1651059 | NA | DcbrA vs WT | 1.645415 | 6.27E-17 |
| PA14_01350 | hypothetical_unclassified | PGD1651061 | NA | DcbrA vs WT | 1.348885 | 4.93E-06 |
| PA14_01390 | energy_metabolism_CAP | PGD1651067 | NA | DcbrA vs WT | -1.28554 | 1.50E-15 |
| PA14_01540 | hypothetical_unclassified | PGD1651091 | NA | DcbrA vs WT | -1.18311 | 9.45E-05 |
| PA14_01610 | carbon_compound_catabolis | PGD1651101 | NA | DcbrA vs WT | -1.08091 | 0.001347 |
| PA14_01670 | transport_of_small_molecule | PGD1651109 | NA | DcbrA vs WT | -1.05362 | 4.29E-04 |
| PA14_01780 | nucleotide_biosynthesis_and | PGD1651127 | NA | DcbrA vs WT | -2.08416 | 8.95E-22 |
| PA14_01840 | transcriptional_regulators | PGD1651137 | NA | DcbrA vs WT | -1.20822 | 0.033476 |
| PA14_01860 | transport_of_small_molecule | PGD1651139 | NA | DcbrA vs WT | -1.2106 | 0.019301 |
| PA14_01890 | transcriptional_regulators | PGD1651143 | pcaQ | DcbrA vs WT | -1.2742 | 6.46E-06 |
| PA14_02030 | transcriptional_regulators | PGD1651165 | NA | DcbrA vs WT | -1.36854 | 8.62E-07 |
| PA14_02100 | hypothetical_unclassified | PGD1651175 | NA | DcbrA vs WT | -1.18735 | 1.44E-05 |
| PA14_02150 | motility_and_attachment | PGD1651183 | NA | DcbrA vs WT | -2.14784 | 6.18E-12 |

| | | | | | | |
|---|---|---|---|---|---|---|
| PA14_02150 | transcriptional_regulators | PGD1651183 | NA | DcbrA vs WT | -2.14784 | 6.18E-12 |
| PA14_02220 | chemotaxis | PGD1651191 | NA | DcbrA vs WT | -1.59851 | 1.23E-20 |
| PA14_02360 | transport_of_small_molecule | PGD1651211 | NA | DcbrA vs WT | -1.0636 | 0.039005 |
| PA14_02380 | fatty_acid_and_phospholipid | PGD1651215 | NA | DcbrA vs WT | 1.425244 | 1.32E-04 |
| PA14_02380 | putative_enzymes | PGD1651215 | NA | DcbrA vs WT | 1.425244 | 1.32E-04 |
| PA14_02450 | energy_metabolism_CAP | PGD1651225 | NA | DcbrA vs WT | -1.51185 | 8.30E-21 |
| PA14_02450 | transport_of_small_molecule | PGD1651225 | NA | DcbrA vs WT | -1.51185 | 8.30E-21 |
| PA14_02520 | hypothetical_unclassified | PGD1651237 | NA | DcbrA vs WT | -1.23773 | 5.72E-06 |
| PA14_02570 | carbon_compound_catabolis | PGD1651245 | mdcC | DcbrA vs WT | -3.0622 | 0.003049 |
| PA14_02610 | carbon_compound_catabolis | PGD1651251 | NA | DcbrA vs WT | -1.07082 | 0.015614 |
| PA14_02630 | membrane_proteins | PGD1651255 | NA | DcbrA vs WT | 1.140759 | 9.15E-04 |
| PA14_02630 | transport_of_small_molecule | PGD1651255 | NA | DcbrA vs WT | 1.140759 | 9.15E-04 |
| PA14_02760 | carbon_compound_catabolis | PGD1651277 | NA | DcbrA vs WT | 1.234938 | 4.29E-08 |
| PA14_02810 | membrane_proteins | PGD1651283 | pcaT | DcbrA vs WT | 1.718193 | 3.44E-22 |
| PA14_02850 | carbon_compound_catabolis | PGD1651289 | pcaC | DcbrA vs WT | 1.268661 | 4.64E-10 |
| PA14_02890 | hypothetical_unclassified | PGD1651293 | NA | DcbrA vs WT | 1.197612 | 8.80E-04 |
| PA14_02900 | carbon_compound_catabolis | PGD1651295 | pcaK | DcbrA vs WT | -1.9206 | 3.15E-11 |
| PA14_02900 | membrane_proteins | PGD1651295 | pcaK | DcbrA vs WT | -1.9206 | 3.15E-11 |
| PA14_02900 | transport_of_small_molecule | PGD1651295 | pcaK | DcbrA vs WT | -1.9206 | 3.15E-11 |
| PA14_02910 | transcriptional_regulators | PGD1651297 | NA | DcbrA vs WT | -1.51794 | 1.24E-10 |
| PA14_02960 | hypothetical_unclassified | PGD1651301 | NA | DcbrA vs WT | -1.20391 | 0.004207 |
| PA14_02990 | transport_of_small_molecule | PGD1651307 | NA | DcbrA vs WT | -1.40275 | 0.011776 |
| PA14_03110 | hypothetical_unclassified | PGD1651329 | NA | DcbrA vs WT | -2.74504 | 2.39E-05 |
| PA14_03160 | hypothetical_unclassified | PGD1651337 | NA | DcbrA vs WT | 1.134158 | 1.72E-09 |
| PA14_03166 | hypothetical_unclassified | PGD1651341 | NA | DcbrA vs WT | 1.081374 | 2.84E-10 |
| PA14_03300 | relative_phage_transposon | PGD1651367 | NA | DcbrA vs WT | 1.155534 | 5.63E-07 |
| PA14_03320 | hypothetical_unclassified | PGD1651371 | NA | DcbrA vs WT | 1.304589 | 2.59E-08 |
| PA14_03350 | hypothetical_unclassified | PGD1651377 | NA | DcbrA vs WT | 1.08433 | 1.83E-06 |
| PA14_03490 | carbon_compound_catabolis | PGD1651401 | NA | DcbrA vs WT | -1.18995 | 7.20E-13 |
| PA14_03560 | hypothetical_unclassified | PGD1651411 | NA | DcbrA vs WT | -1.5798 | 5.07E-05 |
| PA14_03590 | membrane_proteins | PGD1651415 | NA | DcbrA vs WT | -1.08186 | 5.65E-04 |
| PA14_03610 | chaperones_heat_shock | PGD1651417 | NA | DcbrA vs WT | -1.20929 | 0.003467 |
| PA14_03630 | transcriptional_regulators | PGD1651421 | NA | DcbrA vs WT | -1.05595 | 0.008736 |
| PA14_03650 | transport_of_small_molecule | PGD1651423 | cysA | DcbrA vs WT | -1.77329 | 9.93E-11 |
| PA14_03670 | membrane_proteins | PGD1651425 | cysW | DcbrA vs WT | -1.09121 | 0.001074 |
| PA14_03670 | transport_of_small_molecule | PGD1651425 | cysW | DcbrA vs WT | -1.09121 | 0.001074 |
| PA14_03680 | membrane_proteins | PGD1651427 | cysT | DcbrA vs WT | -1.83268 | 1.44E-06 |
| PA14_03680 | transport_of_small_molecule | PGD1651427 | cysT | DcbrA vs WT | -1.83268 | 1.44E-06 |
| PA14_03700 | transport_of_small_molecule | PGD1651429 | sbp | DcbrA vs WT | -1.72695 | 5.40E-08 |
| PA14_04040 | hypothetical_unclassified | PGD1651483 | NA | DcbrA vs WT | 1.232324 | 2.27E-21 |
| PA14_04230 | membrane_proteins | PGD1651513 | NA | DcbrA vs WT | -1.61112 | 0.012325 |
| PA14_04230 | transport_of_small_molecule | PGD1651513 | NA | DcbrA vs WT | -1.61112 | 0.012325 |
| PA14_04290 | amino_acid_biosynthesis_me | PGD1651521 | NA | DcbrA vs WT | -1.01015 | 3.14E-07 |
| PA14_04290 | membrane_proteins | PGD1651521 | NA | DcbrA vs WT | -1.01015 | 3.14E-07 |
| PA14_04530 | hypothetical_unclassified | PGD1651559 | NA | DcbrA vs WT | 1.092823 | 6.96E-09 |
| PA14_04580 | biosynthesis_of_cofactors | PGD1651567 | folA | DcbrA vs WT | -1.35807 | 2.27E-05 |
| PA14_04580 | central_intermediary_metab | PGD1651567 | folA | DcbrA vs WT | -1.35807 | 2.27E-05 |
| PA14_04640 | transport_of_small_molecule | PGD1651575 | NA | DcbrA vs WT | 1.301594 | 1.67E-11 |
| PA14_04650 | translation_posttranslational | PGD1651577 | pfpI | DcbrA vs WT | 1.971818 | 2.93E-24 |
| PA14_04670 | DNA_replication_recombinat | PGD1651581 | mutM | DcbrA vs WT | -1.51141 | 1.52E-13 |

| | | | | | | |
|---|---|---|---|---|---|---|
| PA14_04690 | hypothetical_unclassified | PGD1651585 | NA | DcbrA vs WT | -1.70708 | 8.61E-15 |
| PA14_04760 | central_intermediary_metab | PGD1651595 | coaD | DcbrA vs WT | 1.012291 | 4.85E-09 |
| PA14_04830 | transport_of_small_molecule | PGD1651605 | NA | DcbrA vs WT | -2.0949 | 6.61E-13 |
| PA14_04840 | putative_enzymes | PGD1651607 | NA | DcbrA vs WT | -1.43288 | 2.63E-11 |
| PA14_04970 | biosynthesis_of_cofactors | PGD1651631 | NA | DcbrA vs WT | -3.24915 | 1.14E-15 |
| PA14_05030 | hypothetical_unclassified | PGD1651641 | NA | DcbrA vs WT | 1.018808 | 1.77E-06 |
| PA14_05050 | nucleotide_biosynthesis_and | PGD1651645 | NA | DcbrA vs WT | -1.78557 | 5.54E-18 |
| PA14_05070 | amino_acid_biosynthesis_me | PGD1651649 | NA | DcbrA vs WT | -1.92697 | 2.01E-14 |
| PA14_05130 | membrane_proteins | PGD1651657 | NA | DcbrA vs WT | 1.579944 | 2.32E-16 |
| PA14_05150 | amino_acid_biosynthesis_me | PGD1651659 | proC | DcbrA vs WT | -1.10553 | 1.04E-05 |
| PA14_05210 | membrane_proteins | PGD1651669 | NA | DcbrA vs WT | 1.261508 | 4.95E-12 |
| PA14_05230 | amino_acid_biosynthesis_me | PGD1651673 | NA | DcbrA vs WT | -2.05642 | 6.36E-23 |
| PA14_05250 | nucleotide_biosynthesis_and | PGD1651675 | pyrC | DcbrA vs WT | -1.02566 | 4.68E-06 |
| PA14_05600 | hypothetical_unclassified | PGD1651735 | NA | DcbrA vs WT | -1.35877 | 2.24E-06 |
| PA14_05750 | amino_acid_biosynthesis_me | PGD1651753 | NA | DcbrA vs WT | -2.63325 | 0.011903 |
| PA14_05750 | putative_enzymes | PGD1651753 | NA | DcbrA vs WT | -2.63325 | 0.011903 |
| PA14_05770 | nucleotide_biosynthesis_and | PGD1651755 | dhT | DcbrA vs WT | -1.21092 | 2.27E-05 |
| PA14_05810 | nucleotide_biosynthesis_and | PGD1651761 | amaB | DcbrA vs WT | -1.39397 | 4.65E-06 |
| PA14_05880 | membrane_proteins | PGD1651773 | NA | DcbrA vs WT | -2.61403 | 2.86E-20 |
| PA14_06040 | hypothetical_unclassified | PGD1651795 | NA | DcbrA vs WT | 1.208665 | 2.89E-14 |
| PA14_06120 | amino_acid_biosynthesis_me | PGD1651805 | NA | DcbrA vs WT | -2.70569 | 1.01E-17 |
| PA14_06130 | adaptation_and_protection | PGD1651807 | NA | DcbrA vs WT | -1.45653 | 9.10E-10 |
| PA14_06160 | transport_of_small_molecule | PGD1651811 | NA | DcbrA vs WT | 1.243071 | 6.56E-06 |
| PA14_06350 | fatty_acid_and_phospholipid | PGD1651847 | NA | DcbrA vs WT | -1.66805 | 7.77E-09 |
| PA14_06390 | hypothetical_unclassified | PGD1651851 | NA | DcbrA vs WT | 1.668378 | 1.55E-13 |
| PA14_06430 | putative_enzymes | PGD1651857 | NA | DcbrA vs WT | 1.145676 | 0.016551 |
| PA14_06510 | biosynthesis_of_cofactors | PGD1651867 | bioF | DcbrA vs WT | -1.53225 | 4.26E-09 |
| PA14_06530 | biosynthesis_of_cofactors | PGD1651869 | NA | DcbrA vs WT | -2.08405 | 1.86E-14 |
| PA14_06540 | biosynthesis_of_cofactors | PGD1651871 | NA | DcbrA vs WT | -1.71165 | 8.29E-05 |
| PA14_06570 | biosynthesis_of_cofactors | PGD1651873 | bioD | DcbrA vs WT | -2.32705 | 1.21E-09 |
| PA14_06660 | biosynthesis_of_cofactors | PGD1651885 | nirE | DcbrA vs WT | -2.85479 | 1.10E-12 |
| PA14_06660 | energy_metabolism_CAP | PGD1651885 | nirE | DcbrA vs WT | -2.85479 | 1.10E-12 |
| PA14_06670 | energy_metabolism_CAP | PGD1651887 | nirJ | DcbrA vs WT | -1.95248 | 1.07E-12 |
| PA14_06690 | biosynthesis_of_cofactors | PGD1651891 | nirG | DcbrA vs WT | -1.57262 | 2.15E-06 |
| PA14_06690 | energy_metabolism_CAP | PGD1651891 | nirG | DcbrA vs WT | -1.57262 | 2.15E-06 |
| PA14_06690 | transcriptional_regulators | PGD1651891 | nirG | DcbrA vs WT | -1.57262 | 2.15E-06 |
| PA14_06700 | biosynthesis_of_cofactors | PGD1651893 | nirL | DcbrA vs WT | -2.63587 | 2.95E-13 |
| PA14_06700 | energy_metabolism_CAP | PGD1651893 | nirL | DcbrA vs WT | -2.63587 | 2.95E-13 |
| PA14_06720 | energy_metabolism_CAP | PGD1651897 | nirF | DcbrA vs WT | -1.3159 | 2.79E-06 |
| PA14_06730 | biosynthesis_of_cofactors | PGD1651899 | nirC | DcbrA vs WT | -1.58583 | 1.26E-05 |
| PA14_06730 | energy_metabolism_CAP | PGD1651899 | nirC | DcbrA vs WT | -1.58583 | 1.26E-05 |
| PA14_06740 | energy_metabolism_CAP | PGD1651901 | nirM | DcbrA vs WT | -3.01966 | 1.94E-26 |
| PA14_06770 | central_intermediary_metab | PGD1651905 | nirQ | DcbrA vs WT | -1.58744 | 8.70E-08 |
| PA14_06770 | energy_metabolism_CAP | PGD1651905 | nirQ | DcbrA vs WT | -1.58744 | 8.70E-08 |
| PA14_06800 | hypothetical_unclassified | PGD1651909 | NA | DcbrA vs WT | -2.45454 | 2.19E-07 |
| PA14_06810 | energy_metabolism_CAP | PGD1651911 | norC | DcbrA vs WT | -2.08646 | 2.30E-15 |
| PA14_06840 | energy_metabolism_CAP | PGD1651915 | NA | DcbrA vs WT | -1.71501 | 1.06E-10 |
| PA14_06860 | hypothetical_unclassified | PGD1651917 | NA | DcbrA vs WT | -2.00441 | 9.58E-13 |
| PA14_06920 | putative_enzymes | PGD1651929 | NA | DcbrA vs WT | -1.9882 | 2.28E-06 |
| PA14_06920 | transport_of_small_molecule | PGD1651929 | NA | DcbrA vs WT | -1.9882 | 2.28E-06 |

| | | | | | | |
|---|---|---|---|---|---|---|
| PA14_06960 | carbon_compound_catabolis | PGD1651937 | NA | DcbrA vs WT | -1.28823 | 6.69E-09 |
| PA14_06960 | energy_metabolism_CAP | PGD1651937 | NA | DcbrA vs WT | -1.28823 | 6.69E-09 |
| PA14_06960 | putative_enzymes | PGD1651937 | NA | DcbrA vs WT | -1.28823 | 6.69E-09 |
| PA14_07020 | membrane_proteins | PGD1651949 | NA | DcbrA vs WT | -1.92481 | 9.55E-16 |
| PA14_07110 | transcriptional_regulators | PGD1651963 | NA | DcbrA vs WT | -1.49373 | 2.74E-20 |
| PA14_07200 | hypothetical_unclassified | PGD1651975 | NA | DcbrA vs WT | 1.508821 | 2.38E-23 |
| PA14_07230 | carbon_compound_catabolis | PGD1651979 | fda | DcbrA vs WT | 1.697779 | 1.05E-20 |
| PA14_07230 | central_intermediary_metab | PGD1651979 | fda | DcbrA vs WT | 1.697779 | 1.05E-20 |
| PA14_07370 | membrane_proteins | PGD1652003 | NA | DcbrA vs WT | 2.703159 | 5.55E-36 |
| PA14_07430 | hypothetical_unclassified | PGD1652013 | NA | DcbrA vs WT | -2.38247 | 2.63E-19 |
| PA14_07550 | hypothetical_unclassified | PGD1652031 | NA | DcbrA vs WT | -1.20157 | 1.91E-05 |
| PA14_07710 | hypothetical_unclassified | PGD1652057 | apaG | DcbrA vs WT | -1.1385 | 7.50E-09 |
| PA14_07860 | transport_of_small_molecule | PGD1652081 | NA | DcbrA vs WT | 1.398534 | 2.73E-10 |
| PA14_08000 | hypothetical_unclassified | PGD1652105 | NA | DcbrA vs WT | 1.054151 | 1.43E-08 |
| PA14_08070 | relative_phage_transposon | PGD1652119 | NA | DcbrA vs WT | -1.11995 | 7.86E-04 |
| PA14_08100 | relative_phage_transposon | PGD1652123 | NA | DcbrA vs WT | -2.10563 | 1.74E-04 |
| PA14_08160 | putative_enzymes | PGD1652135 | NA | DcbrA vs WT | -2.07032 | 0.005043 |
| PA14_08180 | hypothetical_unclassified | PGD1652137 | NA | DcbrA vs WT | -3.08403 | 0.033248 |
| PA14_08210 | relative_phage_transposon | PGD1652143 | NA | DcbrA vs WT | 1.148276 | 0.003183 |
| PA14_08230 | hypothetical_unclassified | PGD1652147 | NA | DcbrA vs WT | -1.71424 | 0.002599 |
| PA14_08260 | relative_phage_transposon | PGD1652153 | NA | DcbrA vs WT | -2.31358 | 0.001131 |
| PA14_08330 | hypothetical_unclassified | PGD1652165 | NA | DcbrA vs WT | 1.058179 | 1.29E-04 |
| PA14_08510 | energy_metabolism_CAP | PGD1652199 | NA | DcbrA vs WT | 1.091896 | 1.78E-08 |
| PA14_09210 | central_intermediary_metab | PGD1652323 | pchA | DcbrA vs WT | -1.5098 | 4.32E-09 |
| PA14_09210 | secreted_factors | PGD1652323 | pchA | DcbrA vs WT | -1.5098 | 4.32E-09 |
| PA14_09210 | transport_of_small_molecule | PGD1652323 | pchA | DcbrA vs WT | -1.5098 | 4.32E-09 |
| PA14_09220 | central_intermediary_metab | PGD1652325 | pchB | DcbrA vs WT | -1.29934 | 1.33E-05 |
| PA14_09230 | secreted_factors | PGD1652327 | pchC | DcbrA vs WT | -2.22689 | 1.83E-11 |
| PA14_09230 | transport_of_small_molecule | PGD1652327 | pchC | DcbrA vs WT | -2.22689 | 1.83E-11 |
| PA14_09240 | central_intermediary_metab | PGD1652329 | pchD | DcbrA vs WT | -1.41353 | 1.33E-06 |
| PA14_09240 | secreted_factors | PGD1652329 | pchD | DcbrA vs WT | -1.41353 | 1.33E-06 |
| PA14_09240 | transport_of_small_molecule | PGD1652329 | pchD | DcbrA vs WT | -1.41353 | 1.33E-06 |
| PA14_09260 | transcriptional_regulators | PGD1652331 | pchR | DcbrA vs WT | -1.05984 | 6.08E-07 |
| PA14_09270 | transport_of_small_molecule | PGD1652333 | pchE | DcbrA vs WT | -2.10028 | 4.23E-16 |
| PA14_09280 | transport_of_small_molecule | PGD1652335 | pchF | DcbrA vs WT | -2.01134 | 1.42E-14 |
| PA14_09290 | central_intermediary_metab | PGD1652337 | pchG | DcbrA vs WT | -1.43444 | 6.21E-08 |
| PA14_09290 | membrane_proteins | PGD1652337 | pchG | DcbrA vs WT | -1.43444 | 6.21E-08 |
| PA14_09290 | transport_of_small_molecule | PGD1652337 | pchG | DcbrA vs WT | -1.43444 | 6.21E-08 |
| PA14_09300 | transport_of_small_molecule | PGD1652339 | NA | DcbrA vs WT | -2.30566 | 1.36E-16 |
| PA14_09320 | transport_of_small_molecule | PGD1652341 | NA | DcbrA vs WT | -1.87026 | 2.02E-11 |
| PA14_09350 | hypothetical_unclassified | PGD1652345 | NA | DcbrA vs WT | -2.52287 | 1.05E-11 |
| PA14_09370 | antibiotic_resistance_and_su | PGD1652347 | NA | DcbrA vs WT | -2.46279 | 1.58E-13 |
| PA14_09370 | membrane_proteins | PGD1652347 | NA | DcbrA vs WT | -2.46279 | 1.58E-13 |
| PA14_09380 | antibiotic_resistance_and_su | PGD1652349 | NA | DcbrA vs WT | -1.58724 | 4.95E-09 |
| PA14_09380 | membrane_proteins | PGD1652349 | NA | DcbrA vs WT | -1.58724 | 4.95E-09 |
| PA14_09380 | transport_of_small_molecule | PGD1652349 | NA | DcbrA vs WT | -1.58724 | 4.95E-09 |
| PA14_09400 | putative_enzymes | PGD1652351 | phzS | DcbrA vs WT | -3.14341 | 8.90E-19 |
| PA14_09410 | secreted_factors | PGD1652353 | phzG1 | DcbrA vs WT | -2.2577 | 0.001062 |
| PA14_09450 | secreted_factors | PGD1652359 | phzD1 | DcbrA vs WT | -2.64218 | 5.07E-05 |
| PA14_09460 | secreted_factors | PGD1652361 | phzC1 | DcbrA vs WT | -2.06225 | 1.86E-04 |

| | | | | | | |
|---|---|---|---|---|---|---|
| PA14_09470 | secreted_factors | PGD1652363 | phzB1 | DcbrA vs WT | -2.08652 | 9.87E-06 |
| PA14_09480 | secreted_factors | PGD1652365 | phzA1 | DcbrA vs WT | -2.79237 | 7.75E-07 |
| PA14_09500 | membrane_proteins | PGD1652369 | opmD | DcbrA vs WT | -2.72879 | 1.36E-10 |
| PA14_09500 | transport_of_small_molecule | PGD1652369 | opmD | DcbrA vs WT | -2.72879 | 1.36E-10 |
| PA14_09530 | transport_of_small_molecule | PGD1652373 | mexH | DcbrA vs WT | -1.4739 | 0.003095 |
| PA14_09540 | membrane_proteins | PGD1652375 | mexG | DcbrA vs WT | -1.65695 | 4.97E-04 |
| PA14_09570 | transcriptional_regulators | PGD1652379 | NA | DcbrA vs WT | -1.82677 | 0.001851 |
| PA14_09580 | putative_enzymes | PGD1652381 | NA | DcbrA vs WT | -2.04758 | 5.79E-07 |
| PA14_09600 | cell_wall_LPS | PGD1652383 | ddlA | DcbrA vs WT | -1.67385 | 2.54E-23 |
| PA14_09690 | two_component_regulators | PGD1652393 | NA | DcbrA vs WT | -2.04878 | 2.09E-14 |
| PA14_09780 | hypothetical_unclassified | PGD1652409 | NA | DcbrA vs WT | 1.065623 | 2.26E-06 |
| PA14_09900 | putative_enzymes | PGD1652425 | prpL | DcbrA vs WT | -1.82028 | 6.71E-11 |
| PA14_09900 | secreted_factors | PGD1652425 | prpL | DcbrA vs WT | -1.82028 | 6.71E-11 |
| PA14_09930 | DNA_replication_recombinat | PGD1652431 | NA | DcbrA vs WT | 1.323467 | 1.61E-06 |
| PA14_09940 | putative_enzymes | PGD1652433 | NA | DcbrA vs WT | 1.439863 | 2.25E-13 |
| PA14_10140 | transport_of_small_molecule | PGD1652465 | fepG | DcbrA vs WT | -2.9074 | 0.001831 |
| PA14_10170 | transport_of_small_molecule | PGD1652469 | fepB | DcbrA vs WT | -2.0322 | 8.21E-07 |
| PA14_10210 | energy_metabolism_CAP | PGD1652477 | NA | DcbrA vs WT | -2.36983 | 6.34E-07 |
| PA14_10260 | carbon_compound_catabolis | PGD1652487 | NA | DcbrA vs WT | -1.86151 | 0.002425 |
| PA14_10280 | putative_enzymes | PGD1652491 | NA | DcbrA vs WT | -2.39354 | 0.038792 |
| PA14_10340 | protein_secretion_export | PGD1652501 | NA | DcbrA vs WT | 1.23937 | 4.37E-07 |
| PA14_10350 | protein_secretion_export | PGD1652503 | NA | DcbrA vs WT | 1.627087 | 2.06E-20 |
| PA14_10360 | hypothetical_unclassified | PGD1652505 | NA | DcbrA vs WT | 1.083854 | 8.00E-07 |
| PA14_10370 | fatty_acid_and_phospholipid | PGD1652507 | NA | DcbrA vs WT | 2.350601 | 3.31E-19 |
| PA14_10380 | hypothetical_unclassified | PGD1652509 | NA | DcbrA vs WT | 3.303523 | 2.33E-111 |
| PA14_10490 | hypothetical_unclassified | PGD1652523 | NA | DcbrA vs WT | -1.50806 | 7.30E-35 |
| PA14_10530 | transcriptional_regulators | PGD1652527 | NA | DcbrA vs WT | -1.08477 | 2.04E-17 |
| PA14_10540 | energy_metabolism_CAP | PGD1652529 | NA | DcbrA vs WT | -1.76974 | 7.79E-39 |
| PA14_10560 | hypothetical_unclassified | PGD1652533 | NA | DcbrA vs WT | -1.62774 | 1.99E-13 |
| PA14_10570 | carbon_compound_catabolis | PGD1652535 | NA | DcbrA vs WT | -1.63092 | 1.37E-04 |
| PA14_10830 | transcriptional_regulators | PGD1652577 | NA | DcbrA vs WT | 1.404909 | 5.80E-26 |
| PA14_10850 | putative_enzymes | PGD1652581 | NA | DcbrA vs WT | -1.35792 | 2.19E-05 |
| PA14_10910 | transport_of_small_molecule | PGD1652589 | NA | DcbrA vs WT | -2.27942 | 5.37E-06 |
| PA14_10940 | transcriptional_regulators | PGD1652593 | NA | DcbrA vs WT | 1.472877 | 2.82E-20 |
| PA14_10950 | hypothetical_unclassified | PGD1652595 | NA | DcbrA vs WT | -1.93211 | 3.02E-06 |
| PA14_10990 | carbon_compound_catabolis | PGD1652603 | hpaC | DcbrA vs WT | -2.16705 | 1.08E-08 |
| PA14_11010 | hypothetical_unclassified | PGD1652607 | NA | DcbrA vs WT | -1.1479 | 0.003471 |
| PA14_11060 | motility_and_attachment | PGD1652615 | cupB1 | DcbrA vs WT | 1.661934 | 1.71E-04 |
| PA14_11130 | putative_enzymes | PGD1652629 | NA | DcbrA vs WT | -1.02924 | 1.16E-10 |
| PA14_11140 | biosynthesis_of_cofactors | PGD1652631 | NA | DcbrA vs WT | -1.75052 | 1.78E-14 |
| PA14_11170 | hypothetical_unclassified | PGD1652637 | NA | DcbrA vs WT | -1.53972 | 5.31E-19 |
| PA14_11270 | membrane_proteins | PGD1652653 | oprG | DcbrA vs WT | -1.25052 | 1.55E-14 |
| PA14_11280 | fatty_acid_and_phospholipid | PGD1652655 | NA | DcbrA vs WT | 1.209014 | 5.65E-06 |
| PA14_11350 | hypothetical_unclassified | PGD1652667 | NA | DcbrA vs WT | -1.02079 | 0.001996 |
| PA14_11370 | fatty_acid_and_phospholipid | PGD1652669 | NA | DcbrA vs WT | -1.4807 | 2.59E-06 |
| PA14_11380 | nucleotide_biosynthesis_and | PGD1652671 | nrdR | DcbrA vs WT | -1.06496 | 1.65E-11 |
| PA14_11380 | transcriptional_regulators | PGD1652671 | nrdR | DcbrA vs WT | -1.06496 | 1.65E-11 |
| PA14_11410 | biosynthesis_of_cofactors | PGD1652675 | ribC | DcbrA vs WT | -1.22959 | 6.20E-09 |
| PA14_11670 | hypothetical_unclassified | PGD1652719 | NA | DcbrA vs WT | 1.288832 | 0.02815 |
| PA14_11740 | hypothetical_unclassified | PGD1652731 | NA | DcbrA vs WT | -1.44637 | 1.30E-14 |

| | | | | | | |
|---|---|---|---|---|---|---|
| PA14_11880 | hypothetical_unclassified | PGD1652749 | NA | DcbrA vs WT | -1.37605 | 1.50E-06 |
| PA14_11890 | hypothetical_unclassified | PGD1652751 | NA | DcbrA vs WT | -1.10574 | 3.20E-09 |
| PA14_11960 | membrane_proteins | PGD1652763 | NA | DcbrA vs WT | -1.35934 | 2.16E-07 |
| PA14_11990 | membrane_proteins | PGD1652769 | NA | DcbrA vs WT | -1.12024 | 6.04E-04 |
| PA14_11990 | putative_enzymes | PGD1652769 | NA | DcbrA vs WT | -1.12024 | 6.04E-04 |
| PA14_12050 | hypothetical_unclassified | PGD1652777 | NA | DcbrA vs WT | -1.04031 | 4.76E-08 |
| PA14_12300 | adaptation_and_protection | PGD1652817 | NA | DcbrA vs WT | 1.216557 | 3.45E-27 |
| PA14_12310 | hypothetical_unclassified | PGD1652819 | NA | DcbrA vs WT | 1.017814 | 2.86E-09 |
| PA14_12400 | biosynthesis_of_cofactors | PGD1652831 | thiE | DcbrA vs WT | -1.53484 | 4.88E-08 |
| PA14_12410 | biosynthesis_of_cofactors | PGD1652833 | thiD | DcbrA vs WT | -1.44938 | 7.68E-12 |
| PA14_12440 | transcriptional_regulators | PGD1652837 | NA | DcbrA vs WT | -1.80335 | 7.12E-11 |
| PA14_12470 | hypothetical_unclassified | PGD1652841 | NA | DcbrA vs WT | -1.55291 | 8.03E-05 |
| PA14_12530 | hypothetical_unclassified | PGD1652845 | NA | DcbrA vs WT | -1.04314 | 5.76E-07 |
| PA14_12540 | putative_enzymes | PGD1652847 | NA | DcbrA vs WT | -1.96835 | 2.97E-10 |
| PA14_12540 | transcription_RNA_processin | PGD1652847 | NA | DcbrA vs WT | -1.96835 | 2.97E-10 |
| PA14_12560 | membrane_proteins | PGD1652851 | NA | DcbrA vs WT | 1.239234 | 6.29E-09 |
| PA14_12570 | transcriptional_regulators | PGD1652853 | NA | DcbrA vs WT | 1.092385 | 1.88E-06 |
| PA14_12620 | hypothetical_unclassified | PGD1652859 | NA | DcbrA vs WT | 1.91915 | 1.40E-45 |
| PA14_12640 | hypothetical_unclassified | PGD1652863 | NA | DcbrA vs WT | -1.7943 | 0.003551 |
| PA14_12650 | hypothetical_unclassified | PGD1652865 | NA | DcbrA vs WT | -2.10265 | 3.67E-12 |
| PA14_12670 | DNA_replication_recombinat | PGD1652867 | NA | DcbrA vs WT | -1.21329 | 7.02E-17 |
| PA14_12750 | hypothetical_unclassified | PGD1652881 | NA | DcbrA vs WT | 1.673897 | 1.30E-19 |
| PA14_12810 | two_component_regulators | PGD1652889 | NA | DcbrA vs WT | 1.033291 | 3.24E-07 |
| PA14_12860 | hypothetical_unclassified | PGD1652897 | NA | DcbrA vs WT | -1.15711 | 1.30E-10 |
| PA14_12920 | transport_of_small_molecule | PGD1652907 | NA | DcbrA vs WT | -2.30717 | 1.77E-08 |
| PA14_12940 | transport_of_small_molecule | PGD1652909 | NA | DcbrA vs WT | -2.29455 | 5.14E-06 |
| PA14_12960 | transport_of_small_molecule | PGD1652911 | NA | DcbrA vs WT | -1.55502 | 0.02285 |
| PA14_12970 | carbon_compound_catabolis | PGD1652913 | tauD | DcbrA vs WT | -2.74456 | 1.04E-05 |
| PA14_13000 | transcriptional_regulators | PGD1652919 | NA | DcbrA vs WT | -1.74323 | 1.18E-07 |
| PA14_13050 | hypothetical_unclassified | PGD1652927 | NA | DcbrA vs WT | -4.03577 | 8.96E-42 |
| PA14_13140 | hypothetical_unclassified | PGD1652939 | NA | DcbrA vs WT | -2.201 | 8.20E-25 |
| PA14_13170 | transport_of_small_molecule | PGD1652943 | NA | DcbrA vs WT | -2.53685 | 1.85E-25 |
| PA14_13230 | biosynthesis_of_cofactors | PGD1652953 | moaC | DcbrA vs WT | -1.17488 | 2.52E-13 |
| PA14_13240 | biosynthesis_of_cofactors | PGD1652955 | moaD | DcbrA vs WT | -1.6489 | 1.90E-08 |
| PA14_13250 | biosynthesis_of_cofactors | PGD1652957 | moaE | DcbrA vs WT | -1.39125 | 6.33E-10 |
| PA14_13260 | biosynthesis_of_cofactors | PGD1652959 | moaB1 | DcbrA vs WT | -1.13925 | 2.43E-05 |
| PA14_13280 | biosynthesis_of_cofactors | PGD1652961 | moeA1 | DcbrA vs WT | -1.56161 | 7.61E-07 |
| PA14_13320 | fatty_acid_and_phospholipid | PGD1652967 | NA | DcbrA vs WT | -1.19412 | 7.39E-05 |
| PA14_13370 | hypothetical_unclassified | PGD1652977 | NA | DcbrA vs WT | -1.09398 | 3.13E-07 |
| PA14_13390 | hypothetical_unclassified | PGD1652981 | NA | DcbrA vs WT | -3.38446 | 1.96E-29 |
| PA14_13430 | transport_of_small_molecule | PGD1652987 | fecA | DcbrA vs WT | -1.34705 | 0.008871 |
| PA14_13450 | membrane_proteins | PGD1652989 | NA | DcbrA vs WT | -1.35191 | 0.02021 |
| PA14_13460 | transcriptional_regulators | PGD1652991 | NA | DcbrA vs WT | 1.447142 | 0.005171 |
| PA14_13580 | transport_of_small_molecule | PGD1653007 | NA | DcbrA vs WT | 1.684538 | 1.97E-09 |
| PA14_13590 | membrane_proteins | PGD1653009 | NA | DcbrA vs WT | 1.876195 | 2.16E-11 |
| PA14_13590 | transport_of_small_molecule | PGD1653009 | NA | DcbrA vs WT | 1.876195 | 2.16E-11 |
| PA14_13600 | transport_of_small_molecule | PGD1653011 | NA | DcbrA vs WT | 1.407135 | 1.14E-09 |
| PA14_13610 | membrane_proteins | PGD1653013 | NA | DcbrA vs WT | 1.462739 | 1.14E-12 |
| PA14_13610 | transport_of_small_molecule | PGD1653013 | NA | DcbrA vs WT | 1.462739 | 1.14E-12 |
| PA14_13630 | hypothetical_unclassified | PGD1653017 | NA | DcbrA vs WT | 1.167271 | 2.22E-08 |

| | | | | | | |
|---|---|---|---|---|---|---|
| PA14_13680 | biosynthesis_of_cofactors | PGD1653025 | NA | DcbrA vs WT | -1.33438 | 5.94E-05 |
| PA14_13690 | biosynthesis_of_cofactors | PGD1653027 | NA | DcbrA vs WT | -1.12464 | 7.80E-05 |
| PA14_13720 | hypothetical_unclassified | PGD1653031 | NA | DcbrA vs WT | -1.78039 | 2.13E-07 |
| PA14_13750 | membrane_proteins | PGD1653037 | narK1 | DcbrA vs WT | -1.00358 | 9.82E-05 |
| PA14_13750 | transport_of_small_molecule | PGD1653037 | narK1 | DcbrA vs WT | -1.00358 | 9.82E-05 |
| PA14_13810 | energy_metabolism_CAP | PGD1653045 | narJ | DcbrA vs WT | -1.63545 | 2.15E-06 |
| PA14_13890 | relative_phage_transposon | PGD1653059 | NA | DcbrA vs WT | 1.116749 | 1.62E-06 |
| PA14_14020 | amino_acid_biosynthesis_me | PGD1653079 | NA | DcbrA vs WT | -1.28745 | 0.010747 |
| PA14_14100 | transport_of_small_molecule | PGD1653087 | NA | DcbrA vs WT | -1.7883 | 2.38E-25 |
| PA14_14270 | putative_enzymes | PGD1653111 | NA | DcbrA vs WT | -2.03219 | 5.65E-28 |
| PA14_14280 | transcriptional_regulators | PGD1653113 | NA | DcbrA vs WT | -1.23857 | 9.67E-06 |
| PA14_14330 | chaperones_heat_shock | PGD1653123 | NA | DcbrA vs WT | 1.105068 | 0.020526 |
| PA14_14330 | protein_secretion_export | PGD1653123 | NA | DcbrA vs WT | 1.105068 | 0.020526 |
| PA14_14330 | secreted_factors | PGD1653123 | NA | DcbrA vs WT | 1.105068 | 0.020526 |
| PA14_14370 | transport_of_small_molecule | PGD1653129 | NA | DcbrA vs WT | -1.36816 | 2.15E-10 |
| PA14_14490 | amino_acid_biosynthesis_me | PGD1653151 | NA | DcbrA vs WT | 1.032622 | 4.51E-05 |
| PA14_14500 | membrane_proteins | PGD1653153 | NA | DcbrA vs WT | 1.235582 | 5.95E-29 |
| PA14_14560 | hypothetical_unclassified | PGD1653165 | NA | DcbrA vs WT | -1.56503 | 1.16E-07 |
| PA14_14660 | membrane_proteins | PGD1653179 | NA | DcbrA vs WT | 1.009026 | 7.28E-07 |
| PA14_14660 | protein_secretion_export | PGD1653179 | NA | DcbrA vs WT | 1.009026 | 7.28E-07 |
| PA14_14990 | energy_metabolism_CAP | PGD1653227 | NA | DcbrA vs WT | 1.799442 | 3.57E-43 |
| PA14_15050 | membrane_proteins | PGD1653235 | NA | DcbrA vs WT | -1.02686 | 2.62E-15 |
| PA14_15070 | transport_of_small_molecule | PGD1653237 | oprC | DcbrA vs WT | -1.51459 | 3.00E-18 |
| PA14_15080 | membrane_proteins | PGD1653239 | NA | DcbrA vs WT | -1.12229 | 4.28E-14 |
| PA14_15100 | membrane_proteins | PGD1653243 | NA | DcbrA vs WT | -1.18936 | 4.31E-21 |
| PA14_15160 | hypothetical_unclassified | PGD1653255 | NA | DcbrA vs WT | -1.20237 | 0.001268 |
| PA14_15380 | relative_phage_transposon | PGD1653289 | repA | DcbrA vs WT | 1.097876 | 8.59E-04 |
| PA14_15470 | adaptation_and_protection | PGD1653305 | merP | DcbrA vs WT | 1.003075 | 0.033503 |
| PA14_15475 | hypothetical_unclassified | PGD1653307 | merT | DcbrA vs WT | 1.443492 | 1.84E-05 |
| PA14_15490 | hypothetical_unclassified | PGD1653311 | NA | DcbrA vs WT | 1.163834 | 2.92E-05 |
| PA14_15520 | relative_phage_transposon | PGD1653317 | trbJ | DcbrA vs WT | 1.067174 | 2.54E-05 |
| PA14_15570 | relative_phage_transposon | PGD1653325 | NA | DcbrA vs WT | 1.010985 | 2.33E-05 |
| PA14_15630 | hypothetical_unclassified | PGD1653337 | NA | DcbrA vs WT | 1.416021 | 8.50E-09 |
| PA14_15680 | nucleotide_biosynthesis_and | PGD1653345 | NA | DcbrA vs WT | -1.11145 | 1.75E-11 |
| PA14_15710 | hypothetical_unclassified | PGD1653349 | NA | DcbrA vs WT | -1.23015 | 0.002684 |
| PA14_15870 | transport_of_small_molecule | PGD1653375 | NA | DcbrA vs WT | -1.02087 | 1.09E-14 |
| PA14_15880 | hypothetical_unclassified | PGD1653377 | NA | DcbrA vs WT | -1.24591 | 1.29E-05 |
| PA14_16010 | fatty_acid_and_phospholipid | PGD1653399 | NA | DcbrA vs WT | 1.139236 | 0.028269 |
| PA14_16030 | membrane_proteins | PGD1653403 | NA | DcbrA vs WT | 1.035615 | 2.62E-15 |
| PA14_16030 | transport_of_small_molecule | PGD1653403 | NA | DcbrA vs WT | 1.035615 | 2.62E-15 |
| PA14_16290 | hypothetical_unclassified | PGD1653443 | NA | DcbrA vs WT | 1.988663 | 6.30E-11 |
| PA14_16330 | hypothetical_unclassified | PGD1653451 | NA | DcbrA vs WT | -1.08358 | 8.34E-15 |
| PA14_16370 | membrane_proteins | PGD1653459 | NA | DcbrA vs WT | -1.93418 | 1.77E-22 |
| PA14_16630 | membrane_proteins | PGD1653499 | NA | DcbrA vs WT | 1.286554 | 1.05E-06 |
| PA14_16630 | transport_of_small_molecule | PGD1653499 | NA | DcbrA vs WT | 1.286554 | 1.05E-06 |
| PA14_16690 | central_intermediary_metab | PGD1653509 | ppc | DcbrA vs WT | -1.3913 | 2.12E-21 |
| PA14_16690 | energy_metabolism_CAP | PGD1653509 | ppc | DcbrA vs WT | -1.3913 | 2.12E-21 |
| PA14_16720 | hypothetical_unclassified | PGD1653515 | pemA | DcbrA vs WT | -2.05973 | 6.35E-18 |
| PA14_16740 | carbon_compound_catabolis | PGD1653519 | NA | DcbrA vs WT | -1.11954 | 3.93E-06 |
| PA14_16770 | hypothetical_unclassified | PGD1653523 | NA | DcbrA vs WT | -1.14491 | 1.59E-04 |

| | | | | | | |
|---|---|---|---|---|---|---|
| PA14_16800 | transport_of_small_molecule | PGD1653529 | NA | DcbrA vs WT | -1.98744 | 3.23E-17 |
| PA14_16870 | transport_of_small_molecule | PGD1653539 | NA | DcbrA vs WT | -1.12043 | 1.14E-05 |
| PA14_16990 | hypothetical_unclassified | PGD1653559 | NA | DcbrA vs WT | 3.147324 | 4.65E-17 |
| PA14_17000 | hypothetical_unclassified | PGD1653561 | NA | DcbrA vs WT | -3.49963 | 6.69E-04 |
| PA14_17410 | putative_enzymes | PGD1653627 | NA | DcbrA vs WT | -1.04602 | 0.001171 |
| PA14_17580 | hypothetical_unclassified | PGD1653657 | NA | DcbrA vs WT | -2.24313 | 2.82E-24 |
| PA14_17590 | hypothetical_unclassified | PGD1653659 | NA | DcbrA vs WT | 1.664252 | 5.25E-16 |
| PA14_17600 | protein_secretion_export | PGD1653661 | NA | DcbrA vs WT | 1.062075 | 3.70E-08 |
| PA14_17690 | membrane_proteins | PGD1653679 | NA | DcbrA vs WT | 1.83266 | 1.57E-50 |
| PA14_17690 | transport_of_small_molecule | PGD1653679 | NA | DcbrA vs WT | 1.83266 | 1.57E-50 |
| PA14_17790 | transcriptional_regulators | PGD1653695 | NA | DcbrA vs WT | 1.262646 | 8.14E-09 |
| PA14_18040 | hypothetical_unclassified | PGD1653729 | NA | DcbrA vs WT | 1.422245 | 1.22E-08 |
| PA14_18100 | hypothetical_unclassified | PGD1653741 | NA | DcbrA vs WT | -1.26194 | 2.12E-04 |
| PA14_18160 | energy_metabolism_CAP | PGD1653751 | NA | DcbrA vs WT | -1.17047 | 3.22E-05 |
| PA14_18160 | putative_enzymes | PGD1653751 | NA | DcbrA vs WT | -1.17047 | 3.22E-05 |
| PA14_18180 | antibiotic_resistance_and_su | PGD1653753 | NA | DcbrA vs WT | -1.76557 | 0.019821 |
| PA14_18200 | transcriptional_regulators | PGD1653755 | NA | DcbrA vs WT | -1.54348 | 2.83E-10 |
| PA14_18210 | DNA_replication_recombinat | PGD1653757 | NA | DcbrA vs WT | -2.55284 | 2.13E-08 |
| PA14_18250 | central_intermediary_metab | PGD1653761 | NA | DcbrA vs WT | -1.05687 | 3.25E-04 |
| PA14_18250 | transport_of_small_molecule | PGD1653761 | NA | DcbrA vs WT | -1.05687 | 3.25E-04 |
| PA14_18260 | central_intermediary_metab | PGD1653763 | fruK | DcbrA vs WT | -2.29038 | 3.58E-05 |
| PA14_18260 | transport_of_small_molecule | PGD1653763 | fruK | DcbrA vs WT | -2.29038 | 3.58E-05 |
| PA14_18340 | carbon_compound_catabolis | PGD1653775 | NA | DcbrA vs WT | -2.80184 | 0.016008 |
| PA14_18350 | adaptation_and_protection | PGD1653777 | NA | DcbrA vs WT | 1.268584 | 2.38E-04 |
| PA14_18350 | antibiotic_resistance_and_su | PGD1653777 | NA | DcbrA vs WT | 1.268584 | 2.38E-04 |
| PA14_18350 | cell_wall_LPS | PGD1653777 | NA | DcbrA vs WT | 1.268584 | 2.38E-04 |
| PA14_18350 | putative_enzymes | PGD1653777 | NA | DcbrA vs WT | 1.268584 | 2.38E-04 |
| PA14_18350 | translation_posttranslational | PGD1653777 | NA | DcbrA vs WT | 1.268584 | 2.38E-04 |
| PA14_18370 | amino_acid_biosynthesis_me | PGD1653781 | NA | DcbrA vs WT | -1.66115 | 0.00338 |
| PA14_18580 | adaptation_and_protection | PGD1653805 | algD | DcbrA vs WT | 1.276824 | 1.22E-04 |
| PA14_18580 | cell_wall_LPS | PGD1653805 | algD | DcbrA vs WT | 1.276824 | 1.22E-04 |
| PA14_18580 | secreted_factors | PGD1653805 | algD | DcbrA vs WT | 1.276824 | 1.22E-04 |
| PA14_18590 | hypothetical_unclassified | PGD1653807 | NA | DcbrA vs WT | 1.162256 | 2.42E-04 |
| PA14_18620 | hypothetical_unclassified | PGD1653813 | NA | DcbrA vs WT | -1.03645 | 2.01E-07 |
| PA14_18630 | putative_enzymes | PGD1653815 | NA | DcbrA vs WT | -1.73419 | 7.21E-28 |
| PA14_18690 | adaptation_and_protection | PGD1653827 | NA | DcbrA vs WT | 1.053745 | 4.66E-13 |
| PA14_18690 | putative_enzymes | PGD1653827 | NA | DcbrA vs WT | 1.053745 | 4.66E-13 |
| PA14_18690 | translation_posttranslational | PGD1653827 | NA | DcbrA vs WT | 1.053745 | 4.66E-13 |
| PA14_18810 | hypothetical_unclassified | PGD1653847 | NA | DcbrA vs WT | 1.297095 | 1.21E-06 |
| PA14_18900 | energy_metabolism_CAP | PGD1653863 | NA | DcbrA vs WT | -1.65093 | 1.85E-07 |
| PA14_19030 | hypothetical_unclassified | PGD1653883 | NA | DcbrA vs WT | -1.15563 | 2.79E-09 |
| PA14_19110 | secreted_factors | PGD1653893 | rhlB | DcbrA vs WT | 1.140795 | 2.80E-07 |
| PA14_19120 | transcriptional_regulators | PGD1653895 | rhlR | DcbrA vs WT | 1.252582 | 0.003157 |
| PA14_19205 | hypothetical_unclassified | PGD1653909 | NA | DcbrA vs WT | -1.76932 | 5.77E-05 |
| PA14_19270 | transport_of_small_molecule | PGD1653915 | NA | DcbrA vs WT | -1.93336 | 3.28E-06 |
| PA14_19330 | hypothetical_unclassified | PGD1653923 | NA | DcbrA vs WT | -1.32441 | 1.04E-05 |
| PA14_19340 | two_component_regulators | PGD1653925 | NA | DcbrA vs WT | -1.50596 | 7.55E-23 |
| PA14_19350 | carbon_compound_catabolis | PGD1653927 | NA | DcbrA vs WT | 1.642431 | 2.51E-11 |
| PA14_19370 | amino_acid_biosynthesis_me | PGD1653931 | NA | DcbrA vs WT | 1.207732 | 1.35E-17 |
| PA14_19390 | membrane_proteins | PGD1653935 | NA | DcbrA vs WT | -1.37352 | 8.87E-10 |

| | | | | | | |
|---|---|---|---|---|---|---|
| PA14_19400 | amino_acid_biosynthesis_me | PGD1653937 | mnmC | DcbrA vs WT | -1.34301 | 2.02E-08 |
| PA14_19480 | hypothetical_unclassified | PGD1653947 | NA | DcbrA vs WT | 1.417299 | 2.92E-16 |
| PA14_19500 | transport_of_small_molecule | PGD1653951 | NA | DcbrA vs WT | -1.13472 | 0.02693 |
| PA14_19530 | central_intermediary_metab | PGD1653957 | NA | DcbrA vs WT | -1.71701 | 8.83E-05 |
| PA14_19560 | central_intermediary_metab | PGD1653961 | ssuD | DcbrA vs WT | -2.7957 | 6.56E-04 |
| PA14_19580 | transport_of_small_molecule | PGD1653965 | ssuB | DcbrA vs WT | -3.11757 | 0.016709 |
| PA14_19650 | membrane_proteins | PGD1653979 | NA | DcbrA vs WT | -2.01935 | 0.024601 |
| PA14_19750 | hypothetical_unclassified | PGD1653999 | NA | DcbrA vs WT | -2.56904 | 1.12E-06 |
| PA14_19800 | transcriptional_regulators | PGD1654003 | NA | DcbrA vs WT | 1.409478 | 1.68E-12 |
| PA14_19850 | transcriptional_regulators | PGD1654009 | NA | DcbrA vs WT | -1.84189 | 6.84E-07 |
| PA14_19870 | amino_acid_biosynthesis_me | PGD1654013 | ldh | DcbrA vs WT | -1.07353 | 1.73E-06 |
| PA14_19900 | energy_metabolism_CAP | PGD1654015 | NA | DcbrA vs WT | -2.09994 | 4.46E-18 |
| PA14_19910 | energy_metabolism_CAP | PGD1654017 | NA | DcbrA vs WT | -1.9663 | 2.43E-18 |
| PA14_19920 | energy_metabolism_CAP | PGD1654019 | NA | DcbrA vs WT | -2.18909 | 3.79E-28 |
| PA14_19930 | hypothetical_unclassified | PGD1654021 | NA | DcbrA vs WT | -1.57834 | 1.22E-04 |
| PA14_19940 | hypothetical_unclassified | PGD1654023 | NA | DcbrA vs WT | -1.60819 | 8.28E-05 |
| PA14_20000 | membrane_proteins | PGD1654033 | NA | DcbrA vs WT | -2.52835 | 2.47E-04 |
| PA14_20010 | transport_of_small_molecule | PGD1654035 | hasR | DcbrA vs WT | -1.37641 | 0.026245 |
| PA14_20020 | transport_of_small_molecule | PGD1654037 | hasAp | DcbrA vs WT | -2.1436 | 0.002103 |
| PA14_20030 | protein_secretion_export | PGD1654039 | hasD | DcbrA vs WT | -3.3893 | 0.003145 |
| PA14_20040 | protein_secretion_export | PGD1654041 | hasE | DcbrA vs WT | -2.88462 | 0.003045 |
| PA14_20060 | hypothetical_unclassified | PGD1654045 | NA | DcbrA vs WT | 1.377151 | 9.51E-11 |
| PA14_20070 | membrane_proteins | PGD1654047 | NA | DcbrA vs WT | -1.21065 | 0.005743 |
| PA14_20120 | hypothetical_unclassified | PGD1654055 | NA | DcbrA vs WT | -2.0557 | 2.14E-24 |
| PA14_20150 | energy_metabolism_CAP | PGD1654061 | nosL | DcbrA vs WT | -2.28335 | 1.14E-10 |
| PA14_20180 | energy_metabolism_CAP | PGD1654065 | nosF | DcbrA vs WT | -1.86005 | 9.58E-07 |
| PA14_20180 | transport_of_small_molecule | PGD1654065 | nosF | DcbrA vs WT | -1.86005 | 9.58E-07 |
| PA14_20260 | hypothetical_unclassified | PGD1654077 | NA | DcbrA vs WT | -1.14705 | 1.94E-04 |
| PA14_20430 | transport_of_small_molecule | PGD1654105 | phnM | DcbrA vs WT | -1.73646 | 1.66E-05 |
| PA14_20440 | transport_of_small_molecule | PGD1654107 | phnN | DcbrA vs WT | -1.15789 | 3.12E-05 |
| PA14_20460 | hypothetical_unclassified | PGD1654111 | NA | DcbrA vs WT | 1.288244 | 1.32E-10 |
| PA14_20470 | membrane_proteins | PGD1654113 | NA | DcbrA vs WT | 1.9355 | 1.63E-18 |
| PA14_20480 | membrane_proteins | PGD1654115 | NA | DcbrA vs WT | 1.327009 | 2.52E-09 |
| PA14_20560 | carbon_compound_catabolis | PGD1654129 | amiE | DcbrA vs WT | 2.24892 | 3.04E-28 |
| PA14_20640 | membrane_proteins | PGD1654145 | NA | DcbrA vs WT | -1.51714 | 0.014008 |
| PA14_20680 | membrane_proteins | PGD1654151 | NA | DcbrA vs WT | -1.01102 | 6.69E-13 |
| PA14_20890 | cell_wall_LPS | PGD1654187 | rfaD | DcbrA vs WT | -2.41382 | 7.83E-21 |
| PA14_20900 | membrane_proteins | PGD1654189 | NA | DcbrA vs WT | -1.04166 | 1.44E-04 |
| PA14_20950 | fatty_acid_and_phospholipid | PGD1654195 | fabH2 | DcbrA vs WT | -1.09016 | 3.24E-04 |
| PA14_20980 | putative_enzymes | PGD1654201 | NA | DcbrA vs WT | -1.18831 | 1.47E-05 |
| PA14_21010 | energy_metabolism_CAP | PGD1654205 | NA | DcbrA vs WT | -1.3741 | 1.09E-05 |
| PA14_21050 | putative_enzymes | PGD1654213 | NA | DcbrA vs WT | -1.07572 | 7.61E-05 |
| PA14_21080 | transcriptional_regulators | PGD1654219 | NA | DcbrA vs WT | -1.07534 | 0.004487 |
| PA14_21120 | hypothetical_unclassified | PGD1654225 | NA | DcbrA vs WT | 2.631002 | 1.74E-56 |
| PA14_21160 | transport_of_small_molecule | PGD1654233 | NA | DcbrA vs WT | -2.12259 | 6.67E-04 |
| PA14_21220 | hypothetical_unclassified | PGD1654243 | NA | DcbrA vs WT | -2.71154 | 1.64E-20 |
| PA14_21530 | hypothetical_unclassified | PGD1654289 | NA | DcbrA vs WT | -1.02784 | 0.003941 |
| PA14_21570 | hypothetical_unclassified | PGD1654297 | NA | DcbrA vs WT | 1.433587 | 0.021095 |
| PA14_21600 | membrane_proteins | PGD1654303 | NA | DcbrA vs WT | 1.883577 | 0.008161 |
| PA14_21610 | transport_of_small_molecule | PGD1654305 | oprO | DcbrA vs WT | 2.049807 | 3.70E-10 |

| | | | | | | |
|---|---|---|---|---|---|---|
| PA14_21620 | transport_of_small_molecule | PGD1654307 | oprP | DcbrA vs WT | 1.213218 | 3.00E-04 |
| PA14_21670 | hypothetical_unclassified | PGD1654317 | NA | DcbrA vs WT | 2.896811 | 1.39E-49 |
| PA14_21680 | hypothetical_unclassified | PGD1654319 | NA | DcbrA vs WT | 1.191273 | 1.25E-06 |
| PA14_21730 | transport_of_small_molecule | PGD1654329 | NA | DcbrA vs WT | 1.062723 | 4.35E-07 |
| PA14_21830 | hypothetical_unclassified | PGD1654347 | NA | DcbrA vs WT | 1.619897 | 4.44E-33 |
| PA14_21860 | hypothetical_unclassified | PGD1654353 | NA | DcbrA vs WT | -2.05947 | 7.02E-15 |
| PA14_21920 | membrane_proteins | PGD1654365 | NA | DcbrA vs WT | 1.039232 | 3.89E-04 |
| PA14_21920 | transport_of_small_molecule | PGD1654365 | NA | DcbrA vs WT | 1.039232 | 3.89E-04 |
| PA14_21980 | translation_posttranslational | PGD1654375 | NA | DcbrA vs WT | -1.0141 | 6.40E-06 |
| PA14_22110 | hypothetical_unclassified | PGD1654399 | NA | DcbrA vs WT | 1.485851 | 9.74E-05 |
| PA14_22120 | hypothetical_unclassified | PGD1654401 | NA | DcbrA vs WT | 1.23274 | 3.33E-05 |
| PA14_22240 | hypothetical_unclassified | PGD1654419 | NA | DcbrA vs WT | 2.34531 | 0.008846 |
| PA14_22320 | membrane_proteins | PGD1654433 | NA | DcbrA vs WT | -2.35416 | 1.95E-06 |
| PA14_22340 | membrane_proteins | PGD1654437 | NA | DcbrA vs WT | 1.674402 | 2.60E-11 |
| PA14_22350 | membrane_proteins | PGD1654439 | actP | DcbrA vs WT | 1.63404 | 2.09E-11 |
| PA14_22400 | membrane_proteins | PGD1654445 | NA | DcbrA vs WT | 2.445163 | 4.12E-23 |
| PA14_22410 | hypothetical_unclassified | PGD1654447 | NA | DcbrA vs WT | 1.193704 | 1.52E-13 |
| PA14_22420 | hypothetical_unclassified | PGD1654449 | NA | DcbrA vs WT | 2.854317 | 4.01E-21 |
| PA14_22470 | transcriptional_regulators | PGD1654457 | NA | DcbrA vs WT | -1.57787 | 1.84E-11 |
| PA14_22500 | translation_posttranslational | PGD1654463 | NA | DcbrA vs WT | 1.151687 | 4.21E-04 |
| PA14_22510 | hypothetical_unclassified | PGD1654465 | NA | DcbrA vs WT | 1.649251 | 0.016192 |
| PA14_22520 | cell_wall_LPS | PGD1654467 | NA | DcbrA vs WT | 1.119797 | 0.003997 |
| PA14_22560 | membrane_proteins | PGD1654475 | NA | DcbrA vs WT | -1.35691 | 5.16E-10 |
| PA14_22650 | transport_of_small_molecule | PGD1654491 | NA | DcbrA vs WT | -1.38508 | 5.79E-10 |
| PA14_22710 | energy_metabolism_CAP | PGD1654503 | NA | DcbrA vs WT | -1.88744 | 5.97E-10 |
| PA14_22760 | transcriptional_regulators | PGD1654511 | cpxR | DcbrA vs WT | -1.10553 | 1.04E-05 |
| PA14_22760 | two_component_regulators | PGD1654511 | cpxR | DcbrA vs WT | -1.10553 | 1.04E-05 |
| PA14_22780 | putative_enzymes | PGD1654515 | yciI | DcbrA vs WT | -1.08903 | 8.93E-08 |
| PA14_22800 | cell_division | PGD1654517 | yciB | DcbrA vs WT | 1.247418 | 3.08E-17 |
| PA14_22800 | membrane_proteins | PGD1654517 | yciB | DcbrA vs WT | 1.247418 | 3.08E-17 |
| PA14_22840 | hypothetical_unclassified | PGD1654523 | NA | DcbrA vs WT | 1.306937 | 2.61E-11 |
| PA14_22890 | carbon_compound_catabolis | PGD1654531 | gapA | DcbrA vs WT | 1.236742 | 1.37E-09 |
| PA14_22890 | energy_metabolism_CAP | PGD1654531 | gapA | DcbrA vs WT | 1.236742 | 1.37E-09 |
| PA14_23100 | DNA_replication_recombinat | PGD1654561 | NA | DcbrA vs WT | 1.034565 | 1.76E-10 |
| PA14_23160 | transport_of_small_molecule | PGD1654569 | gltS | DcbrA vs WT | -1.28716 | 0.019242 |
| PA14_23320 | nucleotide_biosynthesis_and | PGD1654595 | cmk | DcbrA vs WT | -1.18276 | 1.92E-10 |
| PA14_23480 | DNA_replication_recombinat | PGD1654627 | NA | DcbrA vs WT | 1.037911 | 2.93E-08 |
| PA14_23520 | transport_of_small_molecule | PGD1654635 | NA | DcbrA vs WT | 1.01096 | 2.31E-04 |
| PA14_23530 | transport_of_small_molecule | PGD1654637 | NA | DcbrA vs WT | 1.960949 | 7.09E-13 |
| PA14_23680 | chaperones_heat_shock | PGD1654661 | ibpA | DcbrA vs WT | 1.963879 | 6.48E-07 |
| PA14_23690 | protein_secretion_export | PGD1654663 | NA | DcbrA vs WT | -1.21093 | 1.49E-06 |
| PA14_23760 | amino_acid_biosynthesis_me | PGD1654673 | leuD | DcbrA vs WT | 1.383595 | 4.17E-11 |
| PA14_23790 | amino_acid_biosynthesis_me | PGD1654677 | leuB | DcbrA vs WT | 1.106726 | 1.64E-18 |
| PA14_23950 | putative_enzymes | PGD1654701 | NA | DcbrA vs WT | -1.27877 | 1.54E-13 |
| PA14_24040 | protein_secretion_export | PGD1654713 | xcpU | DcbrA vs WT | -1.80549 | 4.91E-17 |
| PA14_24050 | protein_secretion_export | PGD1654715 | xcpV | DcbrA vs WT | -2.29621 | 2.57E-25 |
| PA14_24060 | protein_secretion_export | PGD1654717 | xcpW | DcbrA vs WT | -1.27839 | 7.38E-10 |
| PA14_24080 | protein_secretion_export | PGD1654721 | xcpY | DcbrA vs WT | -1.72887 | 1.61E-22 |
| PA14_24100 | protein_secretion_export | PGD1654723 | xcpZ | DcbrA vs WT | -3.17196 | 1.23E-20 |
| PA14_24210 | hypothetical_unclassified | PGD1654741 | NA | DcbrA vs WT | -1.93107 | 1.65E-23 |

| | | | | | | |
|---|---|---|---|---|---|---|
| PA14_24245 | hypothetical_unclassified | PGD1654749 | NA | DcbrA vs WT | 1.040875 | 4.28E-09 |
| PA14_24310 | putative_enzymes | PGD1654759 | NA | DcbrA vs WT | 1.020729 | 2.60E-08 |
| PA14_24350 | transcriptional_regulators | PGD1654765 | NA | DcbrA vs WT | -1.67705 | 1.43E-06 |
| PA14_24350 | two_component_regulators | PGD1654765 | NA | DcbrA vs WT | -1.67705 | 1.43E-06 |
| PA14_24390 | putative_enzymes | PGD1654773 | NA | DcbrA vs WT | -1.00962 | 6.90E-06 |
| PA14_24480 | cell_wall_LPS | PGD1654787 | pelA | DcbrA vs WT | 1.843875 | 6.82E-45 |
| PA14_24510 | cell_wall_LPS | PGD1654793 | pelD | DcbrA vs WT | 1.67971 | 6.63E-13 |
| PA14_24530 | cell_wall_LPS | PGD1654795 | pelE | DcbrA vs WT | 1.25563 | 1.28E-04 |
| PA14_24550 | cell_wall_LPS | PGD1654797 | pelF | DcbrA vs WT | 2.570119 | 8.13E-26 |
| PA14_24560 | cell_wall_LPS | PGD1654799 | pelG | DcbrA vs WT | 2.68292 | 1.09E-47 |
| PA14_24560 | secreted_factors | PGD1654799 | pelG | DcbrA vs WT | 2.68292 | 1.09E-47 |
| PA14_24590 | hypothetical_unclassified | PGD1654805 | NA | DcbrA vs WT | 1.177106 | 1.35E-07 |
| PA14_24630 | hypothetical_unclassified | PGD1654813 | NA | DcbrA vs WT | -1.45973 | 1.14E-11 |
| PA14_24650 | translation_posttranslational | PGD1654817 | rmf | DcbrA vs WT | 1.371118 | 1.12E-08 |
| PA14_24730 | amino_acid_biosynthesis_me | PGD1654831 | NA | DcbrA vs WT | 1.202327 | 1.15E-17 |
| PA14_24760 | membrane_proteins | PGD1654835 | NA | DcbrA vs WT | 1.012461 | 3.34E-06 |
| PA14_24790 | transport_of_small_molecule | PGD1654841 | NA | DcbrA vs WT | 1.216021 | 1.52E-10 |
| NA | NA | PGD1654856 | NA | DcbrA vs WT | 1.203749 | 3.34E-05 |
| PA14_24880 | fatty_acid_and_phospholipid | PGD1654857 | NA | DcbrA vs WT | 1.427409 | 4.60E-11 |
| PA14_24890 | biosynthesis_of_cofactors | PGD1654859 | mobA | DcbrA vs WT | -1.05022 | 7.72E-07 |
| PA14_24950 | carbon_compound_catabolis | PGD1654869 | NA | DcbrA vs WT | -1.81896 | 1.76E-07 |
| PA14_24950 | energy_metabolism_CAP | PGD1654869 | NA | DcbrA vs WT | -1.81896 | 1.76E-07 |
| PA14_25040 | adaptation_and_protection | PGD1654885 | NA | DcbrA vs WT | -1.1007 | 3.49E-14 |
| PA14_25100 | hypothetical_unclassified | PGD1654895 | NA | DcbrA vs WT | 1.037767 | 1.13E-07 |
| PA14_25480 | membrane_proteins | PGD1654953 | NA | DcbrA vs WT | -1.3624 | 3.85E-07 |
| PA14_25520 | hypothetical_unclassified | PGD1654961 | NA | DcbrA vs WT | 1.135585 | 3.50E-04 |
| PA14_25710 | biosynthesis_of_cofactors | PGD1654993 | pabC | DcbrA vs WT | -1.63498 | 3.85E-17 |
| PA14_25740 | nucleotide_biosynthesis_and | PGD1654997 | tmk | DcbrA vs WT | -1.15143 | 1.55E-05 |
| PA14_25930 | biosynthesis_of_cofactors | PGD1655027 | NA | DcbrA vs WT | -2.06267 | 5.43E-04 |
| PA14_25970 | biosynthesis_of_cofactors | PGD1655033 | cobN | DcbrA vs WT | -1.03854 | 8.35E-06 |
| PA14_25980 | amino_acid_biosynthesis_me | PGD1655035 | aroF | DcbrA vs WT | -1.51169 | 6.67E-04 |
| PA14_26000 | biosynthesis_of_cofactors | PGD1655039 | NA | DcbrA vs WT | -2.17861 | 8.02E-09 |
| PA14_26020 | secreted_factors | PGD1655043 | NA | DcbrA vs WT | -1.10365 | 1.69E-05 |
| PA14_26140 | transcriptional_regulators | PGD1655059 | NA | DcbrA vs WT | -1.23223 | 0.001207 |
| PA14_26165 | hypothetical_unclassified | PGD1655065 | NA | DcbrA vs WT | 1.101069 | 1.86E-11 |
| PA14_26200 | hypothetical_unclassified | PGD1655069 | NA | DcbrA vs WT | -4.01404 | 2.09E-06 |
| PA14_26300 | hypothetical_unclassified | PGD1655085 | NA | DcbrA vs WT | -1.85762 | 0.004214 |
| PA14_26330 | transcriptional_regulators | PGD1655089 | NA | DcbrA vs WT | 1.700814 | 1.37E-14 |
| PA14_26340 | membrane_proteins | PGD1655091 | NA | DcbrA vs WT | 1.676691 | 0.015514 |
| PA14_26350 | putative_enzymes | PGD1655093 | NA | DcbrA vs WT | -1.18209 | 7.40E-09 |
| PA14_26360 | membrane_proteins | PGD1655095 | NA | DcbrA vs WT | -1.4465 | 0.044067 |
| PA14_26400 | transport_of_small_molecule | PGD1655099 | NA | DcbrA vs WT | -2.25979 | 0.025622 |
| PA14_26460 | biosynthesis_of_cofactors | PGD1655105 | cobK | DcbrA vs WT | -1.59187 | 2.09E-04 |
| PA14_26470 | biosynthesis_of_cofactors | PGD1655107 | cbiD | DcbrA vs WT | -1.57869 | 3.25E-11 |
| PA14_26530 | biosynthesis_of_cofactors | PGD1655117 | cobJ | DcbrA vs WT | -1.51543 | 6.58E-06 |
| PA14_26600 | transcriptional_regulators | PGD1655131 | sbrI | DcbrA vs WT | 1.166477 | 2.16E-16 |
| PA14_26650 | putative_enzymes | PGD1655139 | NA | DcbrA vs WT | -1.50671 | 2.99E-04 |
| PA14_26670 | fatty_acid_and_phospholipid | PGD1655141 | NA | DcbrA vs WT | -1.54553 | 5.30E-10 |
| PA14_26720 | fatty_acid_and_phospholipid | PGD1655147 | NA | DcbrA vs WT | -1.1045 | 3.43E-06 |
| PA14_26720 | putative_enzymes | PGD1655147 | NA | DcbrA vs WT | -1.1045 | 3.43E-06 |

| | | | | | | |
|---|---|---|---|---|---|---|
| PA14_26730 | putative_enzymes | PGD1655149 | NA | DcbrA vs WT | -1.3223 | 3.66E-04 |
| PA14_26770 | membrane_proteins | PGD1655155 | NA | DcbrA vs WT | 1.626057 | 1.01E-32 |
| PA14_26770 | putative_enzymes | PGD1655155 | NA | DcbrA vs WT | 1.626057 | 1.01E-32 |
| PA14_26780 | hypothetical_unclassified | PGD1655157 | NA | DcbrA vs WT | 2.324316 | 8.36E-25 |
| PA14_26860 | transcriptional_regulators | PGD1655165 | NA | DcbrA vs WT | -1.17421 | 1.84E-07 |
| PA14_26940 | hypothetical_unclassified | PGD1655179 | NA | DcbrA vs WT | -2.52615 | 1.29E-14 |
| PA14_26970 | two_component_regulators | PGD1655183 | NA | DcbrA vs WT | -1.658 | 1.18E-26 |
| PA14_27000 | adaptation_and_protection | PGD1655189 | NA | DcbrA vs WT | -1.10447 | 2.77E-10 |
| PA14_27000 | chemotaxis | PGD1655189 | NA | DcbrA vs WT | -1.10447 | 2.77E-10 |
| PA14_27090 | protein_secretion_export | PGD1655197 | lipH | DcbrA vs WT | -1.32805 | 0.044067 |
| PA14_27090 | secreted_factors | PGD1655197 | lipH | DcbrA vs WT | -1.32805 | 0.044067 |
| PA14_27220 | adaptation_and_protection | PGD1655223 | ohr | DcbrA vs WT | -2.3318 | 9.87E-23 |
| PA14_27270 | membrane_proteins | PGD1655229 | NA | DcbrA vs WT | -1.52933 | 1.08E-08 |
| PA14_27630 | hypothetical_unclassified | PGD1655291 | NA | DcbrA vs WT | 1.834979 | 1.11E-14 |
| PA14_27640 | hypothetical_unclassified | PGD1655293 | NA | DcbrA vs WT | 1.82404 | 5.32E-17 |
| PA14_27675 | hypothetical_unclassified | PGD1655299 | NA | DcbrA vs WT | 1.648375 | 6.60E-27 |
| PA14_27680 | hypothetical_unclassified | PGD1655301 | NA | DcbrA vs WT | 2.22258 | 8.67E-28 |
| PA14_27710 | hypothetical_unclassified | PGD1655307 | NA | DcbrA vs WT | 1.020079 | 4.58E-06 |
| PA14_27720 | hypothetical_unclassified | PGD1655309 | NA | DcbrA vs WT | -1.10805 | 3.11E-08 |
| PA14_27800 | two_component_regulators | PGD1655321 | NA | DcbrA vs WT | -1.09442 | 7.50E-08 |
| PA14_27870 | hypothetical_unclassified | PGD1655331 | NA | DcbrA vs WT | 1.826002 | 2.62E-24 |
| PA14_27930 | hypothetical_unclassified | PGD1655343 | NA | DcbrA vs WT | -1.36711 | 5.73E-13 |
| PA14_27940 | transcriptional_regulators | PGD1655345 | NA | DcbrA vs WT | 1.281733 | 1.22E-16 |
| PA14_27940 | two_component_regulators | PGD1655345 | NA | DcbrA vs WT | 1.281733 | 1.22E-16 |
| PA14_27950 | two_component_regulators | PGD1655347 | NA | DcbrA vs WT | 1.023923 | 9.81E-06 |
| PA14_28050 | adaptation_and_protection | PGD1655365 | NA | DcbrA vs WT | -2.05463 | 6.68E-24 |
| PA14_28050 | chemotaxis | PGD1655365 | NA | DcbrA vs WT | -2.05463 | 6.68E-24 |
| PA14_28070 | hypothetical_unclassified | PGD1655369 | NA | DcbrA vs WT | -1.2161 | 0.001163 |
| PA14_28130 | transcriptional_regulators | PGD1655381 | NA | DcbrA vs WT | 1.649475 | 7.72E-17 |
| PA14_28150 | hypothetical_unclassified | PGD1655385 | NA | DcbrA vs WT | -1.42625 | 9.44E-10 |
| PA14_28170 | transport_of_small_molecule | PGD1655387 | NA | DcbrA vs WT | 1.730959 | 2.09E-28 |
| PA14_28210 | membrane_proteins | PGD1655395 | NA | DcbrA vs WT | -1.1537 | 2.34E-05 |
| PA14_28220 | membrane_proteins | PGD1655397 | NA | DcbrA vs WT | 2.055743 | 5.43E-27 |
| PA14_28230 | hypothetical_unclassified | PGD1655399 | NA | DcbrA vs WT | -1.71129 | 1.58E-09 |
| PA14_28350 | hypothetical_unclassified | PGD1655421 | NA | DcbrA vs WT | 1.06349 | 7.44E-10 |
| PA14_28360 | hypothetical_unclassified | PGD1655423 | NA | DcbrA vs WT | 1.013855 | 0.013487 |
| PA14_28390 | membrane_proteins | PGD1655429 | NA | DcbrA vs WT | -1.64288 | 0.002874 |
| PA14_28460 | hypothetical_unclassified | PGD1655443 | NA | DcbrA vs WT | -1.1235 | 1.01E-10 |
| PA14_28500 | hypothetical_unclassified | PGD1655449 | NA | DcbrA vs WT | -1.63364 | 6.21E-08 |
| PA14_28520 | hypothetical_unclassified | PGD1655453 | NA | DcbrA vs WT | 1.293835 | 9.34E-08 |
| PA14_28530 | membrane_proteins | PGD1655455 | NA | DcbrA vs WT | 2.43987 | 8.31E-70 |
| PA14_28560 | hypothetical_unclassified | PGD1655459 | NA | DcbrA vs WT | -2.39377 | 2.48E-05 |
| PA14_28610 | hypothetical_unclassified | PGD1655469 | NA | DcbrA vs WT | -1.71587 | 6.48E-09 |
| PA14_28750 | DNA_replication_recombinat | PGD1655493 | NA | DcbrA vs WT | 1.741006 | 3.77E-09 |
| PA14_28880 | hypothetical_unclassified | PGD1655517 | NA | DcbrA vs WT | -1.01668 | 0.007775 |
| PA14_28930 | hypothetical_unclassified | PGD1655525 | NA | DcbrA vs WT | -1.99928 | 1.51E-04 |
| PA14_28980 | membrane_proteins | PGD1655535 | NA | DcbrA vs WT | -2.62589 | 3.61E-05 |
| PA14_28980 | transcriptional_regulators | PGD1655535 | NA | DcbrA vs WT | -2.62589 | 3.61E-05 |
| PA14_29120 | hypothetical_unclassified | PGD1655561 | NA | DcbrA vs WT | 1.59576 | 8.12E-22 |
| PA14_29160 | hypothetical_unclassified | PGD1655567 | NA | DcbrA vs WT | 1.395767 | 9.38E-21 |

| | | | | | | |
|---|---|---|---|---|---|---|
| PA14_29190 | adaptation_and_protection | PGD1655571 | NA | DcbrA vs WT | -1.36696 | 0.002179 |
| PA14_29230 | putative_enzymes | PGD1655579 | NA | DcbrA vs WT | -1.56016 | 6.45E-10 |
| PA14_29250 | membrane_proteins | PGD1655583 | NA | DcbrA vs WT | -1.37012 | 0.030848 |
| PA14_29320 | putative_enzymes | PGD1655595 | NA | DcbrA vs WT | -1.70701 | 4.27E-06 |
| PA14_29400 | cell_wall_LPS | PGD1655608 | NA | DcbrA vs WT | -1.51326 | 4.14E-22 |
| PA14_29440 | transcriptional_regulators | PGD1655614 | NA | DcbrA vs WT | -1.21044 | 3.08E-04 |
| PA14_29460 | energy_metabolism_CAP | PGD1655616 | NA | DcbrA vs WT | -1.29745 | 2.31E-04 |
| PA14_29470 | hypothetical_unclassified | PGD1655618 | NA | DcbrA vs WT | 1.274461 | 3.83E-12 |
| PA14_29480 | transport_of_small_molecule | PGD1655620 | NA | DcbrA vs WT | 1.199018 | 1.55E-04 |
| PA14_29490 | protein_secretion_export | PGD1655622 | NA | DcbrA vs WT | 1.377044 | 9.76E-07 |
| PA14_29550 | hypothetical_unclassified | PGD1655634 | NA | DcbrA vs WT | 1.596867 | 3.52E-09 |
| PA14_29560 | hypothetical_unclassified | PGD1655636 | NA | DcbrA vs WT | 1.047078 | 0.001308 |
| PA14_29650 | hypothetical_unclassified | PGD1655650 | NA | DcbrA vs WT | -1.51995 | 0.003258 |
| PA14_29660 | membrane_proteins | PGD1655652 | NA | DcbrA vs WT | -1.26643 | 0.007486 |
| PA14_29750 | hypothetical_unclassified | PGD1655666 | NA | DcbrA vs WT | -1.11209 | 0.001259 |
| PA14_29820 | transport_of_small_molecule | PGD1655674 | NA | DcbrA vs WT | 1.43067 | 1.99E-42 |
| PA14_29850 | antibiotic_resistance_and_su | PGD1655678 | nuoN | DcbrA vs WT | 1.458852 | 2.16E-15 |
| PA14_29850 | energy_metabolism_CAP | PGD1655678 | nuoN | DcbrA vs WT | 1.458852 | 2.16E-15 |
| PA14_29860 | energy_metabolism_CAP | PGD1655680 | nuoM | DcbrA vs WT | 1.504978 | 4.06E-12 |
| PA14_29920 | energy_metabolism_CAP | PGD1655688 | nuoI | DcbrA vs WT | 1.904198 | 5.07E-11 |
| PA14_29930 | energy_metabolism_CAP | PGD1655690 | nuoH | DcbrA vs WT | 1.438792 | 2.15E-15 |
| PA14_29980 | energy_metabolism_CAP | PGD1655696 | nuoE | DcbrA vs WT | 1.265325 | 1.84E-19 |
| PA14_29990 | energy_metabolism_CAP | PGD1655698 | nuoD | DcbrA vs WT | 1.285928 | 1.05E-10 |
| PA14_30010 | energy_metabolism_CAP | PGD1655700 | nuoB | DcbrA vs WT | 1.71385 | 1.57E-32 |
| PA14_30020 | energy_metabolism_CAP | PGD1655702 | nuoA | DcbrA vs WT | 1.509903 | 1.97E-12 |
| PA14_30080 | hypothetical_unclassified | PGD1655712 | NA | DcbrA vs WT | -1.64373 | 1.44E-15 |
| PA14_30200 | adaptation_and_protection | PGD1655732 | cspD | DcbrA vs WT | 1.086771 | 5.60E-08 |
| PA14_30200 | transcriptional_regulators | PGD1655732 | cspD | DcbrA vs WT | 1.086771 | 5.60E-08 |
| PA14_30390 | energy_metabolism_CAP | PGD1655764 | NA | DcbrA vs WT | -2.25929 | 6.06E-13 |
| PA14_30400 | energy_metabolism_CAP | PGD1655766 | NA | DcbrA vs WT | -2.4794 | 1.81E-18 |
| PA14_30430 | transport_of_small_molecule | PGD1655772 | NA | DcbrA vs WT | -1.07007 | 7.63E-04 |
| PA14_30500 | putative_enzymes | PGD1655784 | NA | DcbrA vs WT | -1.03823 | 0.049899 |
| PA14_30650 | transcriptional_regulators | PGD1655806 | gacA | DcbrA vs WT | 1.115159 | 2.46E-19 |
| PA14_30690 | hypothetical_unclassified | PGD1655814 | NA | DcbrA vs WT | 1.056109 | 6.70E-08 |
| PA14_30970 | transcriptional_regulators | PGD1655866 | NA | DcbrA vs WT | 1.981102 | 1.28E-06 |
| PA14_31010 | protein_secretion_export | PGD1655874 | NA | DcbrA vs WT | 1.380683 | 4.86E-13 |
| PA14_31090 | hypothetical_unclassified | PGD1655888 | NA | DcbrA vs WT | -3.25669 | 0.012549 |
| PA14_31170 | hypothetical_unclassified | PGD1655900 | NA | DcbrA vs WT | -2.58192 | 8.28E-05 |
| PA14_31190 | hypothetical_unclassified | PGD1655904 | NA | DcbrA vs WT | -1.23386 | 0.041489 |
| PA14_31220 | hypothetical_unclassified | PGD1655908 | NA | DcbrA vs WT | 1.032035 | 0.005263 |
| PA14_31230 | hypothetical_unclassified | PGD1655910 | NA | DcbrA vs WT | -1.24123 | 0.014005 |
| PA14_31240 | transport_of_small_molecule | PGD1655912 | NA | DcbrA vs WT | 1.089281 | 5.16E-10 |
| PA14_31300 | hypothetical_unclassified | PGD1655924 | NA | DcbrA vs WT | 1.048656 | 1.90E-05 |
| PA14_31350 | energy_metabolism_CAP | PGD1655932 | NA | DcbrA vs WT | 1.239093 | 2.89E-12 |
| PA14_31380 | transport_of_small_molecule | PGD1655938 | NA | DcbrA vs WT | 1.653281 | 8.72E-10 |
| PA14_31480 | transcriptional_regulators | PGD1655956 | NA | DcbrA vs WT | -1.09533 | 1.55E-08 |
| PA14_31510 | biosynthesis_of_cofactors | PGD1655960 | NA | DcbrA vs WT | -2.75927 | 1.82E-26 |
| PA14_31530 | fatty_acid_and_phospholipid | PGD1655962 | NA | DcbrA vs WT | -2.82507 | 4.20E-29 |
| PA14_31530 | putative_enzymes | PGD1655962 | NA | DcbrA vs WT | -2.82507 | 4.20E-29 |
| PA14_31540 | energy_metabolism_CAP | PGD1655964 | NA | DcbrA vs WT | -2.29939 | 4.96E-25 |

| | | | | | | |
|---|---|---|---|---|---|---|
| PA14_31680 | cell_wall_LPS | PGD1655982 | NA | DcbrA vs WT | 1.206252 | 2.62E-15 |
| PA14_31720 | fatty_acid_and_phospholipid | PGD1655988 | NA | DcbrA vs WT | -1.06862 | 1.04E-08 |
| PA14_31730 | putative_enzymes | PGD1655990 | NA | DcbrA vs WT | -1.30418 | 1.97E-05 |
| PA14_31870 | antibiotic_resistance_and_su | PGD1656012 | NA | DcbrA vs WT | 1.098981 | 5.03E-10 |
| PA14_31870 | transport_of_small_molecule | PGD1656012 | NA | DcbrA vs WT | 1.098981 | 5.03E-10 |
| PA14_31990 | transport_of_small_molecule | PGD1656028 | czcB | DcbrA vs WT | -1.49874 | 0.026111 |
| PA14_32080 | carbon_compound_catabolis | PGD1656034 | xylX | DcbrA vs WT | 1.455795 | 0.003148 |
| PA14_32110 | energy_metabolism_CAP | PGD1656038 | xylZ | DcbrA vs WT | -1.90517 | 0.021637 |
| PA14_32140 | energy_metabolism_CAP | PGD1656042 | antC | DcbrA vs WT | 1.1351 | 0.02638 |
| PA14_32150 | biosynthesis_of_cofactors | PGD1656044 | antB | DcbrA vs WT | 1.992093 | 5.59E-04 |
| PA14_32160 | carbon_compound_catabolis | PGD1656046 | antA | DcbrA vs WT | 2.039486 | 3.66E-10 |
| PA14_32230 | carbon_compound_catabolis | PGD1656054 | catC | DcbrA vs WT | 1.254169 | 1.40E-04 |
| PA14_32280 | hypothetical_unclassified | PGD1656062 | NA | DcbrA vs WT | -1.16155 | 5.73E-09 |
| PA14_32350 | hypothetical_unclassified | PGD1656074 | NA | DcbrA vs WT | -2.191 | 0.004504 |
| PA14_32400 | antibiotic_resistance_and_su | PGD1656084 | mexE | DcbrA vs WT | 1.169779 | 0.004473 |
| PA14_32400 | transport_of_small_molecule | PGD1656084 | mexE | DcbrA vs WT | 1.169779 | 0.004473 |
| PA14_32440 | hypothetical_unclassified | PGD1656090 | NA | DcbrA vs WT | 1.433879 | 4.97E-08 |
| PA14_32460 | transcriptional_regulators | PGD1656094 | NA | DcbrA vs WT | -1.05925 | 0.034525 |
| PA14_32480 | hypothetical_unclassified | PGD1656098 | NA | DcbrA vs WT | 1.24151 | 1.03E-05 |
| PA14_32490 | hypothetical_unclassified | PGD1656100 | NA | DcbrA vs WT | 2.288203 | 1.05E-21 |
| PA14_32570 | two_component_regulators | PGD1656110 | NA | DcbrA vs WT | -1.98072 | 1.93E-11 |
| PA14_32590 | chaperones_heat_shock | PGD1656114 | NA | DcbrA vs WT | -1.77773 | 4.83E-13 |
| PA14_32590 | membrane_proteins | PGD1656114 | NA | DcbrA vs WT | -1.77773 | 4.83E-13 |
| PA14_32590 | putative_enzymes | PGD1656114 | NA | DcbrA vs WT | -1.77773 | 4.83E-13 |
| PA14_32600 | chaperones_heat_shock | PGD1656116 | NA | DcbrA vs WT | -1.15114 | 2.04E-04 |
| PA14_32600 | membrane_proteins | PGD1656116 | NA | DcbrA vs WT | -1.15114 | 2.04E-04 |
| PA14_32600 | putative_enzymes | PGD1656116 | NA | DcbrA vs WT | -1.15114 | 2.04E-04 |
| PA14_32690 | carbon_compound_catabolis | PGD1656130 | gtdA | DcbrA vs WT | -1.49017 | 0.011556 |
| PA14_32890 | hypothetical_unclassified | PGD1656162 | NA | DcbrA vs WT | 1.053536 | 3.36E-05 |
| PA14_32930 | hypothetical_unclassified | PGD1656166 | NA | DcbrA vs WT | -1.62053 | 2.61E-15 |
| PA14_33030 | amino_acid_biosynthesis_me | PGD1656180 | sdaA | DcbrA vs WT | -1.43502 | 1.41E-15 |
| PA14_33040 | amino_acid_biosynthesis_me | PGD1656182 | gcvT2 | DcbrA vs WT | -1.31228 | 1.94E-11 |
| PA14_33040 | central_intermediary_metab | PGD1656182 | gcvT2 | DcbrA vs WT | -1.31228 | 1.94E-11 |
| PA14_33050 | hypothetical_unclassified | PGD1656184 | NA | DcbrA vs WT | 1.845437 | 4.42E-21 |
| PA14_33060 | hypothetical_unclassified | PGD1656186 | NA | DcbrA vs WT | 1.366413 | 2.88E-06 |
| PA14_33070 | membrane_proteins | PGD1656188 | NA | DcbrA vs WT | -1.39898 | 1.44E-05 |
| PA14_33110 | membrane_proteins | PGD1656192 | NA | DcbrA vs WT | -1.35182 | 0.002918 |
| PA14_33160 | hypothetical_unclassified | PGD1656200 | NA | DcbrA vs WT | 1.756397 | 3.01E-16 |
| PA14_33190 | hypothetical_unclassified | PGD1656204 | NA | DcbrA vs WT | -1.21106 | 6.31E-07 |
| PA14_33250 | hypothetical_unclassified | PGD1656214 | NA | DcbrA vs WT | -1.53383 | 1.83E-06 |
| PA14_33270 | adaptation_and_protection | PGD1656218 | pvdG | DcbrA vs WT | -2.14172 | 6.55E-16 |
| PA14_33280 | adaptation_and_protection | PGD1656220 | pvdL | DcbrA vs WT | -2.04891 | 4.13E-13 |
| PA14_33310 | hypothetical_unclassified | PGD1656226 | NA | DcbrA vs WT | -1.12194 | 5.64E-07 |
| PA14_33420 | putative_enzymes | PGD1656244 | NA | DcbrA vs WT | 1.744176 | 3.44E-05 |
| PA14_33430 | DNA_replication_recombinat | PGD1656246 | NA | DcbrA vs WT | 1.948451 | 2.58E-10 |
| PA14_33460 | membrane_proteins | PGD1656252 | NA | DcbrA vs WT | 1.511608 | 7.39E-10 |
| PA14_33500 | adaptation_and_protection | PGD1656256 | pvdH | DcbrA vs WT | -1.20198 | 4.69E-04 |
| PA14_33500 | transport_of_small_molecule | PGD1656256 | pvdH | DcbrA vs WT | -1.20198 | 4.69E-04 |
| PA14_33510 | antibiotic_resistance_and_su | PGD1656258 | NA | DcbrA vs WT | -1.11969 | 2.73E-04 |
| PA14_33520 | adaptation_and_protection | PGD1656260 | NA | DcbrA vs WT | -1.55537 | 3.30E-08 |

| | | | | | | |
|---|---|---|---|---|---|---|
| PA14_33520 | biosynthesis_of_cofactors | PGD1656260 | NA | DcbrA vs WT | -1.55537 | 3.30E-08 |
| PA14_33520 | putative_enzymes | PGD1656260 | NA | DcbrA vs WT | -1.55537 | 3.30E-08 |
| PA14_33540 | membrane_proteins | PGD1656264 | NA | DcbrA vs WT | -1.79273 | 1.80E-16 |
| PA14_33540 | transport_of_small_molecule | PGD1656264 | NA | DcbrA vs WT | -1.79273 | 1.80E-16 |
| PA14_33550 | transport_of_small_molecule | PGD1656266 | NA | DcbrA vs WT | -3.31572 | 2.00E-28 |
| PA14_33560 | motility_and_attachment | PGD1656268 | NA | DcbrA vs WT | -1.16753 | 1.62E-07 |
| PA14_33570 | hypothetical_unclassified | PGD1656270 | NA | DcbrA vs WT | -1.99168 | 1.36E-08 |
| PA14_33580 | hypothetical_unclassified | PGD1656272 | NA | DcbrA vs WT | -1.76031 | 1.70E-07 |
| PA14_33690 | adaptation_and_protection | PGD1656286 | pvdE | DcbrA vs WT | -1.13945 | 4.13E-04 |
| PA14_33690 | membrane_proteins | PGD1656286 | pvdE | DcbrA vs WT | -1.13945 | 4.13E-04 |
| PA14_33690 | transport_of_small_molecule | PGD1656286 | pvdE | DcbrA vs WT | -1.13945 | 4.13E-04 |
| PA14_33710 | adaptation_and_protection | PGD1656290 | pvdO | DcbrA vs WT | -2.38384 | 1.76E-10 |
| PA14_33720 | adaptation_and_protection | PGD1656292 | pvdN | DcbrA vs WT | -1.97732 | 5.80E-09 |
| PA14_33720 | transport_of_small_molecule | PGD1656292 | pvdN | DcbrA vs WT | -1.97732 | 5.80E-09 |
| PA14_33730 | central_intermediary_metab | PGD1656294 | NA | DcbrA vs WT | -1.12657 | 0.001091 |
| PA14_33740 | adaptation_and_protection | PGD1656296 | pvdP | DcbrA vs WT | -1.31114 | 4.01E-05 |
| PA14_33750 | transport_of_small_molecule | PGD1656298 | NA | DcbrA vs WT | -2.25159 | 3.39E-11 |
| PA14_33760 | membrane_proteins | PGD1656300 | NA | DcbrA vs WT | -2.19799 | 5.28E-15 |
| PA14_33760 | transport_of_small_molecule | PGD1656300 | NA | DcbrA vs WT | -2.19799 | 5.28E-15 |
| PA14_33770 | protein_secretion_export | PGD1656302 | NA | DcbrA vs WT | -1.69505 | 1.25E-09 |
| PA14_33810 | biosynthesis_of_cofactors | PGD1656308 | pvdA | DcbrA vs WT | -1.30083 | 3.09E-05 |
| PA14_33820 | adaptation_and_protection | PGD1656310 | pvdQ | DcbrA vs WT | -2.07808 | 3.62E-11 |
| PA14_33820 | antibiotic_resistance_and_su | PGD1656310 | pvdQ | DcbrA vs WT | -2.07808 | 3.62E-11 |
| PA14_33840 | transcriptional_regulators | PGD1656314 | NA | DcbrA vs WT | -1.32962 | 5.98E-08 |
| PA14_33870 | hypothetical_unclassified | PGD1656318 | NA | DcbrA vs WT | -1.56997 | 4.46E-20 |
| PA14_33920 | transcriptional_regulators | PGD1656328 | NA | DcbrA vs WT | -1.53311 | 1.50E-05 |
| PA14_33930 | membrane_proteins | PGD1656330 | NA | DcbrA vs WT | -1.29799 | 2.95E-07 |
| PA14_33990 | translation_posttranslational | PGD1656340 | clpV3 | DcbrA vs WT | -1.3244 | 5.06E-11 |
| PA14_34000 | hypothetical_unclassified | PGD1656342 | hsiH3 | DcbrA vs WT | -2.22315 | 5.43E-11 |
| PA14_34010 | membrane_proteins | PGD1656344 | hsiG3 | DcbrA vs WT | -1.91562 | 2.10E-13 |
| PA14_34020 | hypothetical_unclassified | PGD1656346 | hsiF3 | DcbrA vs WT | -2.73947 | 1.66E-11 |
| PA14_34050 | hypothetical_unclassified | PGD1656350 | hsiC3 | DcbrA vs WT | -1.64834 | 3.36E-14 |
| PA14_34110 | hypothetical_unclassified | PGD1656358 | dotU3 | DcbrA vs WT | -1.63696 | 8.99E-19 |
| PA14_34130 | hypothetical_unclassified | PGD1656360 | icmF3 | DcbrA vs WT | -1.75696 | 7.17E-27 |
| PA14_34140 | hypothetical_unclassified | PGD1656362 | hsiA3 | DcbrA vs WT | -1.15621 | 1.68E-14 |
| PA14_34170 | hypothetical_unclassified | PGD1656366 | NA | DcbrA vs WT | -1.35552 | 0.001822 |
| PA14_34180 | carbon_compound_catabolis | PGD1656368 | msuE | DcbrA vs WT | -2.34692 | 0.044104 |
| PA14_34180 | central_intermediary_metab | PGD1656368 | msuE | DcbrA vs WT | -2.34692 | 0.044104 |
| PA14_34300 | putative_enzymes | PGD1656388 | NA | DcbrA vs WT | -2.15675 | 0.007676 |
| PA14_34340 | carbon_compound_catabolis | PGD1656394 | mtlZ | DcbrA vs WT | -2.10125 | 0.002737 |
| PA14_34350 | carbon_compound_catabolis | PGD1656396 | mtlY | DcbrA vs WT | -3.53393 | 0.003712 |
| PA14_34360 | carbon_compound_catabolis | PGD1656398 | mtlD | DcbrA vs WT | -2.28014 | 6.05E-04 |
| PA14_34390 | transport_of_small_molecule | PGD1656402 | NA | DcbrA vs WT | -1.50142 | 0.00703 |
| PA14_34410 | transport_of_small_molecule | PGD1656404 | NA | DcbrA vs WT | -1.93562 | 6.85E-06 |
| PA14_34420 | transport_of_small_molecule | PGD1656406 | NA | DcbrA vs WT | -1.53278 | 5.50E-07 |
| PA14_34440 | transcriptional_regulators | PGD1656408 | mtlR | DcbrA vs WT | 1.052366 | 1.26E-07 |
| PA14_34460 | adaptation_and_protection | PGD1656412 | NA | DcbrA vs WT | -1.05986 | 2.49E-08 |
| PA14_34490 | fatty_acid_and_phospholipid | PGD1656414 | NA | DcbrA vs WT | -2.90986 | 4.00E-21 |
| PA14_34500 | transport_of_small_molecule | PGD1656416 | NA | DcbrA vs WT | -2.3489 | 1.58E-15 |
| PA14_34510 | transport_of_small_molecule | PGD1656418 | NA | DcbrA vs WT | -1.71599 | 1.22E-16 |

| | | | | | | |
|---|---|---|---|---|---|---|
| PA14_34520 | transport_of_small_molecule | PGD1656420 | NA | DcbrA vs WT | -1.27014 | 2.83E-10 |
| PA14_34540 | putative_enzymes | PGD1656422 | NA | DcbrA vs WT | -1.17323 | 4.01E-07 |
| PA14_34640 | carbon_compound_catabolis | PGD1656434 | NA | DcbrA vs WT | 1.849141 | 2.31E-17 |
| PA14_34640 | energy_metabolism_CAP | PGD1656434 | NA | DcbrA vs WT | 1.849141 | 2.31E-17 |
| PA14_34710 | transport_of_small_molecule | PGD1656446 | NA | DcbrA vs WT | -1.87017 | 0.0165 |
| PA14_34730 | transcriptional_regulators | PGD1656450 | NA | DcbrA vs WT | -1.13102 | 0.037363 |
| PA14_34740 | hypothetical_unclassified | PGD1656452 | NA | DcbrA vs WT | -1.70952 | 0.005529 |
| PA14_34780 | transport_of_small_molecule | PGD1656458 | NA | DcbrA vs WT | -4.16974 | 4.60E-04 |
| PA14_34790 | membrane_proteins | PGD1656460 | NA | DcbrA vs WT | 1.075988 | 0.007619 |
| PA14_34790 | transport_of_small_molecule | PGD1656460 | NA | DcbrA vs WT | 1.075988 | 0.007619 |
| PA14_34810 | fatty_acid_and_phospholipid | PGD1656464 | NA | DcbrA vs WT | -1.43742 | 5.27E-14 |
| PA14_34810 | putative_enzymes | PGD1656464 | NA | DcbrA vs WT | -1.43742 | 5.27E-14 |
| PA14_34810 | secreted_factors | PGD1656464 | NA | DcbrA vs WT | -1.43742 | 5.27E-14 |
| PA14_34820 | transcriptional_regulators | PGD1656466 | NA | DcbrA vs WT | -1.06947 | 1.97E-08 |
| PA14_34970 | carbon_compound_catabolis | PGD1656488 | gcd | DcbrA vs WT | -1.40364 | 5.53E-19 |
| PA14_35000 | hypothetical_unclassified | PGD1656492 | NA | DcbrA vs WT | -1.56477 | 1.18E-09 |
| PA14_35160 | hypothetical_unclassified | PGD1656520 | NA | DcbrA vs WT | -1.25675 | 1.22E-05 |
| PA14_35200 | putative_enzymes | PGD1656526 | NA | DcbrA vs WT | -1.45631 | 7.60E-05 |
| PA14_35320 | energy_metabolism_CAP | PGD1656542 | NA | DcbrA vs WT | -1.30871 | 0.009798 |
| PA14_35330 | membrane_proteins | PGD1656544 | NA | DcbrA vs WT | 1.384915 | 6.91E-05 |
| PA14_35330 | transport_of_small_molecule | PGD1656544 | NA | DcbrA vs WT | 1.384915 | 6.91E-05 |
| PA14_35360 | carbon_compound_catabolis | PGD1656548 | NA | DcbrA vs WT | -2.90519 | 1.51E-04 |
| PA14_35390 | amino_acid_biosynthesis_me | PGD1656554 | pvcD | DcbrA vs WT | -1.87276 | 0.004616 |
| PA14_35390 | secreted_factors | PGD1656554 | pvcD | DcbrA vs WT | -1.87276 | 0.004616 |
| PA14_35440 | amino_acid_biosynthesis_me | PGD1656562 | ansA | DcbrA vs WT | -2.35791 | 6.56E-06 |
| PA14_35490 | amino_acid_biosynthesis_me | PGD1656568 | lpdV | DcbrA vs WT | -1.85635 | 1.86E-29 |
| PA14_35490 | energy_metabolism_CAP | PGD1656568 | lpdV | DcbrA vs WT | -1.85635 | 1.86E-29 |
| PA14_35500 | amino_acid_biosynthesis_me | PGD1656570 | bkdB | DcbrA vs WT | -1.02013 | 1.00E-12 |
| PA14_35520 | amino_acid_biosynthesis_me | PGD1656572 | bkdA2 | DcbrA vs WT | -1.32759 | 7.19E-16 |
| PA14_35530 | amino_acid_biosynthesis_me | PGD1656574 | bkdA1 | DcbrA vs WT | -1.14163 | 4.66E-12 |
| PA14_35550 | hypothetical_unclassified | PGD1656578 | pslO | DcbrA vs WT | 2.654636 | 2.86E-25 |
| PA14_35620 | cell_wall_LPS | PGD1656586 | pslK | DcbrA vs WT | -1.67853 | 3.62E-07 |
| PA14_35620 | membrane_proteins | PGD1656586 | pslK | DcbrA vs WT | -1.67853 | 3.62E-07 |
| PA14_35640 | cell_wall_LPS | PGD1656590 | pslI | DcbrA vs WT | -1.20065 | 0.002 |
| PA14_35640 | putative_enzymes | PGD1656590 | pslI | DcbrA vs WT | -1.20065 | 0.002 |
| PA14_35650 | cell_wall_LPS | PGD1656592 | pslH | DcbrA vs WT | -1.47382 | 1.11E-05 |
| PA14_35680 | hypothetical_unclassified | PGD1656596 | pslF | DcbrA vs WT | -1.88612 | 7.45E-05 |
| PA14_35700 | hypothetical_unclassified | PGD1656600 | NA | DcbrA vs WT | 1.117818 | 8.21E-04 |
| PA14_35860 | transport_of_small_molecule | PGD1656632 | NA | DcbrA vs WT | 1.339857 | 3.09E-12 |
| PA14_36070 | putative_enzymes | PGD1656666 | NA | DcbrA vs WT | 1.09502 | 0.002461 |
| PA14_36110 | putative_enzymes | PGD1656674 | NA | DcbrA vs WT | -2.12324 | 0.001081 |
| PA14_36200 | transport_of_small_molecule | PGD1656688 | NA | DcbrA vs WT | -1.02249 | 0.004579 |
| PA14_36220 | membrane_proteins | PGD1656690 | NA | DcbrA vs WT | -1.77504 | 0.010012 |
| PA14_36220 | transport_of_small_molecule | PGD1656690 | NA | DcbrA vs WT | -1.77504 | 0.010012 |
| PA14_36230 | membrane_proteins | PGD1656692 | NA | DcbrA vs WT | -2.00881 | 3.81E-04 |
| PA14_36230 | transport_of_small_molecule | PGD1656692 | NA | DcbrA vs WT | -2.00881 | 3.81E-04 |
| PA14_36280 | antibiotic_resistance_and_su | PGD1656700 | NA | DcbrA vs WT | -1.84265 | 0.016586 |
| PA14_36310 | central_intermediary_metab | PGD1656706 | hcnC | DcbrA vs WT | -1.41502 | 1.97E-12 |
| PA14_36320 | central_intermediary_metab | PGD1656708 | hcnB | DcbrA vs WT | -2.16646 | 4.34E-18 |
| PA14_36330 | central_intermediary_metab | PGD1656710 | hcnA | DcbrA vs WT | -1.51491 | 2.15E-13 |

| | | | | | | |
|---|---|---|---|---|---|---|
| PA14_36350 | hypothetical_unclassified | PGD1656714 | NA | DcbrA vs WT | 1.801311 | 1.16E-15 |
| PA14_36360 | membrane_proteins | PGD1656716 | NA | DcbrA vs WT | 2.102945 | 3.01E-15 |
| PA14_36370 | amino_acid_biosynthesis_me | PGD1656718 | NA | DcbrA vs WT | 1.598117 | 1.16E-07 |
| PA14_36375 | hypothetical_unclassified | PGD1656720 | NA | DcbrA vs WT | 2.639712 | 2.35E-17 |
| PA14_36390 | translation_posttranslational | PGD1656722 | NA | DcbrA vs WT | 1.460709 | 8.26E-07 |
| PA14_36400 | hypothetical_unclassified | PGD1656724 | NA | DcbrA vs WT | 1.108923 | 0.00605 |
| PA14_36410 | hypothetical_unclassified | PGD1656726 | NA | DcbrA vs WT | 1.794422 | 2.51E-15 |
| PA14_36460 | hypothetical_unclassified | PGD1656732 | NA | DcbrA vs WT | 2.721705 | 8.36E-54 |
| PA14_36480 | hypothetical_unclassified | PGD1656736 | NA | DcbrA vs WT | 2.459332 | 4.30E-13 |
| PA14_36500 | carbon_compound_catabolis | PGD1656740 | NA | DcbrA vs WT | 1.786905 | 4.37E-13 |
| PA14_36520 | hypothetical_unclassified | PGD1656742 | NA | DcbrA vs WT | 2.492399 | 1.14E-39 |
| PA14_36530 | hypothetical_unclassified | PGD1656744 | NA | DcbrA vs WT | 1.036848 | 1.93E-06 |
| PA14_36550 | hypothetical_unclassified | PGD1656748 | NA | DcbrA vs WT | 2.243964 | 1.66E-33 |
| PA14_36560 | hypothetical_unclassified | PGD1656750 | NA | DcbrA vs WT | 1.293417 | 1.29E-06 |
| PA14_36570 | carbon_compound_catabolis | PGD1656752 | glgA | DcbrA vs WT | 2.11559 | 9.84E-30 |
| PA14_36570 | energy_metabolism_CAP | PGD1656752 | glgA | DcbrA vs WT | 2.11559 | 9.84E-30 |
| PA14_36580 | putative_enzymes | PGD1656754 | NA | DcbrA vs WT | 1.406895 | 6.25E-08 |
| PA14_36605 | putative_enzymes | PGD1656758 | NA | DcbrA vs WT | 2.236078 | 3.93E-10 |
| PA14_36620 | hypothetical_unclassified | PGD1656760 | NA | DcbrA vs WT | 3.196626 | 0.027344 |
| PA14_36630 | carbon_compound_catabolis | PGD1656762 | NA | DcbrA vs WT | 1.877514 | 1.30E-10 |
| PA14_36630 | putative_enzymes | PGD1656762 | NA | DcbrA vs WT | 1.877514 | 1.30E-10 |
| PA14_36660 | amino_acid_biosynthesis_me | PGD1656766 | NA | DcbrA vs WT | 1.708172 | 3.76E-13 |
| PA14_36670 | hypothetical_unclassified | PGD1656768 | NA | DcbrA vs WT | 2.793765 | 1.35E-16 |
| PA14_36680 | putative_enzymes | PGD1656770 | NA | DcbrA vs WT | 2.350374 | 9.15E-11 |
| PA14_36690 | fatty_acid_and_phospholipid | PGD1656772 | NA | DcbrA vs WT | 2.759768 | 5.24E-18 |
| PA14_36690 | putative_enzymes | PGD1656772 | NA | DcbrA vs WT | 2.759768 | 5.24E-18 |
| PA14_36700 | membrane_proteins | PGD1656774 | NA | DcbrA vs WT | 3.405827 | 1.64E-24 |
| PA14_36710 | energy_metabolism_CAP | PGD1656776 | glgB | DcbrA vs WT | 2.56797 | 1.88E-09 |
| PA14_36730 | carbon_compound_catabolis | PGD1656778 | NA | DcbrA vs WT | 2.076491 | 1.29E-15 |
| PA14_36730 | putative_enzymes | PGD1656778 | NA | DcbrA vs WT | 2.076491 | 1.29E-15 |
| PA14_36740 | putative_enzymes | PGD1656780 | NA | DcbrA vs WT | 1.502419 | 1.46E-15 |
| PA14_36760 | DNA_replication_recombinat | PGD1656782 | NA | DcbrA vs WT | 2.224558 | 3.00E-18 |
| PA14_36770 | hypothetical_unclassified | PGD1656784 | NA | DcbrA vs WT | 1.939102 | 7.44E-08 |
| PA14_36790 | hypothetical_unclassified | PGD1656788 | NA | DcbrA vs WT | 2.570646 | 1.01E-25 |
| PA14_36810 | adaptation_and_protection | PGD1656790 | katE | DcbrA vs WT | 2.261634 | 2.16E-16 |
| PA14_36820 | hypothetical_unclassified | PGD1656792 | NA | DcbrA vs WT | 2.766719 | 1.21E-12 |
| PA14_36830 | hypothetical_unclassified | PGD1656794 | NA | DcbrA vs WT | 1.522128 | 1.27E-11 |
| PA14_36840 | cell_wall_LPS | PGD1656796 | glgP | DcbrA vs WT | 1.519761 | 7.06E-11 |
| PA14_36870 | central_intermediary_metab | PGD1656802 | NA | DcbrA vs WT | 1.432527 | 9.31E-10 |
| PA14_36880 | transcriptional_regulators | PGD1656804 | NA | DcbrA vs WT | 1.913784 | 1.09E-06 |
| PA14_36900 | hypothetical_unclassified | PGD1656808 | NA | DcbrA vs WT | 1.859942 | 1.53E-08 |
| PA14_36910 | DNA_replication_recombinat | PGD1656810 | ligD | DcbrA vs WT | 1.073593 | 5.14E-08 |
| PA14_36920 | two_component_regulators | PGD1656812 | NA | DcbrA vs WT | 1.894349 | 8.21E-19 |
| PA14_36940 | membrane_proteins | PGD1656816 | NA | DcbrA vs WT | 1.74841 | 9.15E-15 |
| PA14_36980 | membrane_proteins | PGD1656820 | NA | DcbrA vs WT | 2.350841 | 1.17E-24 |
| PA14_36990 | two_component_regulators | PGD1656822 | NA | DcbrA vs WT | -1.23315 | 0.040681 |
| PA14_37000 | chaperones_heat_shock | PGD1656824 | cupA5 | DcbrA vs WT | -3.46092 | 0.020223 |
| PA14_37080 | transcriptional_regulators | PGD1656836 | NA | DcbrA vs WT | -2.21174 | 1.68E-10 |
| PA14_37140 | transcriptional_regulators | PGD1656846 | NA | DcbrA vs WT | -1.59549 | 3.53E-06 |
| PA14_37170 | hypothetical_unclassified | PGD1656850 | NA | DcbrA vs WT | -1.215 | 5.63E-06 |

| | | | | | | |
|---|---|---|---|---|---|---|
| PA14_37210 | hypothetical_unclassified | PGD1656856 | NA | DcbrA vs WT | 1.128986 | 4.55E-06 |
| PA14_37250 | transport_of_small_molecule | PGD1656860 | NA | DcbrA vs WT | 2.130451 | 1.07E-23 |
| PA14_37270 | putative_enzymes | PGD1656864 | NA | DcbrA vs WT | -1.21113 | 1.94E-04 |
| PA14_37350 | hypothetical_unclassified | PGD1656874 | NA | DcbrA vs WT | 1.029723 | 0.002837 |
| PA14_37360 | central_intermediary_metab | PGD1656876 | NA | DcbrA vs WT | -2.12723 | 1.54E-12 |
| PA14_37370 | fatty_acid_and_phospholipid | PGD1656878 | NA | DcbrA vs WT | -4.42974 | 3.06E-08 |
| PA14_37380 | transport_of_small_molecule | PGD1656880 | NA | DcbrA vs WT | -1.30259 | 5.80E-09 |
| PA14_37400 | transcriptional_regulators | PGD1656882 | NA | DcbrA vs WT | -1.13246 | 0.028723 |
| PA14_37410 | hypothetical_unclassified | PGD1656884 | NA | DcbrA vs WT | -1.23708 | 3.00E-04 |
| PA14_37440 | membrane_proteins | PGD1656890 | NA | DcbrA vs WT | -1.33653 | 0.001347 |
| PA14_37440 | transport_of_small_molecule | PGD1656890 | NA | DcbrA vs WT | -1.33653 | 0.001347 |
| PA14_37460 | transport_of_small_molecule | PGD1656892 | NA | DcbrA vs WT | -1.28115 | 0.01648 |
| PA14_37510 | hypothetical_unclassified | PGD1656898 | NA | DcbrA vs WT | -4.78037 | 3.34E-04 |
| PA14_37530 | putative_enzymes | PGD1656902 | NA | DcbrA vs WT | -2.96305 | 2.63E-04 |
| PA14_37560 | amino_acid_biosynthesis_me | PGD1656906 | NA | DcbrA vs WT | -2.05073 | 4.99E-05 |
| PA14_37590 | amino_acid_biosynthesis_me | PGD1656912 | kynB | DcbrA vs WT | -1.14326 | 4.51E-07 |
| PA14_37590 | putative_enzymes | PGD1656912 | kynB | DcbrA vs WT | -1.14326 | 4.51E-07 |
| PA14_37630 | transport_of_small_molecule | PGD1656916 | NA | DcbrA vs WT | -1.33716 | 7.89E-06 |
| PA14_37770 | carbon_compound_catabolis | PGD1656938 | NA | DcbrA vs WT | -1.48633 | 5.47E-07 |
| PA14_37980 | transport_of_small_molecule | PGD1656970 | NA | DcbrA vs WT | -3.5619 | 5.80E-04 |
| PA14_37990 | transcriptional_regulators | PGD1656972 | NA | DcbrA vs WT | -2.13326 | 0.005529 |
| PA14_38020 | antibiotic_resistance_and_su | PGD1656978 | NA | DcbrA vs WT | -1.37728 | 4.92E-05 |
| PA14_38090 | translation_posttranslational | PGD1656988 | NA | DcbrA vs WT | -1.1566 | 6.80E-07 |
| PA14_38210 | central_intermediary_metab | PGD1657006 | NA | DcbrA vs WT | -1.31682 | 0.0088 |
| PA14_38260 | hypothetical_unclassified | PGD1657012 | NA | DcbrA vs WT | -1.72402 | 2.98E-09 |
| PA14_38350 | central_intermediary_metab | PGD1657028 | galU | DcbrA vs WT | 1.591571 | 1.21E-53 |
| PA14_38360 | cell_wall_LPS | PGD1657030 | NA | DcbrA vs WT | 1.824882 | 2.82E-20 |
| PA14_38360 | putative_enzymes | PGD1657030 | NA | DcbrA vs WT | 1.824882 | 2.82E-20 |
| PA14_38460 | carbon_compound_catabolis | PGD1657046 | gnyB | DcbrA vs WT | -1.03645 | 9.34E-10 |
| PA14_38480 | carbon_compound_catabolis | PGD1657050 | gnyA | DcbrA vs WT | -1.34888 | 1.54E-13 |
| PA14_38530 | carbon_compound_catabolis | PGD1657058 | fahA | DcbrA vs WT | -1.08564 | 1.75E-04 |
| PA14_38550 | carbon_compound_catabolis | PGD1657060 | maiA | DcbrA vs WT | -1.08447 | 2.73E-05 |
| PA14_38590 | carbon_compound_catabolis | PGD1657068 | bdhA | DcbrA vs WT | -2.59747 | 3.37E-36 |
| PA14_38640 | fatty_acid_and_phospholipid | PGD1657074 | NA | DcbrA vs WT | 1.258279 | 9.75E-16 |
| PA14_38660 | fatty_acid_and_phospholipid | PGD1657076 | NA | DcbrA vs WT | 2.149513 | 4.10E-52 |
| PA14_38740 | two_component_regulators | PGD1657090 | NA | DcbrA vs WT | -1.75717 | 1.21E-14 |
| PA14_38950 | membrane_proteins | PGD1657122 | NA | DcbrA vs WT | -1.27045 | 0.017006 |
| PA14_39010 | biosynthesis_of_cofactors | PGD1657130 | pqqF | DcbrA vs WT | -1.30259 | 1.72E-04 |
| PA14_39060 | fatty_acid_and_phospholipid | PGD1657136 | NA | DcbrA vs WT | 3.140504 | 2.22E-10 |
| PA14_39080 | hypothetical_unclassified | PGD1657140 | NA | DcbrA vs WT | -3.09314 | 1.03E-08 |
| PA14_39090 | hypothetical_unclassified | PGD1657142 | NA | DcbrA vs WT | -1.37011 | 3.05E-08 |
| PA14_39150 | fatty_acid_and_phospholipid | PGD1657152 | NA | DcbrA vs WT | 1.446463 | 0.002014 |
| PA14_39160 | transcriptional_regulators | PGD1657154 | NA | DcbrA vs WT | -1.56349 | 7.42E-07 |
| PA14_39180 | membrane_proteins | PGD1657156 | NA | DcbrA vs WT | -2.41045 | 5.76E-09 |
| PA14_39210 | biosynthesis_of_cofactors | PGD1657162 | NA | DcbrA vs WT | -1.63657 | 2.62E-05 |
| PA14_39220 | hypothetical_unclassified | PGD1657164 | NA | DcbrA vs WT | 2.375328 | 0.025423 |
| PA14_39240 | hypothetical_unclassified | PGD1657168 | fapC | DcbrA vs WT | 2.081671 | 1.25E-06 |
| PA14_39280 | carbon_compound_catabolis | PGD1657176 | rbsK | DcbrA vs WT | -2.17286 | 8.31E-22 |
| PA14_39300 | carbon_compound_catabolis | PGD1657178 | rbsR | DcbrA vs WT | -1.69398 | 9.24E-14 |
| PA14_39330 | transport_of_small_molecule | PGD1657182 | rbsA | DcbrA vs WT | -2.43402 | 1.57E-17 |

| | | | | | | |
|---|---|---|---|---|---|---|
| PA14_39410 | transport_of_small_molecule | PGD1657190 | NA | DcbrA vs WT | -1.25724 | 4.47E-13 |
| PA14_39420 | hypothetical_unclassified | PGD1657192 | NA | DcbrA vs WT | 2.635797 | 1.90E-12 |
| PA14_39520 | energy_metabolism_CAP | PGD1657204 | NA | DcbrA vs WT | 1.149132 | 9.10E-10 |
| PA14_39700 | hypothetical_unclassified | PGD1657234 | NA | DcbrA vs WT | -1.78799 | 4.37E-04 |
| PA14_39710 | nucleotide_biosynthesis_and | PGD1657236 | NA | DcbrA vs WT | -1.38711 | 0.006037 |
| PA14_39710 | translation_posttranslational | PGD1657236 | NA | DcbrA vs WT | -1.38711 | 0.006037 |
| PA14_39780 | transport_of_small_molecule | PGD1657246 | NA | DcbrA vs WT | -3.02751 | 2.03E-31 |
| PA14_39790 | hypothetical_unclassified | PGD1657248 | NA | DcbrA vs WT | -1.69296 | 0.002408 |
| PA14_39800 | transcriptional_regulators | PGD1657250 | NA | DcbrA vs WT | 1.294382 | 0.002622 |
| PA14_39880 | secreted_factors | PGD1657264 | phzG2 | DcbrA vs WT | -1.408 | 0.007781 |
| PA14_39945 | secreted_factors | PGD1657272 | phzC2 | DcbrA vs WT | -2.94435 | 4.33E-07 |
| PA14_39990 | fatty_acid_and_phospholipid | PGD1657280 | NA | DcbrA vs WT | -1.52328 | 2.81E-09 |
| PA14_40010 | hypothetical_unclassified | PGD1657282 | NA | DcbrA vs WT | -2.22609 | 8.48E-27 |
| PA14_40020 | hypothetical_unclassified | PGD1657284 | NA | DcbrA vs WT | -2.21285 | 1.91E-25 |
| PA14_40030 | putative_enzymes | PGD1657286 | NA | DcbrA vs WT | -1.18982 | 1.48E-07 |
| PA14_40040 | central_intermediary_metab | PGD1657288 | NA | DcbrA vs WT | -2.30723 | 2.03E-30 |
| PA14_40040 | putative_enzymes | PGD1657288 | NA | DcbrA vs WT | -2.30723 | 2.03E-30 |
| PA14_40050 | hypothetical_unclassified | PGD1657290 | NA | DcbrA vs WT | -1.39301 | 9.07E-12 |
| PA14_40060 | fatty_acid_and_phospholipid | PGD1657292 | NA | DcbrA vs WT | -3.85629 | 7.63E-16 |
| PA14_40060 | membrane_proteins | PGD1657292 | NA | DcbrA vs WT | -3.85629 | 7.63E-16 |
| PA14_40130 | transport_of_small_molecule | PGD1657304 | NA | DcbrA vs WT | -2.31377 | 6.15E-14 |
| PA14_40200 | putative_enzymes | PGD1657314 | NA | DcbrA vs WT | -1.0575 | 2.09E-06 |
| PA14_40230 | protein_secretion_export | PGD1657320 | NA | DcbrA vs WT | -1.08865 | 5.43E-07 |
| PA14_40240 | protein_secretion_export | PGD1657322 | NA | DcbrA vs WT | -1.92081 | 4.55E-15 |
| PA14_40250 | membrane_proteins | PGD1657324 | NA | DcbrA vs WT | -1.82296 | 9.19E-21 |
| PA14_40260 | hypothetical_unclassified | PGD1657326 | NA | DcbrA vs WT | -2.49133 | 1.35E-27 |
| PA14_40290 | secreted_factors | PGD1657332 | lasA | DcbrA vs WT | -1.49923 | 1.80E-14 |
| PA14_40290 | translation_posttranslational | PGD1657332 | lasA | DcbrA vs WT | -1.49923 | 1.80E-14 |
| PA14_40340 | hypothetical_unclassified | PGD1657342 | NA | DcbrA vs WT | -1.55093 | 1.46E-08 |
| PA14_40380 | transcriptional_regulators | PGD1657348 | NA | DcbrA vs WT | -1.51336 | 0.004739 |
| PA14_40450 | antibiotic_resistance_and_su | PGD1657360 | str | DcbrA vs WT | -1.20648 | 1.04E-05 |
| PA14_40520 | hypothetical_unclassified | PGD1657368 | NA | DcbrA vs WT | -4.22444 | 0.002307 |
| PA14_40660 | secreted_factors | PGD1657390 | NA | DcbrA vs WT | -2.13528 | 9.07E-08 |
| PA14_40780 | hypothetical_unclassified | PGD1657408 | NA | DcbrA vs WT | -2.48809 | 8.92E-30 |
| PA14_40800 | fatty_acid_and_phospholipid | PGD1657412 | NA | DcbrA vs WT | 1.135438 | 2.89E-06 |
| PA14_40940 | membrane_proteins | PGD1657434 | NA | DcbrA vs WT | -1.23451 | 2.64E-05 |
| PA14_40980 | fatty_acid_and_phospholipid | PGD1657440 | NA | DcbrA vs WT | 1.041526 | 7.28E-13 |
| PA14_40980 | putative_enzymes | PGD1657440 | NA | DcbrA vs WT | 1.041526 | 7.28E-13 |
| PA14_41110 | transport_of_small_molecule | PGD1657460 | nppA1 | DcbrA vs WT | 1.042974 | 2.55E-12 |
| PA14_41130 | transport_of_small_molecule | PGD1657462 | nppA2 | DcbrA vs WT | 1.36789 | 1.90E-28 |
| PA14_41150 | transport_of_small_molecule | PGD1657466 | nppC | DcbrA vs WT | 1.003431 | 1.49E-04 |
| PA14_41230 | cell_wall_LPS | PGD1657480 | clpX | DcbrA vs WT | 1.048155 | 2.76E-07 |
| PA14_41230 | chaperones_heat_shock | PGD1657480 | clpX | DcbrA vs WT | 1.048155 | 2.76E-07 |
| PA14_41240 | antibiotic_resistance_and_su | PGD1657482 | clpP | DcbrA vs WT | 1.252751 | 2.01E-22 |
| PA14_41240 | cell_wall_LPS | PGD1657482 | clpP | DcbrA vs WT | 1.252751 | 2.01E-22 |
| PA14_41240 | chaperones_heat_shock | PGD1657482 | clpP | DcbrA vs WT | 1.252751 | 2.01E-22 |
| PA14_41240 | motility_and_attachment | PGD1657482 | clpP | DcbrA vs WT | 1.252751 | 2.01E-22 |
| PA14_41520 | adaptation_and_protection | PGD1657530 | NA | DcbrA vs WT | -1.30363 | 0.004431 |
| PA14_41575 | transcriptional_regulators | PGD1657542 | sigX | DcbrA vs WT | 1.147772 | 1.70E-11 |
| PA14_41650 | fatty_acid_and_phospholipid | PGD1657552 | NA | DcbrA vs WT | -1.21139 | 0.002123 |

| | | | | | | |
|---|---|---|---|---|---|---|
| PA14_41710 | membrane_proteins | PGD1657560 | NA | DcbrA vs WT | 1.026738 | 7.08E-09 |
| PA14_41900 | central_intermediary_metab | PGD1657590 | panE | DcbrA vs WT | 1.393295 | 4.49E-25 |
| PA14_41930 | transport_of_small_molecule | PGD1657596 | NA | DcbrA vs WT | 1.044284 | 5.59E-15 |
| PA14_41970 | hypothetical_unclassified | PGD1657602 | NA | DcbrA vs WT | -2.09343 | 1.86E-16 |
| PA14_41990 | hypothetical_unclassified | PGD1657606 | NA | DcbrA vs WT | 2.463506 | 8.44E-05 |
| PA14_42150 | hypothetical_unclassified | PGD1657632 | NA | DcbrA vs WT | 1.282636 | 1.25E-10 |
| PA14_42160 | hypothetical_unclassified | PGD1657634 | NA | DcbrA vs WT | 1.271153 | 3.84E-17 |
| PA14_42260 | protein_secretion_export | PGD1657646 | pscK | DcbrA vs WT | -3.67601 | 0.018678 |
| PA14_42260 | secreted_factors | PGD1657646 | pscK | DcbrA vs WT | -3.67601 | 0.018678 |
| PA14_42270 | secreted_factors | PGD1657648 | pscJ | DcbrA vs WT | 1.436126 | 4.98E-04 |
| PA14_42400 | protein_secretion_export | PGD1657670 | exsB | DcbrA vs WT | 1.030999 | 0.029705 |
| PA14_42400 | translation_posttranslational | PGD1657670 | exsB | DcbrA vs WT | 1.030999 | 0.029705 |
| PA14_42430 | protein_secretion_export | PGD1657674 | exsC | DcbrA vs WT | 1.919843 | 3.39E-13 |
| PA14_42430 | secreted_factors | PGD1657674 | exsC | DcbrA vs WT | 1.919843 | 3.39E-13 |
| PA14_42430 | translation_posttranslational | PGD1657674 | exsC | DcbrA vs WT | 1.919843 | 3.39E-13 |
| PA14_42440 | protein_secretion_export | PGD1657676 | popD | DcbrA vs WT | -1.44095 | 0.033659 |
| PA14_42470 | protein_secretion_export | PGD1657682 | pcrV | DcbrA vs WT | 1.357104 | 0.001225 |
| PA14_42630 | protein_secretion_export | PGD1657710 | NA | DcbrA vs WT | 1.518782 | 0.024462 |
| PA14_42700 | DNA_replication_recombinat | PGD1657722 | alkA | DcbrA vs WT | -1.07874 | 1.69E-08 |
| PA14_42730 | amino_acid_biosynthesis_me | PGD1657728 | NA | DcbrA vs WT | -1.10282 | 5.53E-06 |
| PA14_42730 | central_intermediary_metab | PGD1657728 | NA | DcbrA vs WT | -1.10282 | 5.53E-06 |
| PA14_42890 | protein_secretion_export | PGD1657756 | stp1 | DcbrA vs WT | -1.71391 | 7.52E-11 |
| PA14_42890 | translation_posttranslational | PGD1657756 | stp1 | DcbrA vs WT | -1.71391 | 7.52E-11 |
| PA14_42950 | hypothetical_unclassified | PGD1657766 | fha2 | DcbrA vs WT | -1.14116 | 2.71E-07 |
| PA14_42980 | chaperones_heat_shock | PGD1657772 | clpV2 | DcbrA vs WT | -1.13591 | 2.55E-12 |
| PA14_43230 | hypothetical_unclassified | PGD1657814 | NA | DcbrA vs WT | -3.91248 | 7.93E-24 |
| PA14_43250 | hypothetical_unclassified | PGD1657818 | NA | DcbrA vs WT | -1.0227 | 6.82E-09 |
| PA14_43280 | translation_posttranslational | PGD1657822 | selD | DcbrA vs WT | -1.08131 | 3.33E-11 |
| PA14_43350 | two_component_regulators | PGD1657834 | kdpD | DcbrA vs WT | -1.19961 | 1.16E-12 |
| PA14_43405 | transport_of_small_molecule | PGD1657842 | kdbF | DcbrA vs WT | 3.234299 | 0.049477 |
| PA14_43460 | fatty_acid_and_phospholipid | PGD1657850 | NA | DcbrA vs WT | -1.47924 | 0.009008 |
| PA14_43490 | transport_of_small_molecule | PGD1657854 | NA | DcbrA vs WT | -1.80373 | 6.48E-06 |
| PA14_43520 | hypothetical_unclassified | PGD1657858 | NA | DcbrA vs WT | -1.17431 | 3.75E-11 |
| PA14_43570 | transport_of_small_molecule | PGD1657866 | NA | DcbrA vs WT | -2.11184 | 0.014721 |
| PA14_43600 | fatty_acid_and_phospholipid | PGD1657870 | NA | DcbrA vs WT | -1.16201 | 5.92E-11 |
| PA14_43630 | putative_enzymes | PGD1657876 | NA | DcbrA vs WT | 1.900673 | 1.07E-16 |
| PA14_43640 | central_intermediary_metab | PGD1657878 | gpsA | DcbrA vs WT | 1.145484 | 2.02E-14 |
| PA14_43640 | fatty_acid_and_phospholipid | PGD1657878 | gpsA | DcbrA vs WT | 1.145484 | 2.02E-14 |
| PA14_43670 | two_component_regulators | PGD1657884 | NA | DcbrA vs WT | 1.161952 | 1.77E-16 |
| PA14_43680 | fatty_acid_and_phospholipid | PGD1657886 | fabA | DcbrA vs WT | 1.206831 | 1.44E-17 |
| PA14_43730 | hypothetical_unclassified | PGD1657894 | NA | DcbrA vs WT | 1.664065 | 8.20E-15 |
| PA14_43740 | putative_enzymes | PGD1657896 | NA | DcbrA vs WT | 1.156766 | 8.23E-18 |
| PA14_43760 | hypothetical_unclassified | PGD1657898 | NA | DcbrA vs WT | -1.31984 | 1.92E-07 |
| PA14_43770 | transcriptional_regulators | PGD1657900 | NA | DcbrA vs WT | -1.30441 | 1.84E-07 |
| PA14_43850 | chaperones_heat_shock | PGD1657914 | htpG | DcbrA vs WT | 1.090501 | 1.67E-11 |
| PA14_44020 | energy_metabolism_CAP | PGD1657938 | sdhB | DcbrA vs WT | 1.239635 | 1.29E-08 |
| PA14_44080 | hypothetical_unclassified | PGD1657948 | NA | DcbrA vs WT | 1.743625 | 8.81E-45 |
| PA14_44170 | hypothetical_unclassified | PGD1657966 | NA | DcbrA vs WT | 1.817259 | 9.24E-20 |
| PA14_44210 | amino_acid_biosynthesis_me | PGD1657974 | NA | DcbrA vs WT | -1.15843 | 2.40E-07 |
| PA14_44270 | translation_posttranslational | PGD1657982 | NA | DcbrA vs WT | 1.00989 | 8.29E-08 |

| | | | | | | |
|---|---|---|---|---|---|---|
| PA14_44300 | chemotaxis | PGD1657988 | aer | DcbrA vs WT | -1.70881 | 8.98E-14 |
| PA14_44440 | membrane_proteins | PGD1658012 | NA | DcbrA vs WT | -1.37999 | 1.49E-22 |
| PA14_44440 | transport_of_small_molecule | PGD1658012 | NA | DcbrA vs WT | -1.37999 | 1.49E-22 |
| PA14_44460 | membrane_proteins | PGD1658016 | NA | DcbrA vs WT | -1.84084 | 4.87E-16 |
| PA14_44490 | transcriptional_regulators | PGD1658022 | anr | DcbrA vs WT | 1.444016 | 1.17E-10 |
| PA14_44520 | membrane_proteins | PGD1658028 | NA | DcbrA vs WT | 2.078924 | 9.44E-10 |
| PA14_44520 | transport_of_small_molecule | PGD1658028 | NA | DcbrA vs WT | 2.078924 | 9.44E-10 |
| PA14_44590 | putative_enzymes | PGD1658040 | NA | DcbrA vs WT | 1.0422 | 7.25E-09 |
| PA14_44620 | hypothetical_unclassified | PGD1658044 | NA | DcbrA vs WT | 1.566915 | 6.78E-31 |
| PA14_44680 | cell_division | PGD1658056 | NA | DcbrA vs WT | 1.511149 | 1.50E-20 |
| PA14_44950 | membrane_proteins | PGD1658096 | NA | DcbrA vs WT | 2.220961 | 4.11E-32 |
| PA14_44950 | transport_of_small_molecule | PGD1658096 | NA | DcbrA vs WT | 2.220961 | 4.11E-32 |
| PA14_45010 | central_intermediary_metab | PGD1658108 | NA | DcbrA vs WT | -1.49634 | 0.003488 |
| PA14_45030 | carbon_compound_catabolis | PGD1658112 | NA | DcbrA vs WT | -1.25606 | 0.036425 |
| PA14_45030 | putative_enzymes | PGD1658112 | NA | DcbrA vs WT | -1.25606 | 0.036425 |
| PA14_45090 | hypothetical_unclassified | PGD1658120 | NA | DcbrA vs WT | -1.87842 | 2.81E-09 |
| PA14_45100 | transcriptional_regulators | PGD1658122 | NA | DcbrA vs WT | 1.106508 | 4.03E-04 |
| PA14_45100 | transport_of_small_molecule | PGD1658122 | NA | DcbrA vs WT | 1.106508 | 4.03E-04 |
| PA14_45130 | membrane_proteins | PGD1658128 | NA | DcbrA vs WT | 1.069635 | 0.002029 |
| PA14_45130 | transport_of_small_molecule | PGD1658128 | NA | DcbrA vs WT | 1.069635 | 0.002029 |
| PA14_45180 | putative_enzymes | PGD1658134 | NA | DcbrA vs WT | -1.85621 | 0.003162 |
| PA14_45190 | energy_metabolism_CAP | PGD1658136 | NA | DcbrA vs WT | -1.10885 | 5.39E-04 |
| PA14_45190 | putative_enzymes | PGD1658136 | NA | DcbrA vs WT | -1.10885 | 5.39E-04 |
| PA14_45210 | amino_acid_biosynthesis_me | PGD1658138 | NA | DcbrA vs WT | -2.19877 | 1.07E-05 |
| PA14_45240 | transport_of_small_molecule | PGD1658140 | NA | DcbrA vs WT | -1.14039 | 0.001675 |
| PA14_45280 | energy_metabolism_CAP | PGD1658146 | cycH | DcbrA vs WT | -1.64104 | 1.62E-16 |
| PA14_45310 | energy_metabolism_CAP | PGD1658152 | ccmF | DcbrA vs WT | -1.07825 | 5.36E-11 |
| PA14_45350 | transport_of_small_molecule | PGD1658158 | ccmC | DcbrA vs WT | -1.14295 | 1.76E-17 |
| PA14_45370 | membrane_proteins | PGD1658160 | ccmB | DcbrA vs WT | -2.22469 | 1.29E-42 |
| PA14_45370 | transport_of_small_molecule | PGD1658160 | ccmB | DcbrA vs WT | -2.22469 | 1.29E-42 |
| PA14_45400 | hypothetical_unclassified | PGD1658164 | NA | DcbrA vs WT | -1.0554 | 7.10E-09 |
| PA14_45480 | hypothetical_unclassified | PGD1658178 | NA | DcbrA vs WT | -1.38243 | 6.09E-08 |
| PA14_45760 | adaptation_and_protection | PGD1658214 | fliQ | DcbrA vs WT | 1.25731 | 3.66E-05 |
| PA14_45760 | chemotaxis | PGD1658214 | fliQ | DcbrA vs WT | 1.25731 | 3.66E-05 |
| PA14_45760 | motility_and_attachment | PGD1658214 | fliQ | DcbrA vs WT | 1.25731 | 3.66E-05 |
| PA14_45780 | adaptation_and_protection | PGD1658218 | fliO | DcbrA vs WT | -2.11086 | 2.42E-08 |
| PA14_45780 | chemotaxis | PGD1658218 | fliO | DcbrA vs WT | -2.11086 | 2.42E-08 |
| PA14_45780 | motility_and_attachment | PGD1658218 | fliO | DcbrA vs WT | -2.11086 | 2.42E-08 |
| PA14_45970 | membrane_proteins | PGD1658250 | NA | DcbrA vs WT | -1.58306 | 3.04E-08 |
| PA14_45970 | transport_of_small_molecule | PGD1658250 | NA | DcbrA vs WT | -1.58306 | 3.04E-08 |
| PA14_46160 | hypothetical_unclassified | PGD1658278 | NA | DcbrA vs WT | -1.15478 | 4.13E-04 |
| PA14_46170 | transcriptional_regulators | PGD1658280 | NA | DcbrA vs WT | -2.05284 | 3.44E-12 |
| PA14_46250 | putative_enzymes | PGD1658292 | NA | DcbrA vs WT | 1.003638 | 3.24E-07 |
| PA14_46260 | hypothetical_unclassified | PGD1658294 | NA | DcbrA vs WT | -1.07422 | 6.69E-07 |
| PA14_46280 | hypothetical_unclassified | PGD1658298 | NA | DcbrA vs WT | 1.456927 | 2.90E-10 |
| PA14_46470 | amino_acid_biosynthesis_me | PGD1658332 | pdxB | DcbrA vs WT | -1.26118 | 1.12E-07 |
| PA14_46470 | biosynthesis_of_cofactors | PGD1658332 | pdxB | DcbrA vs WT | -1.26118 | 1.12E-07 |
| PA14_46470 | carbon_compound_catabolis | PGD1658332 | pdxB | DcbrA vs WT | -1.26118 | 1.12E-07 |
| PA14_46570 | transcriptional_regulators | PGD1658350 | NA | DcbrA vs WT | 1.045214 | 3.68E-08 |
| PA14_46590 | antibiotic_resistance_and_su | PGD1658354 | NA | DcbrA vs WT | 1.016035 | 0.001183 |

| | | | | | | |
|---|---|---|---|---|---|---|
| PA14_46620 | putative_enzymes | PGD1658360 | NA | DcbrA vs WT | 1.10112 | 2.83E-07 |
| PA14_46740 | hypothetical_unclassified | PGD1658380 | NA | DcbrA vs WT | -1.0972 | 1.54E-06 |
| PA14_46750 | hypothetical_unclassified | PGD1658382 | NA | DcbrA vs WT | -1.92134 | 2.16E-08 |
| PA14_46810 | transcriptional_regulators | PGD1658392 | NA | DcbrA vs WT | -1.20778 | 6.84E-06 |
| PA14_46850 | transcriptional_regulators | PGD1658400 | NA | DcbrA vs WT | 3.032677 | 3.21E-04 |
| PA14_46860 | amino_acid_biosynthesis_me | PGD1658402 | NA | DcbrA vs WT | 1.552652 | 0.039608 |
| PA14_46890 | putative_enzymes | PGD1658406 | NA | DcbrA vs WT | -1.00255 | 4.58E-06 |
| PA14_46970 | amino_acid_biosynthesis_me | PGD1658420 | ansB | DcbrA vs WT | -1.79641 | 5.22E-18 |
| PA14_46980 | two_component_regulators | PGD1658422 | NA | DcbrA vs WT | -1.26161 | 1.31E-10 |
| PA14_47080 | transcriptional_regulators | PGD1658438 | NA | DcbrA vs WT | -1.85835 | 2.66E-10 |
| PA14_47100 | amino_acid_biosynthesis_me | PGD1658442 | ilvA2 | DcbrA vs WT | -1.54219 | 1.13E-05 |
| PA14_47120 | hypothetical_unclassified | PGD1658446 | NA | DcbrA vs WT | 1.533579 | 1.99E-12 |
| PA14_47140 | membrane_proteins | PGD1658450 | NA | DcbrA vs WT | 1.299559 | 6.32E-07 |
| PA14_47180 | energy_metabolism_CAP | PGD1658456 | cyoC | DcbrA vs WT | 1.263369 | 0.03264 |
| PA14_47230 | transport_of_small_molecule | PGD1658462 | NA | DcbrA vs WT | -1.21603 | 6.38E-05 |
| PA14_47390 | two_component_regulators | PGD1658492 | NA | DcbrA vs WT | -1.1784 | 0.002413 |
| PA14_47460 | transcription_RNA_processin | PGD1658506 | rnd | DcbrA vs WT | 1.589575 | 7.27E-25 |
| PA14_47530 | hypothetical_unclassified | PGD1658516 | NA | DcbrA vs WT | -1.084 | 8.97E-05 |
| PA14_47640 | transport_of_small_molecule | PGD1658530 | NA | DcbrA vs WT | 1.079916 | 0.00738 |
| PA14_47650 | biosynthesis_of_cofactors | PGD1658532 | cobS | DcbrA vs WT | -1.68845 | 8.08E-07 |
| PA14_47670 | biosynthesis_of_cofactors | PGD1658536 | cobT | DcbrA vs WT | -3.00647 | 1.78E-12 |
| PA14_47680 | biosynthesis_of_cofactors | PGD1658538 | cobU | DcbrA vs WT | -2.59556 | 3.02E-13 |
| PA14_47690 | biosynthesis_of_cofactors | PGD1658540 | cobQ | DcbrA vs WT | -1.04503 | 1.98E-07 |
| PA14_47720 | biosynthesis_of_cofactors | PGD1658542 | cobC | DcbrA vs WT | -2.23549 | 2.40E-09 |
| PA14_47730 | biosynthesis_of_cofactors | PGD1658544 | cobD | DcbrA vs WT | -2.57191 | 4.79E-14 |
| PA14_47750 | putative_enzymes | PGD1658546 | NA | DcbrA vs WT | -1.41794 | 2.33E-06 |
| PA14_47760 | biosynthesis_of_cofactors | PGD1658548 | cobB | DcbrA vs WT | -1.91992 | 9.34E-15 |
| PA14_47860 | putative_enzymes | PGD1658562 | NA | DcbrA vs WT | -2.61009 | 2.50E-09 |
| PA14_47870 | membrane_proteins | PGD1658564 | NA | DcbrA vs WT | -1.91813 | 0.043055 |
| PA14_47880 | transcriptional_regulators | PGD1658566 | NA | DcbrA vs WT | -1.63623 | 2.91E-07 |
| PA14_47910 | transcriptional_regulators | PGD1658572 | NA | DcbrA vs WT | -1.27219 | 5.11E-05 |
| PA14_47920 | transport_of_small_molecule | PGD1658574 | NA | DcbrA vs WT | 2.063518 | 2.03E-04 |
| PA14_48000 | amino_acid_biosynthesis_me | PGD1658586 | NA | DcbrA vs WT | 1.180811 | 0.006323 |
| PA14_48160 | two_component_regulators | PGD1658608 | NA | DcbrA vs WT | 1.218151 | 1.56E-12 |
| PA14_48170 | motility_and_attachment | PGD1658610 | srpP | DcbrA vs WT | 1.365069 | 5.39E-16 |
| PA14_48170 | translation_posttranslational | PGD1658610 | srpP | DcbrA vs WT | 1.365069 | 5.39E-16 |
| PA14_48240 | antibiotic_resistance_and_su | PGD1658620 | NA | DcbrA vs WT | -1.84392 | 0.025531 |
| PA14_48300 | transport_of_small_molecule | PGD1658624 | NA | DcbrA vs WT | 1.081904 | 0.001888 |
| PA14_48420 | transcriptional_regulators | PGD1658644 | NA | DcbrA vs WT | -2.0566 | 1.59E-17 |
| PA14_48440 | putative_enzymes | PGD1658646 | NA | DcbrA vs WT | -2.34237 | 3.34E-07 |
| PA14_48560 | hypothetical_unclassified | PGD1658668 | NA | DcbrA vs WT | 1.503222 | 0.009268 |
| PA14_48710 | hypothetical_unclassified | PGD1658692 | NA | DcbrA vs WT | 1.2454 | 6.16E-10 |
| PA14_48730 | hypothetical_unclassified | PGD1658694 | NA | DcbrA vs WT | -1.65687 | 8.04E-09 |
| PA14_48830 | transcriptional_regulators | PGD1658712 | NA | DcbrA vs WT | -1.14037 | 1.53E-08 |
| PA14_48880 | relative_phage_transposon | PGD1658722 | NA | DcbrA vs WT | 1.189998 | 5.43E-06 |
| PA14_49010 | relative_phage_transposon | PGD1658748 | xisF5 | DcbrA vs WT | 3.195688 | 0.036784 |
| PA14_49050 | membrane_proteins | PGD1658756 | NA | DcbrA vs WT | 1.243953 | 7.04E-09 |
| PA14_49060 | transcription_RNA_processin | PGD1658758 | NA | DcbrA vs WT | -1.05858 | 0.001099 |
| PA14_49070 | putative_enzymes | PGD1658760 | NA | DcbrA vs WT | -1.38947 | 1.01E-08 |
| PA14_49130 | transport_of_small_molecule | PGD1658770 | dctA | DcbrA vs WT | 2.456085 | 5.72E-12 |

| | | | | | | |
|---|---|---|---|---|---|---|
| PA14_49210 | energy_metabolism_CAP | PGD1658782 | napE | DcbrA vs WT | 1.376166 | 3.90E-19 |
| PA14_49220 | energy_metabolism_CAP | PGD1658784 | napF | DcbrA vs WT | -1.92621 | 2.27E-09 |
| PA14_49300 | putative_enzymes | PGD1658798 | NA | DcbrA vs WT | 3.280647 | 1.50E-52 |
| PA14_49310 | hypothetical_unclassified | PGD1658800 | NA | DcbrA vs WT | 3.600412 | 1.36E-58 |
| PA14_49390 | transcription_RNA_processin | PGD1658814 | rrmA | DcbrA vs WT | -1.10268 | 0.009798 |
| PA14_49400 | hypothetical_unclassified | PGD1658816 | NA | DcbrA vs WT | -1.66277 | 6.72E-17 |
| PA14_49500 | hypothetical_unclassified | PGD1658830 | NA | DcbrA vs WT | -2.24726 | 7.87E-06 |
| PA14_49560 | secreted_factors | PGD1658840 | toxA | DcbrA vs WT | -1.07616 | 0.009566 |
| PA14_49570 | membrane_proteins | PGD1658842 | NA | DcbrA vs WT | -1.20399 | 0.028389 |
| PA14_49580 | carbon_compound_catabolis | PGD1658844 | NA | DcbrA vs WT | -1.43042 | 0.003302 |
| PA14_49690 | putative_enzymes | PGD1658862 | NA | DcbrA vs WT | 2.671752 | 2.00E-08 |
| PA14_49780 | antibiotic_resistance_and_su | PGD1658878 | NA | DcbrA vs WT | -1.11146 | 0.027412 |
| PA14_49820 | biosynthesis_of_cofactors | PGD1658886 | NA | DcbrA vs WT | -1.27042 | 9.56E-07 |
| PA14_49850 | hypothetical_unclassified | PGD1658890 | NA | DcbrA vs WT | -1.01319 | 2.31E-09 |
| PA14_49860 | hypothetical_unclassified | PGD1658892 | NA | DcbrA vs WT | -1.61299 | 3.15E-13 |
| PA14_49940 | membrane_proteins | PGD1658908 | NA | DcbrA vs WT | 1.438674 | 3.34E-23 |
| PA14_49960 | hypothetical_unclassified | PGD1658910 | NA | DcbrA vs WT | 1.04719 | 4.60E-10 |
| PA14_50000 | hypothetical_unclassified | PGD1658916 | NA | DcbrA vs WT | 1.354837 | 5.05E-12 |
| PA14_50010 | putative_enzymes | PGD1658918 | NA | DcbrA vs WT | 1.012487 | 3.89E-09 |
| PA14_50040 | transcriptional_regulators | PGD1658924 | NA | DcbrA vs WT | -1.03342 | 2.15E-04 |
| PA14_50070 | hypothetical_unclassified | PGD1658930 | NA | DcbrA vs WT | 1.379929 | 1.96E-10 |
| PA14_50100 | motility_and_attachment | PGD1658934 | fliI | DcbrA vs WT | -1.29137 | 9.83E-15 |
| PA14_50180 | motility_and_attachment | PGD1658944 | fleR | DcbrA vs WT | -1.02195 | 5.88E-06 |
| PA14_50180 | transcriptional_regulators | PGD1658944 | fleR | DcbrA vs WT | -1.02195 | 5.88E-06 |
| PA14_50180 | two_component_regulators | PGD1658944 | fleR | DcbrA vs WT | -1.02195 | 5.88E-06 |
| PA14_50450 | cell_wall_LPS | PGD1658982 | flgE | DcbrA vs WT | -1.64959 | 8.51E-29 |
| PA14_50450 | motility_and_attachment | PGD1658982 | flgE | DcbrA vs WT | -1.64959 | 8.51E-29 |
| PA14_50470 | cell_wall_LPS | PGD1658986 | flgC | DcbrA vs WT | -1.08449 | 7.50E-04 |
| PA14_50470 | motility_and_attachment | PGD1658986 | flgC | DcbrA vs WT | -1.08449 | 7.50E-04 |
| PA14_50650 | hypothetical_unclassified | PGD1659018 | NA | DcbrA vs WT | -1.44755 | 1.36E-05 |
| PA14_50720 | membrane_proteins | PGD1659032 | shaE | DcbrA vs WT | -1.15389 | 1.11E-04 |
| PA14_50720 | transport_of_small_molecule | PGD1659032 | shaE | DcbrA vs WT | -1.15389 | 1.11E-04 |
| PA14_50750 | hypothetical_unclassified | PGD1659038 | NA | DcbrA vs WT | -1.17753 | 1.40E-05 |
| PA14_50760 | putative_enzymes | PGD1659040 | NA | DcbrA vs WT | -3.14156 | 2.26E-10 |
| PA14_50810 | membrane_proteins | PGD1659048 | NA | DcbrA vs WT | 1.209525 | 5.43E-15 |
| PA14_50810 | transport_of_small_molecule | PGD1659048 | NA | DcbrA vs WT | 1.209525 | 5.43E-15 |
| PA14_50860 | hypothetical_unclassified | PGD1659058 | NA | DcbrA vs WT | -1.14675 | 8.84E-11 |
| PA14_50880 | membrane_proteins | PGD1659062 | NA | DcbrA vs WT | -1.30904 | 1.78E-19 |
| PA14_50970 | putative_enzymes | PGD1659078 | NA | DcbrA vs WT | 1.662766 | 3.04E-15 |
| PA14_51090 | putative_enzymes | PGD1659098 | NA | DcbrA vs WT | 1.204788 | 0.026143 |
| PA14_51190 | hypothetical_unclassified | PGD1659112 | NA | DcbrA vs WT | -1.1883 | 1.95E-04 |
| PA14_51200 | hypothetical_unclassified | PGD1659114 | NA | DcbrA vs WT | 1.134574 | 4.22E-05 |
| PA14_51440 | DNA_replication_recombinat | PGD1659158 | ogt | DcbrA vs WT | 1.031038 | 4.75E-07 |
| PA14_51460 | motility_and_attachment | PGD1659162 | cupC2 | DcbrA vs WT | 1.864481 | 5.78E-04 |
| PA14_51490 | hypothetical_unclassified | PGD1659168 | NA | DcbrA vs WT | 2.240153 | 3.74E-24 |
| PA14_51510 | hypothetical_unclassified | PGD1659172 | NA | DcbrA vs WT | 1.352172 | 5.52E-11 |
| PA14_51530 | secreted_factors | PGD1659176 | exoU | DcbrA vs WT | 1.087851 | 4.68E-11 |
| PA14_51550 | relative_phage_transposon | PGD1659180 | NA | DcbrA vs WT | 1.039717 | 1.68E-07 |
| PA14_51620 | relative_phage_transposon | PGD1659194 | NA | DcbrA vs WT | 1.93151 | 2.87E-18 |
| PA14_51630 | relative_phage_transposon | PGD1659196 | NA | DcbrA vs WT | 1.599778 | 2.58E-06 |

| | | | | | | |
|---|---|---|---|---|---|---|
| PA14_51730 | membrane_proteins | PGD1659214 | tolA | DcbrA vs WT | 1.350942 | 8.05E-27 |
| PA14_51730 | transport_of_small_molecule | PGD1659214 | tolA | DcbrA vs WT | 1.350942 | 8.05E-27 |
| PA14_51770 | hypothetical_unclassified | PGD1659220 | NA | DcbrA vs WT | 1.058727 | 5.24E-10 |
| PA14_51790 | DNA_replication_recombinat | PGD1659224 | ruvA | DcbrA vs WT | 1.416114 | 3.21E-22 |
| PA14_51930 | putative_enzymes | PGD1659250 | NA | DcbrA vs WT | 1.252006 | 1.78E-10 |
| PA14_51960 | transcription_RNA_processin | PGD1659256 | NA | DcbrA vs WT | 1.172611 | 9.11E-13 |
| PA14_52060 | protein_secretion_export | PGD1659272 | NA | DcbrA vs WT | 1.205521 | 7.08E-19 |
| PA14_52120 | hypothetical_unclassified | PGD1659282 | NA | DcbrA vs WT | 1.364639 | 1.35E-04 |
| PA14_52230 | transport_of_small_molecule | PGD1659298 | NA | DcbrA vs WT | 1.755542 | 2.07E-17 |
| PA14_52250 | transport_of_small_molecule | PGD1659302 | NA | DcbrA vs WT | 2.437857 | 3.59E-20 |
| PA14_52250 | two_component_regulators | PGD1659302 | NA | DcbrA vs WT | 2.437857 | 3.59E-20 |
| PA14_52300 | membrane_proteins | PGD1659312 | NA | DcbrA vs WT | 1.036332 | 5.66E-05 |
| NA | NA | PGD1659317 | NA | DcbrA vs WT | 1.496032 | 0.017329 |
| PA14_52460 | transport_of_small_molecule | PGD1659336 | mgtE | DcbrA vs WT | 1.013611 | 1.03E-14 |
| PA14_52480 | hypothetical_unclassified | PGD1659340 | NA | DcbrA vs WT | 1.121978 | 0.042578 |
| PA14_52510 | hypothetical_unclassified | PGD1659346 | NA | DcbrA vs WT | -1.60705 | 4.80E-06 |
| PA14_52570 | adaptation_and_protection | PGD1659358 | rsmA | DcbrA vs WT | 1.617028 | 5.38E-11 |
| PA14_52570 | transcriptional_regulators | PGD1659358 | rsmA | DcbrA vs WT | 1.617028 | 5.38E-11 |
| PA14_52570 | translation_posttranslational | PGD1659358 | rsmA | DcbrA vs WT | 1.617028 | 5.38E-11 |
| PA14_52610 | putative_enzymes | PGD1659364 | NA | DcbrA vs WT | -1.53986 | 7.44E-18 |
| PA14_52660 | amino_acid_biosynthesis_me | PGD1659370 | aruB | DcbrA vs WT | -1.62551 | 5.60E-17 |
| PA14_52760 | hypothetical_unclassified | PGD1659386 | NA | DcbrA vs WT | -1.10691 | 1.83E-11 |
| PA14_52800 | central_intermediary_metab | PGD1659394 | acsA | DcbrA vs WT | 1.720266 | 2.30E-20 |
| PA14_52840 | transport_of_small_molecule | PGD1659400 | NA | DcbrA vs WT | 1.545966 | 2.52E-07 |
| PA14_52880 | putative_enzymes | PGD1659406 | NA | DcbrA vs WT | 3.519719 | 0.015503 |
| PA14_52890 | putative_enzymes | PGD1659408 | NA | DcbrA vs WT | 2.238444 | 0.046353 |
| PA14_53010 | amino_acid_biosynthesis_me | PGD1659428 | phhC | DcbrA vs WT | -1.46945 | 6.44E-13 |
| PA14_53070 | amino_acid_biosynthesis_me | PGD1659438 | hpd | DcbrA vs WT | 1.757861 | 1.29E-24 |
| PA14_53160 | hypothetical_unclassified | PGD1659452 | NA | DcbrA vs WT | -1.14052 | 4.47E-11 |
| PA14_53200 | hypothetical_unclassified | PGD1659458 | NA | DcbrA vs WT | 1.286787 | 1.70E-20 |
| PA14_53210 | hypothetical_unclassified | PGD1659460 | NA | DcbrA vs WT | -1.04645 | 7.09E-11 |
| PA14_53250 | secreted_factors | PGD1659466 | cpbD | DcbrA vs WT | -1.16249 | 3.29E-11 |
| PA14_53270 | hypothetical_unclassified | PGD1659470 | NA | DcbrA vs WT | -1.70184 | 9.26E-09 |
| PA14_53290 | nucleotide_biosynthesis_and | PGD1659472 | trxB2 | DcbrA vs WT | -1.93247 | 1.53E-14 |
| PA14_53390 | hypothetical_unclassified | PGD1659488 | NA | DcbrA vs WT | -1.37191 | 5.61E-04 |
| PA14_53450 | hypothetical_unclassified | PGD1659498 | NA | DcbrA vs WT | 1.009954 | 6.20E-07 |
| PA14_53470 | putative_enzymes | PGD1659500 | NA | DcbrA vs WT | -1.41752 | 1.88E-06 |
| PA14_53720 | transcriptional_regulators | PGD1659548 | NA | DcbrA vs WT | -1.87605 | 1.73E-09 |
| PA14_53750 | hypothetical_unclassified | PGD1659554 | NA | DcbrA vs WT | -1.94591 | 6.19E-05 |
| PA14_53780 | membrane_proteins | PGD1659558 | NA | DcbrA vs WT | -1.1341 | 1.92E-09 |
| PA14_53780 | transport_of_small_molecule | PGD1659558 | NA | DcbrA vs WT | -1.1341 | 1.92E-09 |
| PA14_53910 | fatty_acid_and_phospholipid | PGD1659584 | pmtA | DcbrA vs WT | -1.22455 | 1.78E-08 |
| PA14_53970 | energy_metabolism_CAP | PGD1659592 | NA | DcbrA vs WT | -1.15236 | 6.76E-09 |
| PA14_54050 | hypothetical_unclassified | PGD1659604 | NA | DcbrA vs WT | -1.38791 | 1.38E-06 |
| PA14_54230 | hypothetical_unclassified | PGD1659630 | NA | DcbrA vs WT | -1.21713 | 0.007698 |
| PA14_54240 | hypothetical_unclassified | PGD1659632 | NA | DcbrA vs WT | 1.943357 | 3.84E-23 |
| PA14_54400 | cell_wall_LPS | PGD1659654 | mucC | DcbrA vs WT | 1.131429 | 4.65E-13 |
| PA14_54400 | secreted_factors | PGD1659654 | mucC | DcbrA vs WT | 1.131429 | 4.65E-13 |
| PA14_54400 | transcriptional_regulators | PGD1659654 | mucC | DcbrA vs WT | 1.131429 | 4.65E-13 |
| PA14_54430 | transcriptional_regulators | PGD1659660 | algU | DcbrA vs WT | 1.535769 | 3.85E-17 |

| | | | | | | |
|---|---|---|---|---|---|---|
| PA14_54520 | membrane_proteins | PGD1659676 | NA | DcbrA vs WT | 2.708614 | 1.37E-19 |
| PA14_54580 | membrane_proteins | PGD1659684 | NA | DcbrA vs WT | -1.57903 | 7.10E-04 |
| PA14_54630 | putative_enzymes | PGD1659694 | NA | DcbrA vs WT | -1.81932 | 3.75E-28 |
| PA14_54640 | putative_enzymes | PGD1659696 | dspI | DcbrA vs WT | -1.74051 | 1.03E-25 |
| PA14_54660 | putative_enzymes | PGD1659698 | NA | DcbrA vs WT | -1.38817 | 7.49E-13 |
| PA14_54670 | carbon_compound_catabolis | PGD1659700 | NA | DcbrA vs WT | -1.06128 | 1.48E-11 |
| PA14_54720 | hypothetical_unclassified | PGD1659710 | NA | DcbrA vs WT | 2.355937 | 1.47E-11 |
| PA14_54730 | membrane_proteins | PGD1659712 | NA | DcbrA vs WT | 2.052766 | 6.40E-13 |
| PA14_54740 | hypothetical_unclassified | PGD1659714 | NA | DcbrA vs WT | 1.367638 | 4.86E-07 |
| PA14_54750 | hypothetical_unclassified | PGD1659716 | NA | DcbrA vs WT | 1.661375 | 3.76E-12 |
| PA14_54870 | hypothetical_unclassified | PGD1659738 | NA | DcbrA vs WT | 1.250879 | 2.32E-05 |
| PA14_54880 | putative_enzymes | PGD1659740 | NA | DcbrA vs WT | 1.146846 | 1.37E-08 |
| PA14_54890 | hypothetical_unclassified | PGD1659742 | NA | DcbrA vs WT | 1.009476 | 1.47E-10 |
| PA14_54910 | putative_enzymes | PGD1659746 | NA | DcbrA vs WT | 1.275781 | 1.07E-05 |
| PA14_55000 | transport_of_small_molecule | PGD1659762 | NA | DcbrA vs WT | 1.010627 | 0.001888 |
| PA14_55020 | transport_of_small_molecule | PGD1659764 | NA | DcbrA vs WT | 1.428593 | 4.84E-05 |
| PA14_55040 | transport_of_small_molecule | PGD1659768 | NA | DcbrA vs WT | 1.585092 | 5.65E-04 |
| PA14_55130 | central_intermediary_metab | PGD1659786 | gloA2 | DcbrA vs WT | -1.1796 | 0.04503 |
| PA14_55150 | transcriptional_regulators | PGD1659790 | NA | DcbrA vs WT | 1.406492 | 4.45E-06 |
| PA14_55160 | transcriptional_regulators | PGD1659792 | toxR | DcbrA vs WT | -1.55568 | 1.78E-05 |
| PA14_55240 | hypothetical_unclassified | PGD1659804 | NA | DcbrA vs WT | -1.71548 | 2.42E-09 |
| PA14_55250 | transcriptional_regulators | PGD1659806 | NA | DcbrA vs WT | -1.0601 | 0.020501 |
| PA14_55460 | protein_secretion_export | PGD1659838 | hxcZ | DcbrA vs WT | -3.68727 | 0.034768 |
| PA14_55510 | hypothetical_unclassified | PGD1659848 | hxcP | DcbrA vs WT | -1.54097 | 1.99E-05 |
| PA14_55560 | transcriptional_regulators | PGD1659858 | NA | DcbrA vs WT | -1.47185 | 2.49E-04 |
| PA14_55590 | hypothetical_unclassified | PGD1659864 | NA | DcbrA vs WT | -1.98091 | 9.12E-04 |
| PA14_55600 | hypothetical_unclassified | PGD1659866 | NA | DcbrA vs WT | -2.87605 | 3.79E-13 |
| PA14_55740 | transport_of_small_molecule | PGD1659896 | NA | DcbrA vs WT | -1.36585 | 2.27E-07 |
| PA14_55750 | adaptation_and_protection | PGD1659898 | NA | DcbrA vs WT | 2.070838 | 1.31E-22 |
| PA14_55750 | chemotaxis | PGD1659898 | NA | DcbrA vs WT | 2.070838 | 1.31E-22 |
| PA14_55780 | two_component_regulators | PGD1659904 | NA | DcbrA vs WT | -1.82426 | 1.42E-29 |
| PA14_55790 | protein_secretion_export | PGD1659906 | NA | DcbrA vs WT | -1.02273 | 7.49E-11 |
| PA14_55840 | hypothetical_unclassified | PGD1659914 | NA | DcbrA vs WT | -2.72758 | 0.001705 |
| PA14_55850 | protein_secretion_export | PGD1659916 | NA | DcbrA vs WT | -1.31806 | 3.46E-07 |
| PA14_55880 | membrane_proteins | PGD1659920 | NA | DcbrA vs WT | -1.80371 | 4.64E-12 |
| PA14_55880 | motility_and_attachment | PGD1659920 | NA | DcbrA vs WT | -1.80371 | 4.64E-12 |
| PA14_55880 | protein_secretion_export | PGD1659920 | NA | DcbrA vs WT | -1.80371 | 4.64E-12 |
| PA14_55920 | motility_and_attachment | PGD1659926 | NA | DcbrA vs WT | -1.11013 | 1.97E-09 |
| PA14_55920 | protein_secretion_export | PGD1659926 | NA | DcbrA vs WT | -1.11013 | 1.97E-09 |
| PA14_55930 | motility_and_attachment | PGD1659928 | NA | DcbrA vs WT | -1.13719 | 2.95E-13 |
| PA14_55930 | protein_secretion_export | PGD1659928 | NA | DcbrA vs WT | -1.13719 | 2.95E-13 |
| PA14_56050 | membrane_proteins | PGD1659944 | NA | DcbrA vs WT | 1.323097 | 3.15E-18 |
| PA14_56080 | DNA_replication_recombinat | PGD1659950 | sbcB | DcbrA vs WT | 1.022829 | 2.28E-21 |
| PA14_56100 | membrane_proteins | PGD1659954 | NA | DcbrA vs WT | -1.20558 | 2.19E-06 |
| PA14_56170 | hypothetical_unclassified | PGD1659964 | NA | DcbrA vs WT | -1.40386 | 1.04E-10 |
| PA14_56180 | hypothetical_unclassified | PGD1659966 | NA | DcbrA vs WT | 1.390042 | 9.34E-15 |
| PA14_56260 | energy_metabolism_CAP | PGD1659980 | NA | DcbrA vs WT | -2.36499 | 9.29E-38 |
| PA14_56390 | fatty_acid_and_phospholipid | PGD1659994 | NA | DcbrA vs WT | 1.612282 | 1.95E-30 |
| PA14_56510 | hypothetical_unclassified | PGD1660008 | NA | DcbrA vs WT | 1.044464 | 6.40E-11 |
| PA14_56570 | fatty_acid_and_phospholipid | PGD1660020 | NA | DcbrA vs WT | -2.90659 | 4.22E-08 |

| | | | | | | |
|---|---|---|---|---|---|---|
| PA14_56590 | adaptation_and_protection | PGD1660022 | NA | DcbrA vs WT | -2.50195 | 2.70E-20 |
| PA14_56620 | transcriptional_regulators | PGD1660026 | NA | DcbrA vs WT | 2.44762 | 8.82E-13 |
| PA14_56670 | hypothetical_unclassified | PGD1660032 | NA | DcbrA vs WT | -1.7491 | 4.45E-08 |
| PA14_56690 | transport_of_small_molecule | PGD1660036 | feoA | DcbrA vs WT | -1.47563 | 5.77E-07 |
| PA14_56730 | hypothetical_unclassified | PGD1660042 | NA | DcbrA vs WT | -1.19973 | 4.95E-08 |
| PA14_56750 | hypothetical_unclassified | PGD1660046 | NA | DcbrA vs WT | -1.31397 | 0.024013 |
| PA14_56800 | hypothetical_unclassified | PGD1660054 | NA | DcbrA vs WT | -1.42809 | 2.91E-14 |
| PA14_56810 | membrane_proteins | PGD1660056 | NA | DcbrA vs WT | 1.01646 | 1.58E-08 |
| PA14_56830 | membrane_proteins | PGD1660058 | icmP | DcbrA vs WT | -1.42555 | 6.53E-10 |
| PA14_56840 | putative_enzymes | PGD1660060 | NA | DcbrA vs WT | -1.70742 | 1.14E-17 |
| PA14_56870 | hypothetical_unclassified | PGD1660064 | NA | DcbrA vs WT | -1.29639 | 4.03E-08 |
| PA14_56910 | hypothetical_unclassified | PGD1660072 | NA | DcbrA vs WT | -1.39536 | 1.36E-08 |
| PA14_56960 | hypothetical_unclassified | PGD1660082 | NA | DcbrA vs WT | 1.222276 | 0.024309 |
| PA14_57020 | chaperones_heat_shock | PGD1660092 | groES | DcbrA vs WT | 1.29469 | 1.20E-13 |
| PA14_57110 | transport_of_small_molecule | PGD1660108 | NA | DcbrA vs WT | 1.931507 | 6.30E-33 |
| PA14_57275 | cell_division | PGD1660134 | ftsZ | DcbrA vs WT | -1.18105 | 1.42E-12 |
| PA14_57530 | adaptation_and_protection | PGD1660176 | sspA | DcbrA vs WT | 1.121091 | 2.18E-11 |
| PA14_57630 | transcriptional_regulators | PGD1660191 | NA | DcbrA vs WT | 1.069976 | 1.49E-09 |
| PA14_57710 | amino_acid_biosynthesis_me | PGD1660203 | cysN | DcbrA vs WT | -1.45569 | 3.00E-15 |
| PA14_57710 | central_intermediary_metab | PGD1660203 | cysN | DcbrA vs WT | -1.45569 | 3.00E-15 |
| PA14_57990 | transport_of_small_molecule | PGD1660253 | NA | DcbrA vs WT | -1.36431 | 0.005956 |
| PA14_58060 | hypothetical_unclassified | PGD1660265 | NA | DcbrA vs WT | -1.10761 | 3.34E-06 |
| PA14_58080 | putative_enzymes | PGD1660269 | NA | DcbrA vs WT | -1.30038 | 1.06E-10 |
| PA14_58240 | hypothetical_unclassified | PGD1660295 | NA | DcbrA vs WT | -1.27467 | 4.83E-14 |
| PA14_58410 | membrane_proteins | PGD1660321 | opdP | DcbrA vs WT | -2.6078 | 1.28E-35 |
| PA14_58410 | transport_of_small_molecule | PGD1660321 | opdP | DcbrA vs WT | -2.6078 | 1.28E-35 |
| PA14_58420 | transport_of_small_molecule | PGD1660323 | dppA4 | DcbrA vs WT | -1.06904 | 7.08E-07 |
| PA14_58450 | membrane_proteins | PGD1660327 | dppC | DcbrA vs WT | -2.09632 | 3.61E-10 |
| PA14_58450 | transport_of_small_molecule | PGD1660327 | dppC | DcbrA vs WT | -2.09632 | 3.61E-10 |
| PA14_58470 | transport_of_small_molecule | PGD1660329 | dppD | DcbrA vs WT | -2.56693 | 4.39E-18 |
| PA14_58490 | transport_of_small_molecule | PGD1660331 | dppF | DcbrA vs WT | -2.02384 | 5.49E-19 |
| PA14_58580 | membrane_proteins | PGD1660349 | NA | DcbrA vs WT | 1.318776 | 1.06E-04 |
| PA14_58600 | hypothetical_unclassified | PGD1660351 | NA | DcbrA vs WT | -1.02662 | 2.44E-04 |
| PA14_58610 | putative_enzymes | PGD1660353 | NA | DcbrA vs WT | 1.519918 | 2.65E-06 |
| NA | NA | PGD1660370 | NA | DcbrA vs WT | 1.706012 | 0.001324 |
| PA14_58810 | membrane_proteins | PGD1660389 | NA | DcbrA vs WT | 1.071151 | 8.53E-07 |
| PA14_58880 | energy_metabolism_CAP | PGD1660403 | NA | DcbrA vs WT | 1.173143 | 2.44E-13 |
| PA14_58920 | hypothetical_unclassified | PGD1660411 | NA | DcbrA vs WT | 1.202277 | 0.006156 |
| PA14_58990 | putative_enzymes | PGD1660425 | NA | DcbrA vs WT | 1.361066 | 0.002549 |
| PA14_59100 | hypothetical_unclassified | PGD1660443 | NA | DcbrA vs WT | 1.149824 | 7.02E-06 |
| PA14_59160 | hypothetical_unclassified | PGD1660455 | crpP | DcbrA vs WT | 1.013223 | 0.013805 |
| PA14_59250 | motility_and_attachment | PGD1660473 | pilN2 | DcbrA vs WT | 1.408186 | 9.34E-05 |
| PA14_59370 | hypothetical_unclassified | PGD1660491 | NA | DcbrA vs WT | 1.190224 | 3.64E-07 |
| PA14_59410 | hypothetical_unclassified | PGD1660499 | NA | DcbrA vs WT | 1.343659 | 0.001556 |
| PA14_59690 | hypothetical_unclassified | PGD1660549 | NA | DcbrA vs WT | 1.335134 | 5.20E-05 |
| PA14_59750 | motility_and_attachment | PGD1660558 | cupD4 | DcbrA vs WT | 1.561672 | 1.47E-05 |
| PA14_59860 | protein_secretion_export | PGD1660580 | NA | DcbrA vs WT | 3.59589 | 0.031261 |
| PA14_59970 | hypothetical_unclassified | PGD1660602 | NA | DcbrA vs WT | 1.166185 | 2.07E-06 |
| PA14_59990 | hypothetical_unclassified | PGD1660606 | NA | DcbrA vs WT | 1.127543 | 0.033812 |
| PA14_60140 | DNA_replication_recombinat | PGD1660636 | NA | DcbrA vs WT | 1.270101 | 0.007819 |

| | | | | | | |
|---|---|---|---|---|---|---|
| NA | NA | PGD1660639 | NA | DcbrA vs WT | -1.1533 | 2.19E-05 |
| PA14_60240 | hypothetical_unclassified | PGD1660652 | NA | DcbrA vs WT | -1.52748 | 2.14E-14 |
| PA14_60360 | fatty_acid_and_phospholipid | PGD1660674 | lspA | DcbrA vs WT | 1.152169 | 9.62E-13 |
| PA14_60360 | protein_secretion_export | PGD1660674 | lspA | DcbrA vs WT | 1.152169 | 9.62E-13 |
| PA14_60360 | translation_posttranslational | PGD1660674 | lspA | DcbrA vs WT | 1.152169 | 9.62E-13 |
| PA14_60490 | energy_metabolism_CAP | PGD1660698 | NA | DcbrA vs WT | -1.26867 | 2.92E-07 |
| PA14_60530 | membrane_proteins | PGD1660704 | NA | DcbrA vs WT | 1.588685 | 1.38E-13 |
| PA14_60560 | hypothetical_unclassified | PGD1660710 | NA | DcbrA vs WT | -3.25808 | 1.50E-28 |
| PA14_60700 | energy_metabolism_CAP | PGD1660732 | ccpR | DcbrA vs WT | -1.75223 | 1.34E-13 |
| PA14_60750 | carbon_compound_catabolis | PGD1660738 | pra | DcbrA vs WT | -1.30969 | 9.63E-05 |
| PA14_60770 | membrane_proteins | PGD1660742 | NA | DcbrA vs WT | -1.8158 | 9.47E-26 |
| PA14_60780 | membrane_proteins | PGD1660744 | NA | DcbrA vs WT | -1.11672 | 5.27E-05 |
| PA14_60780 | transport_of_small_molecule | PGD1660744 | NA | DcbrA vs WT | -1.11672 | 5.27E-05 |
| PA14_60900 | hypothetical_unclassified | PGD1660764 | NA | DcbrA vs WT | -1.20819 | 1.75E-11 |
| PA14_61000 | hypothetical_unclassified | PGD1660778 | NA | DcbrA vs WT | -3.1205 | 6.34E-18 |
| PA14_61010 | hypothetical_unclassified | PGD1660780 | NA | DcbrA vs WT | 1.305173 | 3.42E-07 |
| PA14_61140 | energy_metabolism_CAP | PGD1660800 | NA | DcbrA vs WT | 1.118481 | 0.022377 |
| PA14_61190 | cell_wall_LPS | PGD1660808 | NA | DcbrA vs WT | 1.004253 | 1.02E-12 |
| PA14_61190 | protein_secretion_export | PGD1660808 | NA | DcbrA vs WT | 1.004253 | 1.02E-12 |
| PA14_61220 | translation_posttranslational | PGD1660814 | NA | DcbrA vs WT | -1.61714 | 5.24E-06 |
| PA14_61260 | membrane_proteins | PGD1660818 | NA | DcbrA vs WT | -1.459 | 1.55E-04 |
| PA14_61320 | hypothetical_unclassified | PGD1660828 | NA | DcbrA vs WT | 1.115114 | 3.53E-07 |
| PA14_61340 | hypothetical_unclassified | PGD1660832 | NA | DcbrA vs WT | -2.29374 | 6.41E-17 |
| PA14_61370 | hypothetical_unclassified | PGD1660838 | NA | DcbrA vs WT | 2.207994 | 1.11E-09 |
| PA14_61400 | central_intermediary_metab | PGD1660844 | mqoB | DcbrA vs WT | 1.284472 | 1.84E-13 |
| PA14_61520 | hypothetical_unclassified | PGD1660864 | NA | DcbrA vs WT | -1.09222 | 2.89E-05 |
| PA14_61530 | motility_and_attachment | PGD1660866 | NA | DcbrA vs WT | -2.08634 | 7.32E-16 |
| PA14_61540 | motility_and_attachment | PGD1660868 | NA | DcbrA vs WT | -1.10931 | 2.24E-06 |
| PA14_61600 | putative_enzymes | PGD1660878 | NA | DcbrA vs WT | -1.05498 | 5.64E-07 |
| PA14_61620 | transcriptional_regulators | PGD1660882 | NA | DcbrA vs WT | -1.48999 | 6.02E-04 |
| PA14_61650 | cell_wall_LPS | PGD1660886 | pagL | DcbrA vs WT | 1.247016 | 2.02E-14 |
| PA14_61670 | biosynthesis_of_cofactors | PGD1660890 | moeB | DcbrA vs WT | -1.04748 | 7.35E-06 |
| NA | NA | PGD1660915 | NA | DcbrA vs WT | -1.55679 | 0.001239 |
| PA14_61890 | hypothetical_unclassified | PGD1660926 | NA | DcbrA vs WT | -1.83321 | 8.46E-11 |
| PA14_61950 | hypothetical_unclassified | PGD1660934 | NA | DcbrA vs WT | 1.230004 | 1.46E-10 |
| PA14_62100 | membrane_proteins | PGD1660962 | NA | DcbrA vs WT | 1.043114 | 0.001021 |
| PA14_62160 | amino_acid_biosynthesis_me | PGD1660972 | ilvI | DcbrA vs WT | 1.002686 | 8.91E-12 |
| PA14_62160 | biosynthesis_of_cofactors | PGD1660972 | ilvI | DcbrA vs WT | 1.002686 | 8.91E-12 |
| PA14_62240 | hypothetical_unclassified | PGD1660984 | NA | DcbrA vs WT | -1.93275 | 3.00E-18 |
| PA14_62290 | transport_of_small_molecule | PGD1660994 | NA | DcbrA vs WT | -1.4772 | 5.29E-08 |
| PA14_62350 | transport_of_small_molecule | PGD1661000 | NA | DcbrA vs WT | 1.160324 | 3.25E-10 |
| PA14_62360 | transport_of_small_molecule | PGD1661002 | NA | DcbrA vs WT | -1.44089 | 2.04E-09 |
| PA14_62380 | hypothetical_unclassified | PGD1661006 | NA | DcbrA vs WT | 1.200078 | 2.66E-23 |
| PA14_62390 | transport_of_small_molecule | PGD1661008 | NA | DcbrA vs WT | -1.02381 | 6.01E-11 |
| PA14_62530 | carbon_compound_catabolis | PGD1661032 | cbrA | DcbrA vs WT | -1.56215 | 0.007495 |
| PA14_62530 | two_component_regulators | PGD1661032 | cbrA | DcbrA vs WT | -1.56215 | 0.007495 |
| PA14_62570 | biosynthesis_of_cofactors | PGD1661038 | folK | DcbrA vs WT | -1.2151 | 3.45E-06 |
| PA14_62670 | hypothetical_unclassified | PGD1661056 | NA | DcbrA vs WT | -1.35178 | 3.93E-10 |
| PA14_62680 | hypothetical_unclassified | PGD1661058 | NA | DcbrA vs WT | 1.404113 | 1.13E-05 |
| PA14_62690 | hypothetical_unclassified | PGD1661060 | NA | DcbrA vs WT | 1.488046 | 5.73E-07 |

| | | | | | | |
|---|---|---|---|---|---|---|
| PA14_62840 | cell_wall_LPS | PGD1661084 | glmM | DcbrA vs WT | 1.103371 | 5.93E-18 |
| PA14_62990 | chaperones_heat_shock | PGD1661110 | grpE | DcbrA vs WT | 1.044887 | 1.08E-08 |
| PA14_62990 | DNA_replication_recombinat | PGD1661110 | grpE | DcbrA vs WT | 1.044887 | 1.08E-08 |
| PA14_63190 | membrane_proteins | PGD1661144 | NA | DcbrA vs WT | -1.1144 | 2.40E-05 |
| PA14_63210 | two_component_regulators | PGD1661148 | NA | DcbrA vs WT | -1.57603 | 5.60E-15 |
| PA14_63230 | transport_of_small_molecule | PGD1661152 | NA | DcbrA vs WT | 1.151176 | 1.93E-04 |
| PA14_63250 | fatty_acid_and_phospholipid | PGD1661156 | NA | DcbrA vs WT | 1.232952 | 1.81E-14 |
| PA14_63250 | putative_enzymes | PGD1661156 | NA | DcbrA vs WT | 1.232952 | 1.81E-14 |
| PA14_63280 | transcriptional_regulators | PGD1661160 | NA | DcbrA vs WT | -1.40295 | 3.38E-20 |
| PA14_63330 | fatty_acid_and_phospholipid | PGD1661170 | NA | DcbrA vs WT | -1.27366 | 7.64E-10 |
| PA14_63340 | fatty_acid_and_phospholipid | PGD1661172 | NA | DcbrA vs WT | -1.02681 | 5.02E-12 |
| PA14_63440 | relative_phage_transposon | PGD1661190 | NA | DcbrA vs WT | 1.439474 | 3.88E-06 |
| PA14_63480 | membrane_proteins | PGD1661198 | NA | DcbrA vs WT | -1.37408 | 2.49E-06 |
| PA14_63480 | protein_secretion_export | PGD1661198 | NA | DcbrA vs WT | -1.37408 | 2.49E-06 |
| PA14_63480 | transport_of_small_molecule | PGD1661198 | NA | DcbrA vs WT | -1.37408 | 2.49E-06 |
| PA14_63530 | translation_posttranslational | PGD1661204 | selB | DcbrA vs WT | -1.08881 | 3.00E-06 |
| PA14_63570 | energy_metabolism_CAP | PGD1661210 | fdnI | DcbrA vs WT | 1.016568 | 4.18E-13 |
| PA14_63640 | fatty_acid_and_phospholipid | PGD1661218 | fadH2 | DcbrA vs WT | -2.90778 | 2.34E-04 |
| PA14_63700 | hypothetical_unclassified | PGD1661226 | NA | DcbrA vs WT | -1.77097 | 0.005922 |
| PA14_63730 | membrane_proteins | PGD1661232 | NA | DcbrA vs WT | -1.18088 | 1.70E-04 |
| PA14_63730 | transport_of_small_molecule | PGD1661232 | NA | DcbrA vs WT | -1.18088 | 1.70E-04 |
| PA14_63740 | hypothetical_unclassified | PGD1661234 | NA | DcbrA vs WT | -1.12851 | 0.009845 |
| PA14_63780 | hypothetical_unclassified | PGD1661240 | NA | DcbrA vs WT | -2.11587 | 0.03233 |
| PA14_63840 | membrane_proteins | PGD1661248 | NA | DcbrA vs WT | -1.17633 | 3.69E-04 |
| PA14_63860 | hypothetical_unclassified | PGD1661252 | NA | DcbrA vs WT | -1.38058 | 7.03E-06 |
| PA14_63880 | transcriptional_regulators | PGD1661254 | NA | DcbrA vs WT | -1.04366 | 1.12E-04 |
| PA14_63960 | membrane_proteins | PGD1661266 | cntO | DcbrA vs WT | -1.22782 | 9.11E-05 |
| PA14_64080 | chaperones_heat_shock | PGD1661282 | dipZ | DcbrA vs WT | -1.09926 | 1.34E-14 |
| PA14_64080 | translation_posttranslational | PGD1661282 | dipZ | DcbrA vs WT | -1.09926 | 1.34E-14 |
| PA14_64270 | transport_of_small_molecule | PGD1661310 | NA | DcbrA vs WT | 1.776539 | 3.83E-04 |
| PA14_64320 | transport_of_small_molecule | PGD1661320 | NA | DcbrA vs WT | -1.72418 | 2.27E-19 |
| PA14_64335 | central_intermediary_metab | PGD1661322 | ureD | DcbrA vs WT | -2.3358 | 1.37E-04 |
| PA14_64360 | transport_of_small_molecule | PGD1661326 | NA | DcbrA vs WT | -1.68486 | 8.13E-05 |
| PA14_64370 | central_intermediary_metab | PGD1661328 | ureB | DcbrA vs WT | -1.60104 | 8.50E-07 |
| PA14_64390 | central_intermediary_metab | PGD1661330 | ureC | DcbrA vs WT | -1.41068 | 4.66E-11 |
| PA14_64420 | hypothetical_unclassified | PGD1661336 | NA | DcbrA vs WT | -2.9345 | 1.08E-04 |
| PA14_64430 | hypothetical_unclassified | PGD1661338 | NA | DcbrA vs WT | -1.80978 | 2.13E-12 |
| PA14_64460 | adaptation_and_protection | PGD1661344 | NA | DcbrA vs WT | -1.27958 | 1.95E-17 |
| PA14_64480 | membrane_proteins | PGD1661348 | osmE | DcbrA vs WT | 2.250601 | 7.48E-30 |
| PA14_64520 | central_intermediary_metab | PGD1661356 | NA | DcbrA vs WT | 2.367059 | 2.23E-50 |
| PA14_64560 | hypothetical_unclassified | PGD1661364 | NA | DcbrA vs WT | -3.74619 | 1.57E-04 |
| PA14_64620 | energy_metabolism_CAP | PGD1661374 | NA | DcbrA vs WT | -1.86543 | 2.15E-06 |
| PA14_64620 | putative_enzymes | PGD1661374 | NA | DcbrA vs WT | -1.86543 | 2.15E-06 |
| PA14_64640 | transcriptional_regulators | PGD1661376 | NA | DcbrA vs WT | -1.54814 | 1.20E-06 |
| PA14_64690 | membrane_proteins | PGD1661386 | NA | DcbrA vs WT | -2.27738 | 8.07E-05 |
| PA14_64750 | membrane_proteins | PGD1661396 | NA | DcbrA vs WT | 2.356539 | 1.07E-16 |
| PA14_64750 | transport_of_small_molecule | PGD1661396 | NA | DcbrA vs WT | 2.356539 | 1.07E-16 |
| PA14_64790 | membrane_proteins | PGD1661402 | NA | DcbrA vs WT | -2.79419 | 3.31E-07 |
| PA14_64790 | transport_of_small_molecule | PGD1661402 | NA | DcbrA vs WT | -2.79419 | 3.31E-07 |
| PA14_64810 | carbon_compound_catabolis | PGD1661406 | vanB | DcbrA vs WT | -1.29939 | 9.67E-04 |

| | | | | | | |
|---|---|---|---|---|---|---|
| PA14_64850 | amino_acid_biosynthesis_me | PGD1661412 | NA | DcbrA vs WT | -1.69155 | 1.25E-05 |
| PA14_64860 | transport_of_small_molecule | PGD1661414 | NA | DcbrA vs WT | -1.73493 | 1.08E-07 |
| PA14_64880 | membrane_proteins | PGD1661418 | NA | DcbrA vs WT | -1.00196 | 0.004944 |
| PA14_64880 | transport_of_small_molecule | PGD1661418 | NA | DcbrA vs WT | -1.00196 | 0.004944 |
| PA14_64930 | nucleotide_biosynthesis_and | PGD1661428 | NA | DcbrA vs WT | -1.41521 | 3.43E-14 |
| PA14_64940 | biosynthesis_of_cofactors | PGD1661430 | NA | DcbrA vs WT | -2.44422 | 7.60E-17 |
| PA14_64960 | biosynthesis_of_cofactors | PGD1661434 | pncB1 | DcbrA vs WT | 1.280476 | 1.43E-10 |
| PA14_64990 | fatty_acid_and_phospholipid | PGD1661438 | NA | DcbrA vs WT | -1.33778 | 8.08E-05 |
| PA14_65150 | translation_posttranslational | PGD1661460 | rplI | DcbrA vs WT | -1.382 | 1.39E-11 |
| PA14_65320 | adaptation_and_protection | PGD1661490 | miaA | DcbrA vs WT | 1.073873 | 3.74E-08 |
| PA14_65320 | translation_posttranslational | PGD1661490 | miaA | DcbrA vs WT | 1.073873 | 3.74E-08 |
| PA14_65380 | putative_enzymes | PGD1661496 | NA | DcbrA vs WT | 1.184608 | 6.20E-08 |
| PA14_65580 | membrane_proteins | PGD1661524 | NA | DcbrA vs WT | -1.39262 | 4.29E-05 |
| PA14_65640 | hypothetical_unclassified | PGD1661532 | NA | DcbrA vs WT | -1.76615 | 2.11E-14 |
| PA14_65795 | amino_acid_biosynthesis_me | PGD1661554 | NA | DcbrA vs WT | -1.40651 | 1.24E-04 |
| PA14_65820 | fatty_acid_and_phospholipid | PGD1661558 | NA | DcbrA vs WT | -1.3209 | 0.011404 |
| PA14_65820 | putative_enzymes | PGD1661558 | NA | DcbrA vs WT | -1.3209 | 0.011404 |
| PA14_65870 | transport_of_small_molecule | PGD1661566 | NA | DcbrA vs WT | -1.31479 | 2.25E-06 |
| PA14_65920 | transport_of_small_molecule | PGD1661572 | NA | DcbrA vs WT | -1.43387 | 0.009103 |
| PA14_65940 | energy_metabolism_CAP | PGD1661574 | NA | DcbrA vs WT | -1.47056 | 5.74E-08 |
| PA14_65940 | putative_enzymes | PGD1661574 | NA | DcbrA vs WT | -1.47056 | 5.74E-08 |
| PA14_65960 | cell_wall_LPS | PGD1661578 | waaA | DcbrA vs WT | -2.47084 | 1.14E-18 |
| PA14_66000 | energy_metabolism_CAP | PGD1661584 | NA | DcbrA vs WT | -1.01921 | 7.35E-04 |
| PA14_66420 | membrane_proteins | PGD1661650 | NA | DcbrA vs WT | -2.17814 | 3.48E-17 |
| PA14_66440 | amino_acid_biosynthesis_me | PGD1661652 | metY | DcbrA vs WT | -1.5969 | 1.03E-17 |
| PA14_66460 | adaptation_and_protection | PGD1661656 | NA | DcbrA vs WT | -2.35444 | 3.39E-21 |
| PA14_66530 | transcriptional_regulators | PGD1661666 | NA | DcbrA vs WT | -2.04782 | 3.59E-07 |
| PA14_66760 | cell_division | PGD1661706 | NA | DcbrA vs WT | -1.35121 | 1.49E-09 |
| PA14_66770 | chaperones_heat_shock | PGD1661708 | hslV | DcbrA vs WT | 1.955947 | 3.75E-28 |
| PA14_66820 | central_intermediary_metab | PGD1661714 | phaC1 | DcbrA vs WT | 1.051738 | 6.48E-10 |
| PA14_66910 | hypothetical_unclassified | PGD1661730 | NA | DcbrA vs WT | -1.68964 | 4.59E-10 |
| PA14_66970 | protein_secretion_export | PGD1661740 | tatB | DcbrA vs WT | -1.07668 | 7.14E-04 |
| PA14_67065 | cell_wall_LPS | PGD1661756 | mdoH | DcbrA vs WT | 1.028672 | 7.83E-21 |
| PA14_67090 | putative_enzymes | PGD1661758 | mdoG | DcbrA vs WT | 1.312381 | 3.89E-52 |
| PA14_67140 | translation_posttranslational | PGD1661768 | NA | DcbrA vs WT | -1.41504 | 0.003706 |
| PA14_67240 | amino_acid_biosynthesis_me | PGD1661786 | hutG | DcbrA vs WT | -1.70673 | 8.69E-19 |
| PA14_67250 | amino_acid_biosynthesis_me | PGD1661788 | hutI | DcbrA vs WT | -2.17408 | 6.34E-18 |
| PA14_67260 | central_intermediary_metab | PGD1661790 | NA | DcbrA vs WT | -1.77971 | 6.77E-16 |
| PA14_67280 | transport_of_small_molecule | PGD1661794 | NA | DcbrA vs WT | -1.44541 | 4.65E-17 |
| PA14_67300 | transport_of_small_molecule | PGD1661796 | NA | DcbrA vs WT | -1.0294 | 1.66E-09 |
| PA14_67310 | transport_of_small_molecule | PGD1661798 | NA | DcbrA vs WT | -1.78176 | 2.79E-13 |
| PA14_67320 | amino_acid_biosynthesis_me | PGD1661800 | hutH | DcbrA vs WT | -2.63436 | 4.42E-18 |
| PA14_67320 | central_intermediary_metab | PGD1661800 | hutH | DcbrA vs WT | -2.63436 | 4.42E-18 |
| PA14_67340 | transport_of_small_molecule | PGD1661802 | NA | DcbrA vs WT | -2.54079 | 1.16E-34 |
| PA14_67350 | amino_acid_biosynthesis_me | PGD1661804 | hutU | DcbrA vs WT | -1.99694 | 3.96E-19 |
| PA14_67370 | transport_of_small_molecule | PGD1661806 | NA | DcbrA vs WT | -1.51503 | 1.79E-11 |
| PA14_67440 | putative_enzymes | PGD1661816 | NA | DcbrA vs WT | -1.1705 | 3.36E-13 |
| PA14_67460 | fatty_acid_and_phospholipid | PGD1661820 | NA | DcbrA vs WT | 1.187953 | 1.98E-19 |
| PA14_67490 | carbon_compound_catabolis | PGD1661824 | fbp | DcbrA vs WT | 1.176246 | 2.55E-23 |
| PA14_67490 | central_intermediary_metab | PGD1661824 | fbp | DcbrA vs WT | 1.176246 | 2.55E-23 |

| | | | | | | |
|---|---|---|---|---|---|---|
| PA14_67500 | central_intermediary_metab | PGD1661826 | gloA3 | DcbrA vs WT | 1.644988 | 1.15E-29 |
| PA14_67540 | hypothetical_unclassified | PGD1661834 | NA | DcbrA vs WT | -2.26607 | 1.88E-09 |
| PA14_67620 | hypothetical_unclassified | PGD1661844 | NA | DcbrA vs WT | -1.67343 | 2.08E-08 |
| PA14_67670 | two_component_regulators | PGD1661852 | ntrB | DcbrA vs WT | 1.068461 | 1.44E-07 |
| PA14_67820 | putative_enzymes | PGD1661874 | NA | DcbrA vs WT | -1.16554 | 3.79E-05 |
| PA14_67830 | hypothetical_unclassified | PGD1661876 | NA | DcbrA vs WT | -1.03132 | 5.66E-11 |
| PA14_68100 | hypothetical_unclassified | PGD1661922 | NA | DcbrA vs WT | -1.06689 | 0.003099 |
| PA14_68110 | antibiotic_resistance_and_su | PGD1661924 | NA | DcbrA vs WT | 1.540593 | 5.76E-09 |
| PA14_68120 | cell_wall_LPS | PGD1661926 | NA | DcbrA vs WT | -1.03806 | 4.18E-04 |
| PA14_68200 | cell_wall_LPS | PGD1661938 | rmlA | DcbrA vs WT | 1.229798 | 1.32E-16 |
| PA14_68260 | membrane_proteins | PGD1661946 | NA | DcbrA vs WT | 1.227429 | 2.06E-08 |
| PA14_68330 | amino_acid_biosynthesis_me | PGD1661954 | arcA | DcbrA vs WT | -1.40544 | 3.04E-07 |
| PA14_68350 | amino_acid_biosynthesis_me | PGD1661958 | arcC | DcbrA vs WT | -1.80957 | 6.18E-12 |
| PA14_68370 | putative_enzymes | PGD1661962 | cysQ | DcbrA vs WT | -1.13641 | 2.53E-10 |
| PA14_68370 | transport_of_small_molecule | PGD1661962 | cysQ | DcbrA vs WT | -1.13641 | 2.53E-10 |
| PA14_68400 | cell_wall_LPS | PGD1661968 | NA | DcbrA vs WT | 1.263109 | 1.16E-15 |
| PA14_68430 | putative_enzymes | PGD1661972 | NA | DcbrA vs WT | -1.21693 | 4.98E-12 |
| PA14_68440 | energy_metabolism_CAP | PGD1661974 | NA | DcbrA vs WT | -1.31459 | 5.67E-10 |
| PA14_68440 | putative_enzymes | PGD1661974 | NA | DcbrA vs WT | -1.31459 | 5.67E-10 |
| PA14_68450 | membrane_proteins | PGD1661976 | NA | DcbrA vs WT | 1.114783 | 1.28E-04 |
| PA14_68660 | transcription_RNA_processin | PGD1662008 | rimK | DcbrA vs WT | 1.616521 | 2.03E-30 |
| PA14_68660 | translation_posttranslational | PGD1662008 | rimK | DcbrA vs WT | 1.616521 | 2.03E-30 |
| PA14_68720 | hypothetical_unclassified | PGD1662018 | NA | DcbrA vs WT | -2.27126 | 2.50E-05 |
| PA14_68955 | putative_enzymes | PGD1662056 | NA | DcbrA vs WT | -1.22765 | 1.15E-07 |
| PA14_68980 | biosynthesis_of_cofactors | PGD1662060 | ubiH | DcbrA vs WT | -1.17862 | 7.27E-10 |
| PA14_68980 | energy_metabolism_CAP | PGD1662060 | ubiH | DcbrA vs WT | -1.17862 | 7.27E-10 |
| PA14_69090 | membrane_proteins | PGD1662078 | NA | DcbrA vs WT | -1.14425 | 5.30E-08 |
| PA14_69200 | energy_metabolism_CAP | PGD1662094 | trxA | DcbrA vs WT | 1.068163 | 2.22E-08 |
| PA14_69200 | nucleotide_biosynthesis_and | PGD1662094 | trxA | DcbrA vs WT | 1.068163 | 2.22E-08 |
| PA14_69200 | translation_posttranslational | PGD1662094 | trxA | DcbrA vs WT | 1.068163 | 2.22E-08 |
| PA14_69320 | membrane_proteins | PGD1662114 | NA | DcbrA vs WT | 1.428676 | 1.75E-33 |
| PA14_69330 | membrane_proteins | PGD1662116 | NA | DcbrA vs WT | 1.01472 | 6.94E-17 |
| PA14_69380 | chaperones_heat_shock | PGD1662124 | NA | DcbrA vs WT | 1.099057 | 5.94E-09 |
| PA14_69380 | translation_posttranslational | PGD1662124 | NA | DcbrA vs WT | 1.099057 | 5.94E-09 |
| PA14_69390 | transcriptional_regulators | PGD1662126 | algQ | DcbrA vs WT | 1.066082 | 6.20E-12 |
| PA14_69560 | secreted_factors | PGD1662152 | hcpB | DcbrA vs WT | -1.14276 | 8.34E-08 |
| PA14_69600 | hypothetical_unclassified | PGD1662160 | NA | DcbrA vs WT | -1.6483 | 8.17E-16 |
| PA14_69630 | transcriptional_regulators | PGD1662166 | rnk | DcbrA vs WT | -1.03904 | 1.65E-10 |
| PA14_69770 | transcriptional_regulators | PGD1662188 | sutA | DcbrA vs WT | -1.43894 | 6.87E-24 |
| PA14_69810 | antibiotic_resistance_and_su | PGD1662194 | glnK | DcbrA vs WT | 1.138342 | 5.86E-15 |
| PA14_69810 | central_intermediary_metab | PGD1662194 | glnK | DcbrA vs WT | 1.138342 | 5.86E-15 |
| PA14_69850 | transport_of_small_molecule | PGD1662200 | NA | DcbrA vs WT | 1.7115 | 7.97E-45 |
| PA14_69880 | transcriptional_regulators | PGD1662204 | NA | DcbrA vs WT | -1.68394 | 4.27E-08 |
| PA14_69890 | antibiotic_resistance_and_su | PGD1662206 | NA | DcbrA vs WT | -1.13552 | 0.004044 |
| PA14_70010 | translation_posttranslational | PGD1662224 | NA | DcbrA vs WT | -1.25088 | 6.77E-16 |
| PA14_70050 | hypothetical_unclassified | PGD1662228 | NA | DcbrA vs WT | -1.03886 | 1.89E-10 |
| PA14_70180 | translation_posttranslational | PGD1662248 | rpmG | DcbrA vs WT | -1.14986 | 1.35E-07 |
| PA14_70400 | hypothetical_unclassified | PGD1662284 | NA | DcbrA vs WT | 1.639992 | 4.42E-32 |
| PA14_70480 | translation_posttranslational | PGD1662296 | NA | DcbrA vs WT | 1.216654 | 3.31E-14 |
| PA14_70510 | transport_of_small_molecule | PGD1662300 | NA | DcbrA vs WT | -1.37659 | 0.013805 |

| | | | | | | |
|---|---|---|---|---|---|---|
| PA14_70550 | carbon_compound_catabolis | PGD1662304 | NA | DcbrA vs WT | -1.40106 | 3.83E-11 |
| PA14_70590 | hypothetical_unclassified | PGD1662312 | NA | DcbrA vs WT | 1.427866 | 8.37E-15 |
| PA14_70640 | carbon_compound_catabolis | PGD1662320 | rubA1 | DcbrA vs WT | -1.68802 | 1.27E-09 |
| PA14_70650 | putative_enzymes | PGD1662322 | NA | DcbrA vs WT | -2.76582 | 3.04E-11 |
| PA14_70670 | carbon_compound_catabolis | PGD1662324 | glcF | DcbrA vs WT | -3.73331 | 1.04E-25 |
| PA14_70670 | central_intermediary_metab | PGD1662324 | glcF | DcbrA vs WT | -3.73331 | 1.04E-25 |
| PA14_70670 | energy_metabolism_CAP | PGD1662324 | glcF | DcbrA vs WT | -3.73331 | 1.04E-25 |
| PA14_70680 | carbon_compound_catabolis | PGD1662326 | glcE | DcbrA vs WT | -3.58693 | 2.01E-30 |
| PA14_70680 | central_intermediary_metab | PGD1662326 | glcE | DcbrA vs WT | -3.58693 | 2.01E-30 |
| PA14_70690 | carbon_compound_catabolis | PGD1662328 | glcD | DcbrA vs WT | -2.26704 | 2.51E-21 |
| PA14_70690 | central_intermediary_metab | PGD1662328 | glcD | DcbrA vs WT | -2.26704 | 2.51E-21 |
| PA14_70720 | biosynthesis_of_cofactors | PGD1662332 | ubiC | DcbrA vs WT | -1.91854 | 1.49E-18 |
| PA14_70720 | central_intermediary_metab | PGD1662332 | ubiC | DcbrA vs WT | -1.91854 | 1.49E-18 |
| PA14_70950 | adaptation_and_protection | PGD1662374 | betB | DcbrA vs WT | 1.440396 | 1.74E-07 |
| PA14_70950 | amino_acid_biosynthesis_me | PGD1662374 | betB | DcbrA vs WT | 1.440396 | 1.74E-07 |
| PA14_71080 | hypothetical_unclassified | PGD1662390 | NA | DcbrA vs WT | -1.38093 | 1.90E-06 |
| PA14_71100 | membrane_proteins | PGD1662394 | NA | DcbrA vs WT | -4.33225 | 2.55E-12 |
| PA14_71170 | transcriptional_regulators | PGD1662406 | NA | DcbrA vs WT | -1.0547 | 4.00E-04 |
| PA14_71250 | hypothetical_unclassified | PGD1662422 | NA | DcbrA vs WT | -1.82637 | 0.006326 |
| PA14_71280 | amino_acid_biosynthesis_me | PGD1662426 | NA | DcbrA vs WT | -2.28158 | 5.06E-08 |
| PA14_71280 | energy_metabolism_CAP | PGD1662426 | NA | DcbrA vs WT | -2.28158 | 5.06E-08 |
| PA14_71300 | energy_metabolism_CAP | PGD1662428 | NA | DcbrA vs WT | -3.53055 | 8.17E-05 |
| PA14_71350 | hypothetical_unclassified | PGD1662438 | NA | DcbrA vs WT | -3.45415 | 0.018154 |
| PA14_71380 | hypothetical_unclassified | PGD1662444 | NA | DcbrA vs WT | -1.34768 | 8.06E-08 |
| PA14_71390 | membrane_proteins | PGD1662446 | NA | DcbrA vs WT | -1.40666 | 3.39E-12 |
| PA14_71400 | hypothetical_unclassified | PGD1662448 | NA | DcbrA vs WT | -3.22176 | 5.78E-08 |
| PA14_71410 | transport_of_small_molecule | PGD1662450 | NA | DcbrA vs WT | -2.43111 | 2.31E-04 |
| PA14_71450 | hypothetical_unclassified | PGD1662458 | NA | DcbrA vs WT | -1.1992 | 4.55E-06 |
| PA14_71460 | amino_acid_biosynthesis_me | PGD1662460 | glyA1 | DcbrA vs WT | -2.42227 | 1.05E-05 |
| PA14_71470 | amino_acid_biosynthesis_me | PGD1662462 | soxB | DcbrA vs WT | -1.45554 | 1.06E-04 |
| PA14_71470 | carbon_compound_catabolis | PGD1662462 | soxB | DcbrA vs WT | -1.45554 | 1.06E-04 |
| PA14_71490 | amino_acid_biosynthesis_me | PGD1662464 | soxD | DcbrA vs WT | -4.37543 | 0.001918 |
| PA14_71490 | carbon_compound_catabolis | PGD1662464 | soxD | DcbrA vs WT | -4.37543 | 0.001918 |
| PA14_71500 | carbon_compound_catabolis | PGD1662466 | soxA | DcbrA vs WT | -2.01275 | 5.98E-08 |
| PA14_71510 | amino_acid_biosynthesis_me | PGD1662468 | soxG | DcbrA vs WT | -1.49281 | 0.005283 |
| PA14_71510 | carbon_compound_catabolis | PGD1662468 | soxG | DcbrA vs WT | -1.49281 | 0.005283 |
| PA14_71530 | nucleotide_biosynthesis_and | PGD1662470 | purU2 | DcbrA vs WT | -1.26934 | 0.012249 |
| PA14_71560 | amino_acid_biosynthesis_me | PGD1662472 | fdhA | DcbrA vs WT | -2.49594 | 8.65E-10 |
| PA14_71580 | hypothetical_unclassified | PGD1662476 | NA | DcbrA vs WT | 1.014985 | 4.41E-07 |
| PA14_71600 | nucleotide_biosynthesis_and | PGD1662480 | purK | DcbrA vs WT | -1.49405 | 1.41E-11 |
| PA14_71630 | carbon_compound_catabolis | PGD1662484 | adhA | DcbrA vs WT | -2.21294 | 1.71E-20 |
| PA14_71700 | transport_of_small_molecule | PGD1662496 | NA | DcbrA vs WT | -1.29884 | 0.029401 |
| PA14_71720 | central_intermediary_metab | PGD1662500 | NA | DcbrA vs WT | -1.32848 | 1.92E-09 |
| PA14_71740 | central_intermediary_metab | PGD1662502 | NA | DcbrA vs WT | -2.09271 | 2.64E-18 |
| PA14_71760 | hypothetical_unclassified | PGD1662506 | NA | DcbrA vs WT | -1.29406 | 7.34E-05 |
| PA14_71890 | fatty_acid_and_phospholipid | PGD1662524 | NA | DcbrA vs WT | 1.020205 | 1.17E-08 |
| PA14_71890 | putative_enzymes | PGD1662524 | NA | DcbrA vs WT | 1.020205 | 1.17E-08 |
| PA14_71900 | hypothetical_unclassified | PGD1662526 | NA | DcbrA vs WT | -1.62914 | 8.28E-18 |
| PA14_72030 | hypothetical_unclassified | PGD1662548 | NA | DcbrA vs WT | -1.58527 | 2.19E-17 |
| PA14_72200 | translation_posttranslational | PGD1662574 | prfH | DcbrA vs WT | -1.91875 | 0.018482 |

| | | | | | | |
|---|---|---|---|---|---|---|
| PA14_72260 | transport_of_small_molecule | PGD1662584 | NA | DcbrA vs WT | -1.66186 | 2.21E-11 |
| PA14_72280 | transport_of_small_molecule | PGD1662586 | citA | DcbrA vs WT | 2.101339 | 5.42E-22 |
| PA14_72300 | membrane_proteins | PGD1662588 | NA | DcbrA vs WT | 1.487255 | 1.14E-16 |
| PA14_72340 | transport_of_small_molecule | PGD1662592 | gltP | DcbrA vs WT | 1.122761 | 1.14E-10 |
| PA14_72360 | hypothetical_unclassified | PGD1662596 | NA | DcbrA vs WT | 1.079326 | 1.66E-04 |
| PA14_72370 | membrane_proteins | PGD1662598 | NA | DcbrA vs WT | 1.986276 | 1.11E-09 |
| PA14_72390 | two_component_regulators | PGD1662602 | NA | DcbrA vs WT | 1.224941 | 8.07E-16 |
| PA14_72410 | antibiotic_resistance_and_su | PGD1662606 | NA | DcbrA vs WT | -1.07303 | 4.34E-05 |
| PA14_72460 | energy_metabolism_CAP | PGD1662614 | cc4 | DcbrA vs WT | -1.61606 | 6.78E-19 |
| PA14_72500 | hypothetical_unclassified | PGD1662622 | NA | DcbrA vs WT | -1.50002 | 4.17E-09 |
| PA14_72550 | motility_and_attachment | PGD1662630 | NA | DcbrA vs WT | -1.08452 | 1.97E-11 |
| PA14_72550 | transport_of_small_molecule | PGD1662630 | NA | DcbrA vs WT | -1.08452 | 1.97E-11 |
| PA14_72600 | fatty_acid_and_phospholipid | PGD1662638 | NA | DcbrA vs WT | -1.39137 | 1.12E-14 |
| PA14_72650 | transcriptional_regulators | PGD1662646 | NA | DcbrA vs WT | -1.70301 | 2.95E-05 |
| PA14_72690 | amino_acid_biosynthesis_me | PGD1662650 | NA | DcbrA vs WT | -2.12051 | 3.14E-06 |
| PA14_72760 | adaptation_and_protection | PGD1662662 | NA | DcbrA vs WT | 1.7935 | 4.46E-29 |
| PA14_72760 | antibiotic_resistance_and_su | PGD1662662 | NA | DcbrA vs WT | 1.7935 | 4.46E-29 |
| PA14_72760 | putative_enzymes | PGD1662662 | NA | DcbrA vs WT | 1.7935 | 4.46E-29 |
| PA14_72780 | biosynthesis_of_cofactors | PGD1662666 | pdxY | DcbrA vs WT | 1.034064 | 1.86E-16 |
| PA14_72870 | biosynthesis_of_cofactors | PGD1662682 | NA | DcbrA vs WT | 1.757435 | 1.17E-53 |
| PA14_72870 | putative_enzymes | PGD1662682 | NA | DcbrA vs WT | 1.757435 | 1.17E-53 |
| PA14_72900 | fatty_acid_and_phospholipid | PGD1662688 | NA | DcbrA vs WT | 1.829525 | 1.73E-25 |
| PA14_72960 | transport_of_small_molecule | PGD1662696 | NA | DcbrA vs WT | 1.634703 | 5.43E-06 |
| PA14_72990 | hypothetical_unclassified | PGD1662702 | NA | DcbrA vs WT | 1.005857 | 8.99E-09 |
| PA14_73000 | hypothetical_unclassified | PGD1662704 | NA | DcbrA vs WT | -1.02856 | 0.023347 |
| PA14_73060 | transport_of_small_molecule | PGD1662716 | NA | DcbrA vs WT | 1.219841 | 1.03E-05 |
| PA14_73100 | hypothetical_unclassified | PGD1662722 | NA | DcbrA vs WT | -2.35439 | 0.00135 |
| PA14_73390 | hypothetical_unclassified | PGD1662768 | NA | DcbrA vs WT | 1.0773 | 0.006697 |
| NA | NA | PGD55179284 | NA | DcbrA vs WT | 1.080912 | 2.38E-05 |
| NA | NA | PGD55179286 | NA | DcbrA vs WT | 1.242431 | 1.26E-04 |
| PA14_00080 | membrane_proteins | PGD1650847 | NA | DrhlR vs WT | -1.28052 | 3.05E-16 |
| PA14_00210 | putative_enzymes | PGD1650873 | NA | DrhlR vs WT | 1.197144 | 6.05E-11 |
| PA14_00230 | DNA_replication_recombinat | PGD1650875 | NA | DrhlR vs WT | 1.094483 | 0.003053 |
| PA14_00230 | membrane_proteins | PGD1650875 | NA | DrhlR vs WT | 1.094483 | 0.003053 |
| PA14_00470 | hypothetical_unclassified | PGD1650911 | NA | DrhlR vs WT | 1.297408 | 3.82E-13 |
| PA14_00640 | putative_enzymes | PGD1650939 | phzH | DrhlR vs WT | -4.50652 | 4.87E-32 |
| PA14_00650 | hypothetical_unclassified | PGD1650941 | NA | DrhlR vs WT | -1.72998 | 2.13E-29 |
| PA14_00700 | antibiotic_resistance_and_su | PGD1650951 | NA | DrhlR vs WT | -1.33672 | 0.012006 |
| PA14_00700 | biosynthesis_of_cofactors | PGD1650951 | NA | DrhlR vs WT | -1.33672 | 0.012006 |
| PA14_00710 | adaptation_and_protection | PGD1650953 | osmC | DrhlR vs WT | 1.039208 | 5.08E-04 |
| PA14_00720 | hypothetical_unclassified | PGD1650955 | NA | DrhlR vs WT | 1.081437 | 2.33E-15 |
| PA14_01490 | adaptation_and_protection | PGD1651083 | NA | DrhlR vs WT | -4.4383 | 1.74E-43 |
| PA14_01490 | putative_enzymes | PGD1651083 | NA | DrhlR vs WT | -4.4383 | 1.74E-43 |
| PA14_02130 | hypothetical_unclassified | PGD1651179 | NA | DrhlR vs WT | 1.051722 | 0.037936 |
| PA14_02180 | chemotaxis | PGD1651185 | cheB | DrhlR vs WT | -1.32715 | 9.72E-06 |
| PA14_02220 | chemotaxis | PGD1651191 | NA | DrhlR vs WT | -1.193 | 4.62E-12 |
| PA14_02380 | fatty_acid_and_phospholipid | PGD1651215 | NA | DrhlR vs WT | 1.115005 | 0.00453 |
| PA14_02380 | putative_enzymes | PGD1651215 | NA | DrhlR vs WT | 1.115005 | 0.00453 |
| PA14_02520 | hypothetical_unclassified | PGD1651237 | NA | DrhlR vs WT | -2.14065 | 2.29E-15 |
| PA14_02760 | carbon_compound_catabolis | PGD1651277 | NA | DrhlR vs WT | -2.26244 | 3.21E-16 |

| | | | | | | |
|---|---|---|---|---|---|---|
| PA14_02770 | carbon_compound_catabolis | PGD1651279 | NA | DrhlR vs WT | -2.87079 | 5.60E-13 |
| PA14_02790 | carbon_compound_catabolis | PGD1651281 | pcaF | DrhlR vs WT | -2.58714 | 1.60E-20 |
| PA14_02790 | fatty_acid_and_phospholipid | PGD1651281 | pcaF | DrhlR vs WT | -2.58714 | 1.60E-20 |
| PA14_02840 | carbon_compound_catabolis | PGD1651287 | pcaD | DrhlR vs WT | 1.102282 | 0.006298 |
| PA14_02900 | carbon_compound_catabolis | PGD1651295 | pcaK | DrhlR vs WT | -2.37738 | 4.95E-17 |
| PA14_02900 | membrane_proteins | PGD1651295 | pcaK | DrhlR vs WT | -2.37738 | 4.95E-17 |
| PA14_02900 | transport_of_small_molecule | PGD1651295 | pcaK | DrhlR vs WT | -2.37738 | 4.95E-17 |
| PA14_02910 | transcriptional_regulators | PGD1651297 | NA | DrhlR vs WT | -1.42359 | 2.50E-10 |
| PA14_02970 | membrane_proteins | PGD1651303 | NA | DrhlR vs WT | 2.642121 | 2.77E-17 |
| PA14_03050 | carbon_compound_catabolis | PGD1651319 | pobA | DrhlR vs WT | -1.18377 | 0.001415 |
| PA14_03110 | hypothetical_unclassified | PGD1651329 | NA | DrhlR vs WT | 3.297038 | 4.33E-21 |
| PA14_03450 | amino_acid_biosynthesis_me | PGD1651395 | gabT | DrhlR vs WT | 1.128999 | 9.14E-08 |
| PA14_03450 | carbon_compound_catabolis | PGD1651395 | gabT | DrhlR vs WT | 1.128999 | 9.14E-08 |
| PA14_03450 | central_intermediary_metab | PGD1651395 | gabT | DrhlR vs WT | 1.128999 | 9.14E-08 |
| PA14_03490 | carbon_compound_catabolis | PGD1651401 | NA | DrhlR vs WT | -3.68515 | 2.52E-97 |
| PA14_03510 | hypothetical_unclassified | PGD1651403 | NA | DrhlR vs WT | -3.65627 | 1.81E-38 |
| PA14_03520 | hypothetical_unclassified | PGD1651405 | NA | DrhlR vs WT | -3.71292 | 2.03E-40 |
| PA14_03855 | transport_of_small_molecule | PGD1651453 | NA | DrhlR vs WT | 1.639552 | 4.05E-13 |
| PA14_04040 | hypothetical_unclassified | PGD1651483 | NA | DrhlR vs WT | 1.031933 | 2.89E-15 |
| PA14_04180 | hypothetical_unclassified | PGD1651505 | NA | DrhlR vs WT | 1.517768 | 4.49E-04 |
| PA14_04290 | amino_acid_biosynthesis_me | PGD1651521 | NA | DrhlR vs WT | 1.008583 | 1.04E-08 |
| PA14_04290 | membrane_proteins | PGD1651521 | NA | DrhlR vs WT | 1.008583 | 1.04E-08 |
| PA14_04530 | hypothetical_unclassified | PGD1651559 | NA | DrhlR vs WT | 1.111833 | 4.84E-09 |
| PA14_04640 | transport_of_small_molecule | PGD1651575 | NA | DrhlR vs WT | 1.273199 | 4.92E-11 |
| PA14_04650 | translation_posttranslational | PGD1651577 | pfpI | DrhlR vs WT | 1.429884 | 5.87E-13 |
| PA14_04970 | biosynthesis_of_cofactors | PGD1651631 | NA | DrhlR vs WT | -1.14432 | 3.38E-04 |
| PA14_05050 | nucleotide_biosynthesis_and | PGD1651645 | NA | DrhlR vs WT | -1.17057 | 1.80E-08 |
| PA14_05070 | amino_acid_biosynthesis_me | PGD1651649 | NA | DrhlR vs WT | -1.24587 | 1.28E-07 |
| PA14_05110 | hypothetical_unclassified | PGD1651653 | NA | DrhlR vs WT | 1.85468 | 6.26E-23 |
| PA14_05340 | chemotaxis | PGD1651693 | pilI | DrhlR vs WT | 1.086258 | 6.62E-07 |
| PA14_05340 | motility_and_attachment | PGD1651693 | pilI | DrhlR vs WT | 1.086258 | 6.62E-07 |
| PA14_05360 | chemotaxis | PGD1651695 | pilJ | DrhlR vs WT | 1.21239 | 7.05E-12 |
| PA14_05510 | membrane_proteins | PGD1651719 | NA | DrhlR vs WT | 1.473221 | 4.10E-13 |
| PA14_05510 | secreted_factors | PGD1651719 | NA | DrhlR vs WT | 1.473221 | 4.10E-13 |
| PA14_05630 | hypothetical_unclassified | PGD1651739 | NA | DrhlR vs WT | 1.219994 | 3.23E-04 |
| PA14_05640 | membrane_proteins | PGD1651741 | NA | DrhlR vs WT | 1.191811 | 3.78E-05 |
| PA14_05660 | transcriptional_regulators | PGD1651745 | NA | DrhlR vs WT | -1.53568 | 2.09E-23 |
| PA14_05880 | membrane_proteins | PGD1651773 | NA | DrhlR vs WT | -1.49403 | 3.43E-07 |
| PA14_05890 | membrane_proteins | PGD1651775 | NA | DrhlR vs WT | -1.59707 | 3.62E-12 |
| PA14_06120 | amino_acid_biosynthesis_me | PGD1651805 | NA | DrhlR vs WT | -1.20646 | 5.31E-05 |
| PA14_06260 | transcriptional_regulators | PGD1651829 | NA | DrhlR vs WT | -1.73205 | 6.63E-14 |
| PA14_06350 | fatty_acid_and_phospholipid | PGD1651847 | NA | DrhlR vs WT | -1.20247 | 1.35E-05 |
| PA14_06360 | nucleotide_biosynthesis_and | PGD1651849 | NA | DrhlR vs WT | 1.692457 | 4.08E-07 |
| PA14_06360 | putative_enzymes | PGD1651849 | NA | DrhlR vs WT | 1.692457 | 4.08E-07 |
| PA14_06390 | hypothetical_unclassified | PGD1651851 | NA | DrhlR vs WT | 1.247876 | 8.95E-08 |
| PA14_06430 | putative_enzymes | PGD1651857 | NA | DrhlR vs WT | 1.206403 | 0.014227 |
| PA14_06500 | biosynthesis_of_cofactors | PGD1651865 | bioB | DrhlR vs WT | 1.271527 | 3.37E-19 |
| PA14_06740 | energy_metabolism_CAP | PGD1651901 | nirM | DrhlR vs WT | -1.26729 | 1.47E-05 |
| PA14_06770 | central_intermediary_metab | PGD1651905 | nirQ | DrhlR vs WT | -1.61477 | 8.60E-08 |
| PA14_06770 | energy_metabolism_CAP | PGD1651905 | nirQ | DrhlR vs WT | -1.61477 | 8.60E-08 |

| | | | | | | |
|---|---|---|---|---|---|---|
| PA14_06790 | energy_metabolism_CAP | PGD1651907 | NA | DrhlR vs WT | -1.83883 | 1.30E-08 |
| PA14_06800 | hypothetical_unclassified | PGD1651909 | NA | DrhlR vs WT | -1.97212 | 3.11E-06 |
| PA14_06840 | energy_metabolism_CAP | PGD1651915 | NA | DrhlR vs WT | -1.89973 | 1.30E-12 |
| NA | NA | PGD1651922 | NA | DrhlR vs WT | -1.86716 | 5.11E-04 |
| PA14_06890 | biosynthesis_of_cofactors | PGD1651925 | NA | DrhlR vs WT | 1.322074 | 1.58E-06 |
| PA14_06930 | transport_of_small_molecule | PGD1651931 | NA | DrhlR vs WT | 1.255958 | 3.45E-04 |
| PA14_06970 | transcriptional_regulators | PGD1651939 | NA | DrhlR vs WT | -1.30288 | 1.58E-16 |
| PA14_07200 | hypothetical_unclassified | PGD1651975 | NA | DrhlR vs WT | 1.055234 | 1.12E-11 |
| PA14_07370 | membrane_proteins | PGD1652003 | NA | DrhlR vs WT | 1.160613 | 3.43E-07 |
| PA14_07850 | transport_of_small_molecule | PGD1652079 | NA | DrhlR vs WT | 1.807391 | 1.75E-16 |
| PA14_08020 | relative_phage_transposon | PGD1652109 | NA | DrhlR vs WT | 1.697303 | 0.019396 |
| PA14_08030 | relative_phage_transposon | PGD1652111 | NA | DrhlR vs WT | 1.274903 | 0.007206 |
| PA14_08040 | relative_phage_transposon | PGD1652113 | NA | DrhlR vs WT | 2.137213 | 0.003113 |
| PA14_08150 | relative_phage_transposon | PGD1652133 | NA | DrhlR vs WT | 1.439619 | 0.002987 |
| PA14_08190 | hypothetical_unclassified | PGD1652139 | NA | DrhlR vs WT | 1.745089 | 0.039307 |
| PA14_08210 | relative_phage_transposon | PGD1652143 | NA | DrhlR vs WT | 1.287566 | 0.001116 |
| PA14_08220 | hypothetical_unclassified | PGD1652145 | NA | DrhlR vs WT | 1.034659 | 0.029902 |
| PA14_08270 | hypothetical_unclassified | PGD1652155 | NA | DrhlR vs WT | 1.992174 | 5.27E-04 |
| NA | NA | PGD1652210 | NA | DrhlR vs WT | -1.3988 | 0.03999 |
| NA | NA | PGD1652224 | NA | DrhlR vs WT | -1.073 | 0.001517 |
| PA14_09400 | putative_enzymes | PGD1652351 | phzS | DrhlR vs WT | -2.61441 | 8.11E-19 |
| PA14_09410 | secreted_factors | PGD1652353 | phzG1 | DrhlR vs WT | -2.98149 | 4.15E-05 |
| PA14_09440 | secreted_factors | PGD1652357 | phzE1 | DrhlR vs WT | -5.13055 | 9.49E-29 |
| PA14_09450 | secreted_factors | PGD1652359 | phzD1 | DrhlR vs WT | -5.53163 | 4.21E-06 |
| PA14_09460 | secreted_factors | PGD1652361 | phzC1 | DrhlR vs WT | -2.61788 | 2.55E-06 |
| PA14_09470 | secreted_factors | PGD1652363 | phzB1 | DrhlR vs WT | -1.8119 | 3.92E-05 |
| PA14_09480 | secreted_factors | PGD1652365 | phzA1 | DrhlR vs WT | -4.38846 | 1.50E-09 |
| PA14_09490 | putative_enzymes | PGD1652367 | phzM | DrhlR vs WT | -1.91041 | 6.26E-19 |
| PA14_09500 | membrane_proteins | PGD1652369 | opmD | DrhlR vs WT | -4.20801 | 4.96E-22 |
| PA14_09500 | transport_of_small_molecule | PGD1652369 | opmD | DrhlR vs WT | -4.20801 | 4.96E-22 |
| PA14_09520 | transport_of_small_molecule | PGD1652371 | mexI | DrhlR vs WT | -3.48753 | 1.45E-52 |
| PA14_09530 | transport_of_small_molecule | PGD1652373 | mexH | DrhlR vs WT | -4.79967 | 9.72E-23 |
| PA14_09540 | membrane_proteins | PGD1652375 | mexG | DrhlR vs WT | -4.56818 | 5.37E-22 |
| PA14_09870 | hypothetical_unclassified | PGD1652419 | eftM | DrhlR vs WT | 1.298279 | 3.12E-05 |
| PA14_09880 | hypothetical_unclassified | PGD1652421 | NA | DrhlR vs WT | 1.584475 | 4.98E-04 |
| PA14_09900 | putative_enzymes | PGD1652425 | prpL | DrhlR vs WT | 2.130775 | 7.32E-15 |
| PA14_09900 | secreted_factors | PGD1652425 | prpL | DrhlR vs WT | 2.130775 | 7.32E-15 |
| PA14_09930 | DNA_replication_recombinat | PGD1652431 | NA | DrhlR vs WT | 1.139667 | 6.58E-05 |
| PA14_09970 | transport_of_small_molecule | PGD1652439 | fpvB | DrhlR vs WT | 1.163026 | 1.50E-04 |
| PA14_10140 | transport_of_small_molecule | PGD1652465 | fepG | DrhlR vs WT | 1.184852 | 0.035775 |
| PA14_10170 | transport_of_small_molecule | PGD1652469 | fepB | DrhlR vs WT | 1.259107 | 0.001257 |
| PA14_10200 | membrane_proteins | PGD1652475 | NA | DrhlR vs WT | 1.2589 | 5.00E-04 |
| PA14_10340 | protein_secretion_export | PGD1652501 | NA | DrhlR vs WT | 1.061008 | 3.14E-05 |
| PA14_10360 | hypothetical_unclassified | PGD1652505 | NA | DrhlR vs WT | -4.13398 | 8.27E-86 |
| PA14_10370 | fatty_acid_and_phospholipid | PGD1652507 | NA | DrhlR vs WT | 1.39664 | 3.57E-07 |
| PA14_10380 | hypothetical_unclassified | PGD1652509 | NA | DrhlR vs WT | 1.082832 | 2.16E-12 |
| PA14_10490 | hypothetical_unclassified | PGD1652523 | NA | DrhlR vs WT | -1.46202 | 1.16E-35 |
| PA14_10910 | transport_of_small_molecule | PGD1652589 | NA | DrhlR vs WT | -1.24956 | 0.008563 |
| PA14_10990 | carbon_compound_catabolis | PGD1652603 | hpaC | DrhlR vs WT | -1.35914 | 1.82E-04 |
| PA14_11010 | hypothetical_unclassified | PGD1652607 | NA | DrhlR vs WT | 1.598788 | 1.22E-05 |

| | | | | | | |
|---|---|---|---|---|---|---|
| PA14_11050 | hypothetical_unclassified | PGD1652613 | NA | DrhlR vs WT | -1.33927 | 0.024043 |
| PA14_11130 | putative_enzymes | PGD1652629 | NA | DrhlR vs WT | -1.74446 | 1.68E-28 |
| PA14_11140 | biosynthesis_of_cofactors | PGD1652631 | NA | DrhlR vs WT | -4.41119 | 3.17E-84 |
| PA14_11170 | hypothetical_unclassified | PGD1652637 | NA | DrhlR vs WT | -1.01861 | 1.46E-09 |
| PA14_11240 | transcriptional_regulators | PGD1652647 | NA | DrhlR vs WT | -1.42296 | 2.47E-20 |
| PA14_11620 | transport_of_small_molecule | PGD1652711 | NA | DrhlR vs WT | -1.10752 | 0.002058 |
| PA14_12440 | transcriptional_regulators | PGD1652837 | NA | DrhlR vs WT | -1.42295 | 1.28E-07 |
| PA14_12620 | hypothetical_unclassified | PGD1652859 | NA | DrhlR vs WT | 1.153558 | 8.35E-17 |
| PA14_12750 | hypothetical_unclassified | PGD1652881 | NA | DrhlR vs WT | 1.001081 | 2.10E-07 |
| PA14_12960 | transport_of_small_molecule | PGD1652911 | NA | DrhlR vs WT | 1.577681 | 0.003174 |
| PA14_13050 | hypothetical_unclassified | PGD1652927 | NA | DrhlR vs WT | -2.02676 | 1.71E-11 |
| PA14_13170 | transport_of_small_molecule | PGD1652943 | NA | DrhlR vs WT | -4.12025 | 3.82E-63 |
| PA14_13210 | hypothetical_unclassified | PGD1652949 | NA | DrhlR vs WT | -3.126 | 5.76E-45 |
| PA14_13230 | biosynthesis_of_cofactors | PGD1652953 | moaC | DrhlR vs WT | -1.52397 | 3.89E-22 |
| PA14_13240 | biosynthesis_of_cofactors | PGD1652955 | moaD | DrhlR vs WT | -1.90213 | 5.74E-11 |
| PA14_13250 | biosynthesis_of_cofactors | PGD1652957 | moaE | DrhlR vs WT | -2.63329 | 1.43E-31 |
| PA14_13260 | biosynthesis_of_cofactors | PGD1652959 | moaB1 | DrhlR vs WT | -2.39955 | 1.26E-19 |
| PA14_13280 | biosynthesis_of_cofactors | PGD1652961 | moeA1 | DrhlR vs WT | -2.82244 | 2.41E-19 |
| PA14_13290 | translation_posttranslational | PGD1652963 | NA | DrhlR vs WT | -1.4034 | 8.23E-08 |
| PA14_13300 | translation_posttranslational | PGD1652965 | NA | DrhlR vs WT | -1.4163 | 6.50E-07 |
| PA14_13320 | fatty_acid_and_phospholipid | PGD1652967 | NA | DrhlR vs WT | -1.16282 | 1.50E-04 |
| PA14_13450 | membrane_proteins | PGD1652989 | NA | DrhlR vs WT | 1.200027 | 0.012895 |
| PA14_13460 | transcriptional_regulators | PGD1652991 | NA | DrhlR vs WT | 1.665206 | 0.0013 |
| PA14_13580 | transport_of_small_molecule | PGD1653007 | NA | DrhlR vs WT | 2.04148 | 3.03E-13 |
| PA14_13590 | membrane_proteins | PGD1653009 | NA | DrhlR vs WT | 2.447011 | 4.16E-19 |
| PA14_13590 | transport_of_small_molecule | PGD1653009 | NA | DrhlR vs WT | 2.447011 | 4.16E-19 |
| PA14_13600 | transport_of_small_molecule | PGD1653011 | NA | DrhlR vs WT | 1.551596 | 1.89E-11 |
| PA14_13610 | membrane_proteins | PGD1653013 | NA | DrhlR vs WT | 1.644964 | 4.55E-16 |
| PA14_13610 | transport_of_small_molecule | PGD1653013 | NA | DrhlR vs WT | 1.644964 | 4.55E-16 |
| PA14_13630 | hypothetical_unclassified | PGD1653017 | NA | DrhlR vs WT | 1.238124 | 4.49E-09 |
| PA14_13750 | membrane_proteins | PGD1653037 | narK1 | DrhlR vs WT | -1.92649 | 3.69E-14 |
| PA14_13750 | transport_of_small_molecule | PGD1653037 | narK1 | DrhlR vs WT | -1.92649 | 3.69E-14 |
| PA14_13770 | transport_of_small_molecule | PGD1653039 | narK2 | DrhlR vs WT | -2.24195 | 5.53E-20 |
| PA14_13780 | energy_metabolism_CAP | PGD1653041 | narG | DrhlR vs WT | -2.01336 | 1.40E-19 |
| PA14_13800 | energy_metabolism_CAP | PGD1653043 | narH | DrhlR vs WT | -1.43133 | 2.21E-09 |
| PA14_13810 | energy_metabolism_CAP | PGD1653045 | narJ | DrhlR vs WT | -1.7431 | 4.04E-07 |
| PA14_13830 | energy_metabolism_CAP | PGD1653047 | narI | DrhlR vs WT | -1.20287 | 1.82E-05 |
| PA14_13840 | chaperones_heat_shock | PGD1653049 | NA | DrhlR vs WT | -1.93667 | 8.60E-09 |
| PA14_13840 | translation_posttranslational | PGD1653049 | NA | DrhlR vs WT | -1.93667 | 8.60E-09 |
| PA14_13920 | hypothetical_unclassified | PGD1653063 | NA | DrhlR vs WT | 1.278527 | 0.002133 |
| PA14_13950 | hypothetical_unclassified | PGD1653067 | NA | DrhlR vs WT | -1.18834 | 0.021004 |
| PA14_13990 | transport_of_small_molecule | PGD1653073 | NA | DrhlR vs WT | -1.07405 | 2.52E-13 |
| PA14_14270 | putative_enzymes | PGD1653111 | NA | DrhlR vs WT | -1.18242 | 4.16E-10 |
| PA14_14320 | hypothetical_unclassified | PGD1653121 | NA | DrhlR vs WT | -1.07183 | 0.023121 |
| PA14_14330 | chaperones_heat_shock | PGD1653123 | NA | DrhlR vs WT | 1.693734 | 1.64E-04 |
| PA14_14330 | protein_secretion_export | PGD1653123 | NA | DrhlR vs WT | 1.693734 | 1.64E-04 |
| PA14_14330 | secreted_factors | PGD1653123 | NA | DrhlR vs WT | 1.693734 | 1.64E-04 |
| PA14_14975 | hypothetical_unclassified | PGD1653225 | NA | DrhlR vs WT | -1.21972 | 1.97E-11 |
| PA14_15710 | hypothetical_unclassified | PGD1653349 | NA | DrhlR vs WT | -1.48579 | 2.72E-04 |
| PA14_15770 | hypothetical_unclassified | PGD1653357 | NA | DrhlR vs WT | 1.135341 | 2.63E-10 |

| | | | | | | |
|---|---|---|---|---|---|---|
| PA14_16010 | fatty_acid_and_phospholipid | PGD1653399 | NA | DrhlR vs WT | 1.349643 | 0.009161 |
| PA14_16100 | hypothetical_unclassified | PGD1653413 | NA | DrhlR vs WT | -3.16725 | 4.09E-49 |
| PA14_16250 | amino_acid_biosynthesis_me | PGD1653435 | lasB | DrhlR vs WT | -2.44443 | 1.70E-15 |
| PA14_16250 | secreted_factors | PGD1653435 | lasB | DrhlR vs WT | -2.44443 | 1.70E-15 |
| PA14_16250 | translation_posttranslational | PGD1653435 | lasB | DrhlR vs WT | -2.44443 | 1.70E-15 |
| PA14_16310 | membrane_proteins | PGD1653447 | NA | DrhlR vs WT | -2.45554 | 7.32E-15 |
| PA14_16310 | transport_of_small_molecule | PGD1653447 | NA | DrhlR vs WT | -2.45554 | 7.32E-15 |
| PA14_16630 | membrane_proteins | PGD1653499 | NA | DrhlR vs WT | 1.732071 | 5.71E-11 |
| PA14_16630 | transport_of_small_molecule | PGD1653499 | NA | DrhlR vs WT | 1.732071 | 5.71E-11 |
| PA14_16680 | hypothetical_unclassified | PGD1653507 | NA | DrhlR vs WT | -1.29653 | 2.12E-14 |
| PA14_16800 | transport_of_small_molecule | PGD1653529 | NA | DrhlR vs WT | 1.271865 | 2.32E-13 |
| PA14_16820 | antibiotic_resistance_and_su | PGD1653531 | NA | DrhlR vs WT | 1.26712 | 6.52E-25 |
| PA14_16990 | hypothetical_unclassified | PGD1653559 | NA | DrhlR vs WT | 1.73619 | 1.51E-05 |
| PA14_17410 | putative_enzymes | PGD1653627 | NA | DrhlR vs WT | -1.19245 | 2.73E-04 |
| PA14_17910 | putative_enzymes | PGD1653711 | NA | DrhlR vs WT | 1.759699 | 9.86E-11 |
| PA14_18020 | secreted_factors | PGD1653727 | NA | DrhlR vs WT | 1.697624 | 1.24E-24 |
| PA14_18090 | adaptation_and_protection | PGD1653739 | NA | DrhlR vs WT | 1.059863 | 5.13E-06 |
| PA14_18090 | transport_of_small_molecule | PGD1653739 | NA | DrhlR vs WT | 1.059863 | 5.13E-06 |
| PA14_18100 | hypothetical_unclassified | PGD1653741 | NA | DrhlR vs WT | 1.08328 | 0.002522 |
| PA14_18120 | amino_acid_biosynthesis_me | PGD1653745 | mmsA | DrhlR vs WT | 1.255792 | 8.68E-24 |
| PA14_18800 | transport_of_small_molecule | PGD1653845 | NA | DrhlR vs WT | -4.9356 | 2.01E-50 |
| PA14_18810 | hypothetical_unclassified | PGD1653847 | NA | DrhlR vs WT | -2.02493 | 1.79E-11 |
| PA14_19100 | secreted_factors | PGD1653891 | rhlA | DrhlR vs WT | -6.66302 | 9.49E-37 |
| PA14_19120 | transcriptional_regulators | PGD1653895 | rhlR | DrhlR vs WT | -5.05362 | 2.12E-36 |
| PA14_19140 | amino_acid_biosynthesis_me | PGD1653899 | pheC | DrhlR vs WT | -1.07175 | 6.41E-13 |
| PA14_19270 | transport_of_small_molecule | PGD1653915 | NA | DrhlR vs WT | -1.64663 | 1.84E-05 |
| PA14_19350 | carbon_compound_catabolis | PGD1653927 | NA | DrhlR vs WT | 1.490186 | 2.96E-09 |
| PA14_19360 | putative_enzymes | PGD1653929 | NA | DrhlR vs WT | 1.128888 | 1.08E-05 |
| PA14_19500 | transport_of_small_molecule | PGD1653951 | NA | DrhlR vs WT | 1.132595 | 0.016706 |
| PA14_19510 | transport_of_small_molecule | PGD1653953 | NA | DrhlR vs WT | 1.025298 | 0.044136 |
| PA14_19520 | transport_of_small_molecule | PGD1653955 | NA | DrhlR vs WT | 1.841472 | 0.001061 |
| PA14_19540 | transport_of_small_molecule | PGD1653959 | NA | DrhlR vs WT | 1.241882 | 0.002675 |
| PA14_19560 | central_intermediary_metab | PGD1653961 | ssuD | DrhlR vs WT | 1.365051 | 0.014632 |
| PA14_19570 | transport_of_small_molecule | PGD1653963 | NA | DrhlR vs WT | 1.323364 | 0.030562 |
| PA14_19580 | transport_of_small_molecule | PGD1653965 | ssuB | DrhlR vs WT | 1.514835 | 0.04388 |
| PA14_19590 | central_intermediary_metab | PGD1653967 | NA | DrhlR vs WT | 1.989776 | 0.001656 |
| PA14_19850 | transcriptional_regulators | PGD1654009 | NA | DrhlR vs WT | -1.21127 | 5.59E-04 |
| PA14_19900 | energy_metabolism_CAP | PGD1654015 | NA | DrhlR vs WT | -1.25579 | 6.41E-07 |
| PA14_19910 | energy_metabolism_CAP | PGD1654017 | NA | DrhlR vs WT | -1.26741 | 4.59E-08 |
| PA14_19920 | energy_metabolism_CAP | PGD1654019 | NA | DrhlR vs WT | -1.51964 | 3.89E-14 |
| PA14_19970 | hypothetical_unclassified | PGD1654029 | NA | DrhlR vs WT | 1.165049 | 0.013218 |
| PA14_19990 | transcriptional_regulators | PGD1654031 | NA | DrhlR vs WT | 2.387312 | 1.08E-05 |
| PA14_20000 | membrane_proteins | PGD1654033 | NA | DrhlR vs WT | 2.015287 | 2.70E-04 |
| PA14_20010 | transport_of_small_molecule | PGD1654035 | hasR | DrhlR vs WT | 3.688214 | 7.84E-11 |
| PA14_20020 | transport_of_small_molecule | PGD1654037 | hasAp | DrhlR vs WT | 4.957392 | 6.10E-14 |
| PA14_20030 | protein_secretion_export | PGD1654039 | hasD | DrhlR vs WT | 3.814278 | 1.20E-08 |
| PA14_20040 | protein_secretion_export | PGD1654041 | hasE | DrhlR vs WT | 3.747566 | 5.29E-08 |
| PA14_20050 | protein_secretion_export | PGD1654043 | NA | DrhlR vs WT | 2.979905 | 3.38E-09 |
| PA14_20300 | transport_of_small_molecule | PGD1654085 | phnC | DrhlR vs WT | 1.660332 | 1.25E-08 |
| PA14_20320 | transport_of_small_molecule | PGD1654087 | phnD | DrhlR vs WT | 3.091122 | 1.75E-08 |

| | | | | | | |
|---|---|---|---|---|---|---|
| PA14_20330 | transport_of_small_molecule | PGD1654089 | phnE | DrhlR vs WT | 2.380909 | 8.16E-05 |
| PA14_20380 | transport_of_small_molecule | PGD1654097 | phnI | DrhlR vs WT | 1.599935 | 0.014121 |
| PA14_20390 | transport_of_small_molecule | PGD1654099 | phnJ | DrhlR vs WT | 1.907979 | 6.09E-04 |
| PA14_20460 | hypothetical_unclassified | PGD1654111 | NA | DrhlR vs WT | 1.090387 | 1.28E-07 |
| PA14_20610 | motility_and_attachment | PGD1654139 | lecB | DrhlR vs WT | -6.61888 | 1.00E-26 |
| PA14_20900 | membrane_proteins | PGD1654189 | NA | DrhlR vs WT | -2.36035 | 6.37E-18 |
| PA14_20920 | hypothetical_unclassified | PGD1654191 | NA | DrhlR vs WT | -1.73696 | 1.38E-22 |
| PA14_20940 | fatty_acid_and_phospholipid | PGD1654193 | NA | DrhlR vs WT | -3.29788 | 7.28E-23 |
| PA14_20950 | fatty_acid_and_phospholipid | PGD1654195 | fabH2 | DrhlR vs WT | -3.25146 | 6.91E-26 |
| PA14_20960 | putative_enzymes | PGD1654197 | NA | DrhlR vs WT | -2.92364 | 6.09E-15 |
| PA14_20970 | adaptation_and_protection | PGD1654199 | cyp23 | DrhlR vs WT | -3.05997 | 5.08E-23 |
| PA14_20980 | putative_enzymes | PGD1654201 | NA | DrhlR vs WT | -3.24626 | 9.22E-31 |
| PA14_21000 | hypothetical_unclassified | PGD1654203 | NA | DrhlR vs WT | -2.9615 | 4.81E-26 |
| PA14_21010 | energy_metabolism_CAP | PGD1654205 | NA | DrhlR vs WT | -3.16576 | 3.73E-22 |
| PA14_21020 | biosynthesis_of_cofactors | PGD1654207 | NA | DrhlR vs WT | -2.93376 | 3.57E-42 |
| PA14_21030 | cell_wall_LPS | PGD1654209 | NA | DrhlR vs WT | -1.73271 | 3.35E-24 |
| PA14_21030 | translation_posttranslational | PGD1654209 | NA | DrhlR vs WT | -1.73271 | 3.35E-24 |
| PA14_21090 | hypothetical_unclassified | PGD1654221 | NA | DrhlR vs WT | -1.18976 | 0.021197 |
| PA14_21410 | central_intermediary_metab | PGD1654271 | phoA | DrhlR vs WT | 2.067934 | 7.93E-04 |
| PA14_21470 | hypothetical_unclassified | PGD1654279 | NA | DrhlR vs WT | -1.05851 | 1.57E-05 |
| PA14_21570 | hypothetical_unclassified | PGD1654297 | NA | DrhlR vs WT | 2.640349 | 1.90E-05 |
| PA14_21580 | hypothetical_unclassified | PGD1654299 | NA | DrhlR vs WT | 2.976014 | 9.72E-06 |
| PA14_21590 | hypothetical_unclassified | PGD1654301 | NA | DrhlR vs WT | 2.530138 | 4.91E-04 |
| PA14_21600 | membrane_proteins | PGD1654303 | NA | DrhlR vs WT | 2.910923 | 3.78E-05 |
| PA14_21630 | membrane_proteins | PGD1654309 | NA | DrhlR vs WT | 1.932661 | 2.92E-14 |
| PA14_21640 | biosynthesis_of_cofactors | PGD1654311 | NA | DrhlR vs WT | 1.022645 | 1.26E-05 |
| PA14_21640 | putative_enzymes | PGD1654311 | NA | DrhlR vs WT | 1.022645 | 1.26E-05 |
| PA14_21670 | hypothetical_unclassified | PGD1654317 | NA | DrhlR vs WT | 1.388881 | 1.14E-11 |
| PA14_21680 | hypothetical_unclassified | PGD1654319 | NA | DrhlR vs WT | 1.089917 | 1.44E-05 |
| PA14_21830 | hypothetical_unclassified | PGD1654347 | NA | DrhlR vs WT | 1.278607 | 6.81E-21 |
| PA14_22010 | cell_division | PGD1654381 | minE | DrhlR vs WT | -1.09858 | 0.00114 |
| PA14_22320 | membrane_proteins | PGD1654433 | NA | DrhlR vs WT | -1.46464 | 0.001213 |
| PA14_22330 | transport_of_small_molecule | PGD1654435 | NA | DrhlR vs WT | -1.01913 | 9.32E-06 |
| PA14_22350 | membrane_proteins | PGD1654439 | actP | DrhlR vs WT | 1.01363 | 8.91E-05 |
| NA | NA | PGD1654630 | NA | DrhlR vs WT | -1.8689 | 0.018309 |
| PA14_23680 | chaperones_heat_shock | PGD1654661 | ibpA | DrhlR vs WT | 2.496085 | 3.54E-10 |
| PA14_23690 | protein_secretion_export | PGD1654663 | NA | DrhlR vs WT | -1.00028 | 2.62E-05 |
| PA14_23720 | translation_posttranslational | PGD1654667 | NA | DrhlR vs WT | -1.04024 | 5.36E-14 |
| PA14_23980 | protein_secretion_export | PGD1654705 | xcpP | DrhlR vs WT | -1.69056 | 8.34E-11 |
| PA14_24180 | hypothetical_unclassified | PGD1654737 | NA | DrhlR vs WT | 1.203215 | 5.02E-18 |
| PA14_24210 | hypothetical_unclassified | PGD1654741 | NA | DrhlR vs WT | -1.29802 | 6.07E-12 |
| PA14_24570 | hypothetical_unclassified | PGD1654801 | NA | DrhlR vs WT | -1.70836 | 0.018817 |
| PA14_24630 | hypothetical_unclassified | PGD1654813 | NA | DrhlR vs WT | -1.12463 | 2.23E-07 |
| PA14_24760 | membrane_proteins | PGD1654835 | NA | DrhlR vs WT | 1.025949 | 4.64E-06 |
| PA14_24860 | energy_metabolism_CAP | PGD1654853 | snr1 | DrhlR vs WT | -1.74115 | 8.03E-19 |
| NA | NA | PGD1654856 | NA | DrhlR vs WT | 1.6231 | 1.04E-08 |
| PA14_25080 | amino_acid_biosynthesis_me | PGD1654891 | fadB | DrhlR vs WT | 1.003171 | 1.31E-11 |
| PA14_25080 | fatty_acid_and_phospholipid | PGD1654891 | fadB | DrhlR vs WT | 1.003171 | 1.31E-11 |
| PA14_25670 | fatty_acid_and_phospholipid | PGD1654989 | acpP | DrhlR vs WT | -1.056 | 2.21E-04 |
| PA14_26000 | biosynthesis_of_cofactors | PGD1655039 | NA | DrhlR vs WT | -1.19462 | 8.43E-04 |

| | | | | | | |
|---|---|---|---|---|---|---|
| PA14_26190 | hypothetical_unclassified | PGD1655067 | NA | DrhlR vs WT | -1.66567 | 8.61E-13 |
| PA14_26200 | hypothetical_unclassified | PGD1655069 | NA | DrhlR vs WT | -1.34221 | 0.003768 |
| PA14_26350 | putative_enzymes | PGD1655093 | NA | DrhlR vs WT | -1.161 | 1.19E-08 |
| PA14_26650 | putative_enzymes | PGD1655139 | NA | DrhlR vs WT | -1.55446 | 1.26E-04 |
| PA14_26670 | fatty_acid_and_phospholipid | PGD1655141 | NA | DrhlR vs WT | -1.61821 | 4.29E-12 |
| PA14_26700 | fatty_acid_and_phospholipid | PGD1655145 | NA | DrhlR vs WT | -1.95522 | 3.21E-13 |
| PA14_26700 | putative_enzymes | PGD1655145 | NA | DrhlR vs WT | -1.95522 | 3.21E-13 |
| PA14_26720 | fatty_acid_and_phospholipid | PGD1655147 | NA | DrhlR vs WT | -2.1719 | 3.09E-18 |
| PA14_26720 | putative_enzymes | PGD1655147 | NA | DrhlR vs WT | -2.1719 | 3.09E-18 |
| PA14_26730 | putative_enzymes | PGD1655149 | NA | DrhlR vs WT | -2.00345 | 7.51E-08 |
| PA14_26750 | hypothetical_unclassified | PGD1655151 | NA | DrhlR vs WT | -1.65344 | 6.10E-14 |
| PA14_26770 | membrane_proteins | PGD1655155 | NA | DrhlR vs WT | 1.248351 | 1.23E-19 |
| PA14_26770 | putative_enzymes | PGD1655155 | NA | DrhlR vs WT | 1.248351 | 1.23E-19 |
| PA14_26810 | two_component_regulators | PGD1655159 | NA | DrhlR vs WT | 1.773373 | 9.73E-04 |
| PA14_26830 | two_component_regulators | PGD1655161 | NA | DrhlR vs WT | 1.145722 | 0.030141 |
| PA14_27100 | secreted_factors | PGD1655199 | lipA | DrhlR vs WT | 1.231943 | 2.42E-04 |
| PA14_27230 | transcriptional_regulators | PGD1655225 | NA | DrhlR vs WT | -1.0344 | 9.52E-06 |
| PA14_27370 | transcription_RNA_processin | PGD1655243 | NA | DrhlR vs WT | 1.175782 | 1.28E-05 |
| PA14_27400 | transcriptional_regulators | PGD1655247 | NA | DrhlR vs WT | -1.62358 | 2.87E-04 |
| NA | NA | PGD1655290 | NA | DrhlR vs WT | -1.08633 | 0.001129 |
| PA14_27890 | putative_enzymes | PGD1655335 | NA | DrhlR vs WT | 2.289623 | 1.64E-04 |
| PA14_27980 | translation_posttranslational | PGD1655351 | NA | DrhlR vs WT | 1.082816 | 1.04E-08 |
| PA14_28050 | adaptation_and_protection | PGD1655365 | NA | DrhlR vs WT | -1.29001 | 5.83E-10 |
| PA14_28050 | chemotaxis | PGD1655365 | NA | DrhlR vs WT | -1.29001 | 5.83E-10 |
| PA14_28100 | hypothetical_unclassified | PGD1655375 | NA | DrhlR vs WT | 1.296452 | 0.001656 |
| PA14_28110 | hypothetical_unclassified | PGD1655377 | NA | DrhlR vs WT | 1.479254 | 0.004733 |
| PA14_28250 | secreted_factors | PGD1655403 | NA | DrhlR vs WT | -4.46803 | 2.48E-51 |
| PA14_28260 | hypothetical_unclassified | PGD1655405 | NA | DrhlR vs WT | -2.30919 | 1.99E-20 |
| PA14_28280 | central_intermediary_metab | PGD1655407 | NA | DrhlR vs WT | -1.22171 | 5.12E-11 |
| PA14_28290 | DNA_replication_recombinat | PGD1655409 | NA | DrhlR vs WT | -1.13481 | 0.001094 |
| PA14_28330 | hypothetical_unclassified | PGD1655417 | NA | DrhlR vs WT | 2.591449 | 0.006298 |
| PA14_28360 | hypothetical_unclassified | PGD1655423 | NA | DrhlR vs WT | -4.33739 | 6.63E-29 |
| PA14_28370 | membrane_proteins | PGD1655425 | NA | DrhlR vs WT | -2.31909 | 4.96E-11 |
| PA14_28390 | membrane_proteins | PGD1655429 | NA | DrhlR vs WT | 2.674714 | 1.87E-12 |
| PA14_28410 | hypothetical_unclassified | PGD1655433 | NA | DrhlR vs WT | -3.33681 | 3.27E-10 |
| PA14_28450 | translation_posttranslational | PGD1655441 | eco | DrhlR vs WT | 1.137599 | 1.36E-11 |
| PA14_28610 | hypothetical_unclassified | PGD1655469 | NA | DrhlR vs WT | -1.71768 | 1.13E-08 |
| PA14_28670 | translation_posttranslational | PGD1655479 | rpmI | DrhlR vs WT | 1.25889 | 1.07E-08 |
| PA14_28850 | relative_phage_transposon | PGD1655513 | NA | DrhlR vs WT | -1.28179 | 7.73E-11 |
| PA14_29230 | putative_enzymes | PGD1655579 | NA | DrhlR vs WT | -1.4181 | 1.67E-08 |
| PA14_29320 | putative_enzymes | PGD1655595 | NA | DrhlR vs WT | -1.25386 | 0.00108 |
| PA14_29420 | putative_enzymes | PGD1655612 | NA | DrhlR vs WT | -1.14671 | 6.27E-08 |
| PA14_29520 | protein_secretion_export | PGD1655628 | NA | DrhlR vs WT | 3.338073 | 0.039012 |
| PA14_29620 | transcriptional_regulators | PGD1655646 | NA | DrhlR vs WT | 1.125731 | 6.76E-05 |
| PA14_30240 | translation_posttranslational | PGD1655738 | infA | DrhlR vs WT | -1.03921 | 5.55E-04 |
| PA14_30560 | translation_posttranslational | PGD1655792 | NA | DrhlR vs WT | -1.84134 | 3.06E-15 |
| PA14_30570 | transport_of_small_molecule | PGD1655794 | NA | DrhlR vs WT | -1.41772 | 8.14E-15 |
| PA14_30620 | transcriptional_regulators | PGD1655802 | NA | DrhlR vs WT | -2.07371 | 1.44E-39 |
| NA | NA | PGD1655813 | NA | DrhlR vs WT | -1.09992 | 0.010527 |
| PA14_31270 | hypothetical_unclassified | PGD1655918 | NA | DrhlR vs WT | -1.40478 | 1.91E-10 |

| | | | | | | |
|---|---|---|---|---|---|---|
| PA14_31280 | relative_phage_transposon | PGD1655920 | NA | DrhlR vs WT | -1.63442 | 2.17E-13 |
| PA14_31290 | adaptation_and_protection | PGD1655922 | pa1L | DrhlR vs WT | -6.24119 | 3.19E-36 |
| PA14_31290 | cell_wall_LPS | PGD1655922 | pa1L | DrhlR vs WT | -6.24119 | 3.19E-36 |
| PA14_31290 | motility_and_attachment | PGD1655922 | pa1L | DrhlR vs WT | -6.24119 | 3.19E-36 |
| PA14_31300 | hypothetical_unclassified | PGD1655924 | NA | DrhlR vs WT | -1.01968 | 7.29E-05 |
| PA14_31350 | energy_metabolism_CAP | PGD1655932 | NA | DrhlR vs WT | -2.62141 | 2.02E-50 |
| PA14_31360 | hypothetical_unclassified | PGD1655934 | NA | DrhlR vs WT | -2.83347 | 1.19E-49 |
| PA14_31370 | putative_enzymes | PGD1655936 | NA | DrhlR vs WT | -2.53191 | 4.15E-55 |
| PA14_31430 | hypothetical_unclassified | PGD1655946 | NA | DrhlR vs WT | 1.119195 | 4.50E-08 |
| PA14_31450 | hypothetical_unclassified | PGD1655950 | NA | DrhlR vs WT | -1.22306 | 5.20E-07 |
| PA14_31780 | transcriptional_regulators | PGD1656000 | NA | DrhlR vs WT | -1.23155 | 1.42E-05 |
| PA14_31820 | amino_acid_biosynthesis_me | PGD1656006 | NA | DrhlR vs WT | 1.963149 | 2.22E-07 |
| PA14_32060 | transcriptional_regulators | PGD1656032 | xylS | DrhlR vs WT | -1.93519 | 1.12E-09 |
| PA14_32140 | energy_metabolism_CAP | PGD1656042 | antC | DrhlR vs WT | -6.83253 | 4.80E-14 |
| PA14_32150 | biosynthesis_of_cofactors | PGD1656044 | antB | DrhlR vs WT | -3.58905 | 1.58E-06 |
| PA14_32160 | carbon_compound_catabolis | PGD1656046 | antA | DrhlR vs WT | -3.08927 | 6.13E-19 |
| PA14_32190 | transcriptional_regulators | PGD1656048 | NA | DrhlR vs WT | -2.78782 | 3.01E-28 |
| PA14_32220 | carbon_compound_catabolis | PGD1656052 | catB | DrhlR vs WT | -3.77212 | 1.03E-13 |
| PA14_32230 | carbon_compound_catabolis | PGD1656054 | catC | DrhlR vs WT | -4.57656 | 3.05E-19 |
| PA14_32240 | carbon_compound_catabolis | PGD1656056 | catA | DrhlR vs WT | -3.88198 | 1.84E-24 |
| PA14_32700 | transcriptional_regulators | PGD1656132 | NA | DrhlR vs WT | -1.03413 | 0.007812 |
| PA14_32710 | transcriptional_regulators | PGD1656134 | NA | DrhlR vs WT | 1.122205 | 3.88E-05 |
| PA14_32720 | membrane_proteins | PGD1656136 | NA | DrhlR vs WT | 1.063272 | 0.009532 |
| PA14_32750 | hypothetical_unclassified | PGD1656140 | NA | DrhlR vs WT | -1.08621 | 0.03391 |
| PA14_32850 | hypothetical_unclassified | PGD1656156 | NA | DrhlR vs WT | 1.19866 | 4.50E-07 |
| PA14_32860 | hypothetical_unclassified | PGD1656158 | NA | DrhlR vs WT | 1.096923 | 8.72E-05 |
| PA14_32890 | hypothetical_unclassified | PGD1656162 | NA | DrhlR vs WT | 1.397359 | 3.22E-08 |
| PA14_32905 | hypothetical_unclassified | PGD1656164 | NA | DrhlR vs WT | 1.788075 | 1.31E-11 |
| PA14_32950 | hypothetical_unclassified | PGD1656170 | NA | DrhlR vs WT | -1.01894 | 1.61E-04 |
| PA14_33120 | hypothetical_unclassified | PGD1656194 | NA | DrhlR vs WT | 1.092538 | 5.55E-08 |
| PA14_33160 | hypothetical_unclassified | PGD1656200 | NA | DrhlR vs WT | 1.518052 | 4.29E-12 |
| PA14_33240 | hypothetical_unclassified | PGD1656212 | NA | DrhlR vs WT | 1.536261 | 4.89E-04 |
| PA14_33250 | hypothetical_unclassified | PGD1656214 | NA | DrhlR vs WT | 1.524062 | 1.70E-06 |
| PA14_33260 | transcriptional_regulators | PGD1656216 | pvdS | DrhlR vs WT | 1.793898 | 2.66E-06 |
| PA14_33270 | adaptation_and_protection | PGD1656218 | pvdG | DrhlR vs WT | 1.384213 | 1.74E-07 |
| PA14_33280 | adaptation_and_protection | PGD1656220 | pvdL | DrhlR vs WT | 1.618724 | 2.04E-08 |
| PA14_33460 | membrane_proteins | PGD1656252 | NA | DrhlR vs WT | 1.854239 | 2.04E-14 |
| PA14_33480 | carbon_compound_catabolis | PGD1656254 | sndH | DrhlR vs WT | 1.248821 | 1.12E-08 |
| PA14_33500 | adaptation_and_protection | PGD1656256 | pvdH | DrhlR vs WT | 2.17925 | 2.06E-11 |
| PA14_33500 | transport_of_small_molecule | PGD1656256 | pvdH | DrhlR vs WT | 2.17925 | 2.06E-11 |
| PA14_33610 | putative_enzymes | PGD1656278 | NA | DrhlR vs WT | 1.3853 | 8.65E-08 |
| PA14_33630 | adaptation_and_protection | PGD1656280 | pvdJ | DrhlR vs WT | 1.513 | 3.71E-10 |
| PA14_33650 | adaptation_and_protection | PGD1656282 | pvdD | DrhlR vs WT | 1.444714 | 1.91E-09 |
| PA14_33680 | transport_of_small_molecule | PGD1656284 | fpvA | DrhlR vs WT | 1.781059 | 3.31E-10 |
| PA14_33690 | adaptation_and_protection | PGD1656286 | pvdE | DrhlR vs WT | 1.776332 | 1.04E-08 |
| PA14_33690 | membrane_proteins | PGD1656286 | pvdE | DrhlR vs WT | 1.776332 | 1.04E-08 |
| PA14_33690 | transport_of_small_molecule | PGD1656286 | pvdE | DrhlR vs WT | 1.776332 | 1.04E-08 |
| PA14_33700 | adaptation_and_protection | PGD1656288 | pvdF | DrhlR vs WT | 1.422741 | 2.61E-09 |
| PA14_33700 | secreted_factors | PGD1656288 | pvdF | DrhlR vs WT | 1.422741 | 2.61E-09 |
| PA14_33710 | adaptation_and_protection | PGD1656290 | pvdO | DrhlR vs WT | 1.91848 | 1.61E-08 |

| | | | | | | |
|---|---|---|---|---|---|---|
| PA14_33720 | adaptation_and_protection | PGD1656292 | pvdN | DrhlR vs WT | 1.845534 | 1.61E-08 |
| PA14_33720 | transport_of_small_molecule | PGD1656292 | pvdN | DrhlR vs WT | 1.845534 | 1.61E-08 |
| PA14_33730 | central_intermediary_metab | PGD1656294 | NA | DrhlR vs WT | 1.883107 | 1.27E-08 |
| PA14_33740 | adaptation_and_protection | PGD1656296 | pvdP | DrhlR vs WT | 1.791309 | 1.35E-08 |
| PA14_33750 | transport_of_small_molecule | PGD1656298 | NA | DrhlR vs WT | 1.646941 | 2.57E-09 |
| PA14_33760 | membrane_proteins | PGD1656300 | NA | DrhlR vs WT | 1.480152 | 1.04E-08 |
| PA14_33760 | transport_of_small_molecule | PGD1656300 | NA | DrhlR vs WT | 1.480152 | 1.04E-08 |
| PA14_33770 | protein_secretion_export | PGD1656302 | NA | DrhlR vs WT | 1.542726 | 9.61E-09 |
| PA14_33810 | biosynthesis_of_cofactors | PGD1656308 | pvdA | DrhlR vs WT | 1.880422 | 1.05E-09 |
| PA14_33820 | adaptation_and_protection | PGD1656310 | pvdQ | DrhlR vs WT | 1.932419 | 2.81E-10 |
| PA14_33820 | antibiotic_resistance_and_su | PGD1656310 | pvdQ | DrhlR vs WT | 1.932419 | 2.81E-10 |
| PA14_33830 | hypothetical_unclassified | PGD1656312 | NA | DrhlR vs WT | 1.094957 | 0.001094 |
| PA14_33910 | transport_of_small_molecule | PGD1656326 | NA | DrhlR vs WT | 4.471422 | 1.46E-40 |
| PA14_33990 | translation_posttranslational | PGD1656340 | clpV3 | DrhlR vs WT | -1.38243 | 7.44E-12 |
| PA14_34010 | membrane_proteins | PGD1656344 | hsiG3 | DrhlR vs WT | -1.00061 | 1.71E-04 |
| PA14_34020 | hypothetical_unclassified | PGD1656346 | hsiF3 | DrhlR vs WT | -1.11344 | 5.72E-04 |
| PA14_34030 | hypothetical_unclassified | PGD1656348 | hcp3 | DrhlR vs WT | -1.38798 | 1.91E-07 |
| PA14_34070 | hypothetical_unclassified | PGD1656352 | hsiB3 | DrhlR vs WT | -1.07754 | 1.70E-04 |
| PA14_34130 | hypothetical_unclassified | PGD1656360 | icmF3 | DrhlR vs WT | -1.21525 | 8.93E-14 |
| PA14_34490 | fatty_acid_and_phospholipid | PGD1656414 | NA | DrhlR vs WT | -1.20966 | 5.12E-05 |
| PA14_34510 | transport_of_small_molecule | PGD1656418 | NA | DrhlR vs WT | -1.28815 | 8.46E-11 |
| PA14_34540 | putative_enzymes | PGD1656422 | NA | DrhlR vs WT | -1.05023 | 2.67E-06 |
| PA14_34730 | transcriptional_regulators | PGD1656450 | NA | DrhlR vs WT | 1.156143 | 0.016725 |
| PA14_34770 | transport_of_small_molecule | PGD1656456 | NA | DrhlR vs WT | 1.362973 | 0.016922 |
| PA14_34810 | fatty_acid_and_phospholipid | PGD1656464 | NA | DrhlR vs WT | -1.38279 | 5.63E-13 |
| PA14_34810 | putative_enzymes | PGD1656464 | NA | DrhlR vs WT | -1.38279 | 5.63E-13 |
| PA14_34810 | secreted_factors | PGD1656464 | NA | DrhlR vs WT | -1.38279 | 5.63E-13 |
| PA14_34820 | transcriptional_regulators | PGD1656466 | NA | DrhlR vs WT | -1.07401 | 1.51E-08 |
| PA14_34870 | carbon_compound_catabolis | PGD1656474 | chiC | DrhlR vs WT | -7.03132 | 4.57E-50 |
| PA14_34880 | transcriptional_regulators | PGD1656476 | NA | DrhlR vs WT | -1.23431 | 6.58E-04 |
| PA14_35160 | hypothetical_unclassified | PGD1656520 | NA | DrhlR vs WT | -2.34385 | 9.03E-16 |
| PA14_35170 | transcriptional_regulators | PGD1656522 | NA | DrhlR vs WT | -1.2675 | 3.29E-05 |
| PA14_35240 | hypothetical_unclassified | PGD1656532 | NA | DrhlR vs WT | -1.50696 | 0.001777 |
| PA14_35380 | transcriptional_regulators | PGD1656552 | ptxR | DrhlR vs WT | 1.182206 | 8.31E-07 |
| PA14_35420 | amino_acid_biosynthesis_me | PGD1656558 | pvcB | DrhlR vs WT | 1.115686 | 0.03884 |
| PA14_35420 | secreted_factors | PGD1656558 | pvcB | DrhlR vs WT | 1.115686 | 0.03884 |
| PA14_35430 | amino_acid_biosynthesis_me | PGD1656560 | pvcA | DrhlR vs WT | 1.479373 | 3.78E-09 |
| PA14_35430 | secreted_factors | PGD1656560 | pvcA | DrhlR vs WT | 1.479373 | 3.78E-09 |
| PA14_35550 | hypothetical_unclassified | PGD1656578 | pslO | DrhlR vs WT | 1.172115 | 2.80E-05 |
| PA14_35680 | hypothetical_unclassified | PGD1656596 | pslF | DrhlR vs WT | -1.63537 | 4.18E-04 |
| PA14_36090 | membrane_proteins | PGD1656670 | NA | DrhlR vs WT | -1.13983 | 0.005848 |
| PA14_36310 | central_intermediary_metab | PGD1656706 | hcnC | DrhlR vs WT | -2.51135 | 3.19E-36 |
| PA14_36320 | central_intermediary_metab | PGD1656708 | hcnB | DrhlR vs WT | -2.77758 | 2.99E-29 |
| PA14_36330 | central_intermediary_metab | PGD1656710 | hcnA | DrhlR vs WT | -3.68522 | 2.75E-50 |
| PA14_36375 | hypothetical_unclassified | PGD1656720 | NA | DrhlR vs WT | 1.315223 | 8.73E-05 |
| PA14_36390 | translation_posttranslational | PGD1656722 | NA | DrhlR vs WT | 1.078349 | 5.78E-04 |
| PA14_36480 | hypothetical_unclassified | PGD1656736 | NA | DrhlR vs WT | 1.689801 | 1.99E-06 |
| PA14_36500 | carbon_compound_catabolis | PGD1656740 | NA | DrhlR vs WT | 1.380208 | 5.13E-08 |
| PA14_36520 | hypothetical_unclassified | PGD1656742 | NA | DrhlR vs WT | 1.445692 | 1.26E-13 |
| PA14_36530 | hypothetical_unclassified | PGD1656744 | NA | DrhlR vs WT | 1.155107 | 1.55E-07 |

| | | | | | | |
|---|---|---|---|---|---|---|
| PA14_36550 | hypothetical_unclassified | PGD1656748 | NA | DrhlR vs WT | 1.084487 | 3.25E-08 |
| PA14_36570 | carbon_compound_catabolis | PGD1656752 | glgA | DrhlR vs WT | 1.128045 | 6.93E-09 |
| PA14_36570 | energy_metabolism_CAP | PGD1656752 | glgA | DrhlR vs WT | 1.128045 | 6.93E-09 |
| PA14_36580 | putative_enzymes | PGD1656754 | NA | DrhlR vs WT | 1.489672 | 1.61E-08 |
| PA14_36590 | carbon_compound_catabolis | PGD1656756 | NA | DrhlR vs WT | 2.050622 | 2.32E-08 |
| PA14_36605 | putative_enzymes | PGD1656758 | NA | DrhlR vs WT | 2.790329 | 5.08E-15 |
| PA14_36620 | hypothetical_unclassified | PGD1656760 | NA | DrhlR vs WT | 4.730649 | 8.25E-04 |
| PA14_36630 | carbon_compound_catabolis | PGD1656762 | NA | DrhlR vs WT | 2.679288 | 5.91E-21 |
| PA14_36630 | putative_enzymes | PGD1656762 | NA | DrhlR vs WT | 2.679288 | 5.91E-21 |
| PA14_36650 | hypothetical_unclassified | PGD1656764 | NA | DrhlR vs WT | 1.306632 | 2.64E-05 |
| PA14_36660 | amino_acid_biosynthesis_me | PGD1656766 | NA | DrhlR vs WT | 1.004554 | 5.79E-05 |
| PA14_36670 | hypothetical_unclassified | PGD1656768 | NA | DrhlR vs WT | 1.552806 | 1.89E-05 |
| PA14_36680 | putative_enzymes | PGD1656770 | NA | DrhlR vs WT | 1.250626 | 0.00171 |
| PA14_36690 | fatty_acid_and_phospholipid | PGD1656772 | NA | DrhlR vs WT | 1.258493 | 3.17E-04 |
| PA14_36690 | putative_enzymes | PGD1656772 | NA | DrhlR vs WT | 1.258493 | 3.17E-04 |
| PA14_36700 | membrane_proteins | PGD1656774 | NA | DrhlR vs WT | 1.408556 | 1.30E-04 |
| PA14_36710 | energy_metabolism_CAP | PGD1656776 | glgB | DrhlR vs WT | 3.115862 | 3.21E-13 |
| PA14_36730 | carbon_compound_catabolis | PGD1656778 | NA | DrhlR vs WT | 1.823747 | 5.21E-12 |
| PA14_36730 | putative_enzymes | PGD1656778 | NA | DrhlR vs WT | 1.823747 | 5.21E-12 |
| PA14_36740 | putative_enzymes | PGD1656780 | NA | DrhlR vs WT | 1.069452 | 3.82E-08 |
| PA14_36760 | DNA_replication_recombinat | PGD1656782 | NA | DrhlR vs WT | 1.039893 | 1.76E-04 |
| PA14_36770 | hypothetical_unclassified | PGD1656784 | NA | DrhlR vs WT | 1.049491 | 0.008556 |
| PA14_36790 | hypothetical_unclassified | PGD1656788 | NA | DrhlR vs WT | 1.291164 | 6.41E-07 |
| PA14_36810 | adaptation_and_protection | PGD1656790 | katE | DrhlR vs WT | 1.477799 | 2.62E-07 |
| PA14_36880 | transcriptional_regulators | PGD1656804 | NA | DrhlR vs WT | 1.072425 | 0.014107 |
| PA14_36900 | hypothetical_unclassified | PGD1656808 | NA | DrhlR vs WT | 1.070708 | 0.002809 |
| PA14_37040 | chaperones_heat_shock | PGD1656830 | cupA2 | DrhlR vs WT | 2.075282 | 0.038359 |
| PA14_37060 | motility_and_attachment | PGD1656832 | cupA1 | DrhlR vs WT | 2.443184 | 6.78E-07 |
| PA14_37070 | central_intermediary_metab | PGD1656834 | NA | DrhlR vs WT | 1.209694 | 2.08E-06 |
| PA14_37070 | transcriptional_regulators | PGD1656834 | NA | DrhlR vs WT | 1.209694 | 2.08E-06 |
| PA14_37310 | transport_of_small_molecule | PGD1656868 | NA | DrhlR vs WT | 1.102082 | 1.53E-05 |
| PA14_37320 | membrane_proteins | PGD1656870 | NA | DrhlR vs WT | 1.347997 | 1.69E-06 |
| PA14_37360 | central_intermediary_metab | PGD1656876 | NA | DrhlR vs WT | -2.73767 | 4.98E-20 |
| PA14_37370 | fatty_acid_and_phospholipid | PGD1656878 | NA | DrhlR vs WT | -2.76621 | 2.61E-06 |
| PA14_37380 | transport_of_small_molecule | PGD1656880 | NA | DrhlR vs WT | -1.95486 | 1.11E-18 |
| PA14_37420 | transport_of_small_molecule | PGD1656886 | NA | DrhlR vs WT | 1.595875 | 8.33E-04 |
| PA14_37460 | transport_of_small_molecule | PGD1656892 | NA | DrhlR vs WT | 1.523784 | 1.96E-04 |
| PA14_37470 | energy_metabolism_CAP | PGD1656894 | NA | DrhlR vs WT | 1.653097 | 9.68E-04 |
| PA14_37510 | hypothetical_unclassified | PGD1656898 | NA | DrhlR vs WT | 1.29495 | 0.011366 |
| PA14_37520 | hypothetical_unclassified | PGD1656900 | NA | DrhlR vs WT | 2.604404 | 0.001737 |
| PA14_37530 | putative_enzymes | PGD1656902 | NA | DrhlR vs WT | 1.233307 | 0.009602 |
| PA14_37550 | carbon_compound_catabolis | PGD1656904 | NA | DrhlR vs WT | 2.031757 | 0.007596 |
| PA14_37560 | amino_acid_biosynthesis_me | PGD1656906 | NA | DrhlR vs WT | 1.205679 | 0.005439 |
| PA14_37730 | transport_of_small_molecule | PGD1656932 | NA | DrhlR vs WT | -1.66597 | 1.18E-17 |
| PA14_37745 | putative_enzymes | PGD1656934 | NA | DrhlR vs WT | -6.36206 | 2.23E-27 |
| PA14_37760 | membrane_proteins | PGD1656936 | NA | DrhlR vs WT | -3.71079 | 8.80E-53 |
| PA14_37760 | transport_of_small_molecule | PGD1656936 | NA | DrhlR vs WT | -3.71079 | 8.80E-53 |
| PA14_37770 | carbon_compound_catabolis | PGD1656938 | NA | DrhlR vs WT | -3.30265 | 1.96E-28 |
| PA14_37780 | hypothetical_unclassified | PGD1656940 | NA | DrhlR vs WT | -3.51006 | 8.08E-33 |
| PA14_37790 | adaptation_and_protection | PGD1656942 | pcoA | DrhlR vs WT | -1.79854 | 1.34E-20 |

| | | | | | | |
|---|---|---|---|---|---|---|
| PA14_37810 | adaptation_and_protection | PGD1656944 | pcoB | DrhlR vs WT | -1.8433 | 9.93E-06 |
| PA14_37840 | transport_of_small_molecule | PGD1656950 | sppD | DrhlR vs WT | -1.1118 | 4.43E-04 |
| PA14_38130 | transport_of_small_molecule | PGD1656992 | NA | DrhlR vs WT | 1.28307 | 5.51E-09 |
| PA14_38140 | amino_acid_biosynthesis_me | PGD1656994 | NA | DrhlR vs WT | 1.015955 | 1.66E-05 |
| PA14_38210 | central_intermediary_metab | PGD1657006 | NA | DrhlR vs WT | 2.527504 | 3.14E-09 |
| PA14_38220 | transport_of_small_molecule | PGD1657008 | NA | DrhlR vs WT | 2.719511 | 3.89E-15 |
| PA14_38260 | hypothetical_unclassified | PGD1657012 | NA | DrhlR vs WT | -3.0727 | 6.66E-26 |
| PA14_38270 | hypothetical_unclassified | PGD1657014 | NA | DrhlR vs WT | -2.66087 | 6.29E-18 |
| PA14_38310 | hypothetical_unclassified | PGD1657020 | NA | DrhlR vs WT | 1.835816 | 0.007722 |
| PA14_38360 | cell_wall_LPS | PGD1657030 | NA | DrhlR vs WT | 1.658472 | 6.50E-17 |
| PA14_38360 | putative_enzymes | PGD1657030 | NA | DrhlR vs WT | 1.658472 | 6.50E-17 |
| PA14_38440 | carbon_compound_catabolis | PGD1657044 | gnyD | DrhlR vs WT | 1.13949 | 3.43E-23 |
| PA14_38440 | fatty_acid_and_phospholipid | PGD1657044 | gnyD | DrhlR vs WT | 1.13949 | 3.43E-23 |
| PA14_38460 | carbon_compound_catabolis | PGD1657046 | gnyB | DrhlR vs WT | 1.04564 | 4.85E-11 |
| PA14_38480 | carbon_compound_catabolis | PGD1657050 | gnyA | DrhlR vs WT | 1.057244 | 4.62E-10 |
| PA14_38590 | carbon_compound_catabolis | PGD1657068 | bdhA | DrhlR vs WT | -1.08123 | 1.31E-07 |
| PA14_38730 | transport_of_small_molecule | PGD1657088 | NA | DrhlR vs WT | 1.160507 | 1.43E-04 |
| PA14_39210 | biosynthesis_of_cofactors | PGD1657162 | NA | DrhlR vs WT | -1.16022 | 0.001822 |
| PA14_39460 | hypothetical_unclassified | PGD1657196 | NA | DrhlR vs WT | -2.86208 | 2.92E-21 |
| PA14_39470 | hypothetical_unclassified | PGD1657198 | NA | DrhlR vs WT | -2.02293 | 6.22E-10 |
| PA14_39500 | hypothetical_unclassified | PGD1657202 | NA | DrhlR vs WT | 3.754472 | 1.06E-36 |
| PA14_39590 | amino_acid_biosynthesis_me | PGD1657216 | metE | DrhlR vs WT | -2.48646 | 4.67E-09 |
| PA14_39780 | transport_of_small_molecule | PGD1657246 | NA | DrhlR vs WT | -2.89104 | 3.16E-29 |
| PA14_39800 | transcriptional_regulators | PGD1657250 | NA | DrhlR vs WT | 2.074354 | 6.53E-07 |
| PA14_39810 | membrane_proteins | PGD1657252 | NA | DrhlR vs WT | 1.468977 | 0.007934 |
| PA14_39820 | transport_of_small_molecule | PGD1657254 | NA | DrhlR vs WT | 1.447153 | 7.34E-04 |
| PA14_39880 | secreted_factors | PGD1657264 | phzG2 | DrhlR vs WT | -6.45554 | 9.47E-28 |
| PA14_39945 | secreted_factors | PGD1657272 | phzC2 | DrhlR vs WT | -7.04317 | 5.61E-29 |
| PA14_39960 | secreted_factors | PGD1657274 | phzB2 | DrhlR vs WT | -7.39445 | 3.24E-37 |
| PA14_39970 | secreted_factors | PGD1657276 | phzA2 | DrhlR vs WT | -9.22206 | 1.00E-29 |
| PA14_39980 | transcriptional_regulators | PGD1657278 | qscR | DrhlR vs WT | -1.23189 | 1.26E-06 |
| PA14_39990 | fatty_acid_and_phospholipid | PGD1657280 | NA | DrhlR vs WT | -1.61392 | 3.60E-10 |
| PA14_40010 | hypothetical_unclassified | PGD1657282 | NA | DrhlR vs WT | -2.10165 | 6.50E-25 |
| PA14_40020 | hypothetical_unclassified | PGD1657284 | NA | DrhlR vs WT | -1.53507 | 1.65E-13 |
| PA14_40040 | central_intermediary_metab | PGD1657288 | NA | DrhlR vs WT | -1.66707 | 2.43E-17 |
| PA14_40040 | putative_enzymes | PGD1657288 | NA | DrhlR vs WT | -1.66707 | 2.43E-17 |
| PA14_40050 | hypothetical_unclassified | PGD1657290 | NA | DrhlR vs WT | -1.21645 | 1.84E-09 |
| PA14_40060 | fatty_acid_and_phospholipid | PGD1657292 | NA | DrhlR vs WT | -1.80961 | 1.58E-08 |
| PA14_40060 | membrane_proteins | PGD1657292 | NA | DrhlR vs WT | -1.80961 | 1.58E-08 |
| PA14_40130 | transport_of_small_molecule | PGD1657304 | NA | DrhlR vs WT | -1.80126 | 2.01E-10 |
| PA14_40230 | protein_secretion_export | PGD1657320 | NA | DrhlR vs WT | -1.55877 | 6.27E-13 |
| PA14_40240 | protein_secretion_export | PGD1657322 | NA | DrhlR vs WT | -1.57944 | 2.43E-10 |
| PA14_40250 | membrane_proteins | PGD1657324 | NA | DrhlR vs WT | -1.91344 | 8.27E-23 |
| PA14_40260 | hypothetical_unclassified | PGD1657326 | NA | DrhlR vs WT | -1.97705 | 1.46E-17 |
| PA14_40270 | membrane_proteins | PGD1657328 | NA | DrhlR vs WT | -1.98768 | 2.54E-07 |
| PA14_40270 | transport_of_small_molecule | PGD1657328 | NA | DrhlR vs WT | -1.98768 | 2.54E-07 |
| PA14_40290 | secreted_factors | PGD1657332 | lasA | DrhlR vs WT | -1.63159 | 9.70E-17 |
| PA14_40290 | translation_posttranslational | PGD1657332 | lasA | DrhlR vs WT | -1.63159 | 9.70E-17 |
| PA14_40310 | fatty_acid_and_phospholipid | PGD1657336 | NA | DrhlR vs WT | -5.01988 | 1.33E-35 |
| PA14_40330 | protein_secretion_export | PGD1657340 | NA | DrhlR vs WT | 1.720575 | 0.018647 |

| | | | | | | |
|---|---|---|---|---|---|---|
| PA14_40750 | hypothetical_unclassified | PGD1657404 | NA | DrhlR vs WT | -2.09346 | 1.06E-18 |
| PA14_40850 | carbon_compound_catabolis | PGD1657420 | NA | DrhlR vs WT | -1.02502 | 1.04E-08 |
| PA14_40860 | transport_of_small_molecule | PGD1657422 | NA | DrhlR vs WT | -1.70157 | 5.21E-12 |
| PA14_42080 | fatty_acid_and_phospholipid | PGD1657620 | NA | DrhlR vs WT | 2.079152 | 2.33E-15 |
| PA14_42080 | putative_enzymes | PGD1657620 | NA | DrhlR vs WT | 2.079152 | 2.33E-15 |
| PA14_42090 | fatty_acid_and_phospholipid | PGD1657622 | NA | DrhlR vs WT | 1.888971 | 1.77E-09 |
| PA14_42270 | secreted_factors | PGD1657648 | pscJ | DrhlR vs WT | 1.167485 | 0.00716 |
| PA14_42340 | protein_secretion_export | PGD1657660 | pscD | DrhlR vs WT | 1.267563 | 0.023888 |
| PA14_42390 | transcriptional_regulators | PGD1657668 | exsA | DrhlR vs WT | 1.216147 | 2.81E-05 |
| PA14_42430 | protein_secretion_export | PGD1657674 | exsC | DrhlR vs WT | 1.535656 | 1.32E-08 |
| PA14_42430 | secreted_factors | PGD1657674 | exsC | DrhlR vs WT | 1.535656 | 1.32E-08 |
| PA14_42430 | translation_posttranslational | PGD1657674 | exsC | DrhlR vs WT | 1.535656 | 1.32E-08 |
| PA14_42450 | protein_secretion_export | PGD1657678 | popB | DrhlR vs WT | 1.470173 | 8.44E-04 |
| PA14_42480 | protein_secretion_export | PGD1657684 | pcrG | DrhlR vs WT | 3.948745 | 0.013042 |
| PA14_42570 | protein_secretion_export | PGD1657700 | pscN | DrhlR vs WT | 2.180726 | 0.002938 |
| PA14_42710 | hypothetical_unclassified | PGD1657724 | NA | DrhlR vs WT | -1.43719 | 1.91E-10 |
| PA14_42980 | chaperones_heat_shock | PGD1657772 | clpV2 | DrhlR vs WT | -1.09535 | 1.81E-11 |
| PA14_43050 | hypothetical_unclassified | PGD1657784 | hsiA2 | DrhlR vs WT | -1.11251 | 7.03E-09 |
| PA14_43100 | hypothetical_unclassified | PGD1657792 | rhsP2 | DrhlR vs WT | -1.09863 | 1.31E-06 |
| PA14_43220 | adaptation_and_protection | PGD1657812 | NA | DrhlR vs WT | -1.16615 | 1.27E-09 |
| PA14_43220 | chemotaxis | PGD1657812 | NA | DrhlR vs WT | -1.16615 | 1.27E-09 |
| PA14_43400 | transport_of_small_molecule | PGD1657840 | kdpA | DrhlR vs WT | 1.223426 | 0.047518 |
| PA14_43610 | fatty_acid_and_phospholipid | PGD1657872 | NA | DrhlR vs WT | -1.2905 | 1.32E-13 |
| PA14_43730 | hypothetical_unclassified | PGD1657894 | NA | DrhlR vs WT | 1.727268 | 3.89E-16 |
| PA14_43850 | chaperones_heat_shock | PGD1657914 | htpG | DrhlR vs WT | 1.112737 | 8.43E-12 |
| PA14_44210 | amino_acid_biosynthesis_me | PGD1657974 | NA | DrhlR vs WT | -1.08096 | 6.92E-07 |
| PA14_44311 | hypothetical_unclassified | PGD1657990 | NA | DrhlR vs WT | 1.121191 | 5.36E-05 |
| PA14_44460 | membrane_proteins | PGD1658016 | NA | DrhlR vs WT | -1.25182 | 7.05E-10 |
| PA14_44740 | nucleotide_biosynthesis_and | PGD1658064 | xdhB | DrhlR vs WT | -1.10637 | 8.67E-15 |
| PA14_44960 | putative_enzymes | PGD1658098 | NA | DrhlR vs WT | 1.052287 | 8.71E-04 |
| PA14_45010 | central_intermediary_metab | PGD1658108 | NA | DrhlR vs WT | -1.10213 | 0.021163 |
| PA14_45030 | carbon_compound_catabolis | PGD1658112 | NA | DrhlR vs WT | -1.41116 | 0.018398 |
| PA14_45030 | putative_enzymes | PGD1658112 | NA | DrhlR vs WT | -1.41116 | 0.018398 |
| PA14_45100 | transcriptional_regulators | PGD1658122 | NA | DrhlR vs WT | 1.290709 | 3.87E-05 |
| PA14_45100 | transport_of_small_molecule | PGD1658122 | NA | DrhlR vs WT | 1.290709 | 3.87E-05 |
| PA14_45950 | adaptation_and_protection | PGD1658246 | rsaL | DrhlR vs WT | -1.16321 | 6.71E-04 |
| PA14_45950 | transcriptional_regulators | PGD1658246 | rsaL | DrhlR vs WT | -1.16321 | 6.71E-04 |
| PA14_46250 | putative_enzymes | PGD1658292 | NA | DrhlR vs WT | 1.078986 | 2.79E-08 |
| PA14_46280 | hypothetical_unclassified | PGD1658298 | NA | DrhlR vs WT | 1.105177 | 4.45E-06 |
| PA14_47080 | transcriptional_regulators | PGD1658438 | NA | DrhlR vs WT | -1.09476 | 4.64E-05 |
| PA14_47120 | hypothetical_unclassified | PGD1658446 | NA | DrhlR vs WT | 1.629205 | 1.27E-13 |
| PA14_47230 | transport_of_small_molecule | PGD1658462 | NA | DrhlR vs WT | 1.161214 | 8.72E-05 |
| PA14_47380 | transport_of_small_molecule | PGD1658490 | NA | DrhlR vs WT | 1.599414 | 6.38E-08 |
| PA14_47390 | two_component_regulators | PGD1658492 | NA | DrhlR vs WT | 2.007968 | 9.88E-09 |
| PA14_47400 | transcriptional_regulators | PGD1658494 | NA | DrhlR vs WT | 2.08096 | 1.22E-09 |
| PA14_47860 | putative_enzymes | PGD1658562 | NA | DrhlR vs WT | -1.1644 | 0.00171 |
| PA14_47950 | transport_of_small_molecule | PGD1658580 | NA | DrhlR vs WT | 3.943209 | 0.018645 |
| PA14_47960 | transport_of_small_molecule | PGD1658582 | NA | DrhlR vs WT | 1.885652 | 0.001458 |
| PA14_48170 | motility_and_attachment | PGD1658610 | srpP | DrhlR vs WT | 1.043435 | 1.61E-09 |
| PA14_48170 | translation_posttranslational | PGD1658610 | srpP | DrhlR vs WT | 1.043435 | 1.61E-09 |

| | | | | | | |
|---|---|---|---|---|---|---|
| PA14_48420 | transcriptional_regulators | PGD1658644 | NA | DrhlR vs WT | -1.11886 | 4.94E-08 |
| PA14_48440 | putative_enzymes | PGD1658646 | NA | DrhlR vs WT | -1.01761 | 0.010694 |
| PA14_48530 | putative_enzymes | PGD1658662 | NA | DrhlR vs WT | -2.65628 | 7.03E-09 |
| PA14_48560 | hypothetical_unclassified | PGD1658668 | NA | DrhlR vs WT | -1.73938 | 0.017305 |
| PA14_48570 | amino_acid_biosynthesis_me | PGD1658670 | NA | DrhlR vs WT | -1.89149 | 1.62E-04 |
| PA14_48590 | hypothetical_unclassified | PGD1658672 | NA | DrhlR vs WT | -2.01241 | 4.81E-04 |
| PA14_48600 | putative_enzymes | PGD1658674 | NA | DrhlR vs WT | -2.19856 | 5.99E-05 |
| PA14_48610 | amino_acid_biosynthesis_me | PGD1658676 | NA | DrhlR vs WT | -3.20537 | 0.002199 |
| PA14_48620 | central_intermediary_metab | PGD1658678 | NA | DrhlR vs WT | -1.91808 | 0.00489 |
| PA14_49020 | relative_phage_transposon | PGD1658750 | pf5r | DrhlR vs WT | -1.1449 | 0.018408 |
| PA14_49080 | putative_enzymes | PGD1658762 | NA | DrhlR vs WT | 1.200649 | 7.49E-04 |
| PA14_49260 | energy_metabolism_CAP | PGD1658790 | napB | DrhlR vs WT | -1.11928 | 1.28E-12 |
| PA14_49390 | transcription_RNA_processin | PGD1658814 | rrmA | DrhlR vs WT | 1.63951 | 5.61E-07 |
| PA14_49480 | hypothetical_unclassified | PGD1658828 | NA | DrhlR vs WT | -1.03083 | 0.036686 |
| PA14_49560 | secreted_factors | PGD1658840 | toxA | DrhlR vs WT | 3.924973 | 2.94E-34 |
| PA14_49570 | membrane_proteins | PGD1658842 | NA | DrhlR vs WT | 1.302078 | 0.00138 |
| PA14_49690 | putative_enzymes | PGD1658862 | NA | DrhlR vs WT | 2.068118 | 3.36E-05 |
| PA14_49720 | hypothetical_unclassified | PGD1658868 | NA | DrhlR vs WT | 2.588734 | 1.20E-11 |
| PA14_49750 | antibiotic_resistance_and_su | PGD1658874 | NA | DrhlR vs WT | -5.24938 | 1.25E-53 |
| PA14_49750 | membrane_proteins | PGD1658874 | NA | DrhlR vs WT | -5.24938 | 1.25E-53 |
| PA14_49750 | transport_of_small_molecule | PGD1658874 | NA | DrhlR vs WT | -5.24938 | 1.25E-53 |
| PA14_49760 | adaptation_and_protection | PGD1658876 | rhlC | DrhlR vs WT | -5.24017 | 6.61E-18 |
| PA14_49800 | putative_enzymes | PGD1658882 | NA | DrhlR vs WT | -1.28273 | 1.04E-08 |
| PA14_49860 | hypothetical_unclassified | PGD1658892 | NA | DrhlR vs WT | -1.14785 | 3.40E-08 |
| PA14_51310 | hypothetical_unclassified | PGD1659136 | NA | DrhlR vs WT | 1.138437 | 1.62E-09 |
| PA14_51350 | adaptation_and_protection | PGD1659144 | phnB | DrhlR vs WT | 2.593378 | 7.44E-82 |
| PA14_51350 | amino_acid_biosynthesis_me | PGD1659144 | phnB | DrhlR vs WT | 2.593378 | 7.44E-82 |
| PA14_51360 | adaptation_and_protection | PGD1659146 | phnA | DrhlR vs WT | 3.416665 | 2.12E-203 |
| PA14_51380 | biosynthesis_of_cofactors | PGD1659148 | pqsE | DrhlR vs WT | 3.094706 | 7.04E-81 |
| PA14_51390 | hypothetical_unclassified | PGD1659150 | pqsD | DrhlR vs WT | 3.552066 | 3.69E-241 |
| PA14_51410 | biosynthesis_of_cofactors | PGD1659152 | pqsC | DrhlR vs WT | 3.712773 | 1.46E-149 |
| PA14_51420 | biosynthesis_of_cofactors | PGD1659154 | pqsB | DrhlR vs WT | 3.251741 | 2.49E-162 |
| PA14_51430 | biosynthesis_of_cofactors | PGD1659156 | pqsA | DrhlR vs WT | 3.191951 | 1.97E-212 |
| PA14_51620 | relative_phage_transposon | PGD1659194 | NA | DrhlR vs WT | 1.129448 | 1.26E-06 |
| PA14_51830 | adaptation_and_protection | PGD1659232 | NA | DrhlR vs WT | 1.149894 | 6.05E-12 |
| PA14_51940 | hypothetical_unclassified | PGD1659252 | NA | DrhlR vs WT | 2.697313 | 1.93E-19 |
| PA14_51950 | hypothetical_unclassified | PGD1659254 | NA | DrhlR vs WT | 2.171605 | 4.40E-18 |
| PA14_52070 | transcriptional_regulators | PGD1659274 | NA | DrhlR vs WT | 1.028822 | 1.23E-05 |
| PA14_52080 | hypothetical_unclassified | PGD1659276 | NA | DrhlR vs WT | 2.253108 | 4.38E-12 |
| PA14_52090 | hypothetical_unclassified | PGD1659278 | NA | DrhlR vs WT | 2.293353 | 1.32E-09 |
| PA14_52130 | hypothetical_unclassified | PGD1659284 | NA | DrhlR vs WT | -1.60991 | 2.30E-20 |
| PA14_52250 | transport_of_small_molecule | PGD1659302 | NA | DrhlR vs WT | 1.393758 | 6.16E-07 |
| PA14_52250 | two_component_regulators | PGD1659302 | NA | DrhlR vs WT | 1.393758 | 6.16E-07 |
| PA14_52290 | hypothetical_unclassified | PGD1659310 | NA | DrhlR vs WT | 1.226828 | 5.83E-10 |
| PA14_52480 | hypothetical_unclassified | PGD1659340 | NA | DrhlR vs WT | 1.180217 | 0.038291 |
| PA14_52490 | hypothetical_unclassified | PGD1659342 | NA | DrhlR vs WT | 1.763296 | 0.022988 |
| PA14_52660 | amino_acid_biosynthesis_me | PGD1659370 | aruB | DrhlR vs WT | -1.00389 | 2.60E-07 |
| PA14_52880 | putative_enzymes | PGD1659406 | NA | DrhlR vs WT | 3.880392 | 0.009806 |
| PA14_52960 | hypothetical_unclassified | PGD1659420 | NA | DrhlR vs WT | 1.03686 | 0.032437 |
| PA14_53020 | cell_wall_LPS | PGD1659430 | pbpG | DrhlR vs WT | -1.01277 | 8.45E-09 |

| | | | | | | |
|---|---|---|---|---|---|---|
| PA14_53250 | secreted_factors | PGD1659466 | cpbD | DrhlR vs WT | -2.75619 | 1.44E-58 |
| PA14_53290 | nucleotide_biosynthesis_and | PGD1659472 | trxB2 | DrhlR vs WT | -2.34029 | 9.14E-21 |
| PA14_53380 | putative_enzymes | PGD1659486 | NA | DrhlR vs WT | 1.767076 | 4.69E-04 |
| PA14_53530 | hypothetical_unclassified | PGD1659512 | NA | DrhlR vs WT | 1.33144 | 1.35E-05 |
| PA14_53590 | hypothetical_unclassified | PGD1659524 | NA | DrhlR vs WT | -1.05465 | 0.001604 |
| PA14_53690 | hypothetical_unclassified | PGD1659544 | NA | DrhlR vs WT | 2.122825 | 7.24E-07 |
| PA14_53700 | putative_enzymes | PGD1659546 | NA | DrhlR vs WT | 1.159129 | 0.014457 |
| PA14_53840 | hypothetical_unclassified | PGD1659570 | NA | DrhlR vs WT | -1.1103 | 0.001289 |
| PA14_53890 | membrane_proteins | PGD1659580 | NA | DrhlR vs WT | 1.010439 | 0.029257 |
| PA14_53920 | transcriptional_regulators | PGD1659586 | NA | DrhlR vs WT | 1.431336 | 1.27E-11 |
| PA14_53940 | carbon_compound_catabolis | PGD1659588 | prpB | DrhlR vs WT | 1.657253 | 1.14E-08 |
| PA14_53940 | central_intermediary_metab | PGD1659588 | prpB | DrhlR vs WT | 1.657253 | 1.14E-08 |
| PA14_53940 | fatty_acid_and_phospholipid | PGD1659588 | prpB | DrhlR vs WT | 1.657253 | 1.14E-08 |
| PA14_53950 | carbon_compound_catabolis | PGD1659590 | prpC | DrhlR vs WT | 1.356031 | 4.88E-10 |
| PA14_53950 | central_intermediary_metab | PGD1659590 | prpC | DrhlR vs WT | 1.356031 | 4.88E-10 |
| PA14_54220 | hypothetical_unclassified | PGD1659628 | icp | DrhlR vs WT | 1.025819 | 2.37E-04 |
| PA14_54520 | membrane_proteins | PGD1659676 | NA | DrhlR vs WT | 1.730303 | 3.01E-08 |
| PA14_54570 | membrane_proteins | PGD1659682 | NA | DrhlR vs WT | 1.067744 | 0.003186 |
| PA14_54720 | hypothetical_unclassified | PGD1659710 | NA | DrhlR vs WT | 1.253885 | 9.66E-04 |
| PA14_54740 | hypothetical_unclassified | PGD1659714 | NA | DrhlR vs WT | 1.13428 | 6.17E-05 |
| PA14_55040 | transport_of_small_molecule | PGD1659768 | NA | DrhlR vs WT | 1.169185 | 0.018249 |
| PA14_55080 | hypothetical_unclassified | PGD1659776 | NA | DrhlR vs WT | -1.30581 | 3.09E-18 |
| PA14_55100 | hypothetical_unclassified | PGD1659780 | NA | DrhlR vs WT | 1.345006 | 1.05E-05 |
| PA14_55110 | hypothetical_unclassified | PGD1659782 | NA | DrhlR vs WT | 1.040448 | 0.001282 |
| PA14_55160 | transcriptional_regulators | PGD1659792 | toxR | DrhlR vs WT | 1.604393 | 4.60E-07 |
| PA14_55360 | membrane_proteins | PGD1659822 | exbB2 | DrhlR vs WT | -1.0244 | 0.017511 |
| PA14_55360 | transport_of_small_molecule | PGD1659822 | exbB2 | DrhlR vs WT | -1.0244 | 0.017511 |
| PA14_55540 | transcriptional_regulators | PGD1659854 | NA | DrhlR vs WT | 1.269437 | 4.66E-04 |
| PA14_55580 | biosynthesis_of_cofactors | PGD1659862 | nemO | DrhlR vs WT | 2.071944 | 1.75E-11 |
| PA14_55600 | hypothetical_unclassified | PGD1659866 | NA | DrhlR vs WT | -1.25045 | 3.29E-05 |
| PA14_55730 | transcriptional_regulators | PGD1659894 | NA | DrhlR vs WT | 2.032347 | 1.16E-06 |
| PA14_55780 | two_component_regulators | PGD1659904 | NA | DrhlR vs WT | -1.26206 | 4.81E-15 |
| PA14_55800 | protein_secretion_export | PGD1659908 | NA | DrhlR vs WT | -1.48074 | 1.60E-05 |
| PA14_55840 | hypothetical_unclassified | PGD1659914 | NA | DrhlR vs WT | -1.74659 | 0.025767 |
| PA14_55880 | membrane_proteins | PGD1659920 | NA | DrhlR vs WT | -1.40795 | 5.41E-09 |
| PA14_55880 | motility_and_attachment | PGD1659920 | NA | DrhlR vs WT | -1.40795 | 5.41E-09 |
| PA14_55880 | protein_secretion_export | PGD1659920 | NA | DrhlR vs WT | -1.40795 | 5.41E-09 |
| PA14_55940 | protein_secretion_export | PGD1659930 | NA | DrhlR vs WT | -3.13157 | 4.74E-23 |
| PA14_56070 | transcriptional_regulators | PGD1659948 | mvaT | DrhlR vs WT | -1.22467 | 7.56E-07 |
| PA14_56090 | membrane_proteins | PGD1659952 | NA | DrhlR vs WT | 1.166608 | 1.34E-10 |
| PA14_56550 | hypothetical_unclassified | PGD1660016 | NA | DrhlR vs WT | -1.13143 | 2.65E-08 |
| PA14_56570 | fatty_acid_and_phospholipid | PGD1660020 | NA | DrhlR vs WT | -1.62729 | 0.001351 |
| PA14_56590 | adaptation_and_protection | PGD1660022 | NA | DrhlR vs WT | -1.95165 | 1.77E-12 |
| PA14_56640 | transport_of_small_molecule | PGD1660028 | NA | DrhlR vs WT | -1.21719 | 0.010136 |
| PA14_56670 | hypothetical_unclassified | PGD1660032 | NA | DrhlR vs WT | -1.25587 | 6.82E-05 |
| PA14_56750 | hypothetical_unclassified | PGD1660046 | NA | DrhlR vs WT | 1.212518 | 0.007557 |
| PA14_56780 | adaptation_and_protection | PGD1660050 | sodB | DrhlR vs WT | -1.04034 | 2.40E-09 |
| PA14_56910 | hypothetical_unclassified | PGD1660072 | NA | DrhlR vs WT | -1.16267 | 4.99E-06 |
| PA14_56970 | hypothetical_unclassified | PGD1660084 | NA | DrhlR vs WT | -1.28839 | 4.85E-11 |
| PA14_56990 | hypothetical_unclassified | PGD1660088 | NA | DrhlR vs WT | -2.95555 | 1.57E-45 |

| | | | | | | |
|---|---|---|---|---|---|---|
| PA14_57010 | chaperones_heat_shock | PGD1660090 | groEL | DrhlR vs WT | 1.027288 | 4.32E-11 |
| PA14_57020 | chaperones_heat_shock | PGD1660092 | groES | DrhlR vs WT | 1.270783 | 5.36E-13 |
| PA14_57030 | membrane_proteins | PGD1660094 | fxsA | DrhlR vs WT | 1.39711 | 6.24E-08 |
| PA14_57060 | hypothetical_unclassified | PGD1660100 | NA | DrhlR vs WT | 1.034037 | 1.52E-08 |
| PA14_57110 | transport_of_small_molecule | PGD1660108 | NA | DrhlR vs WT | 1.399152 | 1.40E-17 |
| PA14_57990 | transport_of_small_molecule | PGD1660253 | NA | DrhlR vs WT | 2.951303 | 4.48E-10 |
| PA14_58000 | adaptation_and_protection | PGD1660255 | sodM | DrhlR vs WT | 3.258083 | 1.10E-12 |
| PA14_58010 | hypothetical_unclassified | PGD1660257 | NA | DrhlR vs WT | 3.119878 | 9.25E-09 |
| PA14_58030 | energy_metabolism_CAP | PGD1660259 | fumC | DrhlR vs WT | 3.052993 | 5.10E-16 |
| PA14_58040 | hypothetical_unclassified | PGD1660261 | NA | DrhlR vs WT | 3.304797 | 5.78E-12 |
| PA14_58080 | putative_enzymes | PGD1660269 | NA | DrhlR vs WT | -1.16396 | 1.03E-08 |
| PA14_58500 | membrane_proteins | PGD1660333 | NA | DrhlR vs WT | 1.727075 | 8.07E-07 |
| PA14_58580 | membrane_proteins | PGD1660349 | NA | DrhlR vs WT | 2.079671 | 5.39E-10 |
| PA14_58630 | amino_acid_biosynthesis_me | PGD1660357 | NA | DrhlR vs WT | -1.3438 | 1.43E-21 |
| PA14_58720 | hypothetical_unclassified | PGD1660371 | NA | DrhlR vs WT | 1.460318 | 3.22E-09 |
| PA14_58730 | motility_and_attachment | PGD1660373 | pilA | DrhlR vs WT | 1.075607 | 2.69E-05 |
| PA14_58770 | motility_and_attachment | PGD1660381 | pilD | DrhlR vs WT | 1.031722 | 4.73E-08 |
| PA14_58770 | protein_secretion_export | PGD1660381 | pilD | DrhlR vs WT | 1.031722 | 4.73E-08 |
| PA14_58770 | secreted_factors | PGD1660381 | pilD | DrhlR vs WT | 1.031722 | 4.73E-08 |
| PA14_58790 | hypothetical_unclassified | PGD1660385 | NA | DrhlR vs WT | 1.213187 | 0.001086 |
| PA14_59120 | hypothetical_unclassified | PGD1660447 | NA | DrhlR vs WT | -1.4647 | 0.002199 |
| PA14_59360 | motility_and_attachment | PGD1660489 | pilM2 | DrhlR vs WT | -1.64936 | 2.38E-07 |
| PA14_59390 | hypothetical_unclassified | PGD1660495 | NA | DrhlR vs WT | -1.51684 | 1.31E-06 |
| PA14_59840 | hypothetical_unclassified | PGD1660574 | NA | DrhlR vs WT | -1.15255 | 7.18E-04 |
| PA14_59860 | protein_secretion_export | PGD1660580 | NA | DrhlR vs WT | 3.563367 | 0.042826 |
| PA14_59890 | hypothetical_unclassified | PGD1660586 | NA | DrhlR vs WT | 2.331761 | 7.16E-04 |
| PA14_60280 | motility_and_attachment | PGD1660660 | fimU | DrhlR vs WT | 1.494977 | 2.75E-17 |
| PA14_60290 | motility_and_attachment | PGD1660662 | pilW | DrhlR vs WT | 1.051392 | 1.40E-08 |
| PA14_60300 | motility_and_attachment | PGD1660664 | pilX | DrhlR vs WT | 1.078827 | 2.42E-07 |
| PA14_60480 | hypothetical_unclassified | PGD1660696 | NA | DrhlR vs WT | 2.891662 | 1.33E-09 |
| PA14_60520 | hypothetical_unclassified | PGD1660702 | NA | DrhlR vs WT | -1.49739 | 3.70E-28 |
| PA14_60730 | membrane_proteins | PGD1660736 | NA | DrhlR vs WT | -1.04191 | 5.21E-08 |
| PA14_60730 | transport_of_small_molecule | PGD1660736 | NA | DrhlR vs WT | -1.04191 | 5.21E-08 |
| PA14_60750 | carbon_compound_catabolis | PGD1660738 | pra | DrhlR vs WT | -1.13211 | 0.00138 |
| PA14_61010 | hypothetical_unclassified | PGD1660780 | NA | DrhlR vs WT | -1.22661 | 3.57E-06 |
| PA14_61080 | transport_of_small_molecule | PGD1660790 | NA | DrhlR vs WT | 1.932931 | 2.52E-11 |
| PA14_61370 | hypothetical_unclassified | PGD1660838 | NA | DrhlR vs WT | 1.479736 | 1.21E-04 |
| PA14_61410 | hypothetical_unclassified | PGD1660846 | NA | DrhlR vs WT | -1.03288 | 6.93E-09 |
| NA | NA | PGD1660915 | NA | DrhlR vs WT | -1.34607 | 0.006298 |
| PA14_61870 | hypothetical_unclassified | PGD1660922 | NA | DrhlR vs WT | -1.62641 | 1.05E-15 |
| PA14_61910 | hypothetical_unclassified | PGD1660928 | NA | DrhlR vs WT | -1.03081 | 3.09E-05 |
| PA14_62110 | putative_enzymes | PGD1660964 | NA | DrhlR vs WT | 1.237253 | 0.006668 |
| PA14_62240 | hypothetical_unclassified | PGD1660984 | NA | DrhlR vs WT | -1.07105 | 3.83E-06 |
| PA14_62810 | membrane_proteins | PGD1661080 | secG | DrhlR vs WT | -1.05025 | 3.41E-04 |
| PA14_62810 | protein_secretion_export | PGD1661080 | secG | DrhlR vs WT | -1.05025 | 3.41E-04 |
| PA14_62990 | chaperones_heat_shock | PGD1661110 | grpE | DrhlR vs WT | 1.666353 | 4.41E-21 |
| PA14_62990 | DNA_replication_recombinat | PGD1661110 | grpE | DrhlR vs WT | 1.666353 | 4.41E-21 |
| PA14_63110 | amino_acid_biosynthesis_me | PGD1661132 | NA | DrhlR vs WT | 1.019252 | 0.010442 |
| PA14_63120 | amino_acid_biosynthesis_me | PGD1661134 | NA | DrhlR vs WT | 1.347096 | 0.004566 |
| PA14_63280 | transcriptional_regulators | PGD1661160 | NA | DrhlR vs WT | -1.38599 | 2.19E-20 |

| | | | | | | |
|---|---|---|---|---|---|---|
| PA14_63700 | hypothetical_unclassified | PGD1661226 | NA | DrhlR vs WT | -1.74181 | 0.005509 |
| PA14_63740 | hypothetical_unclassified | PGD1661234 | NA | DrhlR vs WT | -1.14836 | 0.00728 |
| PA14_63860 | hypothetical_unclassified | PGD1661252 | NA | DrhlR vs WT | -1.1407 | 1.16E-04 |
| PA14_64050 | transcriptional_regulators | PGD1661278 | NA | DrhlR vs WT | 1.498059 | 3.24E-14 |
| PA14_64050 | two_component_regulators | PGD1661278 | NA | DrhlR vs WT | 1.498059 | 3.24E-14 |
| PA14_64460 | adaptation_and_protection | PGD1661344 | NA | DrhlR vs WT | -1.12744 | 9.77E-14 |
| PA14_64480 | membrane_proteins | PGD1661348 | osmE | DrhlR vs WT | 1.936466 | 3.16E-22 |
| PA14_64690 | membrane_proteins | PGD1661386 | NA | DrhlR vs WT | 1.23861 | 0.008851 |
| PA14_64700 | transcriptional_regulators | PGD1661388 | NA | DrhlR vs WT | 1.827963 | 1.22E-05 |
| PA14_64750 | membrane_proteins | PGD1661396 | NA | DrhlR vs WT | -1.89674 | 3.25E-08 |
| PA14_64750 | transport_of_small_molecule | PGD1661396 | NA | DrhlR vs WT | -1.89674 | 3.25E-08 |
| PA14_64930 | nucleotide_biosynthesis_and | PGD1661428 | NA | DrhlR vs WT | -3.10817 | 2.16E-57 |
| PA14_64940 | biosynthesis_of_cofactors | PGD1661430 | NA | DrhlR vs WT | -4.63415 | 3.84E-55 |
| PA14_64960 | biosynthesis_of_cofactors | PGD1661434 | pncB1 | DrhlR vs WT | 1.047232 | 2.35E-07 |
| PA14_65000 | energy_metabolism_CAP | PGD1661440 | azu | DrhlR vs WT | 1.56933 | 2.75E-23 |
| PA14_65690 | nucleotide_biosynthesis_and | PGD1661538 | NA | DrhlR vs WT | 1.347785 | 4.32E-11 |
| PA14_65690 | putative_enzymes | PGD1661538 | NA | DrhlR vs WT | 1.347785 | 4.32E-11 |
| PA14_65920 | transport_of_small_molecule | PGD1661572 | NA | DrhlR vs WT | -1.16966 | 0.023788 |
| PA14_66340 | hypothetical_unclassified | PGD1661640 | NA | DrhlR vs WT | -1.10266 | 5.88E-11 |
| PA14_66450 | hypothetical_unclassified | PGD1661654 | NA | DrhlR vs WT | 2.091147 | 6.81E-21 |
| PA14_66460 | adaptation_and_protection | PGD1661656 | NA | DrhlR vs WT | -1.99484 | 2.26E-15 |
| PA14_66530 | transcriptional_regulators | PGD1661666 | NA | DrhlR vs WT | -1.55995 | 7.23E-06 |
| PA14_66540 | hypothetical_unclassified | PGD1661668 | NA | DrhlR vs WT | 2.080202 | 1.76E-17 |
| PA14_66640 | motility_and_attachment | PGD1661686 | pilO | DrhlR vs WT | 1.296731 | 2.70E-10 |
| PA14_66650 | motility_and_attachment | PGD1661688 | pilN | DrhlR vs WT | 1.153346 | 1.85E-07 |
| PA14_66770 | chaperones_heat_shock | PGD1661708 | hslV | DrhlR vs WT | 1.766243 | 2.75E-23 |
| PA14_66790 | chaperones_heat_shock | PGD1661710 | hslU | DrhlR vs WT | 1.279424 | 1.51E-08 |
| PA14_66850 | transcriptional_regulators | PGD1661720 | NA | DrhlR vs WT | -1.01269 | 2.35E-07 |
| PA14_67150 | amino_acid_biosynthesis_me | PGD1661770 | NA | DrhlR vs WT | 1.117992 | 1.71E-05 |
| PA14_67320 | amino_acid_biosynthesis_me | PGD1661800 | hutH | DrhlR vs WT | 1.481932 | 1.29E-08 |
| PA14_67320 | central_intermediary_metab | PGD1661800 | hutH | DrhlR vs WT | 1.481932 | 1.29E-08 |
| PA14_67440 | putative_enzymes | PGD1661816 | NA | DrhlR vs WT | -1.08064 | 2.90E-11 |
| PA14_67540 | hypothetical_unclassified | PGD1661834 | NA | DrhlR vs WT | -1.53105 | 1.09E-05 |
| PA14_67550 | transcriptional_regulators | PGD1661836 | NA | DrhlR vs WT | -1.08544 | 0.0051 |
| PA14_67640 | hypothetical_unclassified | PGD1661848 | NA | DrhlR vs WT | 1.28728 | 2.72E-09 |
| PA14_67840 | transport_of_small_molecule | PGD1661878 | NA | DrhlR vs WT | 1.383735 | 2.79E-10 |
| PA14_67850 | transport_of_small_molecule | PGD1661880 | NA | DrhlR vs WT | 1.316855 | 8.14E-08 |
| PA14_67860 | transport_of_small_molecule | PGD1661882 | NA | DrhlR vs WT | 2.076386 | 7.16E-14 |
| PA14_68170 | cell_wall_LPS | PGD1661934 | rmlB | DrhlR vs WT | -2.23757 | 1.20E-88 |
| PA14_68190 | cell_wall_LPS | PGD1661936 | rmlD | DrhlR vs WT | -2.33013 | 3.89E-54 |
| PA14_68200 | cell_wall_LPS | PGD1661938 | rmlA | DrhlR vs WT | -1.43289 | 3.44E-21 |
| PA14_68210 | cell_wall_LPS | PGD1661940 | rmlC | DrhlR vs WT | -1.60673 | 5.14E-34 |
| PA14_68300 | amino_acid_biosynthesis_me | PGD1661952 | arcD | DrhlR vs WT | 1.6247 | 2.40E-08 |
| PA14_68300 | membrane_proteins | PGD1661952 | arcD | DrhlR vs WT | 1.6247 | 2.40E-08 |
| PA14_68300 | transport_of_small_molecule | PGD1661952 | arcD | DrhlR vs WT | 1.6247 | 2.40E-08 |
| PA14_68630 | chaperones_heat_shock | PGD1662004 | NA | DrhlR vs WT | -1.79014 | 1.09E-06 |
| PA14_68930 | membrane_proteins | PGD1662052 | NA | DrhlR vs WT | -5.28053 | 1.93E-22 |
| PA14_68930 | transport_of_small_molecule | PGD1662052 | NA | DrhlR vs WT | -5.28053 | 1.93E-22 |
| PA14_68940 | hypothetical_unclassified | PGD1662054 | NA | DrhlR vs WT | -5.96063 | 9.81E-45 |
| PA14_69270 | biosynthesis_of_cofactors | PGD1662106 | NA | DrhlR vs WT | 1.084991 | 1.13E-04 |

| | | | | | | |
|---|---|---|---|---|---|---|
| PA14_69600 | hypothetical_unclassified | PGD1662160 | NA | DrhlR vs WT | -1.48688 | 8.05E-13 |
| PA14_69660 | cell_wall_LPS | PGD1662170 | lppL | DrhlR vs WT | -1.01101 | 7.04E-04 |
| PA14_69660 | fatty_acid_and_phospholipid | PGD1662170 | lppL | DrhlR vs WT | -1.01101 | 7.04E-04 |
| PA14_69780 | hypothetical_unclassified | PGD1662190 | NA | DrhlR vs WT | -1.31631 | 6.63E-14 |
| PA14_69925 | central_intermediary_metab | PGD1662212 | poxB | DrhlR vs WT | 1.664531 | 1.03E-07 |
| PA14_69925 | energy_metabolism_CAP | PGD1662212 | poxB | DrhlR vs WT | 1.664531 | 1.03E-07 |
| PA14_70050 | hypothetical_unclassified | PGD1662228 | NA | DrhlR vs WT | -1.15043 | 4.86E-13 |
| PA14_70100 | amino_acid_biosynthesis_me | PGD1662236 | NA | DrhlR vs WT | 1.082579 | 1.58E-09 |
| PA14_70100 | carbon_compound_catabolis | PGD1662236 | NA | DrhlR vs WT | 1.082579 | 1.58E-09 |
| PA14_70100 | putative_enzymes | PGD1662236 | NA | DrhlR vs WT | 1.082579 | 1.58E-09 |
| PA14_70740 | protein_secretion_export | PGD1662336 | NA | DrhlR vs WT | -1.00452 | 7.46E-09 |
| PA14_70750 | transcriptional_regulators | PGD1662338 | phoB | DrhlR vs WT | 1.086125 | 2.51E-07 |
| PA14_70750 | two_component_regulators | PGD1662338 | phoB | DrhlR vs WT | 1.086125 | 2.51E-07 |
| PA14_70810 | transport_of_small_molecule | PGD1662350 | pstB | DrhlR vs WT | 1.134654 | 4.18E-04 |
| PA14_70850 | membrane_proteins | PGD1662354 | pstC | DrhlR vs WT | 1.301641 | 8.84E-05 |
| PA14_70850 | transport_of_small_molecule | PGD1662354 | pstC | DrhlR vs WT | 1.301641 | 8.84E-05 |
| PA14_70860 | transport_of_small_molecule | PGD1662356 | NA | DrhlR vs WT | 1.521508 | 4.97E-04 |
| PA14_71060 | amino_acid_biosynthesis_me | PGD1662386 | sdaB | DrhlR vs WT | -1.50115 | 1.29E-08 |
| PA14_71080 | hypothetical_unclassified | PGD1662390 | NA | DrhlR vs WT | -1.56831 | 3.25E-08 |
| PA14_71100 | membrane_proteins | PGD1662394 | NA | DrhlR vs WT | 2.123028 | 5.59E-05 |
| PA14_71240 | amino_acid_biosynthesis_me | PGD1662420 | NA | DrhlR vs WT | -1.57553 | 0.001534 |
| PA14_71250 | hypothetical_unclassified | PGD1662422 | NA | DrhlR vs WT | -2.11592 | 0.001553 |
| PA14_71260 | amino_acid_biosynthesis_me | PGD1662424 | NA | DrhlR vs WT | -1.22163 | 4.85E-07 |
| PA14_71260 | energy_metabolism_CAP | PGD1662424 | NA | DrhlR vs WT | -1.22163 | 4.85E-07 |
| PA14_71280 | amino_acid_biosynthesis_me | PGD1662426 | NA | DrhlR vs WT | -1.83811 | 1.16E-06 |
| PA14_71280 | energy_metabolism_CAP | PGD1662426 | NA | DrhlR vs WT | -1.83811 | 1.16E-06 |
| PA14_71300 | energy_metabolism_CAP | PGD1662428 | NA | DrhlR vs WT | -1.57952 | 0.00722 |
| PA14_71410 | transport_of_small_molecule | PGD1662450 | NA | DrhlR vs WT | -3.55715 | 9.84E-08 |
| PA14_71420 | energy_metabolism_CAP | PGD1662452 | NA | DrhlR vs WT | -1.52818 | 1.30E-05 |
| PA14_71460 | amino_acid_biosynthesis_me | PGD1662460 | glyA1 | DrhlR vs WT | -3.29002 | 4.71E-09 |
| PA14_71470 | amino_acid_biosynthesis_me | PGD1662462 | soxB | DrhlR vs WT | -2.14749 | 1.20E-08 |
| PA14_71470 | carbon_compound_catabolis | PGD1662462 | soxB | DrhlR vs WT | -2.14749 | 1.20E-08 |
| PA14_71490 | amino_acid_biosynthesis_me | PGD1662464 | soxD | DrhlR vs WT | -1.82163 | 0.037773 |
| PA14_71490 | carbon_compound_catabolis | PGD1662464 | soxD | DrhlR vs WT | -1.82163 | 0.037773 |
| PA14_71500 | carbon_compound_catabolis | PGD1662466 | soxA | DrhlR vs WT | -2.49934 | 1.35E-11 |
| PA14_71510 | amino_acid_biosynthesis_me | PGD1662468 | soxG | DrhlR vs WT | -1.87831 | 4.30E-04 |
| PA14_71510 | carbon_compound_catabolis | PGD1662468 | soxG | DrhlR vs WT | -1.87831 | 4.30E-04 |
| PA14_71530 | nucleotide_biosynthesis_and | PGD1662470 | purU2 | DrhlR vs WT | -2.06204 | 5.92E-05 |
| PA14_71560 | amino_acid_biosynthesis_me | PGD1662472 | fdhA | DrhlR vs WT | -2.46687 | 1.25E-09 |
| PA14_71580 | hypothetical_unclassified | PGD1662476 | NA | DrhlR vs WT | 1.252113 | 3.61E-10 |
| PA14_72260 | transport_of_small_molecule | PGD1662584 | NA | DrhlR vs WT | -1.17255 | 6.39E-06 |
| PA14_72760 | adaptation_and_protection | PGD1662662 | NA | DrhlR vs WT | 1.001529 | 1.90E-09 |
| PA14_72760 | antibiotic_resistance_and_su | PGD1662662 | NA | DrhlR vs WT | 1.001529 | 1.90E-09 |
| PA14_72760 | putative_enzymes | PGD1662662 | NA | DrhlR vs WT | 1.001529 | 1.90E-09 |
| PA14_72830 | hypothetical_unclassified | PGD1662676 | NA | DrhlR vs WT | -1.07436 | 7.43E-04 |
| PA14_72880 | biosynthesis_of_cofactors | PGD1662684 | NA | DrhlR vs WT | -1.41009 | 5.37E-11 |
| PA14_72890 | transcriptional_regulators | PGD1662686 | NA | DrhlR vs WT | -1.59821 | 2.68E-19 |
| PA14_72970 | transport_of_small_molecule | PGD1662698 | tonB | DrhlR vs WT | 1.111355 | 4.71E-04 |
| PA14_73000 | hypothetical_unclassified | PGD1662704 | NA | DrhlR vs WT | -1.18436 | 0.007809 |
| PA14_00050 | DNA_replication_recombinat | PGD1650841 | gyrB | Dcrc vs WT | 1.013031 | 2.41E-10 |

| | | | | | | |
|---|---|---|---|---|---|---|
| PA14_00100 | amino_acid_biosynthesis_me | PGD1650851 | glyQ | Dcrc vs WT | 1.412525 | 5.12E-05 |
| PA14_00100 | translation_posttranslational | PGD1650851 | glyQ | Dcrc vs WT | 1.412525 | 5.12E-05 |
| PA14_00190 | amino_acid_biosynthesis_me | PGD1650869 | fmt | Dcrc vs WT | 1.146597 | 1.34E-04 |
| PA14_00190 | translation_posttranslational | PGD1650869 | fmt | Dcrc vs WT | 1.146597 | 1.34E-04 |
| PA14_00310 | translation_posttranslational | PGD1650887 | NA | Dcrc vs WT | -1.49929 | 2.04E-18 |
| PA14_00320 | hypothetical_unclassified | PGD1650889 | NA | Dcrc vs WT | -1.62747 | 9.70E-09 |
| PA14_00340 | transport_of_small_molecule | PGD1650891 | NA | Dcrc vs WT | -1.20869 | 0.023893 |
| PA14_00570 | cell_wall_LPS | PGD1650927 | NA | Dcrc vs WT | 1.321645 | 0.002954 |
| PA14_00700 | antibiotic_resistance_and_su | PGD1650951 | NA | Dcrc vs WT | -1.91399 | 7.66E-04 |
| PA14_00700 | biosynthesis_of_cofactors | PGD1650951 | NA | Dcrc vs WT | -1.91399 | 7.66E-04 |
| PA14_00850 | membrane_proteins | PGD1650979 | tagS1 | Dcrc vs WT | -1.55257 | 1.58E-06 |
| PA14_00850 | protein_secretion_export | PGD1650979 | tagS1 | Dcrc vs WT | -1.55257 | 1.58E-06 |
| PA14_00850 | transport_of_small_molecule | PGD1650979 | tagS1 | Dcrc vs WT | -1.55257 | 1.58E-06 |
| PA14_00890 | protein_secretion_export | PGD1650985 | pppA | Dcrc vs WT | -1.47183 | 5.69E-06 |
| PA14_00890 | putative_enzymes | PGD1650985 | pppA | Dcrc vs WT | -1.47183 | 5.69E-06 |
| PA14_00900 | protein_secretion_export | PGD1650987 | tagF1 | Dcrc vs WT | -1.4102 | 1.14E-04 |
| PA14_00970 | hypothetical_unclassified | PGD1650997 | NA | Dcrc vs WT | -1.10049 | 2.12E-05 |
| PA14_00980 | hypothetical_unclassified | PGD1650999 | fha1 | Dcrc vs WT | -1.05278 | 2.21E-07 |
| PA14_01010 | protein_secretion_export | PGD1651003 | hsiB1 | Dcrc vs WT | 1.089794 | 1.28E-04 |
| PA14_01120 | hypothetical_unclassified | PGD1651021 | tsi6 | Dcrc vs WT | 1.024676 | 0.02298 |
| PA14_01190 | fatty_acid_and_phospholipid | PGD1651035 | NA | Dcrc vs WT | -1.50642 | 5.06E-06 |
| PA14_01230 | hypothetical_unclassified | PGD1651041 | NA | Dcrc vs WT | -1.07458 | 6.16E-06 |
| PA14_01310 | central_intermediary_metab | PGD1651053 | NA | Dcrc vs WT | 1.215414 | 2.99E-06 |
| PA14_01310 | energy_metabolism_CAP | PGD1651053 | NA | Dcrc vs WT | 1.215414 | 2.99E-06 |
| PA14_01620 | amino_acid_biosynthesis_me | PGD1651103 | aptA | Dcrc vs WT | 1.088617 | 5.67E-05 |
| PA14_01620 | carbon_compound_catabolis | PGD1651103 | aptA | Dcrc vs WT | 1.088617 | 5.67E-05 |
| PA14_01670 | transport_of_small_molecule | PGD1651109 | NA | Dcrc vs WT | -1.18886 | 9.77E-05 |
| PA14_01710 | adaptation_and_protection | PGD1651115 | ahpC | Dcrc vs WT | 1.247387 | 2.35E-20 |
| PA14_02060 | membrane_proteins | PGD1651169 | NA | Dcrc vs WT | 1.631993 | 4.22E-05 |
| PA14_02100 | hypothetical_unclassified | PGD1651175 | NA | Dcrc vs WT | -1.09136 | 7.38E-05 |
| PA14_02180 | chemotaxis | PGD1651185 | cheB | Dcrc vs WT | -1.30395 | 3.59E-05 |
| PA14_02270 | chemotaxis | PGD1651199 | NA | Dcrc vs WT | -1.01604 | 1.30E-11 |
| PA14_02380 | fatty_acid_and_phospholipid | PGD1651215 | NA | Dcrc vs WT | 1.047744 | 0.009367 |
| PA14_02380 | putative_enzymes | PGD1651215 | NA | Dcrc vs WT | 1.047744 | 0.009367 |
| PA14_02435 | putative_enzymes | PGD1651223 | NA | Dcrc vs WT | -1.31916 | 0.02044 |
| PA14_02520 | hypothetical_unclassified | PGD1651237 | NA | Dcrc vs WT | -1.39847 | 6.15E-07 |
| PA14_02550 | carbon_compound_catabolis | PGD1651241 | mdcA | Dcrc vs WT | 1.113558 | 1.01E-04 |
| PA14_02730 | amino_acid_biosynthesis_me | PGD1651271 | NA | Dcrc vs WT | -1.21077 | 0.001084 |
| PA14_02890 | hypothetical_unclassified | PGD1651293 | NA | Dcrc vs WT | 1.029129 | 0.006746 |
| PA14_02960 | hypothetical_unclassified | PGD1651301 | NA | Dcrc vs WT | -1.41602 | 0.001106 |
| PA14_02970 | membrane_proteins | PGD1651303 | NA | Dcrc vs WT | 1.298345 | 2.84E-04 |
| PA14_03000 | amino_acid_biosynthesis_me | PGD1651309 | NA | Dcrc vs WT | -1.78938 | 1.38E-05 |
| PA14_03000 | putative_enzymes | PGD1651309 | NA | Dcrc vs WT | -1.78938 | 1.38E-05 |
| PA14_03110 | hypothetical_unclassified | PGD1651329 | NA | Dcrc vs WT | 1.639156 | 2.06E-05 |
| PA14_03450 | amino_acid_biosynthesis_me | PGD1651395 | gabT | Dcrc vs WT | 1.33226 | 3.00E-10 |
| PA14_03450 | carbon_compound_catabolis | PGD1651395 | gabT | Dcrc vs WT | 1.33226 | 3.00E-10 |
| PA14_03450 | central_intermediary_metab | PGD1651395 | gabT | Dcrc vs WT | 1.33226 | 3.00E-10 |
| PA14_03590 | membrane_proteins | PGD1651415 | NA | Dcrc vs WT | -1.23597 | 1.16E-04 |
| PA14_03610 | chaperones_heat_shock | PGD1651417 | NA | Dcrc vs WT | 1.318692 | 8.90E-05 |
| PA14_03630 | transcriptional_regulators | PGD1651421 | NA | Dcrc vs WT | -1.0732 | 0.009367 |

| | | | | | | |
|---|---|---|---|---|---|---|
| PA14_03670 | membrane_proteins | PGD1651425 | cysW | Dcrc vs WT | 1.28918 | 1.60E-05 |
| PA14_03670 | transport_of_small_molecule | PGD1651425 | cysW | Dcrc vs WT | 1.28918 | 1.60E-05 |
| PA14_03680 | membrane_proteins | PGD1651427 | cysT | Dcrc vs WT | 1.154154 | 0.001071 |
| PA14_03680 | transport_of_small_molecule | PGD1651427 | cysT | Dcrc vs WT | 1.154154 | 0.001071 |
| PA14_03700 | transport_of_small_molecule | PGD1651429 | sbp | Dcrc vs WT | 1.452448 | 3.53E-06 |
| PA14_03770 | amino_acid_biosynthesis_me | PGD1651439 | speB1 | Dcrc vs WT | 1.019069 | 0.002187 |
| PA14_03880 | amino_acid_biosynthesis_me | PGD1651459 | spuB | Dcrc vs WT | 1.256771 | 2.90E-16 |
| PA14_03880 | carbon_compound_catabolis | PGD1651459 | spuB | Dcrc vs WT | 1.256771 | 2.90E-16 |
| PA14_03880 | putative_enzymes | PGD1651459 | spuB | Dcrc vs WT | 1.256771 | 2.90E-16 |
| PA14_03900 | biosynthesis_of_cofactors | PGD1651461 | spuC | Dcrc vs WT | 1.423998 | 3.07E-28 |
| PA14_03920 | transport_of_small_molecule | PGD1651463 | spuD | Dcrc vs WT | 1.273969 | 2.82E-12 |
| PA14_03930 | transport_of_small_molecule | PGD1651465 | spuE | Dcrc vs WT | 1.132637 | 2.51E-08 |
| PA14_03940 | transport_of_small_molecule | PGD1651467 | spuF | Dcrc vs WT | 1.358906 | 6.53E-09 |
| PA14_03950 | membrane_proteins | PGD1651469 | spuG | Dcrc vs WT | 1.101321 | 1.23E-05 |
| PA14_03950 | transport_of_small_molecule | PGD1651469 | spuG | Dcrc vs WT | 1.101321 | 1.23E-05 |
| PA14_03960 | transport_of_small_molecule | PGD1651471 | spuH | Dcrc vs WT | 1.00671 | 4.87E-11 |
| PA14_04110 | amino_acid_biosynthesis_me | PGD1651497 | serA | Dcrc vs WT | 1.368818 | 7.60E-12 |
| PA14_04180 | hypothetical_unclassified | PGD1651505 | NA | Dcrc vs WT | 1.451494 | 0.001045 |
| PA14_04330 | membrane_proteins | PGD1651529 | NA | Dcrc vs WT | -1.01003 | 4.89E-05 |
| PA14_04340 | transport_of_small_molecule | PGD1651531 | NA | Dcrc vs WT | -1.09752 | 1.87E-05 |
| PA14_04520 | membrane_proteins | PGD1651557 | NA | Dcrc vs WT | -1.01889 | 3.26E-06 |
| PA14_04970 | biosynthesis_of_cofactors | PGD1651631 | NA | Dcrc vs WT | -1.34979 | 3.86E-05 |
| PA14_05080 | amino_acid_biosynthesis_me | PGD1651651 | metX | Dcrc vs WT | 1.055586 | 1.35E-04 |
| PA14_05510 | membrane_proteins | PGD1651719 | NA | Dcrc vs WT | 1.121161 | 8.82E-08 |
| PA14_05510 | secreted_factors | PGD1651719 | NA | Dcrc vs WT | 1.121161 | 8.82E-08 |
| PA14_05600 | hypothetical_unclassified | PGD1651735 | NA | Dcrc vs WT | 1.000198 | 1.62E-04 |
| PA14_05620 | amino_acid_biosynthesis_me | PGD1651737 | sahH | Dcrc vs WT | 1.318138 | 3.03E-13 |
| PA14_05620 | central_intermediary_metab | PGD1651737 | sahH | Dcrc vs WT | 1.318138 | 3.03E-13 |
| PA14_05770 | nucleotide_biosynthesis_and | PGD1651755 | dhT | Dcrc vs WT | -1.2616 | 1.49E-05 |
| PA14_05880 | membrane_proteins | PGD1651773 | NA | Dcrc vs WT | -1.31183 | 1.11E-05 |
| PA14_06570 | biosynthesis_of_cofactors | PGD1651873 | bioD | Dcrc vs WT | -1.06529 | 0.00319 |
| PA14_06650 | energy_metabolism_CAP | PGD1651883 | nirN | Dcrc vs WT | -1.34642 | 2.66E-08 |
| PA14_06660 | biosynthesis_of_cofactors | PGD1651885 | nirE | Dcrc vs WT | -1.67153 | 6.80E-05 |
| PA14_06660 | energy_metabolism_CAP | PGD1651885 | nirE | Dcrc vs WT | -1.67153 | 6.80E-05 |
| PA14_06740 | energy_metabolism_CAP | PGD1651901 | nirM | Dcrc vs WT | -1.68439 | 6.18E-09 |
| PA14_06770 | central_intermediary_metab | PGD1651905 | nirQ | Dcrc vs WT | -1.43831 | 2.92E-06 |
| PA14_06770 | energy_metabolism_CAP | PGD1651905 | nirQ | Dcrc vs WT | -1.43831 | 2.92E-06 |
| PA14_06790 | energy_metabolism_CAP | PGD1651907 | NA | Dcrc vs WT | -1.64391 | 7.39E-07 |
| PA14_06800 | hypothetical_unclassified | PGD1651909 | NA | Dcrc vs WT | -2.884 | 1.04E-08 |
| PA14_06810 | energy_metabolism_CAP | PGD1651911 | norC | Dcrc vs WT | -1.03469 | 2.23E-04 |
| PA14_06840 | energy_metabolism_CAP | PGD1651915 | NA | Dcrc vs WT | -1.77289 | 8.02E-11 |
| PA14_06860 | hypothetical_unclassified | PGD1651917 | NA | Dcrc vs WT | -1.51561 | 1.51E-07 |
| NA | NA | PGD1651922 | NA | Dcrc vs WT | -2.18963 | 3.48E-05 |
| PA14_07050 | biosynthesis_of_cofactors | PGD1651955 | NA | Dcrc vs WT | -1.07869 | 2.73E-08 |
| PA14_07090 | amino_acid_biosynthesis_me | PGD1651961 | metK | Dcrc vs WT | 1.431676 | 2.95E-15 |
| PA14_07090 | antibiotic_resistance_and_su | PGD1651961 | metK | Dcrc vs WT | 1.431676 | 2.95E-15 |
| PA14_07090 | central_intermediary_metab | PGD1651961 | metK | Dcrc vs WT | 1.431676 | 2.95E-15 |
| PA14_07430 | hypothetical_unclassified | PGD1652013 | NA | Dcrc vs WT | -2.14873 | 2.59E-15 |
| PA14_07550 | hypothetical_unclassified | PGD1652031 | NA | Dcrc vs WT | 1.713558 | 1.43E-13 |
| PA14_07970 | transcription_RNA_processin | PGD1652099 | NA | Dcrc vs WT | 1.1513 | 0.030863 |

| | | | | | | |
|---|---|---|---|---|---|---|
| PA14_07980 | hypothetical_unclassified | PGD1652101 | NA | Dcrc vs WT | 1.132456 | 0.00483 |
| PA14_08090 | relative_phage_transposon | PGD1652121 | NA | Dcrc vs WT | 1.291275 | 0.001721 |
| PA14_08210 | relative_phage_transposon | PGD1652143 | NA | Dcrc vs WT | 1.353584 | 6.04E-04 |
| PA14_08220 | hypothetical_unclassified | PGD1652145 | NA | Dcrc vs WT | 1.135449 | 0.016109 |
| PA14_08270 | hypothetical_unclassified | PGD1652155 | NA | Dcrc vs WT | 1.235602 | 0.047911 |
| PA14_08720 | translation_posttranslational | PGD1652237 | rplK | Dcrc vs WT | 1.346648 | 3.54E-24 |
| PA14_08730 | translation_posttranslational | PGD1652239 | rplA | Dcrc vs WT | 1.114863 | 9.31E-14 |
| PA14_08740 | translation_posttranslational | PGD1652241 | rplJ | Dcrc vs WT | 1.373409 | 3.12E-12 |
| PA14_08780 | transcription_RNA_processin | PGD1652247 | rpoC | Dcrc vs WT | 1.103839 | 1.08E-11 |
| PA14_08810 | translation_posttranslational | PGD1652251 | rpsG | Dcrc vs WT | 1.415636 | 1.81E-17 |
| PA14_08820 | transcription_RNA_processin | PGD1652253 | fusA1 | Dcrc vs WT | 1.213236 | 1.21E-10 |
| PA14_08830 | translation_posttranslational | PGD1652255 | tufA | Dcrc vs WT | 1.109813 | 8.33E-09 |
| PA14_08850 | translation_posttranslational | PGD1652259 | rplC | Dcrc vs WT | 1.125987 | 7.19E-11 |
| PA14_08860 | transcription_RNA_processin | PGD1652261 | rplD | Dcrc vs WT | 1.395677 | 1.97E-13 |
| PA14_08860 | translation_posttranslational | PGD1652261 | rplD | Dcrc vs WT | 1.395677 | 1.97E-13 |
| PA14_08870 | translation_posttranslational | PGD1652263 | rplW | Dcrc vs WT | 1.261857 | 1.88E-12 |
| PA14_08880 | translation_posttranslational | PGD1652265 | rplB | Dcrc vs WT | 1.270405 | 2.87E-21 |
| PA14_08930 | translation_posttranslational | PGD1652275 | rpmC | Dcrc vs WT | 1.581108 | 2.24E-17 |
| PA14_08940 | translation_posttranslational | PGD1652277 | rpsQ | Dcrc vs WT | 1.159659 | 5.38E-09 |
| PA14_08970 | translation_posttranslational | PGD1652283 | rplE | Dcrc vs WT | 1.292729 | 1.57E-13 |
| PA14_09000 | translation_posttranslational | PGD1652289 | rplF | Dcrc vs WT | 1.811786 | 2.81E-20 |
| PA14_09010 | translation_posttranslational | PGD1652291 | rplR | Dcrc vs WT | 1.434167 | 2.11E-13 |
| PA14_09020 | translation_posttranslational | PGD1652293 | rpsE | Dcrc vs WT | 1.193471 | 7.04E-11 |
| PA14_09030 | translation_posttranslational | PGD1652295 | rpmD | Dcrc vs WT | 1.384394 | 1.36E-13 |
| PA14_09040 | translation_posttranslational | PGD1652297 | rplO | Dcrc vs WT | 1.527029 | 2.87E-21 |
| PA14_09090 | translation_posttranslational | PGD1652305 | rpsK | Dcrc vs WT | 1.495405 | 8.59E-13 |
| PA14_09100 | translation_posttranslational | PGD1652307 | rpsD | Dcrc vs WT | 1.271482 | 5.80E-09 |
| PA14_09115 | transcription_RNA_processin | PGD1652309 | rpoA | Dcrc vs WT | 1.309867 | 1.20E-10 |
| PA14_09130 | translation_posttranslational | PGD1652311 | rplQ | Dcrc vs WT | 1.130059 | 1.10E-12 |
| PA14_09400 | putative_enzymes | PGD1652351 | phzS | Dcrc vs WT | 2.07891 | 2.23E-18 |
| PA14_09410 | secreted_factors | PGD1652353 | phzG1 | Dcrc vs WT | 2.175303 | 1.44E-07 |
| PA14_09460 | secreted_factors | PGD1652361 | phzC1 | Dcrc vs WT | 1.772963 | 1.63E-07 |
| PA14_09470 | secreted_factors | PGD1652363 | phzB1 | Dcrc vs WT | 2.21381 | 2.88E-09 |
| PA14_09480 | secreted_factors | PGD1652365 | phzA1 | Dcrc vs WT | 2.515461 | 3.04E-12 |
| PA14_09490 | putative_enzymes | PGD1652367 | phzM | Dcrc vs WT | 1.480543 | 3.42E-12 |
| PA14_09520 | transport_of_small_molecule | PGD1652371 | mexI | Dcrc vs WT | 1.128065 | 1.02E-06 |
| PA14_09870 | hypothetical_unclassified | PGD1652419 | eftM | Dcrc vs WT | 1.897147 | 3.57E-10 |
| PA14_10200 | membrane_proteins | PGD1652475 | NA | Dcrc vs WT | 1.456675 | 4.39E-05 |
| PA14_10230 | carbon_compound_catabolis | PGD1652481 | adh | Dcrc vs WT | 2.018615 | 8.61E-09 |
| PA14_10240 | carbon_compound_catabolis | PGD1652483 | NA | Dcrc vs WT | 2.225167 | 0.000249 |
| PA14_10250 | carbon_compound_catabolis | PGD1652485 | acoB | Dcrc vs WT | 3.14792 | 2.67E-10 |
| PA14_10260 | carbon_compound_catabolis | PGD1652487 | NA | Dcrc vs WT | 1.555788 | 3.15E-04 |
| PA14_10270 | carbon_compound_catabolis | PGD1652489 | NA | Dcrc vs WT | 3.237299 | 0.022112 |
| PA14_10280 | putative_enzymes | PGD1652491 | NA | Dcrc vs WT | 2.212654 | 0.002448 |
| PA14_10290 | transcriptional_regulators | PGD1652493 | acoR | Dcrc vs WT | 1.675634 | 1.21E-21 |
| PA14_10300 | protein_secretion_export | PGD1652495 | NA | Dcrc vs WT | 2.137385 | 5.40E-10 |
| PA14_10380 | hypothetical_unclassified | PGD1652509 | NA | Dcrc vs WT | -1.0298 | 5.29E-11 |
| PA14_10770 | two_component_regulators | PGD1652567 | NA | Dcrc vs WT | -1.02642 | 5.32E-11 |
| PA14_10850 | putative_enzymes | PGD1652581 | NA | Dcrc vs WT | 1.313414 | 3.53E-06 |
| PA14_10910 | transport_of_small_molecule | PGD1652589 | NA | Dcrc vs WT | -2.15871 | 1.55E-05 |

| | | | | | | |
|---|---|---|---|---|---|---|
| PA14_10990 | carbon_compound_catabolis | PGD1652603 | hpaC | Dcrc vs WT | 1.738043 | 2.03E-07 |
| PA14_11000 | carbon_compound_catabolis | PGD1652605 | hpaA | Dcrc vs WT | 2.341846 | 3.37E-36 |
| PA14_11050 | hypothetical_unclassified | PGD1652613 | NA | Dcrc vs WT | -2.5817 | 6.55E-04 |
| PA14_11170 | hypothetical_unclassified | PGD1652637 | NA | Dcrc vs WT | -1.09594 | 2.37E-10 |
| PA14_11400 | antibiotic_resistance_and_su | PGD1652673 | ribD | Dcrc vs WT | -1.01054 | 2.76E-08 |
| PA14_11400 | biosynthesis_of_cofactors | PGD1652673 | ribD | Dcrc vs WT | -1.01054 | 2.76E-08 |
| PA14_11690 | central_intermediary_metab | PGD1652723 | ppa | Dcrc vs WT | 1.201646 | 2.07E-21 |
| PA14_11690 | energy_metabolism_CAP | PGD1652723 | ppa | Dcrc vs WT | 1.201646 | 2.07E-21 |
| PA14_11790 | transport_of_small_molecule | PGD1652739 | NA | Dcrc vs WT | 1.061219 | 3.21E-04 |
| PA14_11810 | putative_enzymes | PGD1652741 | NA | Dcrc vs WT | 1.987117 | 4.37E-26 |
| PA14_11880 | hypothetical_unclassified | PGD1652749 | NA | Dcrc vs WT | -1.09739 | 1.43E-04 |
| PA14_11990 | membrane_proteins | PGD1652769 | NA | Dcrc vs WT | -1.40299 | 2.97E-05 |
| PA14_11990 | putative_enzymes | PGD1652769 | NA | Dcrc vs WT | -1.40299 | 2.97E-05 |
| PA14_12140 | transcriptional_regulators | PGD1652795 | NA | Dcrc vs WT | -1.08312 | 4.90E-10 |
| PA14_12400 | biosynthesis_of_cofactors | PGD1652831 | thiE | Dcrc vs WT | -1.14229 | 5.26E-05 |
| PA14_12440 | transcriptional_regulators | PGD1652837 | NA | Dcrc vs WT | -2.18125 | 1.81E-14 |
| PA14_12590 | hypothetical_unclassified | PGD1652855 | NA | Dcrc vs WT | -1.14266 | 1.78E-04 |
| PA14_12640 | hypothetical_unclassified | PGD1652863 | NA | Dcrc vs WT | 1.13196 | 0.028148 |
| PA14_12920 | transport_of_small_molecule | PGD1652907 | NA | Dcrc vs WT | 1.809492 | 2.74E-06 |
| PA14_12940 | transport_of_small_molecule | PGD1652909 | NA | Dcrc vs WT | 1.415639 | 0.001024 |
| PA14_12960 | transport_of_small_molecule | PGD1652911 | NA | Dcrc vs WT | 1.898857 | 2.76E-04 |
| PA14_12970 | carbon_compound_catabolis | PGD1652913 | tauD | Dcrc vs WT | 1.245034 | 0.00924 |
| PA14_13000 | transcriptional_regulators | PGD1652919 | NA | Dcrc vs WT | 1.199871 | 1.85E-05 |
| PA14_13010 | hypothetical_unclassified | PGD1652921 | NA | Dcrc vs WT | 1.697421 | 2.76E-08 |
| PA14_13050 | hypothetical_unclassified | PGD1652927 | NA | Dcrc vs WT | -1.89656 | 6.02E-10 |
| PA14_13230 | biosynthesis_of_cofactors | PGD1652953 | moaC | Dcrc vs WT | -1.16545 | 8.39E-13 |
| PA14_13240 | biosynthesis_of_cofactors | PGD1652955 | moaD | Dcrc vs WT | -1.22496 | 4.26E-05 |
| PA14_13250 | biosynthesis_of_cofactors | PGD1652957 | moaE | Dcrc vs WT | -2.1192 | 3.00E-20 |
| PA14_13260 | biosynthesis_of_cofactors | PGD1652959 | moaB1 | Dcrc vs WT | -1.44328 | 1.63E-07 |
| PA14_13280 | biosynthesis_of_cofactors | PGD1652961 | moeA1 | Dcrc vs WT | -2.17783 | 1.36E-11 |
| PA14_13290 | translation_posttranslational | PGD1652963 | NA | Dcrc vs WT | -1.76446 | 3.08E-11 |
| PA14_13300 | translation_posttranslational | PGD1652965 | NA | Dcrc vs WT | -1.64335 | 1.40E-08 |
| PA14_13320 | fatty_acid_and_phospholipid | PGD1652967 | NA | Dcrc vs WT | -1.72338 | 2.27E-08 |
| PA14_13330 | nucleotide_biosynthesis_and | PGD1652969 | NA | Dcrc vs WT | -1.10813 | 3.41E-06 |
| PA14_13330 | putative_enzymes | PGD1652969 | NA | Dcrc vs WT | -1.10813 | 3.41E-06 |
| PA14_13330 | secreted_factors | PGD1652969 | NA | Dcrc vs WT | -1.10813 | 3.41E-06 |
| PA14_13390 | hypothetical_unclassified | PGD1652981 | NA | Dcrc vs WT | -1.66862 | 5.11E-08 |
| PA14_13580 | transport_of_small_molecule | PGD1653007 | NA | Dcrc vs WT | 1.191158 | 6.14E-05 |
| PA14_13590 | membrane_proteins | PGD1653009 | NA | Dcrc vs WT | 1.491948 | 3.53E-07 |
| PA14_13590 | transport_of_small_molecule | PGD1653009 | NA | Dcrc vs WT | 1.491948 | 3.53E-07 |
| PA14_13720 | hypothetical_unclassified | PGD1653031 | NA | Dcrc vs WT | -1.21051 | 4.57E-04 |
| PA14_13840 | chaperones_heat_shock | PGD1653049 | NA | Dcrc vs WT | -1.58208 | 4.51E-06 |
| PA14_13840 | translation_posttranslational | PGD1653049 | NA | Dcrc vs WT | -1.58208 | 4.51E-06 |
| PA14_14060 | fatty_acid_and_phospholipid | PGD1653083 | NA | Dcrc vs WT | -1.52036 | 5.77E-14 |
| PA14_14060 | putative_enzymes | PGD1653083 | NA | Dcrc vs WT | -1.52036 | 5.77E-14 |
| PA14_14270 | putative_enzymes | PGD1653111 | NA | Dcrc vs WT | -1.08804 | 1.60E-08 |
| PA14_14330 | chaperones_heat_shock | PGD1653123 | NA | Dcrc vs WT | 1.257698 | 0.009367 |
| PA14_14330 | protein_secretion_export | PGD1653123 | NA | Dcrc vs WT | 1.257698 | 0.009367 |
| PA14_14330 | secreted_factors | PGD1653123 | NA | Dcrc vs WT | 1.257698 | 0.009367 |
| PA14_14560 | hypothetical_unclassified | PGD1653165 | NA | Dcrc vs WT | 1.173606 | 7.54E-06 |

| | | | | | | |
|---|---|---|---|---|---|---|
| PA14_14650 | protein_secretion_export | PGD1653177 | secF | Dcrc vs WT | 1.102405 | 3.88E-17 |
| PA14_15150 | transcriptional_regulators | PGD1653253 | NA | Dcrc vs WT | -1.13708 | 1.58E-08 |
| PA14_15180 | membrane_proteins | PGD1653257 | NA | Dcrc vs WT | 1.359372 | 6.38E-06 |
| PA14_15180 | transport_of_small_molecule | PGD1653257 | NA | Dcrc vs WT | 1.359372 | 6.38E-06 |
| PA14_15190 | membrane_proteins | PGD1653259 | NA | Dcrc vs WT | 1.135754 | 0.045512 |
| PA14_15190 | transport_of_small_molecule | PGD1653259 | NA | Dcrc vs WT | 1.135754 | 0.045512 |
| PA14_15200 | transport_of_small_molecule | PGD1653261 | NA | Dcrc vs WT | 1.163504 | 3.98E-05 |
| PA14_15445 | hypothetical_unclassified | PGD1653299 | merE | Dcrc vs WT | 1.153064 | 0.018507 |
| PA14_15680 | nucleotide_biosynthesis_and | PGD1653345 | NA | Dcrc vs WT | -1.04918 | 3.39E-10 |
| PA14_15710 | hypothetical_unclassified | PGD1653349 | NA | Dcrc vs WT | -1.71048 | 6.15E-05 |
| PA14_15850 | nucleotide_biosynthesis_and | PGD1653371 | NA | Dcrc vs WT | -1.12394 | 3.06E-06 |
| PA14_15970 | DNA_replication_recombinat | PGD1653391 | rpsP | Dcrc vs WT | 1.023137 | 7.78E-13 |
| PA14_15970 | translation_posttranslational | PGD1653391 | rpsP | Dcrc vs WT | 1.023137 | 7.78E-13 |
| PA14_15980 | transcription_RNA_processin | PGD1653393 | rimM | Dcrc vs WT | 1.47358 | 4.12E-26 |
| PA14_15990 | transcription_RNA_processin | PGD1653395 | trmD | Dcrc vs WT | 1.398359 | 3.02E-22 |
| PA14_16000 | transcription_RNA_processin | PGD1653397 | rplS | Dcrc vs WT | 1.093392 | 8.13E-14 |
| PA14_16000 | translation_posttranslational | PGD1653397 | rplS | Dcrc vs WT | 1.093392 | 8.13E-14 |
| PA14_16010 | fatty_acid_and_phospholipid | PGD1653399 | NA | Dcrc vs WT | 2.103645 | 1.41E-05 |
| PA14_16410 | transport_of_small_molecule | PGD1653465 | NA | Dcrc vs WT | 1.060042 | 1.08E-04 |
| PA14_16630 | membrane_proteins | PGD1653499 | NA | Dcrc vs WT | 1.522364 | 1.63E-08 |
| PA14_16630 | transport_of_small_molecule | PGD1653499 | NA | Dcrc vs WT | 1.522364 | 1.63E-08 |
| PA14_16700 | nucleotide_biosynthesis_and | PGD1653511 | adk | Dcrc vs WT | 1.188244 | 2.04E-18 |
| PA14_16720 | hypothetical_unclassified | PGD1653515 | pemA | Dcrc vs WT | -1.13625 | 1.79E-06 |
| PA14_16950 | amino_acid_biosynthesis_me | PGD1653551 | dapD | Dcrc vs WT | 1.12942 | 4.04E-06 |
| PA14_16950 | cell_wall_LPS | PGD1653551 | dapD | Dcrc vs WT | 1.12942 | 4.04E-06 |
| PA14_17070 | translation_posttranslational | PGD1653573 | tsf | Dcrc vs WT | 1.023547 | 1.74E-10 |
| PA14_17640 | transport_of_small_molecule | PGD1653669 | potA | Dcrc vs WT | 1.227168 | 0.048205 |
| PA14_17710 | translation_posttranslational | PGD1653683 | rpmJ | Dcrc vs WT | 1.137848 | 1.16E-05 |
| PA14_17880 | carbon_compound_catabolis | PGD1653705 | NA | Dcrc vs WT | -2.20306 | 0.009131 |
| PA14_17880 | fatty_acid_and_phospholipid | PGD1653705 | NA | Dcrc vs WT | -2.20306 | 0.009131 |
| PA14_17900 | transcriptional_regulators | PGD1653709 | metR | Dcrc vs WT | 1.014642 | 6.66E-08 |
| PA14_17960 | central_intermediary_metab | PGD1653719 | glpK | Dcrc vs WT | 1.358202 | 1.96E-08 |
| PA14_18120 | amino_acid_biosynthesis_me | PGD1653745 | mmsA | Dcrc vs WT | 1.405071 | 5.61E-29 |
| PA14_18150 | fatty_acid_and_phospholipid | PGD1653749 | NA | Dcrc vs WT | 2.276753 | 1.17E-41 |
| PA14_18210 | DNA_replication_recombinat | PGD1653757 | NA | Dcrc vs WT | -1.47575 | 4.71E-04 |
| PA14_18590 | hypothetical_unclassified | PGD1653807 | NA | Dcrc vs WT | 1.450667 | 5.13E-06 |
| PA14_18620 | hypothetical_unclassified | PGD1653813 | NA | Dcrc vs WT | -1.00628 | 6.84E-07 |
| PA14_18740 | amino_acid_biosynthesis_me | PGD1653835 | argG | Dcrc vs WT | 1.02958 | 2.40E-11 |
| PA14_18810 | hypothetical_unclassified | PGD1653847 | NA | Dcrc vs WT | 1.31281 | 1.84E-06 |
| PA14_18820 | hypothetical_unclassified | PGD1653849 | NA | Dcrc vs WT | 1.930415 | 1.45E-05 |
| PA14_18850 | carbon_compound_catabolis | PGD1653853 | NA | Dcrc vs WT | 1.620837 | 1.21E-04 |
| PA14_18850 | putative_enzymes | PGD1653853 | NA | Dcrc vs WT | 1.620837 | 1.21E-04 |
| PA14_18870 | hypothetical_unclassified | PGD1653857 | NA | Dcrc vs WT | -1.39027 | 1.03E-06 |
| PA14_19110 | secreted_factors | PGD1653893 | rhlB | Dcrc vs WT | -1.42649 | 5.26E-10 |
| PA14_19270 | transport_of_small_molecule | PGD1653915 | NA | Dcrc vs WT | -1.41617 | 3.70E-04 |
| PA14_19310 | transport_of_small_molecule | PGD1653919 | NA | Dcrc vs WT | -1.14124 | 5.79E-12 |
| PA14_19490 | adaptation_and_protection | PGD1653949 | lsfA | Dcrc vs WT | 1.415562 | 2.93E-05 |
| PA14_19500 | transport_of_small_molecule | PGD1653951 | NA | Dcrc vs WT | 1.994592 | 5.92E-06 |
| PA14_19510 | transport_of_small_molecule | PGD1653953 | NA | Dcrc vs WT | 1.216214 | 0.014322 |
| PA14_19520 | transport_of_small_molecule | PGD1653955 | NA | Dcrc vs WT | 2.092403 | 1.63E-04 |

| | | | | | | |
|---|---|---|---|---|---|---|
| PA14_19530 | central_intermediary_metab | PGD1653957 | NA | Dcrc vs WT | 1.170149 | 0.002256 |
| PA14_19540 | transport_of_small_molecule | PGD1653959 | NA | Dcrc vs WT | 2.282354 | 3.65E-09 |
| PA14_19560 | central_intermediary_metab | PGD1653961 | ssuD | Dcrc vs WT | 1.501614 | 0.006411 |
| PA14_19570 | transport_of_small_molecule | PGD1653963 | NA | Dcrc vs WT | 2.280594 | 5.21E-05 |
| PA14_19580 | transport_of_small_molecule | PGD1653965 | ssuB | Dcrc vs WT | 1.726259 | 0.018773 |
| PA14_19590 | central_intermediary_metab | PGD1653967 | NA | Dcrc vs WT | 1.946092 | 0.002049 |
| PA14_19650 | membrane_proteins | PGD1653979 | NA | Dcrc vs WT | -2.06153 | 0.025549 |
| PA14_19690 | membrane_proteins | PGD1653987 | NA | Dcrc vs WT | 1.552399 | 0.018052 |
| PA14_19690 | protein_secretion_export | PGD1653987 | NA | Dcrc vs WT | 1.552399 | 0.018052 |
| PA14_19850 | transcriptional_regulators | PGD1654009 | NA | Dcrc vs WT | -1.02233 | 0.004681 |
| PA14_19970 | hypothetical_unclassified | PGD1654029 | NA | Dcrc vs WT | 1.121634 | 0.019336 |
| PA14_19990 | transcriptional_regulators | PGD1654031 | NA | Dcrc vs WT | 1.242019 | 0.040056 |
| PA14_20010 | transport_of_small_molecule | PGD1654035 | hasR | Dcrc vs WT | 2.772174 | 2.21E-06 |
| PA14_20020 | transport_of_small_molecule | PGD1654037 | hasAp | Dcrc vs WT | 4.398077 | 7.04E-11 |
| PA14_20030 | protein_secretion_export | PGD1654039 | hasD | Dcrc vs WT | 3.373954 | 8.15E-07 |
| PA14_20040 | protein_secretion_export | PGD1654041 | hasE | Dcrc vs WT | 2.801031 | 8.62E-05 |
| PA14_20050 | protein_secretion_export | PGD1654043 | NA | Dcrc vs WT | 1.948926 | 2.65E-04 |
| PA14_20150 | energy_metabolism_CAP | PGD1654061 | nosL | Dcrc vs WT | -1.31806 | 2.37E-04 |
| PA14_20180 | energy_metabolism_CAP | PGD1654065 | nosF | Dcrc vs WT | -1.12213 | 0.003356 |
| PA14_20180 | transport_of_small_molecule | PGD1654065 | nosF | Dcrc vs WT | -1.12213 | 0.003356 |
| PA14_20240 | putative_enzymes | PGD1654073 | NA | Dcrc vs WT | -1.1412 | 0.003343 |
| PA14_20330 | transport_of_small_molecule | PGD1654089 | phnE | Dcrc vs WT | -1.58126 | 0.046621 |
| PA14_20430 | transport_of_small_molecule | PGD1654105 | phnM | Dcrc vs WT | -1.75331 | 1.77E-05 |
| PA14_21080 | transcriptional_regulators | PGD1654219 | NA | Dcrc vs WT | -1.04087 | 0.007037 |
| PA14_21240 | hypothetical_unclassified | PGD1654247 | NA | Dcrc vs WT | -1.2891 | 2.57E-07 |
| PA14_21260 | hypothetical_unclassified | PGD1654251 | NA | Dcrc vs WT | -1.8288 | 1.32E-07 |
| PA14_21570 | hypothetical_unclassified | PGD1654297 | NA | Dcrc vs WT | 1.690354 | 0.008944 |
| PA14_21580 | hypothetical_unclassified | PGD1654299 | NA | Dcrc vs WT | 2.02903 | 0.003659 |
| PA14_21590 | hypothetical_unclassified | PGD1654301 | NA | Dcrc vs WT | 2.175951 | 0.003177 |
| PA14_21600 | membrane_proteins | PGD1654303 | NA | Dcrc vs WT | 2.515217 | 4.68E-04 |
| PA14_22000 | transcription_RNA_processin | PGD1654379 | rluA | Dcrc vs WT | 1.09765 | 0.003878 |
| PA14_22060 | fatty_acid_and_phospholipid | PGD1654389 | NA | Dcrc vs WT | -1.14101 | 2.76E-08 |
| PA14_22240 | hypothetical_unclassified | PGD1654419 | NA | Dcrc vs WT | 2.234651 | 0.017992 |
| PA14_22320 | membrane_proteins | PGD1654433 | NA | Dcrc vs WT | 1.982757 | 6.01E-07 |
| PA14_22340 | membrane_proteins | PGD1654437 | NA | Dcrc vs WT | 1.050612 | 8.26E-05 |
| PA14_22350 | membrane_proteins | PGD1654439 | actP | Dcrc vs WT | 2.023425 | 2.52E-16 |
| PA14_22590 | hypothetical_unclassified | PGD1654481 | NA | Dcrc vs WT | -1.68458 | 1.06E-05 |
| PA14_22640 | transcriptional_regulators | PGD1654489 | NA | Dcrc vs WT | -1.22024 | 1.65E-15 |
| PA14_22650 | transport_of_small_molecule | PGD1654491 | NA | Dcrc vs WT | -1.27447 | 2.54E-08 |
| PA14_22880 | hypothetical_unclassified | PGD1654529 | NA | Dcrc vs WT | -1.42132 | 0.002657 |
| PA14_22890 | carbon_compound_catabolis | PGD1654531 | gapA | Dcrc vs WT | 1.135452 | 6.66E-08 |
| PA14_22890 | energy_metabolism_CAP | PGD1654531 | gapA | Dcrc vs WT | 1.135452 | 6.66E-08 |
| PA14_22990 | transport_of_small_molecule | PGD1654543 | NA | Dcrc vs WT | 1.906081 | 1.45E-21 |
| PA14_23000 | transport_of_small_molecule | PGD1654545 | NA | Dcrc vs WT | 2.215413 | 5.04E-17 |
| PA14_23010 | transport_of_small_molecule | PGD1654547 | gltK | Dcrc vs WT | 2.436072 | 9.66E-33 |
| PA14_23030 | transport_of_small_molecule | PGD1654549 | oprB | Dcrc vs WT | 2.787396 | 5.33E-65 |
| PA14_23420 | cell_wall_LPS | PGD1654615 | zbdP | Dcrc vs WT | 1.014696 | 1.12E-06 |
| PA14_23680 | chaperones_heat_shock | PGD1654661 | ibpA | Dcrc vs WT | 1.687683 | 4.42E-05 |
| PA14_23690 | protein_secretion_export | PGD1654663 | NA | Dcrc vs WT | -1.05836 | 2.31E-05 |
| PA14_23980 | protein_secretion_export | PGD1654705 | xcpP | Dcrc vs WT | -1.14765 | 2.13E-05 |

| | | | | | | |
|---|---|---|---|---|---|---|
| PA14_24020 | protein_secretion_export | PGD1654711 | xcpT | Dcrc vs WT | 1.059152 | 6.80E-08 |
| PA14_24100 | protein_secretion_export | PGD1654723 | xcpZ | Dcrc vs WT | -1.34292 | 6.58E-05 |
| PA14_24210 | hypothetical_unclassified | PGD1654741 | NA | Dcrc vs WT | -1.26244 | 7.00E-11 |
| PA14_24490 | cell_wall_LPS | PGD1654789 | pelB | Dcrc vs WT | -1.02006 | 1.07E-08 |
| PA14_24500 | cell_wall_LPS | PGD1654791 | pelC | Dcrc vs WT | -1.11655 | 0.002184 |
| PA14_24500 | fatty_acid_and_phospholipid | PGD1654791 | pelC | Dcrc vs WT | -1.11655 | 0.002184 |
| PA14_24630 | hypothetical_unclassified | PGD1654813 | NA | Dcrc vs WT | -1.00103 | 6.44E-06 |
| PA14_24650 | translation_posttranslational | PGD1654817 | rmf | Dcrc vs WT | -1.29685 | 1.84E-07 |
| PA14_24760 | membrane_proteins | PGD1654835 | NA | Dcrc vs WT | 1.040942 | 3.78E-06 |
| PA14_24790 | transport_of_small_molecule | PGD1654841 | NA | Dcrc vs WT | 1.127861 | 8.90E-09 |
| NA | NA | PGD1654856 | NA | Dcrc vs WT | 1.374817 | 2.98E-06 |
| PA14_24950 | carbon_compound_catabolis | PGD1654869 | NA | Dcrc vs WT | -1.01339 | 0.001997 |
| PA14_24950 | energy_metabolism_CAP | PGD1654869 | NA | Dcrc vs WT | -1.01339 | 0.001997 |
| PA14_26000 | biosynthesis_of_cofactors | PGD1655039 | NA | Dcrc vs WT | -1.17663 | 0.001264 |
| PA14_26200 | hypothetical_unclassified | PGD1655069 | NA | Dcrc vs WT | -1.38307 | 0.004634 |
| PA14_26240 | transport_of_small_molecule | PGD1655077 | hisJ | Dcrc vs WT | -2.2972 | 0.036229 |
| PA14_26280 | chemotaxis | PGD1655083 | NA | Dcrc vs WT | -1.10715 | 2.07E-09 |
| PA14_26460 | biosynthesis_of_cofactors | PGD1655105 | cobK | Dcrc vs WT | -1.10352 | 0.008967 |
| PA14_26470 | biosynthesis_of_cofactors | PGD1655107 | cbiD | Dcrc vs WT | -1.07599 | 2.97E-06 |
| PA14_26530 | biosynthesis_of_cofactors | PGD1655117 | cobJ | Dcrc vs WT | -1.3117 | 1.11E-04 |
| PA14_26940 | hypothetical_unclassified | PGD1655179 | NA | Dcrc vs WT | -1.00492 | 0.001718 |
| PA14_27090 | protein_secretion_export | PGD1655197 | lipH | Dcrc vs WT | -1.45155 | 0.034097 |
| PA14_27090 | secreted_factors | PGD1655197 | lipH | Dcrc vs WT | -1.45155 | 0.034097 |
| PA14_27370 | transcription_RNA_processin | PGD1655243 | NA | Dcrc vs WT | 1.658148 | 3.47E-10 |
| PA14_27400 | transcriptional_regulators | PGD1655247 | NA | Dcrc vs WT | -1.74195 | 3.15E-04 |
| PA14_27450 | adaptation_and_protection | PGD1655257 | NA | Dcrc vs WT | -1.13629 | 0.006388 |
| PA14_27720 | hypothetical_unclassified | PGD1655309 | NA | Dcrc vs WT | -1.26668 | 5.09E-10 |
| PA14_27980 | translation_posttranslational | PGD1655351 | NA | Dcrc vs WT | 1.091802 | 1.78E-08 |
| PA14_28140 | hypothetical_unclassified | PGD1655383 | NA | Dcrc vs WT | 1.037939 | 5.16E-08 |
| PA14_28260 | hypothetical_unclassified | PGD1655405 | NA | Dcrc vs WT | -1.17323 | 5.92E-06 |
| PA14_28370 | membrane_proteins | PGD1655425 | NA | Dcrc vs WT | 1.342699 | 4.43E-07 |
| PA14_28390 | membrane_proteins | PGD1655429 | NA | Dcrc vs WT | 1.146783 | 0.007831 |
| PA14_28500 | hypothetical_unclassified | PGD1655449 | NA | Dcrc vs WT | -1.02829 | 0.001369 |
| PA14_28520 | hypothetical_unclassified | PGD1655453 | NA | Dcrc vs WT | -1.43247 | 1.71E-08 |
| PA14_28610 | hypothetical_unclassified | PGD1655469 | NA | Dcrc vs WT | -1.77204 | 5.66E-09 |
| PA14_28620 | membrane_proteins | PGD1655471 | NA | Dcrc vs WT | -1.26984 | 5.55E-11 |
| PA14_28670 | translation_posttranslational | PGD1655479 | rpmI | Dcrc vs WT | 1.290557 | 6.33E-09 |
| PA14_28680 | translation_posttranslational | PGD1655481 | rplT | Dcrc vs WT | 1.071944 | 2.84E-09 |
| PA14_28980 | membrane_proteins | PGD1655535 | NA | Dcrc vs WT | -1.43956 | 0.00647 |
| PA14_28980 | transcriptional_regulators | PGD1655535 | NA | Dcrc vs WT | -1.43956 | 0.00647 |
| PA14_29210 | transport_of_small_molecule | PGD1655575 | NA | Dcrc vs WT | -1.16435 | 5.55E-04 |
| PA14_29320 | putative_enzymes | PGD1655595 | NA | Dcrc vs WT | -1.11931 | 0.00394 |
| PA14_29440 | transcriptional_regulators | PGD1655614 | NA | Dcrc vs WT | -1.22617 | 3.05E-04 |
| PA14_29510 | protein_secretion_export | PGD1655626 | NA | Dcrc vs WT | -1.15083 | 0.020895 |
| PA14_29590 | transcriptional_regulators | PGD1655642 | NA | Dcrc vs WT | 1.07503 | 2.90E-07 |
| PA14_29600 | biosynthesis_of_cofactors | PGD1655644 | NA | Dcrc vs WT | 1.002572 | 2.46E-05 |
| PA14_29750 | hypothetical_unclassified | PGD1655666 | NA | Dcrc vs WT | -1.35048 | 1.40E-04 |
| PA14_29920 | energy_metabolism_CAP | PGD1655688 | nuoI | Dcrc vs WT | 1.029681 | 0.001084 |
| PA14_30180 | energy_metabolism_CAP | PGD1655728 | idh | Dcrc vs WT | 1.029396 | 5.94E-09 |
| PA14_30400 | energy_metabolism_CAP | PGD1655766 | NA | Dcrc vs WT | -1.27767 | 1.83E-06 |

| | | | | | | |
|---|---|---|---|---|---|---|
| NA | NA | PGD1655813 | NA | Dcrc vs WT | -1.09546 | 0.012411 |
| PA14_30750 | amino_acid_biosynthesis_me | PGD1655826 | NA | Dcrc vs WT | 1.040469 | 5.16E-11 |
| PA14_30750 | putative_enzymes | PGD1655826 | NA | Dcrc vs WT | 1.040469 | 5.16E-11 |
| PA14_30800 | energy_metabolism_CAP | PGD1655834 | NA | Dcrc vs WT | 1.292982 | 1.27E-05 |
| PA14_31190 | hypothetical_unclassified | PGD1655904 | NA | Dcrc vs WT | -1.34047 | 0.032481 |
| PA14_31450 | hypothetical_unclassified | PGD1655950 | NA | Dcrc vs WT | -1.47415 | 1.74E-09 |
| PA14_31540 | energy_metabolism_CAP | PGD1655964 | NA | Dcrc vs WT | 1.014241 | 5.03E-06 |
| PA14_31780 | transcriptional_regulators | PGD1656000 | NA | Dcrc vs WT | -1.36487 | 4.21E-06 |
| PA14_31990 | transport_of_small_molecule | PGD1656028 | czcB | Dcrc vs WT | -1.56482 | 0.024254 |
| PA14_32150 | biosynthesis_of_cofactors | PGD1656044 | antB | Dcrc vs WT | 1.85077 | 0.002249 |
| PA14_32220 | carbon_compound_catabolis | PGD1656052 | catB | Dcrc vs WT | 1.259516 | 0.003443 |
| PA14_32230 | carbon_compound_catabolis | PGD1656054 | catC | Dcrc vs WT | 1.291452 | 1.44E-04 |
| PA14_32310 | membrane_proteins | PGD1656068 | NA | Dcrc vs WT | -1.03448 | 2.17E-05 |
| PA14_32330 | transport_of_small_molecule | PGD1656070 | NA | Dcrc vs WT | -1.26738 | 3.42E-07 |
| PA14_32350 | hypothetical_unclassified | PGD1656074 | NA | Dcrc vs WT | -1.80101 | 0.013251 |
| PA14_32390 | antibiotic_resistance_and_su | PGD1656082 | mexF | Dcrc vs WT | 1.116226 | 0.001831 |
| PA14_32570 | two_component_regulators | PGD1656110 | NA | Dcrc vs WT | -1.17035 | 4.92E-05 |
| PA14_32810 | hypothetical_unclassified | PGD1656148 | NA | Dcrc vs WT | -1.01196 | 0.010954 |
| PA14_32830 | hypothetical_unclassified | PGD1656152 | NA | Dcrc vs WT | -1.08003 | 8.94E-05 |
| PA14_33130 | membrane_proteins | PGD1656196 | NA | Dcrc vs WT | -1.24885 | 3.73E-08 |
| PA14_33130 | transport_of_small_molecule | PGD1656196 | NA | Dcrc vs WT | -1.24885 | 3.73E-08 |
| PA14_33920 | transcriptional_regulators | PGD1656328 | NA | Dcrc vs WT | -1.73481 | 1.83E-06 |
| PA14_33960 | hypothetical_unclassified | PGD1656334 | vgrG3 | Dcrc vs WT | -1.45442 | 5.58E-17 |
| PA14_33980 | hypothetical_unclassified | PGD1656338 | NA | Dcrc vs WT | -1.05841 | 3.65E-04 |
| PA14_33990 | translation_posttranslational | PGD1656340 | clpV3 | Dcrc vs WT | -2.02974 | 3.56E-23 |
| PA14_34000 | hypothetical_unclassified | PGD1656342 | hsiH3 | Dcrc vs WT | -1.86836 | 2.04E-08 |
| PA14_34010 | membrane_proteins | PGD1656344 | hsiG3 | Dcrc vs WT | -2.01016 | 3.31E-14 |
| PA14_34020 | hypothetical_unclassified | PGD1656346 | hsiF3 | Dcrc vs WT | -2.11941 | 6.12E-09 |
| PA14_34030 | hypothetical_unclassified | PGD1656348 | hcp3 | Dcrc vs WT | -1.09617 | 6.79E-05 |
| PA14_34080 | hypothetical_unclassified | PGD1656354 | lip3 | Dcrc vs WT | -1.11347 | 1.21E-06 |
| PA14_34110 | hypothetical_unclassified | PGD1656358 | dotU3 | Dcrc vs WT | -1.02124 | 1.91E-08 |
| PA14_34130 | hypothetical_unclassified | PGD1656360 | icmF3 | Dcrc vs WT | -1.42226 | 8.27E-18 |
| PA14_34320 | putative_enzymes | PGD1656390 | NA | Dcrc vs WT | -1.15265 | 0.026327 |
| PA14_34360 | carbon_compound_catabolis | PGD1656398 | mtlD | Dcrc vs WT | 1.367535 | 0.002433 |
| PA14_34370 | transport_of_small_molecule | PGD1656400 | NA | Dcrc vs WT | 1.9894 | 2.22E-08 |
| PA14_34390 | transport_of_small_molecule | PGD1656402 | NA | Dcrc vs WT | 1.151727 | 0.009237 |
| PA14_34420 | transport_of_small_molecule | PGD1656406 | NA | Dcrc vs WT | 1.804066 | 1.41E-12 |
| PA14_34670 | putative_enzymes | PGD1656438 | NA | Dcrc vs WT | 1.668877 | 9.49E-16 |
| PA14_34690 | transcriptional_regulators | PGD1656442 | NA | Dcrc vs WT | -1.21316 | 0.001985 |
| PA14_34710 | transport_of_small_molecule | PGD1656446 | NA | Dcrc vs WT | -1.77178 | 0.025373 |
| PA14_34730 | transcriptional_regulators | PGD1656450 | NA | Dcrc vs WT | 2.087925 | 3.10E-06 |
| PA14_34750 | central_intermediary_metab | PGD1656454 | NA | Dcrc vs WT | 1.18124 | 6.73E-04 |
| PA14_34770 | transport_of_small_molecule | PGD1656456 | NA | Dcrc vs WT | 2.258863 | 2.03E-05 |
| PA14_34810 | fatty_acid_and_phospholipid | PGD1656464 | NA | Dcrc vs WT | -1.69779 | 2.04E-18 |
| PA14_34810 | putative_enzymes | PGD1656464 | NA | Dcrc vs WT | -1.69779 | 2.04E-18 |
| PA14_34810 | secreted_factors | PGD1656464 | NA | Dcrc vs WT | -1.69779 | 2.04E-18 |
| PA14_34820 | transcriptional_regulators | PGD1656466 | NA | Dcrc vs WT | -1.18807 | 9.46E-10 |
| PA14_34840 | putative_enzymes | PGD1656470 | NA | Dcrc vs WT | -1.22164 | 5.71E-10 |
| PA14_34840 | secreted_factors | PGD1656470 | NA | Dcrc vs WT | -1.22164 | 5.71E-10 |
| PA14_34850 | translation_posttranslational | PGD1656472 | NA | Dcrc vs WT | -1.02449 | 1.10E-07 |

| | | | | | | |
|---|---|---|---|---|---|---|
| PA14_34870 | carbon_compound_catabolis | PGD1656474 | chiC | Dcrc vs WT | -1.43219 | 0.006793 |
| PA14_35080 | adaptation_and_protection | PGD1656508 | NA | Dcrc vs WT | 1.094179 | 0.041312 |
| PA14_35170 | transcriptional_regulators | PGD1656522 | NA | Dcrc vs WT | -1.45103 | 3.26E-06 |
| PA14_35230 | membrane_proteins | PGD1656530 | NA | Dcrc vs WT | -1.03102 | 0.01636 |
| PA14_35230 | transport_of_small_molecule | PGD1656530 | NA | Dcrc vs WT | -1.03102 | 0.01636 |
| PA14_35240 | hypothetical_unclassified | PGD1656532 | NA | Dcrc vs WT | -1.45664 | 0.003583 |
| PA14_35390 | amino_acid_biosynthesis_me | PGD1656554 | pvcD | Dcrc vs WT | -2.15429 | 0.001927 |
| PA14_35390 | secreted_factors | PGD1656554 | pvcD | Dcrc vs WT | -2.15429 | 0.001927 |
| PA14_35490 | amino_acid_biosynthesis_me | PGD1656568 | lpdV | Dcrc vs WT | 2.001305 | 5.93E-36 |
| PA14_35490 | energy_metabolism_CAP | PGD1656568 | lpdV | Dcrc vs WT | 2.001305 | 5.93E-36 |
| PA14_35500 | amino_acid_biosynthesis_me | PGD1656570 | bkdB | Dcrc vs WT | 2.597091 | 3.31E-82 |
| PA14_35520 | amino_acid_biosynthesis_me | PGD1656572 | bkdA2 | Dcrc vs WT | 3.035367 | 1.22E-84 |
| PA14_35530 | amino_acid_biosynthesis_me | PGD1656574 | bkdA1 | Dcrc vs WT | 2.737287 | 2.59E-66 |
| PA14_35680 | hypothetical_unclassified | PGD1656596 | pslF | Dcrc vs WT | -2.36231 | 2.39E-06 |
| PA14_36080 | transport_of_small_molecule | PGD1656668 | NA | Dcrc vs WT | -1.93997 | 0.030064 |
| PA14_36090 | membrane_proteins | PGD1656670 | NA | Dcrc vs WT | -1.07402 | 0.011972 |
| PA14_36100 | biosynthesis_of_cofactors | PGD1656672 | pdxA | Dcrc vs WT | 2.085635 | 5.24E-07 |
| PA14_36200 | transport_of_small_molecule | PGD1656688 | NA | Dcrc vs WT | 2.348917 | 5.08E-12 |
| PA14_36220 | membrane_proteins | PGD1656690 | NA | Dcrc vs WT | 1.638785 | 9.47E-04 |
| PA14_36220 | transport_of_small_molecule | PGD1656690 | NA | Dcrc vs WT | 1.638785 | 9.47E-04 |
| PA14_36280 | antibiotic_resistance_and_su | PGD1656700 | NA | Dcrc vs WT | -1.67656 | 0.028257 |
| PA14_36330 | central_intermediary_metab | PGD1656710 | hcnA | Dcrc vs WT | -1.27994 | 4.50E-10 |
| PA14_36400 | hypothetical_unclassified | PGD1656724 | NA | Dcrc vs WT | -1.04653 | 0.025428 |
| PA14_36480 | hypothetical_unclassified | PGD1656736 | NA | Dcrc vs WT | 1.559971 | 2.12E-05 |
| PA14_36520 | hypothetical_unclassified | PGD1656742 | NA | Dcrc vs WT | 1.210343 | 1.88E-09 |
| PA14_36530 | hypothetical_unclassified | PGD1656744 | NA | Dcrc vs WT | 1.116886 | 5.84E-07 |
| PA14_36605 | putative_enzymes | PGD1656758 | NA | Dcrc vs WT | 2.131622 | 7.59E-09 |
| PA14_36620 | hypothetical_unclassified | PGD1656760 | NA | Dcrc vs WT | 4.638301 | 0.001084 |
| PA14_36630 | carbon_compound_catabolis | PGD1656762 | NA | Dcrc vs WT | 3.664055 | 2.11E-38 |
| PA14_36630 | putative_enzymes | PGD1656762 | NA | Dcrc vs WT | 3.664055 | 2.11E-38 |
| PA14_36670 | hypothetical_unclassified | PGD1656768 | NA | Dcrc vs WT | 1.813085 | 5.49E-07 |
| PA14_36680 | putative_enzymes | PGD1656770 | NA | Dcrc vs WT | 1.443938 | 2.51E-04 |
| PA14_36690 | fatty_acid_and_phospholipid | PGD1656772 | NA | Dcrc vs WT | 1.119394 | 0.001767 |
| PA14_36690 | putative_enzymes | PGD1656772 | NA | Dcrc vs WT | 1.119394 | 0.001767 |
| PA14_36700 | membrane_proteins | PGD1656774 | NA | Dcrc vs WT | 1.467196 | 7.57E-05 |
| PA14_36710 | energy_metabolism_CAP | PGD1656776 | glgB | Dcrc vs WT | 2.954993 | 1.17E-11 |
| PA14_36730 | carbon_compound_catabolis | PGD1656778 | NA | Dcrc vs WT | 1.259902 | 4.71E-06 |
| PA14_36730 | putative_enzymes | PGD1656778 | NA | Dcrc vs WT | 1.259902 | 4.71E-06 |
| PA14_36810 | adaptation_and_protection | PGD1656790 | katE | Dcrc vs WT | 1.69068 | 4.01E-09 |
| PA14_36860 | hypothetical_unclassified | PGD1656800 | NA | Dcrc vs WT | -1.02276 | 3.98E-07 |
| PA14_36990 | two_component_regulators | PGD1656822 | NA | Dcrc vs WT | -1.38283 | 0.027201 |
| PA14_37090 | energy_metabolism_CAP | PGD1656838 | NA | Dcrc vs WT | -1.54939 | 0.001763 |
| PA14_37090 | putative_enzymes | PGD1656838 | NA | Dcrc vs WT | -1.54939 | 0.001763 |
| PA14_37170 | hypothetical_unclassified | PGD1656850 | NA | Dcrc vs WT | -1.00486 | 1.91E-04 |
| PA14_37250 | transport_of_small_molecule | PGD1656860 | NA | Dcrc vs WT | 1.579795 | 7.59E-13 |
| PA14_37260 | transport_of_small_molecule | PGD1656862 | NA | Dcrc vs WT | 1.31842 | 5.14E-09 |
| PA14_37270 | putative_enzymes | PGD1656864 | NA | Dcrc vs WT | 1.586171 | 9.99E-08 |
| PA14_37290 | transport_of_small_molecule | PGD1656866 | NA | Dcrc vs WT | 1.55031 | 1.88E-07 |
| PA14_37310 | transport_of_small_molecule | PGD1656868 | NA | Dcrc vs WT | 2.103819 | 7.55E-18 |
| PA14_37320 | membrane_proteins | PGD1656870 | NA | Dcrc vs WT | 1.725441 | 6.35E-10 |

| | | | | | | |
|---|---|---|---|---|---|---|
| PA14_37370 | fatty_acid_and_phospholipid | PGD1656878 | NA | Dcrc vs WT | -1.45511 | 0.01289 |
| PA14_37730 | transport_of_small_molecule | PGD1656932 | NA | Dcrc vs WT | -1.01821 | 2.65E-07 |
| PA14_37840 | transport_of_small_molecule | PGD1656950 | sppD | Dcrc vs WT | -1.58419 | 1.79E-06 |
| PA14_37915 | transport_of_small_molecule | PGD1656962 | NA | Dcrc vs WT | -1.72943 | 1.79E-04 |
| PA14_37940 | transcriptional_regulators | PGD1656964 | cynR | Dcrc vs WT | -1.04011 | 0.007317 |
| PA14_37990 | transcriptional_regulators | PGD1656972 | NA | Dcrc vs WT | 1.263341 | 0.016822 |
| PA14_38050 | hypothetical_unclassified | PGD1656982 | NA | Dcrc vs WT | -1.67322 | 5.57E-07 |
| PA14_38110 | transport_of_small_molecule | PGD1656990 | NA | Dcrc vs WT | 1.107099 | 4.15E-08 |
| PA14_38130 | transport_of_small_molecule | PGD1656992 | NA | Dcrc vs WT | 1.124167 | 6.73E-07 |
| PA14_38160 | transport_of_small_molecule | PGD1656996 | NA | Dcrc vs WT | 1.247594 | 4.70E-05 |
| PA14_38170 | membrane_proteins | PGD1656998 | NA | Dcrc vs WT | 1.11001 | 3.51E-04 |
| PA14_38220 | transport_of_small_molecule | PGD1657008 | NA | Dcrc vs WT | 1.128631 | 0.002718 |
| PA14_38370 | hypothetical_unclassified | PGD1657032 | NA | Dcrc vs WT | -1.42073 | 3.08E-07 |
| PA14_38440 | carbon_compound_catabolis | PGD1657044 | gnyD | Dcrc vs WT | 1.100421 | 5.40E-21 |
| PA14_38440 | fatty_acid_and_phospholipid | PGD1657044 | gnyD | Dcrc vs WT | 1.100421 | 5.40E-21 |
| PA14_38730 | transport_of_small_molecule | PGD1657088 | NA | Dcrc vs WT | 1.32442 | 1.32E-05 |
| PA14_38770 | amino_acid_biosynthesis_me | PGD1657094 | NA | Dcrc vs WT | -1.32822 | 3.51E-07 |
| PA14_38825 | biosynthesis_of_cofactors | PGD1657104 | pqqA | Dcrc vs WT | -1.10571 | 4.26E-04 |
| PA14_38860 | carbon_compound_catabolis | PGD1657110 | exaA | Dcrc vs WT | -1.00727 | 0.009368 |
| PA14_38970 | two_component_regulators | PGD1657124 | NA | Dcrc vs WT | -1.10788 | 0.005135 |
| PA14_39080 | hypothetical_unclassified | PGD1657140 | NA | Dcrc vs WT | -1.43238 | 6.99E-04 |
| PA14_39210 | biosynthesis_of_cofactors | PGD1657162 | NA | Dcrc vs WT | -1.46291 | 1.60E-04 |
| PA14_39240 | hypothetical_unclassified | PGD1657168 | fapC | Dcrc vs WT | -1.51876 | 0.008941 |
| PA14_39320 | transport_of_small_molecule | PGD1657180 | rbsC | Dcrc vs WT | 1.093238 | 0.001036 |
| PA14_39420 | hypothetical_unclassified | PGD1657192 | NA | Dcrc vs WT | 1.757324 | 1.01E-05 |
| PA14_39590 | amino_acid_biosynthesis_me | PGD1657216 | metE | Dcrc vs WT | 2.56092 | 4.92E-10 |
| PA14_39640 | biosynthesis_of_cofactors | PGD1657224 | cobN | Dcrc vs WT | -1.60702 | 9.55E-04 |
| PA14_39700 | hypothetical_unclassified | PGD1657234 | NA | Dcrc vs WT | -1.59712 | 0.002007 |
| PA14_39790 | hypothetical_unclassified | PGD1657248 | NA | Dcrc vs WT | 1.249639 | 0.002866 |
| PA14_39990 | fatty_acid_and_phospholipid | PGD1657280 | NA | Dcrc vs WT | 1.182351 | 5.61E-06 |
| PA14_40020 | hypothetical_unclassified | PGD1657284 | NA | Dcrc vs WT | 1.020851 | 1.37E-06 |
| PA14_40030 | putative_enzymes | PGD1657286 | NA | Dcrc vs WT | 1.422653 | 3.00E-10 |
| PA14_40130 | transport_of_small_molecule | PGD1657304 | NA | Dcrc vs WT | -2.11947 | 2.56E-12 |
| PA14_40160 | energy_metabolism_CAP | PGD1657308 | NA | Dcrc vs WT | -1.32246 | 5.15E-05 |
| PA14_40520 | hypothetical_unclassified | PGD1657368 | NA | Dcrc vs WT | -2.26743 | 0.019105 |
| PA14_40650 | hypothetical_unclassified | PGD1657388 | NA | Dcrc vs WT | 1.466703 | 1.20E-06 |
| PA14_40750 | hypothetical_unclassified | PGD1657404 | NA | Dcrc vs WT | -1.40492 | 1.06E-08 |
| PA14_40860 | transport_of_small_molecule | PGD1657422 | NA | Dcrc vs WT | -1.59062 | 2.49E-10 |
| PA14_41240 | antibiotic_resistance_and_su | PGD1657482 | clpP | Dcrc vs WT | 1.011808 | 2.52E-14 |
| PA14_41240 | cell_wall_LPS | PGD1657482 | clpP | Dcrc vs WT | 1.011808 | 2.52E-14 |
| PA14_41240 | chaperones_heat_shock | PGD1657482 | clpP | Dcrc vs WT | 1.011808 | 2.52E-14 |
| PA14_41240 | motility_and_attachment | PGD1657482 | clpP | Dcrc vs WT | 1.011808 | 2.52E-14 |
| PA14_41520 | adaptation_and_protection | PGD1657530 | NA | Dcrc vs WT | -1.10966 | 0.016398 |
| PA14_41650 | fatty_acid_and_phospholipid | PGD1657552 | NA | Dcrc vs WT | 1.166429 | 1.89E-04 |
| PA14_41790 | hypothetical_unclassified | PGD1657572 | NA | Dcrc vs WT | 1.060389 | 6.98E-08 |
| PA14_42030 | hypothetical_unclassified | PGD1657614 | NA | Dcrc vs WT | -1.45375 | 0.00226 |
| PA14_42050 | putative_enzymes | PGD1657616 | NA | Dcrc vs WT | -1.43473 | 0.004003 |
| PA14_42080 | fatty_acid_and_phospholipid | PGD1657620 | NA | Dcrc vs WT | 1.307863 | 2.22E-06 |
| PA14_42080 | putative_enzymes | PGD1657620 | NA | Dcrc vs WT | 1.307863 | 2.22E-06 |
| PA14_42090 | fatty_acid_and_phospholipid | PGD1657622 | NA | Dcrc vs WT | 1.014109 | 0.002606 |

| | | | | | | |
|---|---|---|---|---|---|---|
| PA14_42550 | membrane_proteins | PGD1657698 | popN | Dcrc vs WT | -1.60222 | 0.012625 |
| PA14_42550 | protein_secretion_export | PGD1657698 | popN | Dcrc vs WT | -1.60222 | 0.012625 |
| PA14_42710 | hypothetical_unclassified | PGD1657724 | NA | Dcrc vs WT | -1.01393 | 1.39E-05 |
| PA14_42830 | membrane_proteins | PGD1657744 | NA | Dcrc vs WT | -1.06806 | 1.69E-05 |
| PA14_43080 | hypothetical_unclassified | PGD1657788 | vgrG14 | Dcrc vs WT | 1.627696 | 0.017149 |
| PA14_43180 | putative_enzymes | PGD1657806 | NA | Dcrc vs WT | -1.45521 | 7.07E-08 |
| PA14_43230 | hypothetical_unclassified | PGD1657814 | NA | Dcrc vs WT | -1.50887 | 4.39E-06 |
| PA14_43340 | transcriptional_regulators | PGD1657832 | kdpE | Dcrc vs WT | -1.02642 | 1.71E-05 |
| PA14_43340 | two_component_regulators | PGD1657832 | kdpE | Dcrc vs WT | -1.02642 | 1.71E-05 |
| PA14_43350 | two_component_regulators | PGD1657834 | kdpD | Dcrc vs WT | -1.07255 | 1.92E-10 |
| PA14_43420 | fatty_acid_and_phospholipid | PGD1657844 | NA | Dcrc vs WT | -1.34866 | 8.59E-13 |
| PA14_43420 | putative_enzymes | PGD1657844 | NA | Dcrc vs WT | -1.34866 | 8.59E-13 |
| PA14_43460 | fatty_acid_and_phospholipid | PGD1657850 | NA | Dcrc vs WT | -1.14576 | 0.042024 |
| PA14_43490 | transport_of_small_molecule | PGD1657854 | NA | Dcrc vs WT | -1.36383 | 4.26E-04 |
| PA14_43950 | energy_metabolism_CAP | PGD1657930 | sucC | Dcrc vs WT | 1.100353 | 4.99E-12 |
| PA14_44030 | energy_metabolism_CAP | PGD1657940 | sdhA | Dcrc vs WT | 1.024576 | 2.88E-16 |
| PA14_44050 | energy_metabolism_CAP | PGD1657942 | sdhD | Dcrc vs WT | 1.631149 | 8.11E-16 |
| PA14_44100 | hypothetical_unclassified | PGD1657952 | NA | Dcrc vs WT | -1.14322 | 1.60E-06 |
| PA14_44210 | amino_acid_biosynthesis_me | PGD1657974 | NA | Dcrc vs WT | -1.42575 | 5.26E-10 |
| PA14_44230 | hypothetical_unclassified | PGD1657976 | NA | Dcrc vs WT | 1.174428 | 0.028211 |
| PA14_44390 | central_intermediary_metab | PGD1658004 | NA | Dcrc vs WT | 1.085963 | 5.92E-06 |
| PA14_44390 | energy_metabolism_CAP | PGD1658004 | NA | Dcrc vs WT | 1.085963 | 5.92E-06 |
| PA14_44480 | hypothetical_unclassified | PGD1658020 | pemB | Dcrc vs WT | -1.02538 | 3.74E-06 |
| PA14_44890 | secreted_factors | PGD1658086 | hcpA | Dcrc vs WT | 1.328334 | 1.16E-04 |
| PA14_45030 | carbon_compound_catabolis | PGD1658112 | NA | Dcrc vs WT | -1.39057 | 0.025602 |
| PA14_45030 | putative_enzymes | PGD1658112 | NA | Dcrc vs WT | -1.39057 | 0.025602 |
| PA14_45050 | carbon_compound_catabolis | PGD1658114 | pykF | Dcrc vs WT | -1.01258 | 0.019714 |
| PA14_45050 | energy_metabolism_CAP | PGD1658114 | pykF | Dcrc vs WT | -1.01258 | 0.019714 |
| PA14_45090 | hypothetical_unclassified | PGD1658120 | NA | Dcrc vs WT | -1.28883 | 1.76E-05 |
| PA14_45180 | putative_enzymes | PGD1658134 | NA | Dcrc vs WT | -1.22176 | 0.047203 |
| PA14_45480 | hypothetical_unclassified | PGD1658178 | NA | Dcrc vs WT | -1.03637 | 7.42E-05 |
| PA14_45920 | membrane_proteins | PGD1658240 | NA | Dcrc vs WT | -1.07338 | 2.07E-07 |
| PA14_45950 | adaptation_and_protection | PGD1658246 | rsaL | Dcrc vs WT | -1.17495 | 5.52E-04 |
| PA14_45950 | transcriptional_regulators | PGD1658246 | rsaL | Dcrc vs WT | -1.17495 | 5.52E-04 |
| PA14_46110 | transport_of_small_molecule | PGD1658270 | NA | Dcrc vs WT | 1.091374 | 0.001344 |
| PA14_46320 | energy_metabolism_CAP | PGD1658306 | NA | Dcrc vs WT | -1.01021 | 9.55E-05 |
| PA14_46420 | putative_enzymes | PGD1658322 | NA | Dcrc vs WT | -1.60379 | 2.69E-07 |
| PA14_46530 | hypothetical_unclassified | PGD1658342 | NA | Dcrc vs WT | -1.05919 | 7.18E-05 |
| PA14_46810 | transcriptional_regulators | PGD1658392 | NA | Dcrc vs WT | -1.1981 | 1.27E-05 |
| PA14_46890 | putative_enzymes | PGD1658406 | NA | Dcrc vs WT | -1.1232 | 5.05E-07 |
| PA14_47080 | transcriptional_regulators | PGD1658438 | NA | Dcrc vs WT | -1.34197 | 1.89E-06 |
| PA14_47120 | hypothetical_unclassified | PGD1658446 | NA | Dcrc vs WT | 1.536237 | 6.75E-12 |
| PA14_47210 | energy_metabolism_CAP | PGD1658460 | cyoA | Dcrc vs WT | -1.23763 | 0.00843 |
| PA14_47510 | putative_enzymes | PGD1658512 | NA | Dcrc vs WT | -1.16192 | 0.001655 |
| PA14_47530 | hypothetical_unclassified | PGD1658516 | NA | Dcrc vs WT | -1.13299 | 7.23E-05 |
| PA14_47560 | membrane_proteins | PGD1658522 | NA | Dcrc vs WT | -1.14447 | 0.04166 |
| PA14_47560 | transport_of_small_molecule | PGD1658522 | NA | Dcrc vs WT | -1.14447 | 0.04166 |
| PA14_47670 | biosynthesis_of_cofactors | PGD1658536 | cobT | Dcrc vs WT | -1.33656 | 2.02E-04 |
| PA14_47730 | biosynthesis_of_cofactors | PGD1658544 | cobD | Dcrc vs WT | -1.21139 | 9.18E-05 |
| PA14_47860 | putative_enzymes | PGD1658562 | NA | Dcrc vs WT | -2.18496 | 1.02E-07 |

| | | | | | | |
|---|---|---|---|---|---|---|
| PA14_47880 | transcriptional_regulators | PGD1658566 | NA | Dcrc vs WT | -1.00833 | 0.001057 |
| PA14_47970 | central_intermediary_metab | PGD1658584 | NA | Dcrc vs WT | -1.14111 | 0.020536 |
| PA14_48420 | transcriptional_regulators | PGD1658644 | NA | Dcrc vs WT | -1.11197 | 2.63E-07 |
| PA14_48440 | putative_enzymes | PGD1658646 | NA | Dcrc vs WT | -1.53834 | 2.26E-04 |
| PA14_48460 | transport_of_small_molecule | PGD1658650 | NA | Dcrc vs WT | -1.56524 | 3.05E-04 |
| PA14_48530 | putative_enzymes | PGD1658662 | NA | Dcrc vs WT | -1.30075 | 0.002599 |
| PA14_48640 | hypothetical_unclassified | PGD1658682 | NA | Dcrc vs WT | -1.48009 | 1.13E-04 |
| PA14_49130 | transport_of_small_molecule | PGD1658770 | dctA | Dcrc vs WT | -1.06299 | 0.017254 |
| PA14_49220 | energy_metabolism_CAP | PGD1658784 | napF | Dcrc vs WT | -1.32625 | 5.76E-05 |
| PA14_49300 | putative_enzymes | PGD1658798 | NA | Dcrc vs WT | -1.02767 | 1.26E-05 |
| PA14_49310 | hypothetical_unclassified | PGD1658800 | NA | Dcrc vs WT | -1.28526 | 6.35E-08 |
| PA14_49320 | hypothetical_unclassified | PGD1658802 | NA | Dcrc vs WT | -1.06735 | 4.76E-19 |
| PA14_49340 | translation_posttranslational | PGD1658806 | pcpS | Dcrc vs WT | -1.48281 | 3.18E-06 |
| PA14_49400 | hypothetical_unclassified | PGD1658816 | NA | Dcrc vs WT | -1.0488 | 1.02E-07 |
| PA14_49720 | hypothetical_unclassified | PGD1658868 | NA | Dcrc vs WT | 1.116753 | 0.009097 |
| PA14_49820 | biosynthesis_of_cofactors | PGD1658886 | NA | Dcrc vs WT | -1.20962 | 3.78E-06 |
| PA14_49860 | hypothetical_unclassified | PGD1658892 | NA | Dcrc vs WT | -1.08486 | 5.23E-07 |
| PA14_50000 | hypothetical_unclassified | PGD1658916 | NA | Dcrc vs WT | 1.625667 | 1.19E-16 |
| PA14_50550 | transport_of_small_molecule | PGD1659000 | livG | Dcrc vs WT | 1.093399 | 6.81E-05 |
| PA14_50610 | putative_enzymes | PGD1659010 | NA | Dcrc vs WT | -1.01244 | 1.44E-04 |
| PA14_50890 | hypothetical_unclassified | PGD1659064 | NA | Dcrc vs WT | -1.18023 | 6.71E-07 |
| PA14_51300 | hypothetical_unclassified | PGD1659134 | NA | Dcrc vs WT | 1.047451 | 1.90E-11 |
| PA14_51310 | hypothetical_unclassified | PGD1659136 | NA | Dcrc vs WT | 1.421691 | 5.68E-14 |
| PA14_51410 | biosynthesis_of_cofactors | PGD1659152 | pqsC | Dcrc vs WT | 1.062635 | 8.90E-12 |
| PA14_51640 | hypothetical_unclassified | PGD1659198 | NA | Dcrc vs WT | -1.14175 | 0.025406 |
| PA14_51710 | membrane_proteins | PGD1659210 | oprL | Dcrc vs WT | 1.221014 | 3.91E-19 |
| PA14_51710 | transport_of_small_molecule | PGD1659210 | oprL | Dcrc vs WT | 1.221014 | 3.91E-19 |
| PA14_51830 | adaptation_and_protection | PGD1659232 | NA | Dcrc vs WT | 1.018988 | 2.39E-09 |
| PA14_51840 | transcriptional_regulators | PGD1659234 | NA | Dcrc vs WT | 1.054103 | 6.28E-06 |
| PA14_51950 | hypothetical_unclassified | PGD1659254 | NA | Dcrc vs WT | 1.170096 | 1.21E-05 |
| PA14_52340 | membrane_proteins | PGD1659320 | NA | Dcrc vs WT | 1.45067 | 0.011385 |
| PA14_52490 | hypothetical_unclassified | PGD1659342 | NA | Dcrc vs WT | 1.861157 | 0.015418 |
| PA14_52570 | adaptation_and_protection | PGD1659358 | rsmA | Dcrc vs WT | -1.24047 | 1.59E-06 |
| PA14_52570 | transcriptional_regulators | PGD1659358 | rsmA | Dcrc vs WT | -1.24047 | 1.59E-06 |
| PA14_52570 | translation_posttranslational | PGD1659358 | rsmA | Dcrc vs WT | -1.24047 | 1.59E-06 |
| PA14_52610 | putative_enzymes | PGD1659364 | NA | Dcrc vs WT | -1.11304 | 3.57E-10 |
| PA14_52880 | putative_enzymes | PGD1659406 | NA | Dcrc vs WT | 3.24114 | 0.037173 |
| PA14_52910 | hypothetical_unclassified | PGD1659412 | NA | Dcrc vs WT | -1.21383 | 0.046671 |
| PA14_52960 | hypothetical_unclassified | PGD1659420 | NA | Dcrc vs WT | 2.059207 | 2.92E-06 |
| PA14_53000 | amino_acid_biosynthesis_me | PGD1659426 | phhB | Dcrc vs WT | 1.075424 | 1.61E-09 |
| PA14_53050 | transport_of_small_molecule | PGD1659436 | aroP2 | Dcrc vs WT | 1.537926 | 3.73E-09 |
| PA14_53290 | nucleotide_biosynthesis_and | PGD1659472 | trxB2 | Dcrc vs WT | -2.30924 | 1.56E-19 |
| PA14_53530 | hypothetical_unclassified | PGD1659512 | NA | Dcrc vs WT | 1.608277 | 1.16E-07 |
| PA14_53550 | transcriptional_regulators | PGD1659516 | NA | Dcrc vs WT | -1.61525 | 2.37E-08 |
| PA14_53650 | hypothetical_unclassified | PGD1659536 | NA | Dcrc vs WT | -1.35951 | 0.01524 |
| PA14_53700 | putative_enzymes | PGD1659546 | NA | Dcrc vs WT | 1.32551 | 0.004347 |
| PA14_53720 | transcriptional_regulators | PGD1659548 | NA | Dcrc vs WT | -1.32204 | 1.32E-05 |
| PA14_53740 | hypothetical_unclassified | PGD1659552 | NA | Dcrc vs WT | -1.04862 | 0.023943 |
| PA14_53750 | hypothetical_unclassified | PGD1659554 | NA | Dcrc vs WT | -1.57857 | 0.001097 |
| PA14_53940 | carbon_compound_catabolis | PGD1659588 | prpB | Dcrc vs WT | 1.213423 | 5.86E-05 |

| | | | | | | |
|---|---|---|---|---|---|---|
| PA14_53940 | central_intermediary_metab | PGD1659588 | prpB | Dcrc vs WT | 1.213423 | 5.86E-05 |
| PA14_53940 | fatty_acid_and_phospholipid | PGD1659588 | prpB | Dcrc vs WT | 1.213423 | 5.86E-05 |
| PA14_53950 | carbon_compound_catabolis | PGD1659590 | prpC | Dcrc vs WT | 1.278918 | 8.33E-09 |
| PA14_53950 | central_intermediary_metab | PGD1659590 | prpC | Dcrc vs WT | 1.278918 | 8.33E-09 |
| PA14_54010 | transcriptional_regulators | PGD1659598 | NA | Dcrc vs WT | -1.11128 | 9.03E-05 |
| PA14_54040 | membrane_proteins | PGD1659602 | NA | Dcrc vs WT | 1.031558 | 2.28E-05 |
| PA14_54050 | hypothetical_unclassified | PGD1659604 | NA | Dcrc vs WT | -1.72483 | 5.46E-09 |
| PA14_54220 | hypothetical_unclassified | PGD1659628 | icp | Dcrc vs WT | 1.005744 | 4.14E-04 |
| PA14_54550 | membrane_proteins | PGD1659680 | NA | Dcrc vs WT | 2.218882 | 0.035035 |
| PA14_54610 | transcriptional_regulators | PGD1659690 | NA | Dcrc vs WT | -1.60329 | 0.003932 |
| PA14_54710 | transcriptional_regulators | PGD1659708 | NA | Dcrc vs WT | -1.11839 | 0.001102 |
| PA14_55240 | hypothetical_unclassified | PGD1659804 | NA | Dcrc vs WT | -1.4779 | 1.41E-07 |
| PA14_55250 | transcriptional_regulators | PGD1659806 | NA | Dcrc vs WT | -1.33879 | 0.004931 |
| PA14_55360 | membrane_proteins | PGD1659822 | exbB2 | Dcrc vs WT | -1.37979 | 0.002301 |
| PA14_55360 | transport_of_small_molecule | PGD1659822 | exbB2 | Dcrc vs WT | -1.37979 | 0.002301 |
| PA14_55510 | hypothetical_unclassified | PGD1659848 | hxcP | Dcrc vs WT | -1.12885 | 0.001699 |
| PA14_55530 | protein_secretion_export | PGD1659852 | hxcW | Dcrc vs WT | -1.31605 | 1.91E-08 |
| PA14_55560 | transcriptional_regulators | PGD1659858 | NA | Dcrc vs WT | -1.07967 | 0.00621 |
| PA14_55580 | biosynthesis_of_cofactors | PGD1659862 | nemO | Dcrc vs WT | 1.041273 | 0.002342 |
| PA14_55590 | hypothetical_unclassified | PGD1659864 | NA | Dcrc vs WT | -1.51044 | 0.007418 |
| PA14_55600 | hypothetical_unclassified | PGD1659866 | NA | Dcrc vs WT | -1.23546 | 8.53E-05 |
| PA14_55730 | transcriptional_regulators | PGD1659894 | NA | Dcrc vs WT | 1.365728 | 0.002114 |
| PA14_55770 | membrane_proteins | PGD1659902 | NA | Dcrc vs WT | 1.06294 | 2.73E-17 |
| PA14_55770 | transport_of_small_molecule | PGD1659902 | NA | Dcrc vs WT | 1.06294 | 2.73E-17 |
| PA14_55880 | membrane_proteins | PGD1659920 | NA | Dcrc vs WT | -1.05393 | 2.39E-05 |
| PA14_55880 | motility_and_attachment | PGD1659920 | NA | Dcrc vs WT | -1.05393 | 2.39E-05 |
| PA14_55880 | protein_secretion_export | PGD1659920 | NA | Dcrc vs WT | -1.05393 | 2.39E-05 |
| PA14_55930 | motility_and_attachment | PGD1659928 | NA | Dcrc vs WT | -1.02676 | 8.22E-11 |
| PA14_55930 | protein_secretion_export | PGD1659928 | NA | Dcrc vs WT | -1.02676 | 8.22E-11 |
| PA14_55940 | protein_secretion_export | PGD1659930 | NA | Dcrc vs WT | -2.20099 | 1.93E-11 |
| PA14_56090 | membrane_proteins | PGD1659952 | NA | Dcrc vs WT | 1.042383 | 2.31E-08 |
| PA14_56260 | energy_metabolism_CAP | PGD1659980 | NA | Dcrc vs WT | -1.28856 | 8.66E-13 |
| PA14_56410 | fatty_acid_and_phospholipid | PGD1659996 | NA | Dcrc vs WT | -1.17064 | 1.08E-08 |
| PA14_56470 | transport_of_small_molecule | PGD1660004 | NA | Dcrc vs WT | -1.29048 | 3.62E-04 |
| PA14_56520 | hypothetical_unclassified | PGD1660010 | NA | Dcrc vs WT | -1.05149 | 5.09E-04 |
| PA14_56570 | fatty_acid_and_phospholipid | PGD1660020 | NA | Dcrc vs WT | -1.24533 | 0.016753 |
| PA14_56590 | adaptation_and_protection | PGD1660022 | NA | Dcrc vs WT | -1.02699 | 4.48E-04 |
| PA14_56660 | adaptation_and_protection | PGD1660030 | NA | Dcrc vs WT | 1.535185 | 3.81E-06 |
| PA14_56670 | hypothetical_unclassified | PGD1660032 | NA | Dcrc vs WT | -2.2782 | 7.60E-12 |
| PA14_56690 | transport_of_small_molecule | PGD1660036 | feoA | Dcrc vs WT | -1.08093 | 2.51E-04 |
| PA14_56740 | DNA_replication_recombinat | PGD1660044 | iciA | Dcrc vs WT | -1.11041 | 9.75E-05 |
| PA14_56910 | hypothetical_unclassified | PGD1660072 | NA | Dcrc vs WT | -1.63919 | 7.00E-11 |
| PA14_57010 | chaperones_heat_shock | PGD1660090 | groEL | Dcrc vs WT | 1.135221 | 4.92E-13 |
| PA14_57720 | amino_acid_biosynthesis_me | PGD1660205 | cysD | Dcrc vs WT | 1.162141 | 1.61E-05 |
| PA14_57720 | central_intermediary_metab | PGD1660205 | cysD | Dcrc vs WT | 1.162141 | 1.61E-05 |
| PA14_57990 | transport_of_small_molecule | PGD1660253 | NA | Dcrc vs WT | 1.762851 | 3.83E-04 |
| PA14_58000 | adaptation_and_protection | PGD1660255 | sodM | Dcrc vs WT | 2.338535 | 8.15E-07 |
| PA14_58010 | hypothetical_unclassified | PGD1660257 | NA | Dcrc vs WT | 2.396232 | 1.87E-05 |
| PA14_58030 | energy_metabolism_CAP | PGD1660259 | fumC | Dcrc vs WT | 2.018544 | 2.63E-07 |
| PA14_58040 | hypothetical_unclassified | PGD1660261 | NA | Dcrc vs WT | 2.097893 | 2.80E-05 |

| | | | | | | |
|---|---|---|---|---|---|---|
| PA14_58080 | putative_enzymes | PGD1660269 | NA | Dcrc vs WT | -1.71708 | 4.78E-17 |
| PA14_58380 | transcriptional_regulators | PGD1660317 | psdR | Dcrc vs WT | 1.022112 | 1.79E-06 |
| PA14_58410 | membrane_proteins | PGD1660321 | opdP | Dcrc vs WT | 1.812088 | 1.33E-19 |
| PA14_58410 | transport_of_small_molecule | PGD1660321 | opdP | Dcrc vs WT | 1.812088 | 1.33E-19 |
| PA14_58420 | transport_of_small_molecule | PGD1660323 | dppA4 | Dcrc vs WT | 2.089588 | 1.57E-23 |
| PA14_58440 | membrane_proteins | PGD1660325 | dppB | Dcrc vs WT | 2.168316 | 1.15E-29 |
| PA14_58440 | transport_of_small_molecule | PGD1660325 | dppB | Dcrc vs WT | 2.168316 | 1.15E-29 |
| PA14_58450 | membrane_proteins | PGD1660327 | dppC | Dcrc vs WT | 2.085378 | 6.72E-12 |
| PA14_58450 | transport_of_small_molecule | PGD1660327 | dppC | Dcrc vs WT | 2.085378 | 6.72E-12 |
| PA14_58470 | transport_of_small_molecule | PGD1660329 | dppD | Dcrc vs WT | 1.655553 | 1.15E-09 |
| PA14_58490 | transport_of_small_molecule | PGD1660331 | dppF | Dcrc vs WT | 1.613173 | 1.81E-14 |
| PA14_58580 | membrane_proteins | PGD1660349 | NA | Dcrc vs WT | 1.181685 | 9.18E-04 |
| PA14_58790 | hypothetical_unclassified | PGD1660385 | NA | Dcrc vs WT | 1.304236 | 4.62E-04 |
| PA14_58920 | hypothetical_unclassified | PGD1660411 | NA | Dcrc vs WT | -1.36765 | 0.016253 |
| PA14_59390 | hypothetical_unclassified | PGD1660495 | NA | Dcrc vs WT | -1.09008 | 7.58E-04 |
| PA14_59845 | hypothetical_unclassified | PGD1660576 | NA | Dcrc vs WT | -1.11315 | 4.53E-05 |
| PA14_60460 | translation_posttranslational | PGD1660692 | rplU | Dcrc vs WT | 1.208546 | 4.26E-13 |
| PA14_60560 | hypothetical_unclassified | PGD1660710 | NA | Dcrc vs WT | -1.39054 | 7.25E-06 |
| PA14_60750 | carbon_compound_catabolis | PGD1660738 | pra | Dcrc vs WT | 1.419987 | 3.72E-05 |
| PA14_61000 | hypothetical_unclassified | PGD1660778 | NA | Dcrc vs WT | -1.07988 | 0.0043 |
| PA14_61010 | hypothetical_unclassified | PGD1660780 | NA | Dcrc vs WT | -1.56514 | 2.39E-09 |
| PA14_61020 | adaptation_and_protection | PGD1660782 | NA | Dcrc vs WT | -1.16284 | 0.002897 |
| PA14_61020 | two_component_regulators | PGD1660782 | NA | Dcrc vs WT | -1.16284 | 0.002897 |
| PA14_61060 | energy_metabolism_CAP | PGD1660788 | NA | Dcrc vs WT | 1.051662 | 5.37E-10 |
| PA14_61060 | putative_enzymes | PGD1660788 | NA | Dcrc vs WT | 1.051662 | 5.37E-10 |
| PA14_61110 | hypothetical_unclassified | PGD1660794 | NA | Dcrc vs WT | -1.1208 | 0.003177 |
| PA14_61250 | transport_of_small_molecule | PGD1660816 | lysP | Dcrc vs WT | 1.226332 | 3.60E-06 |
| PA14_61370 | hypothetical_unclassified | PGD1660838 | NA | Dcrc vs WT | 1.233764 | 0.002137 |
| PA14_61410 | hypothetical_unclassified | PGD1660846 | NA | Dcrc vs WT | -1.00925 | 2.95E-08 |
| PA14_61770 | carbon_compound_catabolis | PGD1660906 | prs | Dcrc vs WT | 1.06952 | 5.74E-12 |
| PA14_61770 | nucleotide_biosynthesis_and | PGD1660906 | prs | Dcrc vs WT | 1.06952 | 5.74E-12 |
| PA14_61780 | adaptation_and_protection | PGD1660908 | NA | Dcrc vs WT | 1.1088 | 8.20E-09 |
| PA14_61780 | translation_posttranslational | PGD1660908 | NA | Dcrc vs WT | 1.1088 | 8.20E-09 |
| PA14_61790 | translation_posttranslational | PGD1660910 | pth | Dcrc vs WT | 1.15568 | 1.24E-10 |
| PA14_61870 | hypothetical_unclassified | PGD1660922 | NA | Dcrc vs WT | -1.31313 | 3.51E-10 |
| PA14_61980 | hypothetical_unclassified | PGD1660938 | NA | Dcrc vs WT | 1.179551 | 0.022501 |
| PA14_62240 | hypothetical_unclassified | PGD1660984 | NA | Dcrc vs WT | -1.33961 | 6.05E-09 |
| PA14_62710 | transcription_RNA_processin | PGD1661062 | pnp | Dcrc vs WT | 1.567016 | 3.22E-11 |
| PA14_63080 | transport_of_small_molecule | PGD1661126 | lldP | Dcrc vs WT | 1.880775 | 3.05E-13 |
| PA14_63100 | energy_metabolism_CAP | PGD1661130 | NA | Dcrc vs WT | 1.024628 | 1.58E-06 |
| PA14_63210 | two_component_regulators | PGD1661148 | NA | Dcrc vs WT | -1.12756 | 6.89E-08 |
| PA14_63240 | transcriptional_regulators | PGD1661154 | NA | Dcrc vs WT | 1.223193 | 0.003127 |
| PA14_63280 | transcriptional_regulators | PGD1661160 | NA | Dcrc vs WT | -1.10053 | 1.14E-12 |
| PA14_63700 | hypothetical_unclassified | PGD1661226 | NA | Dcrc vs WT | -1.67023 | 0.010249 |
| PA14_63720 | membrane_proteins | PGD1661230 | NA | Dcrc vs WT | -3.54298 | 0.045879 |
| PA14_63740 | hypothetical_unclassified | PGD1661234 | NA | Dcrc vs WT | -2.20497 | 1.37E-05 |
| PA14_63770 | hypothetical_unclassified | PGD1661238 | NA | Dcrc vs WT | -3.54571 | 0.028606 |
| PA14_63840 | membrane_proteins | PGD1661248 | NA | Dcrc vs WT | -1.44612 | 2.15E-05 |
| PA14_64060 | adaptation_and_protection | PGD1661280 | NA | Dcrc vs WT | -1.27217 | 0.000249 |
| PA14_64060 | chemotaxis | PGD1661280 | NA | Dcrc vs WT | -1.27217 | 0.000249 |

| | | | | | | |
|---|---|---|---|---|---|---|
| PA14_64100 | fatty_acid_and_phospholipid | PGD1661286 | accB | Dcrc vs WT | 1.027413 | 6.13E-04 |
| PA14_64310 | transport_of_small_molecule | PGD1661318 | NA | Dcrc vs WT | -1.14579 | 0.024814 |
| PA14_64320 | transport_of_small_molecule | PGD1661320 | NA | Dcrc vs WT | -1.14755 | 1.18E-09 |
| PA14_64370 | central_intermediary_metab | PGD1661328 | ureB | Dcrc vs WT | -1.30846 | 4.90E-05 |
| PA14_64480 | membrane_proteins | PGD1661348 | osmE | Dcrc vs WT | 1.645204 | 9.11E-16 |
| PA14_64530 | hypothetical_unclassified | PGD1661358 | NA | Dcrc vs WT | 3.910617 | 1.19E-14 |
| PA14_64620 | energy_metabolism_CAP | PGD1661374 | NA | Dcrc vs WT | -1.23478 | 0.001333 |
| PA14_64620 | putative_enzymes | PGD1661374 | NA | Dcrc vs WT | -1.23478 | 0.001333 |
| PA14_64810 | carbon_compound_catabolis | PGD1661406 | vanB | Dcrc vs WT | -1.23439 | 0.002044 |
| PA14_64860 | transport_of_small_molecule | PGD1661414 | NA | Dcrc vs WT | 1.147238 | 1.25E-04 |
| PA14_64870 | transport_of_small_molecule | PGD1661416 | NA | Dcrc vs WT | 1.477901 | 4.25E-07 |
| PA14_64880 | membrane_proteins | PGD1661418 | NA | Dcrc vs WT | 1.400072 | 6.26E-06 |
| PA14_64880 | transport_of_small_molecule | PGD1661418 | NA | Dcrc vs WT | 1.400072 | 6.26E-06 |
| PA14_64890 | membrane_proteins | PGD1661420 | NA | Dcrc vs WT | 1.988644 | 6.72E-12 |
| PA14_64890 | transport_of_small_molecule | PGD1661420 | NA | Dcrc vs WT | 1.988644 | 6.72E-12 |
| PA14_64900 | transport_of_small_molecule | PGD1661422 | NA | Dcrc vs WT | 1.193558 | 3.18E-15 |
| PA14_64940 | biosynthesis_of_cofactors | PGD1661430 | NA | Dcrc vs WT | -1.40613 | 4.65E-06 |
| PA14_64950 | central_intermediary_metab | PGD1661432 | pncA | Dcrc vs WT | -1.80566 | 1.04E-08 |
| PA14_65000 | energy_metabolism_CAP | PGD1661440 | azu | Dcrc vs WT | 1.168596 | 7.78E-13 |
| PA14_65090 | hypothetical_unclassified | PGD1661454 | NA | Dcrc vs WT | -1.05174 | 5.71E-10 |
| PA14_65150 | translation_posttranslational | PGD1661460 | rplI | Dcrc vs WT | 1.186306 | 3.78E-09 |
| PA14_65160 | membrane_proteins | PGD1661462 | NA | Dcrc vs WT | 1.116068 | 1.66E-09 |
| PA14_65170 | translation_posttranslational | PGD1661464 | rpsR | Dcrc vs WT | 1.642413 | 6.46E-17 |
| PA14_65180 | translation_posttranslational | PGD1661466 | rpsF | Dcrc vs WT | 1.000385 | 5.40E-10 |
| PA14_65960 | cell_wall_LPS | PGD1661578 | waaA | Dcrc vs WT | -1.33448 | 9.62E-07 |
| PA14_66460 | adaptation_and_protection | PGD1661656 | NA | Dcrc vs WT | -1.00807 | 1.53E-04 |
| PA14_66530 | transcriptional_regulators | PGD1661666 | NA | Dcrc vs WT | -1.28447 | 3.70E-04 |
| PA14_67370 | transport_of_small_molecule | PGD1661806 | NA | Dcrc vs WT | -1.29175 | 1.55E-08 |
| PA14_67540 | hypothetical_unclassified | PGD1661834 | NA | Dcrc vs WT | -1.16993 | 0.001084 |
| PA14_67550 | transcriptional_regulators | PGD1661836 | NA | Dcrc vs WT | -2.04399 | 4.91E-07 |
| PA14_67560 | adaptation_and_protection | PGD1661838 | typA | Dcrc vs WT | 1.210686 | 2.48E-14 |
| PA14_67720 | protein_secretion_export | PGD1661860 | secB | Dcrc vs WT | 1.402218 | 1.03E-21 |
| PA14_67740 | energy_metabolism_CAP | PGD1661862 | grx | Dcrc vs WT | 1.467745 | 8.59E-13 |
| PA14_67740 | nucleotide_biosynthesis_and | PGD1661862 | grx | Dcrc vs WT | 1.467745 | 8.59E-13 |
| PA14_68000 | hypothetical_unclassified | PGD1661904 | NA | Dcrc vs WT | 1.058147 | 6.44E-07 |
| PA14_68070 | transport_of_small_molecule | PGD1661916 | NA | Dcrc vs WT | 1.193184 | 3.45E-08 |
| PA14_68120 | cell_wall_LPS | PGD1661926 | NA | Dcrc vs WT | -1.25383 | 3.18E-05 |
| PA14_68190 | cell_wall_LPS | PGD1661936 | rmlD | Dcrc vs WT | -1.21389 | 3.33E-15 |
| PA14_68280 | membrane_proteins | PGD1661948 | NA | Dcrc vs WT | 1.054171 | 1.63E-05 |
| PA14_68280 | transport_of_small_molecule | PGD1661948 | NA | Dcrc vs WT | 1.054171 | 1.63E-05 |
| PA14_68630 | chaperones_heat_shock | PGD1662004 | NA | Dcrc vs WT | -1.04901 | 0.005144 |
| PA14_68720 | hypothetical_unclassified | PGD1662018 | NA | Dcrc vs WT | -1.40704 | 0.005135 |
| PA14_69600 | hypothetical_unclassified | PGD1662160 | NA | Dcrc vs WT | -1.16534 | 4.72E-08 |
| PA14_69820 | hypothetical_unclassified | PGD1662196 | NA | Dcrc vs WT | 1.088077 | 2.63E-05 |
| PA14_69925 | central_intermediary_metab | PGD1662212 | poxB | Dcrc vs WT | 1.503909 | 2.76E-06 |
| PA14_69925 | energy_metabolism_CAP | PGD1662212 | poxB | Dcrc vs WT | 1.503909 | 2.76E-06 |
| PA14_70050 | hypothetical_unclassified | PGD1662228 | NA | Dcrc vs WT | -1.10019 | 2.77E-11 |
| PA14_70390 | carbon_compound_catabolis | PGD1662282 | crc | Dcrc vs WT | -2.86367 | 8.67E-57 |
| PA14_70390 | DNA_replication_recombinat | PGD1662282 | crc | Dcrc vs WT | -2.86367 | 8.67E-57 |
| PA14_70390 | energy_metabolism_CAP | PGD1662282 | crc | Dcrc vs WT | -2.86367 | 8.67E-57 |

| | | | | | | |
|---|---|---|---|---|---|---|
| PA14_70510 | transport_of_small_molecule | PGD1662300 | NA | Dcrc vs WT | -1.42672 | 0.012655 |
| PA14_70640 | carbon_compound_catabolis | PGD1662320 | rubA1 | Dcrc vs WT | 1.051361 | 2.43E-05 |
| PA14_70650 | putative_enzymes | PGD1662322 | NA | Dcrc vs WT | 1.072907 | 0.004946 |
| PA14_70670 | carbon_compound_catabolis | PGD1662324 | glcF | Dcrc vs WT | 1.276561 | 1.67E-04 |
| PA14_70670 | central_intermediary_metab | PGD1662324 | glcF | Dcrc vs WT | 1.276561 | 1.67E-04 |
| PA14_70670 | energy_metabolism_CAP | PGD1662324 | glcF | Dcrc vs WT | 1.276561 | 1.67E-04 |
| PA14_70690 | carbon_compound_catabolis | PGD1662328 | glcD | Dcrc vs WT | 1.064571 | 2.07E-05 |
| PA14_70690 | central_intermediary_metab | PGD1662328 | glcD | Dcrc vs WT | 1.064571 | 2.07E-05 |
| PA14_71060 | amino_acid_biosynthesis_me | PGD1662386 | sdaB | Dcrc vs WT | -1.33216 | 1.09E-06 |
| PA14_71080 | hypothetical_unclassified | PGD1662390 | NA | Dcrc vs WT | -1.43898 | 1.04E-06 |
| PA14_71100 | membrane_proteins | PGD1662394 | NA | Dcrc vs WT | 2.354421 | 6.82E-06 |
| PA14_71240 | amino_acid_biosynthesis_me | PGD1662420 | NA | Dcrc vs WT | -1.03047 | 0.046621 |
| PA14_71250 | hypothetical_unclassified | PGD1662422 | NA | Dcrc vs WT | -1.80893 | 0.008145 |
| PA14_71260 | amino_acid_biosynthesis_me | PGD1662424 | NA | Dcrc vs WT | -1.24456 | 1.28E-06 |
| PA14_71260 | energy_metabolism_CAP | PGD1662424 | NA | Dcrc vs WT | -1.24456 | 1.28E-06 |
| PA14_71280 | amino_acid_biosynthesis_me | PGD1662426 | NA | Dcrc vs WT | -1.95724 | 1.21E-06 |
| PA14_71280 | energy_metabolism_CAP | PGD1662426 | NA | Dcrc vs WT | -1.95724 | 1.21E-06 |
| PA14_71330 | transcriptional_regulators | PGD1662434 | NA | Dcrc vs WT | 1.003241 | 0.047303 |
| PA14_71360 | hypothetical_unclassified | PGD1662440 | NA | Dcrc vs WT | 1.083435 | 2.60E-04 |
| PA14_71380 | hypothetical_unclassified | PGD1662444 | NA | Dcrc vs WT | -1.60874 | 3.65E-10 |
| PA14_71390 | membrane_proteins | PGD1662446 | NA | Dcrc vs WT | -1.08469 | 1.84E-07 |
| PA14_71400 | hypothetical_unclassified | PGD1662448 | NA | Dcrc vs WT | -1.40371 | 8.19E-04 |
| PA14_71410 | transport_of_small_molecule | PGD1662450 | NA | Dcrc vs WT | -1.8209 | 0.009237 |
| PA14_71420 | energy_metabolism_CAP | PGD1662452 | NA | Dcrc vs WT | -1.11364 | 0.002047 |
| PA14_71460 | amino_acid_biosynthesis_me | PGD1662460 | glyA1 | Dcrc vs WT | -1.95423 | 4.30E-04 |
| PA14_71470 | amino_acid_biosynthesis_me | PGD1662462 | soxB | Dcrc vs WT | -1.79935 | 3.35E-06 |
| PA14_71470 | carbon_compound_catabolis | PGD1662462 | soxB | Dcrc vs WT | -1.79935 | 3.35E-06 |
| PA14_71490 | amino_acid_biosynthesis_me | PGD1662464 | soxD | Dcrc vs WT | -2.40998 | 0.01654 |
| PA14_71490 | carbon_compound_catabolis | PGD1662464 | soxD | Dcrc vs WT | -2.40998 | 0.01654 |
| PA14_71500 | carbon_compound_catabolis | PGD1662466 | soxA | Dcrc vs WT | -1.93752 | 2.61E-07 |
| PA14_71510 | amino_acid_biosynthesis_me | PGD1662468 | soxG | Dcrc vs WT | -1.36712 | 0.011464 |
| PA14_71510 | carbon_compound_catabolis | PGD1662468 | soxG | Dcrc vs WT | -1.36712 | 0.011464 |
| PA14_71530 | nucleotide_biosynthesis_and | PGD1662470 | purU2 | Dcrc vs WT | -1.3618 | 0.009757 |
| PA14_71560 | amino_acid_biosynthesis_me | PGD1662472 | fdhA | Dcrc vs WT | -1.56424 | 1.87E-04 |
| PA14_71750 | transcriptional_regulators | PGD1662504 | NA | Dcrc vs WT | 1.394546 | 7.55E-18 |
| PA14_72620 | transport_of_small_molecule | PGD1662640 | metN | Dcrc vs WT | 1.268069 | 8.99E-11 |
| PA14_72660 | biosynthesis_of_cofactors | PGD1662648 | NA | Dcrc vs WT | 1.40368 | 0.001484 |
| PA14_73110 | membrane_proteins | PGD1662724 | NA | Dcrc vs WT | 1.008188 | 2.94E-08 |
| PA14_73110 | transport_of_small_molecule | PGD1662724 | NA | Dcrc vs WT | 1.008188 | 2.94E-08 |
| PA14_73240 | energy_metabolism_CAP | PGD1662744 | atpD | Dcrc vs WT | 1.158163 | 6.13E-09 |
| PA14_73280 | energy_metabolism_CAP | PGD1662750 | atpH | Dcrc vs WT | 1.321896 | 6.43E-12 |
| PA14_73290 | energy_metabolism_CAP | PGD1662752 | atpF | Dcrc vs WT | 1.42005 | 6.23E-19 |
| PA14_73420 | transcription_RNA_processin | PGD1662774 | rnpA | Dcrc vs WT | 1.043445 | 1.37E-15 |
| PA14_73420 | translation_posttranslational | PGD1662774 | rnpA | Dcrc vs WT | 1.043445 | 1.37E-15 |
| PA14_99999 | NA | gene_crcZ | crcZ | Dcrc vs WT | -3.86128 | 3.71E-04 |
| PA14_00190 | amino_acid_biosynthesis_me | PGD1650869 | fmt | DrhlR DcbrA Dcrc vs WT | 1.58615 | 5.43E-08 |
| PA14_00190 | translation_posttranslational | PGD1650869 | fmt | DrhlR DcbrA Dcrc vs WT | 1.58615 | 5.43E-08 |
| PA14_00210 | putative_enzymes | PGD1650873 | NA | DrhlR DcbrA Dcrc vs WT | 1.504761 | 6.69E-17 |
| PA14_00230 | DNA_replication_recombinat | PGD1650875 | NA | DrhlR DcbrA Dcrc vs WT | 1.068131 | 0.006598 |
| PA14_00230 | membrane_proteins | PGD1650875 | NA | DrhlR DcbrA Dcrc vs WT | 1.068131 | 0.006598 |

| | | | | | | |
|---|---|---|---|---|---|---|
| PA14_00620 | hypothetical_unclassified | PGD1650935 | NA | DrhlR DcbrA Dcrc vs WT | 1.787652 | 4.41E-20 |
| PA14_00640 | putative_enzymes | PGD1650939 | phzH | DrhlR DcbrA Dcrc vs WT | -5.2946 | 1.66E-39 |
| PA14_00800 | nucleotide_biosynthesis_and | PGD1650971 | NA | DrhlR DcbrA Dcrc vs WT | -1.22789 | 2.68E-07 |
| PA14_00860 | protein_secretion_export | PGD1650981 | tagT1 | DrhlR DcbrA Dcrc vs WT | 2.158065 | 3.76E-05 |
| PA14_00860 | transport_of_small_molecule | PGD1650981 | tagT1 | DrhlR DcbrA Dcrc vs WT | 2.158065 | 3.76E-05 |
| PA14_01350 | hypothetical_unclassified | PGD1651061 | NA | DrhlR DcbrA Dcrc vs WT | 1.659956 | 2.51E-08 |
| PA14_01360 | energy_metabolism_CAP | PGD1651063 | NA | DrhlR DcbrA Dcrc vs WT | 1.100186 | 5.32E-04 |
| PA14_01360 | membrane_proteins | PGD1651063 | NA | DrhlR DcbrA Dcrc vs WT | 1.100186 | 5.32E-04 |
| PA14_01480 | transcriptional_regulators | PGD1651081 | NA | DrhlR DcbrA Dcrc vs WT | 1.027992 | 5.46E-04 |
| PA14_01490 | adaptation_and_protection | PGD1651083 | NA | DrhlR DcbrA Dcrc vs WT | -5.22712 | 2.52E-59 |
| PA14_01490 | putative_enzymes | PGD1651083 | NA | DrhlR DcbrA Dcrc vs WT | -5.22712 | 2.52E-59 |
| PA14_01620 | amino_acid_biosynthesis_me | PGD1651103 | aptA | DrhlR DcbrA Dcrc vs WT | 1.580743 | 1.23E-09 |
| PA14_01620 | carbon_compound_catabolis | PGD1651103 | aptA | DrhlR DcbrA Dcrc vs WT | 1.580743 | 1.23E-09 |
| PA14_01790 | nucleotide_biosynthesis_and | PGD1651129 | NA | DrhlR DcbrA Dcrc vs WT | 1.036155 | 5.98E-05 |
| PA14_01860 | transport_of_small_molecule | PGD1651139 | NA | DrhlR DcbrA Dcrc vs WT | 1.843924 | 5.67E-06 |
| PA14_01960 | transport_of_small_molecule | PGD1651153 | NA | DrhlR DcbrA Dcrc vs WT | 1.003544 | 2.68E-05 |
| PA14_02060 | membrane_proteins | PGD1651169 | NA | DrhlR DcbrA Dcrc vs WT | 1.35171 | 0.001799 |
| PA14_02190 | chemotaxis | PGD1651187 | NA | DrhlR DcbrA Dcrc vs WT | -1.25334 | 0.003685 |
| PA14_02290 | transcriptional_regulators | PGD1651201 | NA | DrhlR DcbrA Dcrc vs WT | 1.005568 | 2.66E-04 |
| PA14_02560 | biosynthesis_of_cofactors | PGD1651243 | NA | DrhlR DcbrA Dcrc vs WT | 1.218078 | 0.001656 |
| PA14_02730 | amino_acid_biosynthesis_me | PGD1651271 | NA | DrhlR DcbrA Dcrc vs WT | -1.48282 | 2.66E-04 |
| PA14_02760 | carbon_compound_catabolis | PGD1651277 | NA | DrhlR DcbrA Dcrc vs WT | -2.30502 | 1.17E-12 |
| PA14_02770 | carbon_compound_catabolis | PGD1651279 | NA | DrhlR DcbrA Dcrc vs WT | -2.25978 | 1.03E-07 |
| PA14_02790 | carbon_compound_catabolis | PGD1651281 | pcaF | DrhlR DcbrA Dcrc vs WT | -2.16185 | 2.31E-13 |
| PA14_02790 | fatty_acid_and_phospholipid | PGD1651281 | pcaF | DrhlR DcbrA Dcrc vs WT | -2.16185 | 2.31E-13 |
| PA14_02890 | hypothetical_unclassified | PGD1651293 | NA | DrhlR DcbrA Dcrc vs WT | 2.218118 | 8.04E-11 |
| PA14_02900 | carbon_compound_catabolis | PGD1651295 | pcaK | DrhlR DcbrA Dcrc vs WT | -1.93389 | 1.56E-10 |
| PA14_02900 | membrane_proteins | PGD1651295 | pcaK | DrhlR DcbrA Dcrc vs WT | -1.93389 | 1.56E-10 |
| PA14_02900 | transport_of_small_molecule | PGD1651295 | pcaK | DrhlR DcbrA Dcrc vs WT | -1.93389 | 1.56E-10 |
| PA14_02970 | membrane_proteins | PGD1651303 | NA | DrhlR DcbrA Dcrc vs WT | 2.571221 | 1.43E-15 |
| PA14_02980 | membrane_proteins | PGD1651305 | NA | DrhlR DcbrA Dcrc vs WT | -1.38493 | 0.041879 |
| PA14_03050 | carbon_compound_catabolis | PGD1651319 | pobA | DrhlR DcbrA Dcrc vs WT | -1.28513 | 0.002417 |
| PA14_03110 | hypothetical_unclassified | PGD1651329 | NA | DrhlR DcbrA Dcrc vs WT | 2.809941 | 5.10E-15 |
| PA14_03240 | secreted_factors | PGD1651355 | hcpC | DrhlR DcbrA Dcrc vs WT | -1.55538 | 7.51E-07 |
| PA14_03430 | central_intermediary_metab | PGD1651393 | gabD | DrhlR DcbrA Dcrc vs WT | 1.301758 | 7.73E-16 |
| PA14_03450 | amino_acid_biosynthesis_me | PGD1651395 | gabT | DrhlR DcbrA Dcrc vs WT | 1.289257 | 8.31E-10 |
| PA14_03450 | carbon_compound_catabolis | PGD1651395 | gabT | DrhlR DcbrA Dcrc vs WT | 1.289257 | 8.31E-10 |
| PA14_03450 | central_intermediary_metab | PGD1651395 | gabT | DrhlR DcbrA Dcrc vs WT | 1.289257 | 8.31E-10 |
| PA14_03490 | carbon_compound_catabolis | PGD1651401 | NA | DrhlR DcbrA Dcrc vs WT | -3.53733 | 3.68E-78 |
| PA14_03510 | hypothetical_unclassified | PGD1651403 | NA | DrhlR DcbrA Dcrc vs WT | -3.70781 | 5.06E-33 |
| PA14_03520 | hypothetical_unclassified | PGD1651405 | NA | DrhlR DcbrA Dcrc vs WT | -3.88596 | 1.44E-31 |
| PA14_03550 | transport_of_small_molecule | PGD1651409 | NA | DrhlR DcbrA Dcrc vs WT | 1.473127 | 0.030963 |
| PA14_03630 | transcriptional_regulators | PGD1651421 | NA | DrhlR DcbrA Dcrc vs WT | 1.667248 | 1.06E-06 |
| PA14_03855 | transport_of_small_molecule | PGD1651453 | NA | DrhlR DcbrA Dcrc vs WT | 1.364234 | 7.74E-09 |
| PA14_03870 | amino_acid_biosynthesis_me | PGD1651457 | spuA | DrhlR DcbrA Dcrc vs WT | 1.188569 | 5.50E-08 |
| PA14_03880 | amino_acid_biosynthesis_me | PGD1651459 | spuB | DrhlR DcbrA Dcrc vs WT | 1.365689 | 1.41E-19 |
| PA14_03880 | carbon_compound_catabolis | PGD1651459 | spuB | DrhlR DcbrA Dcrc vs WT | 1.365689 | 1.41E-19 |
| PA14_03880 | putative_enzymes | PGD1651459 | spuB | DrhlR DcbrA Dcrc vs WT | 1.365689 | 1.41E-19 |
| PA14_03900 | biosynthesis_of_cofactors | PGD1651461 | spuC | DrhlR DcbrA Dcrc vs WT | 1.242984 | 5.93E-22 |
| PA14_03980 | carbon_compound_catabolis | PGD1651473 | NA | DrhlR DcbrA Dcrc vs WT | 1.285003 | 2.60E-08 |

| | | | | | | |
|---|---|---|---|---|---|---|
| PA14_03980 | central_intermediary_metab | PGD1651473 | NA | DrhlR DcbrA Dcrc vs WT | 1.285003 | 2.60E-08 |
| PA14_04180 | hypothetical_unclassified | PGD1651505 | NA | DrhlR DcbrA Dcrc vs WT | 1.292446 | 0.006768 |
| PA14_04210 | transport_of_small_molecule | PGD1651509 | NA | DrhlR DcbrA Dcrc vs WT | -1.4715 | 0.002066 |
| PA14_04290 | amino_acid_biosynthesis_me | PGD1651521 | NA | DrhlR DcbrA Dcrc vs WT | 1.207862 | 7.61E-12 |
| PA14_04290 | membrane_proteins | PGD1651521 | NA | DrhlR DcbrA Dcrc vs WT | 1.207862 | 7.61E-12 |
| PA14_04330 | membrane_proteins | PGD1651529 | NA | DrhlR DcbrA Dcrc vs WT | 1.227615 | 2.89E-07 |
| PA14_04530 | hypothetical_unclassified | PGD1651559 | NA | DrhlR DcbrA Dcrc vs WT | 1.129441 | 4.68E-09 |
| PA14_04690 | hypothetical_unclassified | PGD1651585 | NA | DrhlR DcbrA Dcrc vs WT | 1.321699 | 8.69E-10 |
| PA14_04780 | energy_metabolism_CAP | PGD1651597 | laoA | DrhlR DcbrA Dcrc vs WT | -1.14154 | 7.45E-07 |
| PA14_04790 | membrane_proteins | PGD1651599 | laoB | DrhlR DcbrA Dcrc vs WT | -1.1444 | 2.00E-06 |
| PA14_04810 | energy_metabolism_CAP | PGD1651601 | laoC | DrhlR DcbrA Dcrc vs WT | -1.02115 | 1.35E-12 |
| PA14_04810 | putative_enzymes | PGD1651601 | laoC | DrhlR DcbrA Dcrc vs WT | -1.02115 | 1.35E-12 |
| PA14_05110 | hypothetical_unclassified | PGD1651653 | NA | DrhlR DcbrA Dcrc vs WT | 1.97734 | 1.08E-25 |
| PA14_05250 | nucleotide_biosynthesis_and | PGD1651675 | pyrC | DrhlR DcbrA Dcrc vs WT | 1.232628 | 8.72E-10 |
| PA14_05330 | chemotaxis | PGD1651691 | pilH | DrhlR DcbrA Dcrc vs WT | 1.074456 | 4.09E-07 |
| PA14_05330 | motility_and_attachment | PGD1651691 | pilH | DrhlR DcbrA Dcrc vs WT | 1.074456 | 4.09E-07 |
| PA14_05330 | two_component_regulators | PGD1651691 | pilH | DrhlR DcbrA Dcrc vs WT | 1.074456 | 4.09E-07 |
| PA14_05340 | chemotaxis | PGD1651693 | pilI | DrhlR DcbrA Dcrc vs WT | 1.111642 | 5.37E-07 |
| PA14_05340 | motility_and_attachment | PGD1651693 | pilI | DrhlR DcbrA Dcrc vs WT | 1.111642 | 5.37E-07 |
| PA14_05360 | chemotaxis | PGD1651695 | pilJ | DrhlR DcbrA Dcrc vs WT | 1.318295 | 7.92E-14 |
| PA14_05390 | chemotaxis | PGD1651699 | chpA | DrhlR DcbrA Dcrc vs WT | 1.165669 | 5.11E-14 |
| PA14_05390 | motility_and_attachment | PGD1651699 | chpA | DrhlR DcbrA Dcrc vs WT | 1.165669 | 5.11E-14 |
| PA14_05390 | two_component_regulators | PGD1651699 | chpA | DrhlR DcbrA Dcrc vs WT | 1.165669 | 5.11E-14 |
| PA14_05630 | hypothetical_unclassified | PGD1651739 | NA | DrhlR DcbrA Dcrc vs WT | 1.40617 | 3.78E-05 |
| PA14_05640 | membrane_proteins | PGD1651741 | NA | DrhlR DcbrA Dcrc vs WT | 1.001827 | 0.001417 |
| PA14_05660 | transcriptional_regulators | PGD1651745 | NA | DrhlR DcbrA Dcrc vs WT | -1.63111 | 1.33E-25 |
| PA14_05690 | nucleotide_biosynthesis_and | PGD1651747 | codA | DrhlR DcbrA Dcrc vs WT | 1.026357 | 0.001826 |
| PA14_05740 | nucleotide_biosynthesis_and | PGD1651751 | NA | DrhlR DcbrA Dcrc vs WT | 3.364663 | 1.22E-06 |
| PA14_05740 | putative_enzymes | PGD1651751 | NA | DrhlR DcbrA Dcrc vs WT | 3.364663 | 1.22E-06 |
| PA14_05750 | amino_acid_biosynthesis_me | PGD1651753 | NA | DrhlR DcbrA Dcrc vs WT | 3.199715 | 1.38E-10 |
| PA14_05750 | putative_enzymes | PGD1651753 | NA | DrhlR DcbrA Dcrc vs WT | 3.199715 | 1.38E-10 |
| PA14_05810 | nucleotide_biosynthesis_and | PGD1651761 | amaB | DrhlR DcbrA Dcrc vs WT | 1.240057 | 1.99E-06 |
| PA14_05890 | membrane_proteins | PGD1651775 | NA | DrhlR DcbrA Dcrc vs WT | -1.36229 | 6.06E-09 |
| PA14_06000 | translation_posttranslational | PGD1651789 | NA | DrhlR DcbrA Dcrc vs WT | 1.114504 | 7.50E-08 |
| PA14_06230 | transport_of_small_molecule | PGD1651823 | NA | DrhlR DcbrA Dcrc vs WT | -1.22809 | 0.002752 |
| PA14_06540 | biosynthesis_of_cofactors | PGD1651871 | NA | DrhlR DcbrA Dcrc vs WT | 1.602142 | 2.79E-06 |
| PA14_06570 | biosynthesis_of_cofactors | PGD1651873 | bioD | DrhlR DcbrA Dcrc vs WT | 1.194292 | 3.66E-04 |
| PA14_06710 | biosynthesis_of_cofactors | PGD1651895 | NA | DrhlR DcbrA Dcrc vs WT | 1.369796 | 1.53E-06 |
| PA14_06710 | energy_metabolism_CAP | PGD1651895 | NA | DrhlR DcbrA Dcrc vs WT | 1.369796 | 1.53E-06 |
| PA14_06710 | transcriptional_regulators | PGD1651895 | NA | DrhlR DcbrA Dcrc vs WT | 1.369796 | 1.53E-06 |
| PA14_06720 | energy_metabolism_CAP | PGD1651897 | nirF | DrhlR DcbrA Dcrc vs WT | 1.18237 | 4.81E-05 |
| PA14_06730 | biosynthesis_of_cofactors | PGD1651899 | nirC | DrhlR DcbrA Dcrc vs WT | 1.511123 | 5.46E-06 |
| PA14_06730 | energy_metabolism_CAP | PGD1651899 | nirC | DrhlR DcbrA Dcrc vs WT | 1.511123 | 5.46E-06 |
| PA14_06750 | energy_metabolism_CAP | PGD1651903 | nirS | DrhlR DcbrA Dcrc vs WT | 1.329007 | 1.46E-06 |
| PA14_06790 | energy_metabolism_CAP | PGD1651907 | NA | DrhlR DcbrA Dcrc vs WT | -1.09807 | 0.001529 |
| PA14_06860 | hypothetical_unclassified | PGD1651917 | NA | DrhlR DcbrA Dcrc vs WT | 1.43504 | 3.81E-07 |
| PA14_06870 | transcriptional_regulators | PGD1651919 | dnr | DrhlR DcbrA Dcrc vs WT | 1.541383 | 1.64E-10 |
| NA | NA | PGD1651922 | NA | DrhlR DcbrA Dcrc vs WT | -2.12884 | 6.36E-05 |
| PA14_07070 | energy_metabolism_CAP | PGD1651959 | NA | DrhlR DcbrA Dcrc vs WT | 1.054233 | 1.76E-05 |
| PA14_07430 | hypothetical_unclassified | PGD1652013 | NA | DrhlR DcbrA Dcrc vs WT | -1.37867 | 6.48E-07 |

| | | | | | | |
|---|---|---|---|---|---|---|
| PA14_07710 | hypothetical_unclassified | PGD1652057 | apaG | DrhlR DcbrA Dcrc vs WT | -1.15188 | 1.48E-08 |
| PA14_07850 | transport_of_small_molecule | PGD1652079 | NA | DrhlR DcbrA Dcrc vs WT | 1.992922 | 1.52E-19 |
| PA14_07970 | transcription_RNA_processin | PGD1652099 | NA | DrhlR DcbrA Dcrc vs WT | 2.268505 | 2.35E-06 |
| PA14_07980 | hypothetical_unclassified | PGD1652101 | NA | DrhlR DcbrA Dcrc vs WT | 1.085038 | 0.010121 |
| PA14_08010 | relative_phage_transposon | PGD1652107 | NA | DrhlR DcbrA Dcrc vs WT | 2.268452 | 1.02E-04 |
| PA14_08020 | relative_phage_transposon | PGD1652109 | NA | DrhlR DcbrA Dcrc vs WT | 2.777447 | 4.53E-05 |
| PA14_08030 | relative_phage_transposon | PGD1652111 | NA | DrhlR DcbrA Dcrc vs WT | 2.502838 | 1.08E-08 |
| PA14_08040 | relative_phage_transposon | PGD1652113 | NA | DrhlR DcbrA Dcrc vs WT | 3.286418 | 1.75E-06 |
| PA14_08070 | relative_phage_transposon | PGD1652119 | NA | DrhlR DcbrA Dcrc vs WT | 1.841724 | 1.74E-09 |
| PA14_08090 | relative_phage_transposon | PGD1652121 | NA | DrhlR DcbrA Dcrc vs WT | 1.9168 | 1.23E-06 |
| PA14_08100 | relative_phage_transposon | PGD1652123 | NA | DrhlR DcbrA Dcrc vs WT | 1.554578 | 4.87E-04 |
| PA14_08110 | hypothetical_unclassified | PGD1652125 | NA | DrhlR DcbrA Dcrc vs WT | 2.011318 | 0.014614 |
| PA14_08120 | relative_phage_transposon | PGD1652127 | NA | DrhlR DcbrA Dcrc vs WT | 2.042094 | 8.38E-06 |
| PA14_08130 | relative_phage_transposon | PGD1652129 | NA | DrhlR DcbrA Dcrc vs WT | 1.694918 | 2.51E-06 |
| PA14_08140 | relative_phage_transposon | PGD1652131 | NA | DrhlR DcbrA Dcrc vs WT | 1.845523 | 0.017091 |
| PA14_08150 | relative_phage_transposon | PGD1652133 | NA | DrhlR DcbrA Dcrc vs WT | 2.673334 | 3.02E-09 |
| PA14_08160 | putative_enzymes | PGD1652135 | NA | DrhlR DcbrA Dcrc vs WT | 1.974912 | 4.96E-04 |
| PA14_08180 | hypothetical_unclassified | PGD1652137 | NA | DrhlR DcbrA Dcrc vs WT | 3.000219 | 9.95E-04 |
| PA14_08190 | hypothetical_unclassified | PGD1652139 | NA | DrhlR DcbrA Dcrc vs WT | 2.956025 | 1.83E-04 |
| PA14_08200 | relative_phage_transposon | PGD1652141 | NA | DrhlR DcbrA Dcrc vs WT | 2.539933 | 5.42E-04 |
| PA14_08210 | relative_phage_transposon | PGD1652143 | NA | DrhlR DcbrA Dcrc vs WT | 2.205659 | 3.39E-09 |
| PA14_08220 | hypothetical_unclassified | PGD1652145 | NA | DrhlR DcbrA Dcrc vs WT | 2.290958 | 8.56E-08 |
| PA14_08230 | hypothetical_unclassified | PGD1652147 | NA | DrhlR DcbrA Dcrc vs WT | 1.494409 | 0.001182 |
| PA14_08240 | relative_phage_transposon | PGD1652149 | NA | DrhlR DcbrA Dcrc vs WT | 2.572719 | 3.10E-07 |
| PA14_08250 | relative_phage_transposon | PGD1652151 | NA | DrhlR DcbrA Dcrc vs WT | 2.092172 | 1.91E-04 |
| PA14_08260 | relative_phage_transposon | PGD1652153 | NA | DrhlR DcbrA Dcrc vs WT | 2.131649 | 4.36E-05 |
| PA14_08270 | hypothetical_unclassified | PGD1652155 | NA | DrhlR DcbrA Dcrc vs WT | 2.926411 | 1.14E-07 |
| PA14_08280 | relative_phage_transposon | PGD1652157 | NA | DrhlR DcbrA Dcrc vs WT | 1.84622 | 7.26E-05 |
| PA14_08300 | relative_phage_transposon | PGD1652159 | NA | DrhlR DcbrA Dcrc vs WT | 1.425683 | 6.90E-05 |
| PA14_09160 | adaptation_and_protection | PGD1652315 | bfrA | DrhlR DcbrA Dcrc vs WT | 1.20144 | 3.51E-13 |
| PA14_09160 | transport_of_small_molecule | PGD1652315 | bfrA | DrhlR DcbrA Dcrc vs WT | 1.20144 | 3.51E-13 |
| PA14_09370 | antibiotic_resistance_and_su | PGD1652347 | NA | DrhlR DcbrA Dcrc vs WT | 1.057544 | 0.001826 |
| PA14_09370 | membrane_proteins | PGD1652347 | NA | DrhlR DcbrA Dcrc vs WT | 1.057544 | 0.001826 |
| PA14_09400 | putative_enzymes | PGD1652351 | phzS | DrhlR DcbrA Dcrc vs WT | -2.02508 | 9.37E-11 |
| PA14_09410 | secreted_factors | PGD1652353 | phzG1 | DrhlR DcbrA Dcrc vs WT | -2.12792 | 0.005587 |
| PA14_09440 | secreted_factors | PGD1652357 | phzE1 | DrhlR DcbrA Dcrc vs WT | -5.99865 | 5.99E-18 |
| PA14_09450 | secreted_factors | PGD1652359 | phzD1 | DrhlR DcbrA Dcrc vs WT | -3.67111 | 6.47E-05 |
| PA14_09460 | secreted_factors | PGD1652361 | phzC1 | DrhlR DcbrA Dcrc vs WT | -1.64815 | 0.004309 |
| PA14_09470 | secreted_factors | PGD1652363 | phzB1 | DrhlR DcbrA Dcrc vs WT | -1.96471 | 9.35E-05 |
| PA14_09480 | secreted_factors | PGD1652365 | phzA1 | DrhlR DcbrA Dcrc vs WT | -6.96736 | 1.67E-07 |
| PA14_09490 | putative_enzymes | PGD1652367 | phzM | DrhlR DcbrA Dcrc vs WT | -1.31801 | 3.24E-09 |
| PA14_09500 | membrane_proteins | PGD1652369 | opmD | DrhlR DcbrA Dcrc vs WT | -3.83901 | 1.98E-17 |
| PA14_09500 | transport_of_small_molecule | PGD1652369 | opmD | DrhlR DcbrA Dcrc vs WT | -3.83901 | 1.98E-17 |
| PA14_09520 | transport_of_small_molecule | PGD1652371 | mexI | DrhlR DcbrA Dcrc vs WT | -3.33924 | 5.95E-45 |
| PA14_09530 | transport_of_small_molecule | PGD1652373 | mexH | DrhlR DcbrA Dcrc vs WT | -5.34542 | 2.35E-25 |
| PA14_09540 | membrane_proteins | PGD1652375 | mexG | DrhlR DcbrA Dcrc vs WT | -4.63629 | 3.60E-21 |
| PA14_09810 | hypothetical_unclassified | PGD1652413 | NA | DrhlR DcbrA Dcrc vs WT | 2.241893 | 0.002055 |
| PA14_09870 | hypothetical_unclassified | PGD1652419 | eftM | DrhlR DcbrA Dcrc vs WT | 1.50249 | 2.28E-06 |
| PA14_09880 | hypothetical_unclassified | PGD1652421 | NA | DrhlR DcbrA Dcrc vs WT | 1.781963 | 1.17E-04 |
| PA14_09900 | putative_enzymes | PGD1652425 | prpL | DrhlR DcbrA Dcrc vs WT | 2.290219 | 4.01E-17 |

| | | | | | | |
|---|---|---|---|---|---|---|
| PA14_09900 | secreted_factors | PGD1652425 | prpL | DrhlR DcbrA Dcrc vs WT | 2.290219 | 4.01E-17 |
| PA14_10160 | transport_of_small_molecule | PGD1652467 | fepD | DrhlR DcbrA Dcrc vs WT | 1.659198 | 0.015278 |
| PA14_10170 | transport_of_small_molecule | PGD1652469 | fepB | DrhlR DcbrA Dcrc vs WT | 1.778368 | 2.40E-06 |
| PA14_10200 | membrane_proteins | PGD1652475 | NA | DrhlR DcbrA Dcrc vs WT | 1.560579 | 1.08E-05 |
| PA14_10240 | carbon_compound_catabolis | PGD1652483 | NA | DrhlR DcbrA Dcrc vs WT | 1.587256 | 0.017486 |
| PA14_10250 | carbon_compound_catabolis | PGD1652485 | acoB | DrhlR DcbrA Dcrc vs WT | 1.803073 | 9.72E-04 |
| PA14_10260 | carbon_compound_catabolis | PGD1652487 | NA | DrhlR DcbrA Dcrc vs WT | 1.715929 | 8.27E-05 |
| PA14_10270 | carbon_compound_catabolis | PGD1652489 | NA | DrhlR DcbrA Dcrc vs WT | 4.127853 | 0.003005 |
| PA14_10290 | transcriptional_regulators | PGD1652493 | acoR | DrhlR DcbrA Dcrc vs WT | 2.207562 | 1.32E-38 |
| PA14_10300 | protein_secretion_export | PGD1652495 | NA | DrhlR DcbrA Dcrc vs WT | 1.240119 | 0.001356 |
| PA14_10330 | protein_secretion_export | PGD1652499 | NA | DrhlR DcbrA Dcrc vs WT | -1.58881 | 7.87E-12 |
| PA14_10340 | protein_secretion_export | PGD1652501 | NA | DrhlR DcbrA Dcrc vs WT | -1.16844 | 2.23E-05 |
| PA14_10350 | protein_secretion_export | PGD1652503 | NA | DrhlR DcbrA Dcrc vs WT | -3.11458 | 6.92E-57 |
| PA14_10360 | hypothetical_unclassified | PGD1652505 | NA | DrhlR DcbrA Dcrc vs WT | -6.33065 | 6.70E-193 |
| PA14_10370 | fatty_acid_and_phospholipid | PGD1652507 | NA | DrhlR DcbrA Dcrc vs WT | -1.04413 | 3.86E-04 |
| PA14_10380 | hypothetical_unclassified | PGD1652509 | NA | DrhlR DcbrA Dcrc vs WT | -1.37978 | 9.97E-20 |
| PA14_10420 | amino_acid_biosynthesis_me | PGD1652515 | tyrS | DrhlR DcbrA Dcrc vs WT | 1.041812 | 1.66E-04 |
| PA14_10420 | translation_posttranslational | PGD1652515 | tyrS | DrhlR DcbrA Dcrc vs WT | 1.041812 | 1.66E-04 |
| PA14_10470 | membrane_proteins | PGD1652519 | NA | DrhlR DcbrA Dcrc vs WT | 1.090275 | 2.26E-04 |
| PA14_10470 | transport_of_small_molecule | PGD1652519 | NA | DrhlR DcbrA Dcrc vs WT | 1.090275 | 2.26E-04 |
| PA14_10570 | carbon_compound_catabolis | PGD1652535 | NA | DrhlR DcbrA Dcrc vs WT | 1.291103 | 1.87E-04 |
| PA14_11000 | carbon_compound_catabolis | PGD1652605 | hpaA | DrhlR DcbrA Dcrc vs WT | 1.379796 | 1.32E-12 |
| PA14_11130 | putative_enzymes | PGD1652629 | NA | DrhlR DcbrA Dcrc vs WT | -1.46702 | 3.37E-19 |
| PA14_11140 | biosynthesis_of_cofactors | PGD1652631 | NA | DrhlR DcbrA Dcrc vs WT | -3.34547 | 4.93E-49 |
| PA14_11320 | hypothetical_unclassified | PGD1652661 | NA | DrhlR DcbrA Dcrc vs WT | -1.04953 | 9.18E-04 |
| PA14_11460 | biosynthesis_of_cofactors | PGD1652683 | thiL | DrhlR DcbrA Dcrc vs WT | 1.054581 | 0.001598 |
| PA14_11790 | transport_of_small_molecule | PGD1652739 | NA | DrhlR DcbrA Dcrc vs WT | 1.505106 | 1.42E-07 |
| PA14_11810 | putative_enzymes | PGD1652741 | NA | DrhlR DcbrA Dcrc vs WT | 2.478482 | 1.29E-41 |
| PA14_12640 | hypothetical_unclassified | PGD1652863 | NA | DrhlR DcbrA Dcrc vs WT | 1.521214 | 0.002822 |
| PA14_12690 | carbon_compound_catabolis | PGD1652871 | NA | DrhlR DcbrA Dcrc vs WT | 1.234564 | 3.58E-11 |
| PA14_12740 | hypothetical_unclassified | PGD1652879 | NA | DrhlR DcbrA Dcrc vs WT | 1.657551 | 5.99E-10 |
| PA14_12940 | transport_of_small_molecule | PGD1652909 | NA | DrhlR DcbrA Dcrc vs WT | 1.132965 | 0.013342 |
| PA14_12960 | transport_of_small_molecule | PGD1652911 | NA | DrhlR DcbrA Dcrc vs WT | 2.07687 | 7.52E-05 |
| PA14_13050 | hypothetical_unclassified | PGD1652927 | NA | DrhlR DcbrA Dcrc vs WT | -1.10636 | 5.77E-04 |
| PA14_13130 | hypothetical_unclassified | PGD1652937 | NA | DrhlR DcbrA Dcrc vs WT | -1.78927 | 1.69E-19 |
| PA14_13140 | hypothetical_unclassified | PGD1652939 | NA | DrhlR DcbrA Dcrc vs WT | -1.04298 | 3.18E-06 |
| PA14_13170 | transport_of_small_molecule | PGD1652943 | NA | DrhlR DcbrA Dcrc vs WT | -3.2802 | 2.81E-40 |
| PA14_13210 | hypothetical_unclassified | PGD1652949 | NA | DrhlR DcbrA Dcrc vs WT | -3.2829 | 2.28E-47 |
| PA14_13240 | biosynthesis_of_cofactors | PGD1652955 | moaD | DrhlR DcbrA Dcrc vs WT | -1.90205 | 4.57E-10 |
| PA14_13250 | biosynthesis_of_cofactors | PGD1652957 | moaE | DrhlR DcbrA Dcrc vs WT | -1.61336 | 2.20E-12 |
| PA14_13260 | biosynthesis_of_cofactors | PGD1652959 | moaB1 | DrhlR DcbrA Dcrc vs WT | -2.58405 | 4.48E-22 |
| PA14_13280 | biosynthesis_of_cofactors | PGD1652961 | moeA1 | DrhlR DcbrA Dcrc vs WT | -2.35846 | 1.77E-13 |
| PA14_13300 | translation_posttranslational | PGD1652965 | NA | DrhlR DcbrA Dcrc vs WT | -1.09403 | 2.60E-04 |
| PA14_13330 | nucleotide_biosynthesis_and | PGD1652969 | NA | DrhlR DcbrA Dcrc vs WT | -1.1887 | 2.30E-06 |
| PA14_13330 | putative_enzymes | PGD1652969 | NA | DrhlR DcbrA Dcrc vs WT | -1.1887 | 2.30E-06 |
| PA14_13330 | secreted_factors | PGD1652969 | NA | DrhlR DcbrA Dcrc vs WT | -1.1887 | 2.30E-06 |
| PA14_13340 | transcription_RNA_processin | PGD1652971 | NA | DrhlR DcbrA Dcrc vs WT | -2.3658 | 3.90E-12 |
| PA14_13450 | membrane_proteins | PGD1652989 | NA | DrhlR DcbrA Dcrc vs WT | 1.078269 | 0.03772 |
| PA14_13460 | transcriptional_regulators | PGD1652991 | NA | DrhlR DcbrA Dcrc vs WT | 1.309437 | 0.023537 |
| PA14_13650 | carbon_compound_catabolis | PGD1653019 | NA | DrhlR DcbrA Dcrc vs WT | -1.00995 | 0.013164 |

| | | | | | | |
|---|---|---|---|---|---|---|
| PA14_13720 | hypothetical_unclassified | PGD1653031 | NA | DrhlR DcbrA Dcrc vs WT | 1.752454 | 1.99E-08 |
| PA14_13730 | two_component_regulators | PGD1653033 | narL | DrhlR DcbrA Dcrc vs WT | 1.470175 | 1.17E-11 |
| PA14_13750 | membrane_proteins | PGD1653037 | narK1 | DrhlR DcbrA Dcrc vs WT | -1.41628 | 7.46E-08 |
| PA14_13750 | transport_of_small_molecule | PGD1653037 | narK1 | DrhlR DcbrA Dcrc vs WT | -1.41628 | 7.46E-08 |
| PA14_13770 | transport_of_small_molecule | PGD1653039 | narK2 | DrhlR DcbrA Dcrc vs WT | -1.22407 | 1.53E-06 |
| PA14_13780 | energy_metabolism_CAP | PGD1653041 | narG | DrhlR DcbrA Dcrc vs WT | -1.26271 | 4.46E-08 |
| PA14_13810 | energy_metabolism_CAP | PGD1653045 | narJ | DrhlR DcbrA Dcrc vs WT | -1.18052 | 0.001309 |
| PA14_13830 | energy_metabolism_CAP | PGD1653047 | narI | DrhlR DcbrA Dcrc vs WT | -1.16296 | 6.86E-05 |
| PA14_13920 | hypothetical_unclassified | PGD1653063 | NA | DrhlR DcbrA Dcrc vs WT | 1.268085 | 0.003084 |
| PA14_14320 | hypothetical_unclassified | PGD1653121 | NA | DrhlR DcbrA Dcrc vs WT | -1.10664 | 0.049956 |
| PA14_14430 | hypothetical_unclassified | PGD1653139 | NA | DrhlR DcbrA Dcrc vs WT | -1.29701 | 4.12E-05 |
| PA14_15160 | hypothetical_unclassified | PGD1653255 | NA | DrhlR DcbrA Dcrc vs WT | 1.241418 | 7.11E-05 |
| PA14_15770 | hypothetical_unclassified | PGD1653357 | NA | DrhlR DcbrA Dcrc vs WT | 1.073475 | 3.45E-09 |
| PA14_16100 | hypothetical_unclassified | PGD1653413 | NA | DrhlR DcbrA Dcrc vs WT | -3.38797 | 1.05E-45 |
| PA14_16110 | hypothetical_unclassified | PGD1653415 | NA | DrhlR DcbrA Dcrc vs WT | -1.56938 | 8.00E-14 |
| PA14_16150 | transcription_RNA_processin | PGD1653421 | NA | DrhlR DcbrA Dcrc vs WT | 1.372768 | 1.40E-13 |
| PA14_16250 | amino_acid_biosynthesis_me | PGD1653435 | lasB | DrhlR DcbrA Dcrc vs WT | -1.84527 | 3.82E-09 |
| PA14_16250 | secreted_factors | PGD1653435 | lasB | DrhlR DcbrA Dcrc vs WT | -1.84527 | 3.82E-09 |
| PA14_16250 | translation_posttranslational | PGD1653435 | lasB | DrhlR DcbrA Dcrc vs WT | -1.84527 | 3.82E-09 |
| PA14_16300 | antibiotic_resistance_and_su | PGD1653445 | NA | DrhlR DcbrA Dcrc vs WT | -1.83475 | 0.008622 |
| PA14_16310 | membrane_proteins | PGD1653447 | NA | DrhlR DcbrA Dcrc vs WT | -2.11235 | 1.74E-09 |
| PA14_16310 | transport_of_small_molecule | PGD1653447 | NA | DrhlR DcbrA Dcrc vs WT | -2.11235 | 1.74E-09 |
| PA14_16800 | transport_of_small_molecule | PGD1653529 | NA | DrhlR DcbrA Dcrc vs WT | 2.598139 | 1.50E-55 |
| PA14_16820 | antibiotic_resistance_and_su | PGD1653531 | NA | DrhlR DcbrA Dcrc vs WT | 1.63781 | 6.00E-41 |
| PA14_16960 | amino_acid_biosynthesis_me | PGD1653553 | NA | DrhlR DcbrA Dcrc vs WT | 1.298538 | 3.17E-04 |
| PA14_17400 | central_intermediary_metab | PGD1653625 | adhC | DrhlR DcbrA Dcrc vs WT | -1.30144 | 1.13E-09 |
| PA14_17410 | putative_enzymes | PGD1653627 | NA | DrhlR DcbrA Dcrc vs WT | -1.202 | 4.11E-04 |
| PA14_17470 | cell_wall_LPS | PGD1653637 | NA | DrhlR DcbrA Dcrc vs WT | -1.11264 | 1.51E-10 |
| PA14_17480 | transcriptional_regulators | PGD1653639 | rpoS | DrhlR DcbrA Dcrc vs WT | -1.09031 | 6.16E-09 |
| PA14_17550 | putative_enzymes | PGD1653653 | NA | DrhlR DcbrA Dcrc vs WT | 1.055238 | 3.87E-10 |
| PA14_17570 | translation_posttranslational | PGD1653655 | NA | DrhlR DcbrA Dcrc vs WT | 1.436534 | 1.22E-11 |
| PA14_17580 | hypothetical_unclassified | PGD1653657 | NA | DrhlR DcbrA Dcrc vs WT | 1.542979 | 6.13E-12 |
| PA14_17960 | central_intermediary_metab | PGD1653719 | glpK | DrhlR DcbrA Dcrc vs WT | 1.474056 | 6.05E-10 |
| PA14_18100 | hypothetical_unclassified | PGD1653741 | NA | DrhlR DcbrA Dcrc vs WT | 3.000864 | 3.81E-20 |
| PA14_18120 | amino_acid_biosynthesis_me | PGD1653745 | mmsA | DrhlR DcbrA Dcrc vs WT | 2.697506 | 5.70E-110 |
| PA14_18140 | carbon_compound_catabolis | PGD1653747 | mmsB | DrhlR DcbrA Dcrc vs WT | 2.179012 | 3.54E-44 |
| PA14_18150 | fatty_acid_and_phospholipid | PGD1653749 | NA | DrhlR DcbrA Dcrc vs WT | 3.785367 | 2.41E-118 |
| PA14_18670 | adaptation_and_protection | PGD1653823 | bfrB | DrhlR DcbrA Dcrc vs WT | 1.186176 | 2.54E-05 |
| PA14_18670 | transport_of_small_molecule | PGD1653823 | bfrB | DrhlR DcbrA Dcrc vs WT | 1.186176 | 2.54E-05 |
| PA14_18720 | membrane_proteins | PGD1653833 | NA | DrhlR DcbrA Dcrc vs WT | 1.626308 | 3.93E-20 |
| PA14_18800 | transport_of_small_molecule | PGD1653845 | NA | DrhlR DcbrA Dcrc vs WT | -5.12902 | 3.96E-50 |
| PA14_18810 | hypothetical_unclassified | PGD1653847 | NA | DrhlR DcbrA Dcrc vs WT | -1.70593 | 1.41E-07 |
| PA14_18850 | carbon_compound_catabolis | PGD1653853 | NA | DrhlR DcbrA Dcrc vs WT | 1.425488 | 0.001549 |
| PA14_18850 | putative_enzymes | PGD1653853 | NA | DrhlR DcbrA Dcrc vs WT | 1.425488 | 0.001549 |
| PA14_18985 | hypothetical_unclassified | PGD1653877 | vgrG4b | DrhlR DcbrA Dcrc vs WT | -1.33248 | 7.79E-09 |
| PA14_19100 | secreted_factors | PGD1653891 | rhlA | DrhlR DcbrA Dcrc vs WT | -8.23226 | 8.88E-49 |
| PA14_19110 | secreted_factors | PGD1653893 | rhlB | DrhlR DcbrA Dcrc vs WT | -1.47373 | 9.72E-11 |
| PA14_19120 | transcriptional_regulators | PGD1653895 | rhlR | DrhlR DcbrA Dcrc vs WT | -7.07113 | 1.93E-67 |
| PA14_19130 | adaptation_and_protection | PGD1653897 | rhlI | DrhlR DcbrA Dcrc vs WT | -1.62567 | 2.16E-22 |
| PA14_19130 | biosynthesis_of_cofactors | PGD1653897 | rhlI | DrhlR DcbrA Dcrc vs WT | -1.62567 | 2.16E-22 |

| | | | | | | |
|---|---|---|---|---|---|---|
| PA14_19140 | amino_acid_biosynthesis_me | PGD1653899 | pheC | DrhlR DcbrA Dcrc vs WT | -1.89855 | 2.89E-35 |
| PA14_19270 | transport_of_small_molecule | PGD1653915 | NA | DrhlR DcbrA Dcrc vs WT | -1.30381 | 0.002237 |
| PA14_19380 | transcriptional_regulators | PGD1653933 | NA | DrhlR DcbrA Dcrc vs WT | 1.081349 | 7.09E-06 |
| PA14_19510 | transport_of_small_molecule | PGD1653953 | NA | DrhlR DcbrA Dcrc vs WT | 1.34844 | 0.007947 |
| PA14_19520 | transport_of_small_molecule | PGD1653955 | NA | DrhlR DcbrA Dcrc vs WT | 2.044429 | 3.25E-04 |
| PA14_19560 | central_intermediary_metab | PGD1653961 | ssuD | DrhlR DcbrA Dcrc vs WT | 1.459942 | 0.011051 |
| PA14_19580 | transport_of_small_molecule | PGD1653965 | ssuB | DrhlR DcbrA Dcrc vs WT | 2.260762 | 0.001769 |
| PA14_19590 | central_intermediary_metab | PGD1653967 | NA | DrhlR DcbrA Dcrc vs WT | 1.745507 | 0.007528 |
| PA14_19690 | membrane_proteins | PGD1653987 | NA | DrhlR DcbrA Dcrc vs WT | 1.864246 | 0.004795 |
| PA14_19690 | protein_secretion_export | PGD1653987 | NA | DrhlR DcbrA Dcrc vs WT | 1.864246 | 0.004795 |
| PA14_19700 | carbon_compound_catabolis | PGD1653989 | NA | DrhlR DcbrA Dcrc vs WT | 1.089926 | 1.10E-06 |
| PA14_19990 | transcriptional_regulators | PGD1654031 | NA | DrhlR DcbrA Dcrc vs WT | 2.005978 | 4.71E-04 |
| PA14_20000 | membrane_proteins | PGD1654033 | NA | DrhlR DcbrA Dcrc vs WT | 3.609527 | 4.15E-12 |
| PA14_20010 | transport_of_small_molecule | PGD1654035 | hasR | DrhlR DcbrA Dcrc vs WT | 3.696597 | 6.99E-11 |
| PA14_20020 | transport_of_small_molecule | PGD1654037 | hasAp | DrhlR DcbrA Dcrc vs WT | 4.561911 | 6.11E-12 |
| PA14_20030 | protein_secretion_export | PGD1654039 | hasD | DrhlR DcbrA Dcrc vs WT | 4.066181 | 1.10E-09 |
| PA14_20040 | protein_secretion_export | PGD1654041 | hasE | DrhlR DcbrA Dcrc vs WT | 4.291718 | 3.10E-10 |
| PA14_20050 | protein_secretion_export | PGD1654043 | NA | DrhlR DcbrA Dcrc vs WT | 3.842668 | 6.91E-15 |
| PA14_20070 | membrane_proteins | PGD1654047 | NA | DrhlR DcbrA Dcrc vs WT | 1.006982 | 0.011932 |
| PA14_20230 | energy_metabolism_CAP | PGD1654071 | nosR | DrhlR DcbrA Dcrc vs WT | 1.236931 | 2.61E-05 |
| PA14_20230 | membrane_proteins | PGD1654071 | nosR | DrhlR DcbrA Dcrc vs WT | 1.236931 | 2.61E-05 |
| PA14_20230 | transcriptional_regulators | PGD1654071 | nosR | DrhlR DcbrA Dcrc vs WT | 1.236931 | 2.61E-05 |
| PA14_20530 | hypothetical_unclassified | PGD1654125 | NA | DrhlR DcbrA Dcrc vs WT | 1.140506 | 0.012613 |
| PA14_20610 | motility_and_attachment | PGD1654139 | lecB | DrhlR DcbrA Dcrc vs WT | -7.5796 | 1.31E-34 |
| PA14_20890 | cell_wall_LPS | PGD1654187 | rfaD | DrhlR DcbrA Dcrc vs WT | 1.335512 | 4.62E-07 |
| PA14_20900 | membrane_proteins | PGD1654189 | NA | DrhlR DcbrA Dcrc vs WT | -2.33248 | 7.40E-16 |
| PA14_20920 | hypothetical_unclassified | PGD1654191 | NA | DrhlR DcbrA Dcrc vs WT | -1.95816 | 5.89E-23 |
| PA14_20940 | fatty_acid_and_phospholipid | PGD1654193 | NA | DrhlR DcbrA Dcrc vs WT | -3.48253 | 4.62E-16 |
| PA14_20950 | fatty_acid_and_phospholipid | PGD1654195 | fabH2 | DrhlR DcbrA Dcrc vs WT | -3.84477 | 7.15E-28 |
| PA14_20960 | putative_enzymes | PGD1654197 | NA | DrhlR DcbrA Dcrc vs WT | -2.30792 | 5.02E-09 |
| PA14_20970 | adaptation_and_protection | PGD1654199 | cyp23 | DrhlR DcbrA Dcrc vs WT | -2.69057 | 1.57E-16 |
| PA14_20980 | putative_enzymes | PGD1654201 | NA | DrhlR DcbrA Dcrc vs WT | -2.19308 | 1.01E-14 |
| PA14_21000 | hypothetical_unclassified | PGD1654203 | NA | DrhlR DcbrA Dcrc vs WT | -2.50278 | 3.84E-17 |
| PA14_21010 | energy_metabolism_CAP | PGD1654205 | NA | DrhlR DcbrA Dcrc vs WT | -1.69446 | 2.11E-07 |
| PA14_21020 | biosynthesis_of_cofactors | PGD1654207 | NA | DrhlR DcbrA Dcrc vs WT | -2.28129 | 1.40E-25 |
| PA14_21030 | cell_wall_LPS | PGD1654209 | NA | DrhlR DcbrA Dcrc vs WT | -1.51256 | 1.84E-18 |
| PA14_21030 | translation_posttranslational | PGD1654209 | NA | DrhlR DcbrA Dcrc vs WT | -1.51256 | 1.84E-18 |
| PA14_21130 | membrane_proteins | PGD1654227 | NA | DrhlR DcbrA Dcrc vs WT | 1.290574 | 7.92E-11 |
| PA14_21220 | hypothetical_unclassified | PGD1654243 | NA | DrhlR DcbrA Dcrc vs WT | 1.197377 | 1.49E-04 |
| PA14_21300 | transport_of_small_molecule | PGD1654257 | NA | DrhlR DcbrA Dcrc vs WT | -1.02795 | 2.82E-04 |
| PA14_21470 | hypothetical_unclassified | PGD1654279 | NA | DrhlR DcbrA Dcrc vs WT | -1.44965 | 5.35E-09 |
| PA14_21570 | hypothetical_unclassified | PGD1654297 | NA | DrhlR DcbrA Dcrc vs WT | 2.222984 | 4.66E-04 |
| PA14_21580 | hypothetical_unclassified | PGD1654299 | NA | DrhlR DcbrA Dcrc vs WT | 1.954238 | 0.006508 |
| PA14_21590 | hypothetical_unclassified | PGD1654301 | NA | DrhlR DcbrA Dcrc vs WT | 3.196718 | 6.49E-06 |
| PA14_21600 | membrane_proteins | PGD1654303 | NA | DrhlR DcbrA Dcrc vs WT | 2.392588 | 0.0012 |
| PA14_21620 | transport_of_small_molecule | PGD1654307 | oprP | DrhlR DcbrA Dcrc vs WT | -1.02198 | 0.019156 |
| PA14_21630 | membrane_proteins | PGD1654309 | NA | DrhlR DcbrA Dcrc vs WT | 2.129148 | 3.22E-17 |
| PA14_21640 | biosynthesis_of_cofactors | PGD1654311 | NA | DrhlR DcbrA Dcrc vs WT | 1.205553 | 3.59E-07 |
| PA14_21640 | putative_enzymes | PGD1654311 | NA | DrhlR DcbrA Dcrc vs WT | 1.205553 | 3.59E-07 |
| PA14_21850 | transcriptional_regulators | PGD1654351 | NA | DrhlR DcbrA Dcrc vs WT | 1.198254 | 4.22E-07 |

| | | | | | | |
|---|---|---|---|---|---|---|
| PA14_22010 | cell_division | PGD1654381 | minE | DrhlR DcbrA Dcrc vs WT | -1.17068 | 5.64E-04 |
| PA14_22220 | hypothetical_unclassified | PGD1654415 | NA | DrhlR DcbrA Dcrc vs WT | -1.34944 | 0.041431 |
| PA14_22250 | hypothetical_unclassified | PGD1654421 | NA | DrhlR DcbrA Dcrc vs WT | -1.78634 | 0.035353 |
| PA14_22320 | membrane_proteins | PGD1654433 | NA | DrhlR DcbrA Dcrc vs WT | -2.0965 | 4.87E-05 |
| PA14_22330 | transport_of_small_molecule | PGD1654435 | NA | DrhlR DcbrA Dcrc vs WT | -1.30654 | 5.25E-07 |
| PA14_22420 | hypothetical_unclassified | PGD1654449 | NA | DrhlR DcbrA Dcrc vs WT | -1.073 | 0.004632 |
| PA14_22700 | transport_of_small_molecule | PGD1654501 | NA | DrhlR DcbrA Dcrc vs WT | 1.031214 | 0.039103 |
| PA14_22880 | hypothetical_unclassified | PGD1654529 | NA | DrhlR DcbrA Dcrc vs WT | 1.742503 | 2.97E-05 |
| PA14_22980 | transport_of_small_molecule | PGD1654541 | NA | DrhlR DcbrA Dcrc vs WT | 1.166967 | 2.02E-11 |
| PA14_22990 | transport_of_small_molecule | PGD1654543 | NA | DrhlR DcbrA Dcrc vs WT | 2.317399 | 9.85E-33 |
| PA14_23000 | transport_of_small_molecule | PGD1654545 | NA | DrhlR DcbrA Dcrc vs WT | 2.040076 | 8.52E-15 |
| PA14_23010 | transport_of_small_molecule | PGD1654547 | gltK | DrhlR DcbrA Dcrc vs WT | 2.629428 | 7.19E-39 |
| PA14_23030 | transport_of_small_molecule | PGD1654549 | oprB | DrhlR DcbrA Dcrc vs WT | 2.425648 | 1.80E-49 |
| PA14_23170 | amino_acid_biosynthesis_me | PGD1654571 | NA | DrhlR DcbrA Dcrc vs WT | 1.393177 | 0.026147 |
| PA14_23680 | chaperones_heat_shock | PGD1654661 | ibpA | DrhlR DcbrA Dcrc vs WT | 2.607165 | 4.61E-11 |
| PA14_23980 | protein_secretion_export | PGD1654705 | xcpP | DrhlR DcbrA Dcrc vs WT | -1.11835 | 3.82E-05 |
| PA14_24170 | fatty_acid_and_phospholipid | PGD1654735 | fadH1 | DrhlR DcbrA Dcrc vs WT | 1.014905 | 2.93E-04 |
| PA14_24170 | putative_enzymes | PGD1654735 | fadH1 | DrhlR DcbrA Dcrc vs WT | 1.014905 | 2.93E-04 |
| PA14_24180 | hypothetical_unclassified | PGD1654737 | NA | DrhlR DcbrA Dcrc vs WT | 1.186059 | 3.96E-17 |
| PA14_24290 | amino_acid_biosynthesis_me | PGD1654755 | gbt | DrhlR DcbrA Dcrc vs WT | -1.08659 | 1.53E-06 |
| PA14_24480 | cell_wall_LPS | PGD1654787 | pelA | DrhlR DcbrA Dcrc vs WT | -1.26568 | 2.28E-15 |
| PA14_24490 | cell_wall_LPS | PGD1654789 | pelB | DrhlR DcbrA Dcrc vs WT | -1.31318 | 6.67E-13 |
| PA14_24500 | cell_wall_LPS | PGD1654791 | pelC | DrhlR DcbrA Dcrc vs WT | -1.20117 | 0.002301 |
| PA14_24500 | fatty_acid_and_phospholipid | PGD1654791 | pelC | DrhlR DcbrA Dcrc vs WT | -1.20117 | 0.002301 |
| PA14_24510 | cell_wall_LPS | PGD1654793 | pelD | DrhlR DcbrA Dcrc vs WT | -1.41743 | 1.44E-05 |
| PA14_24530 | cell_wall_LPS | PGD1654795 | pelE | DrhlR DcbrA Dcrc vs WT | -1.00001 | 0.033774 |
| PA14_24560 | cell_wall_LPS | PGD1654799 | pelG | DrhlR DcbrA Dcrc vs WT | -1.76018 | 4.45E-11 |
| PA14_24560 | secreted_factors | PGD1654799 | pelG | DrhlR DcbrA Dcrc vs WT | -1.76018 | 4.45E-11 |
| PA14_24600 | amino_acid_biosynthesis_me | PGD1654807 | NA | DrhlR DcbrA Dcrc vs WT | 1.84667 | 2.53E-16 |
| PA14_24630 | hypothetical_unclassified | PGD1654813 | NA | DrhlR DcbrA Dcrc vs WT | -1.0896 | 9.61E-07 |
| PA14_24650 | translation_posttranslational | PGD1654817 | rmf | DrhlR DcbrA Dcrc vs WT | -1.2647 | 3.31E-07 |
| PA14_24830 | putative_enzymes | PGD1654847 | NA | DrhlR DcbrA Dcrc vs WT | -1.07568 | 0.018571 |
| PA14_24830 | transport_of_small_molecule | PGD1654847 | NA | DrhlR DcbrA Dcrc vs WT | -1.07568 | 0.018571 |
| PA14_24860 | energy_metabolism_CAP | PGD1654853 | snr1 | DrhlR DcbrA Dcrc vs WT | -1.17822 | 8.23E-09 |
| PA14_24940 | energy_metabolism_CAP | PGD1654867 | NA | DrhlR DcbrA Dcrc vs WT | 1.045712 | 8.00E-08 |
| PA14_24950 | carbon_compound_catabolis | PGD1654869 | NA | DrhlR DcbrA Dcrc vs WT | 1.086505 | 1.84E-04 |
| PA14_24950 | energy_metabolism_CAP | PGD1654869 | NA | DrhlR DcbrA Dcrc vs WT | 1.086505 | 1.84E-04 |
| PA14_25080 | amino_acid_biosynthesis_me | PGD1654891 | fadB | DrhlR DcbrA Dcrc vs WT | 1.326172 | 9.99E-20 |
| PA14_25080 | fatty_acid_and_phospholipid | PGD1654891 | fadB | DrhlR DcbrA Dcrc vs WT | 1.326172 | 9.99E-20 |
| PA14_25180 | transcriptional_regulators | PGD1654907 | psrA | DrhlR DcbrA Dcrc vs WT | 1.038352 | 2.53E-07 |
| PA14_25840 | energy_metabolism_CAP | PGD1655015 | NA | DrhlR DcbrA Dcrc vs WT | 1.201014 | 6.19E-22 |
| PA14_25980 | amino_acid_biosynthesis_me | PGD1655035 | aroF | DrhlR DcbrA Dcrc vs WT | 1.12935 | 0.003684 |
| PA14_26190 | hypothetical_unclassified | PGD1655067 | NA | DrhlR DcbrA Dcrc vs WT | -1.50954 | 4.41E-10 |
| PA14_26300 | hypothetical_unclassified | PGD1655085 | NA | DrhlR DcbrA Dcrc vs WT | -1.46664 | 0.037292 |
| PA14_26340 | membrane_proteins | PGD1655091 | NA | DrhlR DcbrA Dcrc vs WT | 2.199785 | 0.002015 |
| PA14_26700 | fatty_acid_and_phospholipid | PGD1655145 | NA | DrhlR DcbrA Dcrc vs WT | -1.15767 | 4.54E-05 |
| PA14_26700 | putative_enzymes | PGD1655145 | NA | DrhlR DcbrA Dcrc vs WT | -1.15767 | 4.54E-05 |
| PA14_26730 | putative_enzymes | PGD1655149 | NA | DrhlR DcbrA Dcrc vs WT | -1.06674 | 0.007947 |
| PA14_26920 | hypothetical_unclassified | PGD1655175 | NA | DrhlR DcbrA Dcrc vs WT | 1.258116 | 2.21E-07 |
| PA14_27000 | adaptation_and_protection | PGD1655189 | NA | DrhlR DcbrA Dcrc vs WT | 1.041819 | 2.73E-10 |

| | | | | | | |
|---|---|---|---|---|---|---|
| PA14_27000 | chemotaxis | PGD1655189 | NA | DrhlR DcbrA Dcrc vs WT | 1.041819 | 2.73E-10 |
| PA14_27090 | protein_secretion_export | PGD1655197 | lipH | DrhlR DcbrA Dcrc vs WT | -2.02333 | 0.012786 |
| PA14_27090 | secreted_factors | PGD1655197 | lipH | DrhlR DcbrA Dcrc vs WT | -2.02333 | 0.012786 |
| PA14_27360 | fatty_acid_and_phospholipid | PGD1655241 | NA | DrhlR DcbrA Dcrc vs WT | 1.021445 | 3.66E-05 |
| PA14_27360 | putative_enzymes | PGD1655241 | NA | DrhlR DcbrA Dcrc vs WT | 1.021445 | 3.66E-05 |
| PA14_27370 | transcription_RNA_processin | PGD1655243 | NA | DrhlR DcbrA Dcrc vs WT | 1.075537 | 1.21E-04 |
| PA14_27400 | transcriptional_regulators | PGD1655247 | NA | DrhlR DcbrA Dcrc vs WT | -1.10772 | 0.028315 |
| PA14_27650 | hypothetical_unclassified | PGD1655295 | NA | DrhlR DcbrA Dcrc vs WT | -1.20241 | 7.54E-06 |
| PA14_27660 | hypothetical_unclassified | PGD1655297 | NA | DrhlR DcbrA Dcrc vs WT | -1.06162 | 5.09E-07 |
| PA14_27740 | hypothetical_unclassified | PGD1655313 | NA | DrhlR DcbrA Dcrc vs WT | 1.467174 | 2.29E-08 |
| PA14_27840 | transport_of_small_molecule | PGD1655327 | NA | DrhlR DcbrA Dcrc vs WT | -1.08432 | 0.003379 |
| PA14_28100 | hypothetical_unclassified | PGD1655375 | NA | DrhlR DcbrA Dcrc vs WT | 1.276689 | 0.003378 |
| PA14_28110 | hypothetical_unclassified | PGD1655377 | NA | DrhlR DcbrA Dcrc vs WT | 1.72596 | 0.001181 |
| PA14_28250 | secreted_factors | PGD1655403 | NA | DrhlR DcbrA Dcrc vs WT | -4.64346 | 1.39E-47 |
| PA14_28260 | hypothetical_unclassified | PGD1655405 | NA | DrhlR DcbrA Dcrc vs WT | -1.93911 | 3.76E-14 |
| PA14_28290 | DNA_replication_recombinat | PGD1655409 | NA | DrhlR DcbrA Dcrc vs WT | -1.05625 | 0.006054 |
| PA14_28360 | hypothetical_unclassified | PGD1655423 | NA | DrhlR DcbrA Dcrc vs WT | -4.53365 | 5.38E-31 |
| PA14_28370 | membrane_proteins | PGD1655425 | NA | DrhlR DcbrA Dcrc vs WT | -2.34808 | 4.58E-08 |
| PA14_28380 | hypothetical_unclassified | PGD1655427 | NA | DrhlR DcbrA Dcrc vs WT | -1.3759 | 3.41E-06 |
| PA14_28390 | membrane_proteins | PGD1655429 | NA | DrhlR DcbrA Dcrc vs WT | 2.132763 | 8.60E-08 |
| PA14_28410 | hypothetical_unclassified | PGD1655433 | NA | DrhlR DcbrA Dcrc vs WT | -2.93842 | 6.04E-08 |
| PA14_28430 | membrane_proteins | PGD1655437 | NA | DrhlR DcbrA Dcrc vs WT | -1.44217 | 1.15E-05 |
| PA14_28500 | hypothetical_unclassified | PGD1655449 | NA | DrhlR DcbrA Dcrc vs WT | 2.022709 | 1.49E-11 |
| PA14_28620 | membrane_proteins | PGD1655471 | NA | DrhlR DcbrA Dcrc vs WT | -1.03848 | 1.03E-07 |
| PA14_28930 | hypothetical_unclassified | PGD1655525 | NA | DrhlR DcbrA Dcrc vs WT | 1.068817 | 0.011212 |
| PA14_28970 | hypothetical_unclassified | PGD1655533 | NA | DrhlR DcbrA Dcrc vs WT | 1.418415 | 0.009216 |
| PA14_28980 | membrane_proteins | PGD1655535 | NA | DrhlR DcbrA Dcrc vs WT | 1.170323 | 0.008589 |
| PA14_28980 | transcriptional_regulators | PGD1655535 | NA | DrhlR DcbrA Dcrc vs WT | 1.170323 | 0.008589 |
| PA14_29420 | putative_enzymes | PGD1655612 | NA | DrhlR DcbrA Dcrc vs WT | -1.51 | 5.43E-12 |
| PA14_29510 | protein_secretion_export | PGD1655626 | NA | DrhlR DcbrA Dcrc vs WT | -1.16705 | 0.036196 |
| PA14_29620 | transcriptional_regulators | PGD1655646 | NA | DrhlR DcbrA Dcrc vs WT | 2.094377 | 4.37E-15 |
| PA14_29640 | energy_metabolism_CAP | PGD1655648 | fhp | DrhlR DcbrA Dcrc vs WT | 2.52188 | 2.75E-08 |
| PA14_29650 | hypothetical_unclassified | PGD1655650 | NA | DrhlR DcbrA Dcrc vs WT | 2.680904 | 6.97E-08 |
| PA14_29660 | membrane_proteins | PGD1655652 | NA | DrhlR DcbrA Dcrc vs WT | 2.098898 | 7.85E-06 |
| PA14_29760 | chemotaxis | PGD1655668 | NA | DrhlR DcbrA Dcrc vs WT | 1.797618 | 9.08E-36 |
| PA14_30100 | hypothetical_unclassified | PGD1655716 | NA | DrhlR DcbrA Dcrc vs WT | 1.127385 | 3.84E-11 |
| PA14_30400 | energy_metabolism_CAP | PGD1655766 | NA | DrhlR DcbrA Dcrc vs WT | 1.010446 | 8.88E-05 |
| PA14_30500 | putative_enzymes | PGD1655784 | NA | DrhlR DcbrA Dcrc vs WT | 1.03851 | 0.022366 |
| PA14_30560 | translation_posttranslational | PGD1655792 | NA | DrhlR DcbrA Dcrc vs WT | -1.79973 | 7.95E-13 |
| PA14_30570 | transport_of_small_molecule | PGD1655794 | NA | DrhlR DcbrA Dcrc vs WT | -1.3276 | 5.94E-13 |
| PA14_30620 | transcriptional_regulators | PGD1655802 | NA | DrhlR DcbrA Dcrc vs WT | -2.16655 | 1.12E-41 |
| PA14_30630 | biosynthesis_of_cofactors | PGD1655804 | pqsH | DrhlR DcbrA Dcrc vs WT | -1.09538 | 6.18E-08 |
| PA14_30860 | relative_phage_transposon | PGD1655846 | NA | DrhlR DcbrA Dcrc vs WT | -2.09368 | 0.032067 |
| PA14_31270 | hypothetical_unclassified | PGD1655918 | NA | DrhlR DcbrA Dcrc vs WT | -1.70007 | 1.26E-12 |
| PA14_31280 | relative_phage_transposon | PGD1655920 | NA | DrhlR DcbrA Dcrc vs WT | -1.93719 | 3.22E-15 |
| PA14_31290 | adaptation_and_protection | PGD1655922 | pa1L | DrhlR DcbrA Dcrc vs WT | -6.98567 | 2.75E-45 |
| PA14_31290 | cell_wall_LPS | PGD1655922 | pa1L | DrhlR DcbrA Dcrc vs WT | -6.98567 | 2.75E-45 |
| PA14_31290 | motility_and_attachment | PGD1655922 | pa1L | DrhlR DcbrA Dcrc vs WT | -6.98567 | 2.75E-45 |
| PA14_31300 | hypothetical_unclassified | PGD1655924 | NA | DrhlR DcbrA Dcrc vs WT | -1.83437 | 2.60E-13 |
| PA14_31340 | hypothetical_unclassified | PGD1655930 | NA | DrhlR DcbrA Dcrc vs WT | -1.35185 | 0.002055 |

| | | | | | | |
|---|---|---|---|---|---|---|
| PA14_31350 | energy_metabolism_CAP | PGD1655932 | NA | DrhlR DcbrA Dcrc vs WT | -1.32546 | 2.27E-13 |
| PA14_31360 | hypothetical_unclassified | PGD1655934 | NA | DrhlR DcbrA Dcrc vs WT | -2.24158 | 5.09E-30 |
| PA14_31370 | putative_enzymes | PGD1655936 | NA | DrhlR DcbrA Dcrc vs WT | -2.27905 | 1.82E-43 |
| PA14_31420 | hypothetical_unclassified | PGD1655944 | NA | DrhlR DcbrA Dcrc vs WT | -1.33823 | 2.09E-07 |
| PA14_31450 | hypothetical_unclassified | PGD1655950 | NA | DrhlR DcbrA Dcrc vs WT | -1.96695 | 4.06E-16 |
| PA14_31470 | fatty_acid_and_phospholipid | PGD1655954 | NA | DrhlR DcbrA Dcrc vs WT | 1.294519 | 4.89E-21 |
| PA14_31480 | transcriptional_regulators | PGD1655956 | NA | DrhlR DcbrA Dcrc vs WT | 1.166902 | 8.19E-12 |
| PA14_31500 | fatty_acid_and_phospholipid | PGD1655958 | NA | DrhlR DcbrA Dcrc vs WT | 1.549362 | 2.69E-27 |
| PA14_31500 | putative_enzymes | PGD1655958 | NA | DrhlR DcbrA Dcrc vs WT | 1.549362 | 2.69E-27 |
| PA14_31510 | biosynthesis_of_cofactors | PGD1655960 | NA | DrhlR DcbrA Dcrc vs WT | 2.267315 | 1.06E-21 |
| PA14_31530 | fatty_acid_and_phospholipid | PGD1655962 | NA | DrhlR DcbrA Dcrc vs WT | 2.211393 | 3.62E-19 |
| PA14_31530 | putative_enzymes | PGD1655962 | NA | DrhlR DcbrA Dcrc vs WT | 2.211393 | 3.62E-19 |
| PA14_31540 | energy_metabolism_CAP | PGD1655964 | NA | DrhlR DcbrA Dcrc vs WT | 2.007479 | 8.46E-22 |
| PA14_31730 | putative_enzymes | PGD1655990 | NA | DrhlR DcbrA Dcrc vs WT | 1.100338 | 2.23E-05 |
| PA14_31820 | amino_acid_biosynthesis_me | PGD1656006 | NA | DrhlR DcbrA Dcrc vs WT | 1.812142 | 3.46E-06 |
| PA14_32060 | transcriptional_regulators | PGD1656032 | xylS | DrhlR DcbrA Dcrc vs WT | -1.98047 | 8.35E-09 |
| PA14_32140 | energy_metabolism_CAP | PGD1656042 | antC | DrhlR DcbrA Dcrc vs WT | -6.38596 | 3.78E-10 |
| PA14_32150 | biosynthesis_of_cofactors | PGD1656044 | antB | DrhlR DcbrA Dcrc vs WT | -3.60121 | 4.34E-05 |
| PA14_32160 | carbon_compound_catabolis | PGD1656046 | antA | DrhlR DcbrA Dcrc vs WT | -3.53502 | 1.36E-20 |
| PA14_32190 | transcriptional_regulators | PGD1656048 | NA | DrhlR DcbrA Dcrc vs WT | -3.25077 | 2.74E-33 |
| PA14_32220 | carbon_compound_catabolis | PGD1656052 | catB | DrhlR DcbrA Dcrc vs WT | -4.25375 | 5.02E-11 |
| PA14_32230 | carbon_compound_catabolis | PGD1656054 | catC | DrhlR DcbrA Dcrc vs WT | -4.72164 | 8.17E-13 |
| PA14_32240 | carbon_compound_catabolis | PGD1656056 | catA | DrhlR DcbrA Dcrc vs WT | -2.58439 | 2.42E-12 |
| PA14_32250 | hypothetical_unclassified | PGD1656058 | NA | DrhlR DcbrA Dcrc vs WT | -1.4329 | 0.015355 |
| PA14_32380 | antibiotic_resistance_and_su | PGD1656080 | oprN | DrhlR DcbrA Dcrc vs WT | 1.105232 | 0.025102 |
| PA14_32380 | membrane_proteins | PGD1656080 | oprN | DrhlR DcbrA Dcrc vs WT | 1.105232 | 0.025102 |
| PA14_32380 | transport_of_small_molecule | PGD1656080 | oprN | DrhlR DcbrA Dcrc vs WT | 1.105232 | 0.025102 |
| PA14_32580 | two_component_regulators | PGD1656112 | NA | DrhlR DcbrA Dcrc vs WT | 1.269725 | 1.31E-06 |
| PA14_32720 | membrane_proteins | PGD1656136 | NA | DrhlR DcbrA Dcrc vs WT | 1.317118 | 0.001346 |
| PA14_32750 | hypothetical_unclassified | PGD1656140 | NA | DrhlR DcbrA Dcrc vs WT | -1.30413 | 0.027538 |
| PA14_32860 | hypothetical_unclassified | PGD1656158 | NA | DrhlR DcbrA Dcrc vs WT | 1.425631 | 3.07E-07 |
| PA14_33000 | amino_acid_biosynthesis_me | PGD1656176 | gcvP2 | DrhlR DcbrA Dcrc vs WT | 1.253164 | 4.26E-07 |
| PA14_33010 | amino_acid_biosynthesis_me | PGD1656178 | glyA2 | DrhlR DcbrA Dcrc vs WT | 1.347819 | 2.44E-08 |
| PA14_33050 | hypothetical_unclassified | PGD1656184 | NA | DrhlR DcbrA Dcrc vs WT | -1.24297 | 3.46E-07 |
| PA14_33060 | hypothetical_unclassified | PGD1656186 | NA | DrhlR DcbrA Dcrc vs WT | -1.08255 | 0.002957 |
| PA14_33120 | hypothetical_unclassified | PGD1656194 | NA | DrhlR DcbrA Dcrc vs WT | 1.219451 | 2.55E-09 |
| PA14_33270 | adaptation_and_protection | PGD1656218 | pvdG | DrhlR DcbrA Dcrc vs WT | 1.104867 | 5.17E-05 |
| PA14_33280 | adaptation_and_protection | PGD1656220 | pvdL | DrhlR DcbrA Dcrc vs WT | 1.442491 | 8.19E-07 |
| PA14_33420 | putative_enzymes | PGD1656244 | NA | DrhlR DcbrA Dcrc vs WT | -1.79395 | 0.002375 |
| PA14_33460 | membrane_proteins | PGD1656252 | NA | DrhlR DcbrA Dcrc vs WT | 1.013773 | 1.45E-04 |
| PA14_33500 | adaptation_and_protection | PGD1656256 | pvdH | DrhlR DcbrA Dcrc vs WT | 2.118551 | 8.48E-11 |
| PA14_33500 | transport_of_small_molecule | PGD1656256 | pvdH | DrhlR DcbrA Dcrc vs WT | 2.118551 | 8.48E-11 |
| PA14_33520 | adaptation_and_protection | PGD1656260 | NA | DrhlR DcbrA Dcrc vs WT | 1.025536 | 5.90E-04 |
| PA14_33520 | biosynthesis_of_cofactors | PGD1656260 | NA | DrhlR DcbrA Dcrc vs WT | 1.025536 | 5.90E-04 |
| PA14_33520 | putative_enzymes | PGD1656260 | NA | DrhlR DcbrA Dcrc vs WT | 1.025536 | 5.90E-04 |
| PA14_33680 | transport_of_small_molecule | PGD1656284 | fpvA | DrhlR DcbrA Dcrc vs WT | 1.398907 | 1.47E-06 |
| PA14_33690 | adaptation_and_protection | PGD1656286 | pvdE | DrhlR DcbrA Dcrc vs WT | 1.26899 | 8.90E-05 |
| PA14_33690 | membrane_proteins | PGD1656286 | pvdE | DrhlR DcbrA Dcrc vs WT | 1.26899 | 8.90E-05 |
| PA14_33690 | transport_of_small_molecule | PGD1656286 | pvdE | DrhlR DcbrA Dcrc vs WT | 1.26899 | 8.90E-05 |
| PA14_33710 | adaptation_and_protection | PGD1656290 | pvdO | DrhlR DcbrA Dcrc vs WT | 1.336148 | 1.87E-04 |

| | | | | | | |
|---|---|---|---|---|---|---|
| PA14_33720 | adaptation_and_protection | PGD1656292 | pvdN | DrhlR DcbrA Dcrc vs WT | 1.19384 | 5.72E-04 |
| PA14_33720 | transport_of_small_molecule | PGD1656292 | pvdN | DrhlR DcbrA Dcrc vs WT | 1.19384 | 5.72E-04 |
| PA14_33730 | central_intermediary_metab | PGD1656294 | NA | DrhlR DcbrA Dcrc vs WT | 1.374175 | 6.64E-05 |
| PA14_33740 | adaptation_and_protection | PGD1656296 | pvdP | DrhlR DcbrA Dcrc vs WT | 1.437415 | 8.81E-06 |
| PA14_33750 | transport_of_small_molecule | PGD1656298 | NA | DrhlR DcbrA Dcrc vs WT | 1.969636 | 6.67E-13 |
| PA14_33760 | membrane_proteins | PGD1656300 | NA | DrhlR DcbrA Dcrc vs WT | 1.649465 | 1.45E-10 |
| PA14_33760 | transport_of_small_molecule | PGD1656300 | NA | DrhlR DcbrA Dcrc vs WT | 1.649465 | 1.45E-10 |
| PA14_33770 | protein_secretion_export | PGD1656302 | NA | DrhlR DcbrA Dcrc vs WT | 1.223006 | 1.00E-05 |
| PA14_33810 | biosynthesis_of_cofactors | PGD1656308 | pvdA | DrhlR DcbrA Dcrc vs WT | 1.464103 | 3.52E-06 |
| PA14_33820 | adaptation_and_protection | PGD1656310 | pvdQ | DrhlR DcbrA Dcrc vs WT | 1.578037 | 4.50E-07 |
| PA14_33820 | antibiotic_resistance_and_su | PGD1656310 | pvdQ | DrhlR DcbrA Dcrc vs WT | 1.578037 | 4.50E-07 |
| PA14_33910 | transport_of_small_molecule | PGD1656326 | NA | DrhlR DcbrA Dcrc vs WT | 3.126016 | 9.36E-20 |
| PA14_34340 | carbon_compound_catabolis | PGD1656394 | mtlZ | DrhlR DcbrA Dcrc vs WT | 1.806715 | 0.00135 |
| PA14_34350 | carbon_compound_catabolis | PGD1656396 | mtlY | DrhlR DcbrA Dcrc vs WT | 2.316109 | 3.85E-05 |
| PA14_34360 | carbon_compound_catabolis | PGD1656398 | mtlD | DrhlR DcbrA Dcrc vs WT | 2.254874 | 1.07E-07 |
| PA14_34370 | transport_of_small_molecule | PGD1656400 | NA | DrhlR DcbrA Dcrc vs WT | 2.721723 | 1.56E-15 |
| PA14_34420 | transport_of_small_molecule | PGD1656406 | NA | DrhlR DcbrA Dcrc vs WT | 2.386497 | 2.74E-22 |
| PA14_34600 | carbon_compound_catabolis | PGD1656428 | NA | DrhlR DcbrA Dcrc vs WT | 1.138749 | 4.45E-06 |
| PA14_34870 | carbon_compound_catabolis | PGD1656474 | chiC | DrhlR DcbrA Dcrc vs WT | -7.66387 | 1.36E-58 |
| PA14_34880 | transcriptional_regulators | PGD1656476 | NA | DrhlR DcbrA Dcrc vs WT | -1.18813 | 0.001717 |
| PA14_34900 | energy_metabolism_CAP | PGD1656478 | NA | DrhlR DcbrA Dcrc vs WT | -1.51705 | 7.00E-08 |
| PA14_34900 | putative_enzymes | PGD1656478 | NA | DrhlR DcbrA Dcrc vs WT | -1.51705 | 7.00E-08 |
| PA14_35160 | hypothetical_unclassified | PGD1656520 | NA | DrhlR DcbrA Dcrc vs WT | -2.35496 | 1.50E-13 |
| PA14_35300 | hypothetical_unclassified | PGD1656540 | NA | DrhlR DcbrA Dcrc vs WT | 1.120742 | 2.14E-05 |
| PA14_35340 | carbon_compound_catabolis | PGD1656546 | NA | DrhlR DcbrA Dcrc vs WT | 1.293424 | 5.42E-04 |
| PA14_35360 | carbon_compound_catabolis | PGD1656548 | NA | DrhlR DcbrA Dcrc vs WT | 2.031267 | 6.18E-06 |
| PA14_35440 | amino_acid_biosynthesis_me | PGD1656562 | ansA | DrhlR DcbrA Dcrc vs WT | 1.086669 | 0.022346 |
| PA14_35490 | amino_acid_biosynthesis_me | PGD1656568 | lpdV | DrhlR DcbrA Dcrc vs WT | 2.446811 | 9.01E-55 |
| PA14_35490 | energy_metabolism_CAP | PGD1656568 | lpdV | DrhlR DcbrA Dcrc vs WT | 2.446811 | 9.01E-55 |
| PA14_35500 | amino_acid_biosynthesis_me | PGD1656570 | bkdB | DrhlR DcbrA Dcrc vs WT | 3.369532 | 5.09E-140 |
| PA14_35520 | amino_acid_biosynthesis_me | PGD1656572 | bkdA2 | DrhlR DcbrA Dcrc vs WT | 3.0717 | 1.11E-87 |
| PA14_35530 | amino_acid_biosynthesis_me | PGD1656574 | bkdA1 | DrhlR DcbrA Dcrc vs WT | 2.68231 | 2.01E-64 |
| PA14_35900 | putative_enzymes | PGD1656638 | NA | DrhlR DcbrA Dcrc vs WT | -1.15311 | 0.026243 |
| PA14_36100 | biosynthesis_of_cofactors | PGD1656672 | pdxA | DrhlR DcbrA Dcrc vs WT | 2.07906 | 8.46E-07 |
| PA14_36310 | central_intermediary_metab | PGD1656706 | hcnC | DrhlR DcbrA Dcrc vs WT | -1.10293 | 1.05E-07 |
| PA14_36320 | central_intermediary_metab | PGD1656708 | hcnB | DrhlR DcbrA Dcrc vs WT | -1.21961 | 9.18E-07 |
| PA14_36330 | central_intermediary_metab | PGD1656710 | hcnA | DrhlR DcbrA Dcrc vs WT | -1.55893 | 3.12E-13 |
| PA14_36360 | membrane_proteins | PGD1656716 | NA | DrhlR DcbrA Dcrc vs WT | -1.2704 | 3.68E-04 |
| PA14_36605 | putative_enzymes | PGD1656758 | NA | DrhlR DcbrA Dcrc vs WT | 1.183136 | 0.002924 |
| PA14_36710 | energy_metabolism_CAP | PGD1656776 | glgB | DrhlR DcbrA Dcrc vs WT | 1.28063 | 0.008189 |
| PA14_36940 | membrane_proteins | PGD1656816 | NA | DrhlR DcbrA Dcrc vs WT | -1.04235 | 3.99E-04 |
| PA14_37040 | chaperones_heat_shock | PGD1656830 | cupA2 | DrhlR DcbrA Dcrc vs WT | 2.356614 | 0.021857 |
| PA14_37060 | motility_and_attachment | PGD1656832 | cupA1 | DrhlR DcbrA Dcrc vs WT | 2.338 | 2.85E-06 |
| PA14_37070 | central_intermediary_metab | PGD1656834 | NA | DrhlR DcbrA Dcrc vs WT | 2.610901 | 1.19E-27 |
| PA14_37070 | transcriptional_regulators | PGD1656834 | NA | DrhlR DcbrA Dcrc vs WT | 2.610901 | 1.19E-27 |
| PA14_37080 | transcriptional_regulators | PGD1656836 | NA | DrhlR DcbrA Dcrc vs WT | 1.952283 | 6.06E-15 |
| PA14_37190 | DNA_replication_recombinat | PGD1656852 | ada | DrhlR DcbrA Dcrc vs WT | 1.408693 | 1.09E-10 |
| PA14_37190 | transcriptional_regulators | PGD1656852 | ada | DrhlR DcbrA Dcrc vs WT | 1.408693 | 1.09E-10 |
| PA14_37210 | hypothetical_unclassified | PGD1656856 | NA | DrhlR DcbrA Dcrc vs WT | 2.202535 | 5.20E-20 |
| PA14_37250 | transport_of_small_molecule | PGD1656860 | NA | DrhlR DcbrA Dcrc vs WT | 2.304805 | 1.30E-27 |

| | | | | | | |
|---|---|---|---|---|---|---|
| PA14_37260 | transport_of_small_molecule | PGD1656862 | NA | DrhlR DcbrA Dcrc vs WT | 2.735775 | 3.05E-38 |
| PA14_37270 | putative_enzymes | PGD1656864 | NA | DrhlR DcbrA Dcrc vs WT | 4.369126 | 5.22E-57 |
| PA14_37290 | transport_of_small_molecule | PGD1656866 | NA | DrhlR DcbrA Dcrc vs WT | 3.353382 | 1.44E-33 |
| PA14_37310 | transport_of_small_molecule | PGD1656868 | NA | DrhlR DcbrA Dcrc vs WT | 3.449624 | 3.11E-49 |
| PA14_37320 | membrane_proteins | PGD1656870 | NA | DrhlR DcbrA Dcrc vs WT | 2.057108 | 4.61E-14 |
| PA14_37360 | central_intermediary_metab | PGD1656876 | NA | DrhlR DcbrA Dcrc vs WT | -2.24114 | 9.94E-13 |
| PA14_37380 | transport_of_small_molecule | PGD1656880 | NA | DrhlR DcbrA Dcrc vs WT | -2.01147 | 1.05E-17 |
| PA14_37420 | transport_of_small_molecule | PGD1656886 | NA | DrhlR DcbrA Dcrc vs WT | 1.821262 | 1.61E-04 |
| PA14_37460 | transport_of_small_molecule | PGD1656892 | NA | DrhlR DcbrA Dcrc vs WT | 1.262158 | 0.00452 |
| PA14_37470 | energy_metabolism_CAP | PGD1656894 | NA | DrhlR DcbrA Dcrc vs WT | 1.802562 | 4.18E-04 |
| PA14_37520 | hypothetical_unclassified | PGD1656900 | NA | DrhlR DcbrA Dcrc vs WT | 2.495393 | 0.004064 |
| PA14_37530 | putative_enzymes | PGD1656902 | NA | DrhlR DcbrA Dcrc vs WT | 1.384902 | 0.004636 |
| PA14_37560 | amino_acid_biosynthesis_me | PGD1656906 | NA | DrhlR DcbrA Dcrc vs WT | 1.466043 | 6.82E-04 |
| PA14_37730 | transport_of_small_molecule | PGD1656932 | NA | DrhlR DcbrA Dcrc vs WT | -1.74245 | 6.29E-15 |
| PA14_37745 | putative_enzymes | PGD1656934 | NA | DrhlR DcbrA Dcrc vs WT | -6.57202 | 1.09E-27 |
| PA14_37760 | membrane_proteins | PGD1656936 | NA | DrhlR DcbrA Dcrc vs WT | -4.79687 | 1.26E-67 |
| PA14_37760 | transport_of_small_molecule | PGD1656936 | NA | DrhlR DcbrA Dcrc vs WT | -4.79687 | 1.26E-67 |
| PA14_37770 | carbon_compound_catabolis | PGD1656938 | NA | DrhlR DcbrA Dcrc vs WT | -2.47156 | 3.64E-16 |
| PA14_37780 | hypothetical_unclassified | PGD1656940 | NA | DrhlR DcbrA Dcrc vs WT | -3.97997 | 2.46E-37 |
| PA14_37790 | adaptation_and_protection | PGD1656942 | pcoA | DrhlR DcbrA Dcrc vs WT | -1.99852 | 6.10E-19 |
| PA14_37810 | adaptation_and_protection | PGD1656944 | pcoB | DrhlR DcbrA Dcrc vs WT | -1.78599 | 1.19E-04 |
| PA14_38210 | central_intermediary_metab | PGD1657006 | NA | DrhlR DcbrA Dcrc vs WT | 2.273475 | 1.76E-07 |
| PA14_38220 | transport_of_small_molecule | PGD1657008 | NA | DrhlR DcbrA Dcrc vs WT | 1.834753 | 3.31E-07 |
| PA14_38260 | hypothetical_unclassified | PGD1657012 | NA | DrhlR DcbrA Dcrc vs WT | -2.85298 | 3.17E-21 |
| PA14_38270 | hypothetical_unclassified | PGD1657014 | NA | DrhlR DcbrA Dcrc vs WT | -3.60036 | 2.58E-29 |
| PA14_38340 | antibiotic_resistance_and_su | PGD1657026 | NA | DrhlR DcbrA Dcrc vs WT | 1.108146 | 2.25E-05 |
| PA14_38395 | antibiotic_resistance_and_su | PGD1657036 | NA | DrhlR DcbrA Dcrc vs WT | 1.411077 | 3.12E-04 |
| PA14_38430 | transcriptional_regulators | PGD1657042 | gnyR | DrhlR DcbrA Dcrc vs WT | 1.079198 | 5.39E-07 |
| PA14_38440 | carbon_compound_catabolis | PGD1657044 | gnyD | DrhlR DcbrA Dcrc vs WT | 2.054681 | 4.44E-75 |
| PA14_38440 | fatty_acid_and_phospholipid | PGD1657044 | gnyD | DrhlR DcbrA Dcrc vs WT | 2.054681 | 4.44E-75 |
| PA14_38460 | carbon_compound_catabolis | PGD1657046 | gnyB | DrhlR DcbrA Dcrc vs WT | 2.348491 | 2.93E-54 |
| PA14_38470 | carbon_compound_catabolis | PGD1657048 | gnyH | DrhlR DcbrA Dcrc vs WT | 2.208036 | 4.94E-36 |
| PA14_38480 | carbon_compound_catabolis | PGD1657050 | gnyA | DrhlR DcbrA Dcrc vs WT | 2.578491 | 5.11E-58 |
| PA14_38490 | carbon_compound_catabolis | PGD1657052 | gnyL | DrhlR DcbrA Dcrc vs WT | 1.862175 | 1.80E-49 |
| PA14_38580 | membrane_proteins | PGD1657066 | NA | DrhlR DcbrA Dcrc vs WT | 2.216999 | 5.19E-20 |
| PA14_38580 | transport_of_small_molecule | PGD1657066 | NA | DrhlR DcbrA Dcrc vs WT | 2.216999 | 5.19E-20 |
| PA14_38590 | carbon_compound_catabolis | PGD1657068 | bdhA | DrhlR DcbrA Dcrc vs WT | 1.14371 | 1.45E-08 |
| PA14_38630 | fatty_acid_and_phospholipid | PGD1657072 | atoB | DrhlR DcbrA Dcrc vs WT | 1.122469 | 4.80E-06 |
| PA14_38690 | fatty_acid_and_phospholipid | PGD1657080 | NA | DrhlR DcbrA Dcrc vs WT | 1.010724 | 4.21E-07 |
| PA14_38690 | putative_enzymes | PGD1657080 | NA | DrhlR DcbrA Dcrc vs WT | 1.010724 | 4.21E-07 |
| PA14_38730 | transport_of_small_molecule | PGD1657088 | NA | DrhlR DcbrA Dcrc vs WT | 1.455071 | 1.68E-06 |
| PA14_38740 | two_component_regulators | PGD1657090 | NA | DrhlR DcbrA Dcrc vs WT | 1.0659 | 7.60E-08 |
| PA14_38880 | hypothetical_unclassified | PGD1657112 | NA | DrhlR DcbrA Dcrc vs WT | 2.841619 | 0.014671 |
| PA14_38930 | transcriptional_regulators | PGD1657120 | NA | DrhlR DcbrA Dcrc vs WT | 1.262103 | 0.001006 |
| PA14_38950 | membrane_proteins | PGD1657122 | NA | DrhlR DcbrA Dcrc vs WT | 1.339026 | 0.002385 |
| PA14_38970 | two_component_regulators | PGD1657124 | NA | DrhlR DcbrA Dcrc vs WT | 1.033361 | 0.002493 |
| PA14_39180 | membrane_proteins | PGD1657156 | NA | DrhlR DcbrA Dcrc vs WT | 1.073165 | 1.48E-04 |
| PA14_39240 | hypothetical_unclassified | PGD1657168 | fapC | DrhlR DcbrA Dcrc vs WT | -2.13366 | 0.002137 |
| PA14_39280 | carbon_compound_catabolis | PGD1657176 | rbsK | DrhlR DcbrA Dcrc vs WT | 1.08291 | 8.56E-07 |
| PA14_39300 | carbon_compound_catabolis | PGD1657178 | rbsR | DrhlR DcbrA Dcrc vs WT | 1.030393 | 2.91E-06 |

| | | | | | | |
|---|---|---|---|---|---|---|
| PA14_39330 | transport_of_small_molecule | PGD1657182 | rbsA | DrhlR DcbrA Dcrc vs WT | 1.722698 | 8.37E-12 |
| PA14_39360 | transcriptional_regulators | PGD1657186 | NA | DrhlR DcbrA Dcrc vs WT | -1.09398 | 2.11E-05 |
| PA14_39460 | hypothetical_unclassified | PGD1657196 | NA | DrhlR DcbrA Dcrc vs WT | -2.93857 | 5.36E-21 |
| PA14_39470 | hypothetical_unclassified | PGD1657198 | NA | DrhlR DcbrA Dcrc vs WT | -1.58259 | 2.28E-06 |
| PA14_39500 | hypothetical_unclassified | PGD1657202 | NA | DrhlR DcbrA Dcrc vs WT | 3.34141 | 8.31E-29 |
| PA14_39540 | energy_metabolism_CAP | PGD1657208 | NA | DrhlR DcbrA Dcrc vs WT | -1.09675 | 1.08E-09 |
| PA14_39590 | amino_acid_biosynthesis_me | PGD1657216 | metE | DrhlR DcbrA Dcrc vs WT | -2.24045 | 3.11E-07 |
| PA14_39660 | hypothetical_unclassified | PGD1657228 | NA | DrhlR DcbrA Dcrc vs WT | 1.00247 | 0.017831 |
| PA14_39690 | nucleotide_biosynthesis_and | PGD1657232 | NA | DrhlR DcbrA Dcrc vs WT | 1.275873 | 1.82E-06 |
| PA14_39710 | nucleotide_biosynthesis_and | PGD1657236 | NA | DrhlR DcbrA Dcrc vs WT | 1.009389 | 0.029971 |
| PA14_39710 | translation_posttranslational | PGD1657236 | NA | DrhlR DcbrA Dcrc vs WT | 1.009389 | 0.029971 |
| PA14_39780 | transport_of_small_molecule | PGD1657246 | NA | DrhlR DcbrA Dcrc vs WT | -1.73341 | 5.95E-11 |
| PA14_39800 | transcriptional_regulators | PGD1657250 | NA | DrhlR DcbrA Dcrc vs WT | 1.450631 | 0.001341 |
| PA14_39810 | membrane_proteins | PGD1657252 | NA | DrhlR DcbrA Dcrc vs WT | 2.05603 | 1.30E-04 |
| PA14_39880 | secreted_factors | PGD1657264 | phzG2 | DrhlR DcbrA Dcrc vs WT | -6.09041 | 3.81E-22 |
| PA14_39945 | secreted_factors | PGD1657272 | phzC2 | DrhlR DcbrA Dcrc vs WT | -5.53388 | 1.40E-19 |
| PA14_39960 | secreted_factors | PGD1657274 | phzB2 | DrhlR DcbrA Dcrc vs WT | -7.5631 | 8.76E-33 |
| PA14_39970 | secreted_factors | PGD1657276 | phzA2 | DrhlR DcbrA Dcrc vs WT | -8.24963 | 1.71E-24 |
| PA14_40010 | hypothetical_unclassified | PGD1657282 | NA | DrhlR DcbrA Dcrc vs WT | -1.01629 | 2.01E-06 |
| PA14_40020 | hypothetical_unclassified | PGD1657284 | NA | DrhlR DcbrA Dcrc vs WT | -1.10739 | 2.99E-07 |
| PA14_40230 | protein_secretion_export | PGD1657320 | NA | DrhlR DcbrA Dcrc vs WT | -1.23079 | 2.91E-08 |
| PA14_40250 | membrane_proteins | PGD1657324 | NA | DrhlR DcbrA Dcrc vs WT | -1.02399 | 5.03E-07 |
| PA14_40270 | membrane_proteins | PGD1657328 | NA | DrhlR DcbrA Dcrc vs WT | -2.91976 | 1.66E-10 |
| PA14_40270 | transport_of_small_molecule | PGD1657328 | NA | DrhlR DcbrA Dcrc vs WT | -2.91976 | 1.66E-10 |
| PA14_40290 | secreted_factors | PGD1657332 | lasA | DrhlR DcbrA Dcrc vs WT | -2.11535 | 1.19E-27 |
| PA14_40290 | translation_posttranslational | PGD1657332 | lasA | DrhlR DcbrA Dcrc vs WT | -2.11535 | 1.19E-27 |
| PA14_40300 | hypothetical_unclassified | PGD1657334 | NA | DrhlR DcbrA Dcrc vs WT | -1.36095 | 7.81E-11 |
| PA14_40310 | fatty_acid_and_phospholipid | PGD1657336 | NA | DrhlR DcbrA Dcrc vs WT | -5.08121 | 2.80E-36 |
| PA14_40330 | protein_secretion_export | PGD1657340 | NA | DrhlR DcbrA Dcrc vs WT | 2.42845 | 6.14E-04 |
| PA14_40380 | transcriptional_regulators | PGD1657348 | NA | DrhlR DcbrA Dcrc vs WT | 1.194673 | 0.021557 |
| PA14_40430 | putative_enzymes | PGD1657356 | NA | DrhlR DcbrA Dcrc vs WT | 1.044256 | 2.16E-09 |
| PA14_40430 | transport_of_small_molecule | PGD1657356 | NA | DrhlR DcbrA Dcrc vs WT | 1.044256 | 2.16E-09 |
| PA14_41440 | adaptation_and_protection | PGD1657516 | NA | DrhlR DcbrA Dcrc vs WT | 1.299803 | 2.40E-12 |
| PA14_41520 | adaptation_and_protection | PGD1657530 | NA | DrhlR DcbrA Dcrc vs WT | -1.27306 | 0.012613 |
| PA14_42050 | putative_enzymes | PGD1657616 | NA | DrhlR DcbrA Dcrc vs WT | -1.0772 | 0.046134 |
| PA14_42080 | fatty_acid_and_phospholipid | PGD1657620 | NA | DrhlR DcbrA Dcrc vs WT | 3.723237 | 8.88E-49 |
| PA14_42080 | putative_enzymes | PGD1657620 | NA | DrhlR DcbrA Dcrc vs WT | 3.723237 | 8.88E-49 |
| PA14_42090 | fatty_acid_and_phospholipid | PGD1657622 | NA | DrhlR DcbrA Dcrc vs WT | 3.678141 | 9.90E-35 |
| PA14_42390 | transcriptional_regulators | PGD1657668 | exsA | DrhlR DcbrA Dcrc vs WT | 1.577469 | 3.02E-08 |
| PA14_42450 | protein_secretion_export | PGD1657678 | popB | DrhlR DcbrA Dcrc vs WT | 1.134526 | 0.019057 |
| PA14_42460 | protein_secretion_export | PGD1657680 | pcrH | DrhlR DcbrA Dcrc vs WT | 2.156146 | 0.034691 |
| PA14_42460 | secreted_factors | PGD1657680 | pcrH | DrhlR DcbrA Dcrc vs WT | 2.156146 | 0.034691 |
| PA14_42480 | protein_secretion_export | PGD1657684 | pcrG | DrhlR DcbrA Dcrc vs WT | 3.817757 | 0.023509 |
| PA14_42780 | hypothetical_unclassified | PGD1657738 | NA | DrhlR DcbrA Dcrc vs WT | 1.223789 | 6.34E-15 |
| PA14_42860 | transport_of_small_molecule | PGD1657750 | NA | DrhlR DcbrA Dcrc vs WT | 1.638303 | 5.25E-10 |
| PA14_42880 | protein_secretion_export | PGD1657754 | stk1 | DrhlR DcbrA Dcrc vs WT | -1.27622 | 2.54E-09 |
| PA14_42880 | translation_posttranslational | PGD1657754 | stk1 | DrhlR DcbrA Dcrc vs WT | -1.27622 | 2.54E-09 |
| PA14_42890 | protein_secretion_export | PGD1657756 | stp1 | DrhlR DcbrA Dcrc vs WT | -1.09707 | 5.31E-05 |
| PA14_42890 | translation_posttranslational | PGD1657756 | stp1 | DrhlR DcbrA Dcrc vs WT | -1.09707 | 5.31E-05 |
| PA14_42900 | hypothetical_unclassified | PGD1657758 | icmF2 | DrhlR DcbrA Dcrc vs WT | -1.26103 | 1.86E-15 |

| | | | | | | |
|---|---|---|---|---|---|---|
| PA14_42960 | protein_secretion_export | PGD1657768 | lip2.2 | DrhlR DcbrA Dcrc vs WT | -1.85394 | 2.44E-08 |
| PA14_42970 | transcriptional_regulators | PGD1657770 | sfa2 | DrhlR DcbrA Dcrc vs WT | -1.50275 | 1.55E-17 |
| PA14_42980 | chaperones_heat_shock | PGD1657772 | clpV2 | DrhlR DcbrA Dcrc vs WT | -1.17335 | 1.03E-12 |
| PA14_42990 | hypothetical_unclassified | PGD1657774 | hsiH2 | DrhlR DcbrA Dcrc vs WT | -1.73122 | 4.74E-18 |
| PA14_43000 | protein_secretion_export | PGD1657776 | hsiG2 | DrhlR DcbrA Dcrc vs WT | -1.24169 | 7.79E-11 |
| PA14_43020 | protein_secretion_export | PGD1657778 | hsiF2 | DrhlR DcbrA Dcrc vs WT | -1.02589 | 1.17E-05 |
| PA14_43030 | protein_secretion_export | PGD1657780 | hsiC2 | DrhlR DcbrA Dcrc vs WT | -1.35924 | 1.92E-12 |
| PA14_43040 | protein_secretion_export | PGD1657782 | hsiB2 | DrhlR DcbrA Dcrc vs WT | -1.47762 | 5.40E-15 |
| PA14_43050 | hypothetical_unclassified | PGD1657784 | hsiA2 | DrhlR DcbrA Dcrc vs WT | -1.49606 | 1.90E-15 |
| PA14_43070 | secreted_factors | PGD1657786 | hcp2 | DrhlR DcbrA Dcrc vs WT | -1.32109 | 2.27E-05 |
| PA14_43090 | hypothetical_unclassified | PGD1657790 | tap | DrhlR DcbrA Dcrc vs WT | -1.24018 | 0.00134 |
| PA14_43170 | transport_of_small_molecule | PGD1657804 | NA | DrhlR DcbrA Dcrc vs WT | 1.208305 | 4.56E-14 |
| PA14_43200 | transport_of_small_molecule | PGD1657810 | NA | DrhlR DcbrA Dcrc vs WT | 1.002889 | 1.75E-10 |
| PA14_43540 | putative_enzymes | PGD1657862 | NA | DrhlR DcbrA Dcrc vs WT | 1.26728 | 2.00E-06 |
| PA14_43550 | putative_enzymes | PGD1657864 | NA | DrhlR DcbrA Dcrc vs WT | 1.948661 | 8.96E-17 |
| PA14_43710 | chemotaxis | PGD1657890 | NA | DrhlR DcbrA Dcrc vs WT | 1.306098 | 3.27E-10 |
| PA14_43760 | hypothetical_unclassified | PGD1657898 | NA | DrhlR DcbrA Dcrc vs WT | 1.865342 | 3.57E-15 |
| PA14_43850 | chaperones_heat_shock | PGD1657914 | htpG | DrhlR DcbrA Dcrc vs WT | 1.316821 | 4.06E-16 |
| PA14_44340 | energy_metabolism_CAP | PGD1657994 | NA | DrhlR DcbrA Dcrc vs WT | 1.640589 | 2.20E-18 |
| PA14_44350 | central_intermediary_metab | PGD1657996 | NA | DrhlR DcbrA Dcrc vs WT | 2.18829 | 1.02E-18 |
| PA14_44350 | energy_metabolism_CAP | PGD1657996 | NA | DrhlR DcbrA Dcrc vs WT | 2.18829 | 1.02E-18 |
| PA14_44360 | energy_metabolism_CAP | PGD1657998 | NA | DrhlR DcbrA Dcrc vs WT | 1.60282 | 3.36E-15 |
| PA14_44470 | biosynthesis_of_cofactors | PGD1658018 | hemN | DrhlR DcbrA Dcrc vs WT | 1.415733 | 2.38E-12 |
| PA14_44710 | nucleotide_biosynthesis_and | PGD1658062 | xdhA | DrhlR DcbrA Dcrc vs WT | 1.169297 | 7.58E-08 |
| PA14_44960 | putative_enzymes | PGD1658098 | NA | DrhlR DcbrA Dcrc vs WT | 1.638191 | 8.00E-08 |
| PA14_45000 | central_intermediary_metab | PGD1658106 | gcl | DrhlR DcbrA Dcrc vs WT | -1.26312 | 0.002974 |
| PA14_45030 | carbon_compound_catabolis | PGD1658112 | NA | DrhlR DcbrA Dcrc vs WT | -2.20642 | 0.004083 |
| PA14_45030 | putative_enzymes | PGD1658112 | NA | DrhlR DcbrA Dcrc vs WT | -2.20642 | 0.004083 |
| PA14_45150 | transcriptional_regulators | PGD1658130 | NA | DrhlR DcbrA Dcrc vs WT | -1.25503 | 0.022906 |
| PA14_45460 | transcriptional_regulators | PGD1658174 | NA | DrhlR DcbrA Dcrc vs WT | -1.20444 | 2.20E-05 |
| PA14_45970 | membrane_proteins | PGD1658250 | NA | DrhlR DcbrA Dcrc vs WT | 1.641888 | 8.95E-09 |
| PA14_45970 | transport_of_small_molecule | PGD1658250 | NA | DrhlR DcbrA Dcrc vs WT | 1.641888 | 8.95E-09 |
| PA14_46080 | hypothetical_unclassified | PGD1658266 | NA | DrhlR DcbrA Dcrc vs WT | -1.26263 | 0.022912 |
| PA14_46110 | transport_of_small_molecule | PGD1658270 | NA | DrhlR DcbrA Dcrc vs WT | 1.014187 | 0.00477 |
| PA14_46140 | putative_enzymes | PGD1658274 | NA | DrhlR DcbrA Dcrc vs WT | 1.521718 | 0.024095 |
| PA14_46670 | hypothetical_unclassified | PGD1658370 | NA | DrhlR DcbrA Dcrc vs WT | -1.23975 | 0.031453 |
| PA14_46760 | hypothetical_unclassified | PGD1658384 | NA | DrhlR DcbrA Dcrc vs WT | 1.110799 | 0.007259 |
| PA14_47150 | energy_metabolism_CAP | PGD1658452 | cyoE | DrhlR DcbrA Dcrc vs WT | -1.10546 | 0.005439 |
| PA14_47210 | energy_metabolism_CAP | PGD1658460 | cyoA | DrhlR DcbrA Dcrc vs WT | -1.6406 | 0.003051 |
| PA14_47230 | transport_of_small_molecule | PGD1658462 | NA | DrhlR DcbrA Dcrc vs WT | 1.194003 | 6.40E-05 |
| PA14_47380 | transport_of_small_molecule | PGD1658490 | NA | DrhlR DcbrA Dcrc vs WT | 1.119173 | 5.09E-04 |
| PA14_47390 | two_component_regulators | PGD1658492 | NA | DrhlR DcbrA Dcrc vs WT | 1.369011 | 2.27E-04 |
| PA14_47400 | transcriptional_regulators | PGD1658494 | NA | DrhlR DcbrA Dcrc vs WT | 1.114852 | 0.002908 |
| PA14_47530 | hypothetical_unclassified | PGD1658516 | NA | DrhlR DcbrA Dcrc vs WT | 1.623585 | 2.16E-09 |
| PA14_47610 | transcriptional_regulators | PGD1658528 | NA | DrhlR DcbrA Dcrc vs WT | 1.226067 | 4.18E-04 |
| PA14_47670 | biosynthesis_of_cofactors | PGD1658536 | cobT | DrhlR DcbrA Dcrc vs WT | 1.059402 | 0.001729 |
| PA14_48115 | protein_secretion_export | PGD1658602 | aprD | DrhlR DcbrA Dcrc vs WT | 1.037367 | 1.89E-04 |
| PA14_48280 | antibiotic_resistance_and_su | PGD1658622 | NA | DrhlR DcbrA Dcrc vs WT | 1.467792 | 0.049823 |
| PA14_48460 | transport_of_small_molecule | PGD1658650 | NA | DrhlR DcbrA Dcrc vs WT | -1.0979 | 0.017156 |
| PA14_48530 | putative_enzymes | PGD1658662 | NA | DrhlR DcbrA Dcrc vs WT | -2.84645 | 3.76E-07 |

| | | | | | | |
|---|---|---|---|---|---|---|
| PA14_48560 | hypothetical_unclassified | PGD1658668 | NA | DrhlR DcbrA Dcrc vs WT | -2.2913 | 0.01266 |
| PA14_48570 | amino_acid_biosynthesis_me | PGD1658670 | NA | DrhlR DcbrA Dcrc vs WT | -1.47407 | 0.00904 |
| PA14_48590 | hypothetical_unclassified | PGD1658672 | NA | DrhlR DcbrA Dcrc vs WT | -1.61369 | 0.013835 |
| PA14_48600 | putative_enzymes | PGD1658674 | NA | DrhlR DcbrA Dcrc vs WT | -1.5199 | 0.011621 |
| PA14_48630 | transport_of_small_molecule | PGD1658680 | NA | DrhlR DcbrA Dcrc vs WT | -1.15277 | 1.29E-04 |
| PA14_49070 | putative_enzymes | PGD1658760 | NA | DrhlR DcbrA Dcrc vs WT | 1.587755 | 4.15E-13 |
| PA14_49080 | putative_enzymes | PGD1658762 | NA | DrhlR DcbrA Dcrc vs WT | 2.356645 | 8.42E-13 |
| PA14_49220 | energy_metabolism_CAP | PGD1658784 | napF | DrhlR DcbrA Dcrc vs WT | 1.544081 | 8.17E-07 |
| PA14_49300 | putative_enzymes | PGD1658798 | NA | DrhlR DcbrA Dcrc vs WT | -2.32246 | 2.29E-24 |
| PA14_49310 | hypothetical_unclassified | PGD1658800 | NA | DrhlR DcbrA Dcrc vs WT | -2.3059 | 2.93E-24 |
| PA14_49560 | secreted_factors | PGD1658840 | toxA | DrhlR DcbrA Dcrc vs WT | 3.188366 | 2.21E-22 |
| PA14_49570 | membrane_proteins | PGD1658842 | NA | DrhlR DcbrA Dcrc vs WT | 2.418818 | 1.59E-10 |
| PA14_49690 | putative_enzymes | PGD1658862 | NA | DrhlR DcbrA Dcrc vs WT | 2.303892 | 4.43E-06 |
| PA14_49720 | hypothetical_unclassified | PGD1658868 | NA | DrhlR DcbrA Dcrc vs WT | 1.296385 | 0.002759 |
| PA14_49750 | antibiotic_resistance_and_su | PGD1658874 | NA | DrhlR DcbrA Dcrc vs WT | -5.97794 | 4.07E-54 |
| PA14_49750 | membrane_proteins | PGD1658874 | NA | DrhlR DcbrA Dcrc vs WT | -5.97794 | 4.07E-54 |
| PA14_49750 | transport_of_small_molecule | PGD1658874 | NA | DrhlR DcbrA Dcrc vs WT | -5.97794 | 4.07E-54 |
| PA14_49760 | adaptation_and_protection | PGD1658876 | rhlC | DrhlR DcbrA Dcrc vs WT | -4.60498 | 1.02E-13 |
| PA14_50070 | hypothetical_unclassified | PGD1658930 | NA | DrhlR DcbrA Dcrc vs WT | -1.35422 | 4.15E-06 |
| PA14_50750 | hypothetical_unclassified | PGD1659038 | NA | DrhlR DcbrA Dcrc vs WT | 1.951038 | 1.08E-17 |
| PA14_50760 | putative_enzymes | PGD1659040 | NA | DrhlR DcbrA Dcrc vs WT | 1.770637 | 2.82E-07 |
| PA14_50770 | transport_of_small_molecule | PGD1659042 | NA | DrhlR DcbrA Dcrc vs WT | 1.344748 | 3.42E-07 |
| PA14_50790 | hypothetical_unclassified | PGD1659044 | NA | DrhlR DcbrA Dcrc vs WT | 2.004661 | 2.46E-18 |
| PA14_50800 | biosynthesis_of_cofactors | PGD1659046 | pdxH | DrhlR DcbrA Dcrc vs WT | 1.426671 | 5.76E-23 |
| PA14_50930 | hypothetical_unclassified | PGD1659072 | NA | DrhlR DcbrA Dcrc vs WT | 1.254729 | 7.82E-15 |
| PA14_50980 | antibiotic_resistance_and_su | PGD1659080 | pac | DrhlR DcbrA Dcrc vs WT | -1.01665 | 2.45E-04 |
| PA14_50980 | carbon_compound_catabolis | PGD1659080 | pac | DrhlR DcbrA Dcrc vs WT | -1.01665 | 2.45E-04 |
| PA14_50980 | putative_enzymes | PGD1659080 | pac | DrhlR DcbrA Dcrc vs WT | -1.01665 | 2.45E-04 |
| PA14_51350 | adaptation_and_protection | PGD1659144 | phnB | DrhlR DcbrA Dcrc vs WT | 2.698062 | 1.87E-87 |
| PA14_51350 | amino_acid_biosynthesis_me | PGD1659144 | phnB | DrhlR DcbrA Dcrc vs WT | 2.698062 | 1.87E-87 |
| PA14_51360 | adaptation_and_protection | PGD1659146 | phnA | DrhlR DcbrA Dcrc vs WT | 3.245843 | 4.51E-182 |
| PA14_51380 | biosynthesis_of_cofactors | PGD1659148 | pqsE | DrhlR DcbrA Dcrc vs WT | 2.981495 | 9.07E-75 |
| PA14_51390 | hypothetical_unclassified | PGD1659150 | pqsD | DrhlR DcbrA Dcrc vs WT | 3.418646 | 2.65E-221 |
| PA14_51410 | biosynthesis_of_cofactors | PGD1659152 | pqsC | DrhlR DcbrA Dcrc vs WT | 3.295787 | 9.14E-117 |
| PA14_51420 | biosynthesis_of_cofactors | PGD1659154 | pqsB | DrhlR DcbrA Dcrc vs WT | 3.337313 | 6.36E-170 |
| PA14_51430 | biosynthesis_of_cofactors | PGD1659156 | pqsA | DrhlR DcbrA Dcrc vs WT | 2.754592 | 3.00E-157 |
| PA14_51890 | hypothetical_unclassified | PGD1659242 | NA | DrhlR DcbrA Dcrc vs WT | 1.042688 | 2.66E-05 |
| PA14_51940 | hypothetical_unclassified | PGD1659252 | NA | DrhlR DcbrA Dcrc vs WT | 2.371901 | 5.61E-15 |
| PA14_51950 | hypothetical_unclassified | PGD1659254 | NA | DrhlR DcbrA Dcrc vs WT | 2.135698 | 2.22E-17 |
| PA14_52070 | transcriptional_regulators | PGD1659274 | NA | DrhlR DcbrA Dcrc vs WT | 1.025209 | 1.63E-05 |
| PA14_52080 | hypothetical_unclassified | PGD1659276 | NA | DrhlR DcbrA Dcrc vs WT | 1.300105 | 2.41E-04 |
| PA14_52130 | hypothetical_unclassified | PGD1659284 | NA | DrhlR DcbrA Dcrc vs WT | -2.03366 | 9.55E-32 |
| PA14_52480 | hypothetical_unclassified | PGD1659340 | NA | DrhlR DcbrA Dcrc vs WT | 2.03733 | 1.39E-04 |
| PA14_52490 | hypothetical_unclassified | PGD1659342 | NA | DrhlR DcbrA Dcrc vs WT | 2.09236 | 0.00746 |
| PA14_52500 | hypothetical_unclassified | PGD1659344 | NA | DrhlR DcbrA Dcrc vs WT | 2.096306 | 0.012757 |
| PA14_52880 | putative_enzymes | PGD1659406 | NA | DrhlR DcbrA Dcrc vs WT | 3.454622 | 0.031129 |
| PA14_53250 | secreted_factors | PGD1659466 | cpbD | DrhlR DcbrA Dcrc vs WT | -2.86673 | 4.39E-63 |
| PA14_53290 | nucleotide_biosynthesis_and | PGD1659472 | trxB2 | DrhlR DcbrA Dcrc vs WT | -1.30272 | 6.18E-07 |
| PA14_53470 | putative_enzymes | PGD1659500 | NA | DrhlR DcbrA Dcrc vs WT | 1.635539 | 3.60E-08 |
| PA14_53480 | carbon_compound_catabolis | PGD1659502 | pta | DrhlR DcbrA Dcrc vs WT | 1.106232 | 2.39E-07 |

| | | | | | | |
|---|---|---|---|---|---|---|
| PA14_53530 | hypothetical_unclassified | PGD1659512 | NA | DrhlR DcbrA Dcrc vs WT | 3.479304 | 6.76E-35 |
| PA14_53690 | hypothetical_unclassified | PGD1659544 | NA | DrhlR DcbrA Dcrc vs WT | 2.194852 | 4.91E-07 |
| PA14_53700 | putative_enzymes | PGD1659546 | NA | DrhlR DcbrA Dcrc vs WT | 1.404417 | 0.003166 |
| PA14_53820 | antibiotic_resistance_and_su | PGD1659566 | NA | DrhlR DcbrA Dcrc vs WT | 1.075989 | 8.19E-04 |
| PA14_53920 | transcriptional_regulators | PGD1659586 | NA | DrhlR DcbrA Dcrc vs WT | 1.509247 | 1.14E-12 |
| PA14_53940 | carbon_compound_catabolis | PGD1659588 | prpB | DrhlR DcbrA Dcrc vs WT | 1.301924 | 1.60E-05 |
| PA14_53940 | central_intermediary_metab | PGD1659588 | prpB | DrhlR DcbrA Dcrc vs WT | 1.301924 | 1.60E-05 |
| PA14_53940 | fatty_acid_and_phospholipid | PGD1659588 | prpB | DrhlR DcbrA Dcrc vs WT | 1.301924 | 1.60E-05 |
| PA14_53950 | carbon_compound_catabolis | PGD1659590 | prpC | DrhlR DcbrA Dcrc vs WT | 1.155336 | 2.39E-07 |
| PA14_53950 | central_intermediary_metab | PGD1659590 | prpC | DrhlR DcbrA Dcrc vs WT | 1.155336 | 2.39E-07 |
| PA14_54090 | putative_enzymes | PGD1659610 | NA | DrhlR DcbrA Dcrc vs WT | 1.317937 | 6.42E-06 |
| PA14_54170 | amino_acid_biosynthesis_me | PGD1659620 | putA | DrhlR DcbrA Dcrc vs WT | 1.078395 | 2.40E-13 |
| PA14_54540 | hypothetical_unclassified | PGD1659678 | NA | DrhlR DcbrA Dcrc vs WT | 1.222555 | 5.90E-07 |
| PA14_54550 | membrane_proteins | PGD1659680 | NA | DrhlR DcbrA Dcrc vs WT | 2.360405 | 0.030376 |
| PA14_54630 | putative_enzymes | PGD1659694 | NA | DrhlR DcbrA Dcrc vs WT | 1.016902 | 4.56E-11 |
| PA14_54640 | putative_enzymes | PGD1659696 | dspI | DrhlR DcbrA Dcrc vs WT | 1.743888 | 4.50E-30 |
| PA14_54660 | putative_enzymes | PGD1659698 | NA | DrhlR DcbrA Dcrc vs WT | 1.713491 | 3.52E-24 |
| PA14_54880 | putative_enzymes | PGD1659740 | NA | DrhlR DcbrA Dcrc vs WT | -1.13213 | 2.42E-07 |
| PA14_55080 | hypothetical_unclassified | PGD1659776 | NA | DrhlR DcbrA Dcrc vs WT | -1.3679 | 3.60E-19 |
| PA14_55090 | hypothetical_unclassified | PGD1659778 | NA | DrhlR DcbrA Dcrc vs WT | -1.07469 | 1.30E-05 |
| PA14_55100 | hypothetical_unclassified | PGD1659780 | NA | DrhlR DcbrA Dcrc vs WT | 1.167922 | 2.33E-04 |
| PA14_55110 | hypothetical_unclassified | PGD1659782 | NA | DrhlR DcbrA Dcrc vs WT | 1.503133 | 1.34E-06 |
| PA14_55160 | transcriptional_regulators | PGD1659792 | toxR | DrhlR DcbrA Dcrc vs WT | 1.342559 | 4.97E-05 |
| PA14_55540 | transcriptional_regulators | PGD1659854 | NA | DrhlR DcbrA Dcrc vs WT | 1.000809 | 0.012725 |
| PA14_55580 | biosynthesis_of_cofactors | PGD1659862 | nemO | DrhlR DcbrA Dcrc vs WT | 1.278962 | 1.84E-04 |
| PA14_55730 | transcriptional_regulators | PGD1659894 | NA | DrhlR DcbrA Dcrc vs WT | 2.408395 | 6.47E-09 |
| PA14_55940 | protein_secretion_export | PGD1659930 | NA | DrhlR DcbrA Dcrc vs WT | -1.94861 | 2.41E-09 |
| PA14_56220 | adaptation_and_protection | PGD1659974 | NA | DrhlR DcbrA Dcrc vs WT | 1.786489 | 9.72E-19 |
| PA14_56540 | energy_metabolism_CAP | PGD1660014 | NA | DrhlR DcbrA Dcrc vs WT | 1.227629 | 5.84E-07 |
| PA14_56570 | fatty_acid_and_phospholipid | PGD1660020 | NA | DrhlR DcbrA Dcrc vs WT | -2.49736 | 3.76E-06 |
| PA14_56730 | hypothetical_unclassified | PGD1660042 | NA | DrhlR DcbrA Dcrc vs WT | 1.112472 | 1.05E-07 |
| PA14_56750 | hypothetical_unclassified | PGD1660046 | NA | DrhlR DcbrA Dcrc vs WT | 1.28779 | 0.006733 |
| PA14_56770 | membrane_proteins | PGD1660048 | NA | DrhlR DcbrA Dcrc vs WT | 1.756971 | 0.007109 |
| PA14_56770 | transport_of_small_molecule | PGD1660048 | NA | DrhlR DcbrA Dcrc vs WT | 1.756971 | 0.007109 |
| PA14_56990 | hypothetical_unclassified | PGD1660088 | NA | DrhlR DcbrA Dcrc vs WT | -3.13022 | 1.32E-35 |
| PA14_57010 | chaperones_heat_shock | PGD1660090 | groEL | DrhlR DcbrA Dcrc vs WT | 1.133485 | 2.38E-13 |
| PA14_57020 | chaperones_heat_shock | PGD1660092 | groES | DrhlR DcbrA Dcrc vs WT | 1.161988 | 7.28E-11 |
| PA14_57030 | membrane_proteins | PGD1660094 | fxsA | DrhlR DcbrA Dcrc vs WT | 1.435102 | 3.60E-08 |
| PA14_57990 | transport_of_small_molecule | PGD1660253 | NA | DrhlR DcbrA Dcrc vs WT | 2.08904 | 2.08E-05 |
| PA14_58000 | adaptation_and_protection | PGD1660255 | sodM | DrhlR DcbrA Dcrc vs WT | 2.183846 | 4.27E-06 |
| PA14_58010 | hypothetical_unclassified | PGD1660257 | NA | DrhlR DcbrA Dcrc vs WT | 1.805455 | 0.002116 |
| PA14_58030 | energy_metabolism_CAP | PGD1660259 | fumC | DrhlR DcbrA Dcrc vs WT | 1.937657 | 7.45E-07 |
| PA14_58040 | hypothetical_unclassified | PGD1660261 | NA | DrhlR DcbrA Dcrc vs WT | 2.618565 | 8.98E-08 |
| PA14_58500 | membrane_proteins | PGD1660333 | NA | DrhlR DcbrA Dcrc vs WT | 2.85738 | 1.71E-17 |
| PA14_58610 | putative_enzymes | PGD1660353 | NA | DrhlR DcbrA Dcrc vs WT | -1.42407 | 6.47E-05 |
| PA14_58630 | amino_acid_biosynthesis_me | PGD1660357 | NA | DrhlR DcbrA Dcrc vs WT | -1.17402 | 3.79E-16 |
| PA14_58690 | hypothetical_unclassified | PGD1660365 | NA | DrhlR DcbrA Dcrc vs WT | 1.07385 | 1.18E-11 |
| PA14_58720 | hypothetical_unclassified | PGD1660371 | NA | DrhlR DcbrA Dcrc vs WT | 1.951414 | 6.19E-16 |
| PA14_58730 | motility_and_attachment | PGD1660373 | pilA | DrhlR DcbrA Dcrc vs WT | 1.807645 | 1.48E-13 |
| PA14_59220 | secreted_factors | PGD1660467 | NA | DrhlR DcbrA Dcrc vs WT | 1.272507 | 0.0033 |

| | | | | | | |
|---|---|---|---|---|---|---|
| PA14_59360 | motility_and_attachment | PGD1660489 | pilM2 | DrhlR DcbrA Dcrc vs WT | -2.68977 | 8.13E-10 |
| PA14_59390 | hypothetical_unclassified | PGD1660495 | NA | DrhlR DcbrA Dcrc vs WT | -1.01966 | 0.002099 |
| PA14_59780 | two_component_regulators | PGD1660564 | rcsC | DrhlR DcbrA Dcrc vs WT | 1.42447 | 6.17E-11 |
| PA14_59890 | hypothetical_unclassified | PGD1660586 | NA | DrhlR DcbrA Dcrc vs WT | 2.701482 | 8.85E-05 |
| PA14_60190 | chaperones_heat_shock | PGD1660644 | clpB | DrhlR DcbrA Dcrc vs WT | 1.533391 | 4.48E-10 |
| PA14_60280 | motility_and_attachment | PGD1660660 | fimU | DrhlR DcbrA Dcrc vs WT | 1.171875 | 1.08E-10 |
| PA14_60480 | hypothetical_unclassified | PGD1660696 | NA | DrhlR DcbrA Dcrc vs WT | 1.372841 | 0.008848 |
| PA14_60490 | energy_metabolism_CAP | PGD1660698 | NA | DrhlR DcbrA Dcrc vs WT | 1.927322 | 9.86E-16 |
| PA14_60520 | hypothetical_unclassified | PGD1660702 | NA | DrhlR DcbrA Dcrc vs WT | -1.00456 | 8.40E-13 |
| PA14_60560 | hypothetical_unclassified | PGD1660710 | NA | DrhlR DcbrA Dcrc vs WT | 1.269786 | 4.65E-05 |
| NA | NA | PGD1660721 | NA | DrhlR DcbrA Dcrc vs WT | -1.07565 | 2.43E-08 |
| PA14_60700 | energy_metabolism_CAP | PGD1660732 | ccpR | DrhlR DcbrA Dcrc vs WT | 1.158757 | 2.47E-06 |
| PA14_61000 | hypothetical_unclassified | PGD1660778 | NA | DrhlR DcbrA Dcrc vs WT | 1.952473 | 3.29E-08 |
| PA14_61080 | transport_of_small_molecule | PGD1660790 | NA | DrhlR DcbrA Dcrc vs WT | 1.500433 | 9.32E-07 |
| PA14_61190 | cell_wall_LPS | PGD1660808 | NA | DrhlR DcbrA Dcrc vs WT | -1.4349 | 1.63E-23 |
| PA14_61190 | protein_secretion_export | PGD1660808 | NA | DrhlR DcbrA Dcrc vs WT | -1.4349 | 1.63E-23 |
| PA14_61200 | cell_wall_LPS | PGD1660810 | cdrA | DrhlR DcbrA Dcrc vs WT | -2.06044 | 1.63E-19 |
| PA14_61200 | secreted_factors | PGD1660810 | cdrA | DrhlR DcbrA Dcrc vs WT | -2.06044 | 1.63E-19 |
| PA14_61370 | hypothetical_unclassified | PGD1660838 | NA | DrhlR DcbrA Dcrc vs WT | 1.345475 | 0.001079 |
| PA14_62520 | hypothetical_unclassified | PGD1661030 | NA | DrhlR DcbrA Dcrc vs WT | -1.04112 | 3.09E-05 |
| PA14_62530 | carbon_compound_catabolis | PGD1661032 | cbrA | DrhlR DcbrA Dcrc vs WT | -2.8011 | 1.77E-06 |
| PA14_62530 | two_component_regulators | PGD1661032 | cbrA | DrhlR DcbrA Dcrc vs WT | -2.8011 | 1.77E-06 |
| PA14_62940 | amino_acid_biosynthesis_me | PGD1661104 | dapB | DrhlR DcbrA Dcrc vs WT | 1.371376 | 1.04E-09 |
| PA14_62960 | adaptation_and_protection | PGD1661106 | dnaJ | DrhlR DcbrA Dcrc vs WT | 1.521813 | 3.38E-10 |
| PA14_62960 | chaperones_heat_shock | PGD1661106 | dnaJ | DrhlR DcbrA Dcrc vs WT | 1.521813 | 3.38E-10 |
| PA14_62960 | DNA_replication_recombinat | PGD1661106 | dnaJ | DrhlR DcbrA Dcrc vs WT | 1.521813 | 3.38E-10 |
| PA14_62970 | adaptation_and_protection | PGD1661108 | dnaK | DrhlR DcbrA Dcrc vs WT | 1.536182 | 4.72E-17 |
| PA14_62970 | chaperones_heat_shock | PGD1661108 | dnaK | DrhlR DcbrA Dcrc vs WT | 1.536182 | 4.72E-17 |
| PA14_62970 | DNA_replication_recombinat | PGD1661108 | dnaK | DrhlR DcbrA Dcrc vs WT | 1.536182 | 4.72E-17 |
| PA14_62990 | chaperones_heat_shock | PGD1661110 | grpE | DrhlR DcbrA Dcrc vs WT | 1.787078 | 8.13E-24 |
| PA14_62990 | DNA_replication_recombinat | PGD1661110 | grpE | DrhlR DcbrA Dcrc vs WT | 1.787078 | 8.13E-24 |
| PA14_63330 | fatty_acid_and_phospholipid | PGD1661170 | NA | DrhlR DcbrA Dcrc vs WT | 1.238621 | 4.03E-13 |
| PA14_64050 | transcriptional_regulators | PGD1661278 | NA | DrhlR DcbrA Dcrc vs WT | 1.255426 | 8.41E-10 |
| PA14_64050 | two_component_regulators | PGD1661278 | NA | DrhlR DcbrA Dcrc vs WT | 1.255426 | 8.41E-10 |
| PA14_64530 | hypothetical_unclassified | PGD1661358 | NA | DrhlR DcbrA Dcrc vs WT | -1.99238 | 0.002395 |
| PA14_64690 | membrane_proteins | PGD1661386 | NA | DrhlR DcbrA Dcrc vs WT | 1.426543 | 0.002755 |
| PA14_64700 | transcriptional_regulators | PGD1661388 | NA | DrhlR DcbrA Dcrc vs WT | 1.408364 | 0.001549 |
| PA14_64750 | membrane_proteins | PGD1661396 | NA | DrhlR DcbrA Dcrc vs WT | -2.02553 | 2.36E-07 |
| PA14_64750 | transport_of_small_molecule | PGD1661396 | NA | DrhlR DcbrA Dcrc vs WT | -2.02553 | 2.36E-07 |
| PA14_64850 | amino_acid_biosynthesis_me | PGD1661412 | NA | DrhlR DcbrA Dcrc vs WT | 1.426904 | 2.14E-05 |
| PA14_64870 | transport_of_small_molecule | PGD1661416 | NA | DrhlR DcbrA Dcrc vs WT | 1.461333 | 6.59E-07 |
| PA14_64880 | membrane_proteins | PGD1661418 | NA | DrhlR DcbrA Dcrc vs WT | 1.165639 | 3.16E-04 |
| PA14_64880 | transport_of_small_molecule | PGD1661418 | NA | DrhlR DcbrA Dcrc vs WT | 1.165639 | 3.16E-04 |
| PA14_64890 | membrane_proteins | PGD1661420 | NA | DrhlR DcbrA Dcrc vs WT | 1.601245 | 6.97E-08 |
| PA14_64890 | transport_of_small_molecule | PGD1661420 | NA | DrhlR DcbrA Dcrc vs WT | 1.601245 | 6.97E-08 |
| PA14_64900 | transport_of_small_molecule | PGD1661422 | NA | DrhlR DcbrA Dcrc vs WT | 1.006108 | 3.39E-11 |
| PA14_64930 | nucleotide_biosynthesis_and | PGD1661428 | NA | DrhlR DcbrA Dcrc vs WT | -2.70384 | 3.05E-40 |
| PA14_64940 | biosynthesis_of_cofactors | PGD1661430 | NA | DrhlR DcbrA Dcrc vs WT | -3.57885 | 1.38E-33 |
| PA14_64950 | central_intermediary_metab | PGD1661432 | pncA | DrhlR DcbrA Dcrc vs WT | 2.144764 | 4.80E-23 |
| PA14_64960 | biosynthesis_of_cofactors | PGD1661434 | pncB1 | DrhlR DcbrA Dcrc vs WT | 1.445192 | 7.28E-13 |

| | | | | | | |
|---|---|---|---|---|---|---|
| PA14_65690 | nucleotide_biosynthesis_and | PGD1661538 | NA | DrhlR DcbrA Dcrc vs WT | 1.452584 | 1.61E-12 |
| PA14_65690 | putative_enzymes | PGD1661538 | NA | DrhlR DcbrA Dcrc vs WT | 1.452584 | 1.61E-12 |
| PA14_65840 | fatty_acid_and_phospholipid | PGD1661560 | NA | DrhlR DcbrA Dcrc vs WT | 1.125661 | 0.040055 |
| PA14_65920 | transport_of_small_molecule | PGD1661572 | NA | DrhlR DcbrA Dcrc vs WT | 2.522556 | 3.43E-11 |
| PA14_65940 | energy_metabolism_CAP | PGD1661574 | NA | DrhlR DcbrA Dcrc vs WT | 1.780838 | 5.72E-16 |
| PA14_65940 | putative_enzymes | PGD1661574 | NA | DrhlR DcbrA Dcrc vs WT | 1.780838 | 5.72E-16 |
| PA14_66450 | hypothetical_unclassified | PGD1661654 | NA | DrhlR DcbrA Dcrc vs WT | 3.95217 | 1.05E-74 |
| PA14_66510 | membrane_proteins | PGD1661662 | NA | DrhlR DcbrA Dcrc vs WT | -1.47501 | 1.90E-04 |
| PA14_66510 | transport_of_small_molecule | PGD1661662 | NA | DrhlR DcbrA Dcrc vs WT | -1.47501 | 1.90E-04 |
| PA14_66540 | hypothetical_unclassified | PGD1661668 | NA | DrhlR DcbrA Dcrc vs WT | 1.737554 | 5.45E-12 |
| PA14_66630 | motility_and_attachment | PGD1661684 | pilP | DrhlR DcbrA Dcrc vs WT | 1.155133 | 1.43E-06 |
| PA14_66640 | motility_and_attachment | PGD1661686 | pilO | DrhlR DcbrA Dcrc vs WT | 1.06319 | 4.94E-07 |
| PA14_66650 | motility_and_attachment | PGD1661688 | pilN | DrhlR DcbrA Dcrc vs WT | 1.316853 | 2.41E-09 |
| PA14_66660 | motility_and_attachment | PGD1661690 | pilM | DrhlR DcbrA Dcrc vs WT | 1.097169 | 9.04E-07 |
| PA14_66750 | translation_posttranslational | PGD1661704 | argS | DrhlR DcbrA Dcrc vs WT | 1.00416 | 1.13E-06 |
| PA14_66760 | cell_division | PGD1661706 | NA | DrhlR DcbrA Dcrc vs WT | 1.202276 | 3.23E-10 |
| PA14_66770 | chaperones_heat_shock | PGD1661708 | hslV | DrhlR DcbrA Dcrc vs WT | 1.242668 | 2.85E-11 |
| PA14_66790 | chaperones_heat_shock | PGD1661710 | hslU | DrhlR DcbrA Dcrc vs WT | 1.291289 | 1.99E-08 |
| PA14_66850 | transcriptional_regulators | PGD1661720 | NA | DrhlR DcbrA Dcrc vs WT | -1.17391 | 3.31E-09 |
| PA14_66880 | transcriptional_regulators | PGD1661724 | NA | DrhlR DcbrA Dcrc vs WT | -1.22138 | 1.08E-09 |
| PA14_67130 | transport_of_small_molecule | PGD1661766 | NA | DrhlR DcbrA Dcrc vs WT | 1.308653 | 4.03E-12 |
| PA14_67140 | translation_posttranslational | PGD1661768 | NA | DrhlR DcbrA Dcrc vs WT | 1.485141 | 1.17E-04 |
| PA14_67150 | amino_acid_biosynthesis_me | PGD1661770 | NA | DrhlR DcbrA Dcrc vs WT | 2.396147 | 5.89E-23 |
| PA14_67180 | hypothetical_unclassified | PGD1661774 | tli5b4 | DrhlR DcbrA Dcrc vs WT | -1.18521 | 1.36E-07 |
| PA14_67190 | hypothetical_unclassified | PGD1661776 | tli5b3 | DrhlR DcbrA Dcrc vs WT | -1.20249 | 9.06E-07 |
| PA14_67200 | hypothetical_unclassified | PGD1661778 | tli5b2 | DrhlR DcbrA Dcrc vs WT | -1.29824 | 1.25E-07 |
| PA14_67300 | transport_of_small_molecule | PGD1661796 | NA | DrhlR DcbrA Dcrc vs WT | -1.13921 | 1.61E-10 |
| PA14_67310 | transport_of_small_molecule | PGD1661798 | NA | DrhlR DcbrA Dcrc vs WT | -1.59336 | 1.56E-10 |
| PA14_67320 | amino_acid_biosynthesis_me | PGD1661800 | hutH | DrhlR DcbrA Dcrc vs WT | -1.75402 | 2.41E-09 |
| PA14_67320 | central_intermediary_metab | PGD1661800 | hutH | DrhlR DcbrA Dcrc vs WT | -1.75402 | 2.41E-09 |
| PA14_67340 | transport_of_small_molecule | PGD1661802 | NA | DrhlR DcbrA Dcrc vs WT | -1.62795 | 3.19E-16 |
| PA14_67350 | amino_acid_biosynthesis_me | PGD1661804 | hutU | DrhlR DcbrA Dcrc vs WT | -1.13079 | 9.98E-07 |
| PA14_67380 | fatty_acid_and_phospholipid | PGD1661808 | NA | DrhlR DcbrA Dcrc vs WT | -1.26819 | 6.70E-05 |
| PA14_67420 | transcriptional_regulators | PGD1661814 | hutC | DrhlR DcbrA Dcrc vs WT | 1.026093 | 1.60E-17 |
| PA14_67440 | putative_enzymes | PGD1661816 | NA | DrhlR DcbrA Dcrc vs WT | 1.271275 | 1.39E-15 |
| PA14_67640 | hypothetical_unclassified | PGD1661848 | NA | DrhlR DcbrA Dcrc vs WT | 1.28733 | 5.65E-09 |
| PA14_67840 | transport_of_small_molecule | PGD1661878 | NA | DrhlR DcbrA Dcrc vs WT | 1.524169 | 9.02E-12 |
| PA14_67850 | transport_of_small_molecule | PGD1661880 | NA | DrhlR DcbrA Dcrc vs WT | 1.709387 | 2.47E-12 |
| PA14_67860 | transport_of_small_molecule | PGD1661882 | NA | DrhlR DcbrA Dcrc vs WT | 2.418526 | 2.18E-18 |
| PA14_67940 | hypothetical_unclassified | PGD1661894 | NA | DrhlR DcbrA Dcrc vs WT | 1.257201 | 0.019049 |
| PA14_68110 | antibiotic_resistance_and_su | PGD1661924 | NA | DrhlR DcbrA Dcrc vs WT | 1.250044 | 1.17E-05 |
| PA14_68170 | cell_wall_LPS | PGD1661934 | rmlB | DrhlR DcbrA Dcrc vs WT | -2.60104 | 7.02E-113 |
| PA14_68190 | cell_wall_LPS | PGD1661936 | rmlD | DrhlR DcbrA Dcrc vs WT | -2.43732 | 2.28E-54 |
| PA14_68200 | cell_wall_LPS | PGD1661938 | rmlA | DrhlR DcbrA Dcrc vs WT | -2.28186 | 3.97E-48 |
| PA14_68210 | cell_wall_LPS | PGD1661940 | rmlC | DrhlR DcbrA Dcrc vs WT | -2.46874 | 2.02E-66 |
| PA14_68230 | transport_of_small_molecule | PGD1661942 | NA | DrhlR DcbrA Dcrc vs WT | -1.17033 | 2.86E-20 |
| PA14_68230 | two_component_regulators | PGD1661942 | NA | DrhlR DcbrA Dcrc vs WT | -1.17033 | 2.86E-20 |
| PA14_68300 | amino_acid_biosynthesis_me | PGD1661952 | arcD | DrhlR DcbrA Dcrc vs WT | 2.817922 | 8.64E-24 |
| PA14_68300 | membrane_proteins | PGD1661952 | arcD | DrhlR DcbrA Dcrc vs WT | 2.817922 | 8.64E-24 |
| PA14_68300 | transport_of_small_molecule | PGD1661952 | arcD | DrhlR DcbrA Dcrc vs WT | 2.817922 | 8.64E-24 |

| | | | | | | |
|---|---|---|---|---|---|---|
| PA14_68330 | amino_acid_biosynthesis_me | PGD1661954 | arcA | DrhlR DcbrA Dcrc vs WT | 1.713329 | 5.25E-10 |
| PA14_68340 | amino_acid_biosynthesis_me | PGD1661956 | arcB | DrhlR DcbrA Dcrc vs WT | 1.19099 | 3.98E-06 |
| PA14_68430 | putative_enzymes | PGD1661972 | NA | DrhlR DcbrA Dcrc vs WT | -1.57438 | 7.11E-18 |
| PA14_68440 | energy_metabolism_CAP | PGD1661974 | NA | DrhlR DcbrA Dcrc vs WT | -1.92098 | 2.71E-19 |
| PA14_68440 | putative_enzymes | PGD1661974 | NA | DrhlR DcbrA Dcrc vs WT | -1.92098 | 2.71E-19 |
| PA14_68780 | membrane_proteins | PGD1662028 | NA | DrhlR DcbrA Dcrc vs WT | 1.043154 | 8.38E-07 |
| PA14_68780 | transport_of_small_molecule | PGD1662028 | NA | DrhlR DcbrA Dcrc vs WT | 1.043154 | 8.38E-07 |
| PA14_68800 | transport_of_small_molecule | PGD1662030 | NA | DrhlR DcbrA Dcrc vs WT | 1.718702 | 1.26E-19 |
| PA14_68820 | protein_secretion_export | PGD1662034 | NA | DrhlR DcbrA Dcrc vs WT | 1.130667 | 4.05E-10 |
| PA14_68930 | membrane_proteins | PGD1662052 | NA | DrhlR DcbrA Dcrc vs WT | -5.1794 | 2.48E-19 |
| PA14_68930 | transport_of_small_molecule | PGD1662052 | NA | DrhlR DcbrA Dcrc vs WT | -5.1794 | 2.48E-19 |
| PA14_68940 | hypothetical_unclassified | PGD1662054 | NA | DrhlR DcbrA Dcrc vs WT | -4.5175 | 8.04E-27 |
| PA14_69270 | biosynthesis_of_cofactors | PGD1662106 | NA | DrhlR DcbrA Dcrc vs WT | 1.662022 | 1.31E-09 |
| PA14_69550 | hypothetical_unclassified | PGD1662150 | NA | DrhlR DcbrA Dcrc vs WT | -2.69078 | 0.0172 |
| PA14_69560 | secreted_factors | PGD1662152 | hcpB | DrhlR DcbrA Dcrc vs WT | -1.31163 | 2.94E-09 |
| PA14_69795 | membrane_proteins | PGD1662192 | amtB | DrhlR DcbrA Dcrc vs WT | -1.05736 | 5.70E-10 |
| PA14_69795 | transport_of_small_molecule | PGD1662192 | amtB | DrhlR DcbrA Dcrc vs WT | -1.05736 | 5.70E-10 |
| PA14_69890 | antibiotic_resistance_and_su | PGD1662206 | NA | DrhlR DcbrA Dcrc vs WT | -1.24123 | 0.005165 |
| PA14_69925 | central_intermediary_metab | PGD1662212 | poxB | DrhlR DcbrA Dcrc vs WT | 1.328516 | 5.34E-05 |
| PA14_69925 | energy_metabolism_CAP | PGD1662212 | poxB | DrhlR DcbrA Dcrc vs WT | 1.328516 | 5.34E-05 |
| PA14_70390 | carbon_compound_catabolis | PGD1662282 | crc | DrhlR DcbrA Dcrc vs WT | -3.36871 | 4.57E-68 |
| PA14_70390 | DNA_replication_recombinat | PGD1662282 | crc | DrhlR DcbrA Dcrc vs WT | -3.36871 | 4.57E-68 |
| PA14_70390 | energy_metabolism_CAP | PGD1662282 | crc | DrhlR DcbrA Dcrc vs WT | -3.36871 | 4.57E-68 |
| PA14_70600 | DNA_replication_recombinat | PGD1662314 | NA | DrhlR DcbrA Dcrc vs WT | 1.350902 | 2.30E-06 |
| PA14_70650 | putative_enzymes | PGD1662322 | NA | DrhlR DcbrA Dcrc vs WT | 2.278424 | 1.13E-10 |
| PA14_70670 | carbon_compound_catabolis | PGD1662324 | glcF | DrhlR DcbrA Dcrc vs WT | 2.207199 | 7.47E-12 |
| PA14_70670 | central_intermediary_metab | PGD1662324 | glcF | DrhlR DcbrA Dcrc vs WT | 2.207199 | 7.47E-12 |
| PA14_70670 | energy_metabolism_CAP | PGD1662324 | glcF | DrhlR DcbrA Dcrc vs WT | 2.207199 | 7.47E-12 |
| PA14_70680 | carbon_compound_catabolis | PGD1662326 | glcE | DrhlR DcbrA Dcrc vs WT | 2.189824 | 1.08E-15 |
| PA14_70680 | central_intermediary_metab | PGD1662326 | glcE | DrhlR DcbrA Dcrc vs WT | 2.189824 | 1.08E-15 |
| PA14_70690 | carbon_compound_catabolis | PGD1662328 | glcD | DrhlR DcbrA Dcrc vs WT | 1.583441 | 4.61E-11 |
| PA14_70690 | central_intermediary_metab | PGD1662328 | glcD | DrhlR DcbrA Dcrc vs WT | 1.583441 | 4.61E-11 |
| PA14_71060 | amino_acid_biosynthesis_me | PGD1662386 | sdaB | DrhlR DcbrA Dcrc vs WT | -1.16277 | 3.87E-05 |
| PA14_71240 | amino_acid_biosynthesis_me | PGD1662420 | NA | DrhlR DcbrA Dcrc vs WT | -2.29733 | 3.65E-06 |
| PA14_71250 | hypothetical_unclassified | PGD1662422 | NA | DrhlR DcbrA Dcrc vs WT | -2.649 | 0.00117 |
| PA14_71260 | amino_acid_biosynthesis_me | PGD1662424 | NA | DrhlR DcbrA Dcrc vs WT | -1.11433 | 4.06E-05 |
| PA14_71260 | energy_metabolism_CAP | PGD1662424 | NA | DrhlR DcbrA Dcrc vs WT | -1.11433 | 4.06E-05 |
| PA14_71280 | amino_acid_biosynthesis_me | PGD1662426 | NA | DrhlR DcbrA Dcrc vs WT | -1.02625 | 0.012766 |
| PA14_71280 | energy_metabolism_CAP | PGD1662426 | NA | DrhlR DcbrA Dcrc vs WT | -1.02625 | 0.012766 |
| PA14_71400 | hypothetical_unclassified | PGD1662448 | NA | DrhlR DcbrA Dcrc vs WT | 1.236714 | 3.15E-04 |
| PA14_71410 | transport_of_small_molecule | PGD1662450 | NA | DrhlR DcbrA Dcrc vs WT | -2.47324 | 3.72E-04 |
| PA14_71420 | energy_metabolism_CAP | PGD1662452 | NA | DrhlR DcbrA Dcrc vs WT | -1.85366 | 2.16E-06 |
| PA14_71460 | amino_acid_biosynthesis_me | PGD1662460 | glyA1 | DrhlR DcbrA Dcrc vs WT | -3.03593 | 4.23E-07 |
| PA14_71470 | amino_acid_biosynthesis_me | PGD1662462 | soxB | DrhlR DcbrA Dcrc vs WT | -1.96078 | 1.38E-06 |
| PA14_71470 | carbon_compound_catabolis | PGD1662462 | soxB | DrhlR DcbrA Dcrc vs WT | -1.96078 | 1.38E-06 |
| PA14_71490 | amino_acid_biosynthesis_me | PGD1662464 | soxD | DrhlR DcbrA Dcrc vs WT | -3.26489 | 0.0195 |
| PA14_71490 | carbon_compound_catabolis | PGD1662464 | soxD | DrhlR DcbrA Dcrc vs WT | -3.26489 | 0.0195 |
| PA14_71500 | carbon_compound_catabolis | PGD1662466 | soxA | DrhlR DcbrA Dcrc vs WT | -2.01901 | 1.83E-07 |
| PA14_71510 | amino_acid_biosynthesis_me | PGD1662468 | soxG | DrhlR DcbrA Dcrc vs WT | -1.72608 | 0.004721 |
| PA14_71510 | carbon_compound_catabolis | PGD1662468 | soxG | DrhlR DcbrA Dcrc vs WT | -1.72608 | 0.004721 |

| | | | | | | |
|---|---|---|---|---|---|---|
| PA14_71530 | nucleotide_biosynthesis_and | PGD1662470 | purU2 | DrhlR DcbrA Dcrc vs WT | -3.16072 | 7.92E-08 |
| PA14_71560 | amino_acid_biosynthesis_me | PGD1662472 | fdhA | DrhlR DcbrA Dcrc vs WT | -2.40261 | 8.70E-09 |
| PA14_71700 | transport_of_small_molecule | PGD1662496 | NA | DrhlR DcbrA Dcrc vs WT | 1.320568 | 0.00487 |
| PA14_71820 | putative_enzymes | PGD1662512 | NA | DrhlR DcbrA Dcrc vs WT | 1.714516 | 1.33E-13 |
| PA14_72500 | hypothetical_unclassified | PGD1662622 | NA | DrhlR DcbrA Dcrc vs WT | 1.118075 | 2.52E-05 |
| PA14_72660 | biosynthesis_of_cofactors | PGD1662648 | NA | DrhlR DcbrA Dcrc vs WT | 1.044845 | 0.030378 |
| PA14_72690 | amino_acid_biosynthesis_me | PGD1662650 | NA | DrhlR DcbrA Dcrc vs WT | 1.759617 | 5.51E-07 |
| PA14_72830 | hypothetical_unclassified | PGD1662676 | NA | DrhlR DcbrA Dcrc vs WT | -1.01407 | 0.004069 |
| PA14_72880 | biosynthesis_of_cofactors | PGD1662684 | NA | DrhlR DcbrA Dcrc vs WT | -1.50679 | 1.67E-11 |
| PA14_72890 | transcriptional_regulators | PGD1662686 | NA | DrhlR DcbrA Dcrc vs WT | -1.57215 | 6.84E-17 |
| PA14_72970 | transport_of_small_molecule | PGD1662698 | tonB | DrhlR DcbrA Dcrc vs WT | 1.26784 | 5.67E-05 |
| NA | NA | PGD21131238 | NA | DrhlR DcbrA Dcrc vs WT | -1.07575 | 0.020811 |
| PA14_99999 | NA | gene_crcZ | crcZ | DrhlR DcbrA Dcrc vs WT | -10.598 | 8.58E-26 |
