# Supplementary material for: Combinatorial control of Pseudomonas aeruginosa biofilm development by quorum-sensing and nutrient-sensing regulators
Source: mSystems. 2024 Aug 14;9(9):e00372-24. doi: 10.1128/msystems.00372-24 (PMC11406991; doi:10.1128/msystems.00372-24)
Supplement: Table S4 — Crc binding motif analysis. [file msystems.00372-24-s0003.pdf]

locus	gene_id	gene_nam	cds_id	cds_name	Log2FoldChange - Dcrc vs WT	Log2FoldChange - DrhlR DcbrA Dcrc vs DrhlR Dc	start	end	strand	coding_strand_sequence	upstream_50bp	start_codon	best_alignment	match_score	motif_variant	motif_start	motif_end	validation
PA14_18870	PGD1653857		PGD1653858	hypothetical p	-1.404815237	-2.375280874	1627576	1627755	-	[illegible]	[illegible]		[illegible]	8	AAGAAGAA	63	71	AAGAAGAA
PA14_01290	PGD1651049	coxB	PGD1651050	cytochrome c	-0.110515807	-2.001343259	125293	126417	+	[illegible]	[illegible]		[illegible]	8	AAAAACAA	25	33	AAAAACAA
PA14_39670	PGD1657230		PGD1657231	hypothetical p	-0.628706653	-1.916083394	3535826	3535948	-	[illegible]	[illegible]		[illegible]	8	AACAACAA	62	70	AACAACAA
PA14_52840	PGD1659400		PGD1659401	C4-dicarboxyl	0.296486656	-1.861343686	4684070	4685065	-	[illegible]	[illegible]		[illegible]	8	AACAACAA	28	36	AACAACAA
PA14_22110	PGD1654399		PGD1654400	hypothetical p	0.106640821	-1.818026999	1927460	1928419	-	[illegible]	[illegible]		[illegible]	8	AACAACAA	57	65	AACAACAA
PA14_22120	PGD1654401		PGD1654402	hypothetical p	-0.205714925	-1.780017722	1929042	1931366	-	[illegible]	[illegible]		[illegible]	8	AACAAGAA	0	8	AACAAGAA
PA14_44950	PGD1658096		PGD1658097	transporter	-0.238983016	-1.679194641	4007119	4008486	+	[illegible]	[illegible]		[illegible]	8	AACAATAA	24	32	AACAATAA
PA14_32270	PGD1656060		PGD1656061	porin	-0.456151931	-1.606154954	2803427	2804773	+	[illegible]	[illegible]		[illegible]	8	AAGAAAAA	13	21	AAGAAAAA
PA14_21580	PGD1654299		PGD1654300	hypothetical p	-0.686167332	-1.582867912	1870475	1871329	+	[illegible]	[illegible]		[illegible]	8	AAGAAGAA	17	25	AAGAAGAA
PA14_21480	PGD1654281	tli1	PGD1654282	type VI secret	-0.401380119	-1.547752191	1862506	1863060	+	[illegible]	[illegible]		[illegible]	8	AATAAAAA	83	91	AATAAAAA
PA14_20940	PGD1654193		PGD1654194	acyl carrier pr	-0.141997258	-1.539582876	1803293	1803532	-	[illegible]	[illegible]		[illegible]	8	AAGAACAA	43	51	AAGAACAA
PA14_18565	PGD1653803	alg8	PGD1653804	alginate biosy	-0.431896795	-1.521816791	1592374	1593867	-	[illegible]	[illegible]		[illegible]	8	AACAACAA	6	14	AACAACAA
PA14_39000	PGD1657128		PGD1657129	hypothetical p	-0.151140013	-1.472747022	3475025	3476224	-	[illegible]	[illegible]		[illegible]	8	AACAAGAA	53	61	AACAAGAA
PA14_45960	PGD1658248	lasR	PGD1658249	transcriptiona	-0.401656011	-1.405563795	4085339	4086058	-	[illegible]	[illegible]		[illegible]	8	AAGAAGAA	33	41	AAGAAGAA
PA14_04220	PGD1651511		PGD1651512	ABC transport	-0.295811435	-1.403255895	377448	378491	-	[illegible]	[illegible]		[illegible]	8	AAGAAAAA	18	26	AAGAAAAA
PA14_55940	PGD1659930		PGD1659931	hypothetical p	-0.805920055	-1.399476948	4994409	4994621	+	[illegible]	[illegible]		[illegible]	8	AACAAGAA	43	51	AACAAGAA
PA14_55380	PGD1659824		PGD1659825	hypothetical p	-0.200683806	-1.394423955	4915106	4916740	-	[illegible]	[illegible]		[illegible]	8	AACAATAA	16	24	AACAATAA
PA14_70300	PGD1662268	sphA	PGD1662269	SphA	0.156183161	-1.384754195	6268955	6269905	+	[illegible]	[illegible]		[illegible]	8	AACAATAA	29	37	AACAATAA
PA14_64750	PGD1661396		PGD1661397	MFS transport	0.190830324	-1.36985692	5768719	5770059	-	[illegible]	[illegible]		[illegible]	8	AACAAGAA	26	34	AACAAGAA
PA14_66510	PGD1661662		PGD1661663	MFS transport	-1.535769039	-1.293627916	5931759	5933075	+	[illegible]	[illegible]		[illegible]	8	AAGAAGAA	81	89	AAGAAGAA
PA14_14060	PGD1653083		PGD1653084	AMP-binding	-1.383147168	-1.284911539	1204817	1206715	+	[illegible]	[illegible]		[illegible]	8	AACAAGAA	38	46	AACAAGAA
PA14_24310	PGD1654759		PGD1654760	BNR/Asp-box	-0.543310792	-1.252725972	2111927	2113024	+	[illegible]	[illegible]		[illegible]	8	AACAACAA	28	36	AACAACAA
PA14_51510	PGD1659172		PGD1659173	hypothetical p	0.216451556	-1.165172743	4579750	4580154	-	[illegible]	[illegible]		[illegible]	8	AAAAAGAA	70	78	AAAAAGAA
PA14_46230	PGD1658288	aphA	PGD1658289	acetylpolyami	-0.721461852	-1.153484417	4109939	4110979	-	[illegible]	[illegible]		[illegible]	8	AACAAGAA	25	33	AACAAGAA
PA14_66880	PGD1661724		PGD1661725	hypothetical p	-0.475669562	-1.128146021	5973082	5973498	-	[illegible]	[illegible]		[illegible]	8	AAGAAAAA	68	76	AAGAAAAA
PA14_11980	PGD1652767		PGD1652768	hypothetical p	-0.397283354	-1.121892555	1035752	1036609	+	[illegible]	[illegible]		[illegible]	8	AACAACAA	31	39	AACAACAA
PA14_10940	PGD1652593		PGD1652594	AraC family tr	0.005204849	-1.109625962	945954	946913	+	[illegible]	[illegible]		[illegible]	8	AATAAAAA	43	51	AATAAAAA
PA14_54870	PGD1659738		PGD1659739	hypothetical p	-0.236741376	-1.099953221	4861608	4862516	-	[illegible]	[illegible]		[illegible]	8	AACAAAAA	14	22	AACAAAAA
PA14_25060	PGD1654889		PGD1654890	hypothetical p	-0.025001301	-1.096453208	2191504	2192322	-	[illegible]	[illegible]		[illegible]	8	AACAACAA	24	32	AACAACAA
PA14_60480	PGD1660696		PGD1660697	hypothetical p	-1.183262757	-1.092040578	5390436	5390660	+	[illegible]	[illegible]		[illegible]	8	AATAAGAA	3	11	AATAAGAA
PA14_18860	PGD1653855		PGD1653856	hypothetical p	0.008914597	-1.083353537	1626303	1627376	+	[illegible]	[illegible]		[illegible]	8	AACAAGAA	43	51	AACAAGAA
PA14_10440	PGD1652517		PGD1652518	porin	0.44197703	-1.08312277	899938	901194	-	[illegible]	[illegible]		[illegible]	8	AACAACAA	27	35	AACAACAA
PA14_06420	PGD1651855		PGD1651856	hypothetical p	-0.528519367	-1.061076323	564699	565457	+	[illegible]	[illegible]		[illegible]	8	AACAACAA	21	29	AACAACAA
PA14_17790	PGD1653695		PGD1653696	LysR family tr	-0.257173067	-1.0361425	1528469	1529356	-	[illegible]	[illegible]		[illegible]	8	AAGAAAAA	17	25	AAGAAAAA
PA14_71330	PGD1662434		PGD1662435	transcriptiona	0.436860866	-1.035275526	6355782	6355988	+	[illegible]	[illegible]		[illegible]	8	AAGAACAA	83	91	AAGAACAA
PA14_20560	PGD1654129	amiE	PGD1654130	acylamide am	0.244321904	-1.030010507	1768608	1769648	+	[illegible]	[illegible]		[illegible]	8	AAAAATAA	24	32	AAAAATAA
PA14_66660	PGD1661690	pilM	PGD1661691	type 4 fimbria	-0.415106823	-1.006740335	5951039	5952103	-	[illegible]	[illegible]		[illegible]	8	AAAAAGAA	2	10	AAAAAGAA
PA14_35900	PGD1656638		PGD1656639	dehydrogenas	0.030567323	-0.961178507	3195183	3196103	+	[illegible]	[illegible]		[illegible]	8	AACAATAA	26	34	AACAATAA
PA14_53620	PGD1659530		PGD1659531	hypothetical p	0.203531562	-0.957449878	4756330	4756968	+	[illegible]	[illegible]		[illegible]	8	AAGAAAAA	43	51	AAGAAAAA
PA14_68940	PGD1662054		PGD1662055	hypothetical p	0.882973957	-0.94367372	6150109	6150930	+	[illegible]	[illegible]		[illegible]	8	AACAACAA	22	30	AACAACAA
PA14_31560	PGD1655966		PGD1655967	LysR family tr	-0.389048174	-0.941014301	2743301	2744233	-	[illegible]	[illegible]		[illegible]	8	AACAAGAA	23	31	AACAAGAA
PA14_19810	PGD1654005		PGD1654006	hypothetical p	-0.130473529	-0.922710143	1706618	1708375	+	[illegible]	[illegible]		[illegible]	8	AACAAAAA	58	66	AACAAAAA
PA14_64720	PGD1661392		PGD1661393	porin	-0.169140127	-0.890277368	5765866	5767119	-	[illegible]	[illegible]		[illegible]	8	AATAACAA	43	51	AATAACAA
PA14_23340	PGD1654599	ihfB	PGD1654600	integration ho	-0.150751187	-0.863654184	2025973	2026257	+	[illegible]	[illegible]		[illegible]	8	AAAAAGAA	33	41	AAAAAGAA
PA14_53520	PGD1659510	oruR	PGD1659511	transcriptiona	-0.426556158	-0.859806921	4744858	4745877	-	[illegible]	[illegible]		[illegible]	8	AACAATAA	36	44	AACAATAA
PA14_53540	PGD1659514		PGD1659515	hydrolase	-0.428069128	-0.856883744	4746970	4747911	-	[illegible]	[illegible]		[illegible]	8	AACAACAA	24	32	AACAACAA
PA14_10660	PGD1652551		PGD1652552	transcriptiona	0.127811967	-0.847059906	918509	919420	+	[illegible]	[illegible]		[illegible]	8	AACAACAA	29	37	AACAACAA
PA14_07660	PGD1652049		PGD1652050	hypothetical p	-0.29302913	-0.844944088	658179	659450	-	[illegible]	[illegible]		[illegible]	8	AAGAACAA	83	91	AAGAACAA
PA14_18410	PGD1653785	algF	PGD1653786	alginate o-ace	-0.184930825	-0.832895324	1580299	1580949	-	[illegible]	[illegible]		[illegible]	8	AAGAAGAA	25	33	AAGAAGAA
PA14_60630	PGD1660722		PGD1660723	hypothetical p	-0.300684437	-0.799677298	5403895	5405040	+	[illegible]	[illegible]		[illegible]	8	AAGAAGAA	77	85	AAGAAGAA
PA14_63620	PGD1661216	lipC	PGD1661217	lipase LipC	-0.505920603	-0.777947622	5672656	5673585	-	[illegible]	[illegible]		[illegible]	8	AACAAGAA	53	61	AACAAGAA
PA14_46100	PGD1658268		PGD1658269	transporter	0.564573317	-0.770595337	4098409	4099920	+	[illegible]	[illegible]		[illegible]	8	AACAACAA	56	64	AACAACAA
PA14_38680	PGD1657078		PGD1657079	LysR family tr	-0.466702361	-0.758695246	3448793	3449713	+	[illegible]	[illegible]		[illegible]	8	AAAAACAA	38	46	AAAAACAA
PA14_61940	PGD1660932		PGD1660933	hypothetical p	-0.291484848	-0.748593351	5522562	5523608	-	[illegible]	[illegible]		[illegible]	8	AAGAACAA	31	39	AAGAACAA
PA14_41760	PGD1657568		PGD1657569	hypothetical p	0.134490487	-0.746801651	3727851	3728876	-	[illegible]	[illegible]		[illegible]	8	AAAAACAA	19	27	AAAAACAA
PA14_51070	PGD1659094		PGD1659095	porin	0.721495915	-0.740652471	4537734	4538984	-	[illegible]	[illegible]		[illegible]	8	AACAACAA	43	51	AACAACAA
PA14_68060	PGD1661914		PGD1661915	ABC transport	-0.013852409	-0.735963255	6072697	6073470	+	[illegible]	[illegible]		[illegible]	8	AATAACAA	31	39	AATAACAA
PA14_30770	PGD1655830		PGD1655831	AsnC family tr	-1.355530296	-0.723625035	2669350	2669787	-	[illegible]	[illegible]		[illegible]	8	AAAAAAAA	28	36	AAAAAAAA
PA14_22640	PGD1654489		PGD1654490	AraC family tr	-0.682428758	-0.69248127	1968939	1969952	+	[illegible]	[illegible]		[illegible]	8	AACAAGAA	35	43	AACAAGAA
PA14_68260	PGD1661946		PGD1661947	c4-dicarboxyla	-0.429158601	-0.680664372	6089362	6090357	+	[illegible]	[illegible]		[illegible]	8	AAGAACAA	30	38	AAGAACAA
PA14_17890	PGD1653707		PGD1653708	porin	0.426073462	-0.670872658	1536090	1537340	+	[illegible]	[illegible]		[illegible]	8	AACAACAA	56	64	AACAACAA
PA14_49610	PGD1658848		PGD1658849	MFS family tra	-0.085328457	-0.665105756	4413782	4415116	+	[illegible]	[illegible]		[illegible]	8	AACAAGAA	5	13	AACAAGAA
PA14_51200	PGD1659114		PGD1659115	hypothetical p	0.247823755	-0.658441646	4550259	4550645	-	[illegible]	[illegible]		[illegible]	8	AAGAACAA	30	38	AAGAACAA
PA14_23460	PGD1654623	orfN	PGD1654624	group 4 glyco	0.519811384	-0.65169548	2040149	2041165	+	[illegible]	[illegible]		[illegible]	8	AACAATAA	43	51	AACAATAA
PA14_70980	PGD1662378	betT1	PGD1662379	choline transp	-0.017893758	-0.647301056	6324563	6326113	+	[illegible]	[illegible]		[illegible]	8	AAAAACAA	16	24	AAAAACAA
PA14_57930	PGD1660241		PGD1660242	ABC transport	-0.026000598	-0.632488522	5155510	5156235	+	[illegible]	[illegible]		[illegible]	8	AAGAAAAA	33	41	AAGAAAAA
PA14_53340	PGD1659480		PGD1659481	hypothetical p	0.130717165	-0.628592396	4727802	4729814	+	[illegible]	[illegible]		[illegible]	8	AACAAGAA	32	40	AACAAGAA
PA14_01640	PGD1651105		PGD1651106	LysR family tr	-0.206982745	-0.615941183	148823	149740	+	[illegible]	[illegible]		[illegible]	8	AACAATAA	43	51	AACAATAA
PA14_44240	PGD1657978		PGD1657979	glutamine syr	0.598762863	-0.601020302	3935985	3937349	+	[illegible]	[illegible]		[illegible]	8	AACAAGAA	66	74	AACAAGAA
PA14_28140	PGD1655383		PGD1655384	hypothetical p	0.150443488	-0.597355669	2436828	2437226	+	[illegible]	[illegible]		[illegible]	8	AACAACAA	58	66	AACAACAA
PA14_64800	PGD1661404	vanA	PGD1661405	vanillate O-de	-0.673749916	-0.592350848	5774457	5775512	+	[illegible]	[illegible]		[illegible]	8	AAAAAAAA	27	35	AAAAAAAA
PA14_04790	PGD1651599	laoB	PGD1651600	LaoB	-0.609228884	-0.590399411	423650	424198	-	[illegible]	[illegible]		[illegible]	8	AACAACAA	43	51	AACAACAA
PA14_29420	PGD1655612		PGD1655613	hypothetical p	-0.276113451	-0.590017087	2551476	2552717	-	[illegible]	[illegible]		[illegible]	8	AACAACAA	23	31	AACAACAA
PA14_22980	PGD1654541		PGD1654542	sugar ABC tra	0.482086034	-0.578905199	1992306	1993568	+	[illegible]	[illegible]		[illegible]	8	AACAAGAA	29	37	AACAAGAA
PA14_01430	PGD1651073		PGD1651074	short chain de	0.269325011	-0.552007675	135162	135890	+	[illegible]	[illegible]		[illegible]	8	AAAAAGAA	21	29	AAAAAGAA
PA14_28630	PGD1655473		PGD1655474	hydrolase	0.016447699	-0.550820193	2466106	2466969	+	[illegible]	[illegible]		[illegible]	8	AAAAAGAA	24	32	AAAAAGAA
PA14_02930	PGD1651299		PGD1651300	oxidoreductas	0.230915386	-0.547930289	257439	258482	+	[illegible]	[illegible]		[illegible]	8	AACAAGAA	22	30	AACAAGAA
PA14_53070	PGD1659438	hpd	PGD1659439	4-hydroxyphe	-0.249026976	-0.546287306	4704698	4705771	-	[illegible]	[illegible]		[illegible]	8	AACAAGAA	27	35	AACAAGAA
PA14_54520	PGD1659676		PGD1659677	porin	-0.353955393	-0.542062754	4833756	4835039	+	[illegible]	[illegible]		[illegible]	8	AACAAGAA	28	36	AACAAGAA
PA14_07630	PGD1652045		PGD1652046	hypothetical p	0.114590549	-0.529132764	656028	656534	-	[illegible]	[illegible]		[illegible]	8	AACAAGAA	19	27	AACAAGAA
PA14_68570	PGD1661996		PGD1661997	hypothetical p	0.045190138	-0.518686108	6116265	6116621	+	[illegible]	[illegible]		[illegible]	8	AAAAATAA	16	24	AAAAATAA
PA14_17480	PGD1653639	rpoS	PGD1653640	RNA polymera	-0.150923853	-0.515690033	1499097	1500101	+	[illegible]	[illegible]		[illegible]	8	AAAAAGAA	60	68	AAAAAGAA
PA14_62720	PGD1661064	rpsO	PGD1661065	30S ribosoma	0.455640061	-0.511616064	5597582	5597851	-	[illegible]	[illegible]		[illegible]	8	AAGAAAAA	66	74	AAGAAAAA
PA14_58390	PGD1660319	dppA3	PGD1660320	dipeptide ABC	0.351301584	-0.500720009	5200657	5202258	+	[illegible]	[illegible]		[illegible]	8	AAAAAGAA	28	36	AAAAAGAA
PA14_38750	PGD1657092		PGD1657093	iron-containin	0.153388694	-0.495743314	3456053	3457216	-	[illegible]	[illegible]		[illegible]	8	AACAATAA	28	36	AACAATAA
PA14_31870	PGD1656012		PGD1656013	RND efflux m	0.069302958	-0.492859636	2772055	2773335	+	[illegible]	[illegible]		[illegible]	8	AAGAAGAA	40	48	AAGAAGAA
PA14_45240	PGD1658140		PGD1658141	amino acid pe	0.359918261	-0.485736294	4032702	4034069	-	[illegible]	[illegible]		[illegible]	8	AATAACAA	27	35	AATAACAA
PA14_35370	PGD1656550	ptxS	PGD1656551	transcriptiona	-0.611373011	-0.460566145	3144097	3145119	-	[illegible]	[illegible]		[illegible]	8	AATAAGAA	19	27	AATAAGAA
PA14_01300	PGD1651051	coxA	PGD1651052	cytochrome c	0.428171205	-0.460470941	126427	128019	+	[illegible]	[illegible]		[illegible]	8	AACAACAA	30	38	AACAACAA
PA14_13130	PGD1652937		PGD1652938	hypothetical p	-0.597722567	-0.458088722	1126214	1128139	+	[illegible]	[illegible]		[illegible]	8	AACAAAAA	22	30	AACAAAAA
PA14_43610	PGD1657872		PGD1657873	AMP-binding	0.147548139	-0.448941584	3881119	3882786	-	[illegible]	[illegible]		[illegible]	8	AACAACAA	30	38	AACAACAA
PA14_55810	PGD1659910		PGD1659911	two-compone	-0.528454193	-0.437907306	4983977	4984804	+	[illegible]	[illegible]		[illegible]	8	AAAAACAA	18	26	AAAAACAA
PA14_10870	PGD1652583		PGD1652584	hypothetical p	0.828326138	-0.428105092	940586	941890	-	[illegible]	[illegible]		[illegible]	8	AACAACAA	27	35	AACAACAA
PA14_25630	PGD1654981	rpmF	PGD1654982	50S ribosoma	0.123848578	-0.425752494	2240959	2241141	+	[illegible]	[illegible]		[illegible]	8	AACAAAAA	65	73	AACAAAAA
PA14_61560	PGD1660872		PGD1660873	MFS transport	0.44786176	-0.412675603	5492827	5494200	-	[illegible]	[illegible]		[illegible]	8	AACAACAA	56	64	AACAACAA
PA14_36090	PGD1656670		PGD1656671	porin	0.087509398	-0.404998626	3215363	3216613	-	[illegible]	[illegible]		[illegible]	8	AAAAACAA	40	48	AAAAACAA
PA14_51060	PGD1659092		PGD1659093	hypothetical p	0.338893165	-0.404308605	4536881	4537357	+	[illegible]	[illegible]		[illegible]	8	AAGAAAAA	56	64	AAGAAAAA
PA14_69770	PGD1662188	sutA	PGD1662189	SutA	-0.469285238	-0.381100406	6223248	6223565	-	[illegible]	[illegible]		[illegible]	8	AAGAAGAA	57	65	AAGAAGAA
PA14_11990	PGD1652769		PGD1652770	hydrolase	-0.591722726	-0.377602323	1036617	1037840	-	[illegible]	[illegible]		[illegible]	8	AACAACAA	13	21	AACAACAA
PA14_71160	PGD1662404		PGD1662405	hypothetical p	-0.066772451	-0.373726419	6338774	6339712	-	[illegible]	[illegible]		[illegible]	8	AAAAACAA	17	25	AAAAACAA
PA14_05010	PGD1651637		PGD1651638	hypothetical p	-0.147582198	-0.371738696	440240	441586	+	[illegible]	[illegible]		[illegible]	8	AATAATAA	5	13	AATAATAA
PA14_17730	PGD1653687		PGD1653688	hypothetical p	0.594993731	-0.36794875	1523344	1524159	+	[illegible]	[illegible]		[illegible]	8	AACAACAA	33	41	AACAACAA
PA14_18810	PGD1653847		PGD1653848	hypothetical p	0.523984856	-0.362256219	1621282	1622319	+	[illegible]	[illegible]		[illegible]	8	AAAAACAA	31	39	AAAAACAA
PA14_23070	PGD1654555	zwf	PGD1654556	glucose-6-pho	0.569640667	-0.34527038	2000159	2001628	+	[illegible]	[illegible]		[illegible]	8	AACAACAA	42	50	AACAACAA
PA14_57980	PGD1660251		PGD1660252	phosphoryl ca	0.004953989	-0.334250144	5159710	5159982	+	[illegible]	[illegible]		[illegible]	8	AACAAGAA	26	34	AACAAGAA
PA14_60790	PGD1660746		PGD1660747	ABC transport	-0.308645961	-0.320161844	5418265	5418872	-	[illegible]	[illegible]		[illegible]	8	AACAACAA	28	36	AACAACAA
PA14_07840	PGD1652077		PGD1652078	two-compone	0.015579326	-0.319798797	675575	676207	+	[illegible]	[illegible]		[illegible]	8	AAGAACAA	6	14	AAGAACAA
PA14_46770	PGD1658386		PGD1658387	hypothetical p	0.135517637	-0.313556198	4166280	4166705	+	[illegible]	[illegible]		[illegible]	8	AACAACAA	31	39	AACAACAA
PA14_15850	PGD1653371		PGD1653372	hypothetical p	-0.504566951	-0.302377923	1351402	1351956	-	[illegible]	[illegible]		[illegible]	8	AAGAAGAA	36	44	AAGAAGAA
PA14_02380	PGD1651215		PGD1651216	acid phosphat	0.519607447	-0.29708181	213891	214616	+	[illegible]	[illegible]		[illegible]	8	AACAACAA	53	61	AACAACAA
PA14_04250	PGD1651517		PGD1651518	ABC transport	-0.273273467	-0.297053551	380250	381299	-	[illegible]	[illegible]		[illegible]	8	AACAATAA	15	23	AACAATAA
PA14_32080	PGD1656034	xylX	PGD1656035	toluate 1	0.217442819	-0.269837148	2791292	2792659	+	[illegible]	[illegible]		[illegible]	8	AACAATAA	25	33	AACAATAA
PA14_52800	PGD1659394	acsA	PGD1659395	acetyl-CoA sy	0.60232614	-0.245283544	4679966	4681921	-	[illegible]	[illegible]		[illegible]	8	AAAAACAA	18	26	AAAAACAA
PA14_45030	PGD1658112		PGD1658113	hypothetical p	0.226698981	-0.24484598	4016053	4017318	+	[illegible]	[illegible]		[illegible]	8	AACAAGAA	28	36	AACAAGAA
PA14_65740	PGD1661546	thiC	PGD1661547	thiamine bios	0.440674843	-0.230938341	5853158	5855041	-	[illegible]	[illegible]		[illegible]	8	AAGAACAA	65	73	AAGAACAA
PA14_35460	PGD1656564		PGD1656565	AGCS sodium,	0.156912234	-0.230840544	3152097	3153542	-	[illegible]	[illegible]		[illegible]	8	AATAAGAA	27	35	AATAAGAA
PA14_71210	PGD1662414		PGD1662415	hypothetical p	-0.393804117	-0.230803476	6343184	6344536	-	[illegible]	[illegible]		[illegible]	8	AACAAGAA	17	25	AACAAGAA
PA14_25250	PGD1654917		PGD1654918	glyceraldehyd	0.11299087	-0.220442688	2208690	2210075	+	[illegible]	[illegible]		[illegible]	8	AATAACAA	80	88	AATAACAA
PA14_04560	PGD1651563		PGD1651564	hypothetical p	-0.099786637	-0.216096307	405267	406361	+	[illegible]	[illegible]		[illegible]	8	AACAATAA	31	39	AACAATAA
PA14_17780	PGD1653693		PGD1653694	major facilitat	0.064496962	-0.20631777	1526841	1528250	-	[illegible]	[illegible]		[illegible]	8	AACAACAA	25	33	AACAACAA
PA14_38610	PGD1657070		PGD1657071	hypothetical p	0.024224713	-0.198209069	3444304	3445728	-	[illegible]	[illegible]		[illegible]	8	AACAATAA	25	33	AACAATAA
PA14_44710	PGD1658062	xdhA	PGD1658063	xanthine dehy	-0.222028882	-0.182479364	3985489	3986943	+	[illegible]	[illegible]		[illegible]	8	AACAAGAA	25	33	AACAAGAA
PA14_27100	PGD1655199	lipA	PGD1655200	lactonizing lip	-1.013359641	-0.179926517	2354166	2355101	-	[illegible]	[illegible]		[illegible]	8	AAGAAGAA	53	61	AAGAAGAA
PA14_09770	PGD1652407	souR	PGD1652408	sarcosine oxi	0.012334696	-0.177214575	841579	842601	+	[illegible]	[illegible]		[illegible]	8	AACAAGAA	23	31	AACAAGAA
PA14_44510	PGD1658026		PGD1658027	hypothetical p	0.179737397	-0.166043852	3963327	3964163	+	[illegible]	[illegible]		[illegible]	8	AACAACAA	30	38	AACAACAA
PA14_60950	PGD1660770		PGD1660771	hypothetical p	-0.078486468	-0.144182571	5436245	5438311	-	[illegible]	[illegible]		[illegible]	8	AACAATAA	53	61	AACAATAA
PA14_67340	PGD1661802		PGD1661803	cytosine/puri	-0.767042731	-0.143238666	6013782	6015224	-	[illegible]	[illegible]		[illegible]	8	AACAAGAA	8	16	AACAAGAA
PA14_03760	PGD1651437		PGD1651438	sodium:solute	0.467910754	-0.1366603	336327	337712	+	[illegible]	[illegible]		[illegible]	8	AACAACAA	25	33	AACAACAA
PA14_08500	PGD1652197		PGD1652198	hypothetical p	0.172739797	-0.13608005	727721	728143	+	[illegible]	[illegible]		[illegible]	8	AAGAACAA	68	76	AAGAACAA
PA14_63480	PGD1661198		PGD1661199	amino acid pe	-0.126190475	-0.132908836	5659342	5660739	-	[illegible]	[illegible]		[illegible]	8	AACAAGAA	59	67	AACAAGAA
PA14_65200	PGD1661470	rnr	PGD1661471	exoribonuclea	0.075860468	-0.11519318	5810076	5812799	-	[illegible]	[illegible]		[illegible]	8	AAGAAGAA	25	33	AAGAAGAA
PA14_62630	PGD1661048	acsB	PGD1661049	acetyl-CoA sy	0.21356629	-0.099695256	5587464	5589401	+	[illegible]	[illegible]		[illegible]	8	AAAAACAA	31	39	AAAAACAA
PA14_02980	PGD1651305		PGD1651306	porin	0.633373155	-0.099054235	260374	261639	-	[illegible]	[illegible]		[illegible]	8	AACAAGAA	27	35	AACAAGAA
PA14_02630	PGD1651255		PGD1651256	malonate carr	0.118530596	-0.098849027	233582	233986	+	[illegible]	[illegible]		[illegible]	8	AAAAACAA	11	19	AAAAACAA
PA14_15780	PGD1653359		PGD1653360	PTS system N	0.191297309	-0.09576786	1343444	1345156	-	[illegible]	[illegible]		[illegible]	8	AAGAACAA	27	35	AAGAACAA
PA14_58650	PGD1660359		PGD1660360	chemotaxis tr	0.162527591	-0.080977095	5225436	5227457	-	[illegible]	[illegible]		[illegible]	8	AAGAACAA	27	35	AAGAACAA
PA14_69370	PGD1662122	algP	PGD1662123	alginate regul	-0.39414573	-0.074831937	6187256	6188314	-	[illegible]	[illegible]		[illegible]	8	AACAAGAA	59	67	AACAAGAA
PA14_38850	PGD1657108	exaB	PGD1657109	cytochrome c!	-0.497973484	-0.040839509	3464290	3464727	-	[illegible]	[illegible]		[illegible]	8	AACAAGAA	53	61	AACAAGAA
PA14_47420	PGD1658498		PGD1658499	hypothetical p	-0.754494044	-0.031511779	4225361	4225636	-	[illegible]	[illegible]		[illegible]	8	AAGAAGAA	71	79	AAGAAGAA
PA14_62900	PGD1661096	greA	PGD1661097	transcription	0.143919984	-0.029362664	5611320	5611796	-	[illegible]	[illegible]		[illegible]	8	AAGAAGAA	93	101	AAGAAGAA
PA14_32060	PGD1656032	xylS	PGD1656033	transcriptiona	0.859656296	-0.024037801	2790219	2791175	+	[illegible]	[illegible]		[illegible]	8	AACAACAA	17	25	AACAACAA
PA14_51150	PGD1659106	mucK	PGD1659107	cis	-0.183640088	-0.022755721	4544567	4545922	-	[illegible]	[illegible]		[illegible]	8	AACAAGAA	40	48	AACAAGAA
PA14_69130	PGD1662084	glpT	PGD1662085	sn-glycerol-3-	0.207429394	-0.016255352	6165123	6166469	+	[illegible]	[illegible]		[illegible]	8	AACAACAA	30	38	AACAACAA
PA14_70290	PGD1662266	sphR	PGD1662267	Sphingosine-r	0.42852781	-0.012875851	6267659	6268729	-	[illegible]	[illegible]		[illegible]	8	AACAAGAA	12	20	AACAAGAA
PA14_45790	PGD1658220	fliN	PGD1658221	flagellar motc	-0.007213369	-0.0067447	4070205	4070678	-	[illegible]	[illegible]		[illegible]	8	AAGAAAAA	60	68	AAGAAAAA
PA14_58980	PGD1660423		PGD1660424	hypothetical p	-1.606580766	0	5255897	5256121	+	[illegible]	[illegible]		[illegible]	8	AAGAACAA	53	61	AAGAACAA
PA14_58040	PGD1660261		PGD1660262	hypothetical p	-0.52235172	0.000334419	5163500	5163895	-	[illegible]	[illegible]		[illegible]	8	AACAATAA	12	20	AACAATAA
PA14_69980	PGD1662220		PGD1662221	transcriptiona	0.3711989	0.002752055	6243236	6243784	-	[illegible]	[illegible]		[illegible]	8	AAGAATAA	28	36	AAGAATAA
PA14_22340	PGD1654437		PGD1654438	hypothetical p	1.054119292	0.003220026	1945626	1945937	+	[illegible]	[illegible]		[illegible]	8	AAAAACAA	43	51	AAAAACAA
PA14_39750	PGD1657242		PGD1657243	amino acid pe	0.827018174	0.012100942	3540887	3542119	+	[illegible]	[illegible]		[illegible]	8	AACAAGAA	42	50	AACAAGAA
PA14_36080	PGD1656668		PGD1656669	MFS transport	-0.291088318	0.017913701	3213855	3215177	-	[illegible]	[illegible]		[illegible]	8	AACAACAA	28	36	AACAACAA
PA14_66820	PGD1661714	phaC1	PGD1661715	poly(3-hydrox	-0.036732009	0.035748591	5966750	5968429	+	[illegible]	[illegible]		[illegible]	8	AACAATAA	62	70	AACAATAA
PA14_26020	PGD1655043		PGD1655044	aminopeptida	-0.656150602	0.041149698	2273468	2275078	-	[illegible]	[illegible]		[illegible]	8	AACAAGAA	56	64	AACAAGAA
PA14_52990	PGD1659424	phhA	PGD1659425	phenylalanine	-0.373096753	0.044161368	4698197	4698985	+	[illegible]	[illegible]		[illegible]	8	AACAACAA	7	15	AACAACAA
PA14_49150	PGD1658772		PGD1658773	transcriptiona	-0.046038622	0.056823578	4364836	4365837	+	[illegible]	[illegible]		[illegible]	8	AACAACAA	32	40	AACAACAA
PA14_21340	PGD1654263	fadD2	PGD1654264	long-chain-fat	-0.023867502	0.057613625	1848101	1849789	+	[illegible]	[illegible]		[illegible]	8	AAGAATAA	42	50	AAGAATAA
PA14_35330	PGD1656544		PGD1656545	2-ketoglucona	0.935784787	0.059383808	3140930	3142237	-	[illegible]	[illegible]		[illegible]	8	AAGAACAA	18	26	AAGAACAA
PA14_52000	PGD1659262		PGD1659263	hypothetical p	0.249940467	0.060675006	4614957	4615376	+	[illegible]	[illegible]		[illegible]	8	AAGAACAA	59	67	AAGAACAA
PA14_08680	PGD1652229	tufB	PGD1652230	elongation fac	0.208836414	0.062971461	741311	742504	+	[illegible]	[illegible]		[illegible]	8	AAGAAAAA	57	65	AAGAAAAA
PA14_22910	PGD1654533	edd	PGD1654534	phosphogluco	0.797264507	0.068057542	1986649	1988475	+	[illegible]	[illegible]		[illegible]	8	AAAAACAA	14	22	AAAAACAA
PA14_44980	PGD1658102		PGD1658103	TetR family tr	-0.506794106	0.070166391	4010323	4010973	+	[illegible]	[illegible]		[illegible]	8	AAGAACAA	36	44	AAGAACAA
PA14_02470	PGD1651229	pntB	PGD1651230	pyridine nucle	-0.097514715	0.100585343	222339	223775	+	[illegible]	[illegible]		[illegible]	8	AAGAAGAA	18	26	AAGAAGAA
PA14_17320	PGD1653613	eno	PGD1653614	phosphopyruv	0.366053171	0.110916237	1488044	1489333	+	[illegible]	[illegible]		[illegible]	8	AACAAGAA	43	51	AACAAGAA
PA14_44800	PGD1658072		PGD1658073	transporter	0.79049506	0.117529392	3992783	3994132	+	[illegible]	[illegible]		[illegible]	8	AACAACAA	21	29	AACAACAA
PA14_41790	PGD1657572		PGD1657573	hypothetical p	0.703665318	0.120666142	3729757	3730194	-	[illegible]	[illegible]		[illegible]	8	AATAACAA	43	51	AATAACAA
PA14_70930	PGD1662370		PGD1662371	hypothetical p	0.23721013	0.122246608	6319833	6320237	+	[illegible]	[illegible]		[illegible]	8	AACAAGAA	63	71	AACAAGAA
PA14_39460	PGD1657196		PGD1657197	hypothetical p	0.551841536	0.127811652	3510492	3511631	+	[illegible]	[illegible]		[illegible]	8	AAGAATAA	2	10	AAGAATAA
PA14_54700	PGD1659706		PGD1659707	beta-lactama:	0.131566898	0.133204171	4849172	4851148	-	[illegible]	[illegible]		[illegible]	8	AACAATAA	29	37	AACAATAA
PA14_60730	PGD1660736		PGD1660737	outer membra	0.312356836	0.140907685	5411518	5412909	-	[illegible]	[illegible]		[illegible]	8	AACAAAAA	11	19	AACAAAAA
PA14_09610	PGD1652385		PGD1652386	hypothetical p	-0.03981551	0.146979624	826064	826930	-	[illegible]	[illegible]		[illegible]	8	AAGAACAA	32	40	AAGAACAA
PA14_46910	PGD1658410		PGD1658411	ABC transport	0.085463174	0.149124688	4177924	4178832	+	[illegible]	[illegible]		[illegible]	8	AATAAAAA	17	25	AATAAAAA
PA14_67310	PGD1661798		PGD1661799	amino acid pe	-0.222145589	0.157332289	6010690	6012093	-	[illegible]	[illegible]		[illegible]	8	AACAACAA	27	35	AACAACAA
PA14_23540	PGD1654639	act	PGD1654640	transcriptiona	0.208805895	0.162604947	2050430	2051350	+	[illegible]	[illegible]		[illegible]	8	AACAATAA	43	51	AACAATAA
PA14_06960	PGD1651937		PGD1651938	hypothetical p	-0.429140063	0.175481482	603247	604536	+	[illegible]	[illegible]		[illegible]	8	AAAAATAA	17	25	AAAAATAA
PA14_58375	PGD1660315	mdpA	PGD1660316	metallopeptid	0.113795618	0.181239717	5198442	5199659	-	[illegible]	[illegible]		[illegible]	8	AACAACAA	42	50	AACAACAA
PA14_18610	PGD1653811	argF	PGD1653812	ornithine carb	0.143445193	0.218847629	1598184	1599101	-	[illegible]	[illegible]		[illegible]	8	AAAAATAA	29	37	AAAAATAA
PA14_69320	PGD1662114		PGD1662115	integral meml	0.72518226	0.22090048	6183475	6184233	-	[illegible]	[illegible]		[illegible]	8	AACAAAAA	25	33	AACAAAAA
PA14_37590	PGD1656912	kynB	PGD1656913	kynurenine fo	-0.144844216	0.22455525	3345406	3346047	+	[illegible]	[illegible]		[illegible]	8	AAAAACAA	43	51	AAAAACAA
PA14_38140	PGD1656994		PGD1656995	glutamine syr	-0.339571456	0.243587977	3402135	3403511	-	[illegible]	[illegible]		[illegible]	8	AACAAAAA	30	38	AACAAAAA
PA14_65880	PGD1661568		PGD1661569	two-compone	0.311659412	0.252170056	5869705	5870439	-	[illegible]	[illegible]		[illegible]	8	AACAAGAA	13	21	AACAAGAA
PA14_17530	PGD1653649	recA	PGD1653650	recombinase	0.297189357	0.262828928	1505405	1506445	+	[illegible]	[illegible]		[illegible]	8	AACAAGAA	59	67	AACAAGAA
PA14_53190	PGD1659456	bolA	PGD1659457	morphogene	0.029070312	0.270534311	4715309	4715614	-	[illegible]	[illegible]		[illegible]	8	AAGAAAAA	25	33	AAGAAAAA
PA14_32240	PGD1656056	catA	PGD1656057	catechol 1	0.478762995	0.271841867	2801756	2802688	+	[illegible]	[illegible]		[illegible]	8	AACAACAA	27	35	AACAACAA
PA14_15790	PGD1653361		PGD1653362	phosphoenolp	-0.34875892	0.281183982	1345181	1347709	-	[illegible]	[illegible]		[illegible]	8	AACAACAA	53	61	AACAACAA
PA14_27000	PGD1655189		PGD1655190	chemotaxis tr	-0.303091852	0.283478268	2348301	2349773	-	[illegible]	[illegible]		[illegible]	8	AAGAAGAA	34	42	AAGAAGAA
PA14_24445	PGD1654785	gdhB	PGD1654786	NAD-depende	-0.141022485	0.287753929	2130044	2134906	+	[illegible]	[illegible]		[illegible]	8	AATAACAA	24	32	AATAACAA
PA14_50570	PGD1659004		PGD1659005	hypothetical p	0.228099711	0.292737254	4492050	4494344	-	[illegible]	[illegible]		[illegible]	8	AATAAGAA	9	17	AATAAGAA
PA14_01360	PGD1651063		PGD1651064	hypothetical p	1.049585534	0.30447811	131135	132208	+	[illegible]	[illegible]		[illegible]	8	AAGAAAAA	31	39	AAGAAAAA
PA14_67130	PGD1661766		PGD1661767	ABC transport	0.584890856	0.328356837	5992334	5993233	-	[illegible]	[illegible]		[illegible]	8	AAGAAAAA	26	34	AAGAAAAA
PA14_46110	PGD1658270		PGD1658271	sodium:solute	1.188044591	0.351095998	4100014	4101405	+	[illegible]	[illegible]		[illegible]	8	AACAACAA	14	22	AACAACAA
PA14_26230	PGD1655075	hisQ	PGD1655076	histidine trans	0.632064741	0.352445076	2289187	2289876	-	[illegible]	[illegible]		[illegible]	8	AACAACAA	27	35	AACAACAA
PA14_25270	PGD1654919	aroP1	PGD1654920	aromatic amir	0.56285478	0.353900249	2210177	2211586	+	[illegible]	[illegible]		[illegible]	8	AACAAGAA	14	22	AACAAGAA
PA14_13780	PGD1653041	narG	PGD1653042	respiratory nit	0.868347002	0.366273994	1183607	1187392	+	[illegible]	[illegible]		[illegible]	8	AAGAAGAA	83	91	AAGAAGAA
PA14_36120	PGD1656676		PGD1656677	MFS transport	1.134134252	0.368367595	3218748	3220073	-	[illegible]	[illegible]		[illegible]	8	AACAACAA	25	33	AACAACAA
PA14_10960	PGD1652597		PGD1652598	ferredoxin oxi	-0.158203769	0.371996883	947387	948499	-	[illegible]	[illegible]		[illegible]	8	AAGAACAA	27	35	AAGAACAA
PA14_17960	PGD1653719	glpK	PGD1653720	glycerol kinas	0.728025434	0.374371874	1542808	1544325	-	[illegible]	[illegible]		[illegible]	8	AACAAGAA	65	73	AACAAGAA
PA14_24010	PGD1654709	xcpS	PGD1654710	general secre	0.079508865	0.409151213	2086376	2087593	+	[illegible]	[illegible]		[illegible]	8	AAGAATAA	43	51	AAGAATAA
PA14_09750	PGD1652403		PGD1652404	oxidoreductas	0.267401299	0.414847625	839129	840448	-	[illegible]	[illegible]		[illegible]	8	AATAACAA	26	34	AATAACAA
PA14_39980	PGD1657278	qscR	PGD1657279	transcriptiona	1.100219169	0.415123733	3562214	3562927	-	[illegible]	[illegible]		[illegible]	8	AATAACAA	91	99	AATAACAA
PA14_66875	PGD1661722	phaF	PGD1661723	polyhydroxyal	-0.124310127	0.418402071	5972142	5973071	-	[illegible]	[illegible]		[illegible]	8	AAGAAGAA	59	67	AAGAAGAA
PA14_70040	PGD1662226	dadA	PGD1662227	D-amino acid	-0.225020436	0.423980238	6245427	6246725	-	[illegible]	[illegible]		[illegible]	8	AATAACAA	7	15	AATAACAA
PA14_02890	PGD1651293		PGD1651294	hypothetical p	0.573189277	0.434817451	253792	254625	+	[illegible]	[illegible]		[illegible]	8	AACAACAA	21	29	AACAACAA
PA14_11210	PGD1652643		PGD1652644	amino acid pe	0.885648746	0.442355286	975501	976991	+	[illegible]	[illegible]		[illegible]	8	AACAACAA	14	22	AACAACAA
PA14_07870	PGD1652083		PGD1652084	ABC transport	0.36439283	0.445621687	678769	679815	+	[illegible]	[illegible]		[illegible]	8	AACAATAA	43	51	AACAATAA
PA14_71850	PGD1662518		PGD1662519	hypothetical p	0.937540733	0.456084617	6400979	6403831	-	[illegible]	[illegible]		[illegible]	8	AAGAACAA	36	44	AAGAACAA
PA14_23160	PGD1654569	gltS	PGD1654570	sodium/gluta	-0.100696709	0.463481554	2006575	2007789	-	[illegible]	[illegible]		[illegible]	8	AACAACAA	30	38	AACAACAA
PA14_31800	PGD1656002		PGD1656003	sodium:alanir	0.442393547	0.476439086	2765588	2766937	+	[illegible]	[illegible]		[illegible]	8	AACAAGAA	30	38	AACAAGAA
PA14_67510	PGD1661828	estA	PGD1661829	esterase EstA	-0.291293768	0.48735247	6026551	6028491	-	[illegible]	[illegible]		[illegible]	8	AAAAACAA	26	34	AAAAACAA
PA14_11240	PGD1652647		PGD1652648	DNA-binding	0.4406681	0.487491905	977705	978655	+	[illegible]	[illegible]		[illegible]	8	AACAATAA	13	21	AACAATAA
PA14_28970	PGD1655533		PGD1655534	hypothetical p	-0.610775947	0.48879888	2504847	2505386	+	[illegible]	[illegible]		[illegible]	8	AAGAAGAA	81	89	AAGAAGAA
PA14_05590	PGD1651733	metF	PGD1651734	5	0.152664822	0.514599332	495947	496819	-	[illegible]	[illegible]		[illegible]	8	AACAAGAA	31	39	AACAAGAA
PA14_21780	PGD1654337		PGD1654338	transporter	0.259698162	0.514732922	1894225	1895160	-	[illegible]	[illegible]		[illegible]	8	AATAACAA	33	41	AATAACAA
PA14_08830	PGD1652255	tufA	PGD1652256	elongation fac	0.147910869	0.51633559	757816	759009	+	[illegible]	[illegible]		[illegible]	8	AAAAACAA	6	14	AAAAACAA
PA14_47120	PGD1658446		PGD1658447	hypothetical p	1.189203901	0.5169292	4198236	4198748	-	[illegible]	[illegible]		[illegible]	8	AAGAAGAA	62	70	AAGAAGAA
PA14_70600	PGD1662314		PGD1662315	HU family DN	1.249509411	0.585848869	6290069	6290341	-	[illegible]	[illegible]		[illegible]	8	AAAAACAA	21	29	AAAAACAA
PA14_28180	PGD1655389		PGD1655390	hypothetical p	-0.126580067	0.586348212	2439277	2440560	-	[illegible]	[illegible]		[illegible]	8	AAAAATAA	26	34	AAAAATAA
PA14_65280	PGD1661484	hflK	PGD1661485	protease subu	0.336212349	0.589245077	5817176	5818378	-	[illegible]	[illegible]		[illegible]	8	AACAACAA	80	88	AACAACAA
PA14_28400	PGD1655431		PGD1655432	outer membra	0.090321687	0.592655918	2452624	2453901	-	[illegible]	[illegible]		[illegible]	8	AACAATAA	41	49	AACAATAA
PA14_03860	PGD1651455		PGD1651456	glutamine syr	0.199117848	0.603481828	347204	348580	-	[illegible]	[illegible]		[illegible]	8	AACAAAAA	30	38	AACAAAAA
PA14_38860	PGD1657110	exaA	PGD1657111	quinoprotein	-0.023060396	0.622927285	3465040	3466911	+	[illegible]	[illegible]		[illegible]	8	AACAAGAA	55	63	AACAAGAA
PA14_14390	PGD1653133		PGD1653134	ABC-type tran	0.076976334	0.645801215	1227836	1228813	-	[illegible]	[illegible]		[illegible]	8	AAGAATAA	65	73	AAGAATAA
PA14_06190	PGD1651817		PGD1651818	glutathione S-	0.394682178	0.660094857	546995	547744	+	[illegible]	[illegible]		[illegible]	8	AACAAGAA	29	37	AACAAGAA
PA14_43170	PGD1657804		PGD1657805	bile acid/Na+	0.294160093	0.666409365	3845527	3846420	-	[illegible]	[illegible]		[illegible]	8	AACAATAA	43	51	AACAATAA
PA14_38560	PGD1657062		PGD1657063	MFS transport	0.260547596	0.718233169	3438790	3440142	+	[illegible]	[illegible]		[illegible]	8	AATAAGAA	26	34	AATAAGAA
PA14_53470	PGD1659500		PGD1659501	acetate kinas	-0.311698888	0.718467284	4738824	4740008	+	[illegible]	[illegible]		[illegible]	8	AAGAAGAA	22	30	AAGAAGAA
PA14_32530	PGD1656106		PGD1656107	cytochrome c	-0.297435578	0.722434893	2825911	2826564	+	[illegible]	[illegible]		[illegible]	8	AAAAACAA	20	28	AAAAACAA
PA14_32640	PGD1656122		PGD1656123	hypothetical p	-0.490599664	0.761126684	2834255	2835466	-	[illegible]	[illegible]		[illegible]	8	AACAACAA	29	37	AACAACAA
PA14_18820	PGD1653849		PGD1653850	hypothetical p	1.914503738	0.765578636	1622352	1623338	+	[illegible]	[illegible]		[illegible]	8	AACAAGAA	29	37	AACAAGAA
PA14_38690	PGD1657080		PGD1657081	acetoacetyl-C	0.241410612	0.76696292	3449781	3451736	+	[illegible]	[illegible]		[illegible]	8	AACAACAA	29	37	AACAACAA
PA14_16870	PGD1653539		PGD1653540	ABC transport	0.314527805	0.797660975	1446019	1446942	+	[illegible]	[illegible]		[illegible]	8	AACAAGAA	58	66	AACAAGAA
PA14_32660	PGD1656126		PGD1656127	MFS family tra	-0.006770405	0.808484765	2836165	2837511	-	[illegible]	[illegible]		[illegible]	8	AACAACAA	68	76	AACAACAA
PA14_37130	PGD1656844		PGD1656845	hypothetical p	0.028789277	0.813487675	3308261	3309397	-	[illegible]	[illegible]		[illegible]	8	AATAACAA	31	39	AATAACAA
PA14_50770	PGD1659042		PGD1659043	transporter	0.819372944	0.817356788	4510257	4511615	-	[illegible]	[illegible]		[illegible]	8	AACAACAA	31	39	AACAACAA
PA14_67040	PGD1661752		PGD1661753	ABC transport	0.36703267	0.860076635	5983588	5984550	-	[illegible]	[illegible]		[illegible]	8	AAGAAGAA	56	64	AAGAAGAA
PA14_54040	PGD1659602		PGD1659603	amino acid pe	0.320416895	0.864635246	4791877	4793292	-	[illegible]	[illegible]		[illegible]	8	AATAACAA	27	35	AATAACAA
PA14_63060	PGD1661122	smpB	PGD1661123	SsrA-binding	0.427047295	0.868405031	5625711	5626190	+	[illegible]	[illegible]		[illegible]	8	AACAAGAA	92	100	AACAAGAA
PA14_02370	PGD1651213		PGD1651214	porin	1.491086867	0.877718821	212285	213643	-	[illegible]	[illegible]		[illegible]	8	AACAATAA	42	50	AACAATAA
PA14_26750	PGD1655151		PGD1655152	hypothetical p	0.214131101	0.878976259	2329721	2331523	-	[illegible]	[illegible]		[illegible]	8	AAGAACAA	43	51	AAGAACAA
PA14_18120	PGD1653745	mmsA	PGD1653746	methylmalona	0.813190865	0.915423489	1554529	1556022	+	[illegible]	[illegible]		[illegible]	8	AACAATAA	13	21	AACAATAA
PA14_68280	PGD1661948		PGD1661949	dicarboxylate	0.223927649	0.917607834	6090376	6091008	+	[illegible]	[illegible]		[illegible]	8	AATAAGAA	27	35	AATAAGAA
PA14_34250	PGD1656378		PGD1656379	glycerophospl	-0.140317809	0.943455652	3044774	3045901	-	[illegible]	[illegible]		[illegible]	8	AAAAAGAA	25	33	AAAAAGAA
PA14_65840	PGD1661560		PGD1661561	enoyl-CoA hyc	0.922638489	0.982493964	5864030	5864821	-	[illegible]	[illegible]		[illegible]	8	AACAATAA	29	37	AACAATAA
PA14_10910	PGD1652589		PGD1652590	major facilitat	-0.389819485	0.986531202	943770	945029	-	[illegible]	[illegible]		[illegible]	8	AACAACAA	59	67	AACAACAA
PA14_13500	PGD1652997		PGD1652998	2-hydroxyacid	-0.167208783	1.002874517	1160401	1161378	-	[illegible]	[illegible]		[illegible]	8	AAAAAGAA	53	61	AAAAAGAA
PA14_46960	PGD1658418	ggt	PGD1658419	gamma-gluta	0.470622432	1.031123141	4181255	4182928	+	[illegible]	[illegible]		[illegible]	8	AACAATAA	29	37	AACAATAA
PA14_00620	PGD1650935		PGD1650936	hypothetical p	-0.052914027	1.034312684	61430	63091	-	[illegible]	[illegible]		[illegible]	8	AACAAGAA	28	36	AACAAGAA
PA14_11790	PGD1652739		PGD1652740	amino acid tra	0.746077508	1.047410321	1020800	1022248	-	[illegible]	[illegible]		[illegible]	8	AACAACAA	24	32	AACAACAA
PA14_31500	PGD1655958		PGD1655959	AMP-binding	0.23923179	1.060372711	2738366	2740033	+	[illegible]	[illegible]		[illegible]	8	AAAAACAA	26	34	AAAAACAA
PA14_12650	PGD1652865		PGD1652866	hypothetical p	0.884345403	1.0677591	1085653	1086387	-	[illegible]	[illegible]		[illegible]	8	AACAAAAA	24	32	AACAAAAA
PA14_26690	PGD1655143		PGD1655144	enoyl-CoA hyc	0.916822544	1.089901141	2325060	2325854	-	[illegible]	[illegible]		[illegible]	8	AAGAAGAA	15	23	AAGAAGAA
PA14_03920	PGD1651463	spuD	PGD1651464	polyamine tra	0.689678303	1.095038243	352562	353665	+	[illegible]	[illegible]		[illegible]	8	AACAAAAA	28	36	AACAAAAA
PA14_26260	PGD1655079		PGD1655080	hydrolase	-0.018484226	1.096503223	2290777	2291946	-	[illegible]	[illegible]		[illegible]	8	AACAAGAA	40	48	AACAAGAA
PA14_66760	PGD1661706		PGD1661707	hypothetical p	0.170098631	1.124561465	5963146	5963841	+	[illegible]	[illegible]		[illegible]	8	AAGAAGAA	56	64	AAGAAGAA
PA14_09010	PGD1652291	rplR	PGD1652292	50S ribosoma	0.45085515	1.127154016	766895	767245	+	[illegible]	[illegible]		[illegible]	8	AAGAAGAA	28	36	AAGAAGAA
PA14_54170	PGD1659620	putA	PGD1659621	bifunctional p	0.333945529	1.13710265	4802984	4806166	-	[illegible]	[illegible]		[illegible]	8	AATAACAA	19	27	AATAACAA
PA14_23030	PGD1654549	oprB	PGD1654550	glucose/carbo	2.6027413	1.142387562	1996676	1998040	+	[illegible]	[illegible]		[illegible]	8	AACAAGAA	59	67	AACAAGAA
PA14_24920	PGD1654865		PGD1654866	transcriptiona	-0.046452136	1.17479343	2177117	2178148	-	[illegible]	[illegible]		[illegible]	8	AAGAACAA	37	45	AAGAACAA
PA14_38130	PGD1656992		PGD1656993	amino acid pe	0.461064274	1.196306095	3400608	3401978	-	[illegible]	[illegible]		[illegible]	8	AAAAAGAA	21	29	AAAAAGAA
PA14_10260	PGD1652487		PGD1652488	dehydrogenas	2.026472614	1.223956329	883311	884285	-	[illegible]	[illegible]		[illegible]	8	AACAACAA	41	49	AACAACAA
PA14_10290	PGD1652493	acoR	PGD1652494	transcriptiona	1.764839716	1.224512042	886477	888354	+	[illegible]	[illegible]		[illegible]	8	AACAACAA	20	28	AACAACAA
PA14_01620	PGD1651103	aptA	PGD1651104	beta alanine--	1.047032251	1.275705519	147341	148687	-	[illegible]	[illegible]		[illegible]	8	AACAAGAA	25	33	AACAAGAA
PA14_10850	PGD1652581		PGD1652582	dehydrogenas	1.001457025	1.336562164	938752	940431	-	[illegible]	[illegible]		[illegible]	8	AAAAAGAA	1	9	AAAAAGAA
PA14_54150	PGD1659618	putP	PGD1659619	sodium/prolin	0.661898773	1.354838234	4801152	4802672	-	[illegible]	[illegible]		[illegible]	8	AAAAACAA	25	33	AAAAACAA
PA14_54630	PGD1659694		PGD1659695	acyl-CoA dehy	-0.006277336	1.384143188	4843462	4844625	+	[illegible]	[illegible]		[illegible]	8	AAGAACAA	69	77	AAGAACAA
PA14_24790	PGD1654841		PGD1654842	outer membra	1.085992672	1.395325109	2167408	2168673	-	[illegible]	[illegible]		[illegible]	8	AATAACAA	43	51	AATAACAA
PA14_54540	PGD1659678		PGD1659679	hypothetical p	0.487065439	1.478424241	4835069	4836052	+	[illegible]	[illegible]		[illegible]	8	AACAAGAA	26	34	AACAAGAA
PA14_10250	PGD1652485	acoB	PGD1652486	acetoin catab	3.01020927	1.510922266	882259	883278	-	[illegible]	[illegible]		[illegible]	8	AACAAGAA	28	36	AACAAGAA
PA14_65940	PGD1661574		PGD1661575	oxidoreductas	0.995157281	1.543818845	5872546	5874492	+	[illegible]	[illegible]		[illegible]	8	AACAACAA	28	36	AACAACAA
PA14_01580	PGD1651097	gabP	PGD1651098	gamma-amino	0.908690346	1.557918289	143881	145308	-	[illegible]	[illegible]		[illegible]	8	AAGAACAA	26	34	AAGAACAA
PA14_11000	PGD1652605	hpaA	PGD1652606	4-hydroxyphe	1.461250428	1.601741238	951044	952606	-	[illegible]	[illegible]		[illegible]	8	AACAACAA	27	35	AACAACAA
PA14_16410	PGD1653465		PGD1653466	MFS transport	1.255526903	1.711643117	1403759	1405378	+	[illegible]	[illegible]		[illegible]	8	AACAATAA	13	21	AACAATAA
PA14_65920	PGD1661572		PGD1661573	hypothetical p	1.556107823	1.865308985	5871281	5872372	-	[illegible]	[illegible]		[illegible]	8	AACAAGAA	27	35	AACAAGAA
PA14_63080	PGD1661126	lldP	PGD1661127	L-lactate perm	1.257381426	1.976791918	5627315	5628997	+	[illegible]	[illegible]		[illegible]	8	AACAACAA	25	33	AACAACAA
PA14_18150	PGD1653749		PGD1653750	acetyl-coa syr	2.466339628	2.016754732	1557036	1558922	+	[illegible]	[illegible]		[illegible]	8	AATAAGAA	24	32	AATAAGAA
PA14_53050	PGD1659436	aroP2	PGD1659437	aromatic amir	1.089099999	2.04115953	4702982	4704400	-	[illegible]	[illegible]		[illegible]	8	AACAATAA	17	25	AACAATAA
PA14_10280	PGD1652491		PGD1652492	short-chain de	1.946501037	2.099360103	885345	886145	-	[illegible]	[illegible]		[illegible]	8	AAGAACAA	29	37	AAGAACAA
PA14_34420	PGD1656406		PGD1656407	maltose/mani	1.233817164	2.182664869	3061183	3062493	-	[illegible]	[illegible]		[illegible]	8	AACAATAA	0	8	AACAATAA
PA14_68720	PGD1662018		PGD1662019	hypothetical p	-0.306188669	2.183781046	6128146	6128535	+	[illegible]	[illegible]		[illegible]	8	AAGAAGAA	27	35	AAGAAGAA
PA14_58410	PGD1660321	opdP	PGD1660322	glycine-glutar	1.602156419	3.008478972	5202483	5203937	+	[illegible]	[illegible]		[illegible]	8	AACAATAA	29	37	AACAATAA
